# Transition Metal‐Free *N‐*Arylation of Amino Acid Esters with Diaryliodonium Salts

**DOI:** 10.1002/chem.202005351

**Published:** 2021-03-03

**Authors:** Gabriella Kervefors, Leonard Kersting, Berit Olofsson

**Affiliations:** ^1^ Department of Organic Chemistry Arrhenius Laboratory Stockholm University SE-106 91 Stockholm Sweden

**Keywords:** amino acids, arylation, diaryliodonium salts, hypervalent compounds, transition metal-free

## Abstract

A transition metal‐free approach for the *N*‐arylation of amino acid derivatives has been developed. Key to this method is the use of unsymmetric diaryliodonium salts with anisyl ligands, which proved important to obtain high chemoselectivity and yields. The scope includes the transfer of both electron deficient, electron rich and sterically hindered aryl groups with a variety of different functional groups. Furthermore, a cyclic diaryliodonium salt was successfully employed in the arylation. The *N*‐arylated products were obtained with retained enantiomeric excess.

## Graphical abstract

**A transition metal‐free** *N*‐arylation of amino esters is reported, where the use of unsymmetric aryl(anisyl) iodonium salts is key to obtain high yields. The scope includes arylation with electron deficient, electron rich, and sterically hindered aryl groups. Various functional groups are tolerated, and no racemization is generally observed.

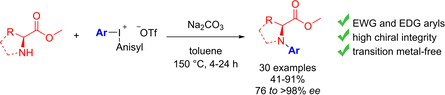

## Introduction

Amino acids are important building blocks in organic synthesis as they are a substantial part of the chiral pool. Functionalization of such species is attractive in drug discovery as a mean of obtaining novel compounds that can act as an affinity probe for enzyme studies^[1]^ or exhibit bioactivity.^[2]^ *N*‐Arylated amino acids are found as a core structure in biologically active compounds such as the protein kinase C (PKC) activator indolactam‐V^[3]^ and its analogue benzolactam‐V8,^[4]^ fibrinogen receptor antagonist SB 214857,^[5]^ and NMDA receptor antagonist L689560.^[6]^

**Scheme 1:**
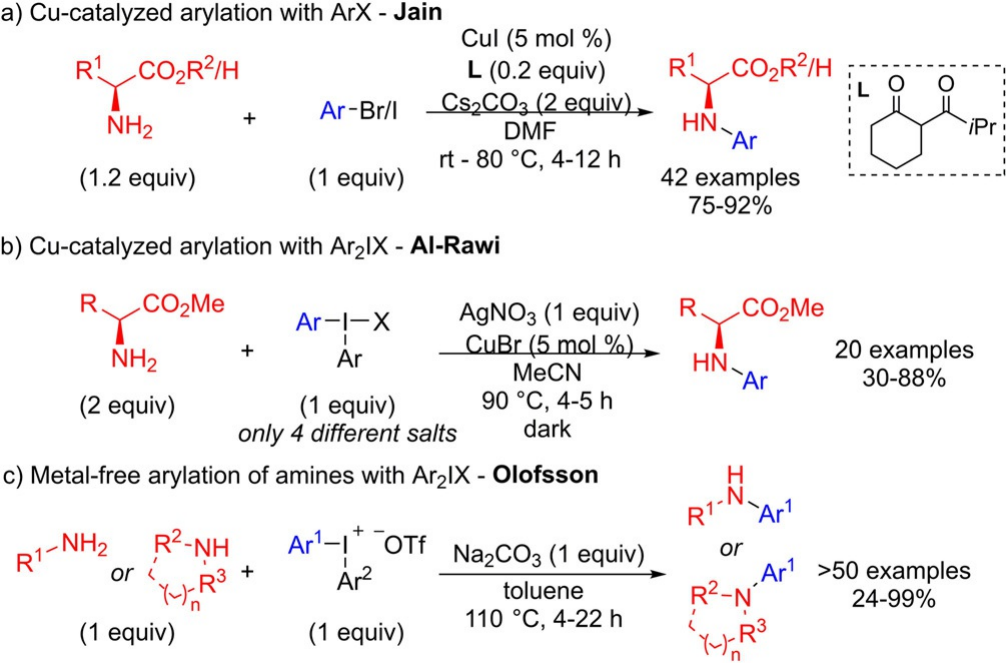
Recent *N*‐arylations of amino acid derivatives and amines.

Existing methods to reach *N*‐aryl amino acids are mostly based on transition metal catalysis.^[7]^ Cu‐catalyzed Ullman cross couplings have a good scope, although high Cu loading and extended reaction times at high temperatures or excess reagents are often needed.[^1c^, ^2a^, ^8^] Jain and co‐workers recently reported an *N*‐arylation under milder conditions with the use of a diketone ligand in DMF (Scheme 1 a).^[9]^ After adjustment of the reaction conditions, also heterocycles could be transferred.^[10]^ Alternatively, Cu‐catalyzed couplings with excess arylboronic acid can be performed at room temperature with a limited scope.^[11]^

Pd‐catalyzed Buchwald‐Hartwig arylations have been less explored, and early methods suffered from limited scope or partial racemization.^[12]^ Recent reports partly circumvent that problem through the use of advanced catalytic systems.^[13]^

The development transition metal‐free coupling reactions has received increasing attention to overcome drawbacks with transition metals, such as toxicity, cost, need for substrate‐dependent designer ligands, reaction sensitivity and the risk of product contamination in the pharmaceutical industry.^[14]^ Transition metal‐free syntheses of *N*‐aryl amino acids and their derivatives are, however, much less explored. Reported syntheses of specific targets include reactions with anilines and suitable electrophilic species through S_N_2 displacement or S_N_Ar.^[15]^ Reactions with enantiomerically enriched trichloromethyl alcohols provided *N*‐aryl amino amides through a Jocic‐type reaction.^[16]^ Alternatively, amino acid derivatives can be *N*‐arylated with the biobased reagent methyl‐3‐dehydroshikimate as aryl precursor.^[17]^ Triphenylsulfonium triflate can be employed in the *N*‐arylation of racemic amino acid derivatives in a reaction proceeding through arynes.^[18]^ A common feature with the methods above is that their scope is very limited.

Poelarends and co‐workers recently published an elegant biocatalytic approach to *N*‐arylated aspartic acids through enzymatic reactions of anilines with fumarate.^[19]^ Moderate to good yields with excellent *ee* were obtained, although sterically hindered or strongly electron deficient anilines were not tolerated.

Hypervalent iodine compounds have become efficient reagents for a wide variety of transition metal‐free reactions.^[20]^ Diaryliodonium salts have several attractive features including easy availability, high stability, and low toxicity. They are highly reactive electrophilic arylation reagents, and have been applied successfully in a variety of transition metal‐free *C*‐, *O*‐, *N*‐ and *S*‐arylations.^[21]^

Iodonium salts have been utilized to functionalize amino acid derivatives through fluoroalkylation,^[22]^ and a small set of diaryliodonium bromides were employed in a Cu‐catalyzed *N*‐arylation of amino acid esters, which required excess substrate and a stochiometric amount of AgNO_3_ (Scheme 1 b).^[23]^ We have recently reported a metal‐free *N*‐arylation of aliphatic amines with diaryliodonium salts under mild conditions (Scheme 1 c).^[24]^ The methodology has a broad amine scope, but proved inefficient for arylation of amino acids. To facilitate the access to enantiomerically enriched *N*‐arylated amino acid derivatives, we set out to develop a transition metal‐free *N*‐arylation of amino acids derivatives, and herein present our results.

## Results and Discussion

Phenylalanine methyl ester (**1 a**) was chosen as the model substrate, and was obtained by deprotonation of the corresponding hydrochloride salt **1 a‐HCl**. The free amine **1 a** proved to be unstable upon storage,[^23a^, ^25^] and was therefore prepared within 5 days of use.

An extensive optimization was performed,^[26]^ with initial screening of the arylation conditions using 4‐nitrophenyl(phenyl)iodonium triflate (**2 aa‐OTf**). The conditions used in our arylation of aliphatic amines^[24a]^ gave poor conversion into product **3 a** with substantial amounts of recovered **1 a** (Table 1, entry 1). The conversion was improved by increasing the temperature (entries 2,3), and the combination with excess iodonium salt resulted in 59 % yield of **3 a** (entry 4). Arylations with iodonium salt **2 aa‐OTf** generally give complete chemoselectivity,^[27]^ with transfer of only the nitroaryl group, but we observed small amounts of phenylated side‐product **3‐Ar^2^** and a deterioration of the overall mass balance when the reaction was performed at 150 °C.

**Table 1:** Optimization of the 4‐nitrophenylation of **1 a**.^[a]^

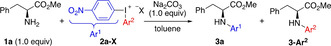							
Entry	Salt **2 a‐X**			*T*	*t*	Yield [%]^[b]^ of	
		Ar^2^	(equiv)	[°C]	[h]	**3 a**	**3‐Ar^2^**
1	**2 aa‐OTf**	Ph	1.0	110	22	16 (15)	0
2	**2 aa‐OTf**	Ph	1.0	130	22	(28)	0
3	**2 aa‐OTf**	Ph	1.0	150	4	47 (45)	(6)
4	**2 aa‐OTf**	Ph	2.0	150	4	59	12
5	**2 aa‐BF_4_**	Ph	2.0	150	4	34	14
6^[c]^	**2 aa‐OTs**	Ph	2.0	150	4	12 (14)	(12)
7^[d]^	**2 aa‐Br**	Ph	2.0	150	4	0	0
8	**2 ab‐OTf**	Mesityl	2.0	150	4	49	10
9	**2 ac‐OTf**	Anisyl	2.0	150	4	85 (79)	0
10	**2 ac‐OTf**	Anisyl	1.5	150	4	66	0
11	**2 ac‐OTf**	Anisyl	1.0^[e]^	150	4	73	0
12	**2 ac‐OTf**	Anisyl	2.0	130	4	78 (77)	0
13	**2 ac‐OTf**	Anisyl	2.0	110	24	60	0

[a] Reaction conditions: **1 a** (0.2 mmol, 1 equiv), **2 a‐X** (1–2 equiv) and base (1 equiv) were mixed under argon. Degassed, anhydrous toluene (1 mL) was added, and the reaction was heated in an oil bath with stirring. [b] ^1^H NMR yield with trimethoxybenzene (TMB) as internal standard, isolated yields given in parentheses. [c]>95 % **4‐OTs** formed. [d]>95 % **4‐Br** formed. [e] 2.0 equiv of **1 a** and base were used.

The reactivity of 4‐nitrophenyl(phenyl)iodonium salts with other anions (**2 aa‐X**) was next examined, and salts with tetrafluoroborate, tosylate or bromide anions proved to give inferior results (entries 5–7). In fact, iodonium salts **2 aa‐OTs** and **2 aa‐Br** suffered from a competing pathway where the anion acted as nucleophile to deliver the corresponding 4‐nitrophenyl tosylate (**4‐OTs**) and bromide (**4‐Br**), respectively. Such side‐products have previously been reported with diaryliodonium bromides,^[28]^ whereas reactions with diaryliodonium tosylates are often efficient also at elevated temperatures.[^24a^, ^28b^, ^29^]

Unsymmetric diaryliodonium salts are known to react with high chemoselectivity when they have sufficiently different electronic properties.^[30]^ The non‐transferable aryl group is called a “dummy group”, and the phenyl group is generally a sufficient dummy group in transfer of strongly electron‐deficient aryl groups.^[27]^ Since the phenylated side‐product was observed in this reaction, we investigated iodonium salts with other dummy groups to improve the chemoselectivity and simplify the isolation of product **3 a**. Reactions with mesityl salt **2 ab‐OTf** resulted in similar yield and chemoselectivity (entry 8). The anisyl moiety is often a good dummy group^[27]^ and salt **2 ac‐OTf** indeed reacted with complete chemoselectivity. More surprisingly, the conversion was also improved and **3 a** was isolated in 79 % yield as the only product (entry 9).

Further investigations with salt **2 ac‐OTf** showed that changes in reaction stoichiometry had a negative impact (entries 10, 11). Comparable results were obtained at 130 °C (entry 12), and the reaction could even be performed at 110 °C with this salt, illustrating the reactivity difference to salt **2 aa‐OTf** (entry 13 vs. 1). Importantly, the enantiomeric excess of product **3 a** was >98 %,^[26]^ demonstrating that the reaction conditions were mild enough to not cause racemization despite the high reaction temperature.

**Scheme 2:**
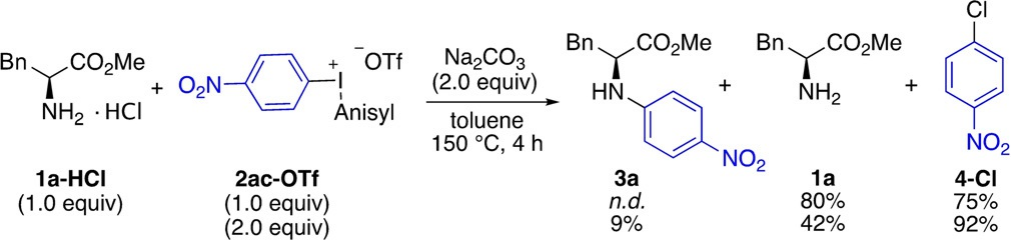
Attempted arylation of amino acid ester salt **1 a‐HCl** (^1^H NMR yields with TMB as internal standard).

We next examined whether the deprotonation of **1 a‐HCl** could be combined with the arylation to circumvent the handling of unstable amine **1 a. 1 a‐HCl** was thus reacted with salt **2 ac‐OTf** (1–2 equiv) in the presence of 2 equiv sodium carbonate (Scheme 2). However, only minor amounts of **3 a** were detected and the major product was instead **4‐Cl**, which formed when the released chloride anion acted as a competing nucleophile. The mass balance in the first reaction shows that **1 a** is rather stable at elevated temperatures once **2 ac‐OTf** is consumed, but partly decomposes in the presence of excess **2 ac‐OTf**, likely due to the high oxidation potential of the diaryliodonium salt.^[26]^

**Scheme 3:**
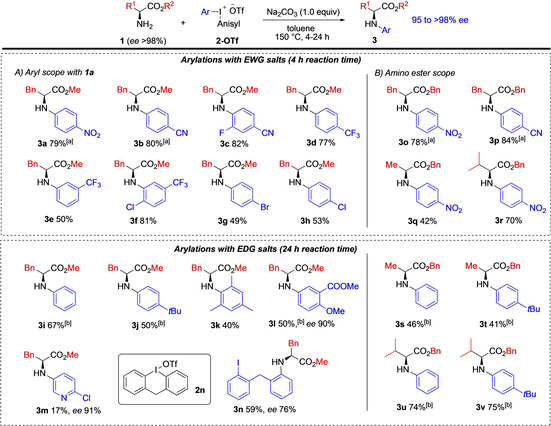
Arylation scope with amino acid esters **1**, *ee* values 95–98 % unless stated. [a] Reaction at 130 and 150 °C gave similar results. [b] Symmetric salt **2** (Ar^1^=Ar^2^) used.

The arylation scope of amino ester **1 a** with various aryl(anisyl)iodonium triflates **2‐OTf** was subsequently examined. Aryl groups with electron‐withdrawing groups (EWG) were efficiently transferred using the optimized conditions (Scheme 3 A), as exemplified by the synthesis of *p*‐CN substituted products **3 b** and **3 c**. Aryl groups with CF_3_ substituents were also well tolerated (**3 d**–**3 f**), where even the sterically encumbered product **3 f** was formed in high yield. Also halogen‐containing aryl moieties could be transferred to provide **3 f**–**3 h**. Iodonium salts with strong EWG reacted with similar efficiency at 130 and 150 °C while the other salts showed reduced activity at 130 °C.^[26]^

The transfer of aryls with electron‐donating groups (EDG) is generally more challenging in reactions with diaryliodonium salts.[^27a^, ^31^] We were hence pleased to see that such arylations were feasible by prolonging the reaction time to 24 h. In this fashion, the phenylated product **3 i** was isolated in 67 % yield, and a *tert‐*butyl‐substituted aryl moiety could also be transferred to provide **3 j**. The method demonstrated good compatibility with the sterically demanding mesityl group (**3 k**) and a highly functionalized aryl moiety could be transferred (**3 l**). A heteroaryliodonium salt was employed to give pyridyl product **3 m** in modest yield. Additionally, the 6‐membred cyclic diaryliodonium salt **2 n** could be used to generate the iodo‐substituted product **3 n** in 59 % yield. Reactions with cyclic diaryliodonium salts generally require transition metal catalysis due to decreased reactivity,^[32]^ and this result is conceptually important as reactions with such salts have higher atom efficiency and deliver products with a convenient iodine handle for further transformations.^[32d]^

The substrate scope of primary amino esters **1** was next examined. Arylation of benzyl ester **1 b** was first investigated and provided the arylated products **3 o** and **3 p** in equally high yields as the corresponding methyl ester **1 a** (cf. products **3 a**, **3 b**). Having demonstrated the compatibility of the benzyl protecting group, it was used to protect alanine (**1 c**) and valine (**1 d**), as their corresponding methyl esters (**1 e**, **1 f**) proved to be volatile and easily evaporated under vacuum or high temperatures. Arylation of the alanine and valine benzyl esters was successful providing **3 q** and **3 r** in moderate to good yields. Substrates **1 c** and **1 d** could also be arylated with less activated diaryliodonium salts, providing the phenylated and *tert*‐butyl‐substituted products **3 s**–**3 v**. The reactions with valine ester **1 d** gave consistently higher yields than the corresponding reactions with **1 c**, a trend that has also been reported in previous *N*‐arylations.[^2a^, ^12a^, ^13b^]

**Scheme 4:**
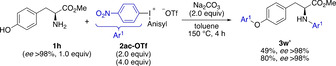
Diarylation of tyrosine methyl ester.

We next explored the compatibility with more challenging substrates, such as unprotected, heteroatom‐substituted amino acid esters. Heteroatom substituents are usually well tolerated in metal‐free arylations with diaryliodonium salts,^[21]^ but such substrates proved difficult to arylate under the current conditions. For example, reactions with tryptophan methyl ester (**1 g**) gave only 15 % product with **2 ac‐OTf**,^[26]^ which could be due to competing coordination of the indole nitrogen to the iodine(III) reagent. To the contrary, arylation of tyrosine methyl ester (**1 h**) delivered the diarylated product **3 w′** in high yield and retained *ee*, without detection of the corresponding monoarylated product **3 w** (Scheme 4).

To investigate the performance of the unsymmetric anisyl salts, a few products in the scope were also synthesized using the corresponding aryl(phenyl) salts or symmetric diaryliodonium salts. Reagents with the anisyl dummy generally performed best, as chemoselectivity problems were avoided and better arylation yields were obtained.^[26]^

SFC and HPLC analyses were performed to determine the *ee* of the products, since previously reported methods for *N*‐arylation of amino esters showed partial racemization[^7c^, ^12a^, ^13^, ^25c^] or had insufficient data to judge the enantiomeric purity.[^1c^, ^9^, ^10^, ^12b^] As expected, the current methodology generally left the existing stereocenter intact and the majority of the products were isolated with 95 to >98 % *ee*. Even when prolonged reaction time was applied in reactions with EDG salts, very little racemization occurred.

**Scheme 5:**
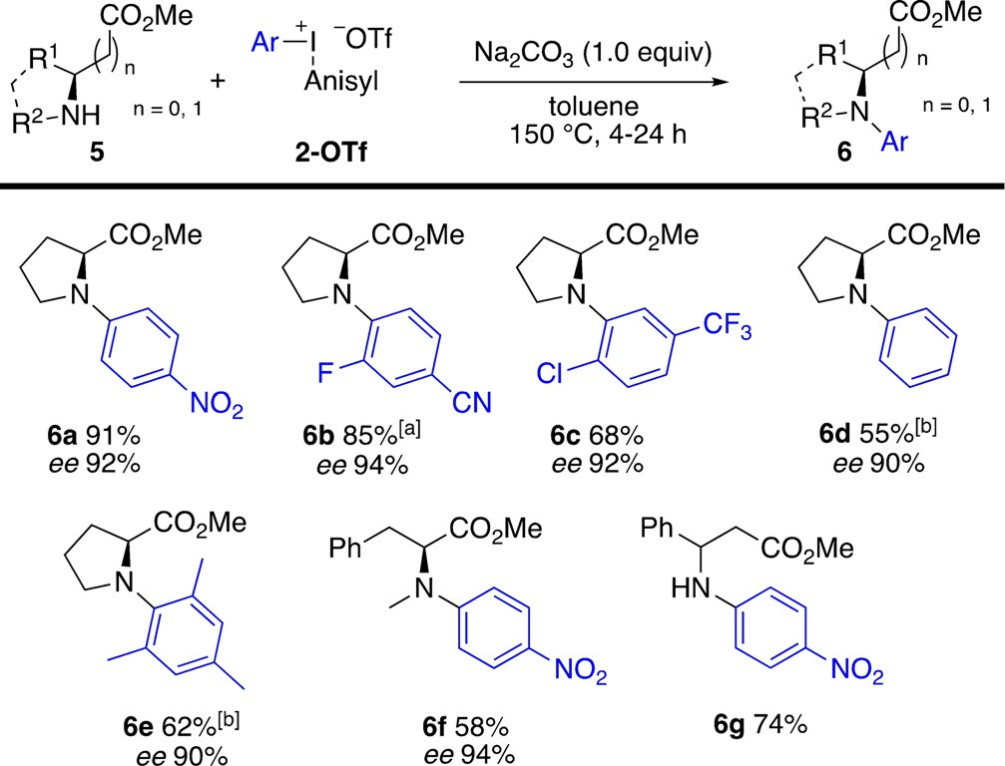
Arylation of amino acid esters **5**. [a] Reaction at 130 °C. [b] Symmetric salt **2** (Ar^1^=Ar^2^) used, 24 h reaction time.

Secondary amino esters were evaluated next, and proline methyl ester (**5 a**) proved to be a suitable substrate (Scheme 5). Arylation with electron‐withdrawing aryl moieties provided products **6 a‐**‐**6 c** in high yields. Upon prolonged reaction time, also phenylation (**6 d**) and mesitylation (**6 e**) was feasible, these transformations proved better with the symmetric iodonium salts than with the anisyl salts. *N*‐arylation of the *N*‐methylated α‐phenylalanine ester **5 b** resulted in 58 % yield of **6 f** with high enantiomeric excess, and β‐phenylalanine ester (**5 c**) gave the nitrophenylated product **6 g** in good yield. The SFC analysis of products **6** showed a small decrease of the enantiomeric purity (90–94 % *ee*).

*N*‐Protected derivatives of **1 a,b** remained largely untouched under the reaction conditions, which is useful in arylation of more complex substates. To this end, acetyl‐ and tosyl‐protected derivatives of **1 a** gave no arylation, whereas a Boc‐protected derivative of **1 b** gave 9 % of **3 o** with 82 % recovered substrate, meaning that the Boc‐group had been partially fragmented under the reaction conditions.^[26]^

A series of control experiments were performed to indicate a possible mechanism of the arylation.^[26]^ Running the reaction without argon atmosphere gave a significantly lower yield. Addition of DPE as radical trap did not affect the outcome of the reaction, indicating that a radical pathway is unlikely.^[33]^ Furthermore, reactions in the presence of furan (5 equiv) as aryne trap did not result in formation of Diels–Alder adducts. Based on that experiment and the formation of only one regioisomeric product in reactions with substituted diaryliodonium salts, an aryne pathway can be excluded.^[33]^ Additionally, an attempted arylation of **1 a** with iodobenzene or 1‐iodo‐4‐nitrobenzene at 150 °C resulted in quantitative recovery of the starting materials, and an S_N_Ar pathway with the iodoarene formed from **2 aa‐OTf** can therefore be ruled out.

**Scheme 6:**
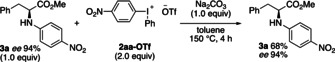
Stability test of **3 a**.

Finally, the product stability was investigated by subjecting **3 a** to the reaction conditions (Scheme 6). No diarylated product was formed and **3 a** could be recovered in 68 % yield. The *ee* of **3 a** remained intact, demonstrating that the arylated product is stable to racemization under the reaction conditions. However, partial decomposition of **3 a** had occurred, which explains the mass balance problems observed in some reactions. A stability test of **2 ac‐OTf** in the absence of any amino acid ester also showed partial decomposition at 150 °C.^[26]^

**Scheme 7:**
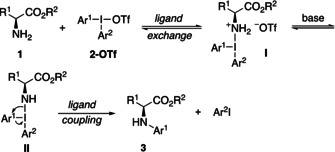
Suggested mechanism.

Based on the experiments described above, we suggest that the reaction follows a traditional ligand coupling pathway, where the amine does a ligand exchange with the triflate to give intermediate **I**, followed by deprotonation to **II** and ligand coupling to yield product **3** (Scheme 7).

## Conclusions

A transition metal‐free *N*‐arylation of amino acid derivatives with diaryliodonium salts has been presented. The moderate reactivity of the substrates was overcome by increasing the reaction temperature, which initially resulted in a chemoselectivity problem. The method has a broad arylation scope and is compatible with transfer of both electron deficient and electron rich aryl groups in good to high yields. *ortho*‐Substituents and various functional groups are well tolerated, including a cyclic diaryliodonium salt. The amino acid ester scope includes primary and secondary α‐amino esters, as well as β‐amino esters, and the products were generally obtained with excellent enantiomeric excess. The use of aryl(anisyl)iodonium triflates proved to be key to increase both the reactivity and chemoselectivity of the process. This interesting observation will be further studied in reactions with related nucleophiles.

## Experimental Section

**Arylation of amino acid esters 1 and 5**: Amino acid ester **1** (0.2 mmol), salt **2** (0.4 mmol, 2.0 equiv) and Na_2_CO_3_ (0.2 mmol, 1.0 equiv) were added to an oven‐dried, pressure‐stable microwave vial and dried under vacuum for 15 min. The vial was flushed with argon 3–4 times followed by the addition of anhydrous toluene (1 mL, degassed by bubbling with argon for 20 min). The vial was added to a preheated oil bath at 150 °C, and stirred for 4–24 h. After completion, the reaction was cooled to rt and Celite was added. The volatiles were removed under reduced pressure, and the mixture was purified by column chromatography (SiO_2_ with pentane/EtOAc as eluent system), to provide product **3** or **6**. The enantiomeric purity was analyzed by Chiral SFC, Diacel OJ‐H, 25 °C, 0.3 cm Ø, 15 cm column, 10 % MeOH in CO_2_, flow rate: 0.8 mL min^−1^.

## Conflict of interest

The authors declare no conflict of interest.

## Supporting information

As a service to our authors and readers, this journal provides supporting information supplied by the authors. Such materials are peer reviewed and may be re‐organized for online delivery, but are not copy‐edited or typeset. Technical support issues arising from supporting information (other than missing files) should be addressed to the authors.

Supplementary
