# Supplementary material for: Transition Metal‐Free N‐Arylation of Amino Acid Esters with Diaryliodonium Salts
Source: Chemistry. 2021 Mar 3;27(18):5790–5. doi: 10.1002/chem.202005351 (PMC8048889; doi:10.1002/chem.202005351)
Supplement: Supplementary file 1 — Supplementary [file CHEM-27-5790-s001.pdf]

Chemistry–A European Journal

Supporting Information

Transition Metal-Free *N*-Arylation of Amino Acid Esters with Diaryliodonium Salts

Gabriella Kervefors, Leonard Kersting, and Berit Olofsson*[a]

1 General experiential procedure 2
2 Synthesis of Diaryliodonium Salts 3
2.1 Structure of Diaryliodonium Salts 3
2.2 Applying our One-Pot Methods 3
2.3 Synthesis of Novel Aryl(Anisyl) Iodonium Triflates 7
2.4 Other Methods used for Synthesis of Diaryliodonium Salts 10
3 Synthesis of Amino Acid Esters 11
3.1 Substrates Used in the Study 11
3.2 Synthesis of Amino Acid Methyl Ester HCl Salts 11
3.3 Synthesis of Amino Acid Benzyl Ester TFA Salts 12
3.4 Synthesis of Free Amino Acid Esters 14
3.5 N-Functionalization of Amino Acid Esters 17
4 Arylation of Amino Acid Esters 19
4.1 Optimization Studies 19
4.2 Arylation of Phenylalanine Ester Precursors 25
4.3 Chemoselectivity Study 26
4.4 Further Studies 29
4.5 Limitations 31
4.6 Preparation of Racemic Samples for ee Analysis 33
4.7 Substrate Scope of The Amino Acid Esters 34
4.8 Arylation of Primary Amino Acid Esters 35
4.9 DiArylation of Tyrosine Methyl Ester 52
4.10 Arylation of Secondary Amino Acid Esters 54
5 Refrences 61
6 NMR Spectra 62

1 GENERAL EXPERIENTIAL PROCEDURE

All reactions were carried out in non-dried glassware unless dry anhydrous solvents were used. Reactions run above the boiling point of the solvent were performed in pressure-stable microwave vials. All solvents and reagents were synthesized via literature protocols or purchased from commercial sources and used without further purifications. *m*CPBA (Aldrich, 77% active oxidant) was dried at rt on high vacuum for 2-4 h, and titrated by iodometric titration[1] prior to use. TfOH was stored and handled under argon, using Hamilton syringes and oven-dried metal syringes. All diaryliodonium salts were synthesized according to procedures described in **Section 1.2**. Amino acid derivatives were bought or synthesized according to protocols in literature (**Section 1.3**). TLC analysis was performed on pre-coated Merck silica gel 60 F254 plates using UV light. Column chromatography was conducted by flash column chromatography using 40-60 µm, 60 Å silica gel as stationary phase. Flash column chromatography was done on SiO_2 purchased from Aldrich (technical grade, 60 Å pore size, 230-400 mesh, 40-63 µm). Alternatively, automated flash system Teledyne ISCO CombiFlash Rf 200 with RediSep Rf columns was used. Melting points were measured using a STUART SMP3 and are reported uncorrected. The melting point measurements refer to the solidified materials as the result of the given experimental procedures, no additional recrystallization was done. All NMR spectra were recorded using a 400 or 500 MHz Bruker AVANCE II with a BBO probe at 298 K using $CDCl_3$, or DMSO-d_6 as solvents. Chemical shifts are given in ppm relative to the residual solvent peak (^1H NMR: $CDCl_3$ δ 7.26; DMSO-d_6 2.50; ^{13}C NMR: $CDCl_3$ δ 77.16; DMSO-d_6 39.52,) with multiplicity (br = broad, s = singlet, d = doublet, t = triplet, q = quartet, m = multiplet, app = apparent), coupling constants (in Hz) and integration. High resolution mass analyses were obtained using a Bruker microTOF ESI. Analytical data is given if the compound is novel or not fully characterized in the literature. Determination of Enantiomeric Purity was performed with chiral SFC or HPLC. SFC was performed using Chiralpak IA and OJ-H, columns (3.0 × 150 mm × 5 µm) eluting with MeOH/CO_2 and monitored by DAD (Diode Array Detector). HPLC analysis on chiral stationary phase was performed on an Agilent 1200-series instrument, employing Daicel Chiralpak columns IA, IB, ID, OJ-H and AD-H (4.6 x 250 mm).

2 SYNTHESIS OF DIARYLIODONIUM SALTS

2.1 STRUCTURE OF DIARYLIODONIUM SALTS

Diaryliodonium salts are hypervalent compounds, where the two aryl ligands have a bond angle of about 90°and the hypervalent bond is shared between the counterion (X), the iodine and the aryl moiety in the apical position (Ar-I-X). This structure is supported by X-ray analysis.[2]

In symmetric diaryliodonium salts, the position of the two aryl groups is indifferent and Ar-I-X is depicted covalently (**Figure S1a**). In unsymmetric diaryliodonium salts, we have chosen to depict the bond as ionic (**Figure S1b)** due to the rapid interchangeability of the two aryl moieties and as a result, both aryl ligands contribute to the hypervalent bond.[3]

Figure S1. Depictions of symmetric and unsymmetric diaryliodonium salts.

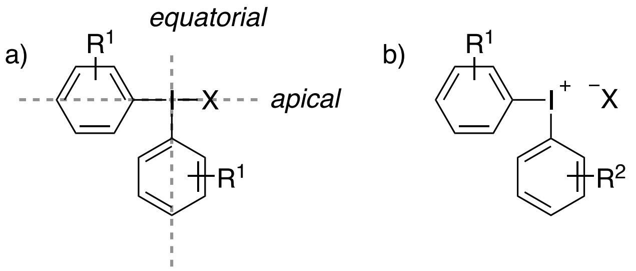

2.2 APPLYING OUR ONE-POT METHODS

2.2.1 One-Pot Methods for Synthesis of Diaryliodonium Salts

The general methods developed in our group were used for synthesis of diaryliodonium salts featured in the arylation of amino acid derivatives (**Table S1**). No precautions were taken to avoid air or moisture. In the synthesis of unsymmetric diaryliodonium salts, the substrates should be selected such that an aryl group with electron-withdrawing substituents is introduced as the ArI, and the other aryl group as ArH. See **Section 2.2.2** for synthetic details and references to analytical data.

Table S1. General methods to synthesize diaryliodonium salts.

**Method**	Scheme
**I**[4]	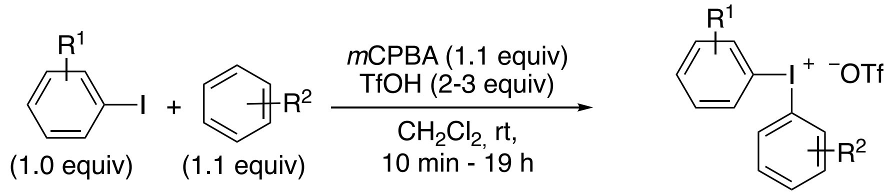
**II**[4]	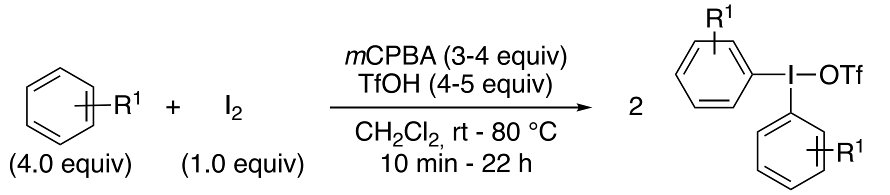

III[5]

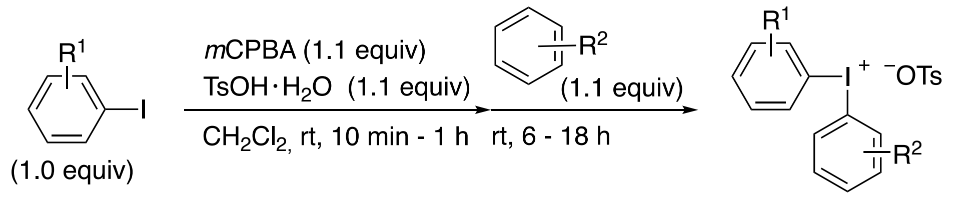

IV[6]

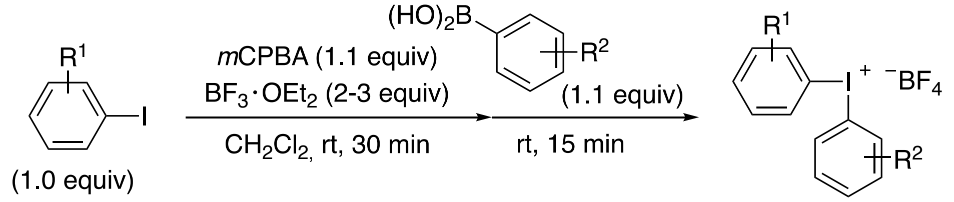

V[7]

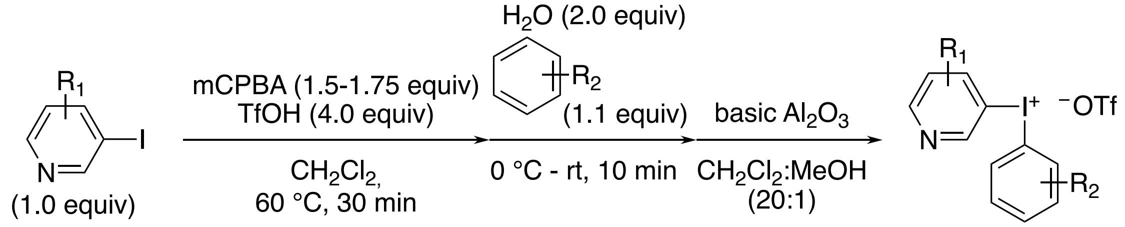

Method VI Anion exchange[8]

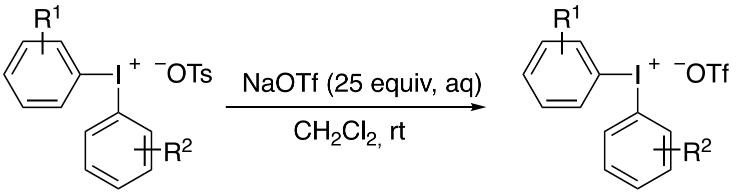

Procedure:

NaOTf (85 mmol, 25 equiv) was dissolved in H_2O (100 mL). Diaryliodonium salt (3.4 mmol, 1.0 equiv) was dissolved in CH_2Cl_2 (20 mL) and washed 5×20 mL with the aqueous solution. The organic layer was concentrated without drying. Et_2O was added and the mixture was stirred at rt for 30 min. The solid was filtered, washed with Et_2O and dried under vacuum.

2.2.2 Diaryliodonium Salts Synthesized through our One-Pot Methods

The diaryliodonium salts used in this study were synthesized as detailed in **Table S2**, with the numbering system explained in **Figure S2**.

Figure S2. Dummy groups used in the synthesis of iodonium salts.

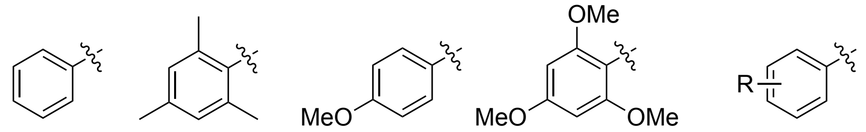

Phenyl **2xa** | Mesityl **2xb** | Anisyl **2xc** | TMP **2xd** | Other, in symmetric salts **2xe**

Table S2. Synthesis of reported diaryliodonium salts **2**.

Diaryliodonium salt		Method	Acid (equiv)	Temp. [°C]	Time [h]	Yield (%)	Ref.
**2aa-OTf**	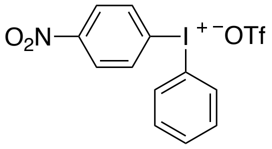	**I**	2.0	rt	18	88	[4], [9]
**2aa-BF4**	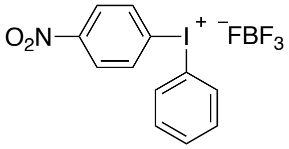	**IV**	2.6	rt	1) 60 min<br>2) 30 min	54	[10]
**2ac-OTs**	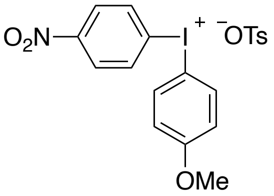	**III**[a]	1.0	rt	18	82	[11]
**2ba-OTf**	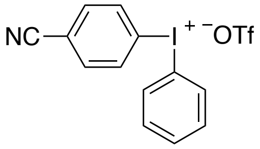	**I**	2.0	rt	18	79	[4], [9a]
**2ea-OTf**	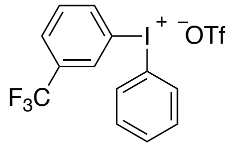	**I**	2.0	80[b]	15	63	[4b]
**2ec-OTf**	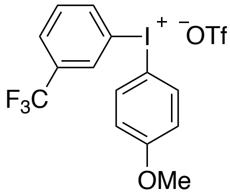	**III & VI**	1.0	1) 40<br>2) rt	1) 1<br>2) 24	**III** 89<br>**VI** 91	[9b], [12]
**2gc-OTf**	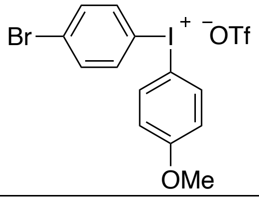	**I**	2.0	rt	1	68	[4b]

**2hc-OTf**	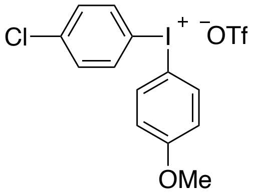	**I**	2.0	rt	1	76	[4b]
**2ia-OTf**	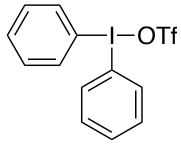	**I**	3.0	rt	30 min	95	[4]
**2ic-OTf**	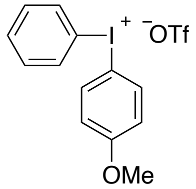	**III & VI**	1.0	rt	6	**III** 56<br>**VI** 97	[4]
**2je-OTf**	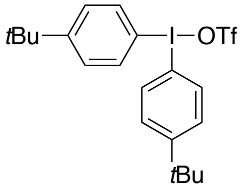	**II**	4.0	rt	1	78	[4a]
**2kb-OTf**	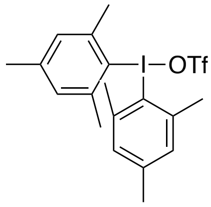	**II**	4.0	rt	1	52	[4]
**2kc-OTf**	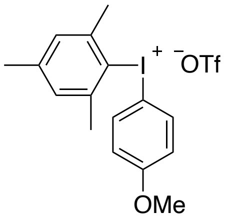	**III & VI**	1.0	rt	1.5	**III** 65<br>**VI** 91	[11b], [13]
**2le-OTf**	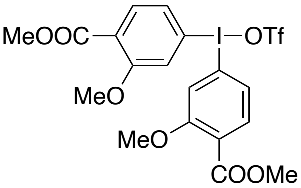	**II**	4.0	rt	6	92	[14]
**2mc-OTf**	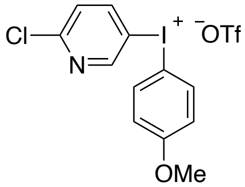	**I**	2.5	1) 0<br>2) rt	1) 15 min<br>2) 0.5	78	[4a]
**2n-OTf**	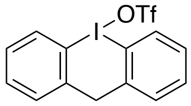	**I**	3.0	rt	22	97	[15]
**2o-OTf**	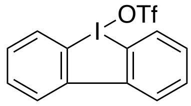	**I**	3.0	rt	1	90	[16]

**2pe-OTf**	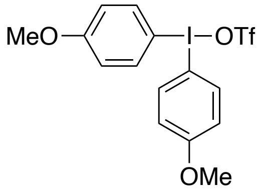	**III & VI**	4.0	1) rt<br>2) rt	1) 1<br>2) 15 min	**III** 89<br>**VI** 97	[5], [9b]
**2qc-OTf**	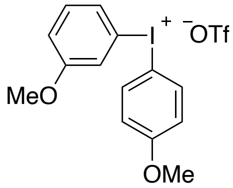	**III & VI**	2.0	rt	14 h	**III** 58<br>**VI** 67	[9a]
**2rc-OTf**	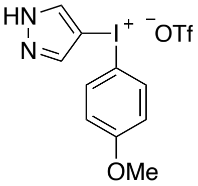	**V**	4.0	1) 60*ᵇ*<br>2) 0	1) 30 min<br>2) 15 min	65%*ᶜ*	[7]
**2sb-OTf**	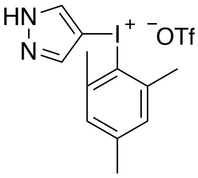	**V**	4.0	1) 60*ᵇ*<br>2) rt	1) 30 min<br>2) 30 min	54%*ᶜ*	[7]
**2te-OTf**	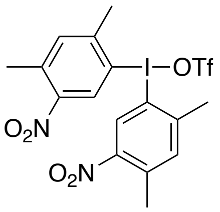	**II**	10	rt	19	27	[4b]

[a] CH_2Cl_2:TFE (1:1) was used as solvent mixture. [b] Reactions performed above the boiling point of the solvent were performed in pressure-stable microwave vials. [c] Overall yield of the reaction and filtration over Al_2O_3 plug to remove excess OTf anions.

2.3 SYNTHESIS OF NOVEL ARYL(ANISYL) IODONIUM TRIFLATES

In the synthesis of electron rich diaryliodonium triflates, the use of TfOH generally results in formation of dark-colored solids, without any visible impurities on ^{1}H, ^{13}C or ^{19}F NMR. While the use of these dark salts is straightforward in arylation of certain nucleophile classes, we have experienced that other arylations are low-yielding. The use of a weaker acid (TsOH) in the synthesis is hence preferred (see **Method III**), followed by *in situ* anion exchange with TfOH.[5] When this is unsuccessful, the anion exchange with NaOTf can be employed (**Method VI**).[8] Furthermore, we have previously reported a stepwise one-pot method employing TfOH, where addition of water quenches the excess of TfOH before the anisole is added, in the synthesis of pyridyl(aryl)iodonium salts (see **Method V**).[7]

In the arylation of amino acid derivatives, the purity of the salt was vital for high conversion. Therefore, all diaryliodonium triflates containing an anisyl dummy were obtained either via anion exchange from the corresponding tosylate (**Method III**), or synthesized according to a modified version of our step-wise one-pot procedure with water quench,[7] as described below.

Method VII: Synthesis of electron rich diaryliodonium salts with water quench.

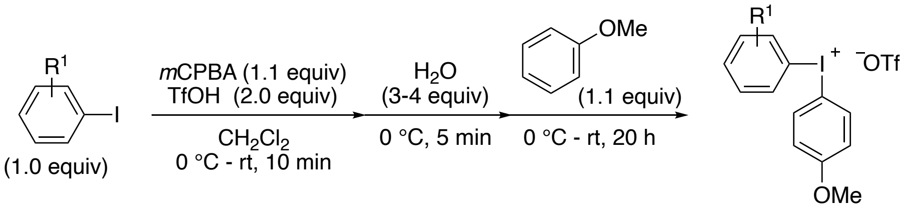

*m*CPBA (5.5 mmol, 1.1 equiv, 88% active oxidant) was dissolved in CH_2Cl_2 (20 mL) followed by the addition of iodoarene (5.0 mmol, 1.0 equiv). The solution was cooled to 0 °C by ice-bath followed by drop-wise addition of TfOH (10 mmol, 2.0 equiv). The mixture stirred at rt for 10 min and then again cooled down to 0 °C. H_2O (3-4 equiv, 15-20 mmol) was added and the solution was allowed to stir for 5 min in order to quench excess TfOH. To the cooled solution, anisole (5.5 mmol, 1.1 equiv) was added dropwise. Change of color occurred upon addition and solid formation was observed in most cases. The ice-bath was removed, and the reaction was stirred at rt overnight (20 h) and subsequently concentrated under vacuum. Et_2O (60 mL) was added and the reaction was stirred at rt for 10 minutes to precipitate the target diaryliodonium salt. The flask was then stored in the freezer for at least 1 h to ensure complete precipitation before the solid was filtered off, washed with Et_2O and dried under vacuum to give the diaryliodonium salt **2**.

4-Nitrophenyl(anisyl)iodonium triflate (2ac-OTf)

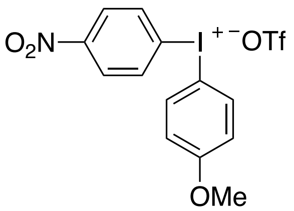

Synthesized according to **Method VII** with 1-iodo-4-nitrobenzene (5.0 mmol, 1.25 g) and *m*CPBA (5.5 mmol, 1.08 g, 88% active oxidant). Followed by the addition of TfOH (10.0 mmol, 0.89 mL), H_2O (4.0 equiv, 0.4 mL) and anisole (5.5 mmol, 0.60 mL). Reaction at rt for 23 h, concentrated under vacuum and excess Et_2O was added. The flask was stored in the freezer for 4 h and the precipitant was filtered off to give the diaryliodonium salt **2ac-OTf** as a fluffy white solid (2.13 g, 4.2 mmol, 84%).

mp: 153.2 °C; ^{1}H NMR (400 MHz, DMSO-d_6) δ 8.45 – 8.38 (m, 2H), 8.34 – 8.27 (m, 2H), 8.27 – 8.19 (m, 2H), 7.14 – 7.07 (m, 2H), 3.80 (s, 3H).^{13}C NMR (101 MHz, DMSO-d_6) δ 162.3, 149.3, 137.6, 136.0, 126.2, 123.1, 120.7 (q, *J* = 321.4 Hz, $CF_3SO_3^-$), 117.7, 105.6, 55.8. ^{19}F NMR (377 MHz, DMSO-d_6) δ -77.7; HRMS (ESI): calcd for $C_{14}H_{11}INO_3$ [M – OTf]$^+$: 355,9778; found: 355,9778.

4-Cyanophenyl(anisyl)iodonium triflate (2bc-OTf)

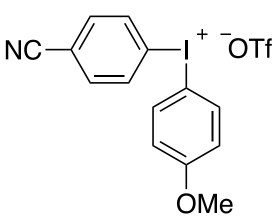

Synthesized according to **Method VII** with 4-iodobenzonitrile (5.0 mmol, 1.15 g) and *m*CPBA (5.5 mmol, 1.08 g, 88% active oxidant). Followed by the addition of TfOH (10.0 mmol, 0.89 mL), H_2O (3 equiv, 0.3 mL) and anisole (5.5 mmol, 0.60 mL). Reaction at rt for 21 h, concentrated under vacuum and excess Et_2O was added. The flask was stored in the freezer for 4 h and the precipitant was filtered off to give the diaryliodonium salt **2bc-OTf** as a pale yellow solid (2.19 g, 4.5 mmol, 90%).

mp: 157.9 °C; ^{1}H NMR (400 MHz, DMSO-d_6) δ 8.36 (d, *J* = 8.5 Hz, 2H), 8.21 (d, *J* = 9.0 Hz, 2H), 7.99 (d, *J* = 8.5 Hz, 2H), 7.10 (d, *J* = 9.0 Hz, 2H) 3.80 (s, 3H). ^{13}C NMR (101 MHz, DMSO-d_6 δ 162.2, 137.6, 135.4, 134.9, 121.8, 120.7 (q, *J* = 323.8 Hz, $CF_3SO_3^-$), 117.7, 117.5, 114.5, 105.5, 55.8. ^{19}F NMR (377 MHz, DMSO-d_6) δ -77.7; HRMS (ESI): calcd for $C_{14}H_{11}INO$ [M – OTf]$^+$: 335.9880; found: 335.9879.

4-Cyano-2-fluorophenyl(anisyl)iodonium triflate (2cc-OTf)

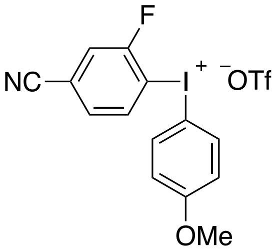

Synthesized according to **Method VII** with 2-fluoro-4-iodobenzonitrile (4.0 mmol, 1.00 g) and *m*CPBA (4.4 mmol, 0.82 g, 93% active oxidant). Followed by the addition of TfOH (8.0 mmol, 0.71 mL), H_2O (3.0 equiv, 0.2 mL) and anisole (4.4 mmol, 0.48 mL). Reaction at rt for 18 h, concentrated under vacuum and excess Et_2O was added. The flask was stored in the freezer for 18 h and the precipitant was filtered off to give the diaryliodonium salt **2cc-OTf** as a light pink solid (1.58 g, 3.1 mmol, 79%).

mp: 145.7 °C; ^{1}H NMR (400 MHz, DMSO-d_6) δ 8.56 (dd, J = 8.2, 6.0 Hz, 1H), 8.22 – 8.17 (m, 3H), 7.88 (dd, J = 8.2, 1.7 Hz, 1H), 7.08 (d, J = 9.1 Hz, 2H), 3.79 (s, 3H). ^{13}C NMR (101 MHz, DMSO-d_6) δ 162.3, 158.8 (d, $^1J_{F\text{-}C}$ = 250.8 Hz), 137.8, 137.5, 131.2 (d, $^3J_{F\text{-}C}$ = 3.7 Hz), 120.7 (d, $^2J_{F\text{-}C}$ = 27.1 Hz), 117.78, 117.1 (d, $^3J_{F\text{-}C}$ = 9.8 Hz), 116.5 (d, $^4J_{F\text{-}C}$ = 3.1 Hz), 110.1 (d, $^2J_{F\text{-}C}$ = 24.4 Hz), 105.8, 55.8. ^{19}F NMR (377 MHz, DMSO-d_6) δ -77.8, -95.5 (dd, J = 8.2, 6.1 Hz); HRMS (ESI): calcd for $[M – OTf]^+$: 353.9786; found: 353.9785.

4-(Trifluoromethyl)phenyl(anisyl)iodonium triflate (2dc-OTf)

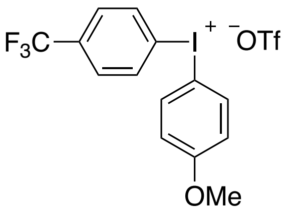

Synthesized according to **Method I** with 4-iodobenzotrifluoride (5.0 mmol, 0.75 mL) and *m*CPBA (5.5 mmol, 1.08 g, 85% active oxidant). Followed by the addition of TfOH (10.0 mmol, 0.89 mL), and anisole (5.0 mmol, 0.55 mL). *NB: No H_2O was added to the reaction.** Reaction at 0 °C for 1 h, concentrated under vacuum and excess Et_2O was added. The flask was stored in the freezer for 24 h and the precipitant was filtered off to give the diaryliodonium salt **2dc-OTf** as an off-white solid (2.40 g, 4.5 mmol, 89%), mp: 149.6 °C; ^{1}H NMR (400 MHz, DMSO-d_6) δ 8.39 (d, J = 8.3 Hz, 2H), 8.22 (d, J = 9.1 Hz, 2H), 7.91 (d, J = 8.3 Hz, 2H), 7.10 (d, J = 9.1 Hz, 2H), 3.80 (s, 3H).^{13}C NMR (101 MHz, DMSO-d_6) δ162.2, 137.5, 135.6, 131.7 (d, $^2J_{F\text{-}C}$ = 32.2 Hz), 128.3 (q, $^3J_{F\text{-}C}$ = 3.1 Hz), 123.4 (d, $^1J_{F\text{-}C}$ = 273.4 Hz), 121.2, 120.7 (q, J = 323.7 Hz, $CF_3SO_3^-$), 117.7 105.5, 55.8. ^{19}F NMR (377 MHz, DMSO-d_6) δ -61.6, -77.8; HRMS (ESI): calcd for $C_{14}H_{11}F_3IO$ $[M – OTf]^+$: 378.9801; found: 378.9805.

2dc-OTf* was synthesized before method VI was developed. Since the color of **2dc-OTf was off-white, it was judged acceptable to be used in this project too.*

2-Chloro-5-(trifluoromethyl)phenyl(anisyl)iodonium triflate (2fc-OTf)

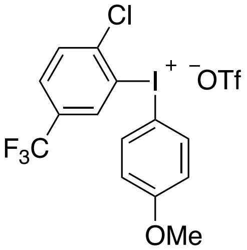

Synthesized according to **Method VII** with 4-chloro-3-iodobenzotrifluoride (4.0 mmol, 0.62 mL) and *m*CPBA (4.4 mmol, 0.82 g, 93% active oxidant). Followed by the addition of TfOH (8.0 mmol, 0.71 mL), H_2O (4.0 equiv, 0.40 mL) and anisole (4.4 mmol, 0.48 mL). Reaction at rt for 18 h, concentrated under vacuum and excess Et_2O was added. The flask was stored in the freezer for 24 h and the precipitant was filtered off to give the diaryliodonium salt **2fc-OTf** as a pale pink solid (1.38 g, 2.4 mmol, 61%).

mp: 176.6 °C; ^{1}H NMR (400 MHz, DMSO-d_6) δ 9.06 (s, 1H), 8.21 (d, J = 9.1 Hz, 2H), 8.11 – 7.98 (m, 2H), 7.11 (d, J = 9.1 Hz, 2H), 3.80 (s, 3H). ^{13}C NMR (101 MHz, DMSO-d_6) δ 162.3, 140.5 (q, $^3J_{F\text{-}C}$ = 1.4 Hz), 137.4, 135.2 (q, $^3J_{F\text{-}C}$ = 3.8 Hz), 131.2 , 131.2, 129.8 (q, $^2J_{F\text{-}C}$ = 33.6 Hz), 120.8, 120.7 (q, J = 323.9 Hz, $CF_3SO_3^-$), 184.64 (q, $^1J_{F\text{-}C}$ = 273.7 Hz), 117.8, 105.9, 55.8. ^{19}F NMR (377 MHz, DMSO-d_6) δ -61.0, -77.8; HRMS (ESI): calcd for $C_{14}H_{10}F_3ClIO$ $[M – OTf]^+$: 412.9411; found: 412.9410.

4-*tert*-Butylphenyl(anisyl)iodonium triflate (2jc-OTf)

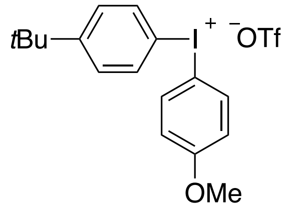

Synthesized according to **Method VII** with 4-tertButyliodobenzene (5.0 mmol, 0.87 mL) and *m*CPBA (5.5 mmol, 1.08g, 88% active oxidant). Followed by the addition of TfOH (10.0 mmol, 0.89 mL), H_2O (3.0 equiv, 0.3 mL) and anisole (5.5 mmol, 0.6 mL). Reaction at rt for 17 h, concentrated under vacuum and excess Et_2O was added. The flask was stored in the freezer for 24 h and the precipitant was filtered off to give the diaryliodonium salt **2jc-OTf** as an off-white solid (1.79 g, 3.5 mmol, 69%).

mp: 181.8 °C; ^{1}H NMR (400 MHz, DMSO-d_6) δ 8.17 (d, *J* = 9.0 Hz, 2H), 8.09 (d, *J* = 8.7 Hz, 2H), 7.53 (d, *J* = 8.7 Hz, 2H), 7.07 (d, *J* = 9.0 Hz, 2H), 3.79 (s, 3H), 1.25 (s, 9H). ^{13}C NMR (101 MHz, DMSO-d_6) δ 162.0, 155.0, 137.2, 134.6, 128.8, 120.7 (q, *J* = 322.1 Hz, $CF_3SO_3^-$), 117.5, 113.5, 105.3, 55.7, 34.9, 30.7. ^{19}F NMR (377 MHz, DMSO-d_6) δ -77.7; HRMS (ESI): calcd for $C_{17}H_{20}IO$ [M – OTf]$^+$: 367.0553; found: 367.0554.

2.4 Other Methods used for Synthesis of Diaryliodonium Salts

Table S3. Diaryliodonium salts synthesized by other methods.

**2aa-OTs**	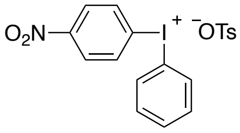	4-Nitrophenyl(phenyl)iodonium tosylate (**2aa-OTs**) was prepared in 86% in a stepwise fashion, via the isolated Koser reagent derivative, following literature reports.[17]
**2aa-Br**	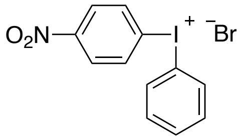	4-Nitrophenyl(phenyl)iodonium bromide (**2aa-Br**)[18] was prepared in 85% via an anion exchange from **2aa-OTf** according to literature reports.[19]
**2ab-OTf**	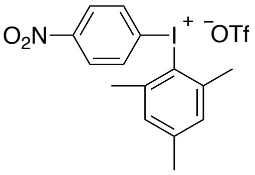	4-Nitrophenyl(mesityl)iodonium triflate (**2ab-OTf**)[11b] was prepared in 48% in a stepwise fashion, via the isolated Koser's reagent derivative, following literature reports,[17] then conducting anion exchange from OTs to OTf following **Method VI**.
**2ad-OTs**	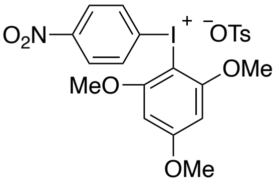	4-Nitrophenyl(2,4,6-trimethoxyphenyl)iodonium tosylate (**2ad-OTs**) was prepared in 90% according to literature reports.[19]
**2ub-OTf**	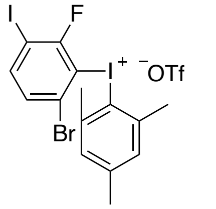	(6-Bromo-2-fluoro-3-iodophenyl)mesityl triflate (**2ub-OTf**) was prepared in 78% in a stepwise fashion, via the isolated Koser's reagent derivative, following literature reports,[20] then conducting anion exchange from OTs to OTf following **Method VI**.

3 Synthesis of Amino Acid Esters

3.1 Substrates Used in the Study

Figure S3. Synthesized amino acid esters.

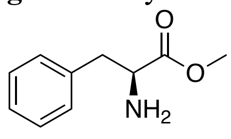

1a

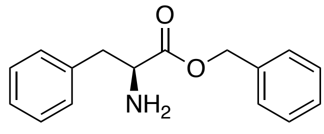

1b

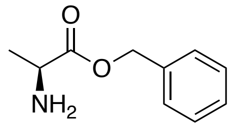

1c

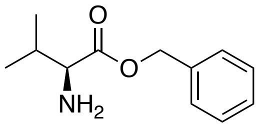

1d

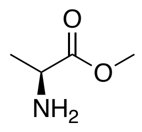

1e

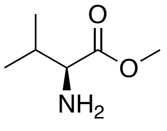

1f

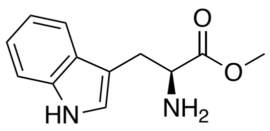

1g

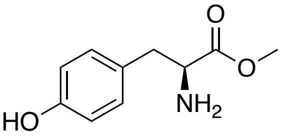

1h

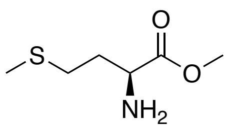

1i

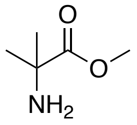

1j

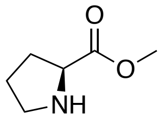

5a

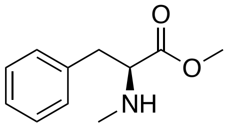

5b

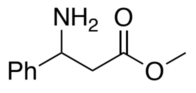

5c

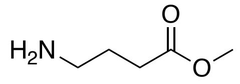

5d

3.2 Synthesis of Amino Acid Methyl Ester HCl Salts

Amino acid hydrochlorides **1a-HCl**, **1e-HCl**, **1f-HCl**, **1g-HCl** and **1i-HCl** were purchased from commercial sources. **1h-HCl, 1j-HCl**, **5c-HCl** and **5d-HCl** were synthesized as described below, according to a modified literature procedure.[21]

General procedure 1 (GP1):

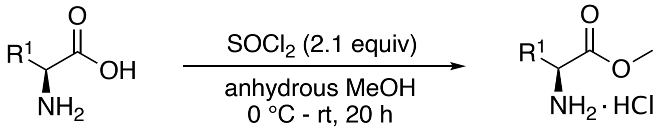

In an oven dried round bottomed flask, amino acid *or* amino acid derivative (10.0 mmol, 1.0 equiv) was dissolved in anhydrous MeOH (13 mL) and then cooled to 0 °C. Thionyl chloride (21 mmol, 2.1 equiv) was added slowly and the solution turned yellow transparent. The reaction was allowed to reach rt and stirred for 20 h followed by removal of solvent. The product was obtained as a solid that was used without further purification.

***L*-Tyrosine methyl ester hydrochloride (1h-HCl)**

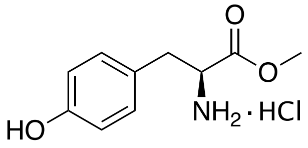

Synthesized according to **GP1** with L-Tyrosine (5.0 mmol, 1.0 equiv, 0.91 g) and $SOCl_2$ (7.5 mmol, 1.5 equiv, 0.5 mL) dissolved in MeOH (7 mL). The reaction stirred for 20 h at rt followed by removal of solvent. The product **1h-HCl** was obtained as a white solid (1.12 g, 4.8 mmol, 96%).
^{1}H NMR (400 MHz, DMSO-d$_6$) δ 9.45 (s, 1H), 8.57 (br. s, 2H), 7.00 (d, *J* = 8.4 Hz, 2H), 6.72 (d, *J* = 8.4 Hz, 2H), 4.16 (t, *J* = 6.4 Hz, 1H), 3.67 (s, 3H), 3.06 (dd, *J* = 14.2, 5.9 Hz, 1H), 2.98 (dd, *J* = 14.2, 7.0 Hz, 1H). ^{13}C NMR (101 MHz, DMSO-d$_6$) δ 169.5, 156.7, 130.4, 124.3, 115.4, 53.4, 52.6, 35.1; The analytical data are consistent with previous reports.[22]

Methyl α-aminoisobutyrate hydrochloride (1j-HCl)

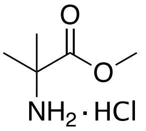

Synthesized according to **GP1** with 2-aminoisobutyric acid (10 mmol, 1.0 equiv, 1.06 g) and $SOCl_2$ (21 mmol, 2.1 equiv, 1.5 mL) dissolved in MeOH (13 mL).The reaction stirred for 20 h at rt followed by removal of solvent. The product **1j-HCl** was obtained as a white solid (1.5 g, 9.8 mmol, 98%).

^{1}H NMR (400 MHz, DMSO-d_6) δ ^{1}H NMR (400 MHz, DMSO) δ 8.77 (br. s, 3H), 3.74 (s, 3H), 1.48 (s, 6H). ^{13}C NMR (101 MHz, DMSO-d_6) δ 172.0, 55.8, 53.1, 23.4. The analytical data are consistent with previous reports.[23]

β-Phenylalanine methyl ester hydrochloride (5c-HCl)

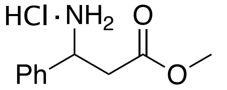

Synthesized according to **GP1** with β-Phenylalanine (1.9 mmol, 1.0 equiv, 0.31 g) and $SOCl_2$ (3.7 mmol, 2.1 equiv, 0.3 mL) dissolved in MeOH (3 mL). The reaction stirred for 20 h at rt followed by removal of solvent. The product **5c-HCl** was obtained as a white solid (0.40 g, 1.9 mmol, quant).

^{1}H NMR (400 MHz, DMSO-d_6) δ 8.60 (br. s, 3H), 7.56 – 7.49 (m, 2H), 7.47 – 7.34 (m, 3H), 4.60 (app. dd, J = 8.3, 6.1 Hz, 1H), 3.56 (s, 3H), 3.16 (dd, J = 16.2, 6.1 Hz, 1H), 2.99 (dd, J = 16.2, 8.3 Hz, 1H). ^{13}C NMR (101 MHz, DMSO-d_6) δ 169.6, 136.8, 128.9, 128.7, 127.5, 51.8, 50.9, 38.5; The analytical data are consistent with previous reports.[24]

Methyl γ-aminobutyrate hydrochloride (5d-HCl)

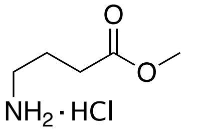

Synthesized according to **GP1** with 4-aminobutyric acid (10 mmol, 1.0 equiv, 1.03 g) and $SOCl_2$ (21 mmol, 2.1 equiv, 1.5 mL) dissolved in MeOH (13 mL).The reaction stirred for 22 h at rt followed by removal of solvent. The product **5d-HCl** was obtained as a white solid (1.5 g, 9.9 mmol, 99%).

^{1}H NMR (400 MHz, DMSO-d_6) δ ^{1}H NMR (400 MHz, DMSO) 8.06 (br. s, 3H), 3.60 (s, 3H), 2.87 – 2.68 (m, 2H), 2.44 (t, J = 7.4 Hz, 2H), 1.81 (p, J = 7.4 Hz, 2H). ^{13}C NMR (101 MHz, DMSO-d_6) δ 172.7, 51.4, 38.0, 30.1, 22.4. The analytical data are consistent with previous reports.[25]

3.3 Synthesis of Amino Acid Benzyl Ester TFA Salts

The synthesis of **1b-TFA**, **1c-TFA** and **1d-TFA** was done through modification of a reported procedure.[26]

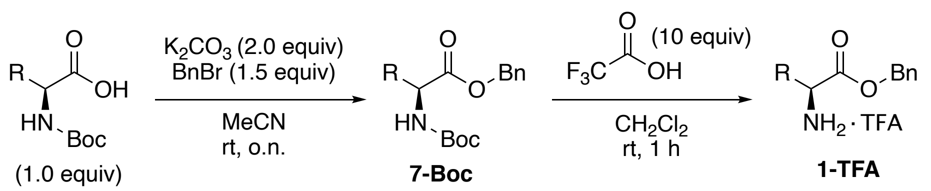

General procedure 2 (GP2):

In an oven dried round bottomed flask, N-Boc protected amino acid (5.0 mmol, 1.0 equiv) and K_2CO_3 (10.0 mmol, 2.0 equiv) were dissolved in anhydrous MeCN (17 mL, partially soluble). The solution was cooled to 0 °C followed by dropwise addition of BnBr (7.5 mmol, 1.5 equiv). The reaction was stirred at rt for 19-24 h. EtOAc (20 mL) was added and the mixture was washed with H_2O (3×20 mL). The organic phase was collected, the solvent was removed under reduce pressure and the crude was dried over Celite then purified by column chromatography (n-pentane/EtOAc) to obtain product **7-Boc**.

General procedure 3 (GP3):

N-Boc protected amino benzyl ester **7-Boc** (3.0 mmol) was dissolved in CH_2Cl_2 (6 mL) and cooled to 0 °C. To the cold solution, trifluoroacetic acid (30.0 mmol, 30 equiv) was added dropwise over a period of 10 min. The reaction mixture was allowed to stir for 30 min at rt. The solvent and excess

TFA was partially removed under reduced pressure, until 2 mL remained. The residue was co-evaporated with toluene under reduced pressure in order to ensure complete removal of TFA (bp: 72.4 °C) and to prevent too high concentration of the acid, which could cause racemization of the product. The product **1-TFA** was obtained as a salt without further purification.

***N*-Boc-*L*-phenylalanine benzyl ester (7b-Boc)**

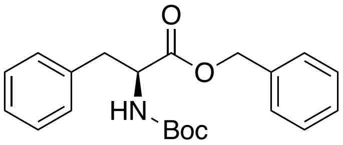

Synthesized according to **GP2** with Boc-L-Phe-OH (5.0 mmol, 1.33 g), K_2CO_3 (10.0 mmol, 1.38 g) and BnBr (5.5 mmol, 0.89 mL) Reaction time 24 h, purified by column chromatography (*n*-pentane/EtOAc 1:0 to 9:1) to yield **7b-Boc** as a colorless solid (1.78 g, 5.0 mmol, quant).
R_f = 0.68 (*n*-pentane/EtOAc, 9:1). ^{1}H NMR (400 MHz, $CDCl_3$) δ 7.39 – 7.33 (m, 3H), 7.31 – 7.28 (m, 2H), 7.24 – 7.22 (m, 3H), 7.06 – 7.02 (m, 2H), 5.17 (d, *J* = 12.3 Hz, 1H), 5.11 (d, *J* = 12.3 Hz, 1H), 4.98 (d, *J* = 8.4 Hz, 1H), 4.63 (q, *J* = 6.6 Hz, 1H), 3.14 – 3.04 (m, 2H), 1.41 (s, 9H). ^{13}C NMR (101 MHz, $CDCl_3$) δ 171.9, 155.2, 136.0, 135.3, 129.5, 128.7, 128.7, 128.6, 127.1, 80.1, 67.2, 54.6, 38.4, 28.4. The analytical data are consistent with previous reports. [26-27]

***L*-phenylalanine benzyl ester trifluoroacetic acid salt (1b-TFA)**

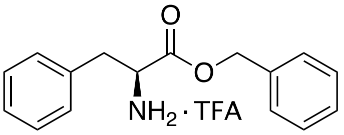

Synthesized according to **GP3** with **7b-Boc** (3.32 mmol, 1.18 g) and TFA (33.2 mmol, 2.5 mL). The reaction was stirred for 1 h, product **1b-TFA** was obtained as a white solid (1.12 g, 3.0 mmol, 91%).
^{1}H NMR (400 MHz, $CDCl_3$) δ 7.76 (br s, 2H), 7.33 – 7.30 (m, 3H), 7.24 – 7.14 (m, 5H), 7.12 – 7.04 (m, 2H), 5.09 (d, *J* = 12.0 Hz, 1H), 5.02 (d, *J* = 12.0 Hz, 1H), 4.23 (t, *J* = 6.5 Hz, 1H), 3.31 – 3.13 (m, 2H). ^{13}C NMR (101 MHz, $CDCl_3$) δ 169.1, 162.4 (q, *J* = 35.2 Hz), 134.3, 133.4, 129.5, 129.2, 128.9, 128.9, 128.8, 128.0, 116.6 (q, *J* = 291.3 Hz), 68.4, 54.2, 36.4. ^{19}F NMR (377 MHz, $CDCl_3$) δ -75.6; HRMS (ESI): calcd for $C_{16}H_{17}NO_2$ [M – TFA]$^+$: 255.3110; found: 255.31109.

***N*-Boc-*L*-alanine benzyl ester (7c-Boc)**

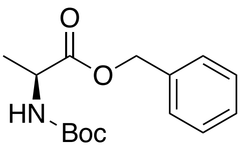

Synthesized according to **GP2** with Boc-L-Ala-OH (5.0 mmol, 0.95 g), K_2CO_3 (10.0 mmol, 1.38 g) and BnBr (5.5 mmol, 0.89 mL). Reaction time 19 h, purified by column chromatography (*n*-pentane/EtOAc 10:1 to 5:1) to obtain **7c-Boc** as a colorless oil (1.41 g, 4.9 mmol, 97%).
R_f= 0.22 (*n*-pentane/EtOAc, 10:1). ^{1}H NMR (400 MHz, $CDCl_3$) δ 7.42 – 7.26 (m, 5H), 5.25 – 5.10 (m, 2H), 5.04 (br s, 1H), 4.41 – 4.33 (m 1H), 1.43 (s, 9H), 1.39 (d, *J* = 7.2 Hz, 3H). The analytical data are consistent with previous reports.[27]

***L*-Alanine benzyl ester trifluoroacetic acid salt (1c-TFA)**

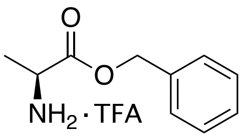

Synthesized according to **GP3** with **7c-Boc** (4.85 mmol, 1.36 g) and TFA (48.5 mmol, 3.7 mL). The reaction was stirred for 10 min, product **1c-TFA** was obtained as a white viscous oil (1.40 g, 4.8 mmol, quant).
^{1}H NMR (400 MHz, $CDCl_3$) δ 8.23 (s, 3H), 7.36 – 7.32 (m, 3H), 7.31 – 7.28 (m, 2H), 5.24 – 5.11 (m, 2H), 4.15 – 4.04 (m, 1H), 1.58 (dd, *J* = 7.3, 3.3 Hz, 3H). ^{13}C NMR (101 MHz, $CDCl_3$) δ 170.2, 162.5 (q, *J* = 36.6 Hz), 134.4, 129.0, 128.9, 128.5, 116.0 (q, *J* = 290.8 Hz), 68.6, 49.3, 15.9. ^{19}F NMR (377 MHz, $CDCl_3$) δ -76.0; HRMS (ESI): calcd for $C_9H_{13}NO_2$ [M – TFA]$^+$: 167.2045; found: 167.2040.

N-Boc-L-valine benzyl ester (7d-Boc)

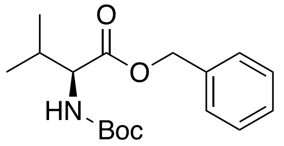

Synthesized according to **GP2** with Boc-L-Val-OH (5.0 mmol, 1.07 g), K_2CO_3 (10.0 mmol, 1.38 g) and BnBr (5.5 mmol, 0.89 mL) Reaction time 20 h. Purification by column chromatography (*n*-pentane/EtOAc 1:0 to 19:1) yielded **7d-Boc** as a colorless oil (1.46 g, 4.8 mmol, 95%).

R_f = 0.30 (*n*-pentane/EtOAc, 10:1). ^{1}H NMR (400 MHz, $CDCl_3$) δ 7.39 – 7.32 (m, 5H), 5.20 (d, J = 12.4 Hz, 1H), 5.13 (d, J = 12.4 Hz, 1H), 5.02 (d, J = 9.2 Hz, 1H), 4.27 (dd, J = 9.2, 4.7 Hz, 1H), 2.18 – 2.12 (m, 1H), 1.44 (s, 9H), 0.94 (d, J = 6.8 Hz, 3H), 0.85 (d, J = 6.8 Hz, 3H). ^{13}C NMR (101 MHz, $CDCl_3$) δ 172.5, 155.8, 135.6, 128.7, 128.5, 128.5, 79.9, 67.0, 58.7, 31.5, 28.5, 19.2, 17.6. The analytical data are consistent with previous reports.[27a], [28]

L-alanine benzyl ester trifluoroacetic acid salt (1d-TFA)

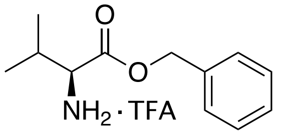

Synthesized according to **GP3** with **7d-Boc** (4.8 mmol, 1.46 g) and TFA (47.6 mmol, 3.6 mL). Reaction time 1 h, product **1d-TFA** was obtained as a white viscous oil (1.52 g, 4.8 mmol, quant).

^{1}H NMR (400 MHz, $CDCl_3$) δ 7.99 (br s, 2H), 7.41 – 7.23 (m, 4H), 7.21 – 7.10 (m, 1H), 5.28 (d, J = 11.9 Hz, 1H), 5.16 (d, J = 11.9 Hz, 1H), 4.00 (d, J = 4.0 Hz, 1H), 2.41 – 2.29 (m, 1H), 1.00 (dd, J = 7.0, 5.3 Hz, 6H). ^{13}C NMR (101 MHz, $CDCl_3$) δ 169.1, 161.7 (q, J = 36.4 Hz), 134.2, 129.2, 128.9, 128.9, 128.4, 125.5, 68.9, 58.8, 29.9, 17.6, 17.5. ^{19}F NMR (377 MHz, $CDCl_3$) δ -76.0; The analytical data are consistent with previous reports.[29]

3.4 Synthesis of Free Amino Acid Esters

Several methods were employed to remove the HCl or TFA from **1-HCl** or **1-TFA** to obtain the free amino acid esters **1**, as detailed below.

NB: Due to the known instability of the amino acid esters **1**,[30] they were stored under argon in the freezer (-18 °C) and could be used up to 1.5 weeks after deprotonation. Material which had started to solidify was discarded.

3.4.1 Treatment with K_2CO_3 or Na_2CO_3

Performed according to an established procedure.[21]

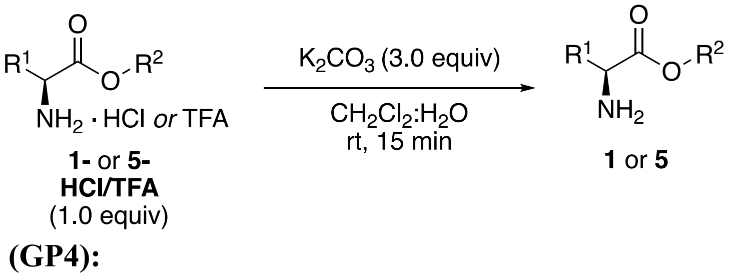

General Procedure 4 (GP4):

L-Amino methyl ester hydrochloride or trifluoroacetic acid salt **1-** or **5-HCl/1-TFA** (2 mmol) was suspended in CH_2Cl_2 (6 mL). The solution was cooled to 0 °C followed by addition of K_2CO_3 (30% aq, 3.25 equiv) dissolved in H_2O (6 mL). *Na_2CO_3 proved to also be an efficient base*. The mixture was left to stir vigorously at rt for 20-30 min. After the solid had completely dissolved, additional water (6 mL) was added, and the mixture was extracted with CH_2Cl_2 (2×10 mL). The organic phase was collected, and the solvent was removed under reduced pressure. The crude was filtrated through a Celite plug with 1:1 *n*-pentane/EtOAc followed by the removal of solvent under reduced pressure. Product **1** was obtained as a pale yellow oil that was used without further purification.

***L*-Phenylalanine methyl ester (1a)**

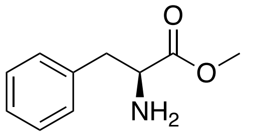

Synthesized according to **GP4** with **1a-HCl** (2.0 mmol, 0.43 g), product **1a** was obtained as a pale yellow oil (0.35 g, 1.9 mmol, 97%).
^{1}H NMR (400 MHz, $CDCl_3$) δ 7.31 – 7.27 (m, 2H), 7.25 – 7.15 (m, 3H), 3.75 – 3.71 (m, 1H), 3.70 (s, 3H), 3.08 (dd, *J* = 13.5, 5.2 Hz, 1H), 2.85 (dd, *J* = 13.5, 7.9 Hz, 1H), 1.42 (br s, 2H). ^{13}C NMR (101 MHz, $CDCl_3$) δ 175.6, 137.4, 129.4, 128.7, 127.0, 56.0, 52.1, 41.3; The analytical data are consistent with previous reports.[31]

***L*-Phenylalanine benzyl ester (1b)**

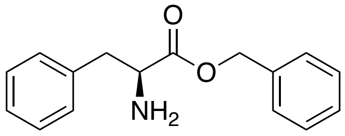

Synthesized according to **GP4** with **1b-TFA** (1.3 mmol, 0.49 g), product **1b** was obtained as a pale yellow oil (0.31 g, 1.2 mmol, 92%).
^{1}H NMR (400 MHz, $CDCl_3$) δ 7.39 – 7.32 (m, 3H), 7.32 – 7.20 (m, 5H), 7.17 – 7.14 (m, 2H), 5.14 (s, 2H), 3.78 (dd, *J* = 7.6, 5.5 Hz, 1H), 3.09 (dd, *J* = 13.5, 5.5 Hz, 1H), 2.90 (dd, *J* = 13.5, 7.6 Hz, 1H), 1.50 (br. s, 2H). ^{13}C NMR (101 MHz, $CDCl_3$) δ 175.1, 137.2, 135.7, 129.5, 128.7, 128.7, 128.6, 128.5, 127.0, 66.9, 56.0, 41.2; The analytical data are consistent with previous reports.[32]

***L*-Alanine benzyl ester (1c)**

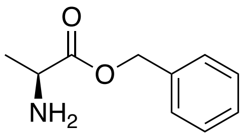

Synthesized according to **GP4** with **1c-TFA** (2.1 mmol, 0.63 g), product **1c** was obtained as a pale yellow oil (0.34 g, 1.9 mmol, 89%).
^{1}H NMR (400 MHz, $CDCl_3$) δ 7.47 – 7.28 (m, 5H), 5.13 (s, 2H), 3.57 (q, *J* = 7.1 Hz, 1H), 1.75 (br s, 2H), 1.33 (d, *J* = 7.1 Hz, 3H). ^{13}C NMR (101 MHz, $CDCl_3$) δ 176.4, 135.8, 128.6, 128.3, 128.2, 66.6, 50.1, 20.6; HRMS (ESI): calcd for $C_{10}H_{14}NO_2$ ([M + Na^+]): 180.1019; found: 180.1021.

***L*-Valine benzyl ester (1d)**

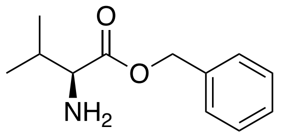

Synthesized according to **GP4** with **1d-TFA** (1.3 mmol, 0.42 g), product **1d** was obtained as a pale yellow oil (0.21 g, 1.0 mmol, 79%).
^{1}H NMR (400 MHz, $CDCl_3$) δ 7.40 – 7.29 (m, 5H), 5.16 (dd, *J* = 12.9, 2.7 Hz, 2H), 3.34 (d, *J* = 4.9 Hz, 1H), 2.05 (pd, *J* = 6.8, 4.9 Hz, 1H), 0.96 (d, *J* = 6.8 Hz, 3H), 0.88 (d, *J* = 6.8 Hz, 3H). ^{13}C NMR (101 MHz, $CDCl_3$) δ 175.6, 135.9, 128.7, 128.5, 128.5, 66.7, 60.1, 32.3, 19.5, 17.2; The analytical data are consistent with previous reports.[28b]

Methyl α-aminoisobutyrate (1j)

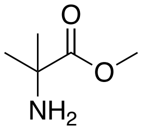

Synthesized according to **GP4** with **1i-HCl** (1.0 mmol, 0.17 g), however, product **1j** proved to be too volatile and could not be isolated by following **GP4**.

β-Phenylalanine methyl ester (5c)

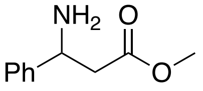

Synthesized according to **GP4** with **5c-HCl** (0.74 mmol, 0.16 g), product **5c** was obtained as a pale yellow oil (0.13 g, 0.72 mmol, 96%).
^{1}H NMR (400 MHz, $CDCl_3$) δ 7.44 – 7.21 (m, 5H), 4.43 (t, *J* = 6.8 Hz, 1H), 3.69 (s, 3H), 2.68 (d, *J* = 6.8 Hz, 2H), 1.76 – 1.71 (m, 2H). ^{13}C NMR (101 MHz, $CDCl_3$) δ 172.6, 144.6, 128.8, 127.6, 126.3, 52.8, 51.9, 44.0; The analytical data are consistent with previous reports.[33]

Methyl γ-aminobutyrate (5d)

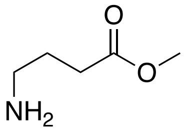

Synthesized according to **GP4** with **5d-HCl** (1.0 mmol, 0.16 g), product **5d** was obtained as a pale yellow oil (35 mg, 0.30 mmol, 30%).
^{1}H NMR (400 MHz, $CDCl_3$) δ 5.57 (br. s, 2H), 3.46 – 3.34 (m, 2H), 2.31 (td, *J* = 7.9, 7.4, 1.0 Hz, 2H), 2.21 – 2.08 (m, 2H), 1.59 (s, 3H). The analytical data are consistent with previous reports.[34]

3.4.2 Treatment with $KHCO_3$ or $NaHCO_3$

L-Tyrosine methyl ester (**1h**) was prepared according to established procedure.[35]

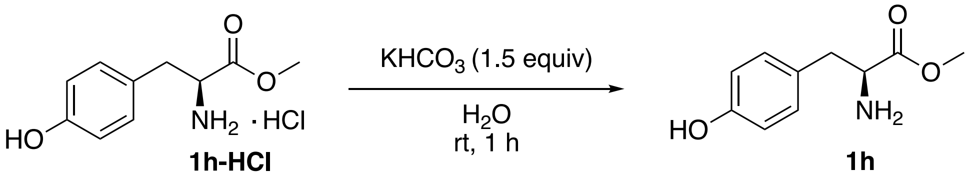

1h-HCl (1.0 equiv) **1h**

General Procedure 5 (GP5):
L-Tyrosine methyl ester hydrochloride (**1h-HCl**, 1.7 mmol, 0.40 g) was suspended in H_2O (5 mL). The solution was cooled to 0 °C followed by addition of $KHCO_3$ (2.6 mmol, 1.5 equiv, 0.22 g). *Na_2CO_3 proved to also be an efficient base*. The mixture stirred at rt for 1 h followed by washing with chloroform (3×15 mL). The organic phase was collected, the solvent was removed under reduced pressure and the crude was filtered through a Celite plug with chloroform. The solvent was removed again under reduced pressure. Product **1h** was obtained as a white solid that was used without further purification. (0.23 g, 1.2 mmol, 68%). ^{1}H NMR (400 MHz, $CDCl_3$) δ 7.03 (d, *J* = 8.5 Hz, 2H), 6.72 (d, *J* = 8.5 Hz, 2H), 3.73 (s, 3H), 3.72 – 3.68 (m, 1H), 3.03 (dd, *J* = 13.7, 5.1 Hz, 1H), 2.81 (dd, *J* = 13.7, 7.7 Hz, 1H). ^{13}C NMR (101 MHz, $CDCl_3$) δ 175.6, 154.9, 130.5, 128.9, 115.7, 55.9, 52.2, 40.2; The analytical data are consistent with previous reports.[36]

3.4.3 Treatment with Et_3N

Amino acid derivatives that proved to be volatile were treated with Et_3N according to an established procedure.[37]

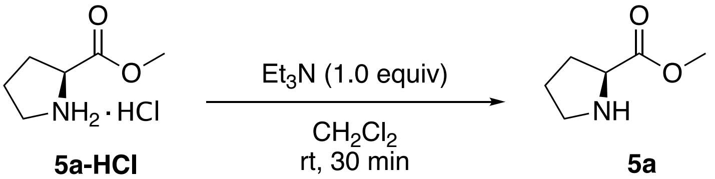

5a-HCl (1.0 equiv) **5a**

General Procedure 6 (GP6):
L-Proline methyl ester hydrochloride **5a-HCl** (2.0 mmol, 0.33 g) was dissolved in CH_2Cl_2 (4 mL). The solution was cooled to 0 °C and triethylamine (2.0 mmol, 1.0 equiv, 0.28 mL) was added dropwise. The mixture was left to stir vigorously at rt for 30 min. After the solid had completely dissolved the crude was concentrated under reduced pressure under cooling. **NB**: *not until dryness, as deprotonated L-proline methyl ester **5a** is easily lost under high vacuum presumably due to its volatility.* The crude was dissolved in Et_2O and filtrated through a celite plug. The solvent was again removed by reduced pressure under cooling, but not to complete dryness. Residual solvent was removed with a gentle stream of air over the liquid oil. The wanted product **5a** was obtained as a pale yellow oil (0.20 g, 1.6 mmol, 79%). ^{1}H NMR (400 MHz, $CDCl_3$) δ 3.74 (dd, *J* = 8.7, 5.7 Hz, 1H), 3.70 (s, 3H), 3.05 (dt, *J* = 10.1, 6.7 Hz, 1H), 2.89 (dt, *J* = 10.1, 6.7 Hz, 1H), 2.22 (br s, 1H), 2.15 – 2.06 (m, 1H), 1.89 – 1.66 (m, 3H). The analytical data are consistent with previous reports.[31b, 37]

Other Methods used for Deprotonation

Table S4. Deprotonation of amino acid derivatives by other methods.

**1e**	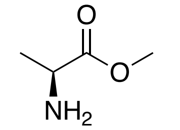	Deprotonation of L-Ala-OMe HCl followed established procedure[38] to obtain L-Ala-OMe **1e** in 58% yield. *(Solvent removed under vacuum at 0 °C)*
**1f**	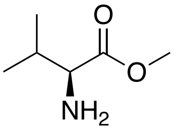	Deprotonation of L-Val-OMe HCl followed established procedure[38] to obtain L-Val-OMe **1f** in 65% yield. *(Solvent removed under vacuum at 0 °C)*
**1g**	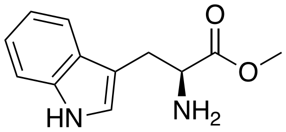	Deprotonation of L-Trp-OMe HCl followed established procedure[39] to obtain L-Trp-OMe **1g** in 52% yield.
**1i**	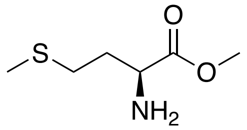	Deprotonation of L-Met-OMe HCl followed established procedure[40] to obtain L-Met-Ome **1i** in 64% yield.

3.5 N-Functionalization of Amino Acid Esters

N-Methyl-*L*-phenylalanine methyl ester (5b)

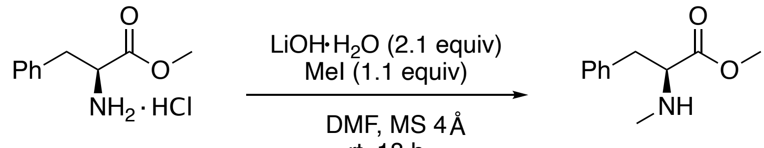

1a-HCl (1.0 equiv) **5b**

Synthesized according to a modified reported procedure:[41] LiOH·H_2O (2.15 mmol, 2.15 equiv, 90 mg) and 4 Å molecular sieves were mixed under argon atmosphere in anhydrous DMF (3 mL) for 20 min. L-Phenylalanine methyl ester hydrochloride (1.0 mmol, 0.22 g) was added and the solution was stirred for a further 40 min. The mixture was cooled to 0 °C before the addition of MeI (1.12 mmol, 1.12 equiv, 70 µL) and the reaction was left to stir at rt for 18 h. The brown suspension was filtered, and the filter cake was washed with EtOAc (40 mL). The filtrate was collected and washed with H_2O (3×20 mL) followed by removal of solvent under reduced pressure. The crude was purified by flash column chromatography (*n*-pentane/EtOAc, 1:0 to 5:1) to give **5b** as a pale-yellow oil (37 mg, 0.19 mmol, 19 %), with the corresponding dimethylated byproduct isolated in 14% yield.

R_f = 0.35 (*n*-pentane/EtOAc, 2:1). ^{1}H NMR (400 MHz, $CDCl_3$) δ 7.38 – 7.15 (m, 5H), 3.70 (s, 3H), 3.49 (t, *J* = 6.8 Hz, 1H), 2.99 (dd, *J* = 6.8, 2.1 Hz, 2H), 2.40 (s, 3H), 1.63 (br s, 1H). ^{13}C NMR (101 MHz, $CDCl_3$) δ 174.9, 137.3, 129.2, 128.5, 126.8, 64.7, 51.7, 39.5, 34.8; The analytical data are consistent with previous reports.[41]

N-Acetyl-*L*-phenylalanine methyl ester (7a-Ac)

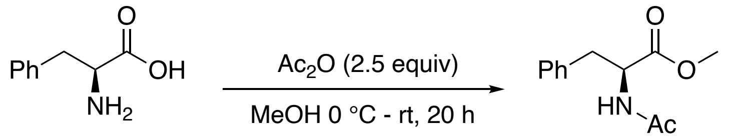

Phe (1.0 equiv) **7a-Ac**

Synthesized according to a modified reported procedure:[42] **Phe** (5.0 mmol, 1.0 equiv, 0.83 g) was dissolved in in anhydrous MeOH (2.2 mL) and acetic anhydride (12.9 mmol, 2.6 equiv, 1.2 mL). The mixture was stirred under reflux for 6 h then after allowing the mixture to cool to rt, solvent was removed under reduced pressure obtaining the crude as a colorless oil. The crude was dried over celite and purified by column chromatography (*n*-pentane/EtOAc 1:1) and product *N*-Ac-Phe-OMe **7a-Ac** was obtained as a white solid (0.32 g, 1.5 mmol, 29%).

Rf= 0.46 (*n*-pentane:EtOAc, 1:1). ^{1}H NMR (400 MHz, $CDCl_3$) δ 7.34 – 7.21 (m, 4H), 7.12 – 7.02 (m, 2H), 5.88 (d, *J* = 7.8 Hz, 1H), 4.89 (dt, *J* = 7.8, 5.7 Hz, 1H), 3.73 (s, 3H), 3.16 (dd, *J* = 13.9, 5.7 Hz, 1H), 3.10 (dd, *J* = 13.9, 5.7 Hz, 1H), 1.99 (s, 3H). ^{13}C NMR (101 MHz, $CDCl_3$) δ 172.2, 169.7, 136.0, 129.4, 128.8, 127.3, 53.2, 52.5, 38.0, 23.3. The analytical data are consistent with previous reports.[42]

N-Tosyl-*L*-phenylalanine methyl ester (7a-Ts)

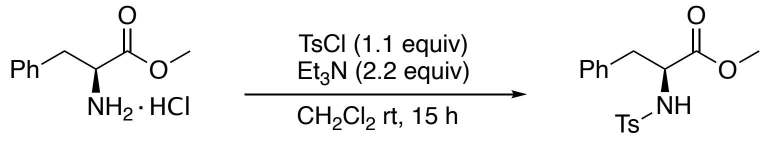

1a-HCl (1.0 equiv) **7a-Ts**

Synthesized according to a modified reported procedure:[43] To a dry round bottomed flask, **1a-HCl** (1.0 mmol, 1.0 equiv, 0.22 g) was dissolved in anhydrous CH_2Cl_2 (1.5 mL). The mixture was cooled to 0 °C followed by addition of *p*-toluenesulfonyl chloride (1.1 mmol, 1.1 equiv, 0.21 g). The ice bath was removed, and the reaction was stirred at rt for 15 h. Then solvent was removed under reduced pressure, EtOAc (8 mL) was added followed by washing with H_2O (3×8 mL) followed by drying over Na_2CO_4. The crude mixture was dried over Celite and purified by column chromatography (*n*-pentane/EtOAc, 10:1 to 1:1) to deliver product **7a-Ts** as a white solid (0.29 g, 0.86 mmol, 86%).

R_f= 0.23 (*n*-pentane/EtOAc, 5:1). ^{1}H NMR (400 MHz, $CDCl_3$) δ 7.66 – 7.60 (m, 2H), 7.26 – 7.20 (m, 5H), 7.09 – 7.04 (m, 2H), 5.05 (d, *J* = 9.2 Hz, 1H), 4.20 (dt, *J* = 9.2, 6.0 Hz, 1H), 3.49 (s, 3H), 3.03 (d, *J* = 6.6 Hz, 2H). ^{13}C NMR (101 MHz, $CDCl_3$) δ 171.4, 143.7, 136.7, 135.0, 129.7, 129.5, 128.7, 127.4, 127.3, 56.7, 52.5, 39.5, 21.7. The analytical data are consistent with previous reports.[43]

4 Arylation of Amino Acid Esters

4.1 Optimization Studies

4.1.1 Screening of Reaction Conditions

Table S5 depicts the initial screening of the reaction between **1a** and **2aa-OTf** with various bases and solvents. It became apparent that a high-boiling, non-polar solvent was required for the transformation. The high temperatures had a negative effect on the chemoselectivity and mass balance of the reaction.

Table S5. Solvent screening.

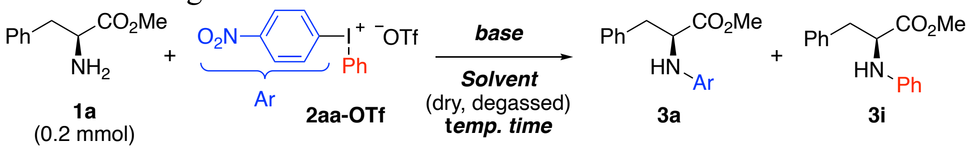

**2aa-OTf** (equiv)	**Base** (equiv)	**Solvent** (mL)	**T** [°C]	**Time** [h]	**3a** (%)[a]	**3i** (%)[a]	recov **1a** (%)[a]
1.2	NaOtBu (1.2)	CH_2Cl_2 (1)	40	18	*n.d.*	*n.d.*	81
1.2	NaO*t*Bu (1.2)	THF (1)	60	18	*n.d.*	*n.d.*	46
1.2	NaOtBu (1.2)	MeOH (1)	60	18	*n.d.*	*n.d.*	59
1.2	NaO*t*Bu (1.2)	EtOAc (0.7)	60	18	*n.d.*	*n.d.*	37 (34)
1.0	NaO*t*Bu (1.0)	Toluene (1)	110	22	*n.d.*	-	77
1.2	Na_2CO_3 (1.2)	CH_2Cl_2 (1)	40	18	*n.d.*	*n.d.*	>95
1.2	Na_2CO_3 (1.2)	THF (1)	60	18	*n.d.*	*n.d.*	58
1.2	Na_2CO_3 (1.2)	CH_2Cl_2:H_2O (1)	60	18	*n.d.*	*n.d.*	46
1.2	Na_2CO_3 (1.2)	H_2O (1)	60	18	*n.d.*	*n.d.*	0
1.2	Na_2CO_3 (1.2)	MeOH (1)	60	18	*n.d.*	*n.d.*	52
1.2	Na_2CO_3 (1.2)	EtOAc (1)	60	18	*n.d.*	*n.d.*	72
1.0	Na_2CO_3 (1.0)	MeCN (1)	90	4	3	*n.d.*	65
1.0	Na_2CO_3 (1.0)	MeCN (1)	110	4	8	*n.d.*	50
1.0	Na_2CO_3 (1.0)	Toluene (1)	110	22	16 (15)	*n.d.*	65 (49)
1.0	Na_2CO_3 (1.0)	Toluene (1)	130	22	(28)	*n.d.*	(>80)[b]
1.0	Na_2CO_3 (1.0)	p-xylene (1)	130	22	(30)	*n.d.*	(45)
1.0	Na_2CO_3 (1.0)	2,6-Lutidine (1)	130	22	*n.d.*	*n.d.*	40
1.0	Na_2CO_3 (1.0)	DMF (1)	130	22	*n.d.*	*n.d.*	38
**1.0**	**Na_2CO_3 (1.0)**	**Toluene (1)**	**150**	**4**	**47 (45)**	**(6)**	**34**
1.0	Na_2CO_3 (1.0)	p-xylene (1)	150	4	37	6	32
2.0	Na_2CO_3 (1.0)	2,6-Lutidine (1)	150	4	*n.d.*	*n.d.*	visible
1.0	Na_2CO_3 (1.0)	Pyridine (1)	150	4	*n.d.*	*n.d.*	51
1.0	Na_2CO_3 (1.0)	Toluene (0.5)	150	4	43	5	36
1.0	Na_2CO_3 (1.0)	Toluene (2.0)	150	4	36	4	36

[a] ^{1}H NMR yield compared to trimethoxybenzene (TMB) as internal standard (IS), (isolated yields in parentheses). [b] Isolated as a white solid instead as a yellow oil. *n.d.* = not detected.

Table S6 depicts reactions between **1a** and **2aa-OTf** in toluene at 150 °C with various bases. The use of a mild base was necessary to prevent aryne formation.

Table S6. Base screening.

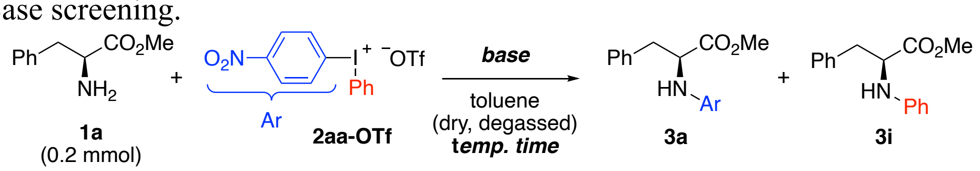

**2aa-OTf** (equiv)	**Base** (equiv)	**Time** [h]	**3a** (%)[a]	**3i** (%)[a]	recov **1a** (%)[a]
1.0	Na_2CO_3 (1.0)	4	47 (45)	(6)	34
1.0	K_2CO_3 (1.0)	4	25	9	34
1.0	Li_2CO_3 (1.0)	4	22	8	24
2.0	Li_2CO_3 (1.0)	4	30	9	25
1.0	Cs_2CO_3 (1.0)	4	2	*n.d.*	60
2.0	Cs_2CO_3 (1.0)	4	14	2	68
1.0	Et_3N (1.0)	4	19	4	67
1.0	Na_2CO_3 (1.0) + Et_3N (0.5)	4	28	traces	0
1.0	TMG (1.0)	4	traces	*n.d.*	46
2.0	TMG (1.0)	4	18	3	visible
1.0	Na_2CO_3 (1.0)	2	42	13	31
1.0	Na_2CO_3 (1.0)	6	44	8	35
1.0	Na_2CO_3 (1.0)	8	44	13	30
1.0	Na_2CO_3 (1.0)	18	44	10	31
1.0	Na_2CO_3 (1.0)	24	44	8	32
1.0[b]	Na_2CO_3 (1.0)	4	46	9	36
1.0[c]	Na_2CO_3 (1.0)	4	42	8	37
1.0[d]	Na_2CO_3 (1.0)	4	35	7	38
1.0[e]	Na_2CO_3 (1.0)	4	37	11	33
1.5	Na_2CO_3 (1.0)	4	49	10	20
1.5	Na_2CO_3 (1.0)	24	49	11	11
**2.0**	**Na_2CO_3 (1.0)**	**4**	**59**	**12**	**0**
2.0	Na_2CO_3 (1.0)	24	40	traces	21
2.0	Na_2CO_3 (1.5)	4	40	10	10
**1.0**[f]	**Na_2CO_3 (2.0)**	**4**	**56**	**5**	**0**

[a] ^1H NMR yield compared to TMB as IS, (isolated yields in parentheses). [b] Wet and degassed toluene. [c] Wet and non-degassed toluene. [d] Anhydrous and non-degassed toluene. [e] Reaction in the presence of air. [f] 2.0 equiv of **1a** was used. *n.d.* = not detected.

4.1.2 Effect of the Counterion and Dummy Ligand in the Diaryliodonium Salt

See the discussion in the manuscript. Also TMP salt **2ad-OTs** resulted in significant lower yield. Using an anisyl ligand (**2ac-OTf**) gave best results, both with regards to the yield and to the chemoselectivity.

Table S7. Evaluation of the effect of the counterion and dummy-ligand.

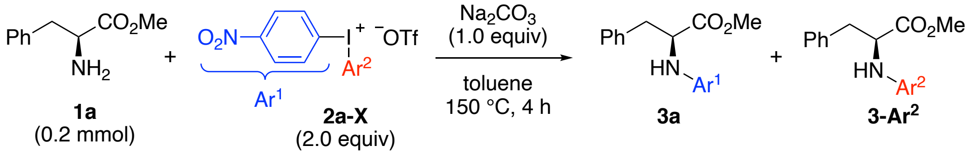

	Salt **2a-X**		**3a**	**3-Ar²**	recov **1a**
	**X**	**Ar²**	(%)[a]	(%)[a]	(%)[a]
**2aa-OTf**	OTf	Ph	59	12	*n.d.*
**2aa-BF₄**	BF_4	Ph	34	14	visible
**2aa-Br**	Br[b]	Ph	*n.d.*	*n.d.*	>99
**2aa-OTs**	OTs[c]	Ph	12 (14)	12	visible
**2ac-OTs**	OTs[c]	anisyl	40 (44)	7	22
**2ad-OTs**	OTs[c]	TMP	16	*n.d.*	visible
**2ac-OTf**	OTf	anisyl	85 (79)	*n.d.*	*n.d.*
**2ab-OTf**	OTf	Mes	49	10	25

[a] ^{1}H NMR yield compared to TMB as IS, (isolated yields in parentheses).[b] Obtained >95% **4-Br**. [c] Isolated >95% **4-OTs**. *n.d.* = not detected.

Further optimization of the conditions was done after uncovering the increased yield with 4-nitrophenyl(anisyl)iodonium triflate (**2ac-OTf**). The set-up did not deviate much from previous established conditions except that the temperature could be decreased to 130 °C. Using diaryliodonium salt batches that were synthesized without quenching with remaining acid with H_2O (see **Section 2.2, Method I**) resulted in lower yield, illustrating the sensitivity of the reaction. Applying the phenylalanine benzyl ester (**1b**) gave comparable yields with **1a** at both 150 °C and 130 °C.

Table S8. Optimization with 4-nitrophenyl(anisyl)iodonium triflate (**2ac-OTf**).

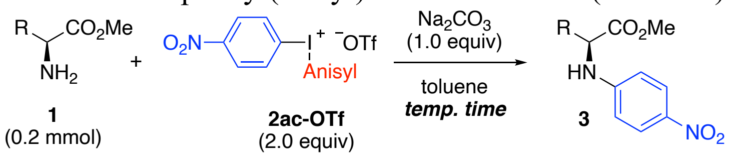

Substrate 1 R	Substrate 1 (equiv)	**2aa-OTf** (equiv)	**T** [°C]	**Time** [h]	**3** (%)[a]	recov **1** (%)[a]
Me	1.0	2.0	150	4	85 (79)	0
Me	1.0	2.0	150	2	83	0
Me	1.0	2.0	150	1	76	7
Me	1.0	1.5	150	4	66	0
Me[b]	2.0	1.0	150	4	73	>95
Me	1.0	2.0	130	4	82	0
Me	1.0	2.0	130	4	78 (77)	0
Me	1.0	2.0	150	4	73	>95
Me	1.0	2.0	110	24	60	11
Me[c]	1.0	2.0	150	2	63	0
Me[c]	1.0	2.0	150	1	64	0
Me[d]	1.0	2.0	150	4	53	19
Me[b, d]	2.0	1.0	150	4	57	>95
Bn	1.0	2.0	150	4	80 (75)	0
Bn	1.0	2.0	130	4	(78)	0

[a] ^{1}H NMR yield compared to TMB as IS, (isolated yields in parentheses).[b] 2.0 equiv base was used. [c] Diaryliodonium synthesized according to **Method I** (see **Section 2.2**). [d] Reaction in the presence of air. *n.d.* = not detected.

Arylations with diaryliodonium salts are in general insensitive to oxygen, but this reaction is clearly not. The yield of **3a** decreased from 85% to 53% in the presence of air (footnote d).
We have previously observed that reactions in the presence of oxygen can result in decreased yields for certain oxidation-sensitive substrate classes, like thioamides.[44]

4.1.3 Optimization with Electron-Rich Diaryliodonium Salts

The transfer of electron rich aryl groups is generally more difficult with diaryliodonium salts, they tend to be less reactive and give lower yields.[9a, 45] This trend was observed in phenylations with **2ia-OTf,** where reaction at 4 h gave incomplete conversion.

Table S9. Mini-optimization with diphenyliodonium triflate (**2ia-OTf**).

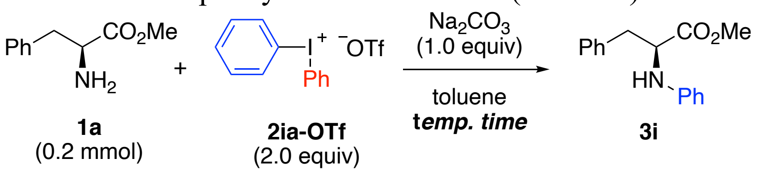

**2ia-OTf** (equiv)	**T** [°C]	**Time** [h]	**3i** (%)^a	recov **1a** (%)^a
1.0	110	22	10 (5)	80 (82)
1.0	130	22	(27)	(>90)
1.0	150	4	27	53
1.5	150	4	36	49
2.0	150	4	43	34
1.0	150	24	49	34
1.5	150	24	48	18
2.0	150	24	70 (67)	0
2.0	150	18	49 (44)	16

^{a} ^{1}H NMR yield compared to TMB as IS, (isolated yields in parentheses).

Reactions with bis(4-*tert*-butylphenyl)iodonium triflate (**2je-OTf**) showed the same outcome as **2ia-OTf** and needed prolonged reaction time. The reaction was found to be good yielding with both 1.5 and 2.0 equiv of salt **2je-OTf**.

Table S10. Mini-optimization with bis(4-*tert*-butylphenyl)iodonium triflate (**2je-OTf**).

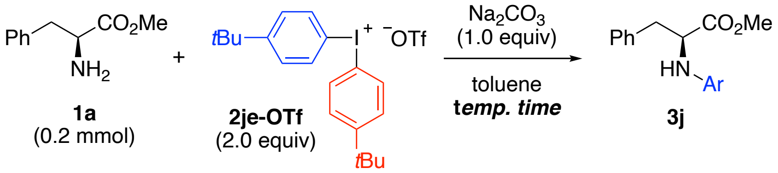

**2je-OTf** (equiv)	**T** [°C]	**Time** [h]	**3j** (%)^a	recov **1a** (%)^a
1.0	130	22	28	48
1.0	150	4	27 (32)	62
2.0	150	4	38	58
1.0	150	24	36	33
1.5	150	24	53	20
2.0	150	24	54 (50)	0
2.0	150	18	45 (48)	traces

^{a} ^{1}H NMR yield compared to TMB as IS (isolated yields in parentheses).

4.1.4 ^{1}H NMR Yield Determination with Internal Standard

All the reactions during the optimization were done on a 0.2 mmol scale and the NMR yields were determined by using 1,3,5-trimethoxybenzene (TMB) as internal standard (IS) as described below:

When the reaction time was over, the crude reaction was removed from the oil bath and allowed to cool down to rt. TMB (3.4 mg, 0.02 mmol, 0.1 equiv) was added followed by water (2 mL). The solution was extracted with EtOAc (3×2-3 mL). The solvent was removed under reduced pressure and $CDCl_3$ was added to the residue.

The yield was determined by comparing the ^{1}H NMR integration of the singlet generated by TMB ArH (3H, 6.08 ppm, set to 0.3) to the α-H of **3a** (CH_2-CH(-NH-Ar)-CO_2Me). In the example given in **Figure S4**, the yield of **3a** was 46%, and **3i** was generated as side-product in 5% using salt **2aa-OTf**.

Figure S4. NMR yield of **3a**.

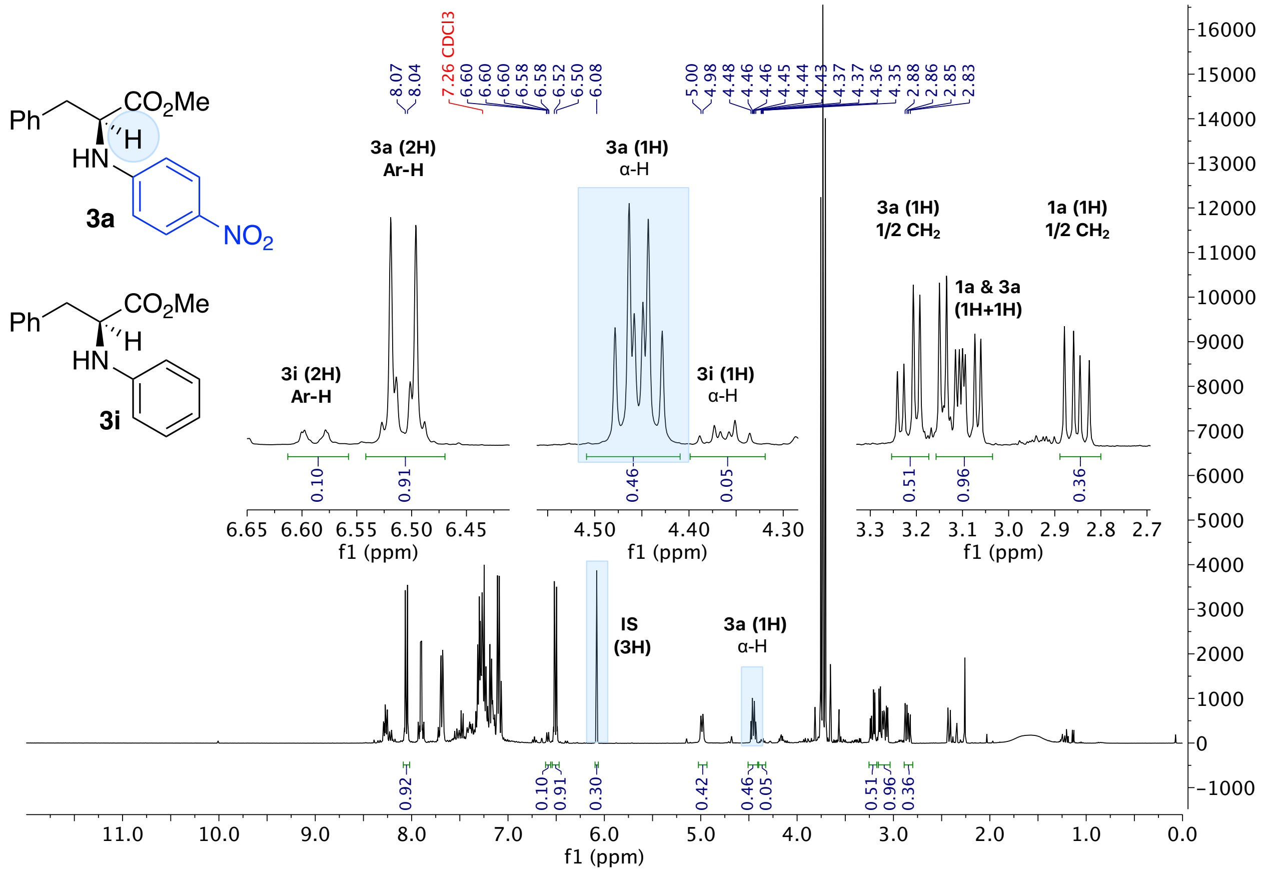

4.2 Arylation of Phenylalanine Ester Precursors

Since phenylalanine methyl ester proved prone to react with itself upon storage,[30] it was necessary to remove the TFA or HCl salt from the amino acid esters shortly prior to use. With the attempt to simplify the set-up and prevent additional preparation steps for each arylation reaction experiments on the phenylalanine methyl ester salt and phenylalanine (in its carboxylic acid form) were done.

4.2.1 Arylation of Phenylalanine Methyl Ester Hydrochloride Salt

Subjecting the hydrochloride salt of phenylalanine methyl ester (**1a-HCl**) to the reaction conditions was unsuccessful (**Scheme S1a**). The yield is reported as ^{1}H NMR yield (compared to TMB as IS) with only 3% of the desired arylated product **3a**. In the reaction, the chloride ion from the HCl salt acted as a competing nucleophile and **4-Cl** was the major side-product. Furthermore, 90% of the deprotonated phenylalanine methyl ester was obtained. The reaction had a good mass balance and the amino acid derivative remained intact. This result indicates that the decomposition of the starting material could be caused by the iodonium salt. Iodine(III) compounds have a high oxidation potential and are often used as oxidants.[2a-c] At the elevated temperatures applied in our reaction, some phenylalanine methyl ester could have been oxidized by the diaryliodonium salt instead of the desired arylation. The reaction was repeated with iodonium salt **2ac-OTf** with similar results (**Scheme S1b**). The last reaction shows that the mass balance deteriorates with excess iodonium salt, and only modest amounts of **3a** are formed.

Scheme S1. Reaction with phenylalanine methyl ester ammonium hydrochloride salt.

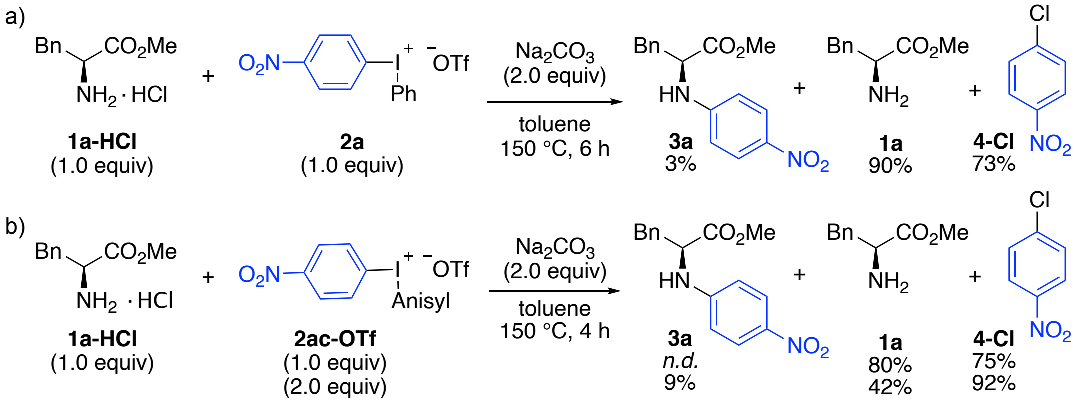

4.2.2 Arylation of Phenylalanine

Arylation of phenylalanine in its carboxylic acid form resulted neither *N*- nor *O*-arylated product (**Scheme S2**). The crude was treated with extraction work-up. ^{1}H NMR analysis of the organic phase showed no arylation product. The water phase was collected, and solvent was removed under high pressure generating a mixture of white solids. HRMS analysis of materials recovered from the water phase revealed only the amino acid. Previous reports by our group had confirmed that carboxylic acids are *O*-arylated at reflux in toluene (however with a stronger base *t*BuOK),[11a, 12c] so the absence of any *O*-arylated product **Phe-*O*** was unexpected.

Scheme S2. Attempted arylation of phenylalanine and **2aa-OTf**.

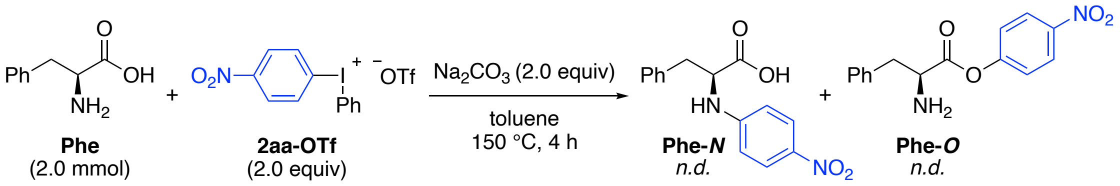

4.3 Chemoselectivity Study

To avoid product mixtures in arylation with unsymmetric diaryliodonium salts, a high chemoselectivity is very important.[46] Examples of commonly used dummy ligands are given in **Figure S5** and the general rule is that the dummy has to be more electron-donating compared to the aryl involved in transition metal-free aryl transfer.

Figure S5. Examples of dummy ligands.

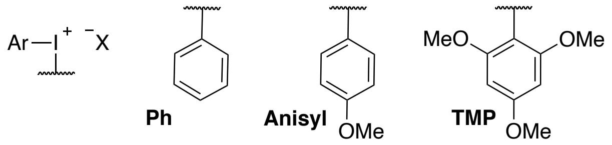

Surprisingly, moderate chemoselectivity was observed with 4-nitrophenyl(phenyl)iodonium triflate (**2aa-OTf**) in the *N*-arylation of phenylalanine methyl ester (**1a**). The desired product **3a** was obtained together with the phenylated product **3a** (see **Table S5** and **S6**). This was peculiar as **2aa-OTf** gives complete chemoselectivity in reactions with a range of nucleophiles.[2c, 8-9] This problem was solved by using an anisyl dummy, which ensured a greater electronic difference between the ligands in the salt.

4.3.1 Electronic Effect of the Dummy Ligand

The same chemoselective problem was observed in arylations with 4-cyanophenyl(phenyl)iodonium triflate **2ba-OTf,** but the desired product was obtained in higher yield (**Table S11**). Using an anisyl dummy (**2bc-OTf**) slightly increased the yield and no anisyl byproduct **3-Ar²** was observed. Similar yields were obtained at 130 °C and applying the phenylalanine benzyl ester gave comparable yields at both 150 °C and 130 °C. Reaction with a diaryliodonium batch synthesized without quenching with remaining acid with H_2O (see **Section 2.2**, **Method I**) resulted in slightly lower yield.

Table S11. Chemoselectivity evaluation in the synthesis of **3b**.

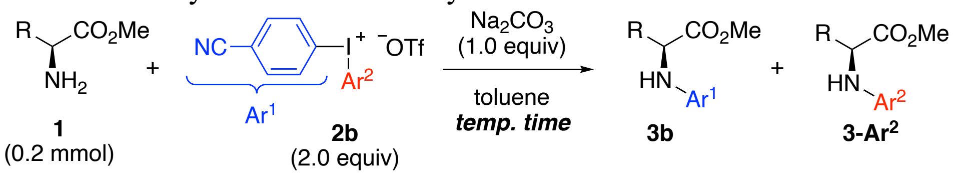

R	2b	Ar²	T [°C]	3b (%)^a	3-Ar² (%)^a	recov 1 (%)^a
Me	**2ba**	Ph	150	70	13	12
Me	**2ba**	Ph	150	73	9	9
Me	**2bc**	Anisyl	150	78 (77)	*n.d.*	0
Me	**2bc**	Anisyl^b	150	66 (62)	*n.d.*	0
Me	**2bc**	Anisyl	130	82 (80)	*n.d.*	0
Bn	**2bc**	Anisyl	150	(82)	*n.d.*	7
Bn	**2bc**	Anisyl	130	(84)	*n.d.*	0

^{a} ^{1}H NMR yield compared to TMB as IS, (isolated yields in parentheses).^{b} Diaryliodonium synthesized according to **Method I** (see **Section 2.2**). *n.d.* = not detected.

Reactions with 3-trifluoromethylphenyl(phenyl)iodonium triflate (**2ea**) gave moderate yields and showed a greater chemoselectivity problem compared to aryl transfer with **2aa-OTf** and **2ba-OTf**. The increased formation of byproduct **3-Ar²** is due to reduced electronic difference between the two ligands. The chemoselectivity problem was again solved by using salt **2ec** with an anisyl dummy, however, the yield of the desired product **3e** did not increase even upon prolonged reaction time.

Table S12. Chemoselectivity evaluation in the synthesis of **3e**.

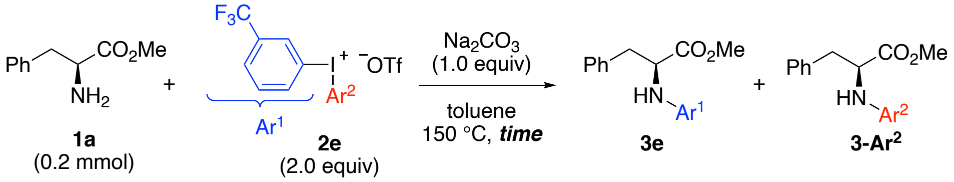

**2e**	**Ar²**	**Time** [h]	**3e** (%)[a]	**3-Ar²** (%)[a]	recov **1a** (%)[a]
**2ea**	Ph	4	50	27	20
**2ea**	Ph	24	43 (41)	15	20
**2ec**	Anisyl	4	50	*n.d.*	23
**2ec**	Anisyl[b]	4	49	*n.d.*	24
**2ec**	Anisyl	4	(50)	*n.d.*	visible
**2ec**	Anisyl	24	55 (54)	*n.d.*	15

[a] ^{1}H NMR yield compared to TMB as IS, (isolated yields in parentheses). [b] Excess amino acid derivative and base were used (2.0 equiv). *n.d.* = not detected.

Transition metal-free arylation with electron-rich ligands tends to be lower yielding compared to aryl transfer of electron-poor ligands. This was also the case in *N*-arylation of amino acid derivatives (see **Table S9** and **Table S10**). Using an anisyl dummy did not aid the aryl transfer and a lower yield was observed with the phenyl(anisyl)iodonium triflate (**2ic-OTf**),which is an opposite trend compared to transfer of electron-poor aryls.

Table S13. Chemoselectivity evaluation in the synthesis of **3i**.

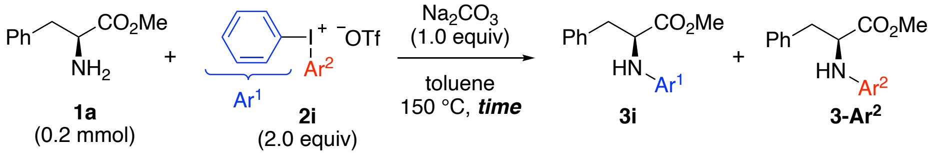

**2i**	**Ar²**	**Time** [h]	**3i** (%)[a]	**3-Ar²** (%)[a]	recov **1a** (%)[a]
**2ia**	Ph	4	50	-	37
**2ia**	Ph	24	70 (67)	-	0
**2ic**	Anisyl	4	28	*n.d*	57
**2ic**	Anisyl	24	43	*n.d*	21

[a] ^{1}H NMR yield compared to TMB as IS (isolated yields in parentheses). *n.d.* = not detected.

Arylation with bis(4-*tert*-butylphenyl)iodonium triflate (**2je-OTf**) showed the same trend as the phenylation, where the corresponding unsymmetrical salt **2jc-OTf** gave lower yield.

Table S14. Chemoselectivity evaluation in the synthesis of **3j**.

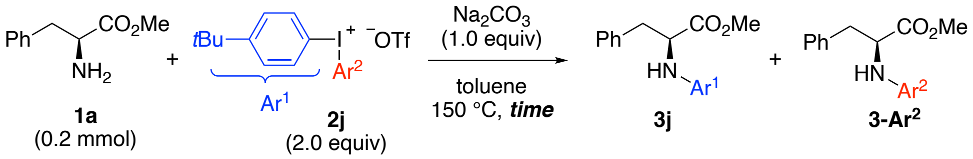

**2j**	**Ar²**	**Time** [h]	**3j** (%)[a]	**3-Ar²** (%)[a]	recov **1a** (%)[a]
**2ja**	Ar¹	4	38	-	58
**2ja**	Ar¹	24	54 (49)	-	not determined
**2jc**	Anisyl	4	38	*n.d.*	0
**2jc**	Anisyl	24	42	*n.d.*	0

[a] ^{1}H NMR yield compared to TMB as IS (isolated yields in parentheses). *n.d.* = not detected.

4.3.2 Electronic vs Steric Preferences

A series of reactions were run to analyze the influence of *ortho*-substituents, and an *ortho*-effect could be observed (**Scheme S3**). Furthermore, arylation with *meta*- and *ortho*-substituted ligands gave higher yields compared to the *meta*-substituted counterpart (see the scope of **1a** in the manuscript).

Scheme S3. Examples of steric effects in arylation of **1a** & **5a**.

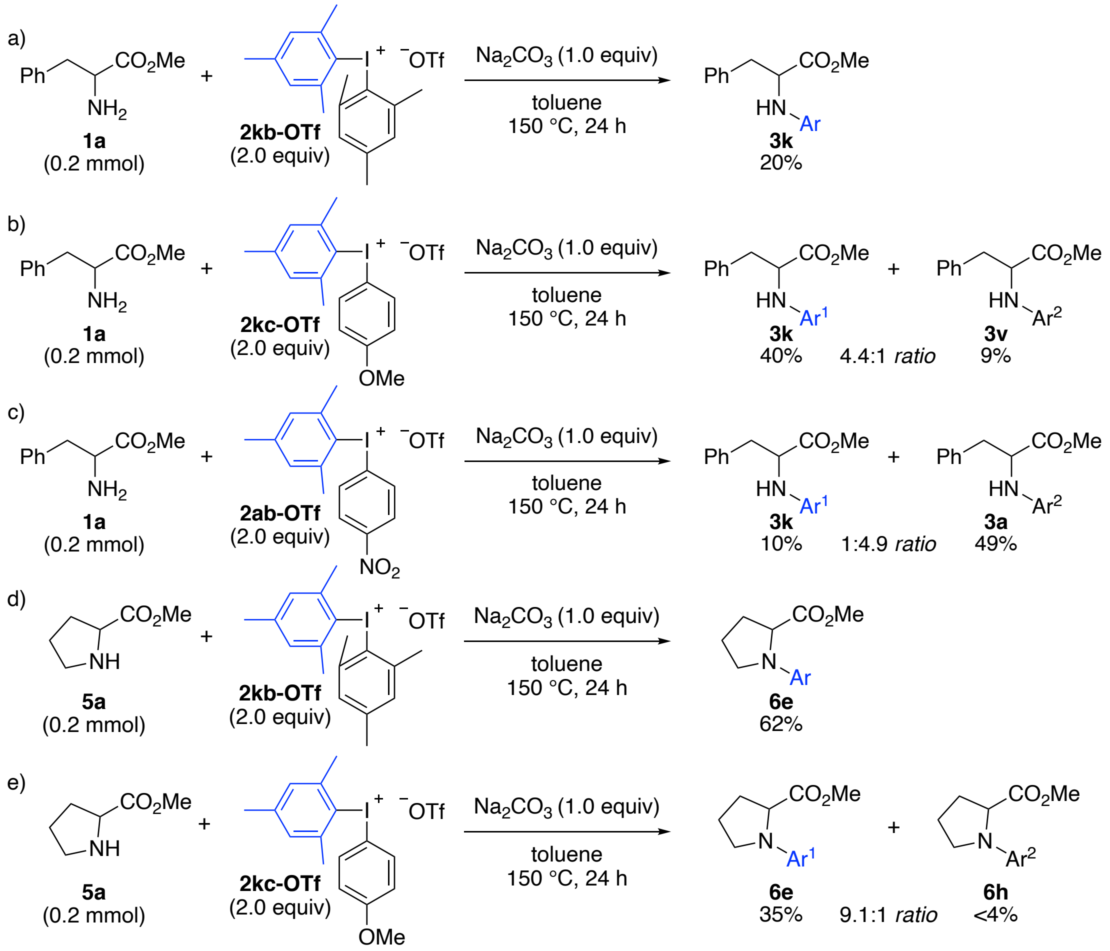

4.4 Further Studies

4.4.1 Mechanistic Experiments

Apart from regular ligand coupling mechanism, diaryliodonium salts can form radicals[47] or arynes,[48] which can be used for further transformation. Furthermore, a mechanistic study published by our group showed that the substituents on the ligands of the diaryliodonium salt had a strong influence on the reaction outcome and could result in a regioisomeric mixture.[49]

A way to control whether the arylation with diaryliodonium salts follows a radical pathway is by the addition of a radical scavenger, for example 1,1-diphenylethylene (DPE).[50] Furan can be used as aryne trap, as Diels-Alder adducts easily form.[49] Especially arylations with *p*-substituted electron-rich iodonium salts tend to give regioisomeric product mixtures when an aryne mechanism competes with the ligand coupling.

Reactions with DPE showed no effect of the reaction outcome, but the yield declined in reactions with furan (**Table S15**). No Diels-Alder adduct was observed in the reaction and the reason for the lower yield is probably that furan acts as a co-solvent. These results shows that the reaction most likely does not follow aryne or radical pathways.

Table S15. Initial mechanistic investigation.

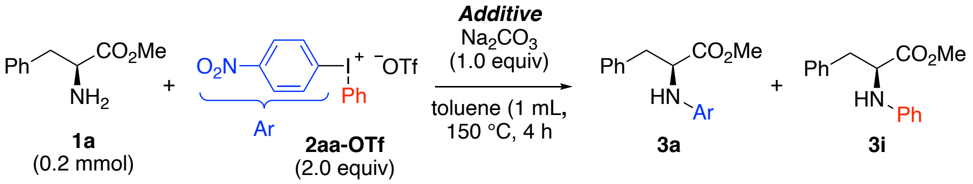

**Additive** (equiv)	**3a** (%)^{a}	**3i** (%)^{a}	recov **1a** (%)^{a}
-	59	12	0
DPE (2.0)	60	10	-
Furan (5.0)^{b}	35 (34)	11	>22

^{a} ^{1}H NMR yield compared to TMB as IS (isolated yields in parentheses). ^{b} No Diels-Alder adduct observed.

Since the reaction takes place at elevated temperature, nucleophilic aromatic substitution (SNAr) becomes a possible competing pathway. Therefore, two different aryl iodides were reacted with **1a** in order to exclude *C-N* bond formation through this mechanism (**Scheme S4**). Reaction at 150 °C for 4-24 h resulted in quantitative recovery of **1a**, which shows that **1a** is stable at elevated temperature *in the absence of the diaryliodonium salt*. The problem with the mass balance in previous reactions (**Table S5** and **Table S6**) could be explained by the high oxidation potential of hypervalent iodine,[2a-c] causing the amino acid derivative to oxidize.

Scheme S4. SNAr investigation.

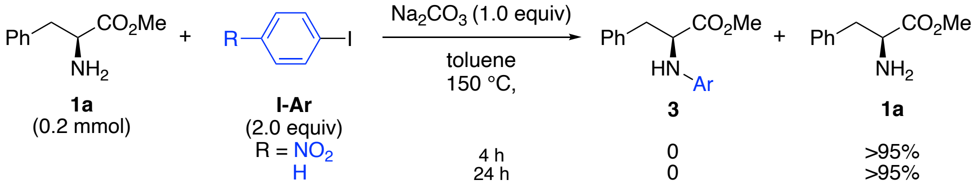

Stability Tests

The product stability was investigated by subjecting **3a** to the reaction conditions (**Scheme S5a**). No diarylated product was formed, and the *ee* of **3a** remained intact, demonstrating that the arylated product is stable to racemization under the reaction conditions. However, **3a** could only be recovered in 68% yield, indicating a partial decomposition of **3a** had occurred.

The stability of the diaryliodonium salt **2ac-OTf** was also investigated. Our group have previously reported successful arylation at 110 °C[9a], [11a] and it is the first time we have raised the temperature to 150 °C. In the absence of any amino acid ester, **2ac-OTf** showed partial decomposition at 150 °C and 51% could be recovered (**Scheme S5b**). Both iodoanisole and 4-iodonitrobenzene was visible by ^{1}H NMR of the crude but no anisole or nitrobenzene was observed.

Interestingly, in the absence of both amino acid ester and base, total decomposition of the iodonium salt occurred and iodoarene was observed together with unknown impurities (**Scheme S5c**).

Scheme S5. Stability test of **3a** and **2ac-OTf**.

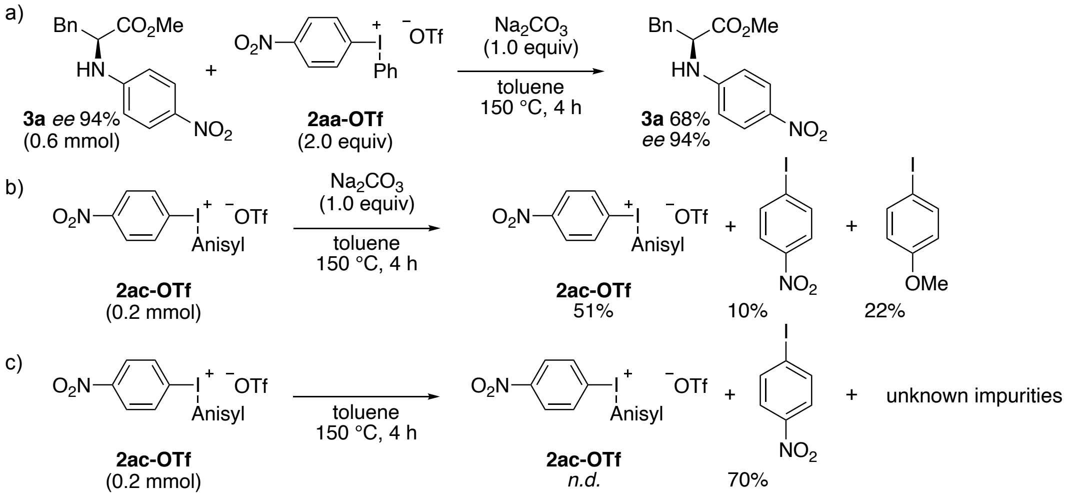

4.4.2 Protecting Group Compatibility

N-protected derivatives of **1a** showed poor reactivity (**Scheme S6**). Boc-protected amino ester gave no arylated Boc-product and 82% of the substrate was recovered, nonetheless, 9% of **3o** was isolated, meaning that the Boc-group had been partially cleaved off. Acetyl- and tosyl-protected substrates remained untouched without detection of any arylated products and could be recovered.

Scheme S6. Arylation of *N*-protected phenylalanine esters.

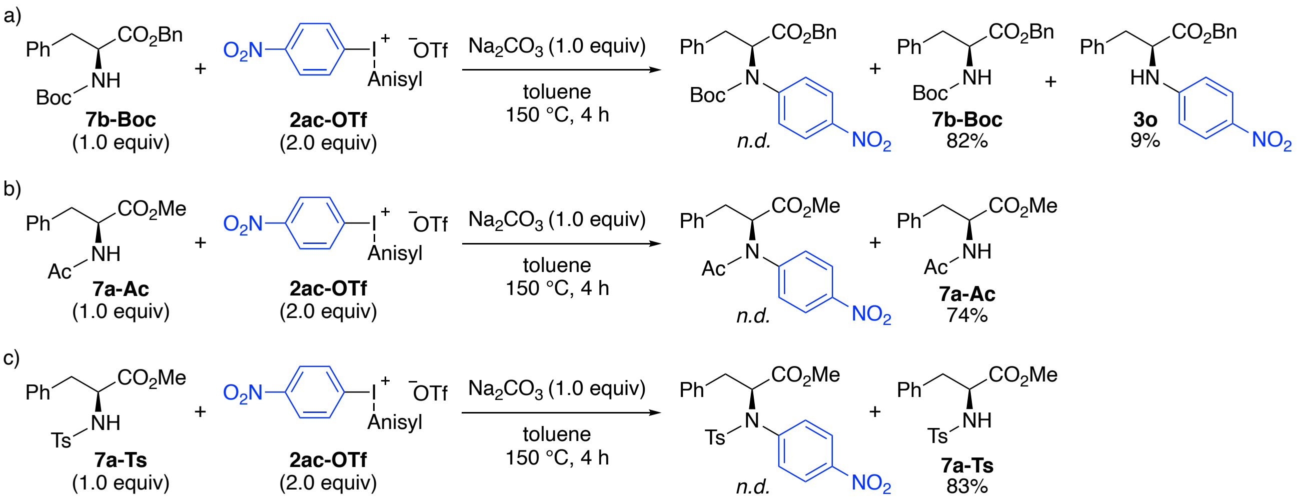

4.5 Limitations

Amino acid derivatives with a low molecular weight, such as alanine and valine methyl ester (**1e** and **1f**), proved to be volatile under high vacuum and elevated temperature, making them difficult to use under our reaction conditions (**Scheme S7a**). Other compounds with light molecular weight exhibited similar trends, and deprotonation of **1i** was troublesome, which made the substate not suitable for the reaction. On the other hand, arylation of the γ-amino acid derivative **5d** resulted in 12% product.

Heteroatom substituents are usually well tolerated in arylations with diaryliodonium salts, but it proved difficult to arylate amino acids with coordinative and competing nucleophilic nature of functional groups. Tryptophan methyl ester **1g** could be arylated in 15% yield, which might be increased through *N*-protection of the indole (not attempted). Arylation with methionine methyl ester **1i** yielded the expected product together with unknown side products. Hypervalent iodine(III) reagents have previously been used to oxidize sulfur compounds and been observed to cause undesired oxidation in ligand transfer reactions,[51] which could explain the mixture of products obtained.

We tried to deprotonate histidine methyl ester hydrochloride (**1k-HCl**) following two different procedures, but without success (**Scheme S7b**).[52] Reaction with Et_3N gave no free histidine, while using the stronger NaOMe base did lead to some desired product. However, upon scaling up, the reaction could not be reproduced. Furthermore, undesired racemization of the product will most likely occur with the use of NaOMe.

Scheme S7. Limitations with amino acid derivatives.

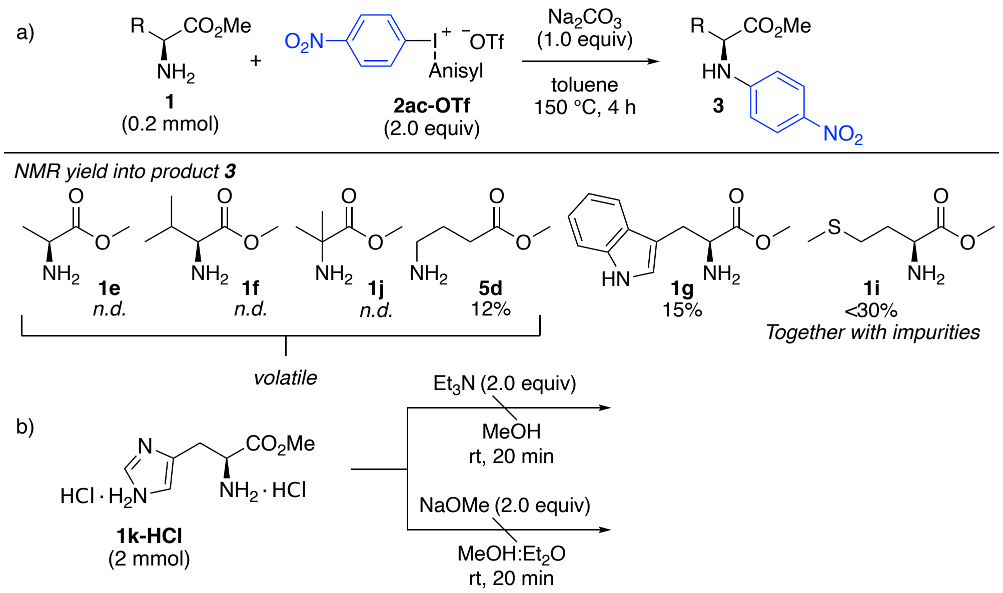

Scheme S8 depicts diaryliodonium salts that gave unsatisfactory results in the arylation. The symmetric anisyl **salt 2pe-OTf** gave small amounts of product. A similar outcome was observed with pyrazole salt **2rc-OTf**, however, changing the dummy-group from anisyl to meistyl resulted in no product formation. Two sterically hindered iodonium salts were also employed, with no *C-N* bond formation with the desired aryl group. In the reaction with the halogen substituted ligand, we unexpectedly observed aryl transfer of the meistyl dummy-ligand.

Reactions with unsymmetric methoxy salt **2pc-OTf** surprisingly proceeded without chemoselectivity (**Scheme S8b**). This iodonium salt has been used with other nucleophiles to give selective transfer of the *m*-OMe aryl moiety,[9a] but the conditions for *N*-arylation of amino acid esters yielded the two *N*-arylated compound in a 1:1 ratio.

The successful arylation with 6-membred cyclic diaryliodonium salt **2n-OTf**, which yielded 59% of the iodo-substituted product **3n**, was surprising as reactions with cyclic diaryliodonium salts generally require transition metal-catalysis due to decreased reactivity.[53] Due to this we also tried arylation with the 5-membred cyclic diaryliodonium salt **2o-OTf**, however, no product was formed (**Scheme S8c**).

Scheme S8. Limitations with diaryliodonium salts.

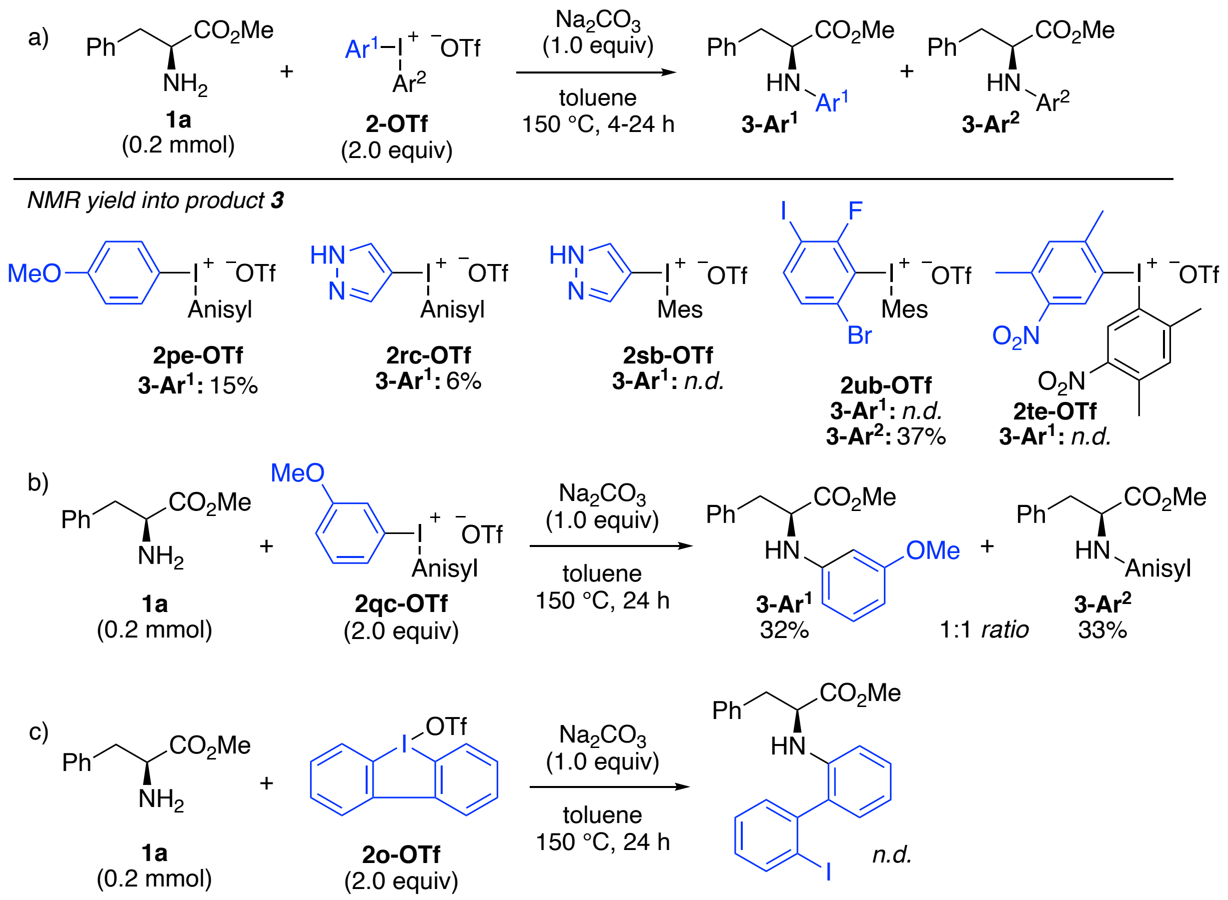

4.6 Preparation of Racemic Samples for ee Analysis

Racemic samples for SFC or HPLC analysis were either prepared through *N*-arylation of racemic starting material or via basic treatment of the *N*-arylated product.

4.6.1 Synthesis of Racemic Starting Material (Rac I)

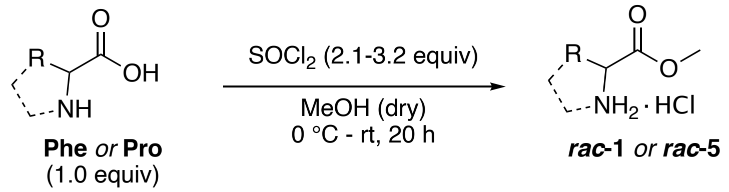

Procedure:
The esterification was performed according to **GP1** (see **Section 3.1**).[21] The free amino acid was obtained by basic extraction according to **Section 3.4**, and the arylation was performed according to **GP7** (see **Section 4.8**).

Table S16. Synthesis according to **Rac I**

***rac*-1a-HCl**	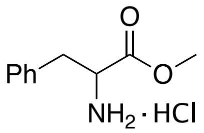	*rac*-Phe-OMe HCl[31a] (***rac*-1a-HCl**) was prepared in 99% with 2.1 equiv $SOCl_2$ according to literature reports.[21] The free amino acid was obtained by basic extraction following **GP4** (**Section 3.4**).
***rac*-5a-HCl**	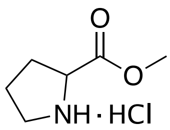	*rac*-Pro-OMe HCl (***rac*-5a-HCl**) was prepared in 98% with 3.2 equiv $SOCl_2$ according to literature reports.[54] The free amino acid was obtained by basic extraction following **GP6** (**Section 3.4**).

4.6.2 Racemization of *N*-Arylated Compounds (Rac II)

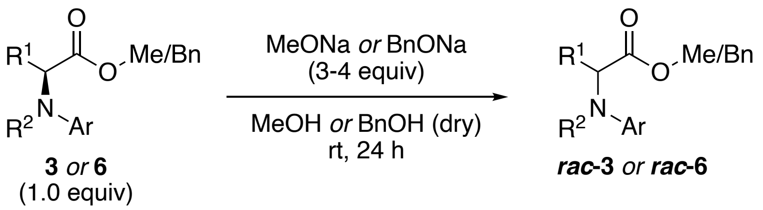

Procedure:
To a dry round bottomed flask, *N*-aryl amino acid derivative **3** or **6** (1.0 equiv) and NaOMe *or* NaOBn (3-4 equiv) was dissolved in anhydrous MeOH *or* BnOH (0.05 M). The reaction was left to stir over night at rt then EtOAc (15 mL) was added and the mixture was washed with H_2O (3×15 mL). No further purification was done with reactions in MeOH. Reactions in BnOH were purified by column chromatography (*n*-pentane/EtOAc as eluent).

4.7 Substrate Scope of The Amino Acid Esters

The scope of the reaction is further discussed in the manuscript. An overview is given in **Scheme S9**. For further experimental information see **Section 4.8.**

Ee analysis was only done with chiral SFC and HPLC on arylations with **1a**, **1h**, **5a** and **5b.** Aryl transfer of electron-poor ligands did not have a negative impact on existing stereocenter and the products were isolated in excellent *ee's*. Very little racemization occurred in reaction with electron-rich diaryliodonium salts despite the prolonged reaction time, and *ee's* of ≥ 95% were observed in products **3i**, **3j** and **3k**. Unfortunately, arylated products **3l**, **3m** and **3n** were obtained with lower *ee*. Arylation of secondary amino acid ester, such as proline and *N*-methyl-*L*-phenylalanine, were more prone to racemization and the *ee's* of the final product varied (90% – 94% *ee*).

Since phenylation and transfer of *p*-NO_2, *p*-CN and *p*-*t*Bu substituted aryls gave products with excellent *ee*s, SFC analysis was done on product **3o** from benzyl ester **1b**.

Scheme S9. Substrate scope of amino acid esters and diaryliodonium salts.

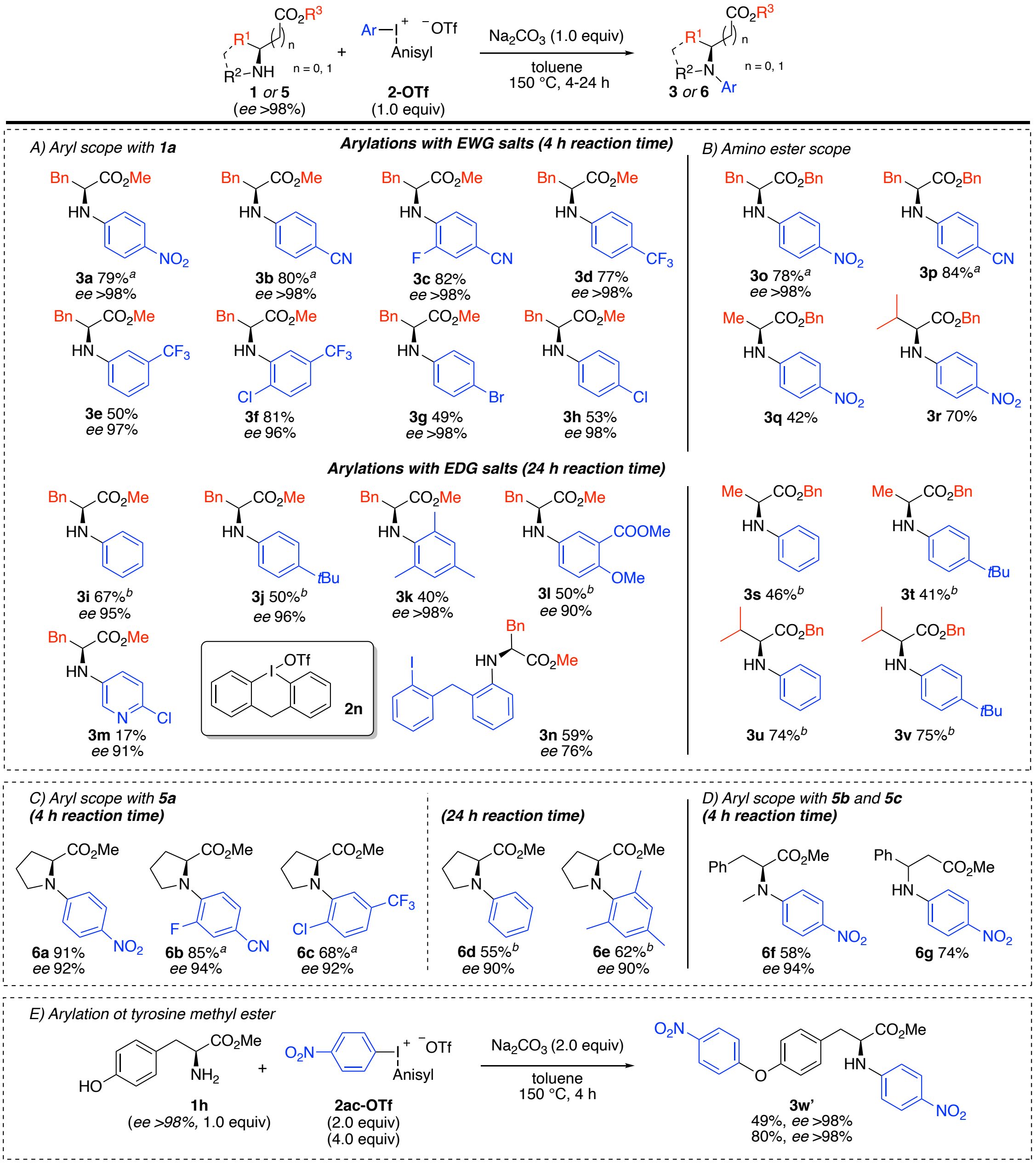

4.8 ARYLATION OF PRIMARY AMINO ACID ESTERS

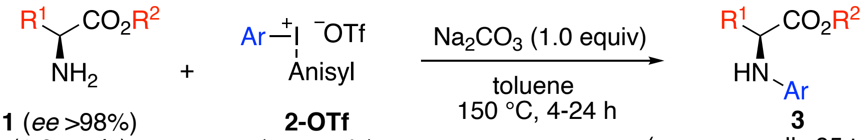

1 (*ee* >98%) (1.0 equiv) **2-OTf** (2.0 equiv) **3** (*ee:s* generally 95 to >98%)

General Procedure 7 (GP7):

Freshly prepared amino acid ester **1** (0.2 mmol), salt **2-OTf** (0.4 mmol, 2.0 equiv) and Na_2CO_3 (0.2 mmol, 1.0 equiv) were added to an oven-dried and pressure stable microwave vial. The MW tube was well sealed with a MW-cap and dried under vacuum for ca 15 min. The air was exchanged with argon 3-4 times followed by the addition of anhydrous toluene (1 mL, degassed by bubbling with argon for ca 20 min). The vial was placed in a preheated oil bath at 150 °C and was stirred for either 4 or 24 h. After completion of the reaction, the vial was cooled to rt and Celite was added. The volatiles were removed under reduced pressure, and the mixture was purified by column chromatography (SiO_2 with *n*-pentane/EtOAc as eluent system) to provide **3**.

***N*-4-nitrophenyl-*L*-phenylalanine methyl ester (3a)**

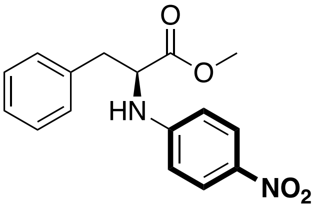

Synthesized according to **GP7** with phenylalanine methyl ester (**1a**, 0.2 mmol, 36 mg), diaryliodonium salt **2ac-OTf** (0.4 mmol, 203 mg) and Na_2CO_3 (0.2 mmol, 22 mg), 4 h reaction time. Purification provided **3a** (0.16 mmol, 48 mg, 79%) as a bright yellow oil.
*Reaction at 130 °C gave **3a*** in 77% *(0.15 mmol, 46 mg).*
R_f = 0.23 (*n*-pentane/EtOAc, 9:1). ^{1}H NMR (400 MHz, $CDCl_3$): δ 8.12 – 7.99 (m, 2H), 7.38 – 7.21 (m, 3H), 7.17 – 7.04 (m, 2H), 6.61 – 6.44 (m, 2H), 5.00 (d, *J* = 8.1 Hz, 1H), 4.47 (dt, *J* = 8.1, 5.7 Hz, 1H), 3.75 (s, 3H), 3.23 (dd, *J* = 13.8, 5.7 Hz, 1H), 3.14 (dd, *J* = 13.8, 6.2 Hz, 1H). ^{13}C NMR (101 MHz, $CDCl_3$): δ 172.2, 151.6, 139.0, 135.4, 129.3, 128.9, 127.5, 126.4, 111.9, 56.8, 52.7, 38.3; HRMS (ESI): calcd for $C_{16}H_{16}N_2O_4$ $[M+Na]^+$: 323.1002; found: 323.1004.

Analysis of enantiomeric purity:

The racemic sample was obtained via **Rac II** (0.5 mmol scale, 70% yield).
Chiral SFC, Diacel OJ-H (0.3 cm ϕ, 15 cm column), 25 °C, [10% MeOH in CO_2, 0.8 mL/min]; t_R: 12.51 min (minor enantiomer), 15.33 min (major enantiomer); *ee* = >98% (e.r. = 100:0).

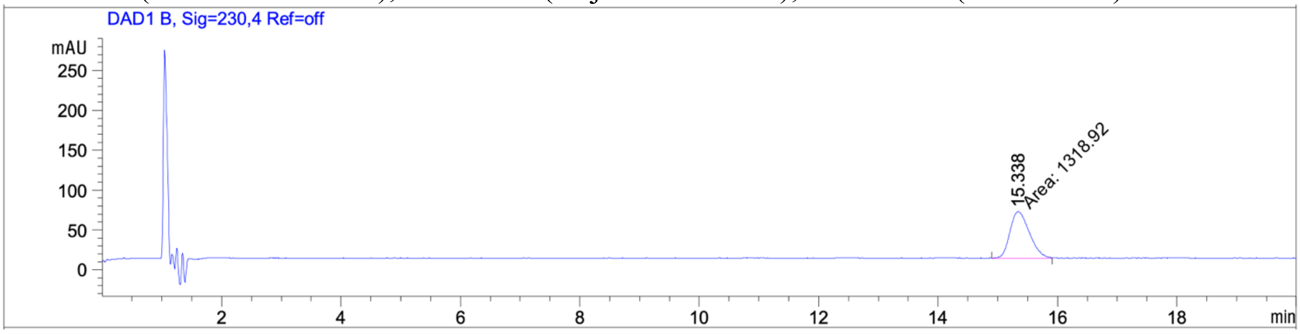

Signal 2: DAD1 B, Sig=230,4 Ref=off

Peak #	RetTime [min]	Type	Width [min]	Area [mAU*s]	Height [mAU]	Area %
1	15.338	MM	0.3770	1318.92236	58.30536	100.0000

Totals : 1318.92236 58.30536

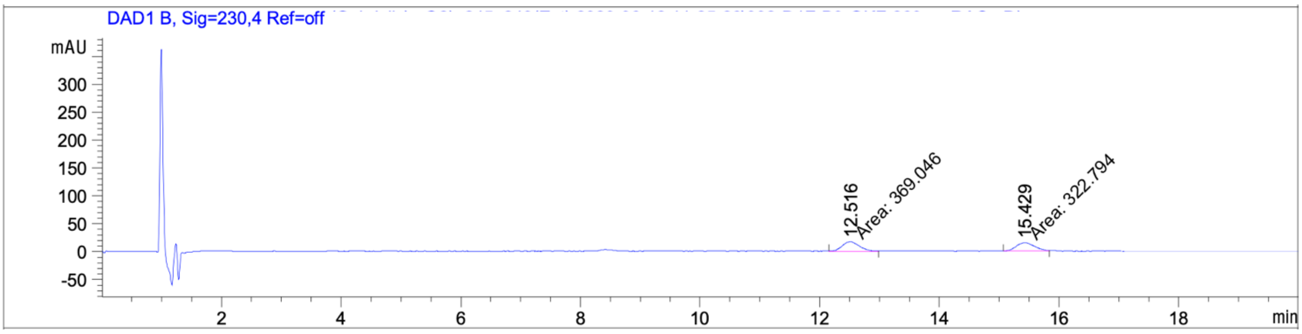

Signal 2: DAD1 B, Sig=230,4 Ref=off

Peak #	RetTime [min]	Type	Width [min]	Area [mAU*s]	Height [mAU]	Area %
1	12.516	MM	0.3529	369.04645	17.43142	53.3427
2	15.429	MM	0.3618	322.79422	14.87056	46.6573
Totals :				691.84067	32.30199	

N-4-cyanophenyl-*L*-phenylalanine methyl ester (3b)

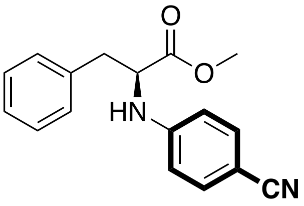

Synthesized according to **GP7** with phenylalanine methyl ester (**1a**, 0.2 mmol, 36 mg), salt **2bc-OTf** (0.4 mmol, 195 mg) and Na_2CO_3 (0.2 mmol, 22 mg), 4 h reaction time. Purification provided **3b** (0.15 mmol, 43 mg, 77%) as a yellow oil.

*Reaction at 130 °C delivered **3b** in 80% yield (0.16 mmol, 45 mg).*

R_f = 0.15 (*n*-pentane/EtOAc, 9:1). ^1H NMR (400 MHz, $CDCl_3$): δ 7.45 – 7.38 (m, 2H), 7.34 – 7.22 (m, 3H), 7.14 – 7.09 (m, 2H), 6.60 – 6.48 (m, 2H), 4.72 (d, J = 8.2 Hz, 1H), 4.40 (dt, J = 8.2, 6.0 Hz, 1H), 3.72 (s, 3H), 3.20 (dd, J = 13.7, 6.0 Hz, 1H), 3.11 (dd, J = 13.7, 6.0 Hz, 1H). ^{13}C NMR (101 MHz, $CDCl_3$): δ 172.5, 149.6, 135.6, 133.9, 129.3, 128.8, 127.4, 120.1, 113.0, 100.1, 56.8, 52.5, 38.3; HRMS (ESI): calcd for $C_{17}H_{16}N_2O_2$ $[M+Na]^+$: 303.1104; found: 303.1103.

Analysis of enantiomeric purity:

The racemic sample was obtained via **Rac I** followed by **GP7** (0.16 mmol scale, 76% yield).
Chiral SFC, Diacel OJ-H (0.3 cm ϕ, 15 cm column), 25 °C, [10% MeOH in CO_2, 0.8 mL/min]; t_R: 5.97 min (minor enantiomer), 7.55 min (major enantiomer); *ee* = >98% (e.r. = 99.4:0.6).

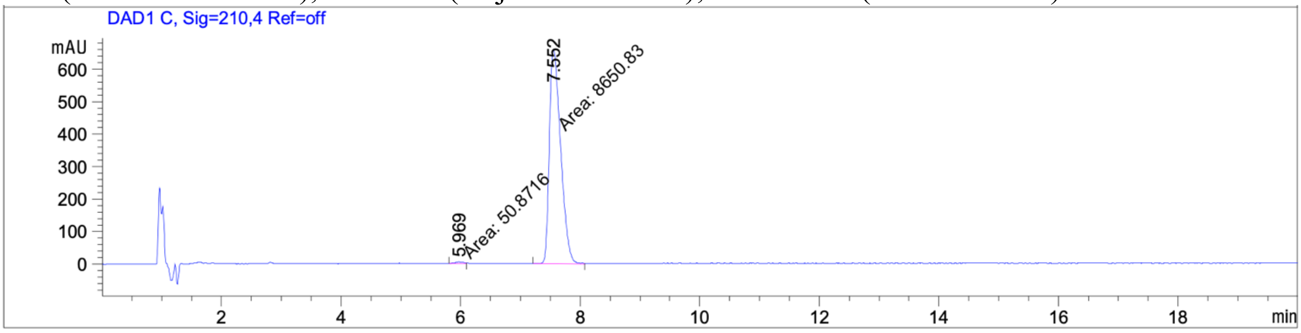

Signal 3: DAD1 C, Sig=210,4 Ref=off

Peak #	RetTime [min]	Type	Width [min]	Area [mAU*s]	Height [mAU]	Area %
1	5.969	MM	0.1742	50.87157	4.86782	0.5846
2	7.552	MM	0.2187	8650.82617	659.11322	99.4154
Totals :				8701.69775	663.98104	

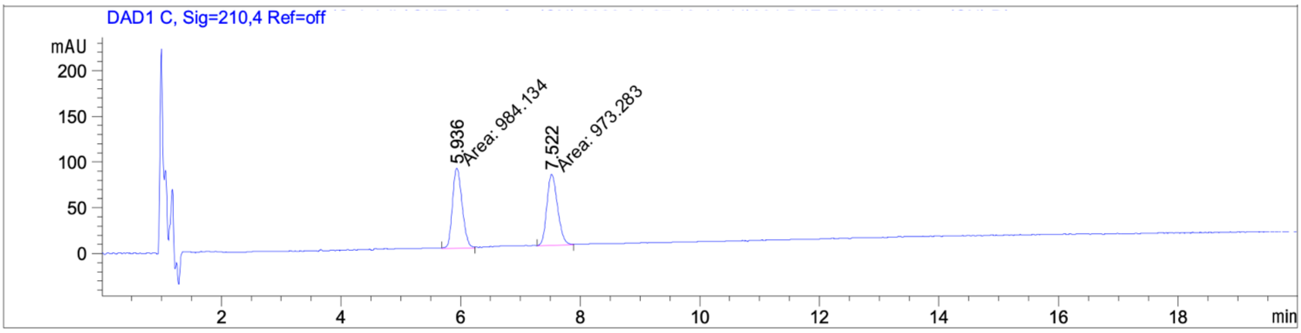

Signal 2: DAD1 B, Sig=230,4 Ref=off

Peak #	RetTime [min]	Type	Width [min]	Area [mAU*s]	Height [mAU]	Area %
1	5.935	MM	0.1927	135.11230	11.68792	50.1114
2	7.525	MM	0.2129	134.51180	10.52867	49.8886
Totals :				269.62410	22.21659	

_N_-4-cyano-2-fluorophenyl-_L_-phenylalanine methyl ester (3c)

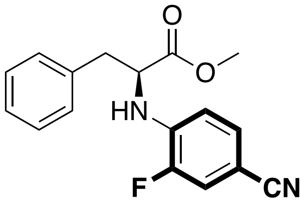

Synthesized according to **GP7** with phenylalanine methyl ester (**1a**, 0.17 mmol, 36 mg), diaryliodonium salt **2cc-OTf** (0.4 mmol, 202 mg) and Na_2CO_3 (0.2 mmol, 22 mg), 4 h reaction time. Purification provided **3c** (0.16 mmol, 49 mg, 82%) as a yellow oil.

R_f = 0.54 (*n*-pentane/EtOAc, 9:1). ^{1}H NMR (400 MHz, $CDCl_3$): δ 7.34 – 7.18 (m, 5H), 7.15 – 7.09 (m, 2H), 6.52 (t, J = 8.4 Hz, 1H), 4.92 (dd, J = 8.4, 3.4 Hz, 1H), 4.39 (dt, J = 8.4, 6.2 Hz, 1H), 3.73 (s, 3H), 3.22 (dd, J = 13.8, 6.2 Hz, 1H), 3.13 (dd, J = 13.8, 6.6 Hz, 1H). ^{13}C NMR (101 MHz, $CDCl_3$): δ 172.1, 150.3 (d, $^1J_{F\text{-}C}$ = 243.7 Hz), 139.31 (d, $^2J_{F\text{-}C}$ = 11.0 Hz), 135.5, 130.1 (d, $^4J_{F\text{-}C}$ = 3.2 Hz), 129.3, 128.9, 127.6, 119.03 (d, $^4J_{F\text{-}C}$ = 2.7 Hz), 118.2 (d, $^2J_{F\text{-}C}$ = 21.6 Hz), 111.8 (d, $^3J_{F\text{-}C}$ = 3.8 Hz), 99.4 (d, $^3J_{F\text{-}C}$ = 8.7 Hz), 56.8, 52.7, 38.6. ^{19}F NMR (376 MHz, $CDCl_3$) δ -133.5 – 155.9 (m); HRMS (ESI): calcd for $C_{17}H_{15}FN_2O_4$ $[M+Na]^+$: 321.1015; found: 321.1010.

Analysis of enantiomeric purity:

The racemic sample was obtained via **Rac II** (0.16 mmol scale, 59% yield).

HPLC, Chiralpak-IB (0.46 cm ϕ, 25 cm column), 25 °C, [hexane/*i*PrOH (90:10), 1.0 mL/min]; t_R: 15.80 min (major enantiomer), 16.67 min (minor enantiomer); *ee* = >98% (e.r. = 99.7:0.3).

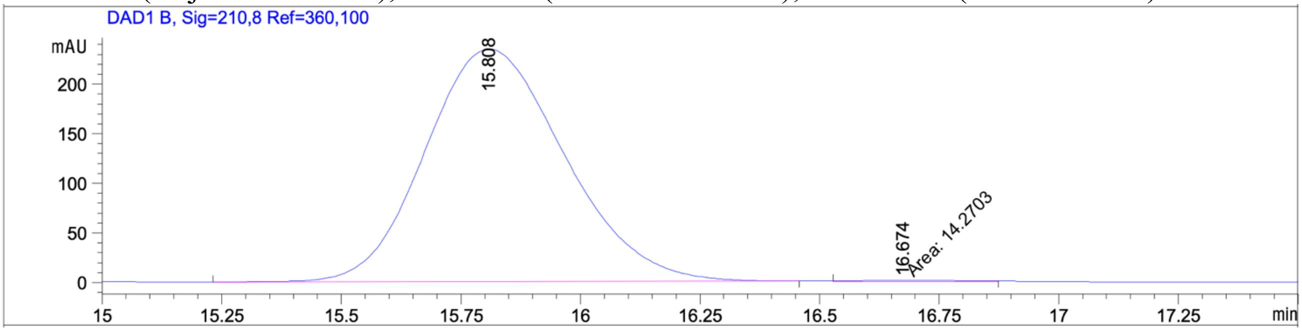

Signal 2: DAD1 B, Sig=210,8 Ref=360,100

Peak #	RetTime [min]	Type	Width [min]	Area [mAU*s]	Height [mAU]	Area %
1	15.808	BB	0.3166	4753.90674	234.44577	99.7007
2	16.674	MM	0.2546	14.27032	9.34035e-1	0.2993
Totals :				4768.17706	235.37981	

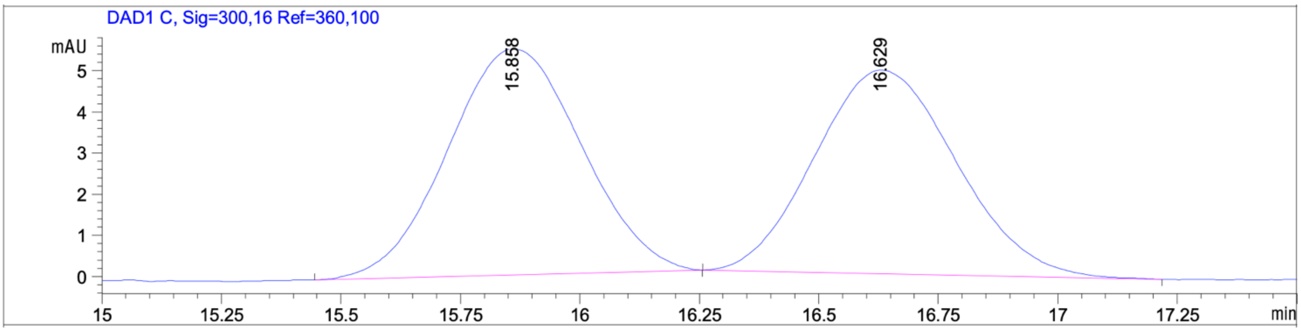

Signal 3: DAD1 C, Sig=300,16 Ref=360,100

Peak #	RetTime [min]	Type	Width [min]	Area [mAU*s]	Height [mAU]	Area %
1	15.858	BB	0.3086	106.66579	5.49155	51.1457
2	16.629	BB	0.3185	101.88694	4.94334	48.8543
Totals :				208.55273	10.43490	

N-4-(trifluoromethyl)phenyl-*L*-phenylalanine methyl ester (3d)

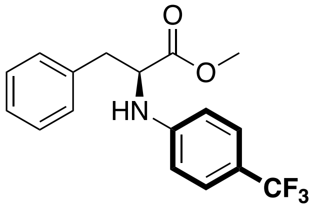

Synthesized according to **GP7** with phenylalanine methyl ester (**1a**, 0.2 mmol, 36 mg), diaryliodonium salt **2dc-OTf** (0.4 mmol, 212 mg) and Na_2CO_3 (0.2 mmol, 22 mg), 4 h reaction time. Purification provided **3d** (0.16 mmol, 50 mg, 77%) as a colorless oil.

Reaction at 130 °C gave ***3d*** *in 49% (0.10 mmol, 32 mg).*

R_f = 0.61 (*n*-pentane/EtOAc, 9:1). ^{1}H NMR (400 MHz, $CDCl_3$): δ 7.41 (d, J = 8.7 Hz, 2H), 7.35 – 7.22 (m, 3H), 7.17 – 7.11 (m, 2H), 6.60 (d, J = 8.7 Hz, 2H), 4.50 (d, J = 8.4 Hz, 1H), 4.41 (dt, J = 8.4, 6.0 Hz, 1H), 3.71 (s, 3H), 3.20 (dd, J = 13.7, 6.2 Hz, 1H), 3.12 (dd, J = 13.7, 6.0 Hz, 1H). ^{13}C NMR (101 MHz, $CDCl_3$): δ 173.0, 149.0, 135.9, 129.4, 128.8, 127.4, 126.8 (q, $^3J_{F\text{-}C}$ = 3.8 Hz), 124.9 (q, $^1J_{F\text{-}C}$ = 270.1 Hz),120.1 (q, $^2J_{F\text{-}C}$ = 32.8 Hz), 112.7, 57.2, 52.4, 38.5. ^{19}F NMR (376 MHz, $CDCl_3$) δ -61.2. The analytical data are consistent with previous reports.[55]

Analysis of enantiomeric purity:

The racemic sample was obtained via **Rac I** followed by **GP7** (0.2 mmol scale, 73% yield).

Chiral SFC, Diacel OJ-H (0.3 cm ϕ, 15 cm column), 25 °C, [10% MeOH in CO_2, 0.8 mL/min]; t_R: 2.70 min (minor enantiomer), 3.30 min (major enantiomer); *ee* = >98% (e.r. = 99.5:0.5).

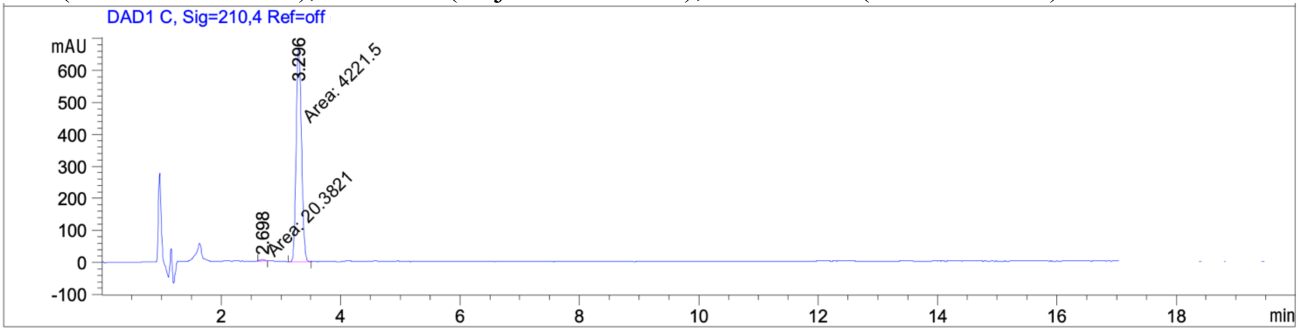

Signal 3: DAD1 C, Sig=210,4 Ref=off

Peak #	RetTime [min]	Type	Width [min]	Area [mAU*s]	Height [mAU]	Area %
1	2.698	MM	0.0946	20.38210	3.59004	0.4805
2	3.296	MM	0.1054	4221.50439	667.38483	99.5195

Totals : 4241.88650 670.97487

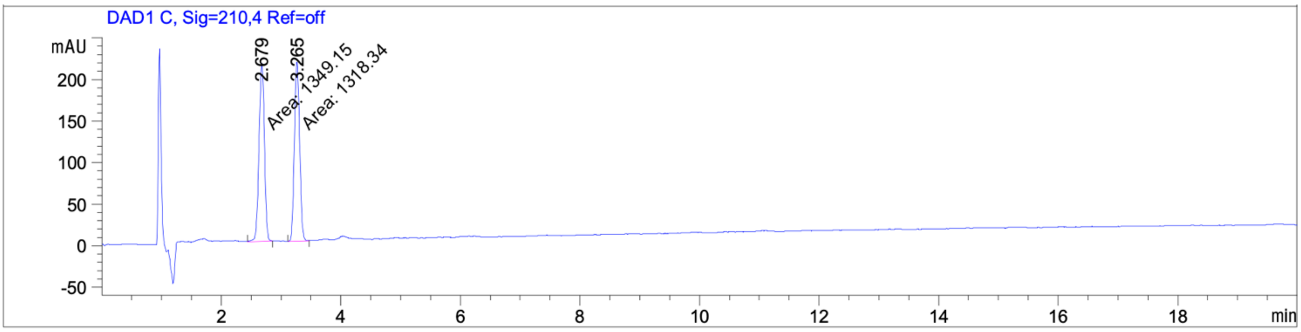

Signal 3: DAD1 C, Sig=210,4 Ref=off

Peak #	RetTime [min]	Type	Width [min]	Area [mAU*s]	Height [mAU]	Area %
1	2.679	MM	0.1047	1349.15320	214.72575	50.5775
2	3.265	MM	0.1018	1318.34351	215.78230	49.4225

Totals : 2667.49670 430.50806

_N_-3-(trifluoromethyl)phenyl-_L_-phenylalanine methyl ester (3e)

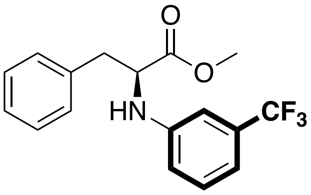

Synthesized according to **GP7** with phenylalanine methyl ester (**1a**, 0.2 mmol, 36 mg), diaryliodonium salt **3ec-OTf** (0.4 mmol, 211 mg) and Na_2CO_3 (0.2 mmol, 22 mg), 4 h reaction time. Purification gave **3e** (0.05 mmol, 23 mg, 50%) as a pale yellow oil.

R_f = 0.55 (*n*-pentane/EtOAc, 9:1). ^{1}H NMR (400 MHz, $CDCl_3$): δ 7.37 – 7.20 (m, 4H), 7.20 – 7.10 (m, 2H), 7.00 – 6.94 (m, 1H), 6.79 – 6.77 (m, 1H), 6.72 (app. dd, *J* = 8.2, 2.5 Hz, 1H), 4.43 – 4.35 (m, 2H), 3.70 (s, 3H), 3.19 (dd, *J* = 13.7, 4.9 Hz, 1H), 3.11 (dd, *J* = 13.7, 5.5 Hz, 1H). ^{13}C NMR (101 MHz, $CDCl_3$): δ 173.3, 146.7, 136.1, 131.8 (q, $^2J_{F\text{-}C}$ = 31.8 Hz), 129.9, 129.4, 128.8, 127.3, 124.3 (q, $^1J_{F\text{-}C}$ = 272.8 Hz), 116.5, 114.9 (q, $^3J_{F\text{-}C}$ = 3.9 Hz), 109.8 (q, $^3J_{F\text{-}C}$ = 4.0 Hz), 57.6, 52.4, 38.7. ^{19}F NMR (376 MHz, $CDCl_3$) δ -62.9; HRMS (ESI): calcd for $C_{17}H_{16}F_3NO_2$ $[M+Na]^+$: 346.1025; found: 346.1023.

Analysis of enantiomeric purity:

The racemic sample was obtained via **Rac I** followed by **GP7** (0.2 mmol scale, 49% yield).

Chiral SFC, Diacel OJ-H (0.3 cm ϕ, 15 cm column), 25 °C, [10% MeOH in CO_2, 0.8 mL/min]; t_R: 4.33 min (minor enantiomer), 5.29 min (major enantiomer); *ee* = 97% (e.r. = 98.6:1.4).

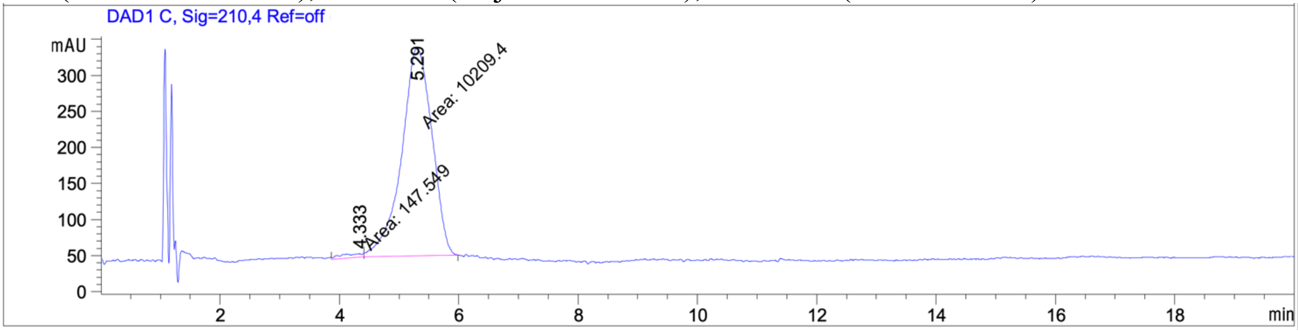

Signal 3: DAD1 C, Sig=210,4 Ref=off

Peak #	RetTime [min]	Type	Width [min]	Area [mAU*s]	Height [mAU]	Area %
1	4.333	MM	0.5072	147.54863	4.84801	1.4246
2	5.291	MM	0.5894	1.02094e4	288.67273	98.5754

Totals : 1.03570e4 293.52074

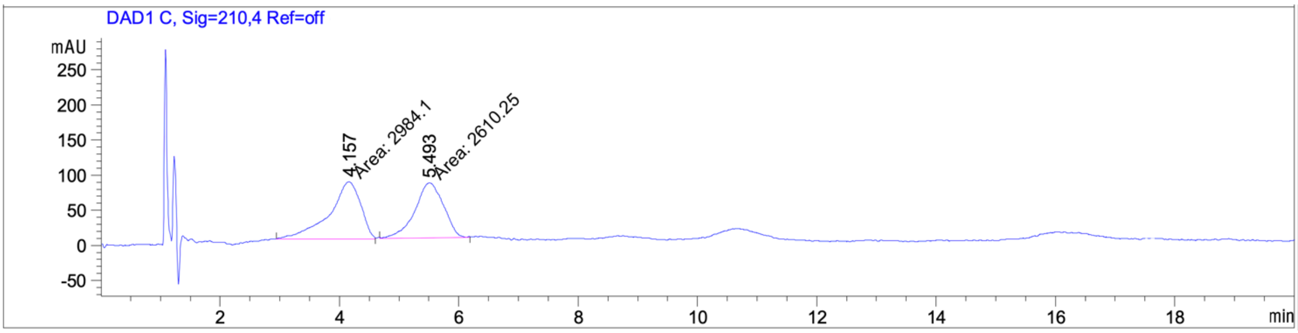

Signal 3: DAD1 C, Sig=210,4 Ref=off

Peak #	RetTime [min]	Type	Width [min]	Area [mAU*s]	Height [mAU]	Area %
1	4.157	MM	0.6124	2984.10425	81.21890	53.3413
2	5.493	MM	0.5578	2610.25439	77.99490	46.6587

Totals : 5594.35864 159.21381

N-2-chloro-5-(trifluoromethyl)phenyl-*L*-phenylalanine methyl ester (3f)

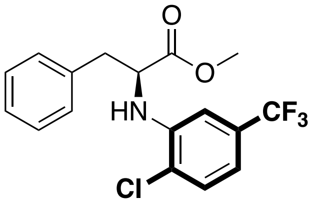

Synthesized according to **GP7** with phenylalanine methyl ester (**1a**, 0.2 mmol, 36 mg), diaryliodonium salt **3fc-OTf** (0.4 mmol, 225 mg) and Na_2CO_3 (0.2 mmol, 22 mg), 4 h reaction time. Purification provided **3f** (0.16 mmol, 58 mg, 81%) as a pale yellow oil.
R_f = 0.56 (*n*-pentane/EtOAc, 9:1). ^{1}H NMR (400 MHz, $CDCl_3$): δ 7.37 – 7.25 (m, 4H), 7.20 – 7.14 (m, 2H), 6.89 (dd, *J* = 8.2, 2.0 Hz, 1H), 6.68 (d, *J* = 2.0 Hz, 1H), 5.03 (d, *J* = 8.2 Hz, 1H), 4.39 (dt, *J* = 8.2, 6.0 Hz, 1H), 3.72 (s, 3H), 3.23 (dd, *J* = 13.6, 6.0 Hz, 1H), 3.15 (dd, *J* = 13.6, 6.6 Hz, 1H). ^{13}C NMR (101 MHz, $CDCl_3$): δ 172.6, 142.8, 135.8, 130.3 (q, $^2J_{F\text{-}C}$ = 32.5 Hz), 129.8, 129.4, 128.9, 124.0 (q, $^1J_{F\text{-}C}$ = 272.8 Hz), 125.3, 123.1 (q, $^4J_{F\text{-}C}$ = 1.5 Hz), 114.8 (q, $^3J_{F\text{-}C}$ = 3.9 Hz), 108.0 (q, $^3J_{F\text{-}C}$ = 4.0 Hz), 57.5, 52.6, 38.8. ^{19}F NMR (376 MHz, $CDCl_3$) δ -62.8; HRMS (ESI): calcd for $C_{17}H_{15}ClF_3NO_2$ $[M+Na]^+$: 380.0636; found: 380.0641.

Analysis of enantiomeric purity:

The racemic sample was obtained via **Rac II** (0.15 mmol scale, 72% yield).
HPLC, Chiralpak-IB (0.46 cm ϕ, 25 cm column), 25 °C, [hexane/*i*PrOH (80:20), 1.0 mL/min]; t_R: 4.58 min (minor enantiomer), 5.85 min (major enantiomer); *ee* = 96% (e.r. = 98.1:1.9).

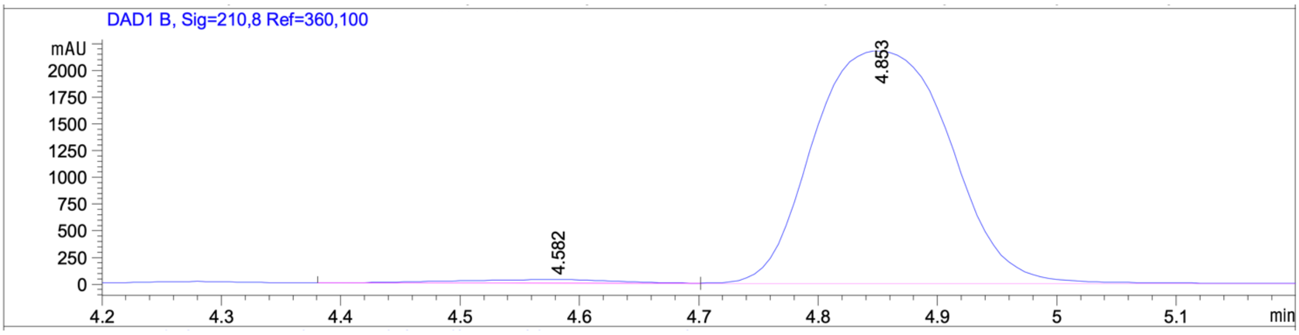

Signal 2: DAD1 B, Sig=210,8 Ref=360,100

Peak #	RetTime [min]	Type	Width [min]	Area [mAU*s]	Height [mAU]	Area %
1	4.582	VV	0.1355	326.78741	34.18490	1.8538
2	4.853	VB	0.1307	1.73012e4	2176.77100	98.1462
Totals :				1.76280e4	2210.95590	

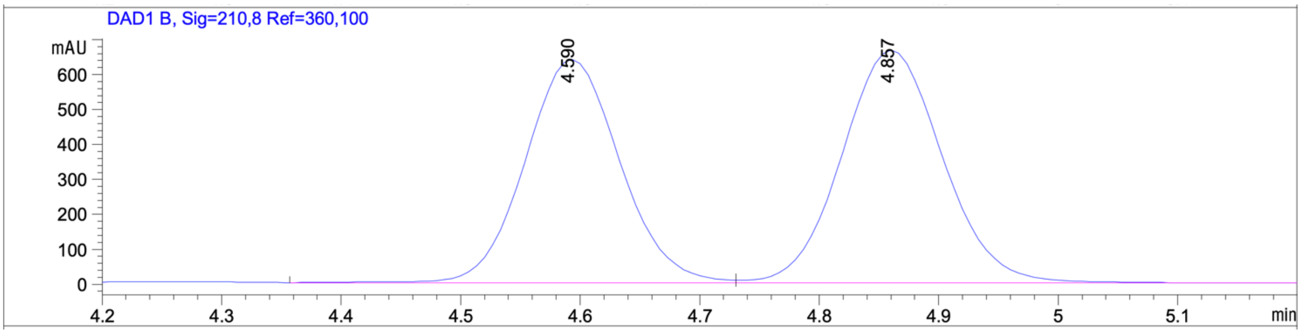

Signal 2: DAD1 B, Sig=210,8 Ref=360,100

Peak #	RetTime [min]	Type	Width [min]	Area [mAU*s]	Height [mAU]	Area %
1	4.590	BV	0.0862	3519.29541	639.99060	47.4176
2	4.857	VB	0.0903	3902.62817	666.61212	52.5824
Totals :				7421.92358	1306.60272	

N-4-bromophenyl-*L*-phenylalanine methyl ester (3g)

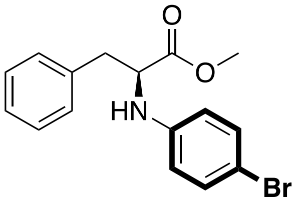

Synthesized according to **GP7** with phenylalanine methyl ester (**1a,** 0.2 mmol, 36 mg), diaryliodonium salt **2gc-OTf** (0.4 mmol, 244 mg) and Na_2CO_3 (0.2 mmol, 22 mg), 4 h reaction time. Purification gave **3g** (0.10 mmol, 35 mg, 49%) as a pale yellow oil.

R_f = 0.54 (*n*-pentane/EtOAc, 9:1). ^{1}H NMR (400 MHz, $CDCl_3$): δ 7.35 – 7.21 (m, 5H), 7.18 – 7.11 (m, 2H), 6.50 – 6.44 (m, 2H), 4.32 (dt, *J* = 8.1, 6.0 Hz, 1H), 4.18 (d, *J* = 8.1 Hz, 1H), 3.68 (s, 3H), 3.16 (dd, *J* = 13.7, 6.3 Hz, 1H), 3.09 (dd, *J* = 13.7, 6.0 Hz, 1H). ^{13}C NMR (101 MHz, $CDCl_3$): δ 173.4, 145.5, 136.2, 132.2, 129.3, 128.7, 127.3, 115.3, 110.2, 57.8, 52.3, 38.6; HRMS (ESI): calcd for $C_{16}H_{16}BrNO_2$ $[M+Na]^+$: 356.0257; found: 356.0260.

Analysis of enantiomeric purity:

The racemic sample was obtained via **Rac I** followed by **GP7** (0.2 mmol scale, 46% yield).

Chiral SFC, Diacel OJ-H (0.3 cm ϕ, 15 cm column), 25 °C, [10% MeOH in CO_2, 0.8 mL/min]; t_R: 7.80 min (minor enantiomer), 9.51 min (major enantiomer); *ee* = >98% (e.r. = 99.2:0.8).

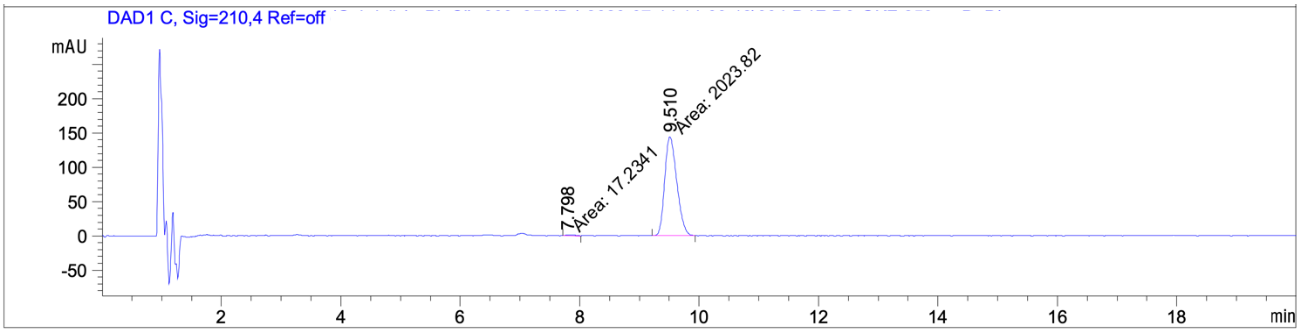

Signal 3: DAD1 C, Sig=210,4 Ref=off

Peak #	RetTime [min]	Type	Width [min]	Area [mAU*s]	Height [mAU]	Area %
1	7.798	MM	0.2159	17.23413	1.33049	0.8444
2	9.510	MM	0.2341	2023.82312	144.09145	99.1556

Totals : 2041.05725 145.42194

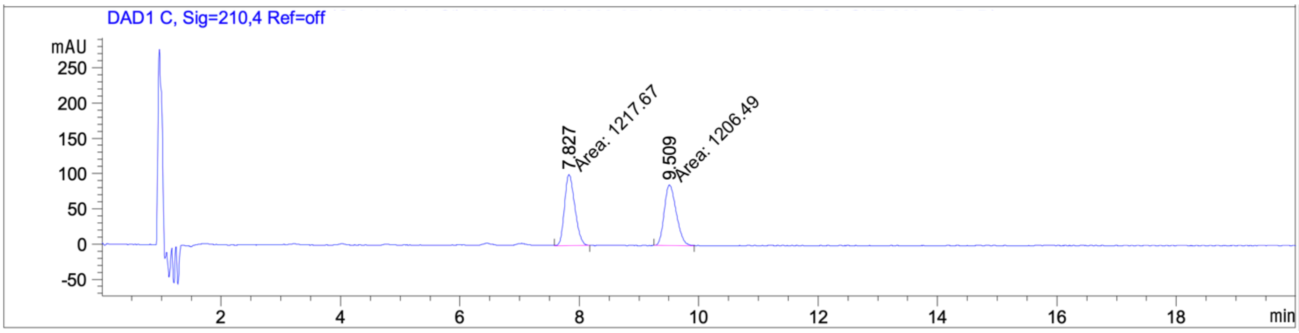

Signal 3: DAD1 C, Sig=210,4 Ref=off

Peak #	RetTime [min]	Type	Width [min]	Area [mAU*s]	Height [mAU]	Area %
1	7.827	MM	0.2016	1217.67151	100.66829	50.2306
2	9.509	MM	0.2333	1206.49084	86.18404	49.7694

Totals : 2424.16235 186.85233

_N_-4-chlorophenyl-_L_-phenylalanine methyl ester (3h)

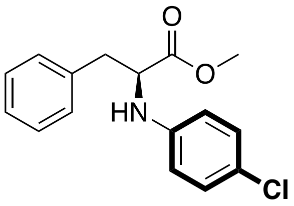

Synthesized according to **GP7** with phenylalanine methyl ester (**1a**, 0.2 mmol, 36 mg), diaryliodonium salt **2hc-OTf** (0.4 mmol, 207 mg) and Na_2CO_3 (0.2 mmol, 22 mg), 4 h reaction time. Purification gave **3h** (0.11 mmol, 32 mg, 53%) as a pale yellow oil.

*Reaction at 130 °C delivered **3h** in 20% yield (0.04 mmol, 12 mg).*

R_f = 0.54 (*n*-pentane/EtOAc, 9:1). ^{1}H NMR (400 MHz, $CDCl_3$): δ 7.35 – 7.21 (m, 3H), 7.19 – 7.08 (m, 4H), 6.54 – 6.49 (m, 2H), 4.32 (t, *J* = 6.2 Hz, 1H), 3.68 (s, 3H), 3.16 (dd, *J* = 13.7, 6.2 Hz, 1H), 3.09 (dd, *J* = 13.7, 6.3 Hz, 1H). ^{13}C NMR (101 MHz, $CDCl_3$): δ 173.4, 145.1, 136.2, 129.3, 128.7, 127.3, 123.2, 114.8, 57.9, 52.3, 38.6; HRMS (ESI): calcd for $C_{16}H_{16}ClNO_2$ $[M+Na]^+$: 312.0762; found: 312.0762.

Analysis of enantiomeric purity:

The racemic sample was obtained via **Rac I** followed by **GP7** (0.2 mmol scale, 49% yield).

Chiral SFC, Diacel OJ-H (0.3 cm ϕ, 15 cm column), 25 °C, [10% MeOH in CO_2, 0.8 mL/min]; t_R: 6.05 min (minor enantiomer), 7.00 min (major enantiomer); *ee* = 98% (e.r. = 98.9:1.1).

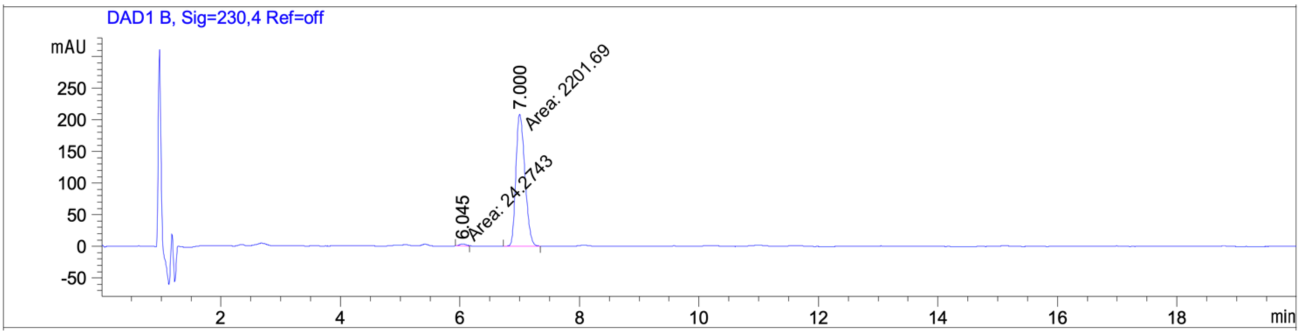

Signal 2: DAD1 B, Sig=230,4 Ref=off

Peak #	RetTime [min]	Type	Width [min]	Area [mAU*s]	Height [mAU]	Area %
1	6.045	MM	0.1378	24.27431	2.93548	1.0905
2	7.000	MM	0.1761	2201.68555	208.41048	98.9095
Totals :				2225.95985	211.34596	

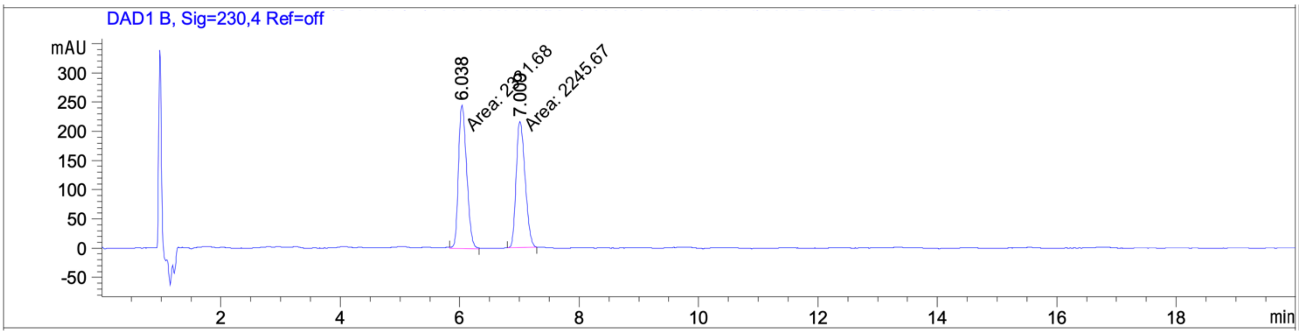

Signal 2: DAD1 B, Sig=230,4 Ref=off

Peak #	RetTime [min]	Type	Width [min]	Area [mAU*s]	Height [mAU]	Area %
1	6.038	MM	0.1588	2331.68140	244.68921	50.9395
2	7.009	MM	0.1735	2245.67383	215.75986	49.0605
Totals :				4577.35522	460.44907	

_N_-phenyl-_L_-phenylalanine methyl ester (3i)

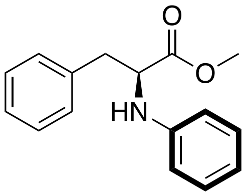

Synthesized according to **GP7** with phenylalanine methyl ester (**1a**, 0.2 mmol, 36 mg), diaryliodonium salt **2ia-OTf** (0.4 mmol, 177 mg) and Na_2CO_3 (0.2 mmol, 22 mg), 24 h reaction time. Purification provided **3i** (0.14 mmol, 25 mg, 67%) as a pale yellow oil.

R_f = 0.57 (*n*-pentane/EtOAc, 9:1). ^1H NMR (400 MHz, $CDCl_3$): δ 7.35 – 7.21 (m, 4H), 7.22 – 7.14 (m, 3H), 6.76 (tt, *J* = 7.3, 1.1 Hz, 1H), 6.66 – 6.55 (m, 2H), 4.39 (t, *J* = 6.2 Hz, 1H), 4.16 (br. s, 1H), 3.68 (s, 3H), 3.18 (dd, *J* = 13.6, 6.2 Hz, 1H), 3.11 (dd, *J* = 13.6, 6.2 Hz, 1H). ^{13}C NMR (101 MHz, $CDCl_3$): δ 173.7, 146.5, 136.5, 129.5, 129.4, 128.7, 127.2, 118.6, 113.7, 57.8, 52.2, 38.8. The analytical data are consistent with previous reports.[56]

Analysis of enantiomeric purity:

The racemic sample was obtained via **Rac I** followed by **GP7** (0.2 mmol scale, 65% yield).
Chiral SFC, Diacel OJ-H (0.3 cm ϕ, 15 cm column), 25 °C, [10% MeOH in CO_2, 0.8 mL/min]; t_R: 4.07 min (minor enantiomer), 4.83 min (major enantiomer); *ee* = 95% (e.r. = 97.5:2.5).

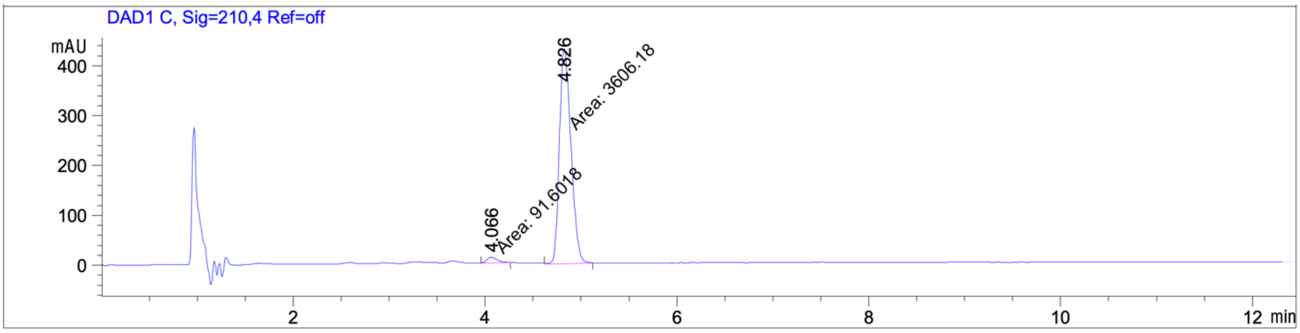

Signal 3: DAD1 C, Sig=210,4 Ref=off

Peak #	RetTime [min]	Type	Width [min]	Area [mAU*s]	Height [mAU]	Area %
1	4.066	MM	0.1304	91.60181	11.70962	2.4772
2	4.826	MM	0.1394	3606.17505	431.03342	97.5228

Totals : 3697.77686 442.74303

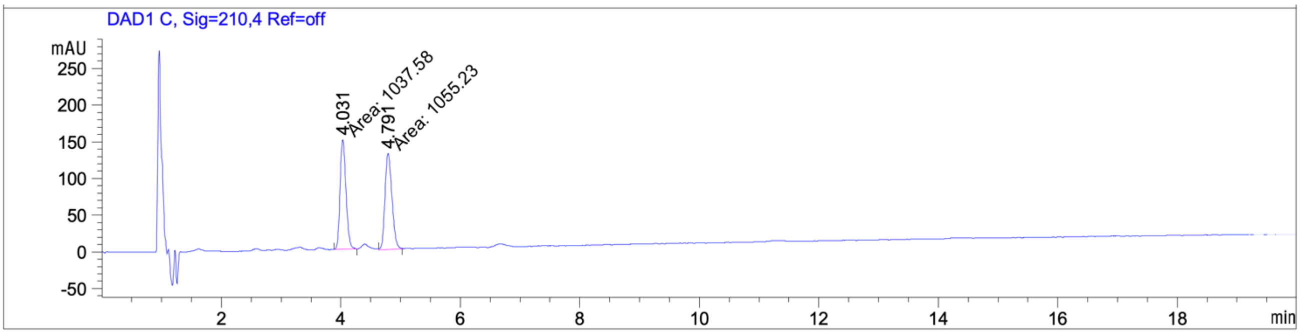

Signal 3: DAD1 C, Sig=210,4 Ref=off

Peak #	RetTime [min]	Type	Width [min]	Area [mAU*s]	Height [mAU]	Area %
1	4.031	MM	0.1157	1037.58020	149.42671	49.5782
2	4.791	MM	0.1343	1055.23462	130.92140	50.4218

Totals : 2092.81482 280.34811

_N_-4-(_tert_-butyl)phenyl-_L_-phenylalanine methyl ester (3j)

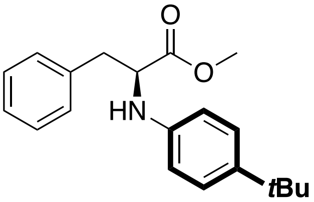

Synthesized according to **GP7** with phenylalanine methyl ester (**1a**, 0.2 mmol, 36 mg), diaryliodonium salt **2je-OTf** (0.4 mmol, 220 mg) and Na_2CO_3 (0.2 mmol, 22 mg), 24 h reaction time. Purification gave **3j** (0.10 mmol, 31 mg, 50%) as a yellow oil.

R_f = 0.59 (*n*-pentane/EtOAc, 9:1). ^{1}H NMR (400 MHz, $CDCl_3$): δ 7.35 – 7.24 (m, 3H), 7.22 – 7.16 (m, 4H), 6.61 – 6.53 (m, 2H), 4.38 – 4.31 (m, 1H), 4.08 (br. s, 1H), 3.68 (s, 3H), 3.16 (dd, *J* = 13.6, 6.4 Hz, 1H), 3.10 (dd, *J* = 13.6, 6.5 Hz, 1H), 1.28 (s, 9H). ^{13}C NMR (101 MHz, $CDCl_3$): δ 174.0, 144.1, 141.3, 136.6, 129.4, 128.7, 127.1, 126.3, 113.4, 58.2, 52.2, 39.0, 34.0, 31.6; HRMS (ESI): calcd for $C_{20}H_{25}NO_2$ $[M+Na]^+$: 334.1778; found: 334.1772.

Analysis of enantiomeric purity:

The racemic sample was obtained via **Rac I** followed by **GP7** (0.2 mmol scale, 48% yield).

Chiral SFC, Diacel OJ-H (0.3 cm ϕ, 15 cm column), 25 °C, [10% MeOH in CO_2, 0.8 mL/min]; t_R: 3.54 min (minor enantiomer), 4.64 min (major enantiomer); *ee* = 96% (e.r. = 97.9:2.1).

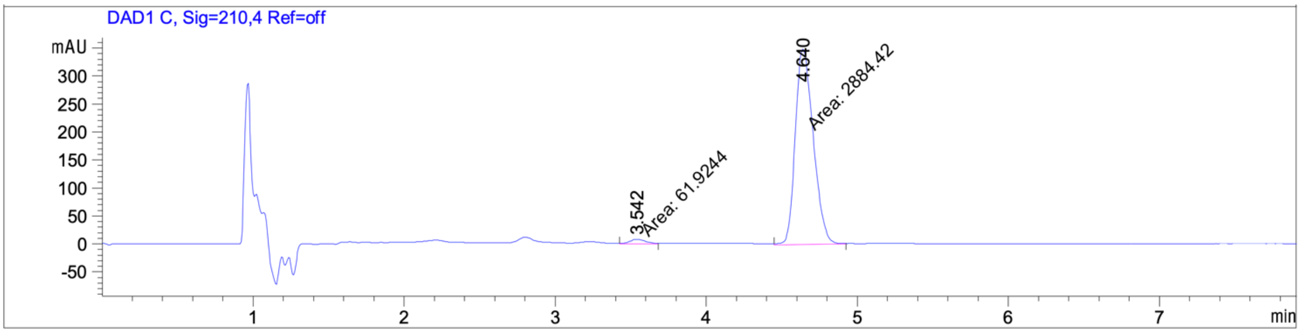

Signal 3: DAD1 C, Sig=210,4 Ref=off

Peak #	RetTime [min]	Type	Width [min]	Area [mAU*s]	Height [mAU]	Area %
1	3.542	MM	0.1244	61.92440	8.29681	2.1017
2	4.640	MM	0.1377	2884.41821	349.14059	97.8983
Totals :				2946.34261	357.43740	

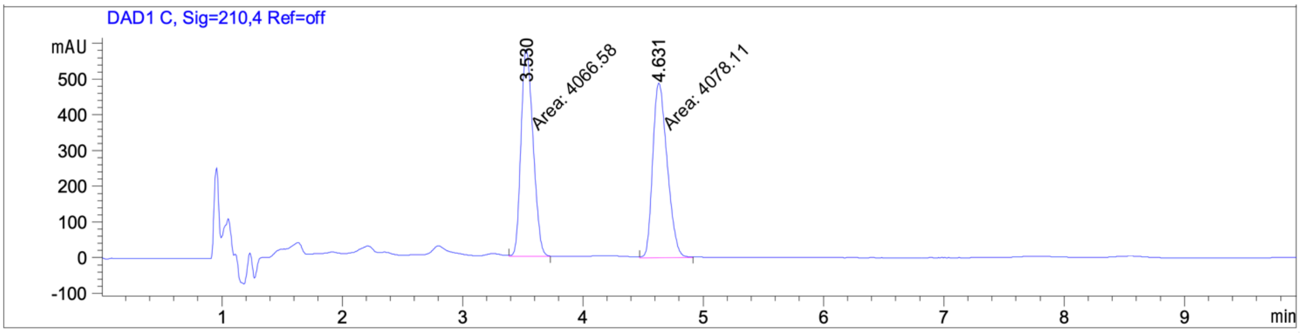

Signal 3: DAD1 C, Sig=210,4 Ref=off

Peak #	RetTime [min]	Type	Width [min]	Area [mAU*s]	Height [mAU]	Area %
1	3.530	MM	0.1166	4066.57935	581.31549	49.9292
2	4.631	MM	0.1389	4078.11108	489.38931	50.0708
Totals :				8144.69043	1070.70480	

***N*-mesityl-*L*-phenylalanine methyl ester (3k)**

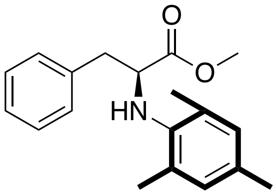

Synthesized according to **GP7** with phenylalanine methyl ester (**1a**, 0.2 mmol, 36 mg), diaryliodonium salt **2kc-OTf** (0.4 mmol, 216 mg) and Na_2CO_3 (0.2 mmol, 22 mg), 24 h reaction time. Purification gave **3k** (0.08 mmol, 24 mg, 40%) as a yellow oil.

R_f = 0.63 (*n*-pentane/EtOAc, 9:1). ^{1}H NMR (400 MHz, $CDCl_3$): δ 7.23 – 7.09 (m, 3H), 7.09 – 7.05 (m, 2H), 6.67 (s, 2H), 3.97 (dd, *J* = 7.8, 6.0 Hz, 1H), 3.58 (s, 1H), 3.44 (s, 3H), 3.00 (dd, *J* = 13.3, 5.0 Hz, 1H), 2.92 (dd, *J* = 13.3, 7.8 Hz, 1H), 2.10 (s, 9H). ^{13}C NMR (101 MHz, $CDCl_3$): δ 175.0, 141.3, 137.1, 131.3, 129.8, 129.4, 129.0, 128.5, 126.9, 61.7, 51.8, 40.5, 20.6, 18.7. The analytical data are consistent with previous reports.[57]

Analysis of enantiomeric purity:

The racemic sample was obtained via **Rac I** followed by **GP7** with symmetric salt **2kb-OTf** (0.2 mmol scale, 20% yield).

HPLC, Chiralpak-ID (0.46 cm ϕ, 25 cm column), 25 °C, [hexane/*i*PrOH (95:5), 1.0 mL/min]; t_R: 4.61 min (major enantiomer), 4.89 min (minor enantiomer); *ee* = >98% (e.r. = 99.2:0.8).

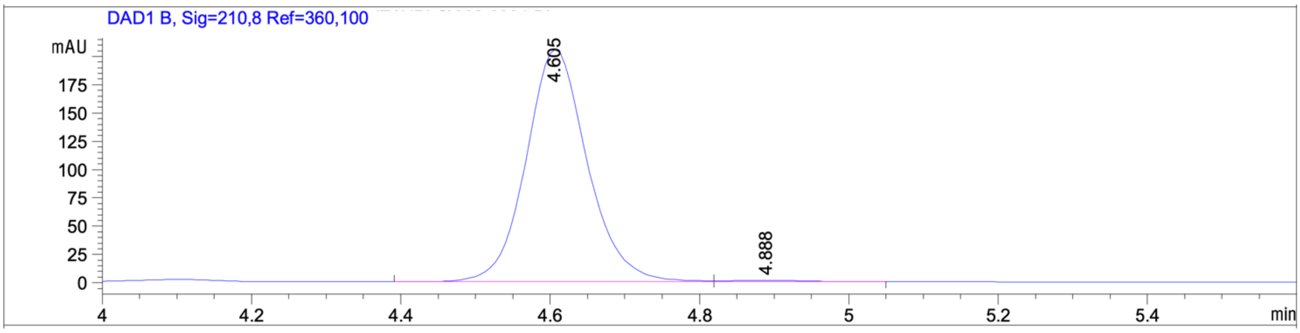

Signal 2: DAD1 B, Sig=210,8 Ref=360,100

Peak #	RetTime [min]	Type	Width [min]	Area [mAU*s]	Height [mAU]	Area %
1	4.605	BV	0.0883	1168.09119	205.43137	99.2195
2	4.888	VB	0.0999	9.18857	1.37553	0.7805

Totals : 1177.27975 206.80689

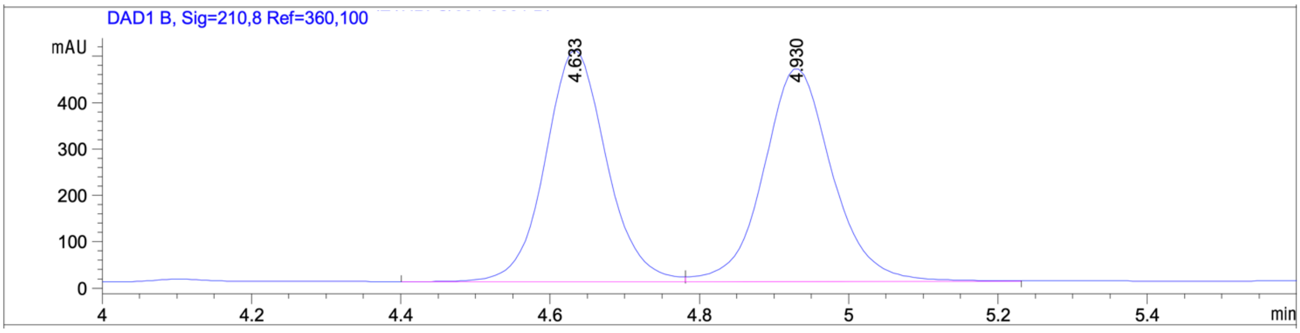

Signal 2: DAD1 B, Sig=210,8 Ref=360,100

Peak #	RetTime [min]	Type	Width [min]	Area [mAU*s]	Height [mAU]	Area %
1	4.633	BV	0.0883	2844.66870	500.69455	49.1581
2	4.930	VV	0.0986	2942.10962	460.08911	50.8419

Totals : 5786.77832 960.78366

***N*-4-methoxy-3-(methoxycarbonyl)phenyl-*L*-phenylalanine methyl ester (3l)**

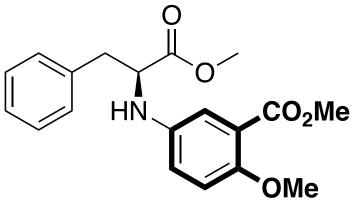

Synthesized according to **GP7** with phenylalanine methyl ester (**1a**, 0.2 mmol, 36 mg), diaryliodonium salt **2le-OTf** (0.4 mmol, 247 mg) and Na_2CO_3 (0.2 mmol, 22 mg), 24 h reaction time. Purification provided **3l** (0.10 mmol, 35 mg, 50%) as a colorless oil.

R_f = 0.29 (*n*-pentane/EtOAc, 4:1). ^{1}H NMR (400 MHz, $CDCl_3$): δ 7.34 – 7.20 (m, 3H), 7.20 – 7.12 (m, 2H), 7.07 (d, *J* = 3.0 Hz, 1H), 6.83 (d, *J* = 8.9 Hz, 1H), 6.72 (dd, *J* = 8.9, 3.0 Hz, 1H), 4.31 (t, *J* = 6.2 Hz, 1H), 3.87 (s, 3H), 3.81 (s, 3H), 3.66 (s, 3H), 3.17 – 3.05 (m, 2H). ^{13}C NMR (101 MHz, $CDCl_3$): δ 173.7, 166.9, 152.5, 140.1, 136.4, 129.4, 128.7, 127.2, 120.8, 119.3, 117.1, 114.3, 58.8, 56.9, 52.2, 52.2, 38.9; HRMS (ESI): calcd for $C_{19}H_{21}NO_5$ $[M+Na]^+$: 366.1312; found: 366.1314.

Analysis of enantiomeric purity:

The racemic sample was obtained via **Rac I** followed by **GP7** (0.2 mmol scale, 37% yield).

Chiral SFC, Diacel IA (0.3 cm ϕ, 15 cm column), 25 °C, [5% MeOH in CO_2, 0.8 mL/min]; t_R: 8.43 min (minor enantiomer), 11.25 min (major enantiomer); *ee* = 90% (e.r. = 95.4:4.6).

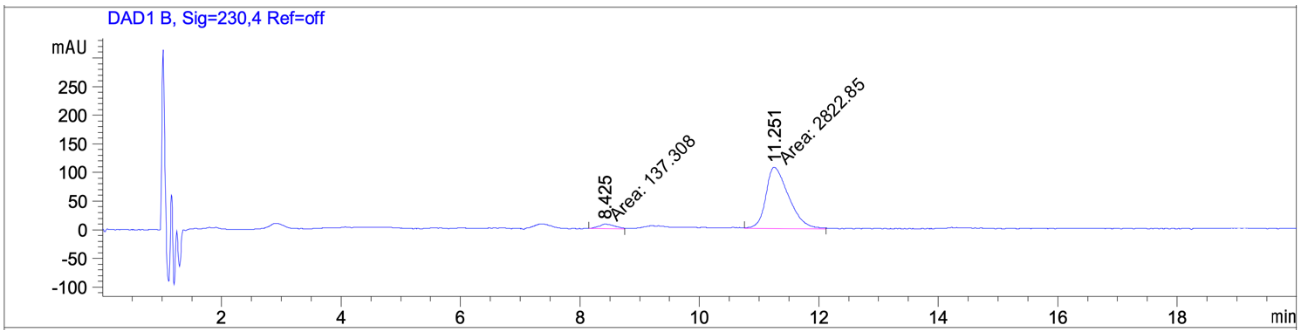

Signal 2: DAD1 B, Sig=230,4 Ref=off

Peak #	RetTime [min]	Type	Width [min]	Area [mAU*s]	Height [mAU]	Area %
1	8.425	MM	0.3033	137.30751	7.54459	4.6385
2	11.251	MM	0.4412	2822.85205	106.62596	95.3615
Totals :				2960.15956	114.17055	

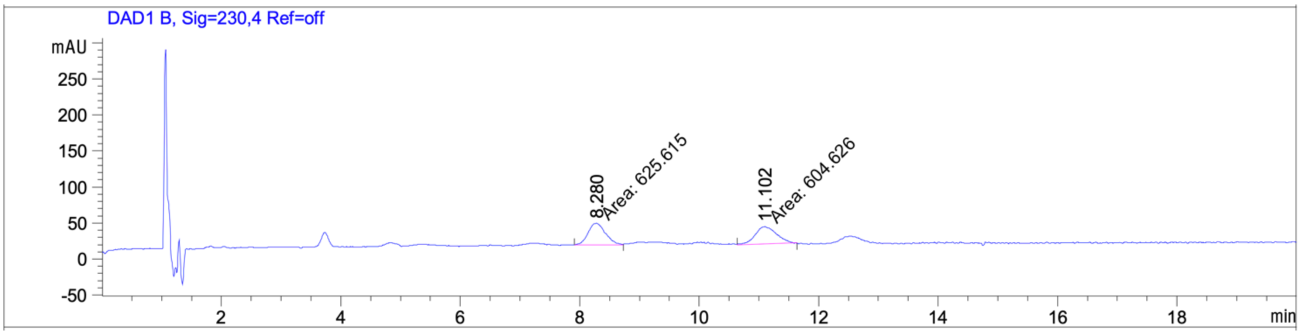

Signal 2: DAD1 B, Sig=230,4 Ref=off

Peak #	RetTime [min]	Type	Width [min]	Area [mAU*s]	Height [mAU]	Area %
1	8.280	MM	0.3447	625.61548	30.25180	50.8531
2	11.102	MM	0.4175	604.62555	24.13464	49.1469
Totals :				1230.24103	54.38644	

_N_-4-chloro-pyridin-3-yl-_L_-phenylalanine methyl ester (3m)

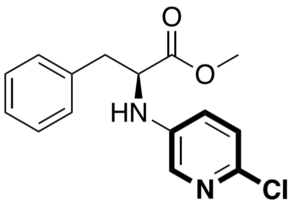

Synthesized according to **GP7** with phenylalanine methyl ester (**1a**, 0.2 mmol, 36 mg), diaryliodonium salt **2mc-OTf** (0.4 mmol, 202 mg) and Na_2CO_3 (0.2 mmol, 22 mg), 24 h reaction time. Purification provided **3m** (0.035 mmol, 10 mg, 17%) as a yellow oil.
R_f = 0.11 (*n*-pentane/EtOAc, 9:1). ^{1}H NMR (400 MHz, $CDCl_3$): δ 7.75 (d, *J* = 3.1 Hz, 1H), 7.36 – 7.24 (m, 3H), 7.14 (dd, *J* = 6.4, 1.8 Hz, 2H), 7.07 (d, *J* = 8.6 Hz, 1H), 6.82 (dd, *J* = 8.6, 3.1 Hz, 1H), 4.30 (br. s, 1H), 4.23 (br. s, 1H), 3.71 (s, 3H), 3.18 (dd, *J* = 13.7, 5.6 Hz, 1H), 3.10 (dd, *J* = 13.7, 6.3 Hz, 1H). ^{13}C NMR (101 MHz, $CDCl_3$): δ 172.9, 141.8, 140.3, 135.8, 135.3, 129.3, 128.9, 127.5, 124.3, 123.3, 57.6, 52.5, 38.6; HRMS (ESI): calcd for $C_{15}H_{15}ClN_2O_2$ $[M+Na]^+$: 313.0711; found: 313.0714.

Analysis of enantiomeric purity:

The racemic sample was obtained via **Rac I** followed by **GP7** at 110 °C (0.2 mmol scale, 9% yield). Chiral SFC, Diacel OJ-H (0.3 cm ϕ, 15 cm column), 25 °C, [10% MeOH in CO_2, 0.8 mL/min]; t_R: 5.66 min (minor enantiomer), 6.20 min (major enantiomer); *ee* = 91% (e.r. = 95.7:4.3).

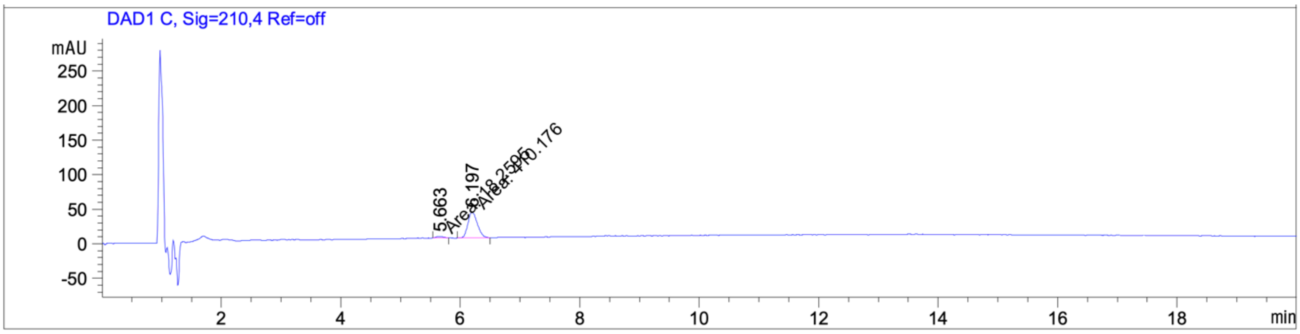

Signal 3: DAD1 C, Sig=210,4 Ref=off

Peak #	RetTime [min]	Type	Width [min]	Area [mAU*s]	Height [mAU]	Area %
1	5.663	MM	0.1431	18.25948	2.12703	4.2619
2	6.197	MM	0.1841	410.17560	37.13997	95.7381

Totals : 428.43508 39.26700

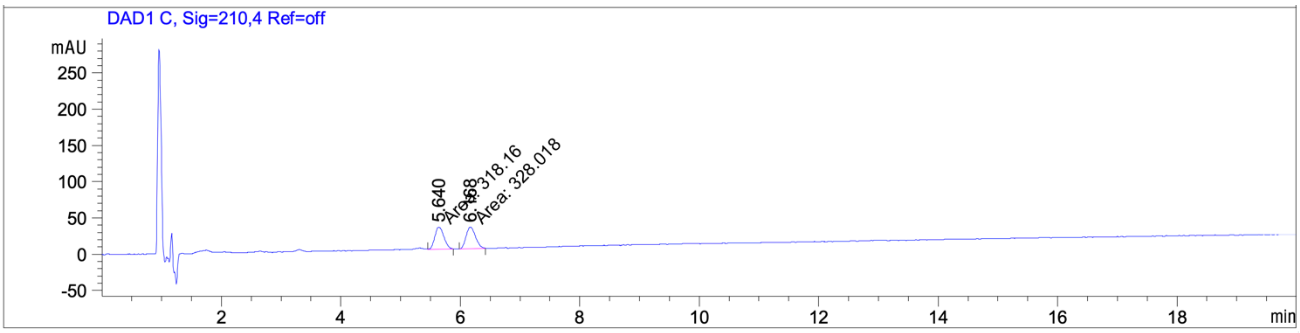

Signal 3: DAD1 C, Sig=210,4 Ref=off

Peak #	RetTime [min]	Type	Width [min]	Area [mAU*s]	Height [mAU]	Area %
1	5.640	MM	0.1753	318.15967	30.25718	49.2372
2	6.168	MM	0.1832	328.01797	29.83612	50.7628

Totals : 646.17764 60.09330

_N_-2-(2-iodobenzyl)phenyl-_L_-phenylalanine methyl ester (3n)

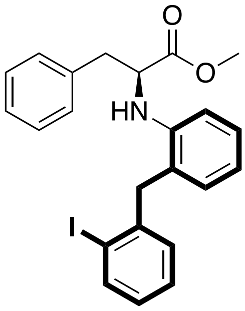

Synthesized according to **GP7** with phenylalanine methyl ester (**1a**, 0.2 mmol, 36 mg), diaryliodonium salt **2n-OTf** (0.4 mmol, 179 mg) and Na_2CO_3 (0.2 mmol, 22 mg), 24 h reaction time. Purification provided **3n** (0.12 mmol, 57 mg, 59%) as a pale yellow oil.

R_f = 0.47 (*n*-pentane/EtOAc, 20:1). ^{1}H NMR (400 MHz, $CDCl_3$): δ 7.88 (dd, *J* = 7.9, 1.2 Hz, 1H), 7.25 – 7.11 (m, 5H), 7.02 (dd, *J* = 7.7, 1.8 Hz, 2H), 6.95 – 6.89 (m, 2H), 6.87 (dd, *J* = 7.7, 1.7 Hz, 1H), 6.73 (td, *J* = 7.4, 1.2 Hz, 1H), 6.57 (app. d, *J* = 8.2 Hz, 1H), 4.34 (q, *J* = 6.6 Hz, 1H), 3.91 – 3.88 (m, 1H), 3.86 (s, 2H), 3.64 (s, 3H), 3.09 (dd, *J* = 13.6, 6.0 Hz, 1H), 3.02 (dd, *J* = 13.6, 6.6 Hz, 1H). ^{13}C NMR (101 MHz, $CDCl_3$): δ 173.7, 144.5, 141.5, 139.5, 136.3, 130.9, 129.6, 129.2, 128.7, 128.6, 128.4, 128.1, 127.1, 124.4, 118.4, 111.2, 101.7, 57.6, 52.2, 43.2, 38.9; HRMS (ESI): calcd for $C_{23}H_{22}INO_2$ $[M+Na]^+$: 494.0587; found: 494.0588.

Analysis of enantiomeric purity:

The racemic sample was obtained via **Rac I** followed by **GP7** (0.2 mmol scale, 54% yield).

Chiral SFC, Diacel OJ-H (0.3 cm ϕ, 15 cm column), 25 °C, [10% MeOH in CO_2, 0.8 mL/min]; t_R: 9.50 min (minor enantiomer), 10.65 min (major enantiomer); *ee* = 76% (e.r. = 88.1:11.9).

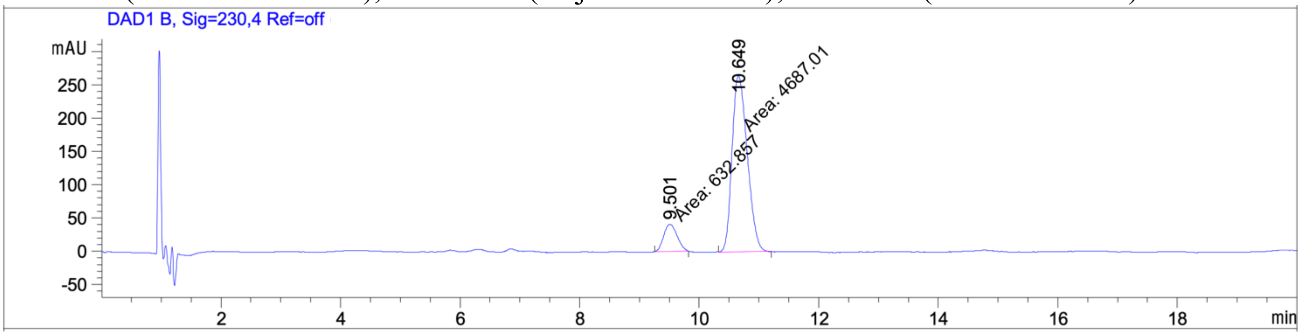

Signal 2: DAD1 B, Sig=230,4 Ref=off

Peak #	RetTime [min]	Type	Width [min]	Area [mAU*s]	Height [mAU]	Area %
1	9.501	MM	0.2593	632.85699	40.67851	11.8961
2	10.649	MM	0.2953	4687.01172	264.48920	88.1039
Totals :				5319.86871	305.16771	

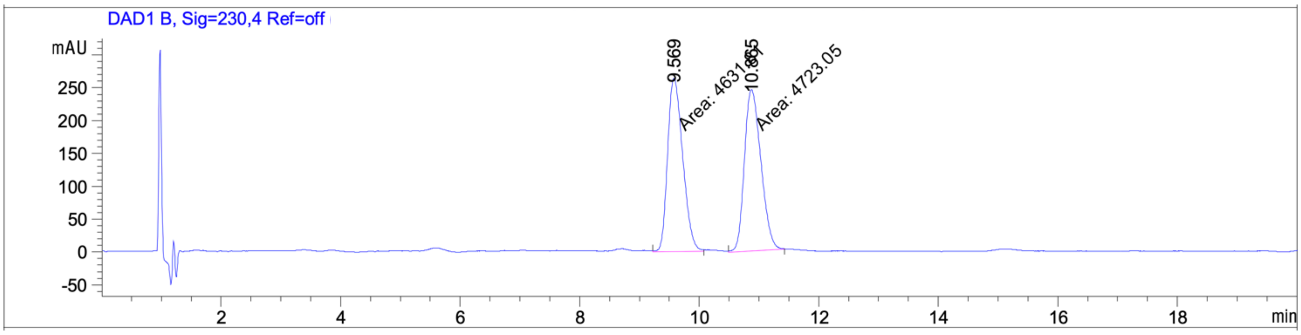

Signal 2: DAD1 B, Sig=230,4 Ref=off

Peak #	RetTime [min]	Type	Width [min]	Area [mAU*s]	Height [mAU]	Area %
1	9.569	MM	0.2924	4631.80859	264.03876	49.5123
2	10.865	MM	0.3198	4723.04932	246.12773	50.4877
Totals :				9354.85791	510.16649	

***N*-4-nitrophenyl-*L*-phenylalanine benzyl ester (3o)**

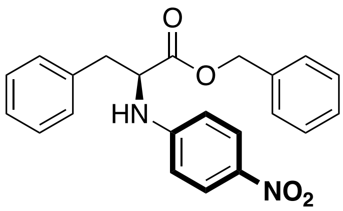

Synthesized according to **GP7** with phenylalanine benzyl ester (**1b**, 0.2 mmol, 51 mg), diaryliodonium salt **2ac-OTf** (0.4 mmol, 203 mg) and Na_2CO_3 (0.2 mmol, 22 mg), 4 h reaction time. Purification provided **3o** (0.15 mmol, 56 mg, 75%) as a bright yellow oil.

*Reaction at 130 °C (0.4 mmol scale) gave **3o** in 78% (0.32 mmol, 119 mg).*

R_f = 0.24 (*n*-pentane/EtOAc, 9:1). ^{1}H NMR (400 MHz, $CDCl_3$): δ 8.06 – 7.97 (m, 2H), 7.37 – 7.31 (m, 3H), 7.28 – 7.20 (m, 5H), 7.07 – 7.00 (m, 2H), 6.56 – 6.41 (m, 2H), 5.14 (s, 2H), 4.98 (d, *J* = 8.2 Hz, 1H), 4.47 (dt, *J* = 8.2, 6.0 Hz, 1H), 3.20 (dd, *J* = 13.8, 6.0 Hz, 1H), 3.13 (dd, *J* = 13.8, 6.0 Hz, 1H). ^{13}C NMR (101 MHz, $CDCl_3$): δ 171.3, 151.6, 139.0, 135.3, 134.9, 129.4, 128.8, 128.8, 128.8, 127.5, 126.4, 112.0, 67.6, 56.9, 38.2; HRMS (ESI): calcd for $C_{22}H_{20}N_2O_4$ $[M+Na]^+$: 399.1315; found: 399.1314.

Analysis of enantiomeric purity:

The racemic sample was obtained via **Rac II** (0.10 mmol scale, 61% yield).
Chiral SFC, Diacel OJ-H (0.3 cm ϕ, 15 cm column), 25 °C, [10% MeOH in CO_2, 0.8 mL/min]; t_R: 4.84 min (major enantiomer), 6.11 min (minor enantiomer); *ee* = >98% (e.r. = 100:0).

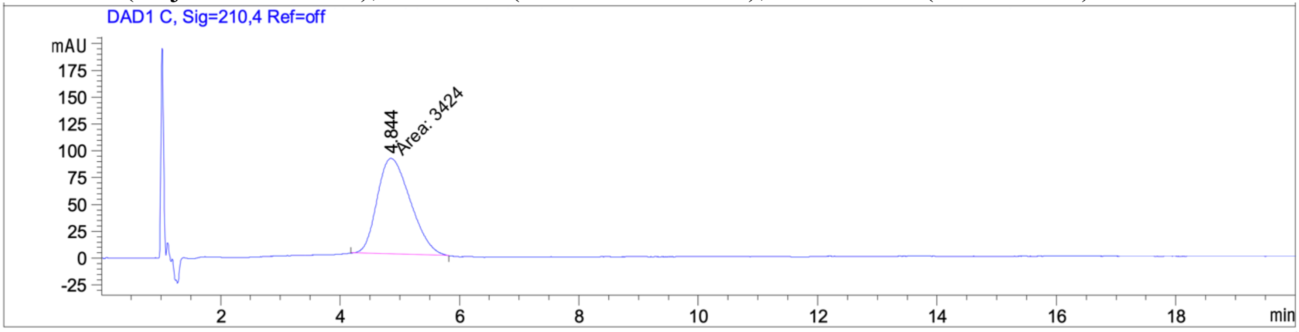

Signal 3: DAD1 C, Sig=210,4 Ref=off

Peak #	RetTime [min]	Type	Width [min]	Area [mAU*s]	Height [mAU]	Area %
1	4.844	MM	0.6424	3424.00244	88.83248	100.0000
Totals :				3424.00244	88.83248	

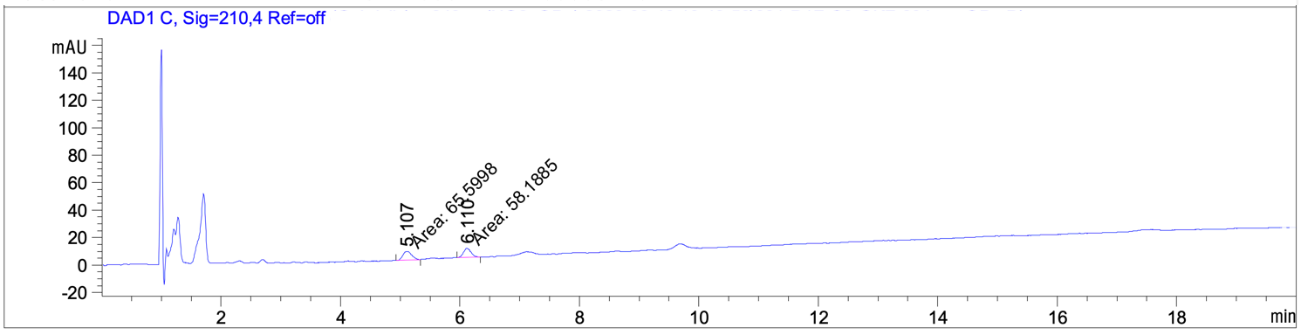

Signal 3: DAD1 C, Sig=210,4 Ref=off

Peak #	RetTime [min]	Type	Width [min]	Area [mAU*s]	Height [mAU]	Area %
1	5.107	MM	0.1736	65.59984	6.29912	52.9935
2	6.110	MM	0.1482	58.18855	6.54574	47.0065
Totals :				123.78838	12.84486	

N-4-cyanophenyl-*L*-phenylalanine benzyl ester (3p)

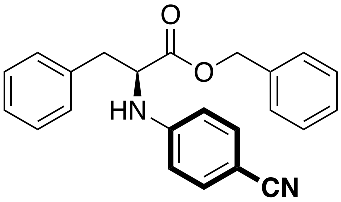

Synthesized according to **GP7** with phenylalanine benzyl ester (**1b**, 0.2 mmol, 51 mg), diaryliodonium salt **2bc-OTf** (0.4 mmol, 204 mg) and Na_2CO_3 (0.2 mmol, 22 mg), 4 h reaction time. Purification provided **3p** (0.16 mmol, 57 mg, 80%) as a yellow oil.
Reaction at 130 °C gave ***3p*** *in 84% (0.17 mmol, 60 mg).*
R_f = 0.59 (*n*-pentane/EtOAc, 9:1). ^{1}H NMR (400 MHz, $CDCl_3$): δ 7.40 (d, *J* = 8.7 Hz, 2H), 7.38 – 7.34 (m, 3H), 7.26 – 7.22 (m, 5H), 7.08 – 7.04 (m, 2H), 6.54 (d, *J* = 8.7 Hz, 2H), 5.14 (s, 2H), 4.71 (d, *J* = 8.3 Hz, 1H), 4.44 (dt, *J* = 8.3, 6.0 Hz, 1H), 3.19 (dd, *J* = 13.7, 6.3 Hz, 1H), 3.12 (dd, *J* = 13.7, 6.0 Hz, 1H). ^{13}C NMR (101 MHz, $CDCl_3$): δ 172.0, 149.6, 135.4, 135.0, 133.9, 129.4, 128.8, 128.8, 128.6, 127.4, 120.2, 113.1, 100.2, 67.5, 56.8, 38.2; HRMS (ESI): calcd for $C_{23}H_{20}N_2O_2$ $[M+Na]^+$: 379.1417; found: 379.1424.

***N*-4-nitrophenyl-*L*-alanine benzyl ester (3q)**

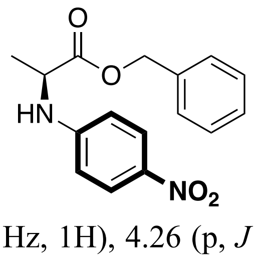

Synthesized according to **GP7** with alanine benzyl ester (**1c**, 0.2 mmol, 36 mg), diaryliodonium salt **2ac-OTf** (0.4 mmol, 200 mg) and Na_2CO_3 (0.2 mmol, 22 mg), 4 h reaction time. Purification provided **3q** (0.09 mmol, 26 mg, 42%) as a bright yellow oil.

R_f = 0.20 (*n*-pentane/EtOAc, 9:1). ^{1}H NMR (400 MHz, $CDCl_3$): δ 8.11 – 8.01 (m, 2H), 7.40 – 7.28 (m, 5H), 6.54 – 6.50 (m, 2H), 5.20 (s, 2H), 5.03 (d, *J* = 7.8 Hz, 1H), 4.26 (p, *J* = 7.0 Hz, 1H), 1.54 (d, *J* = 7.0 Hz, 3H). ^{13}C NMR (101 MHz, $CDCl_3$): δ 173.1, 151.7, 139.0, 135.2, 128.8, 128.8, 128.5, 126.5, 111.8, 67.6, 51.5, 18.6.; HRMS (ESI): calcd for $C_{16}H_{16}N_2O_4$ $[M+Na]^+$: 323.1002; found: 323.0999.

***N*-4-nitrophenyl-*L*-valine benzyl ester (3r)**

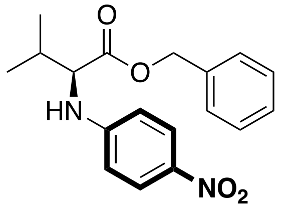

Synthesized according to **GP7** with valine benzyl ester (**1d**, 0.2 mmol, 41 mg), diaryliodonium salt **2ac-OTf** (0.4 mmol, 204 mg) and Na_2CO_3 (0.2 mmol, 22 mg), 4 h reaction time. Purification gave **3r** (0.14 mmol, 46 mg, 70%) as a bright yellow oil.

R_f = 0.33 (*n*-pentane/EtOAc, 9:1). ^{1}H NMR (400 MHz, $CDCl_3$): δ 8.07 – 8.01 (m, 2H), 7.38 – 7.28 (m, 5H), 6.58 – 6.52 (m, 2H), 5.19 (s, 2H), 5.00 (d, *J* = 8.8 Hz, 1H), 4.01 (dd, *J* = 8.8, 5.7 Hz, 1H), 2.26 – 2.15 (m, 1H), 1.03 (d, *J* = 6.9 Hz, 3H), 0.99 (d, *J* = 6.9 Hz, 3H). ^{13}C NMR (101 MHz, $CDCl_3$): δ 172.1, 152.5, 138.8, 135.2, 128.8, 128.7, 126.4, 111.9, 67.4, 61.7, 31.6, 19.0, 18.6; HRMS (ESI): calcd for $C_{18}H_{20}N_2O_4$ $[M+Na]^+$: 351.1315; found: 351.1316.

***N*-phenyl-*L*-alanine benzyl ester (3s)**

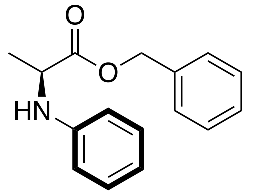

Synthesized according to **GP7** with alanine benzyl ester (**1c**, 0.2 mmol, 36 mg), diaryliodonium salt **2ia-OTf** (0.4 mmol, 177 mg) and Na_2CO_3 (0.2 mmol, 22 mg), 24 h reaction time. Purification provided **3s** (0.09 mmol, 24 mg, 46%) as a pale yellow oil.

R_f = 0.57 (*n*-pentane/EtOAc, 9:1). ^{1}H NMR (400 MHz, $CDCl_3$): δ 7.39 – 7.32 (m, 3H), 7.231 – 7.27 (m, 2H), 7.18 (dd, *J* = 8.6, 7.3 Hz, 2H), 6.80 – 6.71 (m, 1H), 6.62 (dd, *J* = 8.7, 1.1 Hz, 2H), 5.17 (s, 2H), 4.21 (q, *J* = 7.0 Hz, 1H), 1.50 (d, *J* = 6.9 Hz, 3H). ^{13}C NMR (101 MHz, $CDCl_3$): 174.6, 146.7, 135.7, 129.5, 128.7, 128.5, 128.3, 118.5, 113.6, 67.0, 52.2, 19.0. The analytical data are consistent with previous reports.[58]

***N*-4-(*tert*-butyl)phenyl-*L*-alanine benzyl ester (3t)**

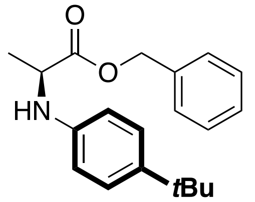

Synthesized according to **GP7** with alanine benzyl ester (**1c**, 0.2 mmol, 36 mg), diaryliodonium salt **2je-OTf** (0.4 mmol, 220 mg) and Na_2CO_3 (0.2 mmol, 22 mg), 24 h reaction time. Purification provided **3t** (0.08 mmol, 26 mg, 41%) as a yellow oil.

R_f = 0.53 (*n*-pentane/EtOAc, 9:1). ^{1}H NMR (400 MHz, $CDCl_3$): δ 7.38 – 7.32 (m, 3H), 7.30 – 7.27 (m, 2H), 7.23 – 7.16 (m, 2H), 6.60 – 6.53 (m, 2H), 5.17 (s, 2H), 4.19 (q, *J* = 7.0 Hz, 1H), 1.49 (d, *J* = 7.0 Hz, 3H), 1.29 (s, 9H). ^{13}C NMR (101 MHz, $CDCl_3$): δ 174.8, 144.3, 141.3, 135.8, 128.7, 128.4, 128.3, 126.2, 113.3, 66.9, 52.5, 34.0, 31.7, 19.1; HRMS (ESI): calcd for $C_{20}H_{25}NO_2$ $[M+Na]^+$: 311.4170; found: 311.4173.

***N*-phenyl-*L*-valine benzyl ester (3u)**

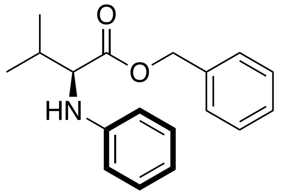

Synthesized according to **GP7** with valine benzyl ester (**1d**, 0.2 mmol, 41 mg), diaryliodonium salt **2ia-OTf** (0.4 mmol, 177 mg) and Na_2CO_3 (0.2 mmol, 22 mg), 24 h reaction time. Purification provided **3u** (0.15 mmol, 42 mg, 74%) as a pale yellow oil.

R_f = 0.57 (*n*-pentane/EtOAc, 9:1). ^{1}H NMR (400 MHz, $CDCl_3$): δ 7.40 – 7.27 (m, 5H), 7.23 – 7.13 (m, 2H), 6.76 (tt, *J* = 7.3, 1.1 Hz, 1H), 6.66 (dd, *J* = 8.7, 1.1 Hz, 2H), 5.17 (d, *J* = 12.3 Hz, 1H), 5.14 (d, *J* = 12.3 Hz, 1H), 4.15 (br. s, 1H), 3.93 (d, *J* = 5.9 Hz, 1H), 2.15 (dq, *J* = 6.8, 5.9 Hz, 1H), 1.05 (d, *J* = 6.8 Hz, 3H), 1.02 (d, *J* = 6.8 Hz, 3H). ^{13}C NMR (101 MHz, $CDCl_3$): δ 173.7, 147.5, 135.7, 129.4, 128.7, 128.5, 128.5, 118.4, 113.8, 66.8, 62.7, 31.7, 19.3, 18.8. The analytical data are consistent with previous reports.[56]

N-4-(*tert*-butyl)phenyl-*L*-valine benzyl ester (3v)

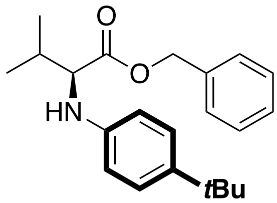

Synthesized according to **GP7** with valine benzyl ester (**1d**, 0.2 mmol, 41 mg), diaryliodonium salt **2je-OTf** (0.4 mmol, 220 mg) and Na_2CO_3 (0.2 mmol, 22 mg), 24 h reaction time. Purification provided **3v** (0.15 mmol, 51 mg, 74%) as a yellow oil.

R_f = 0.68 (*n*-pentane/EtOAc, 9:1). ^{1}H NMR (400 MHz, $CDCl_3$): δ 7.41 – 7.29 (m, 5H), 7.22 (dd, *J* = 8.6, 1.8 Hz, 2H), 6.63 (dd, *J* = 8.6, 1.8 Hz, 2H), 5.20 (d, *J* = 12.2 Hz, 1H), 5.15 (d, *J* = 12.2 Hz, 1H), 4.09 (br. s, 1H), 3.93 (d, *J* = 6.0 Hz, 1H), 2.16 (h, *J* = 6.8 Hz, 1H), 1.32 (s, 9H), 1.05 (app. t, *J* = 6.8 Hz, 6H). ^{13}C NMR (101 MHz, $CDCl_3$): δ 173.9, 145.0, 141.1, 135.8, 128.7, 128.5, 128.4, 126.2, 113.5, 66.8, 62.9, 34.0, 31.8, 31.7, 19.32, 18.8; HRMS (ESI): calcd for $C_{22}H_{29}NO_4$ $[M+Na]^+$: 362.2091; found: 362.2095.

4.9 Diarylation of Tyrosine Methyl Ester

Synthesis of *N*-4-nitrophenyl-*L*-Tyrosine methyl ester (3w')

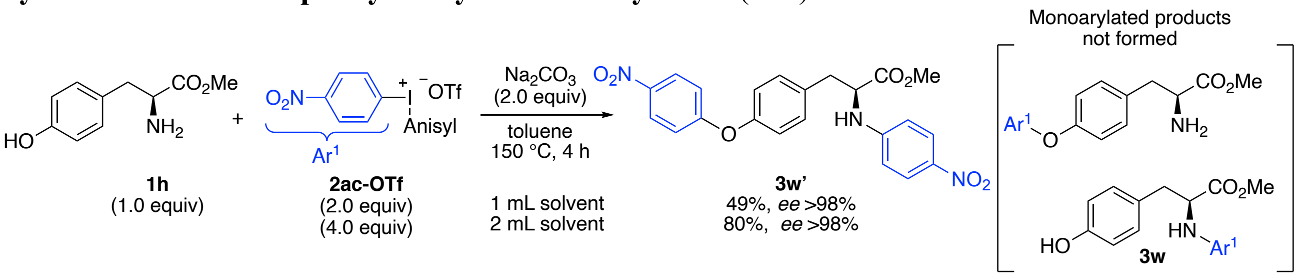

Diarylated product **3w'** was obtained as the only isolated product under the reaction conditions described below. No monoarylated product was observed, which was unexpected as we have published efficient arylation of phenols at room temperature, albeit in the presence of the stronger base *t*BuOK.[59]

TLC analysis of the crude reaction with *p*-NO_2 substituted aryl products are revealed as a bright yellow spot on the TLC plates. In the reaction with **1h**, two yellow spots were observed, one being the product and the other one unknown impurities (*probably traces from salt decomposition, this spot is visible in other reactions with **2ac-OTf***). The crude was purified by silica chromatography without any workup and the column was flushed with EtOAc. ^{1}H NMR analysis showed no monoarylated product nor starting material. Since amino acid derivatives can be purified by rather column chromatography (**1a** has an R_f-value of 0.34 (*n*-pentane/EtOAc, 1.5:1), it is unlikely that the *Ar^1-O* product was stuck in the column.

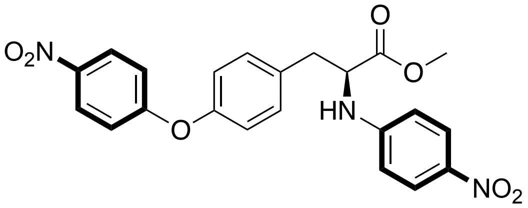

Synthesized according to **GP7** with tyrosine methyl ester (**1h**, 0.2 mmol, 40 mg), diaryliodonium salt **2ac-OTf** (0.8 mmol, 411 mg) and Na_2CO_3 (0.4 mmol, 44 mg), 4 h reaction time and 2 mL toluene. Purification provided **3w'** (0.16 mmol, 50 mg, 80%) as a yellow oil.

R_f = 0.57 (*n*-pentane/EtOAc, 2:1). ^{1}H NMR (400 MHz, $CDCl_3$): δ 8.18 (d, *J* = 9.2 Hz, 2H), 8.07 (d, *J* = 9.2 Hz,

2H), 7.18 (d, J = 8.4 Hz, 2H), 7.04 – 6.94 (m, 4H), 6.55 (d, J = 9.2 Hz, 2H), 5.07 (d, J = 8.1 Hz, 1H), 4.49 (dt, J = 8.2, 5.9 Hz, 1H), 3.77 (s, 3H), 3.27 (dd, J = 13.8, 5.9 Hz, 1H), 3.16 (dd, J = 13.8, 6.2 Hz, 1H). ^{13}C NMR (101 MHz, $CDCl_3$): δ 172.0, 163.1, 154.2, 151.5, 142.9, 139.1, 132.7, 131.3, 126.5, 126.1, 120.8, 117.3, 111.9, 56.8, 52.8, 37.6; HRMS (ESI): calcd for $C_{22}H_{19}N_3O_7$ $[M+Na]^+$: 460.1115; found: 460.1112.

Analysis of enantiomeric purity:

The racemic sample was obtained via **Rac II** (0.03 mmol scale, 82% yield).

Chiral SFC, Diacel OJ-H (0.3 cm ϕ, 15 cm column), 25 °C, [20% MeOH in CO_2, 1.2 mL/min]; t_R: 12.68 min (minor enantiomer), 14.14 min (major enantiomer); *ee* = >98% (e.r. = 99.3:0.7).

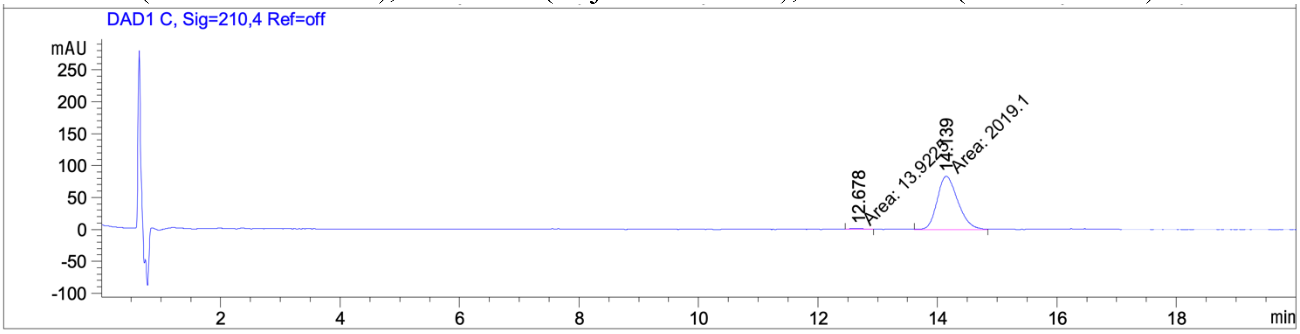

Signal 3: DAD1 C, Sig=210,4 Ref=off

Peak #	RetTime [min]	Type	Width [min]	Area [mAU*s]	Height [mAU]	Area %
1	12.678	MM	0.2652	13.92246	8.75095e-1	0.6848
2	14.139	MM	0.4022	2019.09985	83.66547	99.3152
Totals :				2033.02231	84.54057	

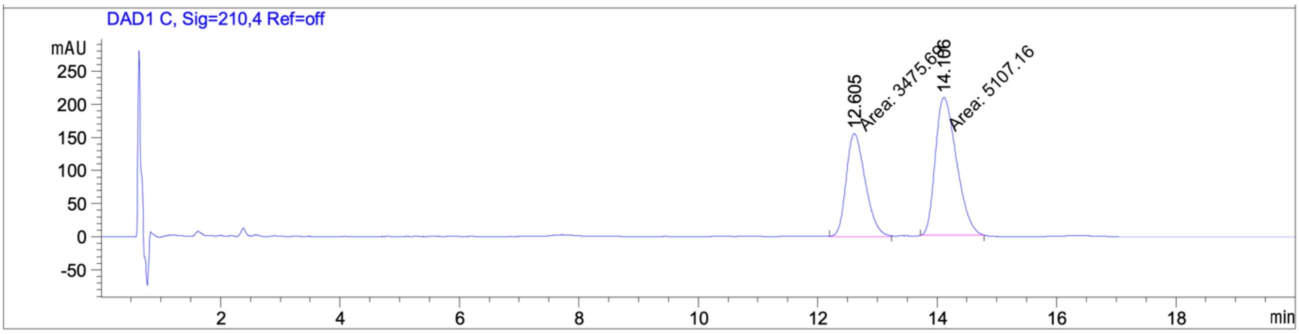

Signal 3: DAD1 C, Sig=210,4 Ref=off

Peak #	RetTime [min]	Type	Width [min]	Area [mAU*s]	Height [mAU]	Area %
1	12.605	MM	0.3716	3475.68970	155.88547	40.4958
2	14.106	MM	0.4094	5107.16064	207.89726	59.5042
Totals :				8582.85034	363.78273	

4.10 ARYLATION OF SECONDARY AMINO ACID ESTERS

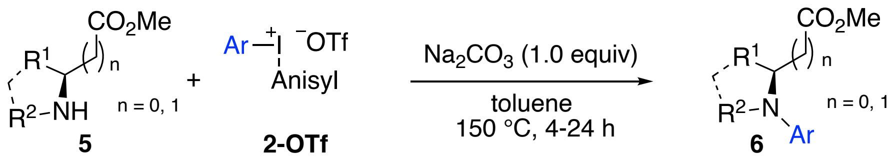

Arylation of proline methyl ester (**5a**) followed **GP7** with a small difference. The air exchange occurred without applying vacuum and instead argon was flushed to the system together with an air outlet.

N-4-nitrophenyl-*L*-proline methyl ester (6a)

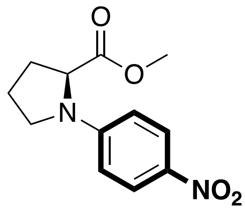

Synthesized according to **GP7** with proline methyl ester (**5a**, 0.2 mmol, 26 mg), diaryliodonium salt **2ac-OTf** (0.4 mmol, 203 mg) and Na_2CO_3 (0.2 mmol, 22 mg), 4 h reaction time. Purification provided **5a** (0.18 mmol, 24 mg, 91%) as a bright yellow oil.

R_f = 0.39 (*n*-pentane/EtOAc, 4:1). ^{1}H NMR (400 MHz, $CDCl_3$): δ 8.13 – 8.05 (m, 2H), 6.50 – 6.42 (m, 2H), 4.37 (dd, *J* = 8.6, 2.2 Hz, 1H), 3.73 (s, 3H), 3.68-3.60 (m, 1H), 3.49 – 3.43 (m, 1H), 2.43 – 2.27 (m, 1H), 2.28 – 2.06 (m, 3H). ^{13}C NMR (101 MHz, $CDCl_3$): δ 173.2, 151.2, 137.9, 126.3, 111.1, 60.9, 52.6, 48.7, 30.9, 23.7; HRMS (ESI): calcd for $C_{12}H_{14}N_2O_4$ $[M+Na]^+$: 273.0846; found: 273.0845.

Analysis of enantiomeric purity:

The racemic sample was obtained via **Rac I** followed by **GP7** at 130 °C (0.2 mmol scale, 71% yield). Chiral SFC, Diacel OJ-H (0.3 cm ϕ, 15 cm column), 25 °C, [5% MeOH in CO_2, 0.8 mL/min]; t_R: 5.66 min (minor enantiomer), 6.20 min (major enantiomer); *ee* = 92% (e.r. = 95.7:4.3).

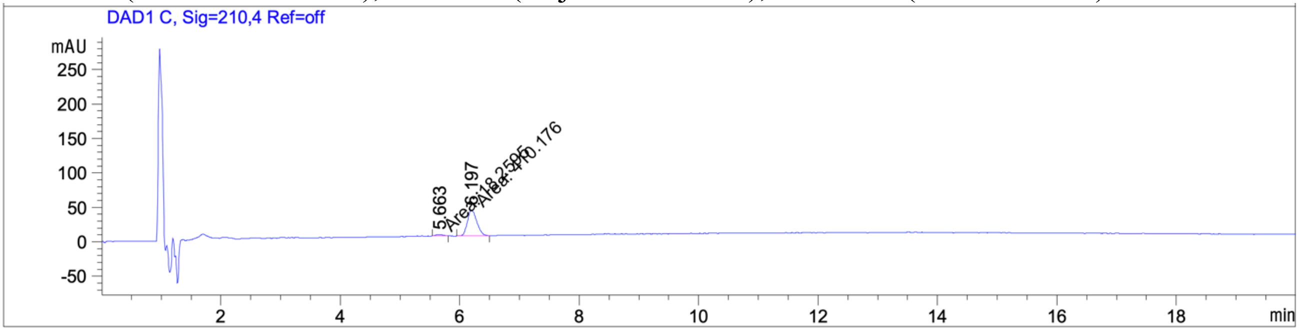

Signal 3: DAD1 C, Sig=210,4 Ref=off

Peak #	RetTime [min]	Type	Width [min]	Area [mAU*s]	Height [mAU]	Area %
1	5.663	MM	0.1431	18.25948	2.12703	4.2619
2	6.197	MM	0.1841	410.17560	37.13997	95.7381
Totals :				428.43508	39.26700	

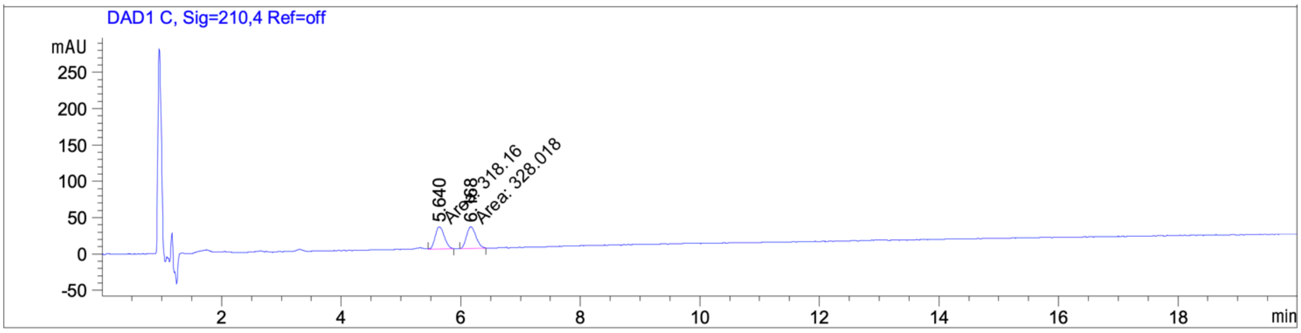

Signal 3: DAD1 C, Sig=210,4 Ref=off

Peak #	RetTime [min]	Type	Width [min]	Area [mAU*s]	Height [mAU]	Area %
1	5.640	MM	0.1753	318.15967	30.25718	49.2372
2	6.168	MM	0.1832	328.01797	29.83612	50.7628
Totals :				646.17764	60.09330	

N-4-cyano-2-fluorophenyl-L-proline methyl ester (6b)

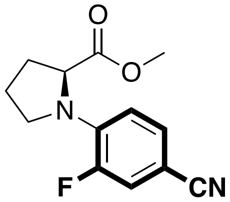

Synthesized according to **GP7** with proline methyl ester (**5a**, 0.2 mmol, 26 mg), diaryliodonium salt **2cc-OTf** (0.4 mmol, 202 mg) and Na_2CO_3 (0.2 mmol, 22 mg), 4 h reaction time. Purification provided **6b** (0.15 mmol, 38 mg, 75%) as a pale yellow viscous oil. *Reaction at 130 °C gave **6b** in 85% (0.17 mmol, 42 mg).* R_f = 0.46 (*n*-pentane/EtOAc, 4:1). ^{1}H NMR (400 MHz, $CDCl_3$): δ 7.28 (dd, J = 8.8, 1.9 Hz, 1H), 7.22 – 7.16 (m, 1H), 6.61 (t, J = 8.8 Hz, 1H), 4.66 (ddd, J = 8.5, 4.9, 3.2 Hz, 1H), 3.73 (s, 3H), 3.69 – 3.59 (m, 1H), 3.51 (app. q, J = 8.7 Hz, 1H), 2.32 (dq, J = 12.7, 8.6 Hz, 1H), 2.20 – 2.09 (m, 1H), 2.09 – 1.98 (m, 2H). ^{13}C NMR (101 MHz, $CDCl_3$): δ173.5 (d, J = 1.6 Hz), 150.0 (d, $^1J_{F\text{-}C}$ = 241.8 Hz), 139.4 (d, $^{2?}J_{F\text{-}C}$ = 8.5 Hz), 129.7 (d, $^{3or4}J_{F\text{-}C}$ = 2.4 Hz), 119.7 (d, $^2J_{F\text{-}C}$ = = 25.3 Hz), 119.2 (d, $^{3or4}J_{F\text{-}C}$ = 2.4 Hz), 115.4 (d, $^3J_{F\text{-}C}$ = 5.8 Hz), 98.7 (d, J = 9.2 Hz), 62.0 (d, J = 8.1 Hz), 52.4, 50.2 (d, J = 2.5 Hz), 31.2 (d, J = 1.6 Hz), 23.1 (d, J = 1.4 Hz). ^{19}F NMR (376 MHz, $CDCl_3$) δ -127.7; HRMS (ESI): calcd for $C_{13}H_{13}FN_2O_2$ $[M+Na]^+$: 271.0853; found: 271.0848.

Analysis of enantiomeric purity:

The racemic sample was obtained via **Rac I** followed by **GP7** at 130 °C (0.22 mmol scale, 85% yield). Chiral SFC, Diacel OJ-H (0.3 cm ϕ, 15 cm column), 25 °C, [10% MeOH in CO_2, 0.8 mL/min]; t_R: 2.00 min (minor enantiomer), 3.07 min (major enantiomer); *ee* = 94% (e.r. = 97.0:3.0).

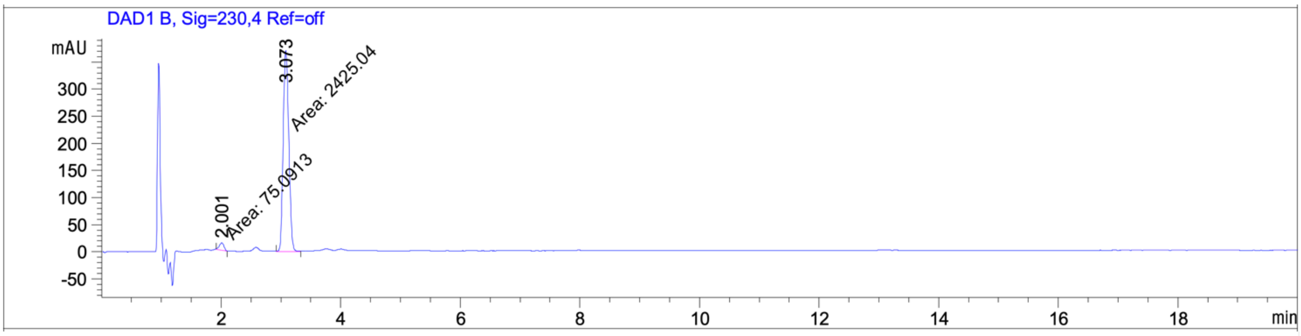

Signal 2: DAD1 B, Sig=230,4 Ref=off

Peak #	RetTime [min]	Type	Width [min]	Area [mAU*s]	Height [mAU]	Area %
1	2.001	MM	0.0892	75.09129	14.03458	3.0035
2	3.073	MM	0.1087	2425.03809	371.72253	96.9965
Totals :				2500.12937	385.75711	

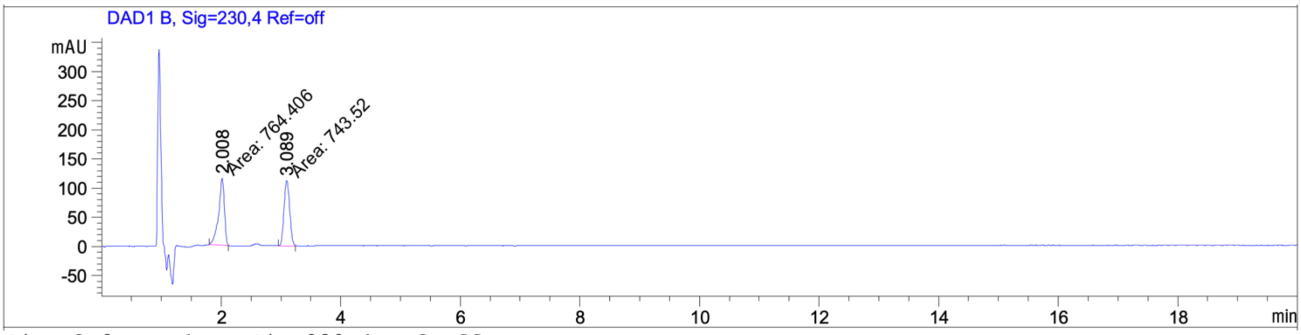

Signal 2: DAD1 B, Sig=230,4 Ref=off

Peak #	RetTime [min]	Type	Width [min]	Area [mAU*s]	Height [mAU]	Area %
1	2.008	MM	0.1113	764.40643	114.48495	50.6925
2	3.089	MM	0.1102	743.52045	112.46719	49.3075
Totals :				1507.92688	226.95214	

_N_-(2-chloro-5-(trifluoromethyl)phenyl-_L_-proline methyl ester (6c)

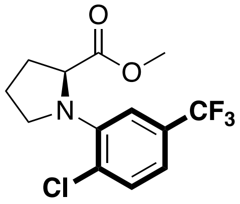

Synthesized according to **GP7** with proline methyl ester (**5a**, 0.2 mmol, 26 mg), diaryliodonium salt **2fc-OTf** (0.4 mmol, 225 mg) and Na_2CO_3 (0.2 mmol, 22 mg), 4 h reaction time. Purification provided **6c** (0.13 mmol, 41 mg, 67%) as a colorless oil.

Reaction at 130 °C gave ***5a*** *in 68% yield (0.14 mmol, 42 mg, 68%).*

R_f = 0.54 (*n*-pentane/EtOAc, 4:1) ^{1}H NMR (400 MHz, $CDCl_3$): δ 7.35 (dd, J = 8.4, 1.0 Hz, 1H), 7.12 (d, J = 2.1 Hz, 1H), 7.02 (dd, J = 8.4, 2.1 Hz, 1H), 4.93 (dd, J = 8.2, 5.0 Hz, 1H), 3.76 (td, J = 8.7, 6.9 Hz, 1H), 3.63 (s, 3H), 3.42 – 3.33 (m, 1H), 2.48 – 2.35 (m, 1H), 2.15 – 1.91 (m, 3H). ^{13}C NMR (101 MHz, $CDCl_3$): δ 174.0, 145.4, 131.8, 129.8 (q, $^2J_{F-C}$ = 32.5 Hz), 126.3 (q, $^4J_{F-C}$ = 1.7 Hz), 124. 1 (q, $^1J_{F-C}$ = 272.1 Hz), 117.19 (q, $^3J_{F-C}$ = 3.8 Hz), 115.32 (q, $^3J_{F-C}$ = 3.9 Hz), 61.7, 52.1, 51.5, 31.1, 23.9. ^{19}F NMR (376 MHz, $CDCl_3$) δ - 62.6; HRMS (ESI): calcd for $C_{13}H_{13}ClF_3NO_2$ $[M+Na]^+$: 330.0479; found: 330.0478.

Analysis of enantiomeric purity:

The racemic sample was obtained via **Rac I** followed by **GP7** at 130 °C (0.2 mmol scale, 68% yield).

HPLC, Chiralpak-AD-H (0.46 cm ϕ, 25 cm column), 25 °C, [Hexane/*i*PrOH (90:10), 1.0 mL/min]; t_R: 3.86 min (minor enantiomer), 4.15 min (major enantiomer); *ee* = 92% (e.r. = 95.7:4.3).

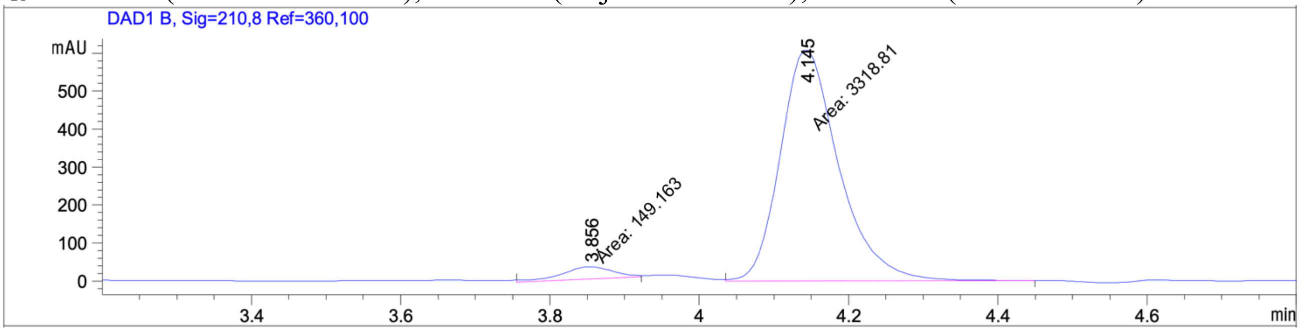

Signal 2: DAD1 B, Sig=210,8 Ref=360,100

Peak #	RetTime [min]	Type	Width [min]	Area [mAU*s]	Height [mAU]	Area %
1	3.856	MM	0.0783	149.16272	31.73822	4.3011
2	4.145	MM	0.0904	3318.81396	611.94708	95.6989
Totals :				3467.97668	643.68530	

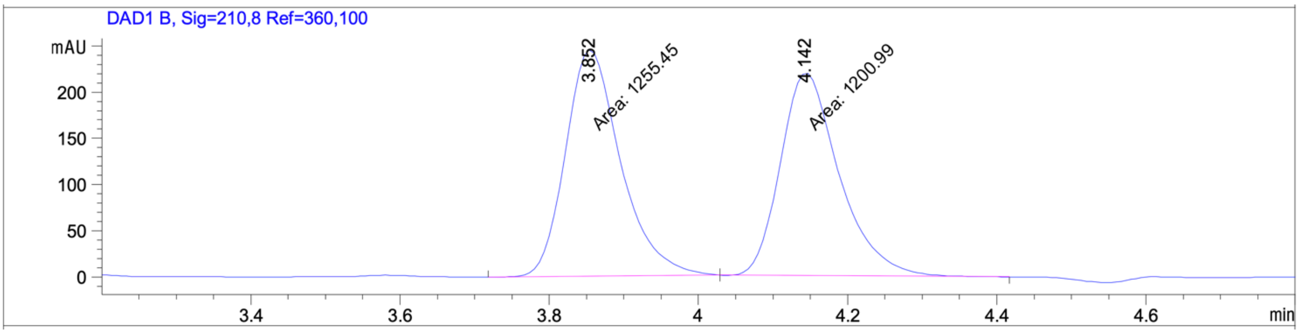

Signal 2: DAD1 B, Sig=210,8 Ref=360,100

Peak #	RetTime [min]	Type	Width [min]	Area [mAU*s]	Height [mAU]	Area %
1	3.852	MM	0.0853	1255.44678	245.28217	51.1085
2	4.142	MM	0.0912	1200.98779	219.55569	48.8915
Totals :				2456.43457	464.83786	

***N*-phenyl-*L*-proline methyl ester (6d)**

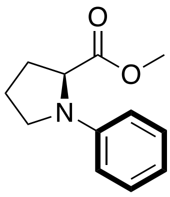

Synthesized according to **GP7** with proline methyl ester (**5a**, 0.2 mmol, 26 mg), diaryliodonium salt **2ia-OTf** (0.4 mmol, 177 mg) and Na_2CO_3 (0.2 mmol, 22 mg), 24 h reaction time. Purification provided **6d** (0.11 mmol, 23 mg, 55%) as a colorless oil. R_f = 0.60 (*n*-pentane/EtOAc, 9:1). ^{1}H NMR (400 MHz, $CDCl_3$): δ 7.25 – 7.20 (m, 2H), 6.72 (t, *J* = 7.3 Hz, 1H), 6.55 (d, *J* = 8.7 Hz, 2H), 4.26 (dd, *J* = 8.7, 2.1 Hz, 1H), 3.72 (s, 3H), 3.59 (td, *J* = 7.8, 2.6 Hz, 1H), 3.37 (dt, *J* = 8.7, 7.3 Hz, 1H), 2.37 – 2.00 (m, 4H). ^{13}C NMR (101 MHz, $CDCl_3$): δ 175.1, 146.8, 129.4, 116.8, 112.0, 60.9, 52.2, 48.4, 31.0, 24.0. The analytical data are consistent with previous reports.[30d, 60]

Analysis of enantiomeric purity:

The racemic sample was obtained via **Rac I** followed by **GP7** (0.2 mmol scale, 53% yield).

Chiral SFC, Diacel OJ-H (0.3 cm ϕ, 15 cm column), 25 °C, [10% MeOH in CO_2, 0.8 mL/min]; t_R: 3.43 min (minor enantiomer), 4.87 min (major enantiomer); *ee* = 90% (e.r. = 94.9:5.1).

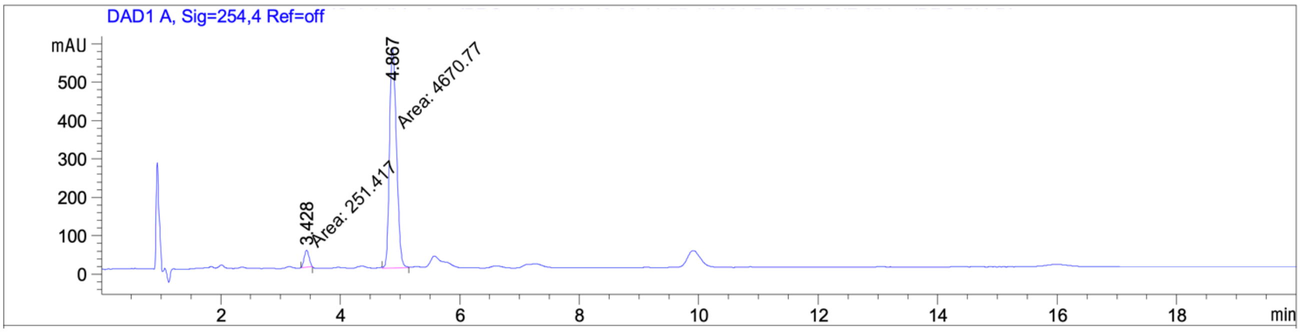

Signal 1: DAD1 A, Sig=254,4 Ref=off

Peak #	RetTime [min]	Type	Width [min]	Area [mAU*s]	Height [mAU]	Area %
1	3.428	MM	0.0942	251.41740	44.50146	5.1078
2	4.867	MM	0.1355	4670.76758	574.55829	94.8922

Totals : 4922.18498 619.05975

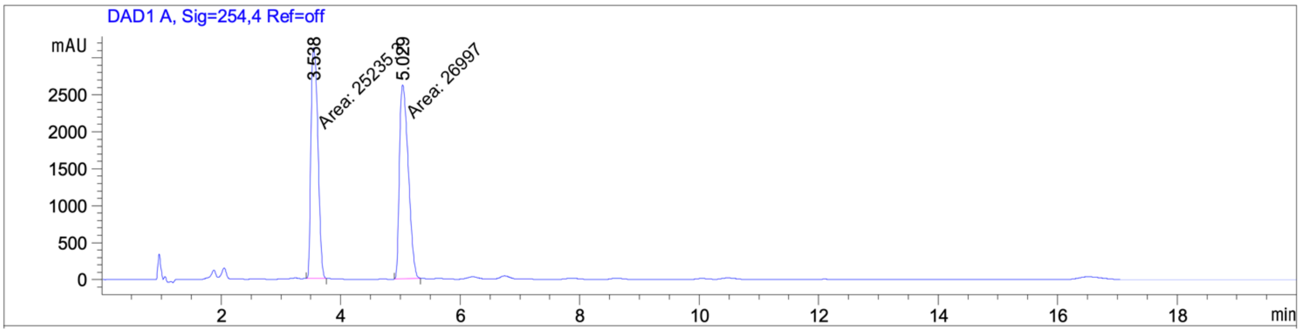

Signal 1: DAD1 A, Sig=254,4 Ref=off

Peak #	RetTime [min]	Type	Width [min]	Area [mAU*s]	Height [mAU]	Area %
1	3.538	MM	0.1349	2.52353e4	3118.01392	48.3137
2	5.029	MM	0.1717	2.69970e4	2621.00757	51.6863

Totals : 5.22323e4 5739.02148

***N*-mesityl-*L*-proline methyl ester (6e)**

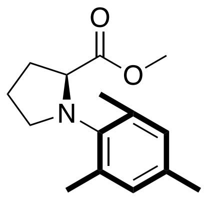

Synthesized according to **GP7** with proline methyl ester (**5a**, 0.2 mmol, 26 mg), diaryliodonium salt **2kb-OTf** (0.4 mmol, 216 mg) and Na_2CO_3 (0.2 mmol, 22 mg), 4 h reaction time. Purification provided **6e** (0.13 mmol, 32 mg, 62%) as a pale yellow oil.

R_f = 0.54 (*n*-pentane/EtOAc, 9:1). ^{1}H NMR (400 MHz, $CDCl_3$): δ 6.86 (s, 2H), 4.10 (dd, *J* = 8.5, 2.6 Hz, 1H), 3.61 (s, 3H), 3.43 (td, *J* = 7.8, 3.3 Hz, 1H), 3.08 (td, *J* = 8.5, 6.5 Hz, 1H), 2.27 (s, 6H), 2.26 (s, 3H), 2.25 – 2.12 (m, 2H), 2.08 – 1.97 (m, 1H). ^{13}C NMR (101 MHz, $CDCl_3$): δ 175.8, 141.7, 135.2, 129.6, 63.1, 52.3, 51.2, 30.9, 25.9, 20.9, 18.6; HRMS (ESI): calcd for $C_{15}H_{21}NO_2$ $[M+Na]^+$: 270.1465; found: 270.1461.

Analysis of enantiomeric purity:

The racemic sample was obtained via **Rac I** followed by **GP7** with **2kc-OTf** (0.2 mmol scale, 35% yield).

HPLC, Chiralpak-OJ-H (0.46 cm ϕ, 25 cm column), 25 °C, [hexane/*i*PrOH (90:10), 1.0 mL/min]; t_R: 4.69 min (major enantiomer), 6.64 min (minor enantiomer) ; *ee* = 90% (e.r. = 94.7 : 5.3).

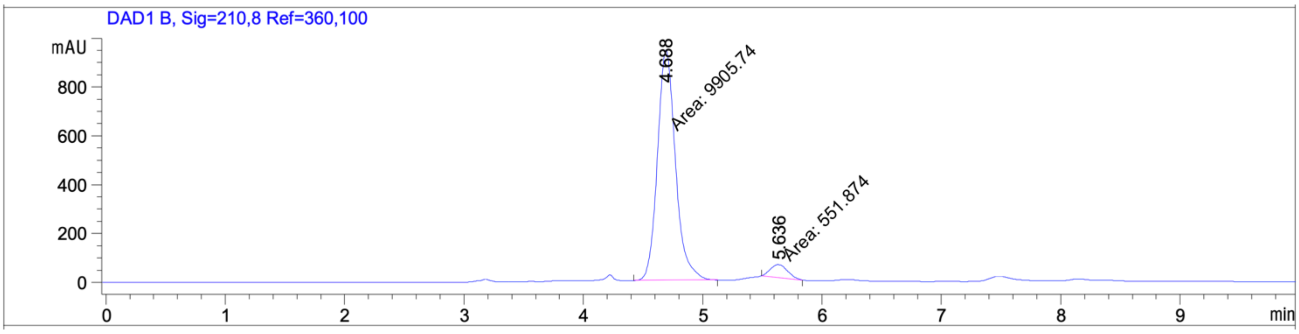

Signal 2: DAD1 B, Sig=210,8 Ref=360,100

Peak #	RetTime [min]	Type	Width [min]	Area [mAU*s]	Height [mAU]	Area %
1	4.688	MM	0.1749	9905.74316	944.16699	94.7228
2	5.636	MM	0.1702	551.87366	54.04318	5.2772
Totals :				1.04576e4	998.21017	

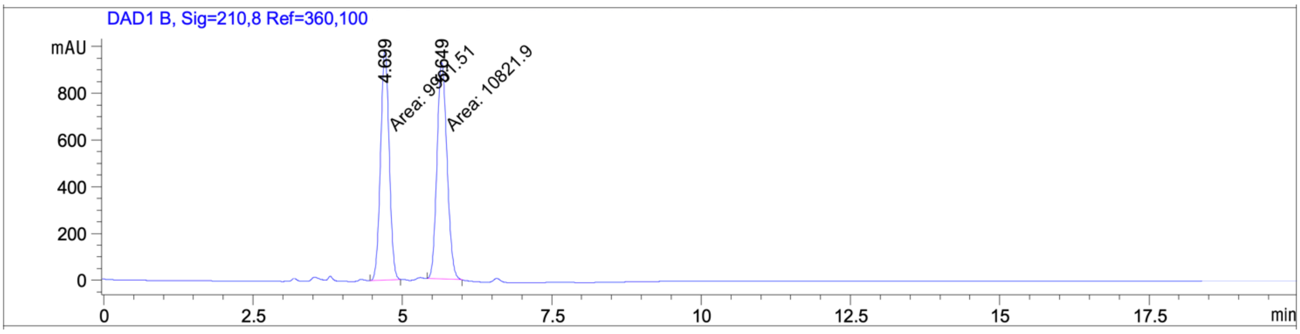

Signal 2: DAD1 B, Sig=210,8 Ref=360,100

Peak #	RetTime [min]	Type	Width [min]	Area [mAU*s]	Height [mAU]	Area %
1	4.699	MM	0.1674	9901.51367	985.64178	47.7794
2	5.649	MM	0.1946	1.08219e4	926.82104	52.2206
Totals :				2.07234e4	1912.46283	

***N*-methyl-*N*-4-nitrophenyl-*L*-phenylalanine methyl ester (6f)**

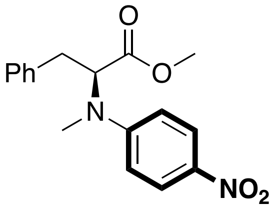

Synthesized according to **GP7** with *N*-Methyl phenylalanine methyl ester (**5c-Me**, 0.2 mmol, 38 mg), diaryliodonium salt **2ac-OTf** (0.4 mmol, 203 mg) and Na_2CO_3 (0.2 mmol, 22 mg), 4 h reaction time. Purification provided **6f** (0.11 mmol, 35 mg, 58%) as a bright yellow oil.

R_f = 0.24 (*n*-pentane/EtOAc, 9:1). ^{1}H NMR (400 MHz, $CDCl_3$): δ 8.05 (d, *J* = 9.5 Hz, 2H), 7.30 – 7.11 (m, 5H), 6.60 (d, *J* = 9.5 Hz, 2H), 4.76 (dd, *J* = 9.4, 5.8 Hz, 1H), 3.74 (s, 3H), 3.41 (dd, *J* = 14.3, 5.8 Hz, 1H), 3.14 (dd, *J* = 14.3, 9.4 Hz, 1H), 2.99 (s, 3H). ^{13}C NMR (101 MHz, $CDCl_3$): δ 171.3, 154.1, 138.2, 136.8, 128.9, 128.9, 127.2, 126.1, 111.4, 63.2, 52.6, 35.8, 34.5; HRMS (ESI): calcd for $C_{17}H_{18}N_2O_4$ $[M+Na]^+$: 314.3351; found: 314.3348.

Analysis of enantiomeric purity:

The racemic sample was obtained via **Rac II** (0.15 mmol scale, 50% yield).

HPLC, Chiralpak-IA (0.46 cm ϕ, 25 cm column), 25 °C, [Hexane/*i*PrOH (90:10), 1.0 mL/min]; t_R: 15.80 min (major enantiomer), 16.67 min (minor enantiomer); *ee* (major enantiomer) = 94% (e.r. = 97.1:2.9).

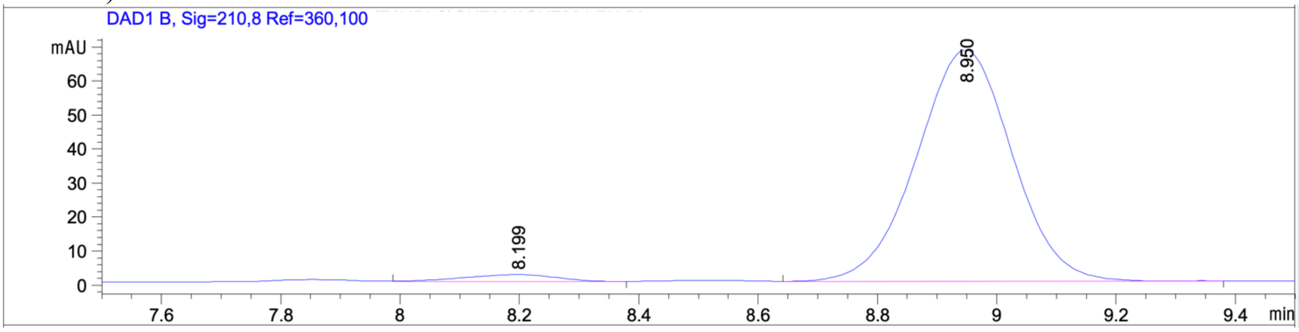

Signal 2: DAD1 B, Sig=210,8 Ref=360,100

Peak #	RetTime [min]	Type	Width [min]	Area [mAU*s]	Height [mAU]	Area %
1	8.199	VB	0.1573	22.32487	2.04528	2.8714
2	8.950	BB	0.1716	755.15814	67.99866	97.1286
Totals :				777.48301	70.04395	

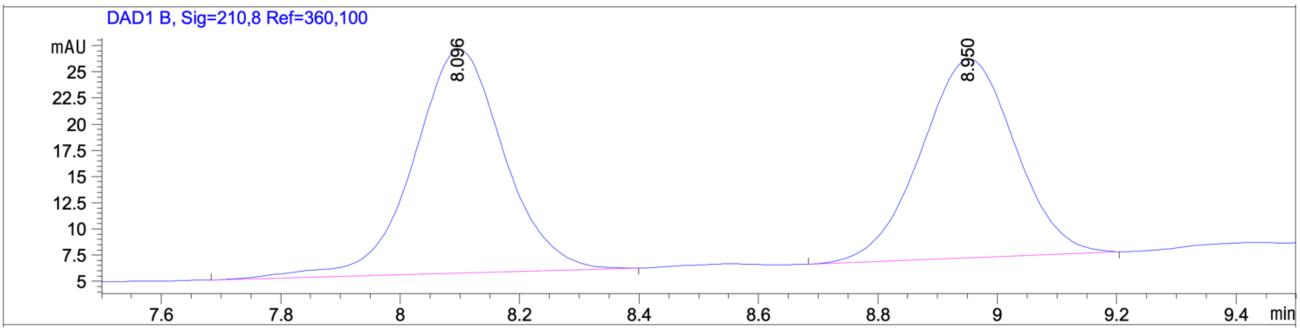

Signal 2: DAD1 B, Sig=210,8 Ref=360,100

Peak #	RetTime [min]	Type	Width [min]	Area [mAU*s]	Height [mAU]	Area %
1	8.096	BB	0.1613	225.99329	21.37521	52.0680
2	8.950	BB	0.1678	208.04131	18.98472	47.9320
Totals :				434.03459	40.35994	

N-4-nitrophenyl-β-phenylalanine methyl ester (6g)

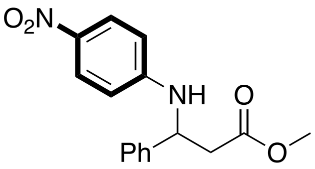

Synthesized according to **GP7** with proline methyl ester (**5c**, 0.2 mmol, 36 mg), diaryliodonium salt **2ac-OTf** (0.4 mmol, 203 mg) and Na_2CO_3 (0.2 mmol, 22 mg), 4 h reaction time. Purification provided **6g** (0.15 mmol, 47 mg, 74%) as a bright yellow oil.

R_f = 0.55 (*n*-pentane/EtOAc, 9:1). ^{1}H NMR (400 MHz, $CDCl_3$): δ 8.00 (d, *J* = 9.2 Hz, 2H), 7.40 – 7.27 (m, 5H), 6.51 (d, *J* = 9.2 Hz, 2H), 5.59 (d, *J* = 6.7 Hz, 1H), 4.92 (td, *J* = 7.2, 5.2 Hz, 1H), 3.66 (s, 3H), 2.92 (dd, *J* = 15.2, 5.2 Hz, 1H), 2.85 (dd, *J* = 15.2, 7.5 Hz, 1H). ^{13}C NMR (101 MHz, $CDCl_3$): δ 171.3, 152.1, 140.4, 138.6, 129.2, 128.2, 126.3, 126.1, 112.2, 54.5, 52.2, 42.1. The analytical data are consistent with previous reports.[55b]

5 Refrences

[1] A. I. Vogel, B. S. Furniss, A. J. Hannaford, V. Rogers, P. W. G. Smith, A. R. Tatchell, *Vogel's Textbook of Practical Organic Chemistry 5th Ed.*, Prentice Hall: Harlow, , **1996**.

[2] a) T. Wirth, Hypervalent Iodine Chemistry Vol. 373, Springer International Publishing, Cham, **2016**; b) A. Yoshimura, V. V. Zhdankin, *Chem. Rev.* **2016**, *116*, 3328-3435; c) B. Olofsson, I. Marek, Z. Rappoport, *Patai's Chemistry of Functional Groups: The Chemistry of Hypervalent Halogen Compounds* **2019**, Eds., Wiley, 2019; d) N. W. Alcock, R. M. Countryman, *J. Chem. Soc., Dalton Trans* **1977**, 217-219; e) M. Ochiai, *Top. Curr. Chem.* **2003**, *224*, 5-68; f) I. Sokolovs, D. Lubriks, E. Suna, *J. Am. Chem. Soc.* **2014**, *136*, 6920-6928.

[3] B. Olofsson, in *Top. Curr. Chem., Vol. 373* (Ed.: T. Wirth), Springer International Publishing, Cham, **2016**, pp. 135-166.

[4] a) M. Bielawski, B. Olofsson, *Chem. Commun.* **2007**, 2521-2523; b) M. Bielawski, M. Zhu, B. Olofsson, *Adv. Synth. Catal.* **2007**, *349*, 2610-2618.

[5] M. Zhu, N. Jalalian, B. Olofsson, *Synlett* **2008**, *4*, 592-596.

[6] M. Bielawski, D. Aili, B. Olofsson, *J. Org. Chem.* **2008**, *73*, 4602-4607.

[7] M. Bielawski, J. Malmgren, L. M. Pardo, Y. Wikmark, B. Olofsson, *ChemistryOpen* **2014**, *3*, 19-22.

[8] M. Reitti, P. Villo, B. Olofsson, *Angew. Chem. Int. Ed.* **2016**, *55*, 8928-8932.

[9] a) N. Purkait, G. Kervefors, E. Linde, B. Olofsson, *Angew. Chem. Int. Ed.* **2018**, *57*, 11427-11431; b) G. L. Tolnai, U. J. Nilsson, B. Olofsson, *Angew. Chem. Int. Ed.* **2016**, *55*, 11226-11230.

[10] M. Reitti, R. Gurubrahamam, M. Walther, E. Lindstedt, B. Olofsson, *Org. Lett.* **2018**, *20*, 1785-1788.

[11] a) T. B. Petersen, R. Khan, B. Olofsson, *Org. Lett.* **2011**, *13*, 3462-3465; b) Z. Gonda, Z. Novák, *Chem. Eur. J.* **2015**, *21*, 16801-16806.

[12] a) J.-H. Chun, S. Lu, V. W. Pike, *Eur. J. Org. Chem.* **2011**, *2011*, 4439-4447; b) R. Ghosh, E. Lindstedt, N. Jalalian, B. Olofsson, *ChemistryOpen* **2014**, *3*, 54-57; c) N. Jalalian, T. B. Petersen, B. Olofsson, *Chem. Eur. J.* **2012**, *18*, 14140-14149.

[13] E. Lindstedt, M. Reitti, B. Olofsson, *J. Org. Chem.* **2017**, *82*, 11909-11914.

[14] Y. Wang, C. Wang, Y. Wang, L. Dong, J. Sun, *RSC Advances* **2015**, *5*, 12354-12357.

[15] D. Zhu, M. Li, Z. Wu, Y. Du, B. Luo, P. Huang, S. Wen, *Eur. J. Org. Chem.* **2019**, *2019*, 4566-4571.

[16] a) G. Wang, B. Xiong, C. Zhou, Y. Liu, W. Xu, C. A. Yang, K. W. Tang, W. Y. Wong, *Chem Asian J* **2019**, *14*, 4365-4374; b) M. Jiang, J. Guo, B. Liu, Q. Tan, B. Xu, *Org. Lett.* **2019**, *21*, 8328-8333.

[17] a) E. A. Merritt, V. M. T. Carneiro, L. F. Silva, B. Olofsson, *J. Org. Chem.* **2010**, *75*, 7416-7419; b) T. Dohi, M. Ito, K. Morimoto, Y. Minamitsuji, N. Takenaga, Y. Kita, *Chem. Commun.* **2007**, 4152-4154.

[18] P. Kazmierczak, L. Skulski, *Synthesis* **1995**, 1027-1032.

[19] T. L. Seidl, S. K. Sundalam, B. McCullough, D. R. Stuart, *J. Org. Chem.* **2016**, *81*, 1998-2009.

[20] E. Lindstedt, E. Stridfeldt, B. Olofsson, *Org. Lett.* **2016**, *18*, 4234-4237.

[21] L. Ferrié, J. Fenneteau, B. Figadère, *Org. Lett.* **2018**, *20*, 3192-3196.

[22] a) R. R. Mehra, P. Tiwari, A. Basu, A. DuttKonar, *New J. Chem.* **2019**, *43*, 11666-11678; b) C. D. McCune, M. L. Beio, J. M. Sturdivant, R. de la Salud-Bea, B. M. Darnell, D. B. Berkowitz, *J. Am. Chem. Soc.* **2017**, *139*, 14077-14089.

[23] a) T. Maegawa, K. Otake, K. Hirosawa, A. Goto, H. Fujioka, *Org. Lett.* **2012**, *14*, 4798-4801; b) Ø. Jacobsen, H. Maekawa, N.-H. Ge, C. H. Görbitz, P. Rongved, O. P. Ottersen, M. Amiry-Moghaddam, J. Klaveness, *J. Org. Chem.* **2011**, *76*, 1228-1238.

[24] L. V. Adriaenssens, R. C. Hartley, *J. Org. Chem.* **2007**, *72*, 10287-10290.

[25] J. Liu, C. Tian, T. Jiang, Y. Gao, Y. Zhou, M. Li, L. Du, *ACS Medicinal Chemistry Letters* **2017**, *8*, 428-432.

[26] N. Naganna, N. Madhavan, *J. Org. Chem.* **2014**, *79*, 11549-11557.

[27] a) K. C. Nadimpally, K. Thalluri, N. B. Palakurthy, A. Saha, B. Mandal, *Tetrahedron Lett.* **2011**, *52*, 2579-2582; b) J. Feng, S. Liang, S.-Y. Chen, J. Zhang, S.-S. Fu, X.-Q. Yu, *Adv. Synth. Catal.* **2012**, *354*, 1287-1292.

[28] a) B. F. Fisher, L. Guo, B. S. Dolinar, I. A. Guzei, S. H. Gellman, *J. Am. Org. Chem.* **2015**, *137*, 6484-6487; b) A. M. King, M. De Ryck, R. Kaminski, A. Valade, J. P. Stables, H. Kohn, *J. Med. Chem.* **2011**, *54*, 6432-6442.

[29] I. D'Acquarica, A. Cerreto, G. Delle Monache, F. Subrizi, A. Boffi, A. Tafi, S. Forli, B. Botta, *J. Org. Chem.* **2011**, *76*, 4396-4407.

[30] a) A. Dominguez-Huerta, I. Perepichka, C.-J. Li, *Commun. Chem.* **2018**, *1*, 45; b) A. Isidro-Llobet, M. Álvarez, F. Albericio, *Chem. Rev.* **2009**, *109*, 2455-2504; c) Nonappa, K. Ahonen, M. Lahtinen, E. Kolehmainen, *Green Chem.* **2011**, *13*, 1203-1209; d) J. D. McKerrow, J. M. A. Al-Rawi, P. Brooks, *Synthetic Communications* **2010**, *40*, 1161-1179.

[31] a) J. Britton, J. M. Chalker, C. L. Raston, *Chem. Eur. J.* **2015**, *21*, 10660-10665; b) K. Omata, S. Aoyagi, K. Kabuto, *Tetrahedron: Asymmetry* **2004**, *15*, 2351-2356.

[32] A. Sakakura, Y. Koshikari, K. Ishihara, *Tetrahedron Lett.* **2008**, *49*, 5017-5020.
[33] M. Kapoor, P. Chand-Thakuri, M. C. Young, *J. Am. Org. Chem.* **2019**, *141*, 7980-7989.
[34] G. Bartoli, G. Di Antonio, R. Giovannini, S. Giuli, S. Lanari, M. Paoletti, E. Marcantoni, *J. Org. Chem.* **2008**, *73*, 1919-1924.
[35] F. P. Garrido González, T. Mancilla Percino, *Bioorg. Chem.* **2020**, *102*, 104080.
[36] a) S. Vudhgiri, R. B. N. Prasad, Y. Poornachandra, C. Ganesh Kumar, E. Anjaneyulu, K. Sirisha, R. C. R. Jala, *J. Chem. Scien.* **2017**, *129*, 663-677; b) A. Nagai, J. Ishikawa, H. Kudo, T. Endo, *J. Polym. Sci., Part A: Polym. Chem.* **2004**, *42*, 1143-1153.
[37] H. Zhu, G. Xu, H. Du, C. Zhang, N. Ma, W. Zhang, *J. Catal.* **2019**, *374*, 217-229.
[38] H. Tsuji, H. Yamamoto, *Journal of the American Chemical Society* **2016**, *138*, 14218-14221.
[39] A. M. Deveau, N. E. Costa, E. M. Joshi, T. L. Macdonald, *Bio. Med. Chem. Lett.* **2008**, *18*, 3522-3525.
[40] Y. Kawase, T. Yamagishi, T. Kutsuma, T. Kataoka, K. Ueda, T. Iwakuma, T. Nakata, T. Yokomatsu, *Synthesis* **2010**, *10*, 1673-1677.
[41] M. J. Terrey, C. C. Perry, W. B. Cross, *Org. Lett.* **2019**, *21*, 104-108.
[42] B. Ticconi, A. Colcerasa, S. Di Stefano, O. Lanzalunga, A. Lapi, M. Mazzonna, G. Olivo, *RSC Advances* **2018**, *8*, 19144-19151.
[43] M. Ordóñez, R. De la Cruz-Cordero, M. Fernández-Zertuche, M. Angel Muñoz-Hernández, O. García-Barradas, *Tetrahedron: Asymmetry* **2004**, *15*, 3035-3043.
[44] P. Villo, G. Kervefors, B. Olofsson, *Chem. Commun.* **2018**, *54*, 8810-8813.
[45] A. H. Sandtorv, D. R. Stuart, *Angew. Chem. Int. Ed.* **2016**, *55*, 15812-15815.
[46] a) J. Malmgren, S. Santoro, N. Jalalian, F. Himo, B. Olofsson, *Chem. Eur. J.* **2013**, *19*, 10334-10342; b) D. R. Stuart, *Chem. Eur. J.* **2017**, *23*, 15852-15863.
[47] T. Dohi, M. Ito, N. Yamaoka, K. Morimoto, H. Fujioka, Y. Kita, *Angew. Chem. Int. Ed.* **2010**, *49*, 3334-3337.
[48] a) F. M. Beringer, S. J. Huang, *J. Org. Chem.* **1964**, *29*, 445-448; b) T. Akiyama, Y. Imasaki, M. Kawmisi, *Chem. Lett.* **1974**, *6*, 229-230; c) S. K. Sundalam, A. Nilova, T. L. Seidl, D. R. Stuart, *Angew. Chem. Int. Ed.* **2016**, *55*, 8431-8434; d) T. Kitamura, M. Yamane, K. Inoue, M. Todaka, N. Fukatsu, Z. Meng, Y. Fujiwara, *J. Am. Chem. Soc.* **1999**, *121*, 11674-11679; e) H. Chen, J. Han, L. Wang, *Beilstein J. Org. Chem.* **2018**, *14*, 354-363.
[49] E. Stridfeldt, E. Lindstedt, M. Reitti, J. Blid, P.-O. Norrby, B. Olofsson, *Chem. Eur. J.* **2017**, *23*, 13249-13258.
[50] J. J. Lubinkowski, J. W. Knapczyk, J. L. Calderon, L. R. Petit, W. E. McEwen, *J. Org. Chem.* **1975**, *40*, 3010-3015.
[51] a) A. Tota, S. St John-Campbell, E. L. Briggs, G. O. Estévez, M. Afonso, L. Degennaro, R. Luisi, J. A. Bull, *Org. Lett.* **2018**, *20*, 2599-2602; b) R. Frei, M. D. Wodrich, D. P. Hari, P.-A. Borin, C. Chauvier, J. Waser, *J. Am. Org. Chem.* **2014**, *136*, 16563-16573.
[52] a) Y. Qiao, B. Chen, Y. Yang, X. Wang, Y. Xu, H. Li, *Dalton Transactions* **2016**, *45*, 1310-1314; b) E. Fischer, L. H. Cone, *Justus Liebigs Annalen der Chemie* **1908**, *363*, 107-117.
[53] a) X. Peng, Z. Sun, P. Kuang, L. Li, J. Chen, J. Chen, *Org. Lett.* **2020**, *22*, 5789-5795; b) N. Chatterjee, A. Goswami, *Eur. J. Org. Chem.* **2017**, *2017*, 3023-3032; c) L. D. Caspers, J. Spils, M. Damrath, E. Lork, B. J. Nachtsheim, *J. Org. Chem.* **2020**, *85*, 9161-9178; d) A. Boelke, P. Finkbeiner, B. J. Nachtsheim, *Beilstein J. Org. Chem.* **2018**, *14*, 1263-1280.
[54] G. Szöllösi, I. Kun, M. Bartók, *Chirality* **2001**, *13*, 619-624.
[55] a) Y. Kawamata, J. C. Vantourout, D. P. Hickey, P. Bai, L. Chen, Q. Hou, W. Qiao, K. Barman, M. A. Edwards, A. F. Garrido-Castro, J. N. deGruyter, H. Nakamura, K. Knouse, C. Qin, K. J. Clay, D. Bao, C. Li, J. T. Starr, C. Garcia-Irizarry, N. Sach, H. S. White, M. Neurock, S. D. Minteer, P. S. Baran, *J. Am. Org. Chem.* **2019**, *141*, 6392-6402; b) P. Zardi, A. Caselli, P. Macchi, F. Ferretti, E. Gallo, *Organometallics* **2014**, *33*, 2210-2218.
[56] S. M. King, S. L. Buchwald, *Org. Lett.* **2016**, *18*, 4128-4131.
[57] F. Zhao, Q. Yang, J. Zhang, W. Shi, H. Hu, F. Liang, W. Wei, S. Zhou, *Org. Lett.* **2018**, *20*, 7753-7757.
[58] P. Le Maux, G. Simonneaux, *Tetrahedron* **2015**, *71*, 9333-9338.
[59] N. Jalalian, E. E. Ishikawa, L. F. Silva, B. Olofsson, *Org. Lett.* **2011**, *13*, 1552-1555.
[60] a) T. Ishikawa, E. Uedo, R. Tani, S. Saito, *J. Org. Chem.* **2001**, *66*, 186-191; b) K. K. Sharma, S. Sharma, A. Kudwal, R. Jain, *Org. Biomol. Chem.* **2015**, *13*, 4637-4641.

6 NMR SPECTRA

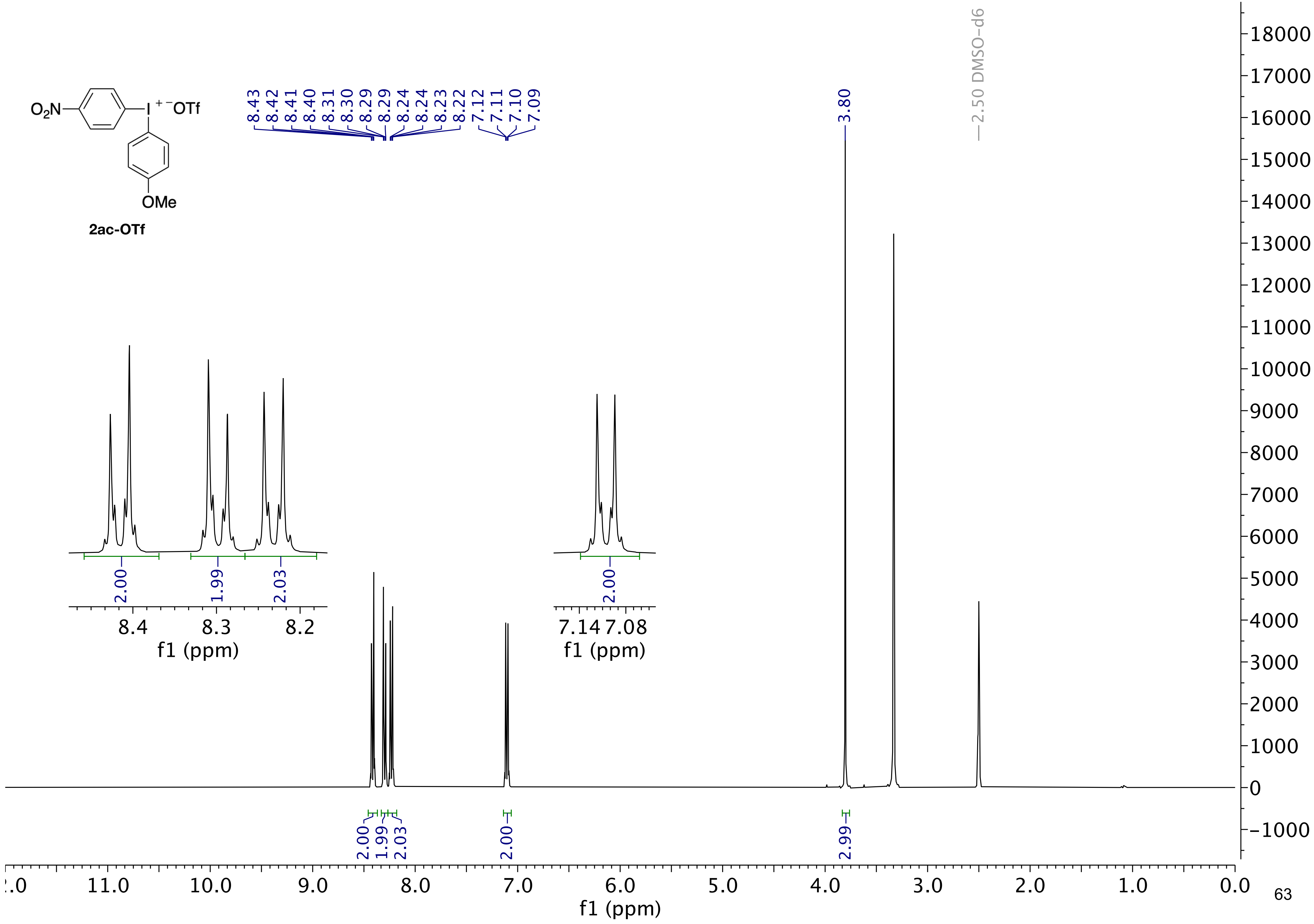
O2N
OTf
OMe
2ac-OTf
8.43
8.42
8.41
8.40
8.31
8.30
8.29
8.29
8.24
8.24
8.23
8.22
7.12
7.11
7.10
7.09
3.80
2.50 DMSO-d6
2.00
1.99
2.03
2.00
2.99
f1 (ppm)

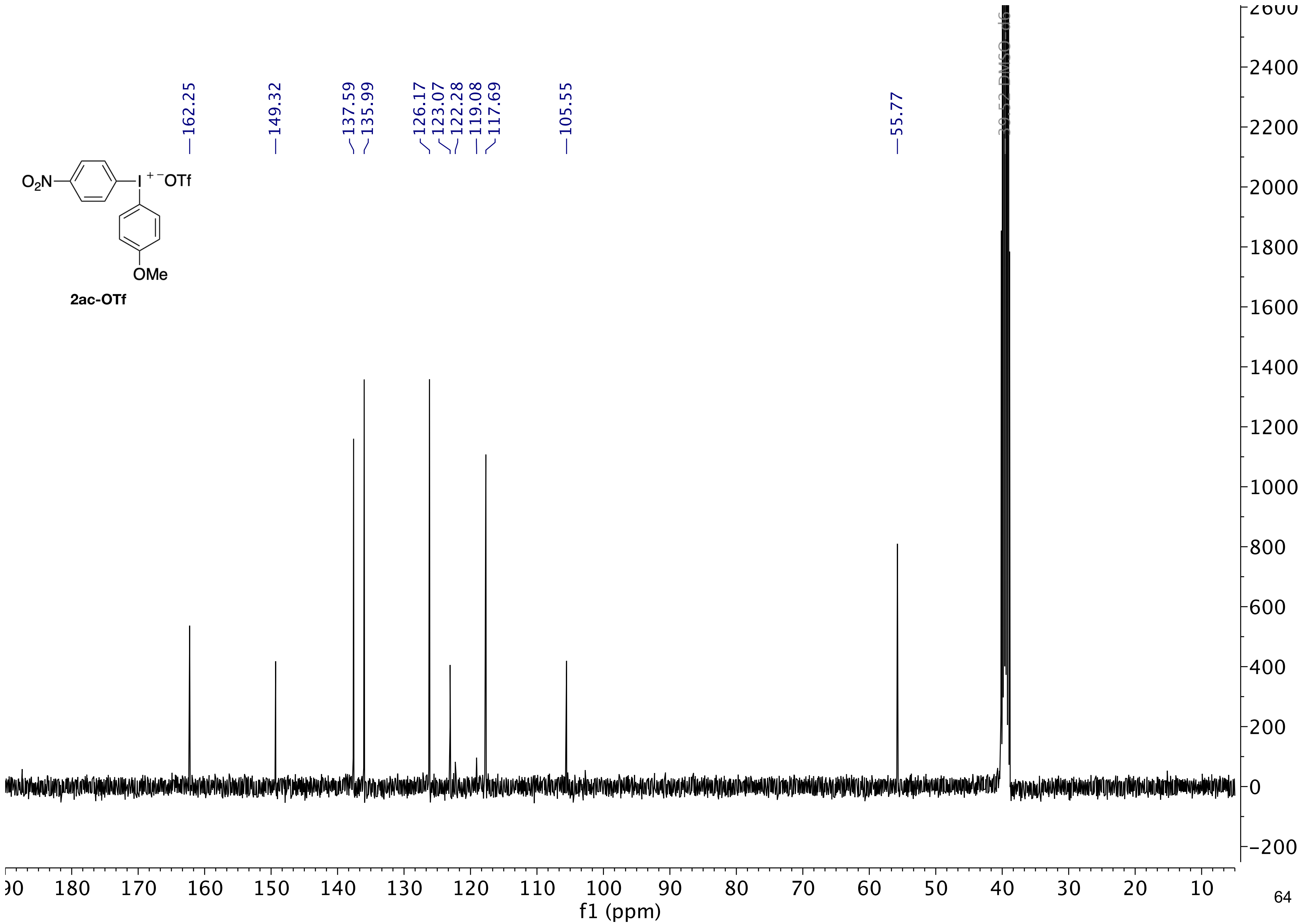
162.25
149.32
137.59
135.99
126.17
123.07
122.28
119.08
117.69
105.55
55.77
39.52 DMSO-d6
O2N
I
+ −OTf
OMe
2ac-OTf
f1 (ppm)

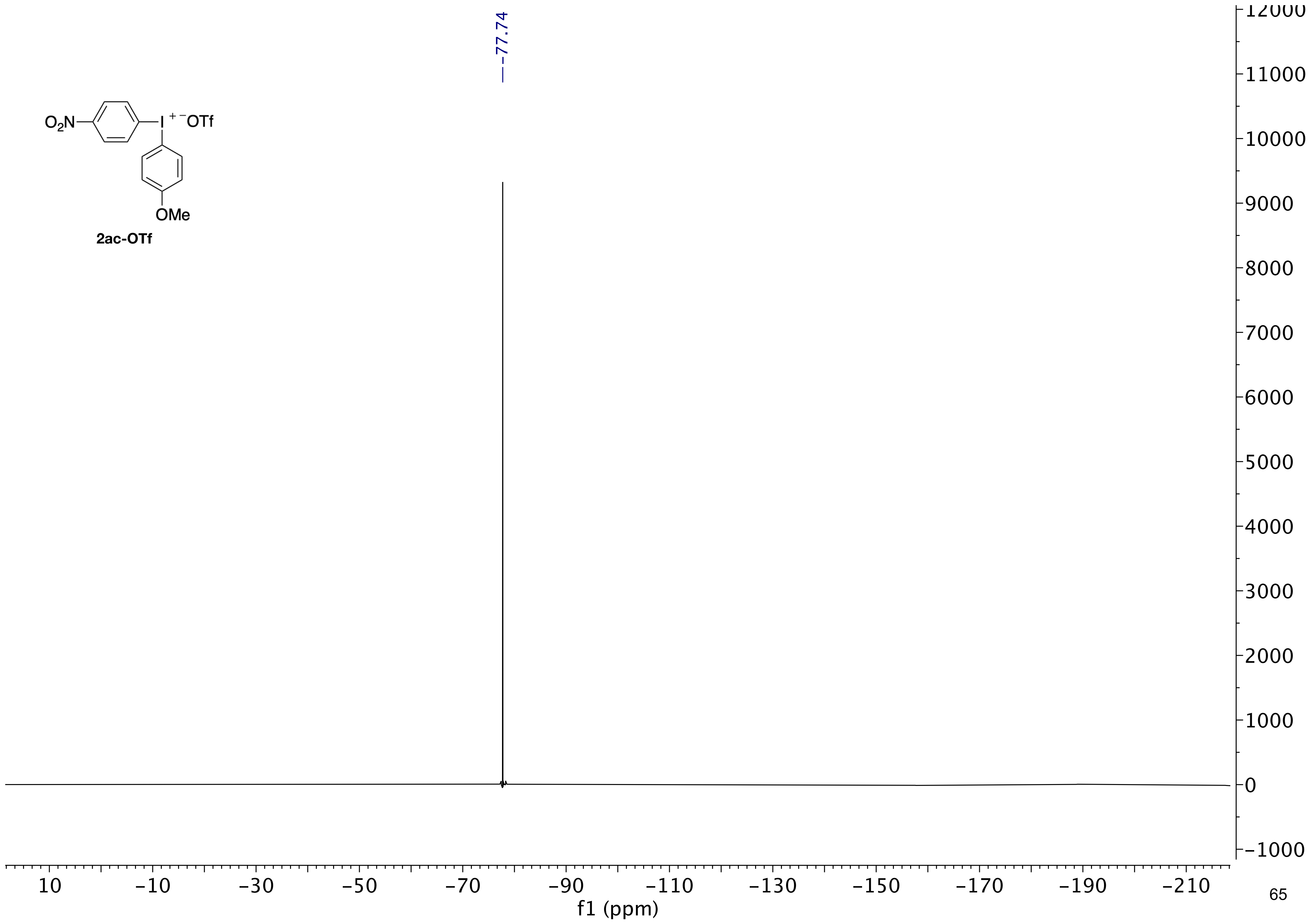

-77.74
O_2N
I^+ ^-OTf
OMe
2ac-OTf
12000
11000
10000
9000
8000
7000
6000
5000
4000
3000
2000
1000
0
−1000
10 −10 −30 −50 −70 −90 −110 −130 −150 −170 −190 −210
f1 (ppm)

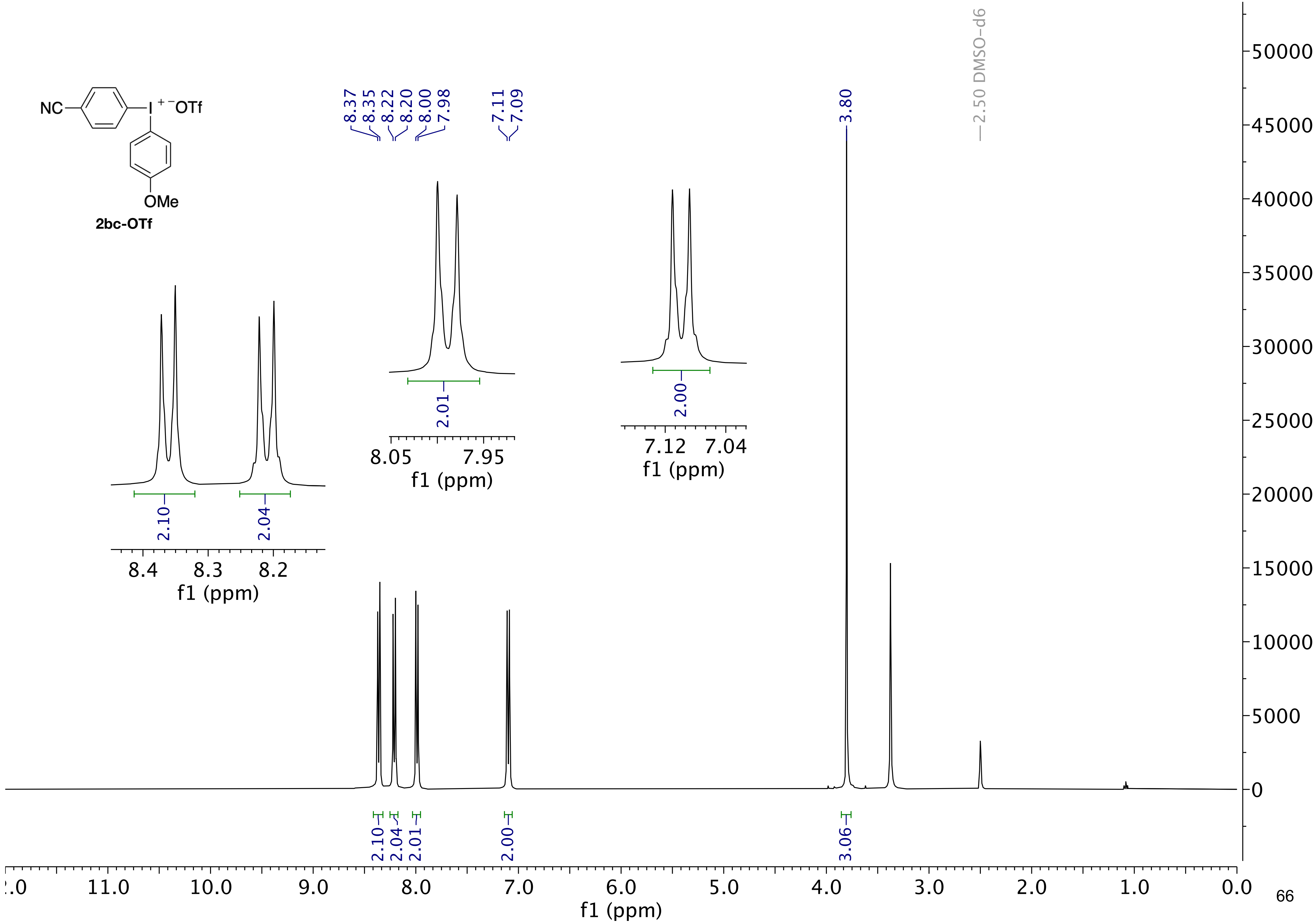
NC
OTf
OMe
2bc-OTf
8.37
8.35
8.22
8.20
8.00
7.98
7.11
7.09
3.80
2.50 DMSO-d6
2.10
2.04
2.01
2.00
3.06
f1 (ppm)

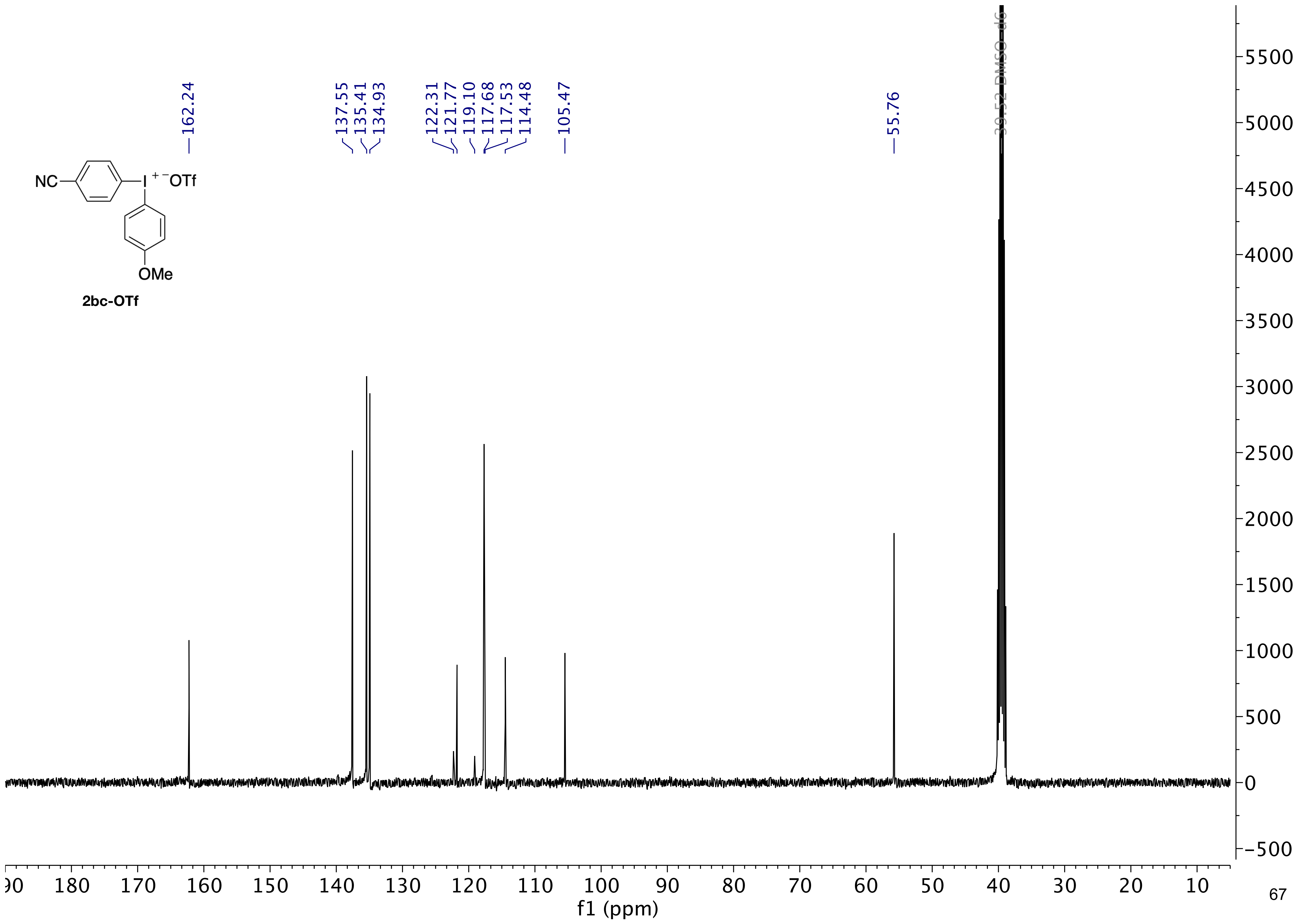

162.24
137.55
135.41
134.93
122.31
121.77
119.10
117.68
117.53
114.48
105.47
55.76
39.52 DMSO-d6
NC
I
+ ⁻OTf
OMe
2bc-OTf
f1 (ppm)

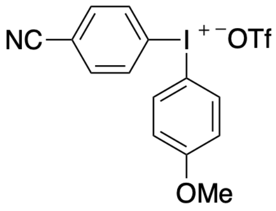

2bc-OTf

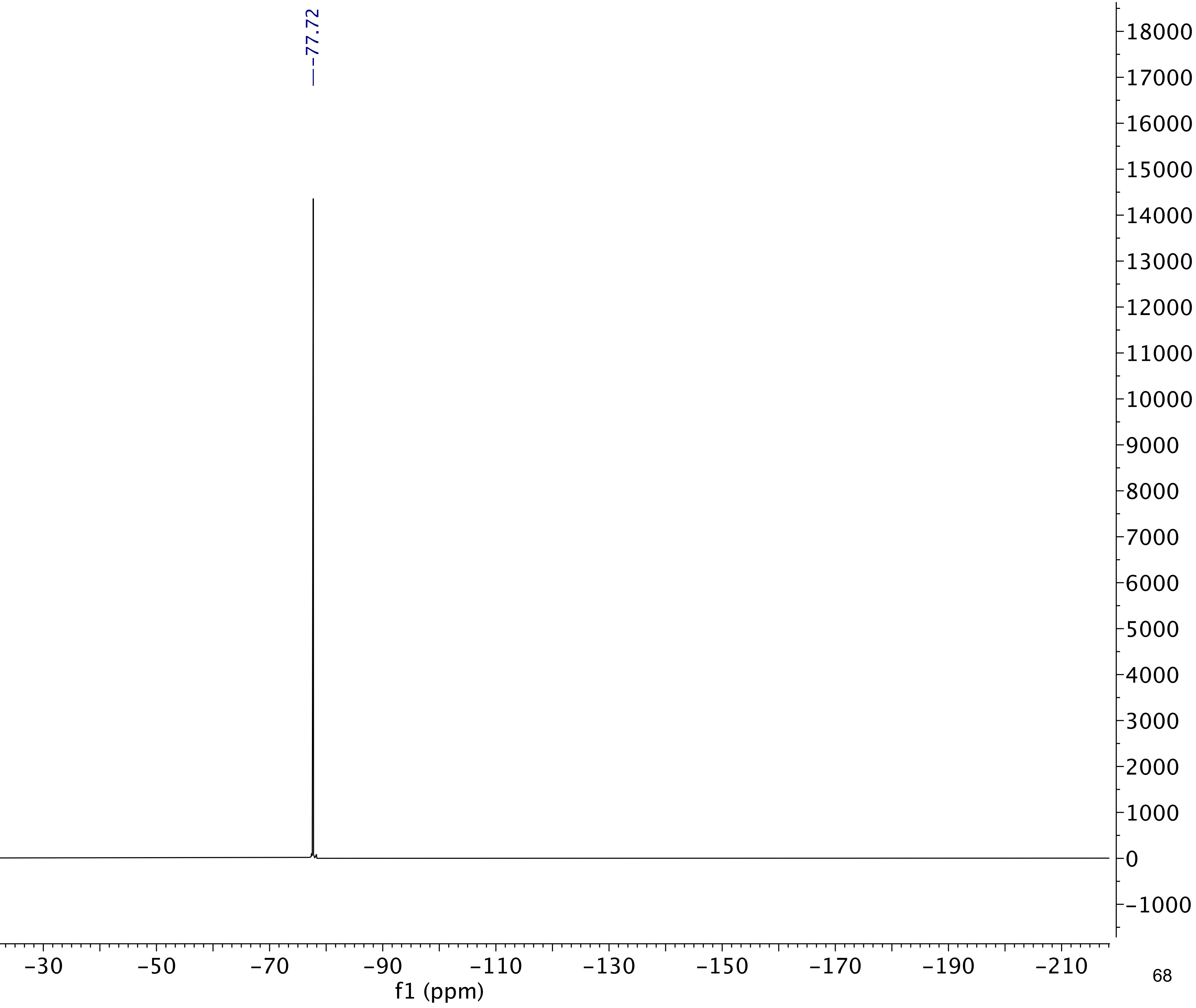

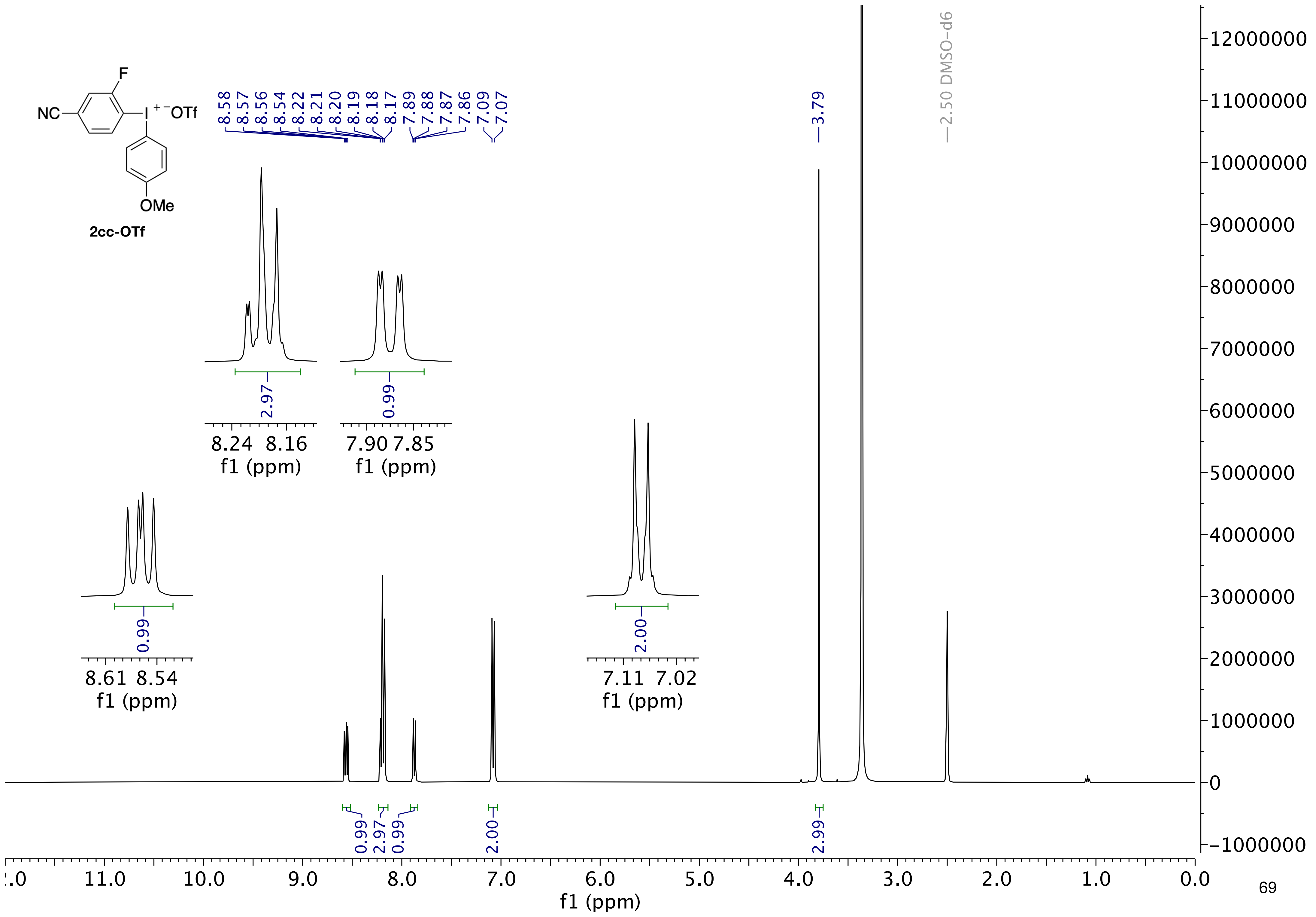

8.58
8.57
8.56
8.54
8.22
8.21
8.20
8.19
8.18
8.17
7.89
7.88
7.87
7.86
7.09
7.07
3.79
2.50 DMSO-d6
2.97
0.99
0.99
2.00
2.99
8.24 8.16
7.90 7.85
8.61 8.54
7.11 7.02
f1 (ppm)

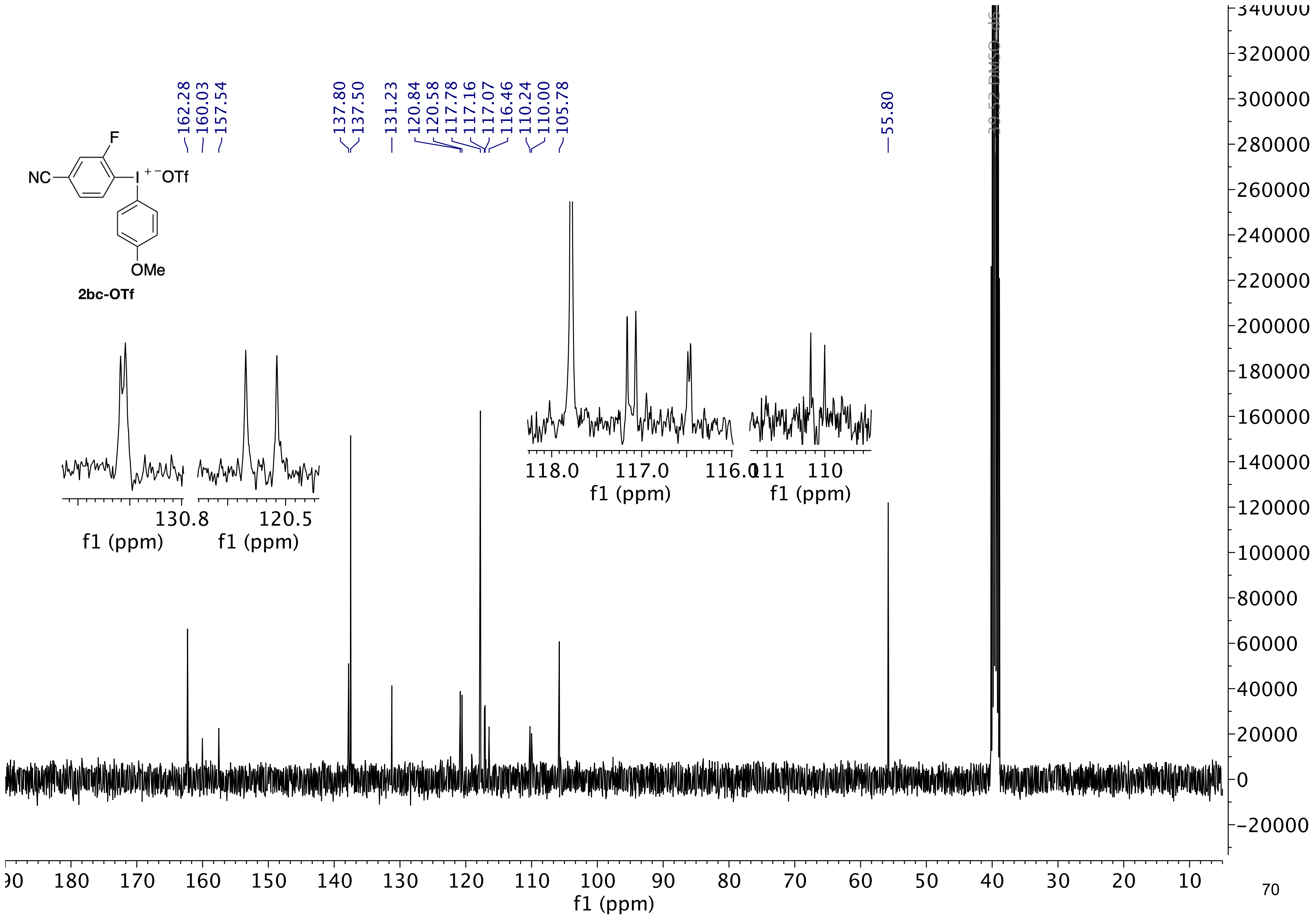
162.28
160.03
157.54
137.80
137.50
131.23
120.84
120.58
117.78
117.16
117.07
116.46
110.24
110.00
105.78
55.80
39.52 DMSO-d6
NC
F
I
+ −OTf
OMe
2bc-OTf
130.8
f1 (ppm)
120.5
f1 (ppm)
118.0
117.0
116.0
f1 (ppm)
111
110
f1 (ppm)
f1 (ppm)

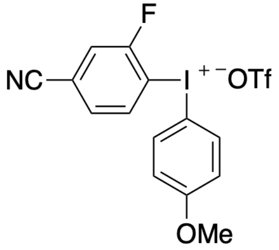
F
NC
I
+ −OTf
OMe

2bc-OTf

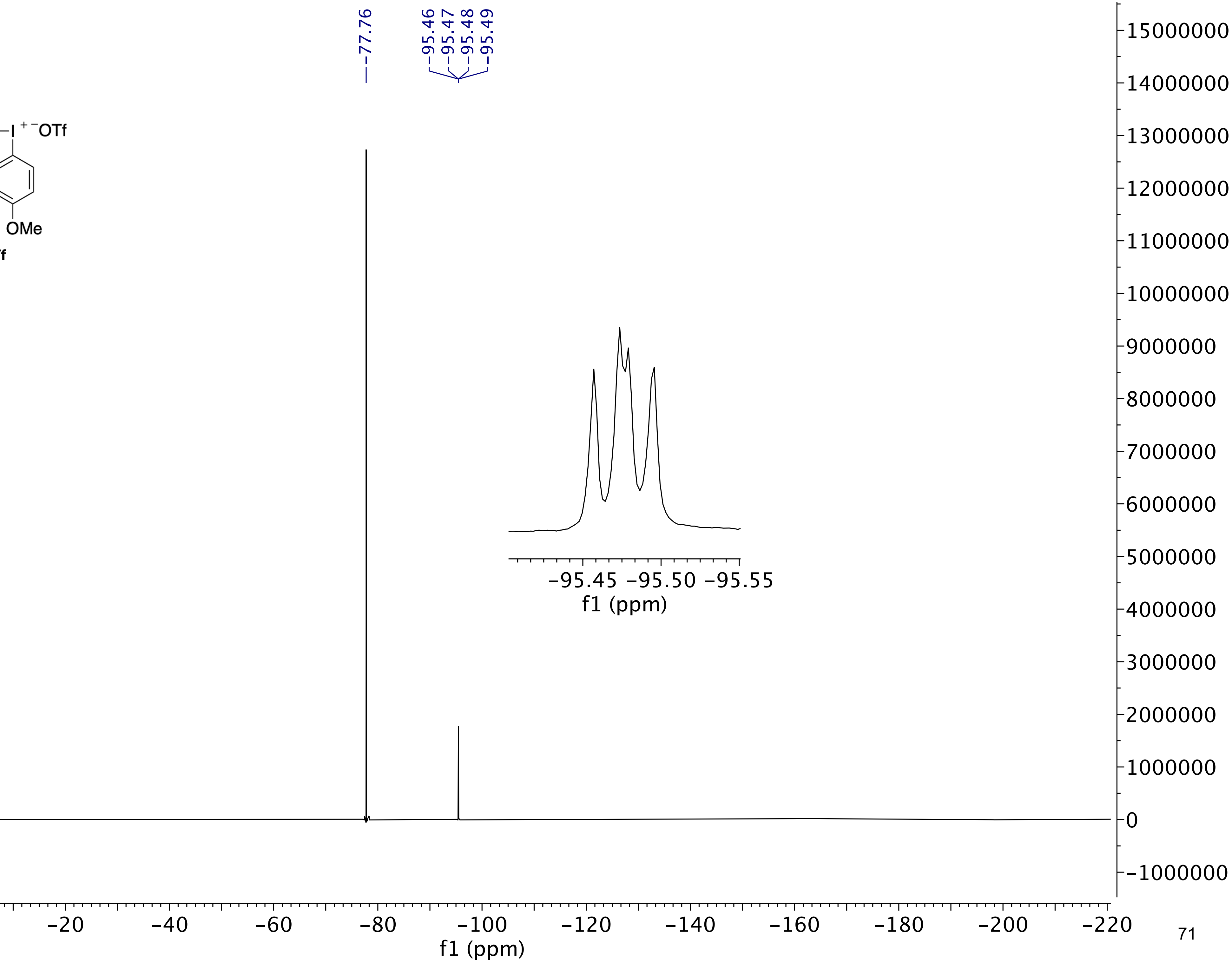
−77.76
−95.46
−95.47
−95.48
−95.49
−95.45 −95.50 −95.55
f1 (ppm)
15000000
14000000
13000000
12000000
11000000
10000000
9000000
8000000
7000000
6000000
5000000
4000000
3000000
2000000
1000000
0
−1000000
0 −20 −40 −60 −80 −100 −120 −140 −160 −180 −200 −220
f1 (ppm)

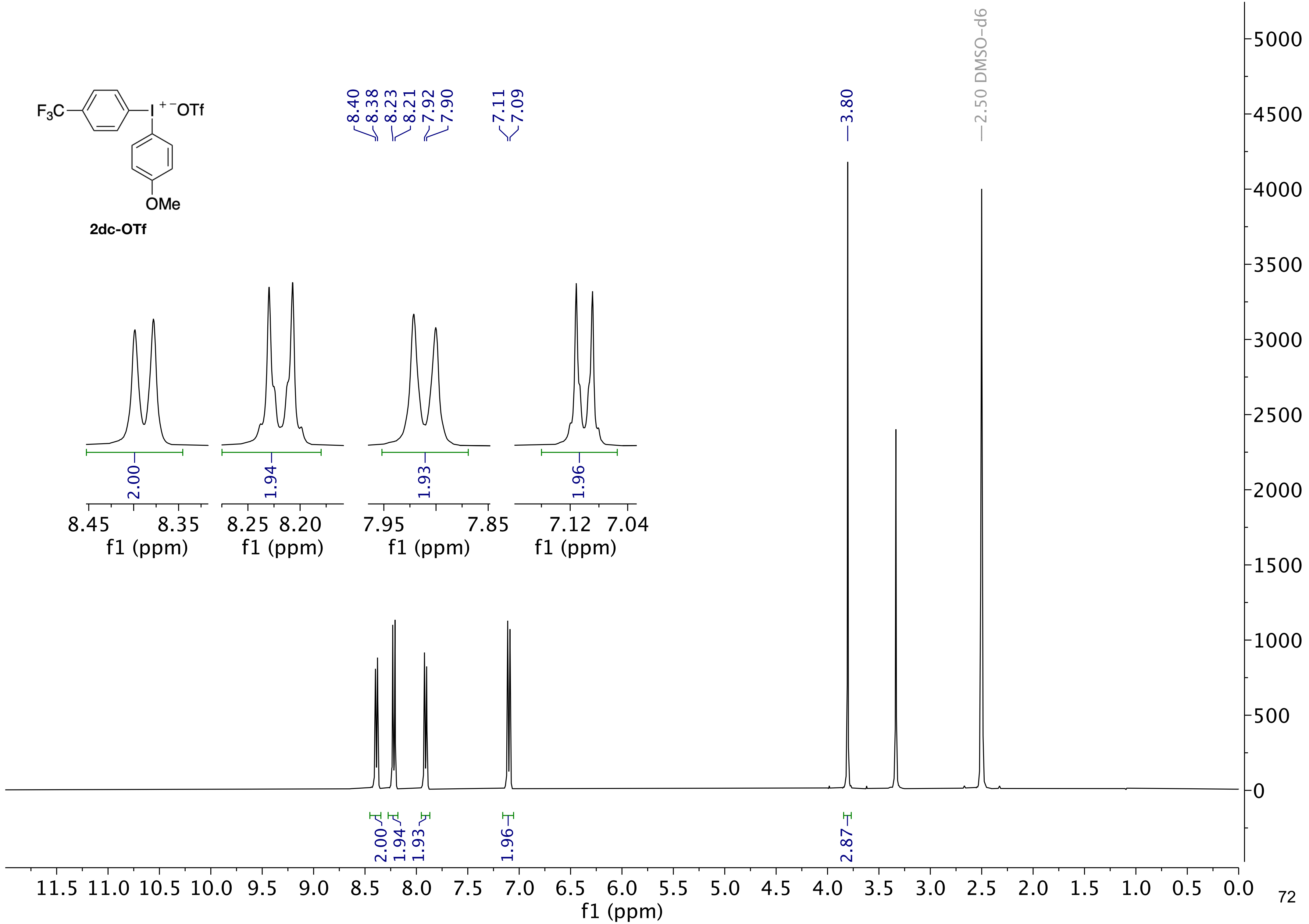

2dc-OTf
8.40
8.38
8.23
8.21
7.92
7.90
7.11
7.09
3.80
2.50 DMSO-d6
2.00
1.94
1.93
1.96
2.87
f1 (ppm)

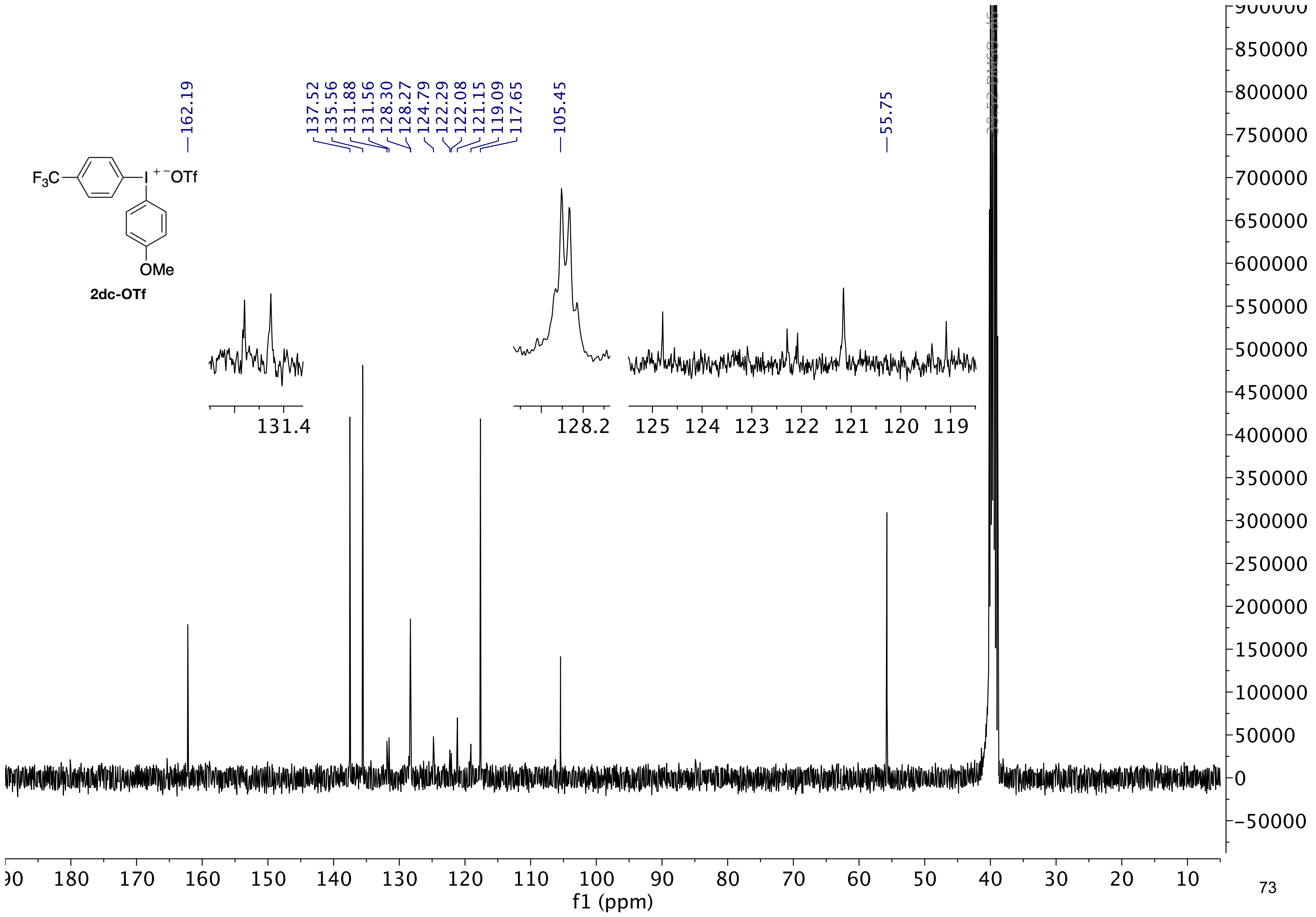

F3C
I
OTf
OMe
2dc-OTf
162.19
137.52
135.56
131.88
131.56
128.30
128.27
124.79
122.29
122.08
121.15
119.09
117.65
105.45
55.75
39.52 DMSO-d6
131.4
128.2
125 124 123 122 121 120 119
f1 (ppm)

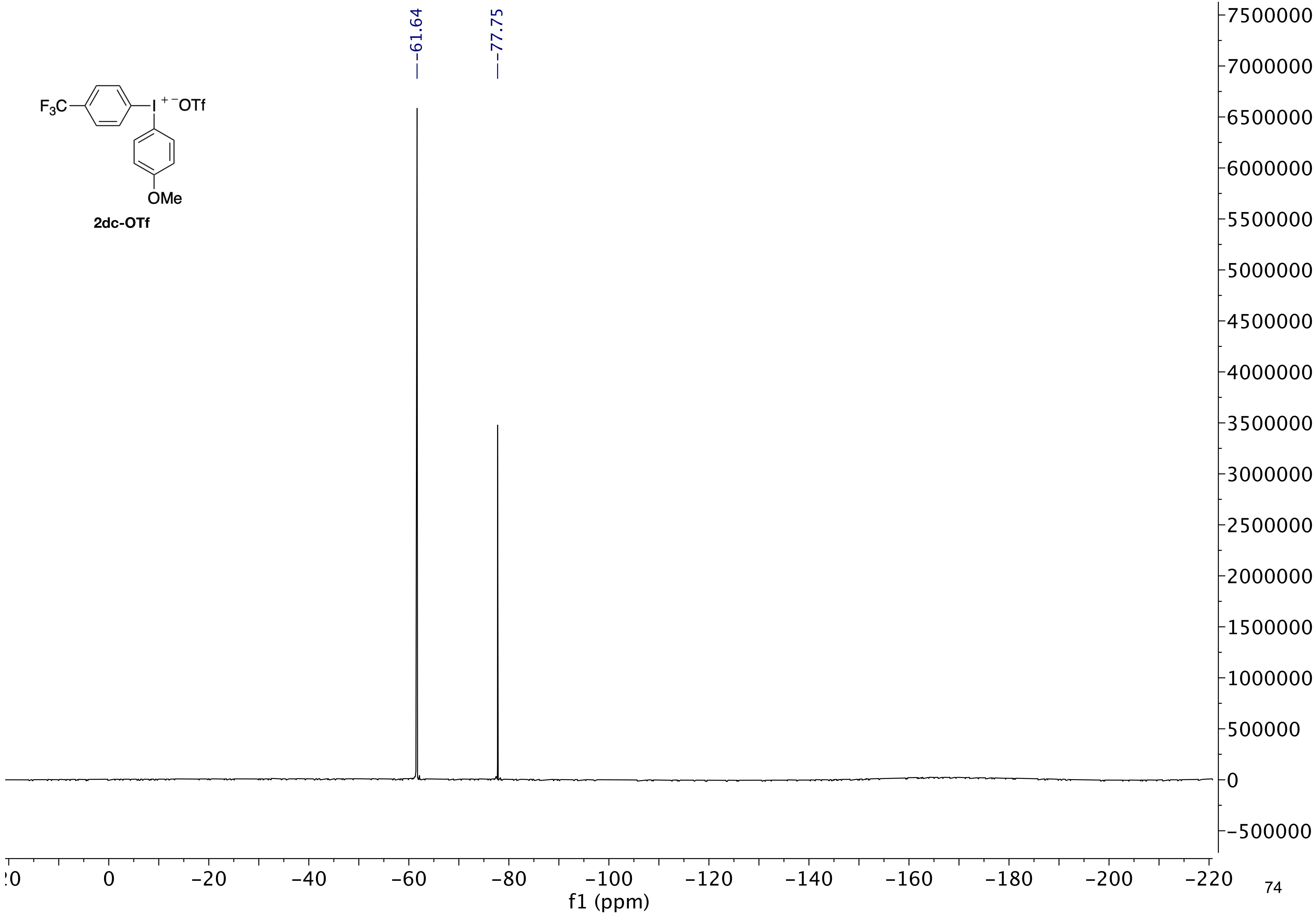

−61.64
−77.75
F3C
I+ −OTf
OMe
2dc-OTf
7500000
7000000
6500000
6000000
5500000
5000000
4500000
4000000
3500000
3000000
2500000
2000000
1500000
1000000
500000
0
−500000
0
−20
−40
−60
−80
−100
−120
−140
−160
−180
−200
−220
f1 (ppm)

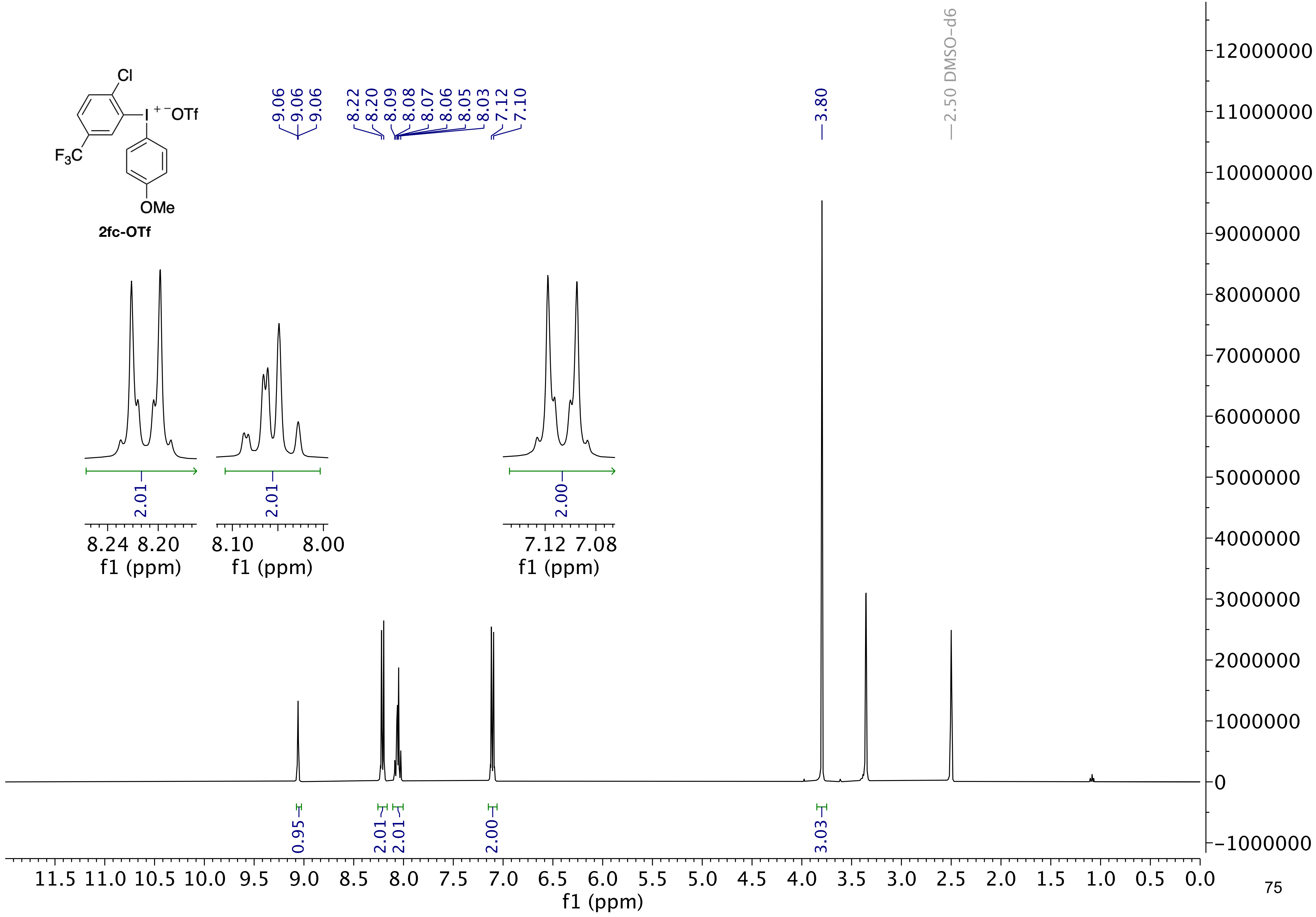

Cl
I+ −OTf
F3C
OMe
2fc-OTf
9.06 9.06 9.06
8.22 8.20 8.09 8.08 8.07 8.06 8.05 8.03 7.12 7.10
3.80
2.50 DMSO-d6
2.01
2.01
2.00
8.24 8.20
8.10 8.00
7.12 7.08
f1 (ppm)
0.95
2.01
2.01
2.00
3.03
12000000
11000000
10000000
9000000
8000000
7000000
6000000
5000000
4000000
3000000
2000000
1000000
0
−1000000
11.5 11.0 10.5 10.0 9.5 9.0 8.5 8.0 7.5 7.0 6.5 6.0 5.5 5.0 4.5 4.0 3.5 3.0 2.5 2.0 1.5 1.0 0.5 0.0
f1 (ppm)

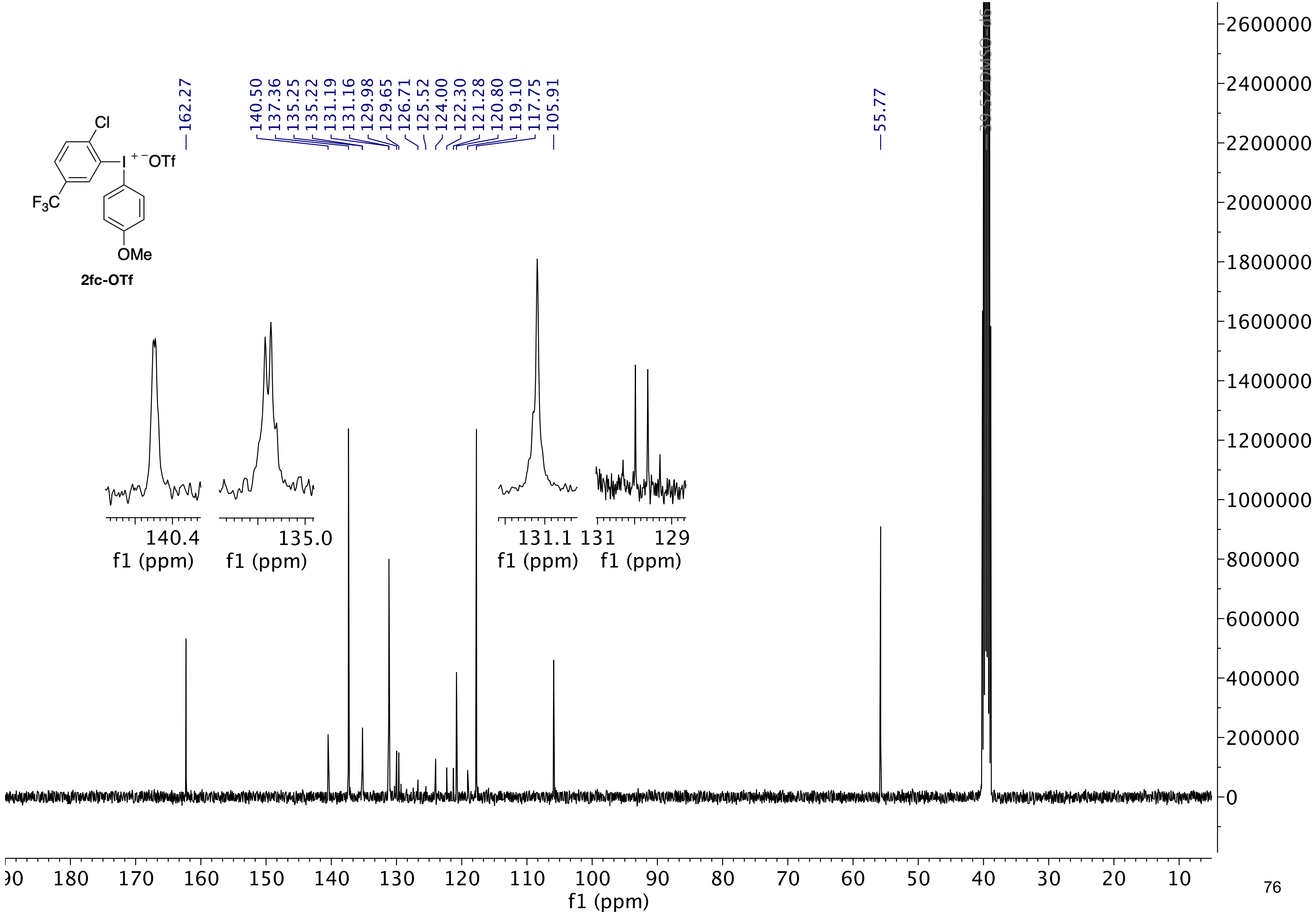
Cl
OTf
F3C
OMe
2fc-OTf
162.27
140.50
137.36
135.25
135.22
131.19
131.16
129.98
129.65
126.71
125.52
124.00
122.30
121.28
120.80
119.10
117.75
105.91
55.77
39.52 DMSO-d6
140.4
135.0
131.1
131
129
f1 (ppm)
2600000
2400000
2200000
2000000
1800000
1600000
1400000
1200000
1000000
800000
600000
400000
200000
0
90 180 170 160 150 140 130 120 110 100 90 80 70 60 50 40 30 20 10

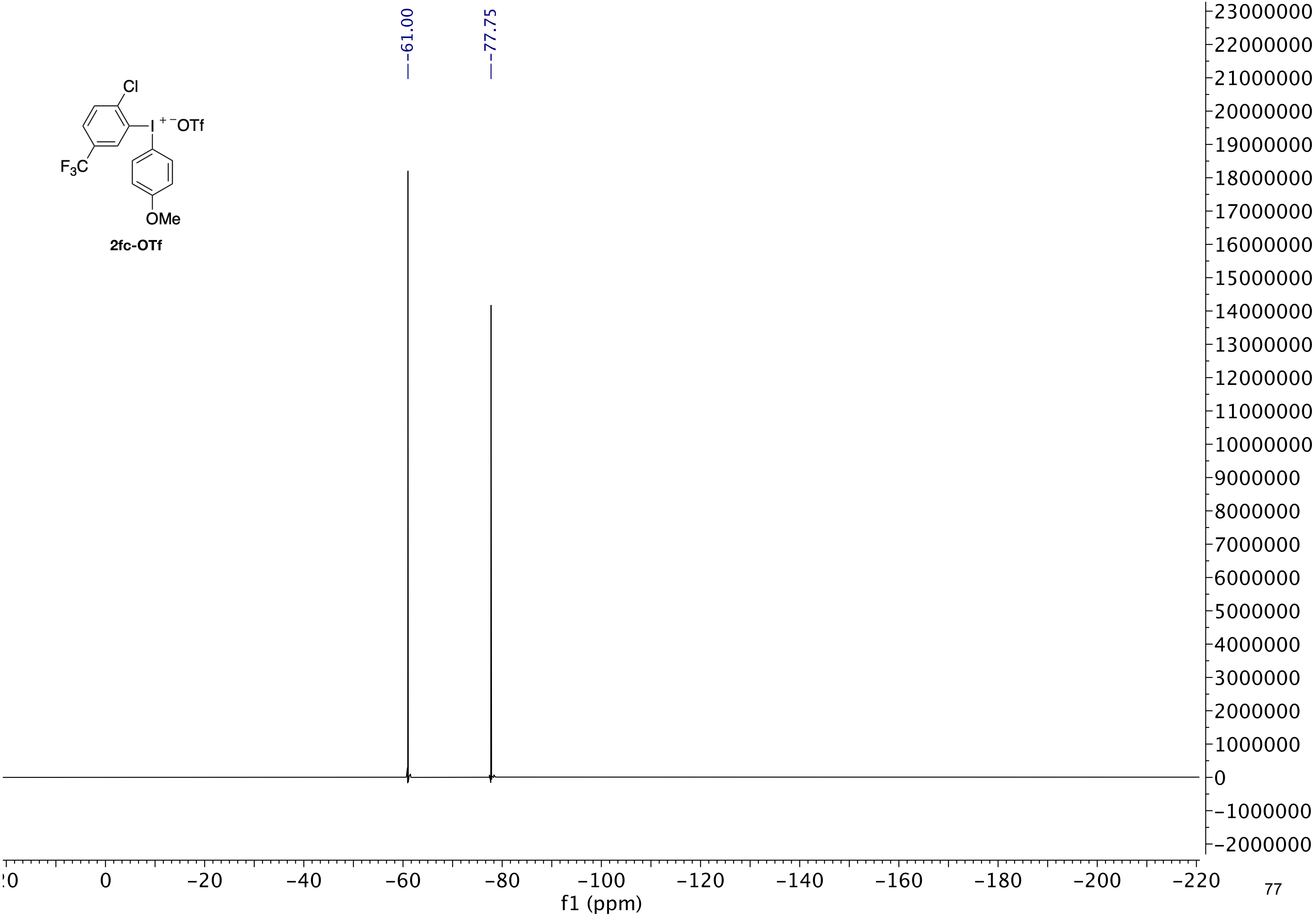
-61.00
-77.75
Cl
I
+ −OTf
F3C
OMe
2fc-OTf
23000000
22000000
21000000
20000000
19000000
18000000
17000000
16000000
15000000
14000000
13000000
12000000
11000000
10000000
9000000
8000000
7000000
6000000
5000000
4000000
3000000
2000000
1000000
0
−1000000
−2000000
0
−20
−40
−60
−80
−100
−120
−140
−160
−180
−200
−220
f1 (ppm)

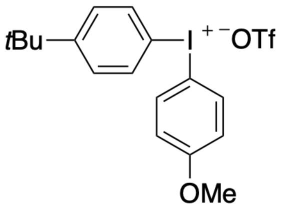

2jc-OTf

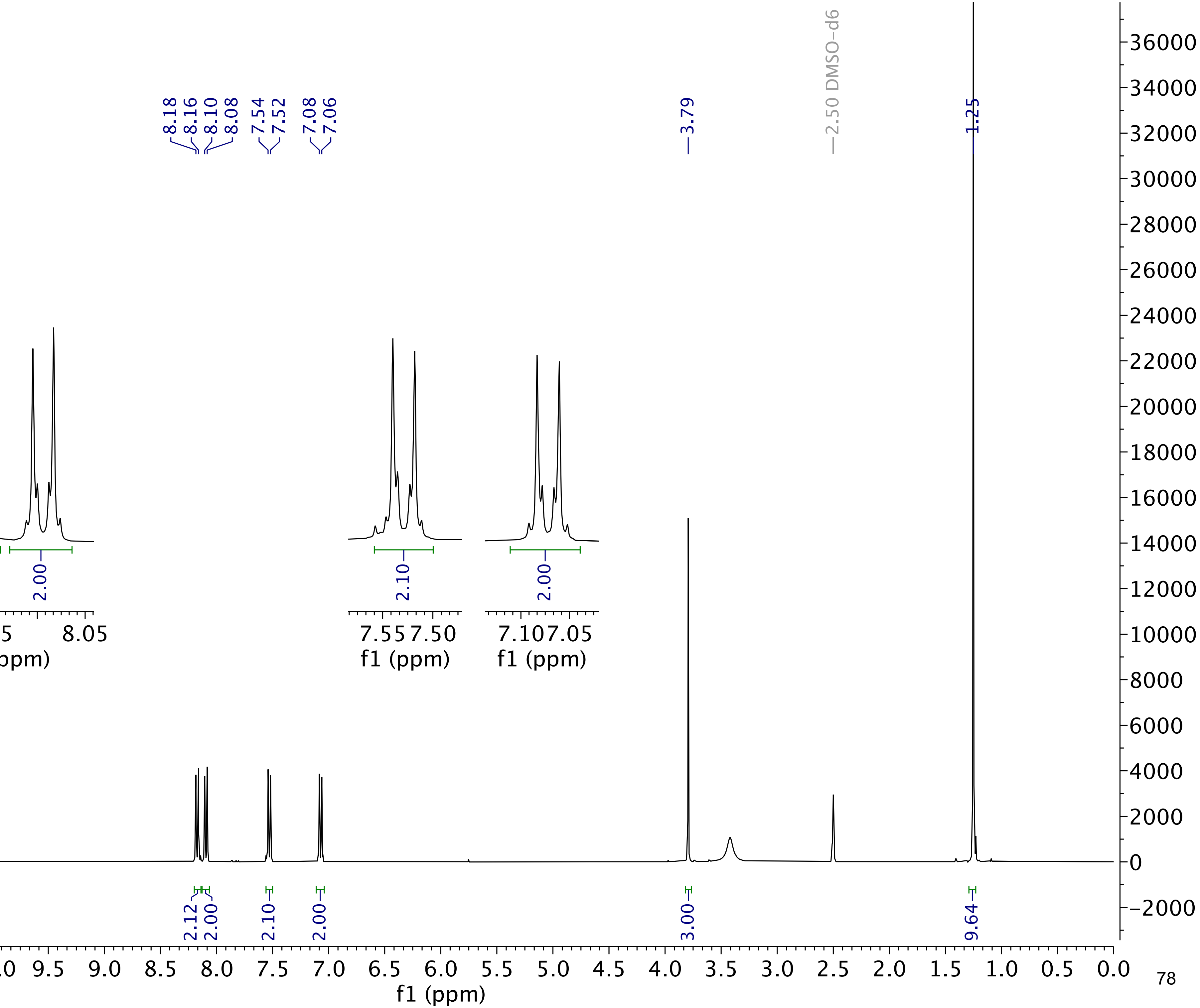

8.18
8.16
8.10
8.08
7.54
7.52
7.08
7.06
3.79
2.50 DMSO-d6
1.25
2.12
2.00
2.10
2.00
8.15
8.05
f1 (ppm)
7.55
7.50
7.10
7.05
3.00
9.64
36000
34000
32000
30000
28000
26000
24000
22000
20000
18000
16000
14000
12000
10000
8000
6000
4000
2000
0
−2000
11.5 11.0 10.5 10.0 9.5 9.0 8.5 8.0 7.5 7.0 6.5 6.0 5.5 5.0 4.5 4.0 3.5 3.0 2.5 2.0 1.5 1.0 0.5 0.0

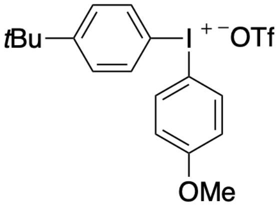
tBu
I
+ −OTf
OMe

2jc-OTf

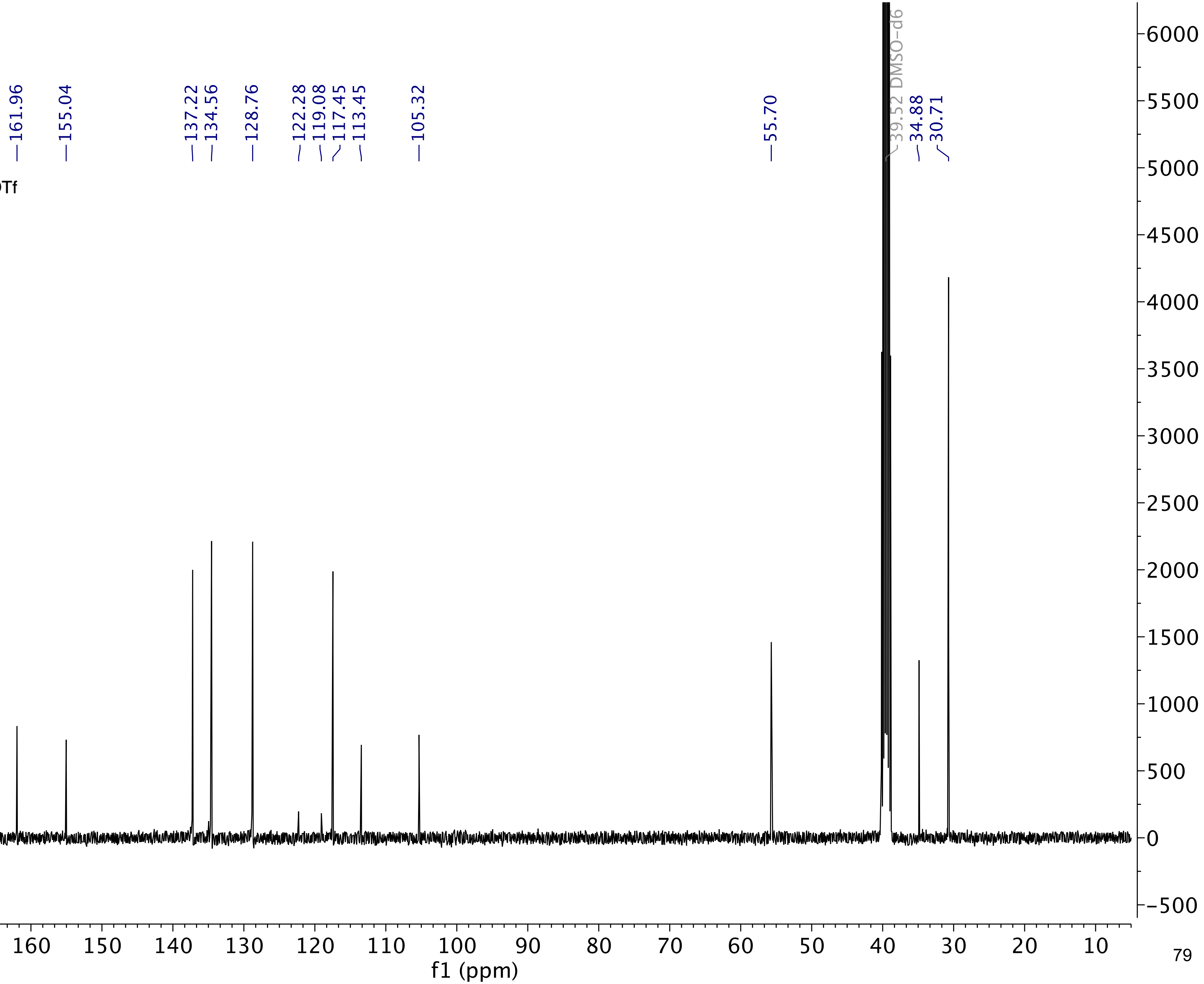
161.96
155.04
137.22
134.56
128.76
122.28
119.08
117.45
113.45
105.32
55.70
39.52 DMSO-d6
34.88
30.71
f1 (ppm)

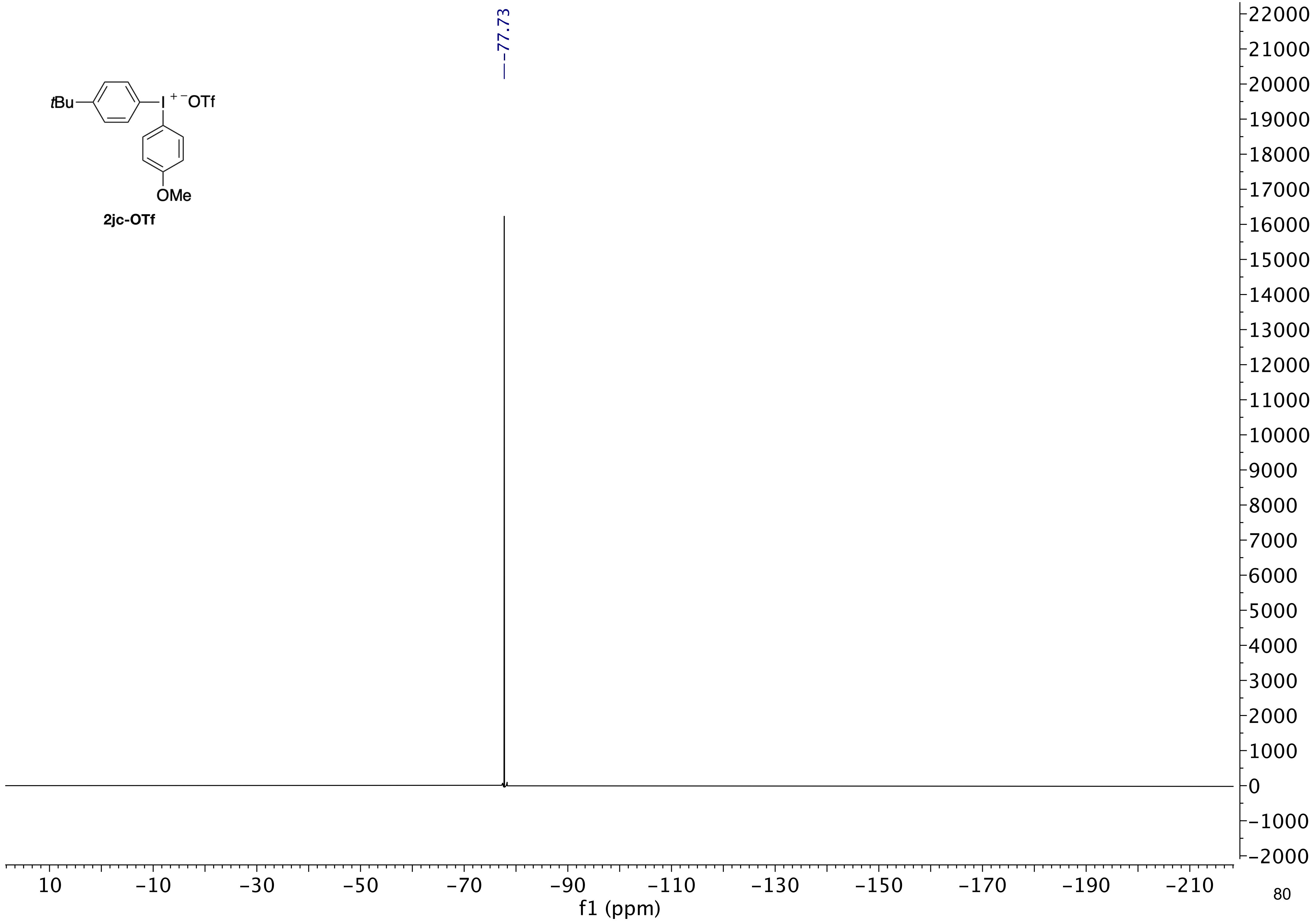

-77.73
tBu
I
+ −OTf
OMe
2jc-OTf
22000
21000
20000
19000
18000
17000
16000
15000
14000
13000
12000
11000
10000
9000
8000
7000
6000
5000
4000
3000
2000
1000
0
−1000
−2000
10
−10
−30
−50
−70
−90
−110
−130
−150
−170
−190
−210
f1 (ppm)

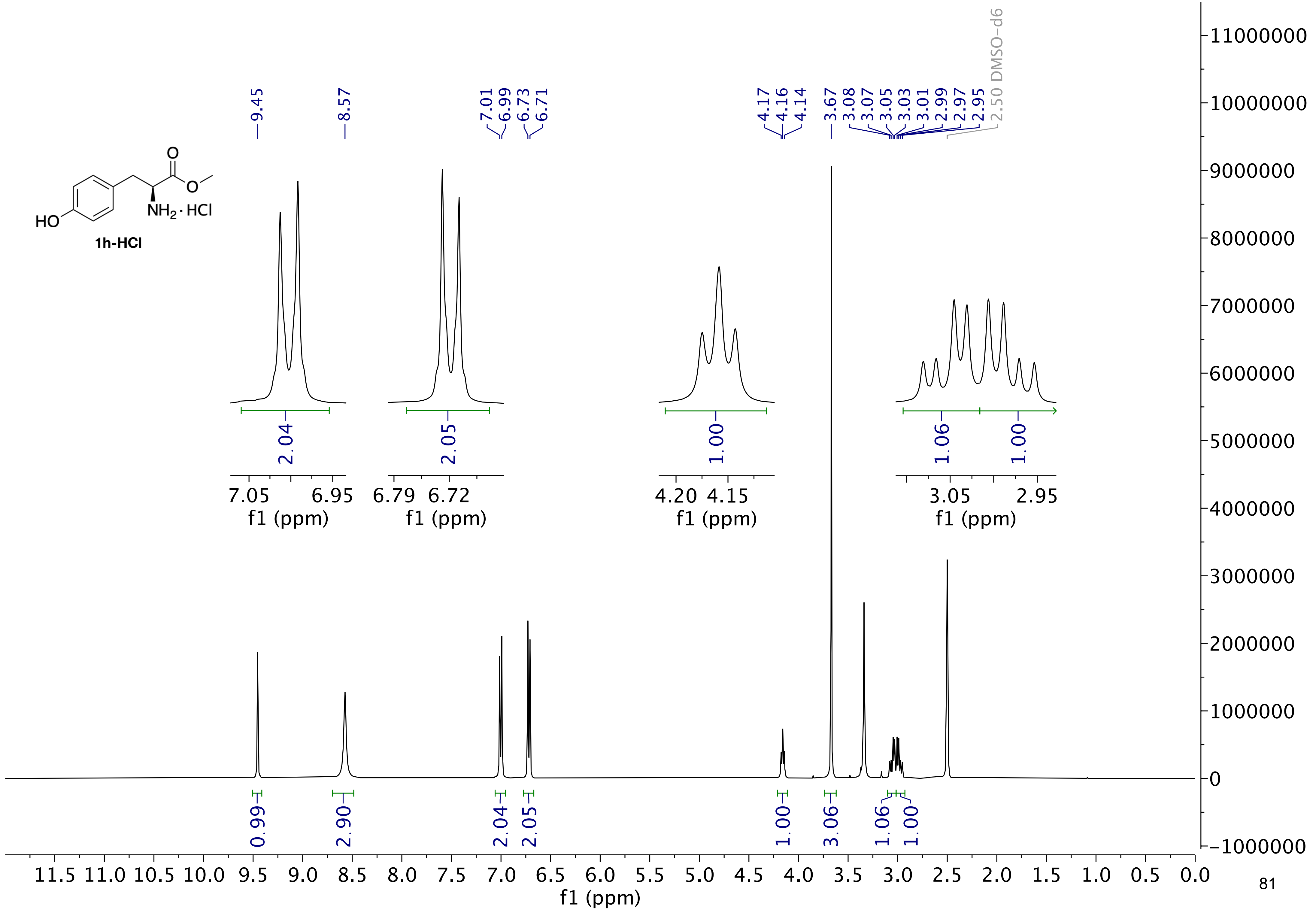

HO
O
O
$NH_2 \cdot HCl$
1h-HCl
9.45
8.57
7.01
6.99
6.73
6.71
4.17
4.16
4.14
3.67
3.08
3.07
3.05
3.03
3.01
2.99
2.97
2.95
2.50 DMSO-d6
2.04
2.05
1.00
1.06
1.00
7.05
6.95
6.79
6.72
4.20
4.15
3.05
2.95
f1 (ppm)
0.99
2.90
2.04
2.05
1.00
3.06
1.06
1.00
11.5 11.0 10.5 10.0 9.5 9.0 8.5 8.0 7.5 7.0 6.5 6.0 5.5 5.0 4.5 4.0 3.5 3.0 2.5 2.0 1.5 1.0 0.5 0.0
f1 (ppm)
11000000
10000000
9000000
8000000
7000000
6000000
5000000
4000000
3000000
2000000
1000000
0
-1000000

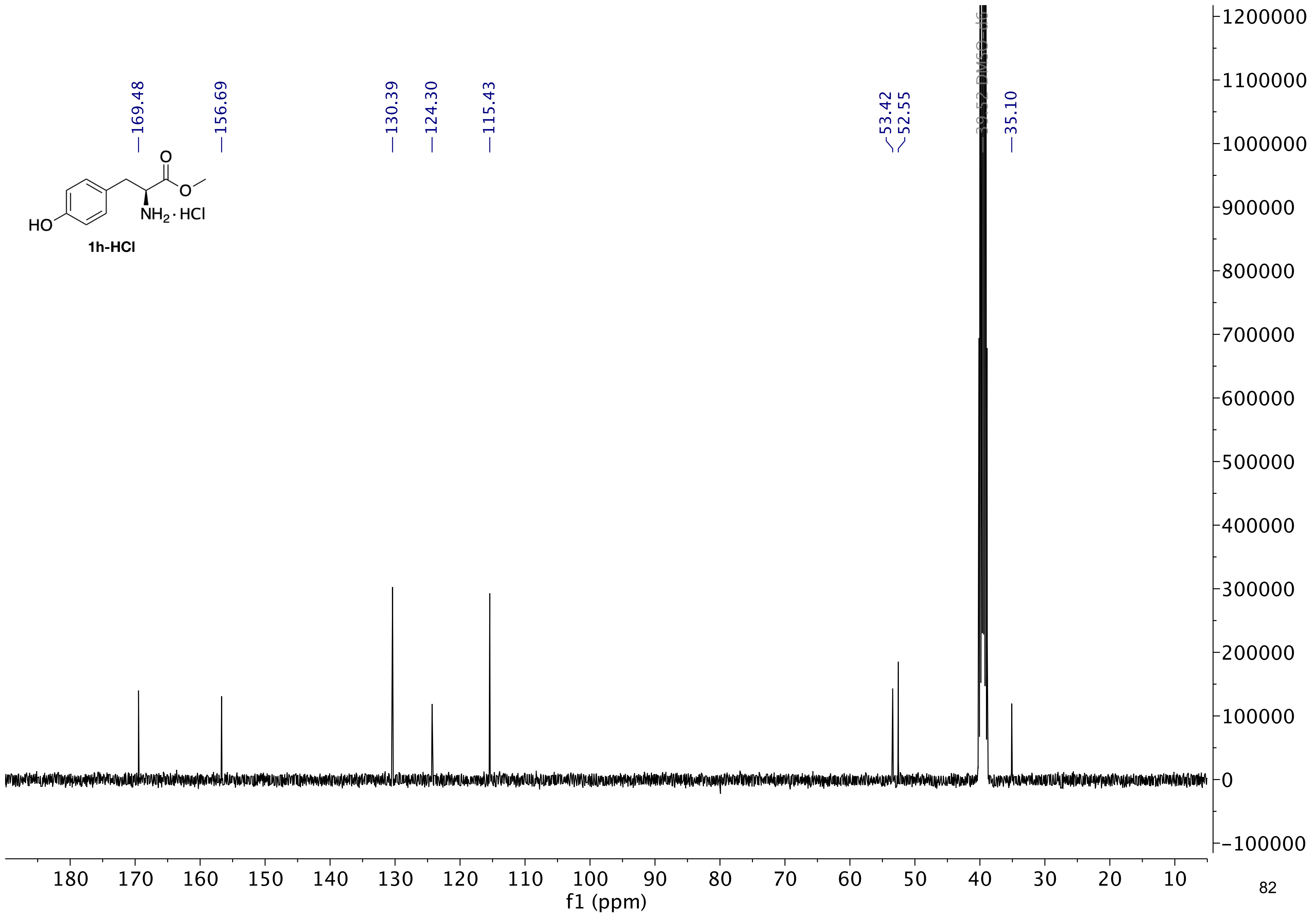

169.48
156.69
130.39
124.30
115.43
53.42
52.55
39.52 DMSO-d6
35.10
O
O
HO
NH2 · HCl
1h-HCl
f1 (ppm)
180
170
160
150
140
130
120
110
100
90
80
70
60
50
40
30
20
10
1200000
1100000
1000000
900000
800000
700000
600000
500000
400000
300000
200000
100000
0
−100000

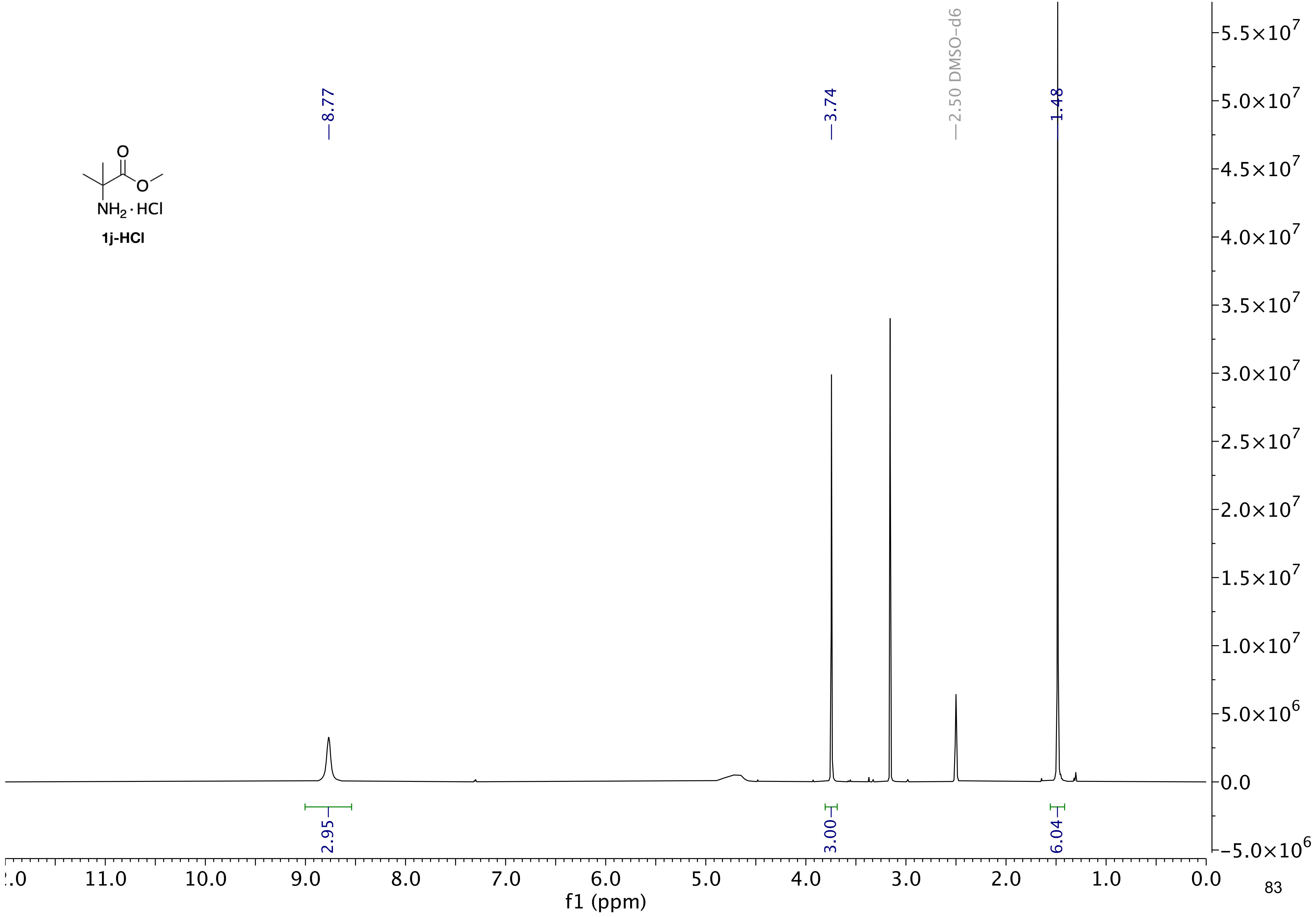

1j-HCl
NH2 · HCl
8.77
3.74
2.50 DMSO-d6
1.48
2.95
3.00
6.04
f1 (ppm)

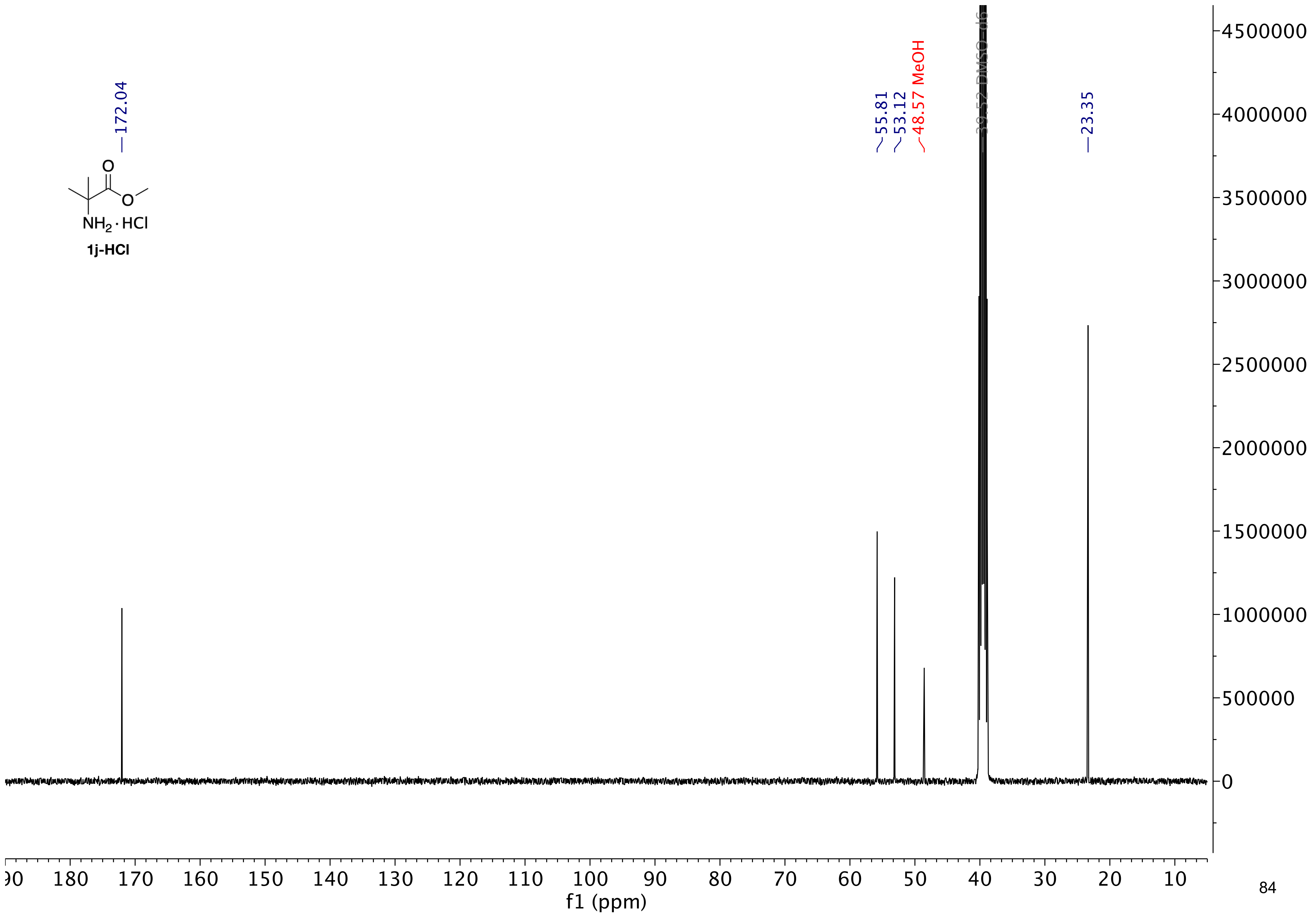

172.04
55.81
53.12
48.57 MeOH
39.52 DMSO-d6
23.35
O
O
NH2 · HCl
4500000
4000000
3500000
3000000
2500000
2000000
1500000
1000000
500000
0
180
170
160
150
140
130
120
110
100
90
80
70
60
50
40
30
20
10
f1 (ppm)

1j-HCl

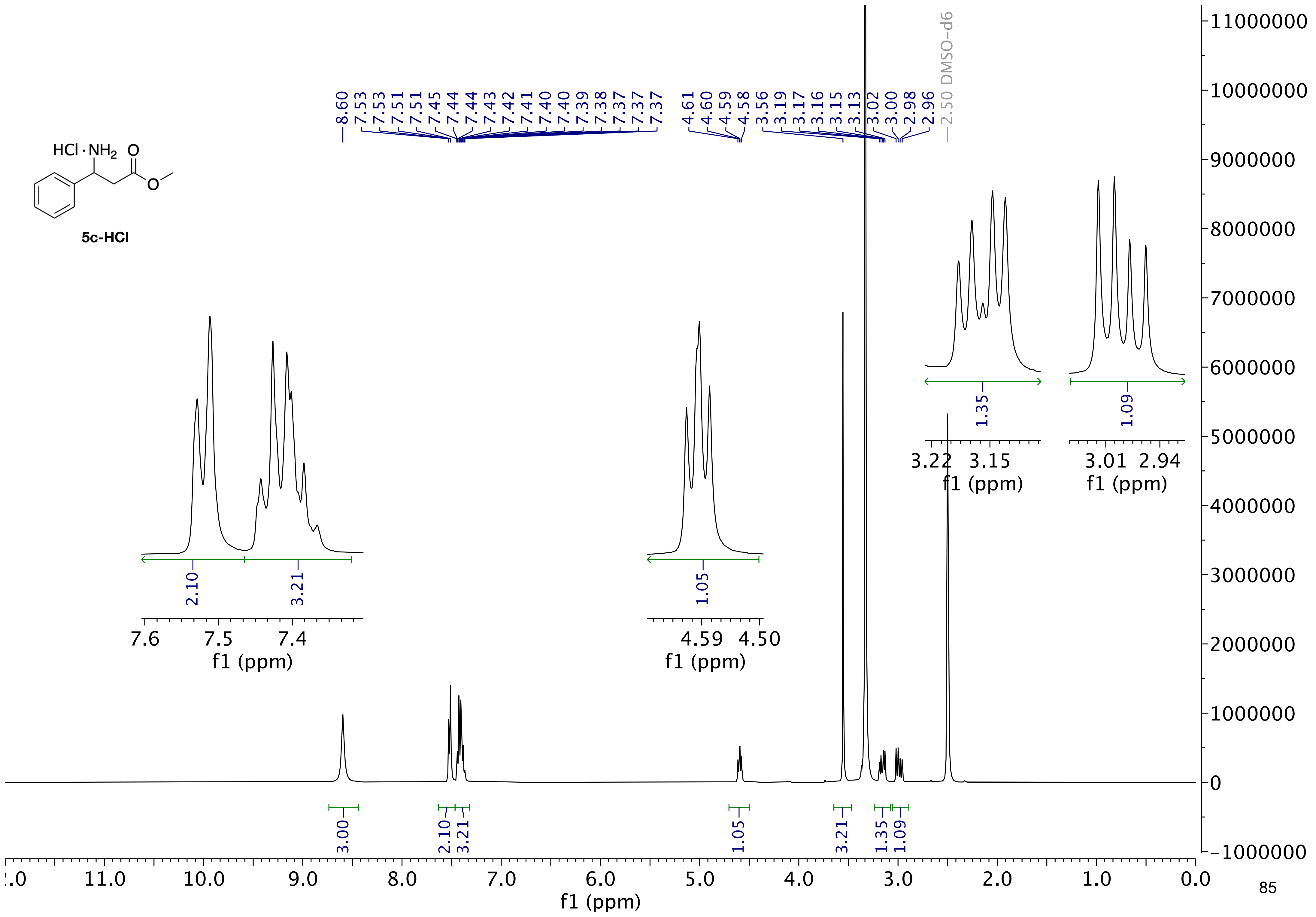
HCl · NH_2 O
8.60 7.53 7.53 7.51 7.51 7.45 7.44 7.44 7.43 7.42 7.41 7.40 7.40 7.39 7.38 7.37 7.37 7.37 4.61 4.60 4.59 4.58 3.56 3.19 3.17 3.16 3.15 3.13 3.02 3.00 2.98 2.96
2.50 DMSO-d6
2.10 3.21 1.05 1.35 1.09
3.00 2.10 3.21 1.05 3.21 1.35 1.09
f1 (ppm)

5c-HCl

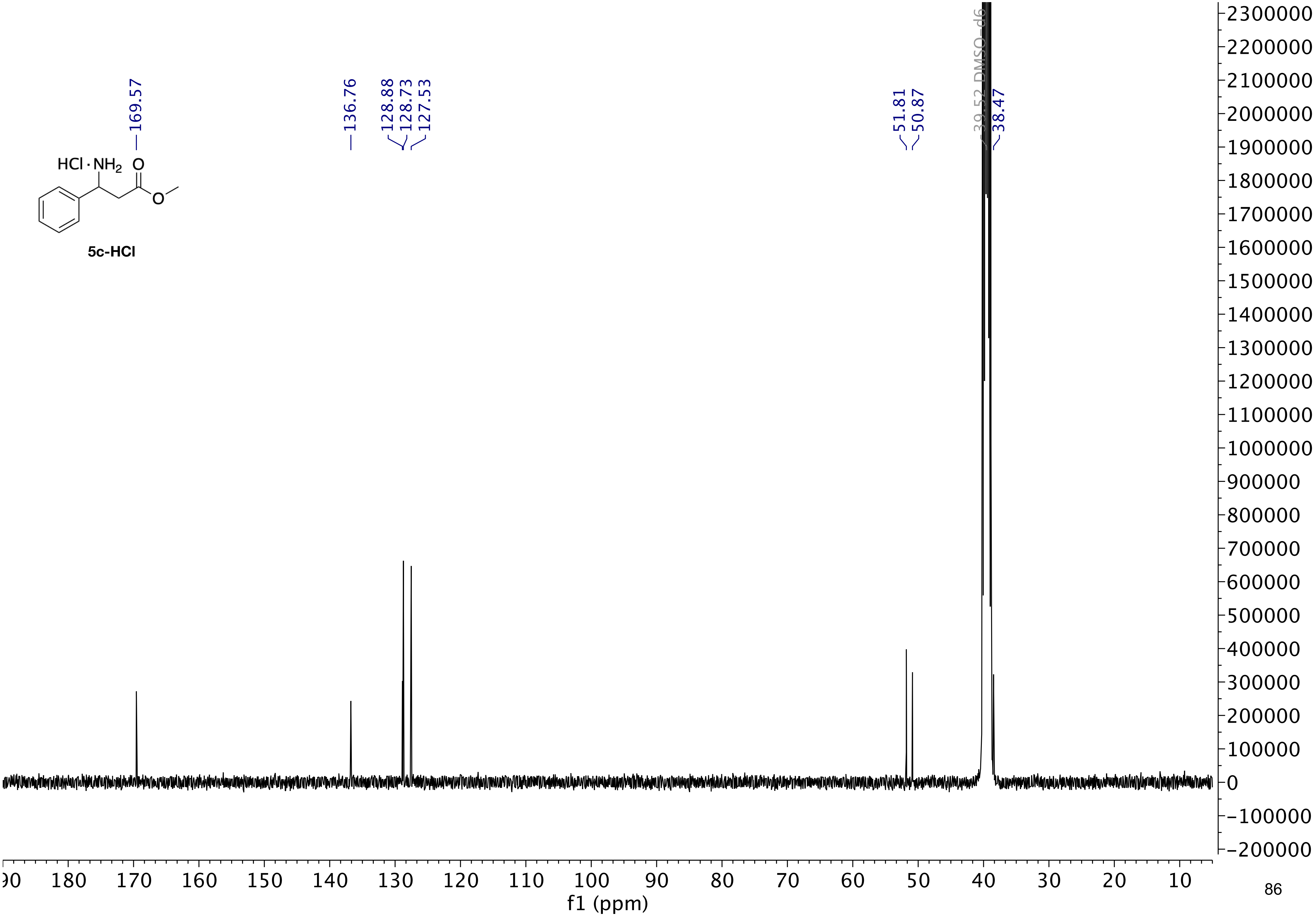

HCl · NH2 O
O
5c-HCl
169.57
136.76
128.88
128.73
127.53
51.81
50.87
39.52 DMSO-d6
38.47
2300000
2200000
2100000
2000000
1900000
1800000
1700000
1600000
1500000
1400000
1300000
1200000
1100000
1000000
900000
800000
700000
600000
500000
400000
300000
200000
100000
0
−100000
−200000
180 170 160 150 140 130 120 110 100 90 80 70 60 50 40 30 20 10
f1 (ppm)

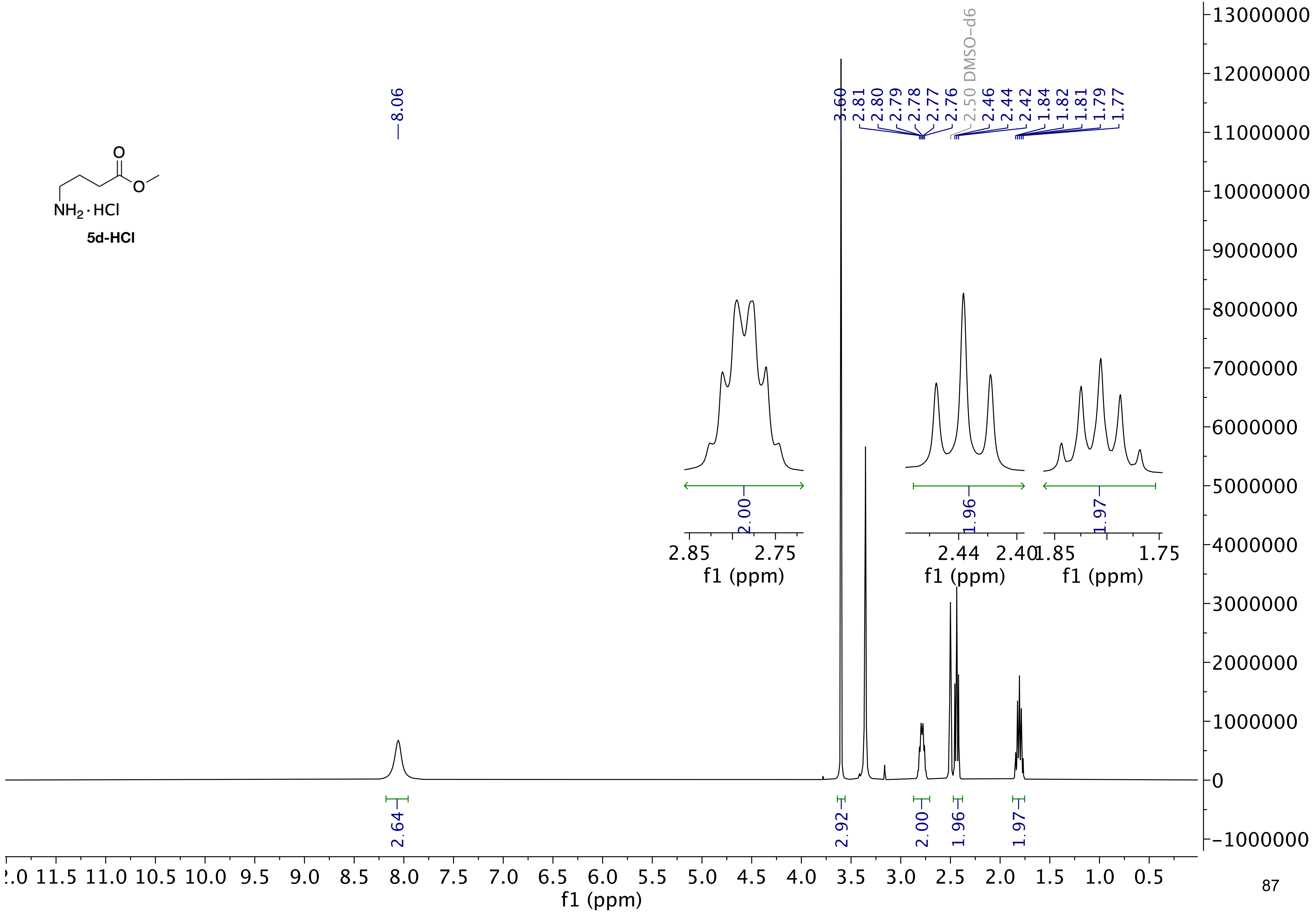

NH2 · HCl
O
O
5d-HCl
8.06
3.60
2.81
2.80
2.79
2.78
2.77
2.76
2.50 DMSO-d6
2.46
2.44
2.42
1.84
1.82
1.81
1.79
1.77
2.00
1.96
1.97
2.64
2.92
f1 (ppm)

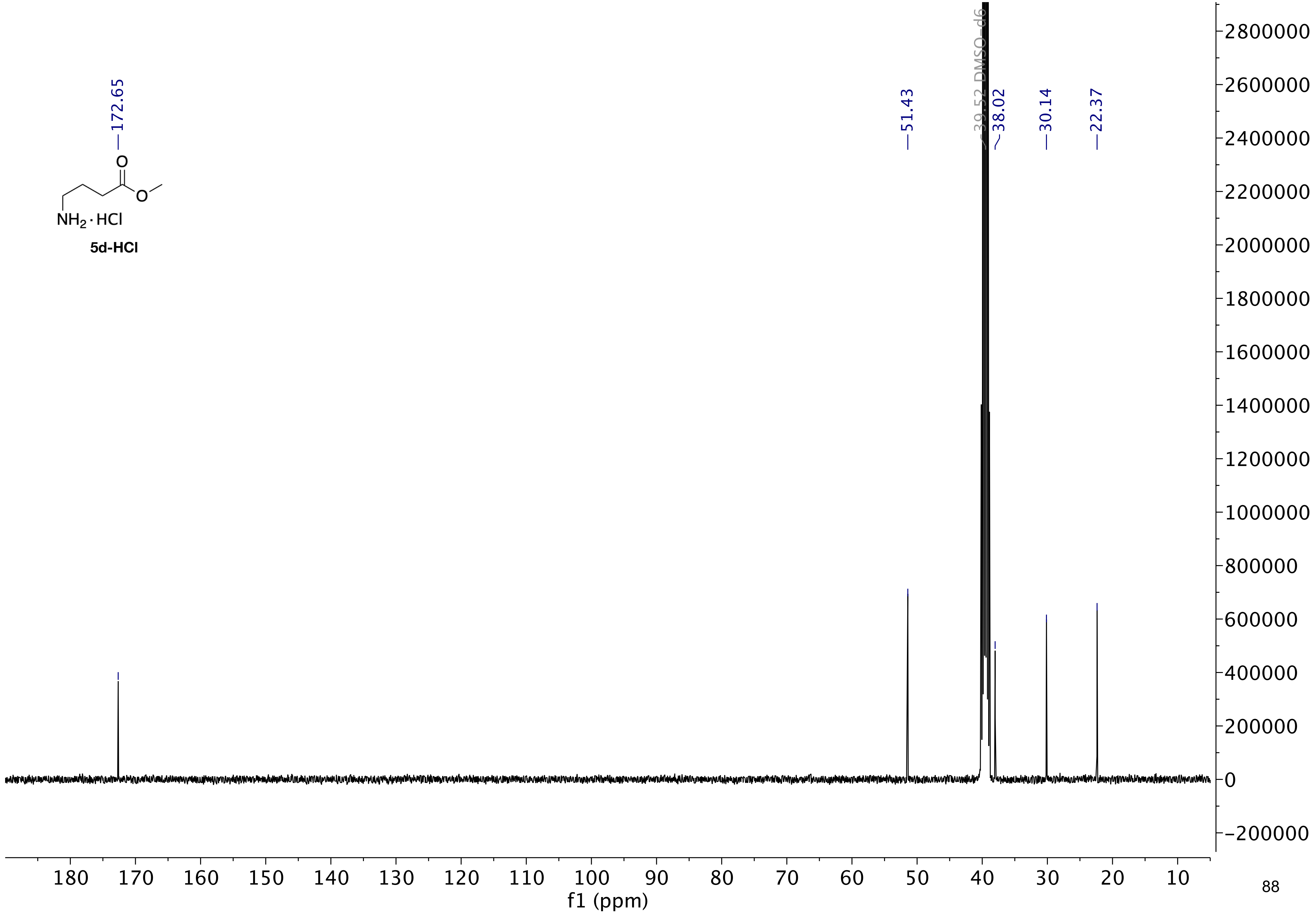
172.65
51.43
39.52 DMSO_d6
38.02
30.14
22.37
NH2 · HCl
5d-HCl
f1 (ppm)

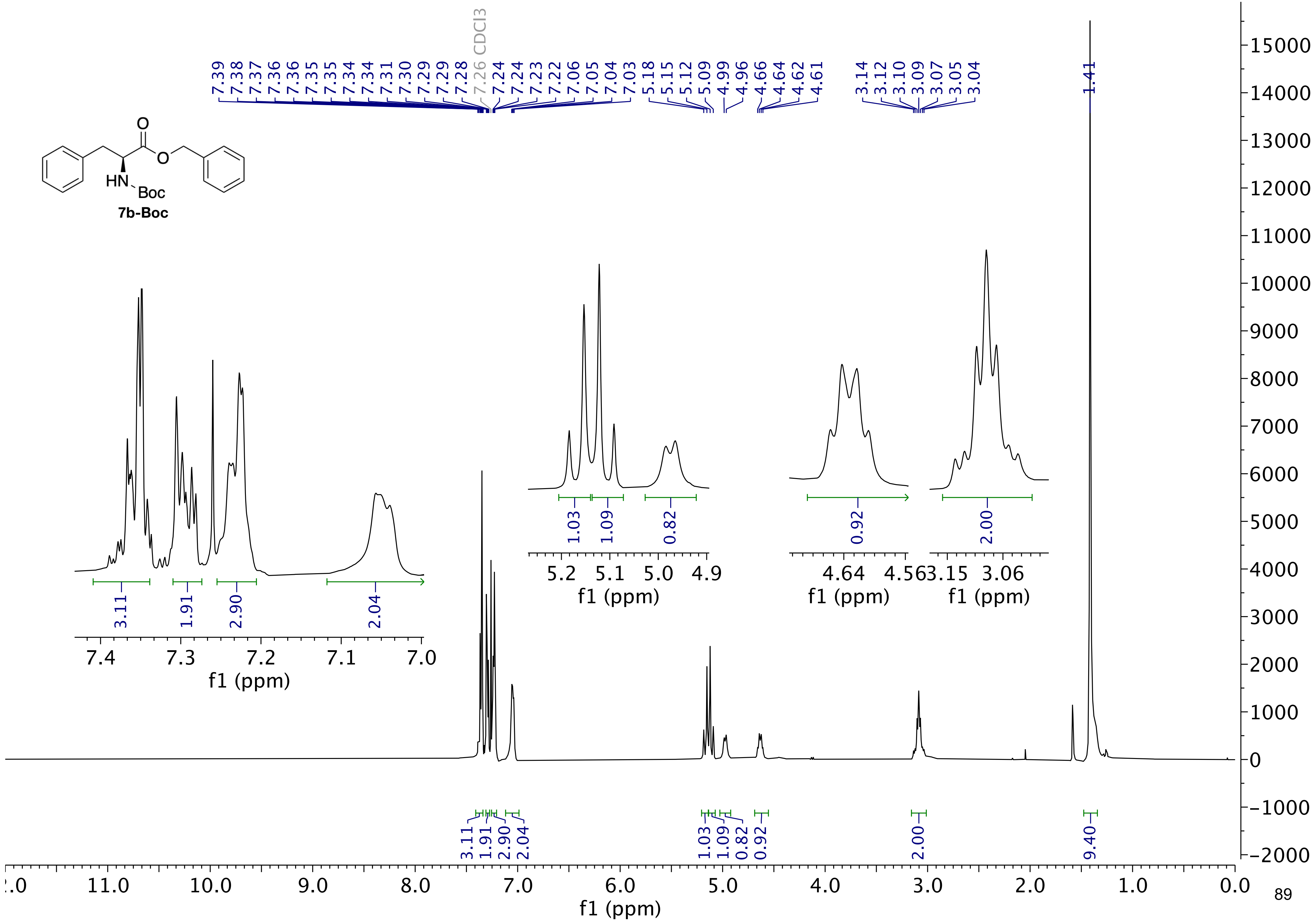
7.39 7.38 7.37 7.36 7.36 7.35 7.35 7.34 7.34 7.31 7.30 7.29 7.29 7.28 7.26 CDCl3 7.24 7.24 7.23 7.22 7.06 7.05 7.04 7.03 5.18 5.15 5.12 5.09 4.99 4.96 4.66 4.64 4.62 4.61 3.14 3.12 3.10 3.09 3.07 3.05 3.04 1.41
O
O
HN
Boc
7b-Boc
3.11
1.91
2.90
2.04
1.03
1.09
0.82
0.92
2.00
9.40
f1 (ppm)
15000
14000
13000
12000
11000
10000
9000
8000
7000
6000
5000
4000
3000
2000
1000
0
−1000
−2000

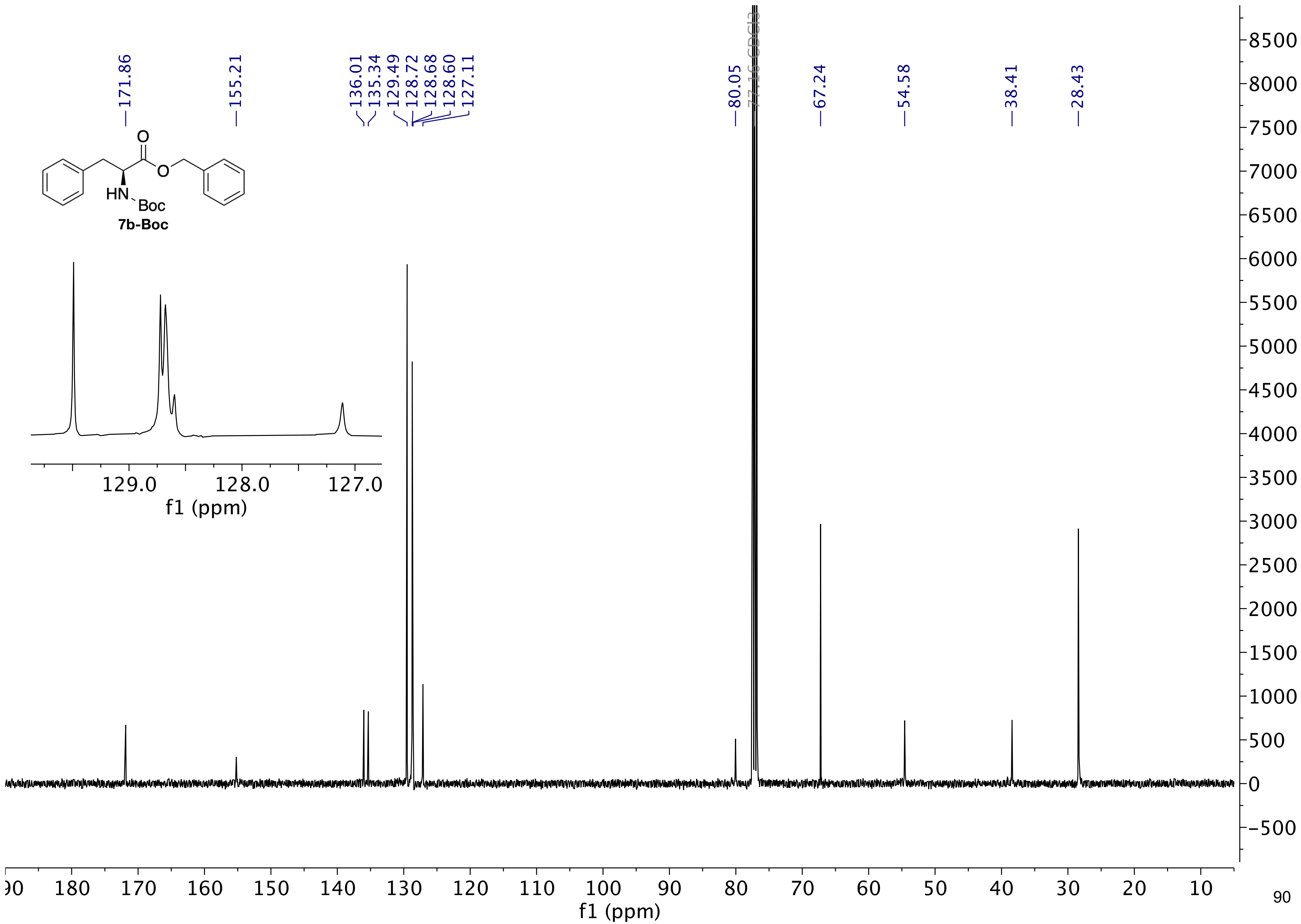

171.86
155.21
136.01
135.34
129.49
128.72
128.68
128.60
127.11
80.05
77.16 CDCl3
67.24
54.58
38.41
28.43
HN
Boc
O
7b-Boc
129.0
128.0
127.0
f1 (ppm)
8500
8000
7500
7000
6500
6000
5500
5000
4500
4000
3500
3000
2500
2000
1500
1000
500
0
−500
180
170
160
150
140
130
120
110
100
90
80
70
60
50
40
30
20
10
f1 (ppm)

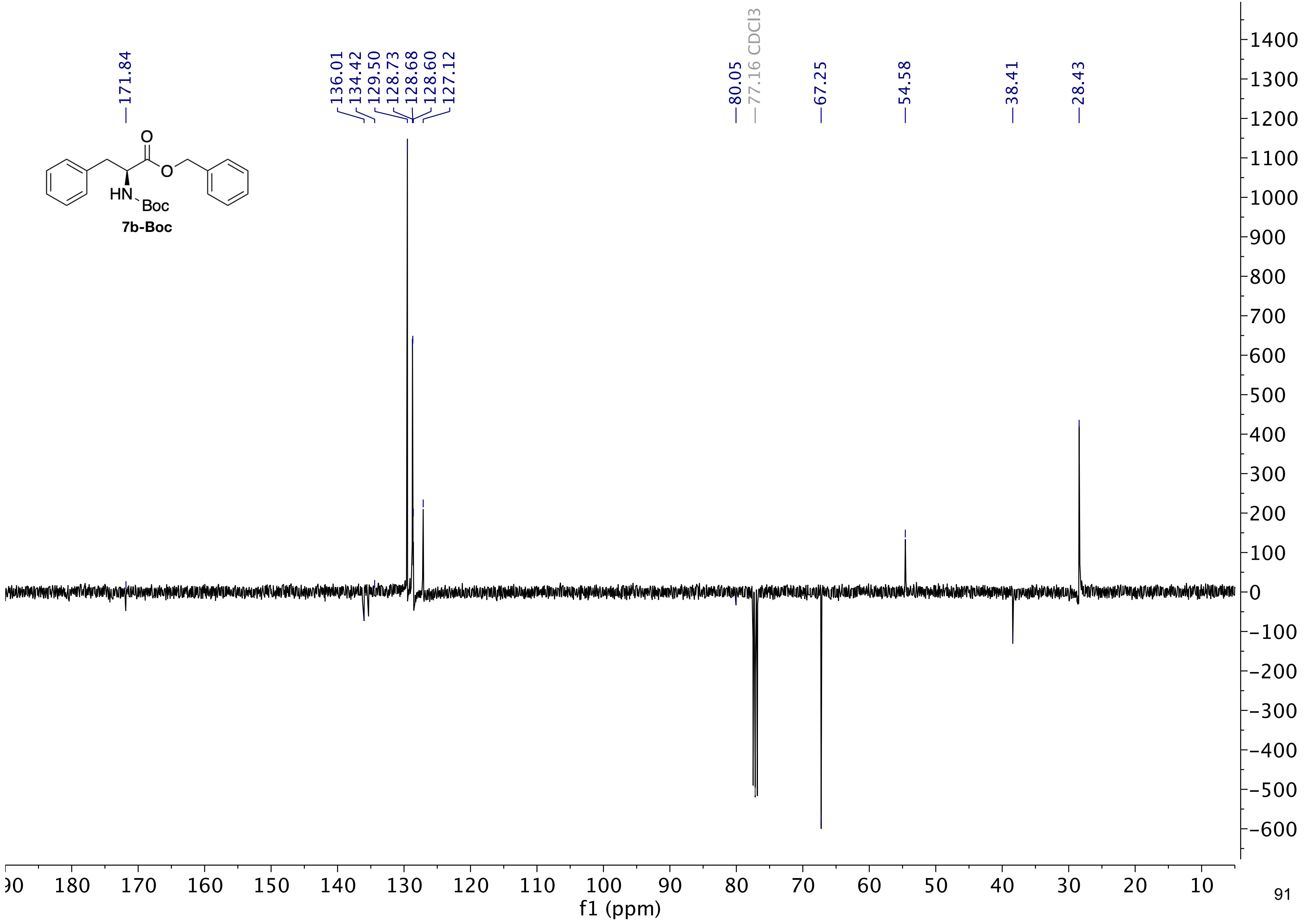
171.84
136.01
134.42
129.50
128.73
128.68
128.60
127.12
80.05
77.16 CDCl3
67.25
54.58
38.41
28.43
O
O
HN
Boc
7b-Boc
f1 (ppm)

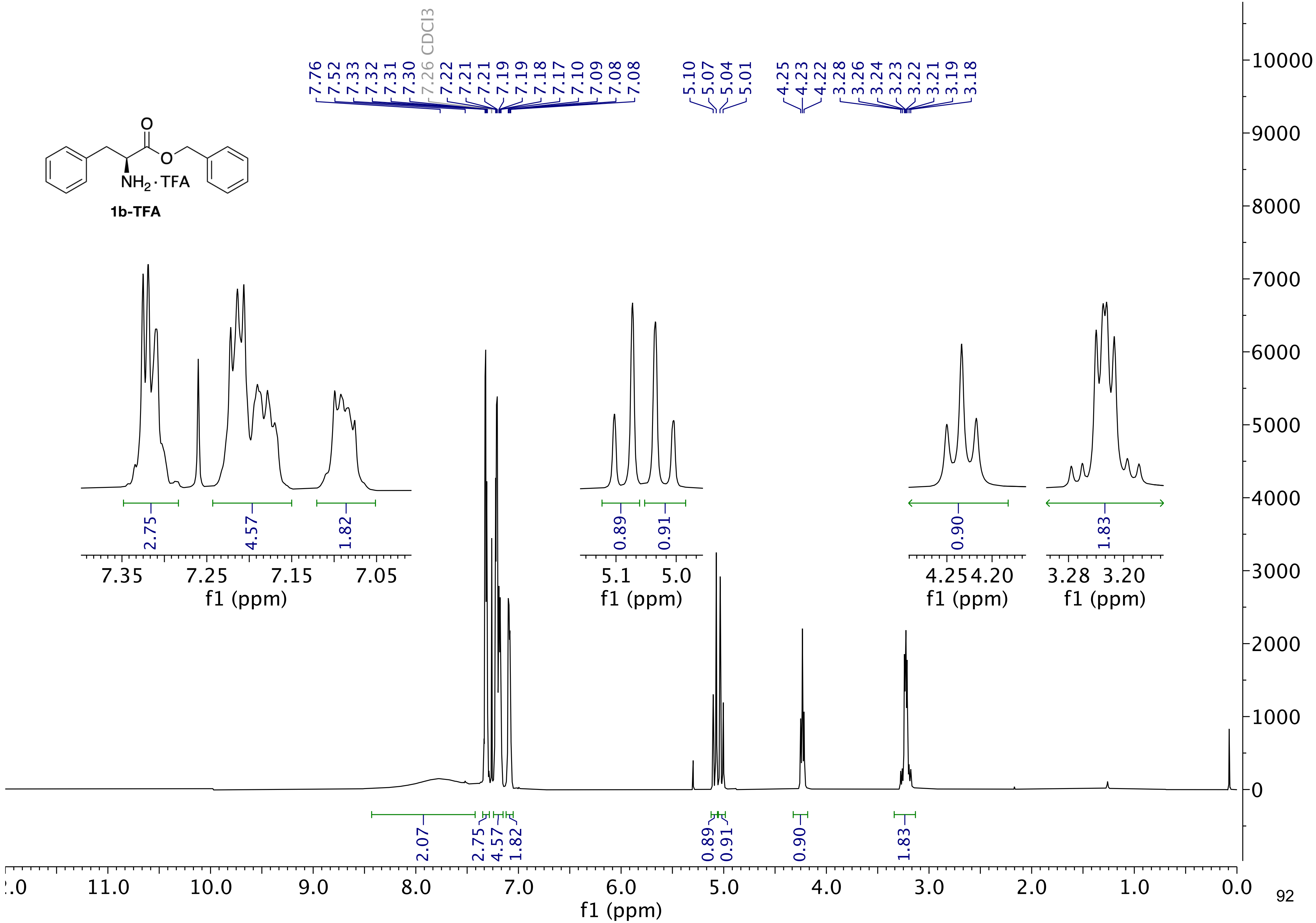

1b-TFA
NH2·TFA
7.76 7.52 7.33 7.32 7.31 7.30 7.26 CDCl3 7.22 7.21 7.21 7.19 7.19 7.18 7.17 7.10 7.09 7.08 7.08
5.10 5.07 5.04 5.01
4.25 4.23 4.22
3.28 3.26 3.24 3.23 3.22 3.21 3.19 3.18
2.07 2.75 4.57 1.82 0.89 0.91 0.90 1.83
f1 (ppm)

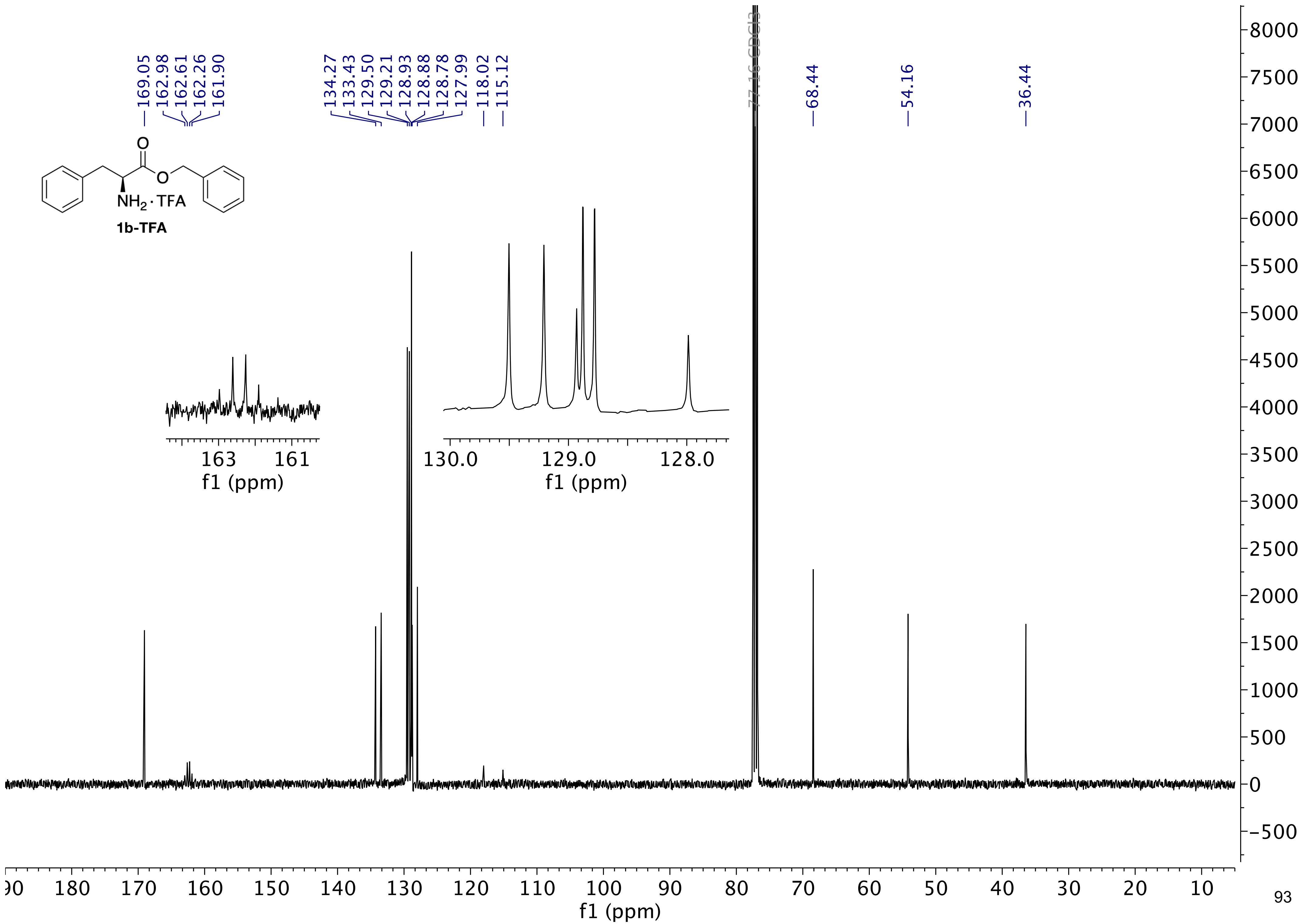
169.05
162.98
162.61
162.26
161.90
134.27
133.43
129.50
129.21
128.93
128.88
128.78
127.99
118.02
115.12
77.16 CDCl3
68.44
54.16
36.44
NH2 · TFA
1b-TFA
f1 (ppm)

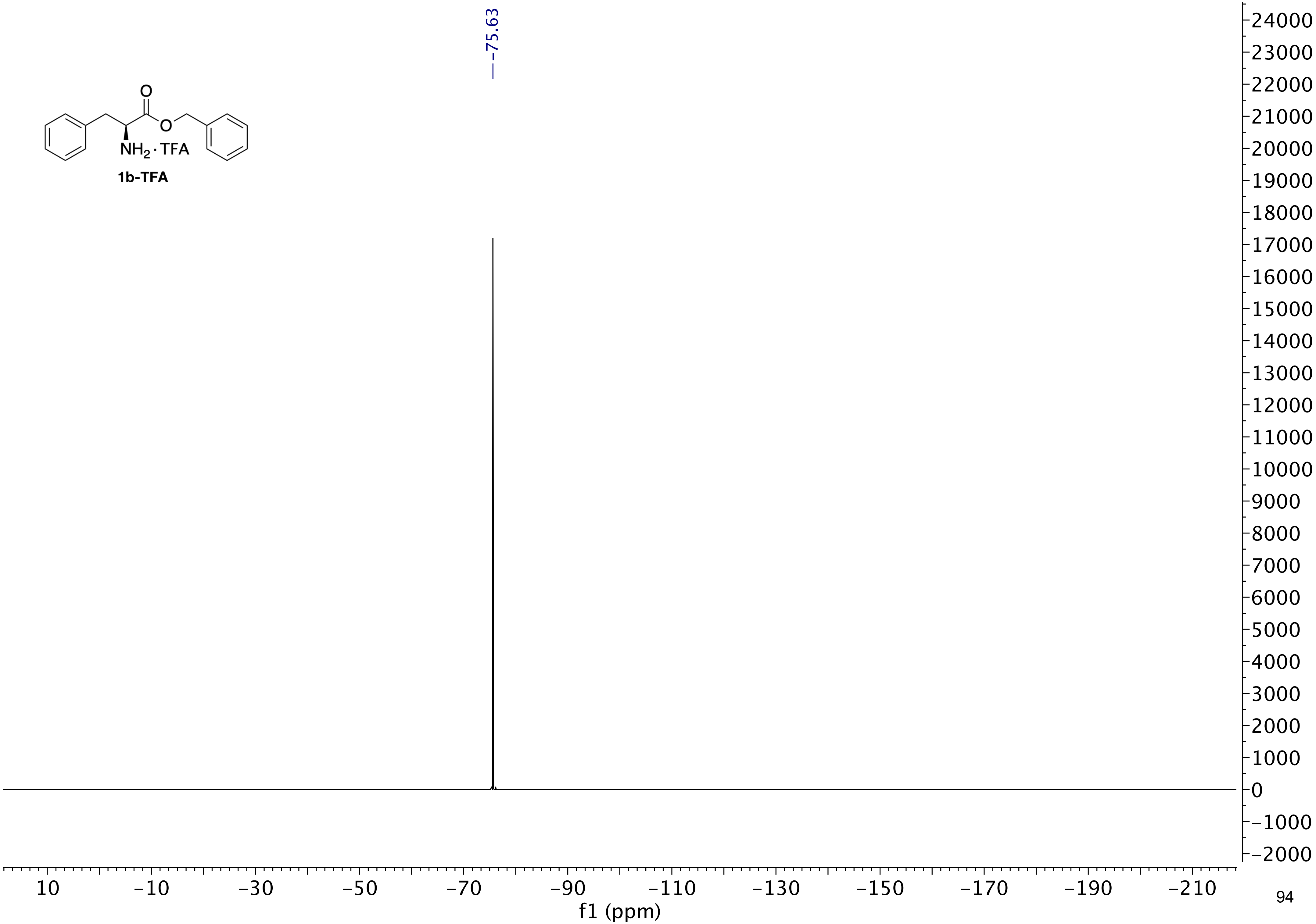

−75.63
NH2 · TFA
1b-TFA
f1 (ppm)
10 −10 −30 −50 −70 −90 −110 −130 −150 −170 −190 −210
24000 23000 22000 21000 20000 19000 18000 17000 16000 15000 14000 13000 12000 11000 10000 9000 8000 7000 6000 5000 4000 3000 2000 1000 0 −1000 −2000

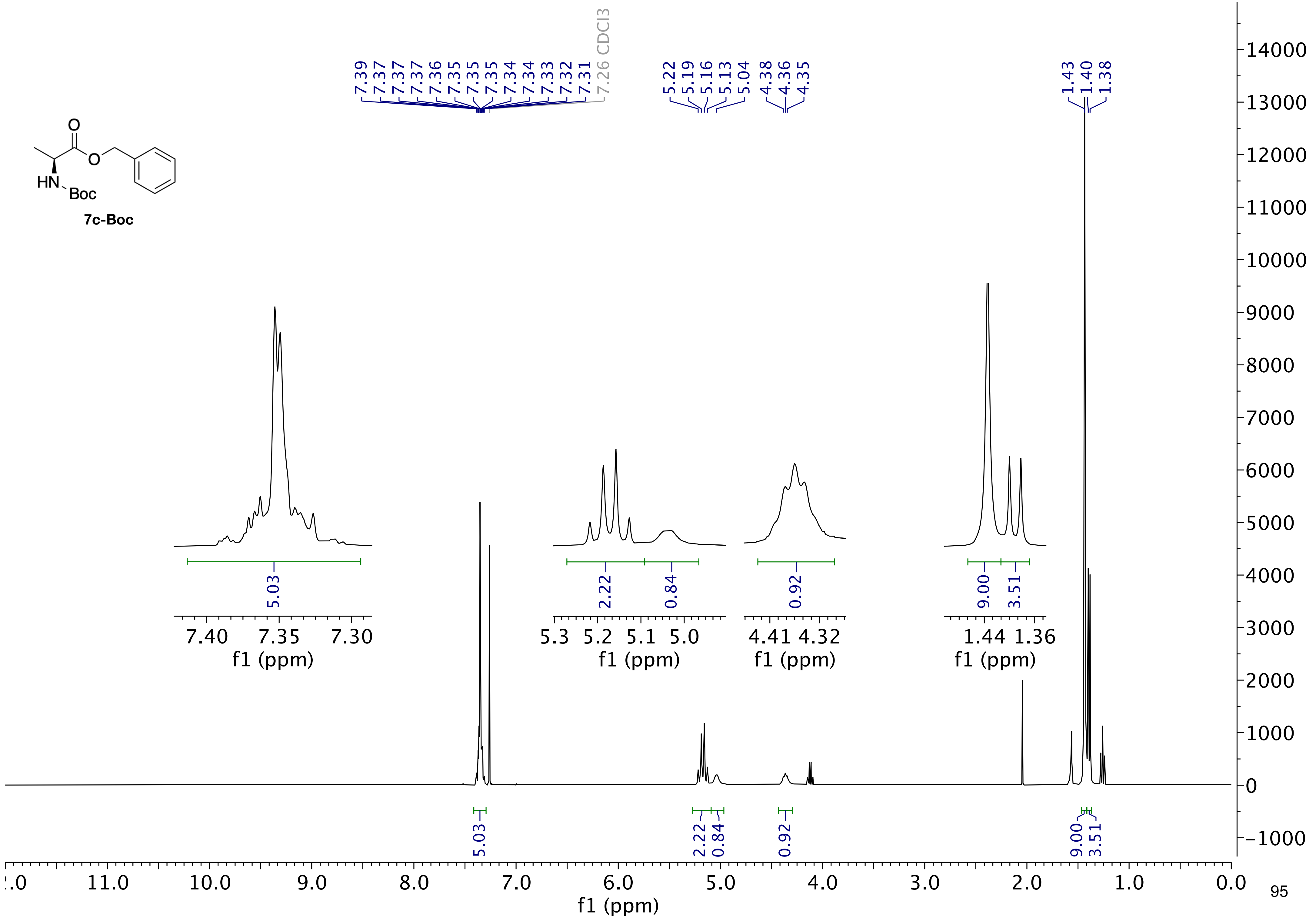

7.39
7.37
7.37
7.37
7.36
7.35
7.35
7.35
7.34
7.34
7.33
7.32
7.31
7.26 CDCl3
5.22
5.19
5.16
5.13
5.04
4.38
4.36
4.35
1.43
1.40
1.38
7c-Boc
5.03
2.22
0.84
0.92
9.00
3.51
f1 (ppm)

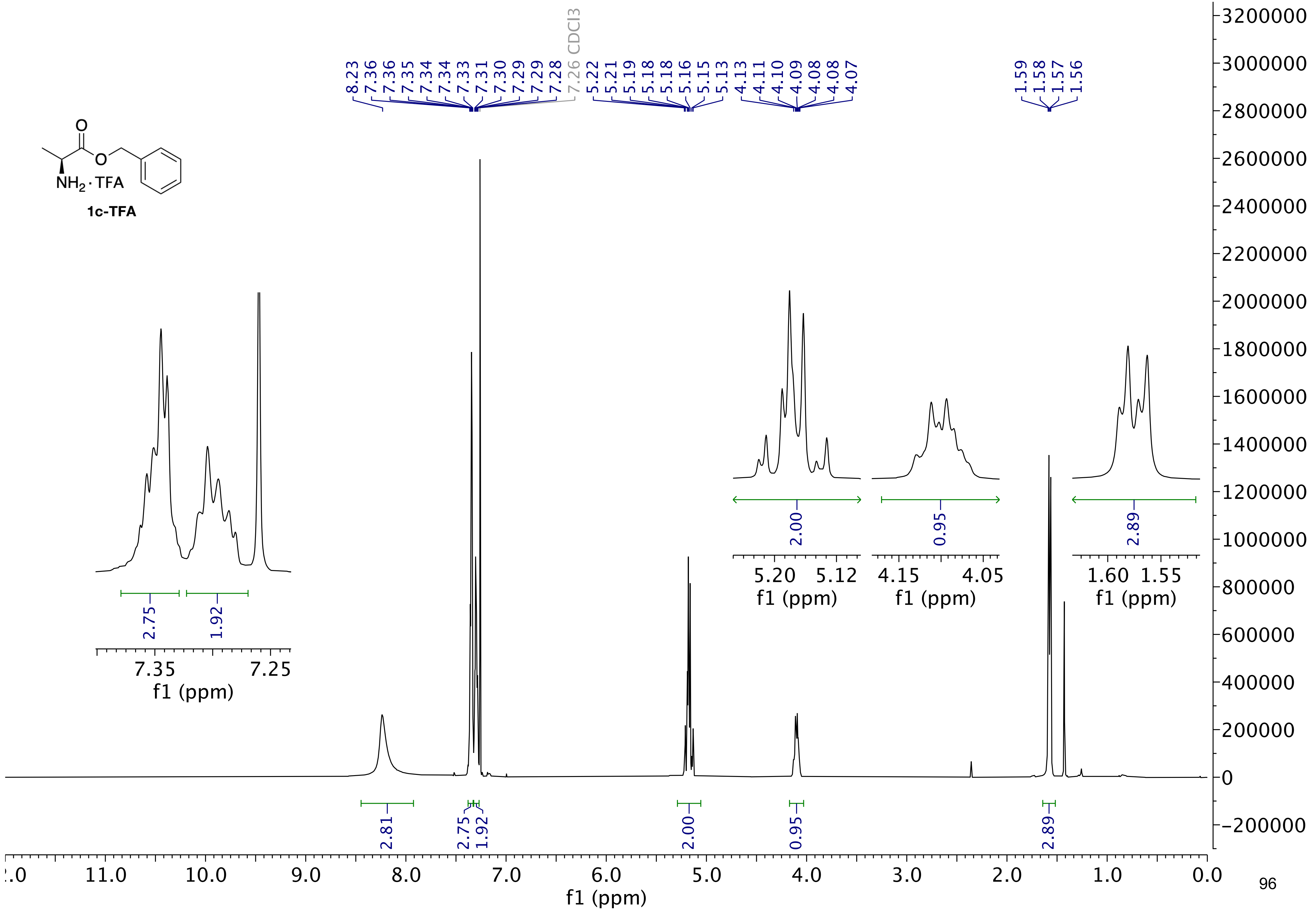

1c-TFA
NH2 · TFA
8.23 7.36 7.36 7.35 7.34 7.34 7.33 7.31 7.30 7.29 7.29 7.28 7.26 CDCl3 5.22 5.21 5.19 5.18 5.18 5.16 5.15 5.13 4.13 4.11 4.10 4.09 4.08 4.08 4.07
1.59 1.58 1.57 1.56
2.81 2.75 1.92 2.00 0.95 2.89
f1 (ppm)

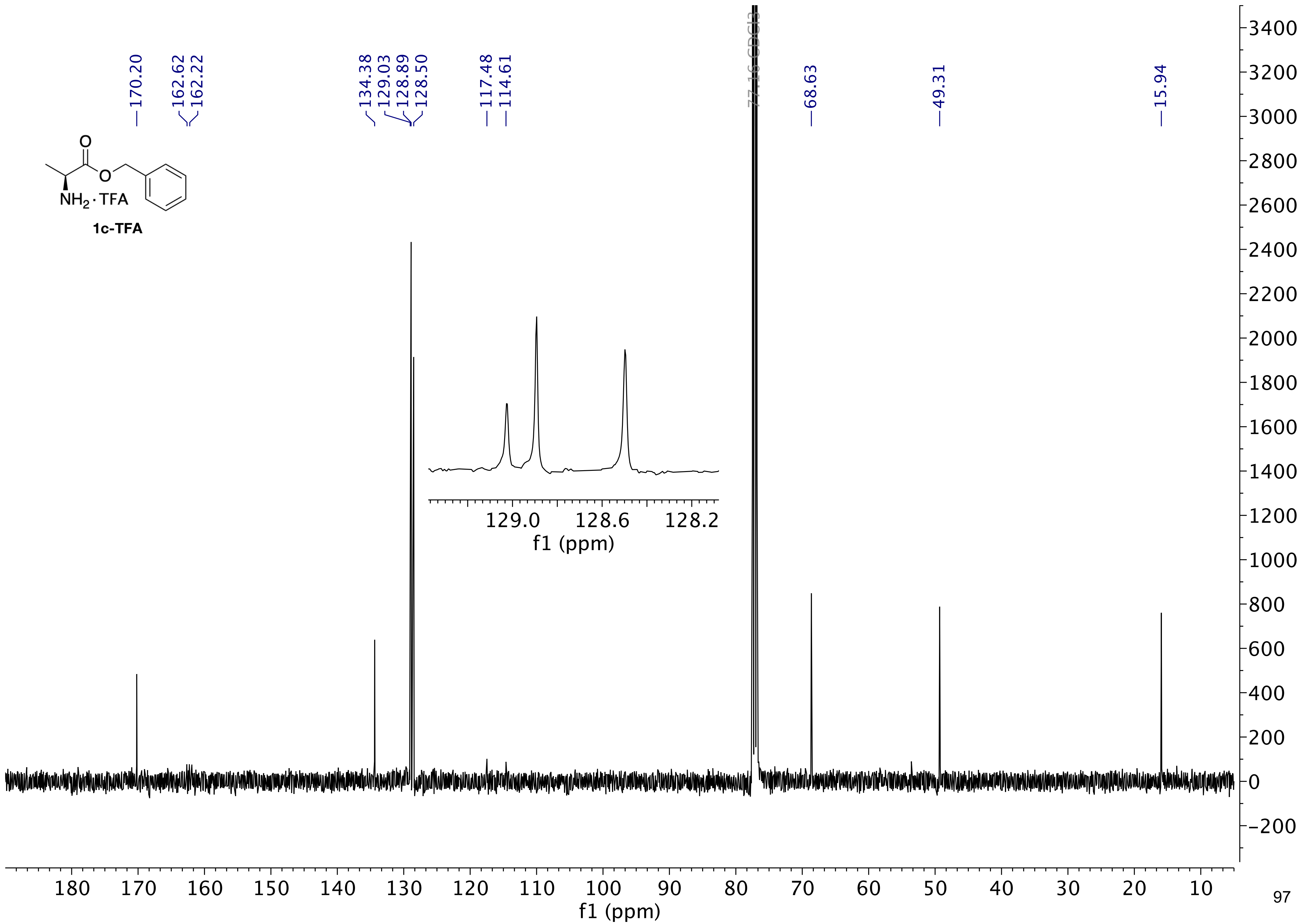
170.20
162.62
162.22
134.38
129.03
128.89
128.50
117.48
114.61
77.16 CDCl3
68.63
49.31
15.94
O
O
NH2 · TFA
1c-TFA
129.0
128.6
128.2
f1 (ppm)
f1 (ppm)

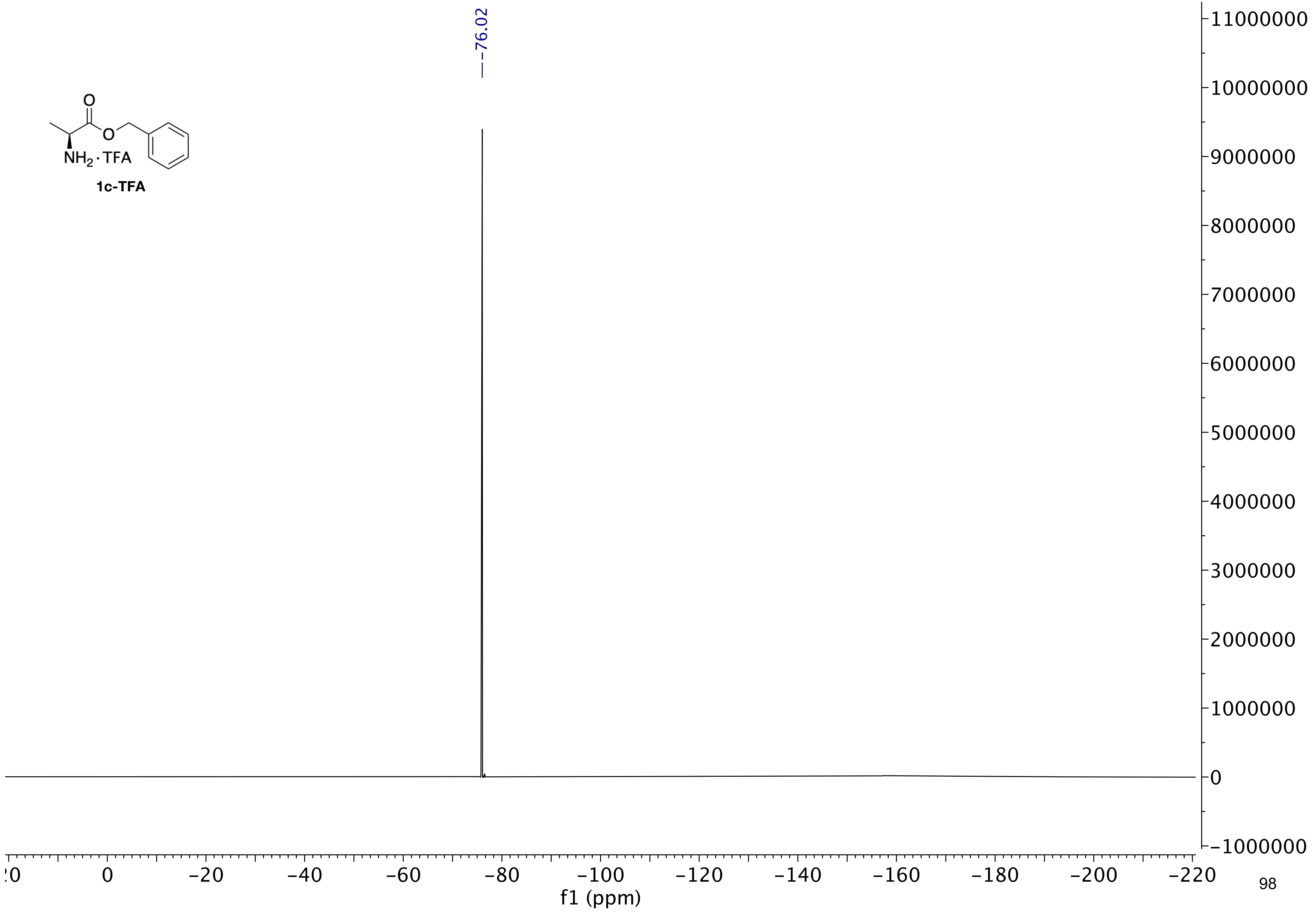

−76.02
NH2 · TFA
1c-TFA
f1 (ppm)

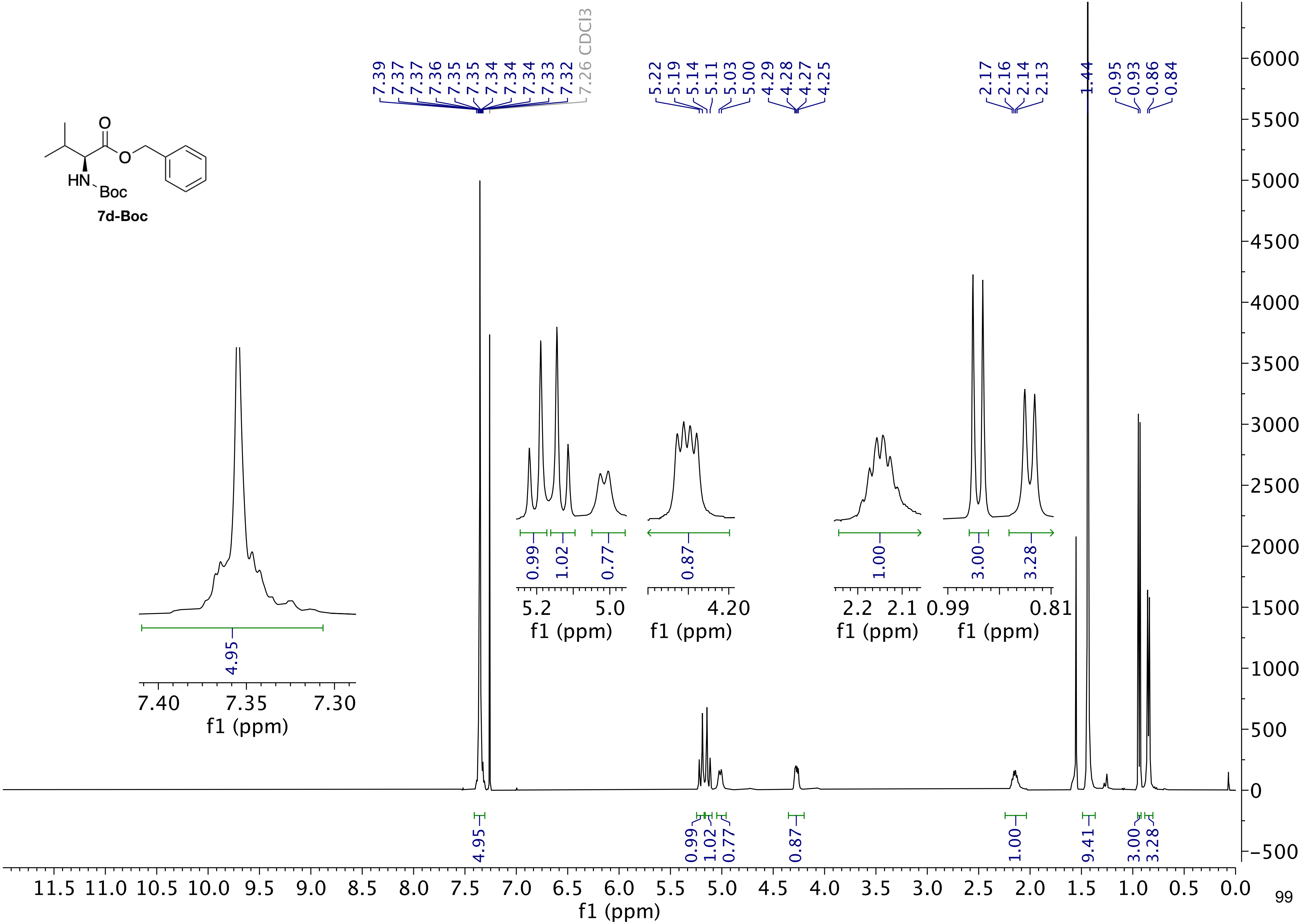

7d-Boc

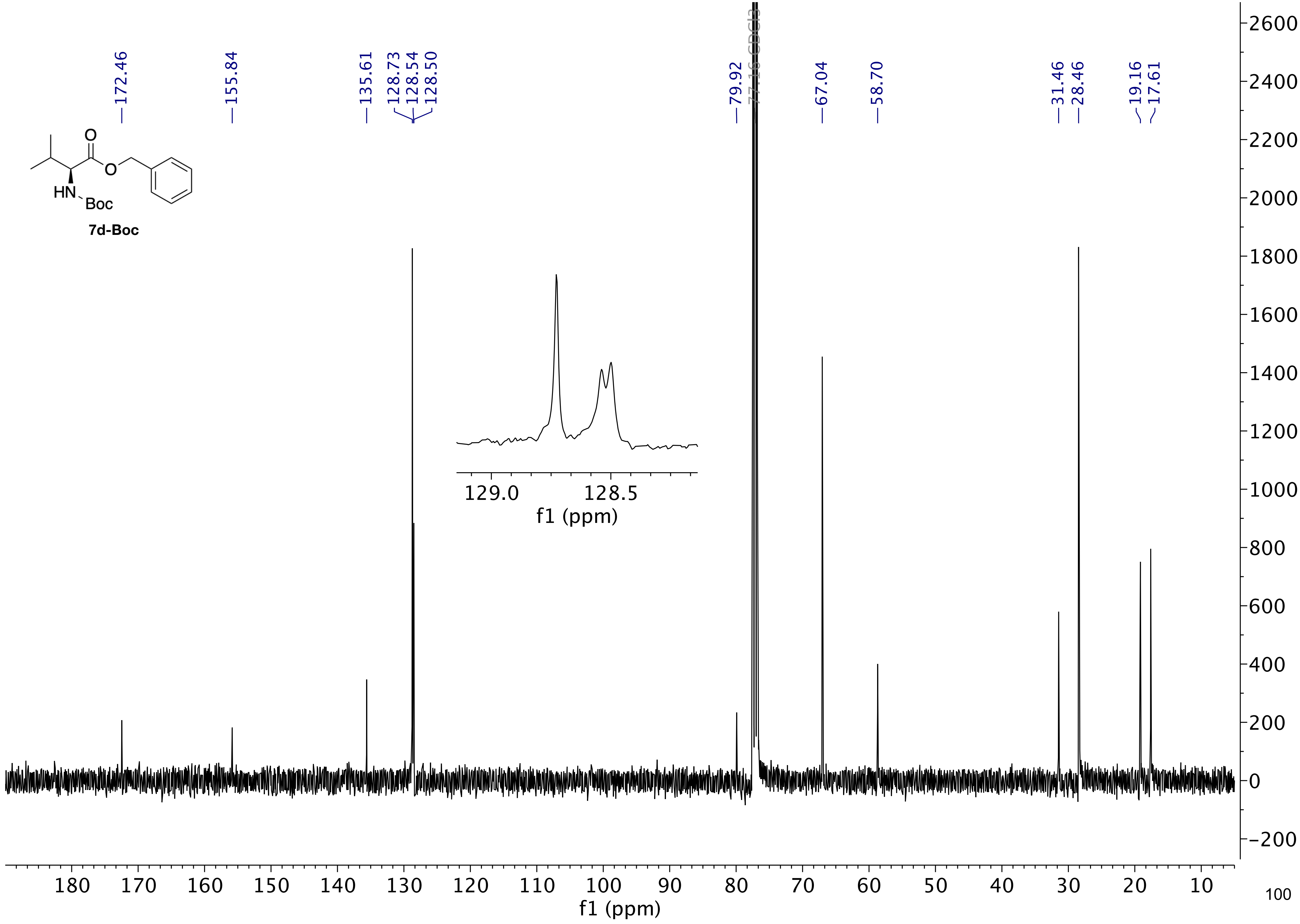

172.46
155.84
135.61
128.73
128.54
128.50
79.92
77.16 CDCl3
67.04
58.70
31.46
28.46
19.16
17.61
O
O
HN
Boc
7d-Boc
129.0
128.5
f1 (ppm)
f1 (ppm)

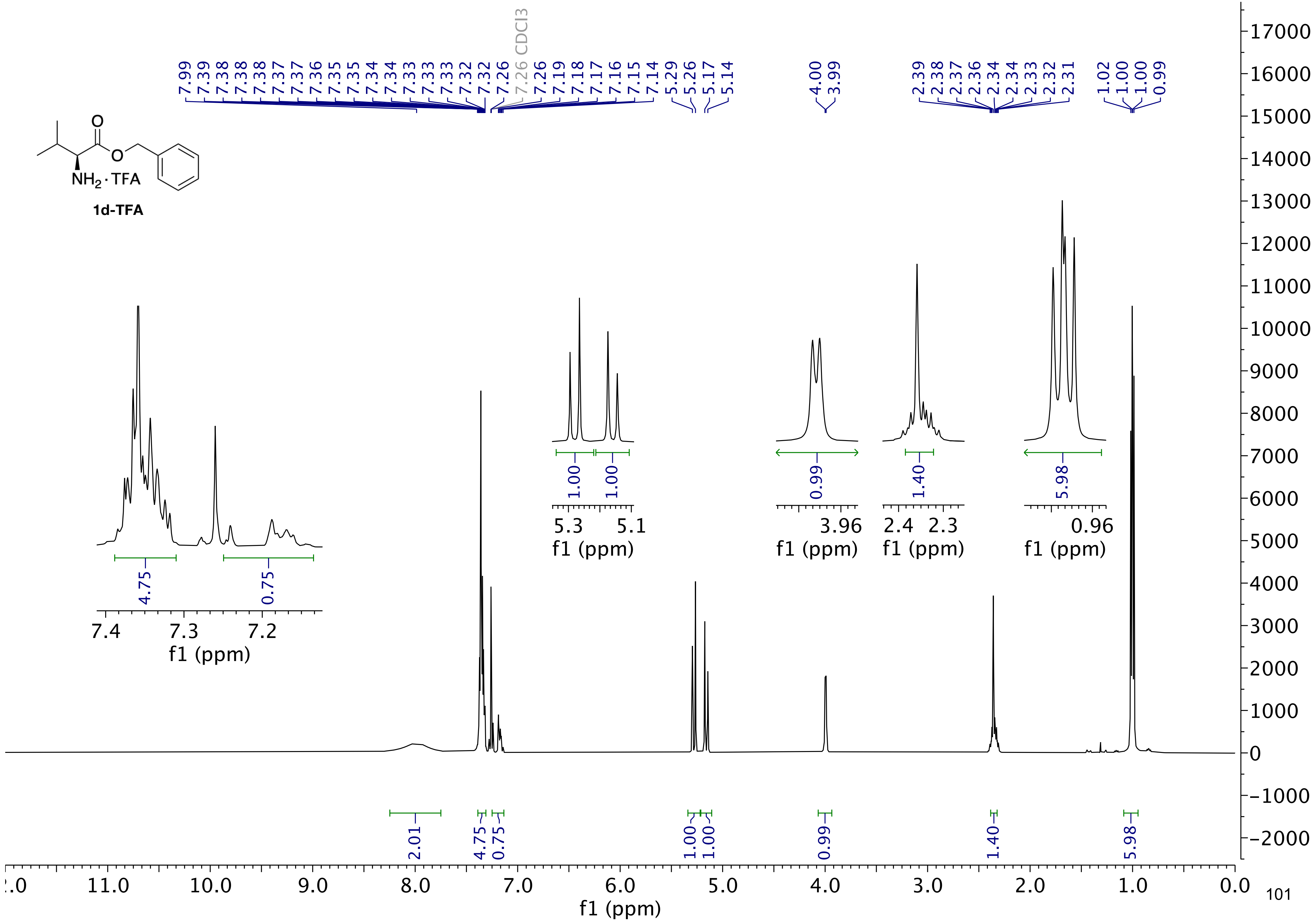
NH2·TFA
1d-TFA
7.99 7.39 7.38 7.38 7.38 7.37 7.37 7.36 7.35 7.35 7.34 7.34 7.33 7.33 7.33 7.32 7.32 7.26 7.26 CDCl3 7.26 7.19 7.18 7.17 7.16 7.15 7.14 5.29 5.26 5.17 5.14
4.00 3.99
2.39 2.38 2.37 2.36 2.34 2.34 2.33 2.32 2.31
1.02 1.00 1.00 0.99
4.75 0.75
1.00 1.00
0.99
1.40
5.98
2.01 4.75 0.75 1.00 1.00 0.99 1.40 5.98
f1 (ppm)

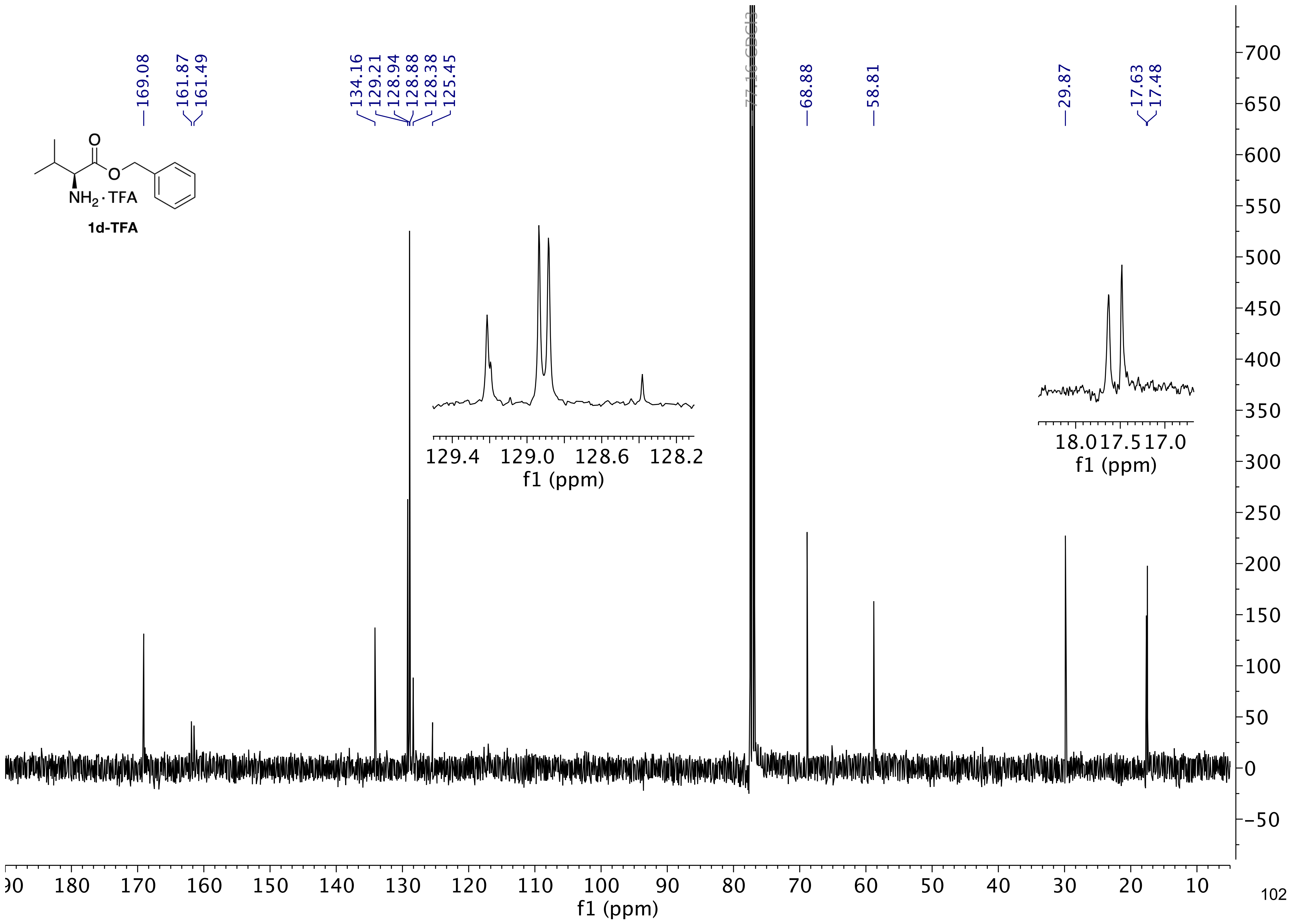
169.08
161.87
161.49
134.16
129.21
128.94
128.88
128.38
125.45
77.16 CDCl3
68.88
58.81
29.87
17.63
17.48
NH2 · TFA
1d-TFA
f1 (ppm)

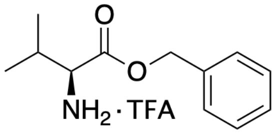
O
O
NH2 · TFA

1d-TFA

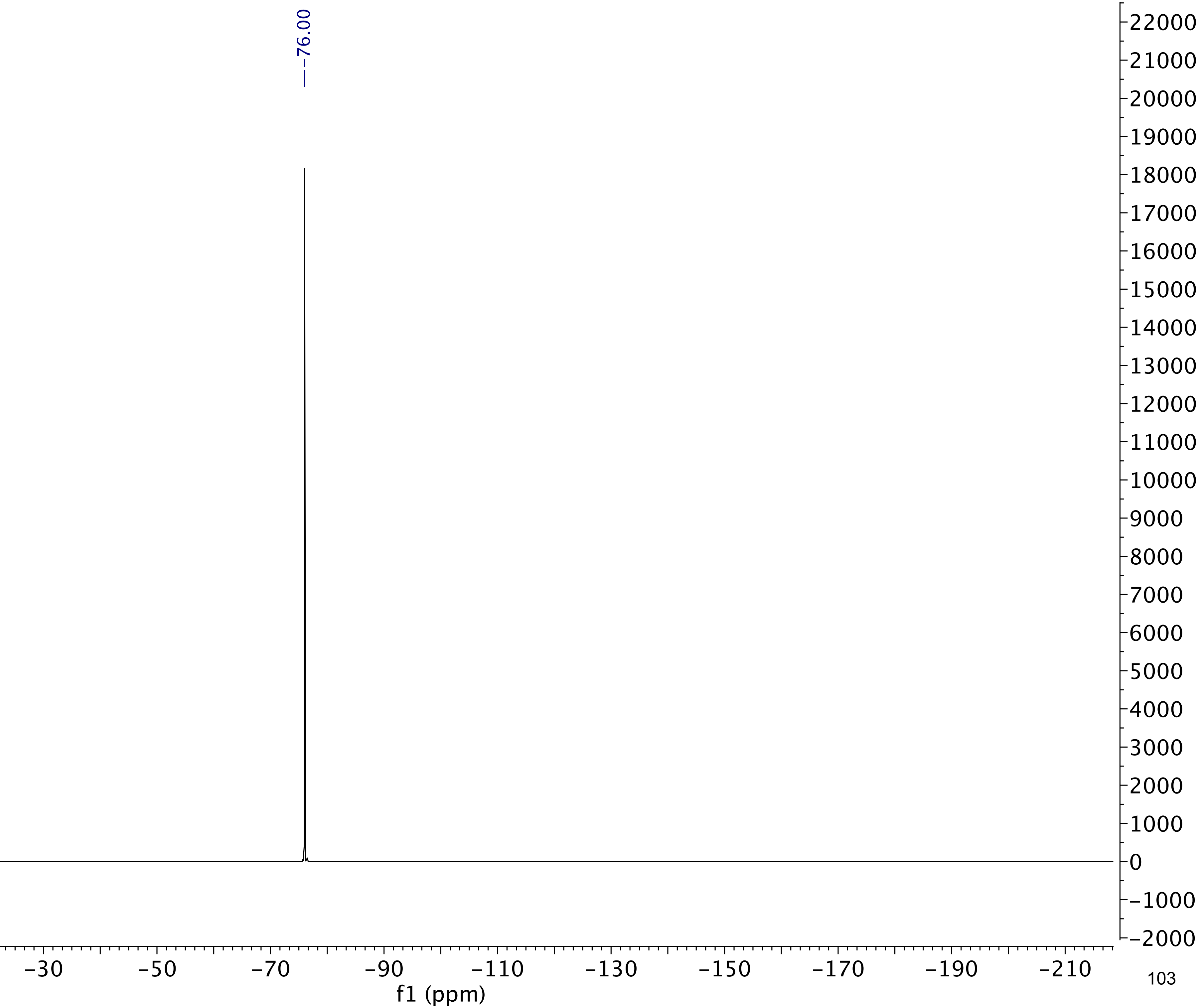
−76.00
22000
21000
20000
19000
18000
17000
16000
15000
14000
13000
12000
11000
10000
9000
8000
7000
6000
5000
4000
3000
2000
1000
0
−1000
−2000
10
−10
−30
−50
−70
−90
−110
−130
−150
−170
−190
−210
f1 (ppm)

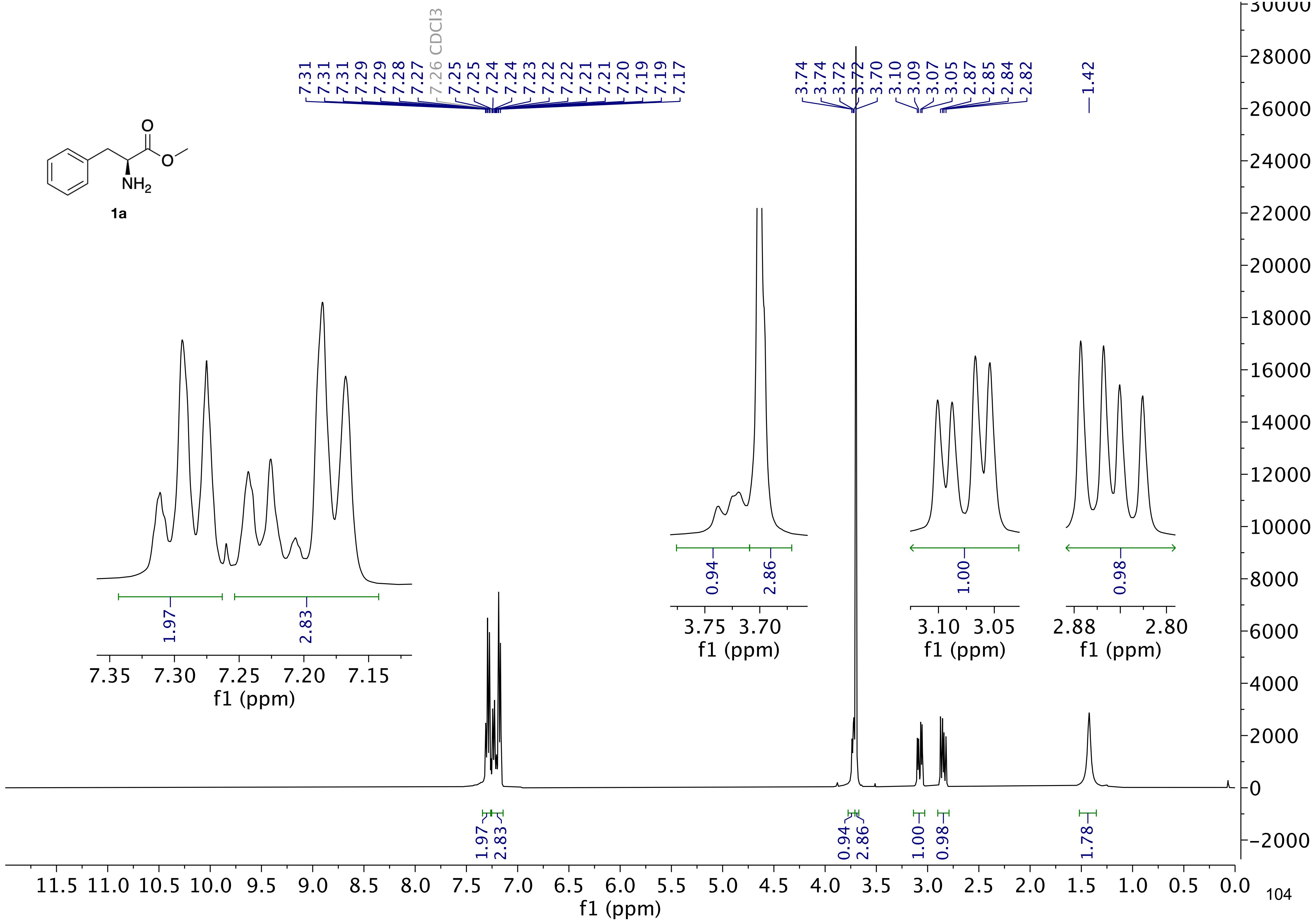
7.31 7.31 7.31 7.29 7.29 7.28 7.27 7.26 CDCl3 7.25 7.25 7.24 7.24 7.23 7.22 7.22 7.21 7.21 7.20 7.19 7.19 7.17
3.74 3.74 3.72 3.72 3.70 3.10 3.09 3.07 3.05 2.87 2.85 2.84 2.82
1.42
O
O
NH2
1a
1.97
2.83
0.94
2.86
1.00
0.98
1.78
f1 (ppm)

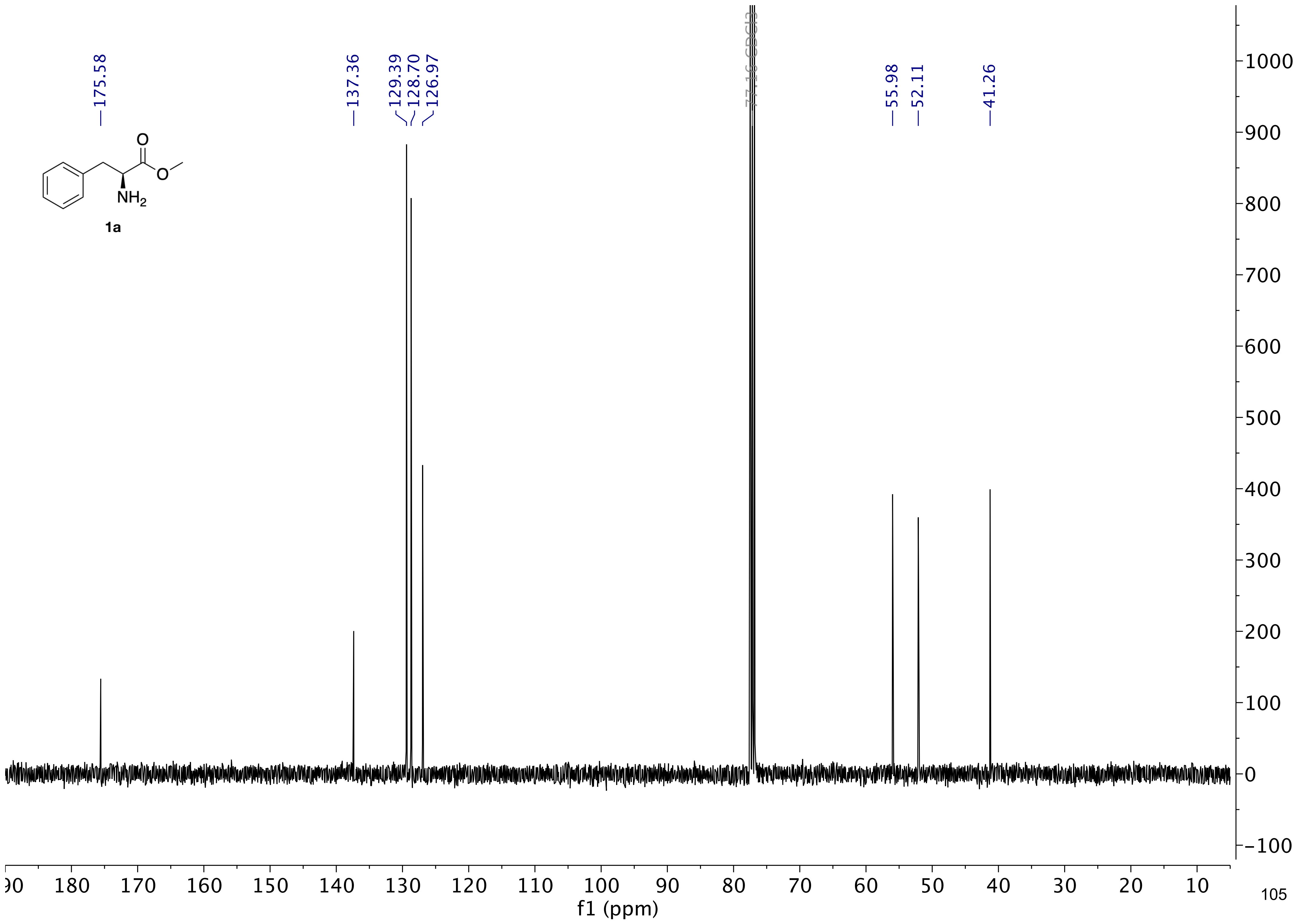
175.58
137.36
129.39
128.70
126.97
77.16 CDCl3
55.98
52.11
41.26
1a
f1 (ppm)

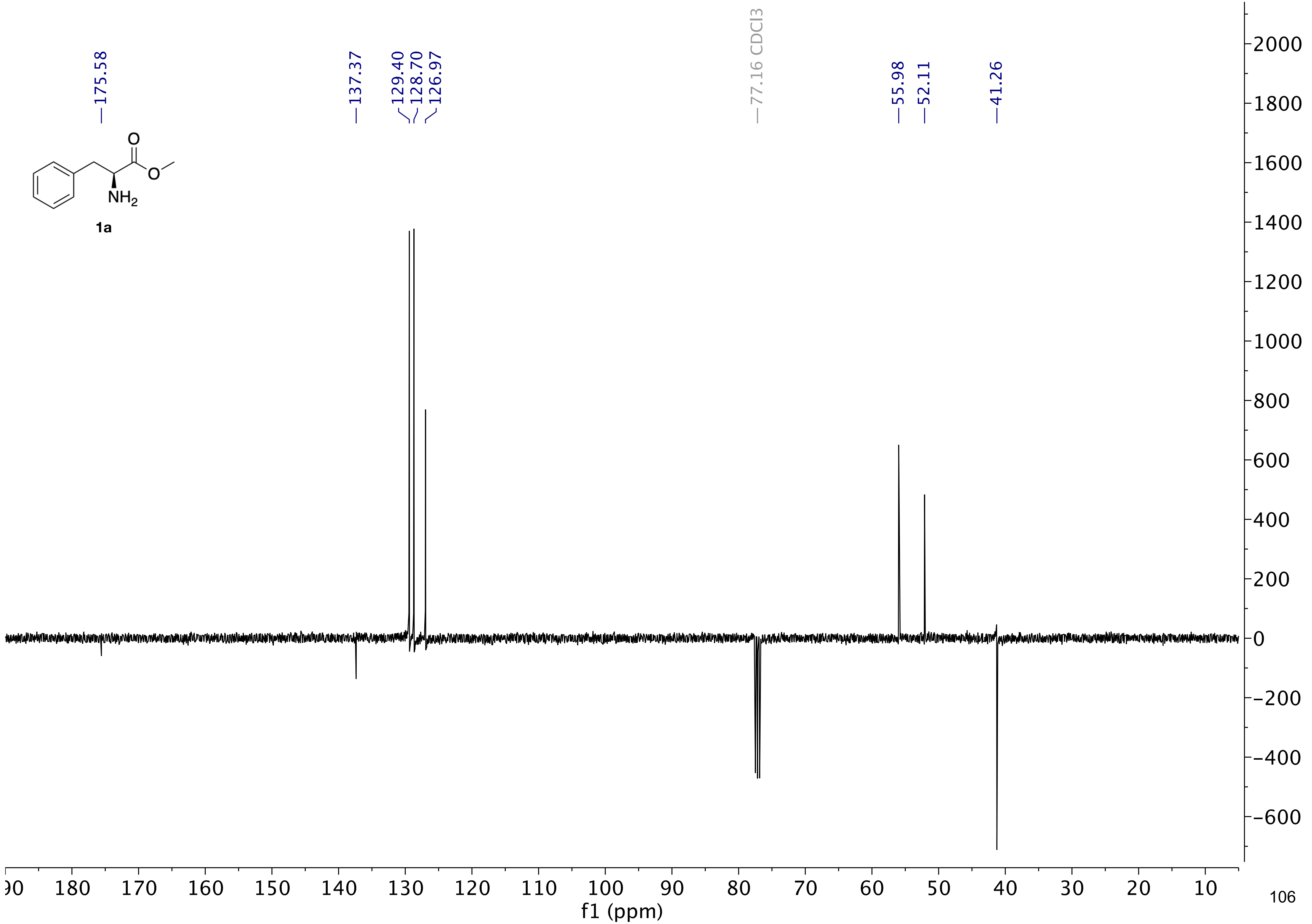
175.58
137.37
129.40
128.70
126.97
77.16 CDCl3
55.98
52.11
41.26
O
O
NH2
1a
f1 (ppm)

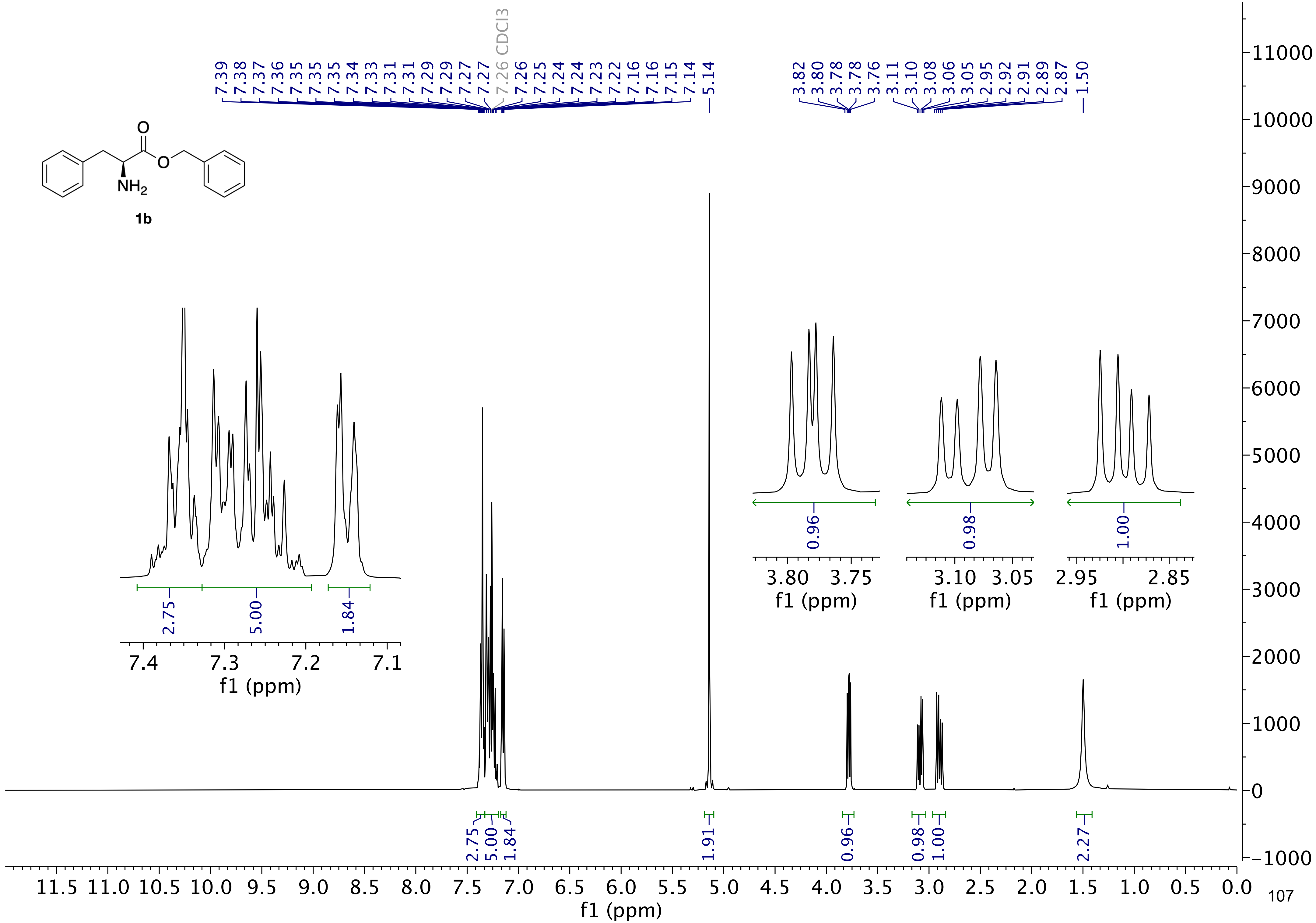
7.39 7.38 7.37 7.36 7.35 7.35 7.35 7.34 7.33 7.31 7.31 7.29 7.29 7.27 7.27 7.26 CDCl3 7.26 7.25 7.24 7.24 7.23 7.22 7.16 7.16 7.15 7.14 5.14
3.82 3.80 3.78 3.78 3.76 3.11 3.10 3.08 3.06 3.05 2.95 2.92 2.91 2.89 2.87 1.50
O
O
NH2
1b
2.75 5.00 1.84
0.96 0.98 1.00
2.75 5.00 1.84 1.91 0.96 0.98 1.00 2.27
f1 (ppm)

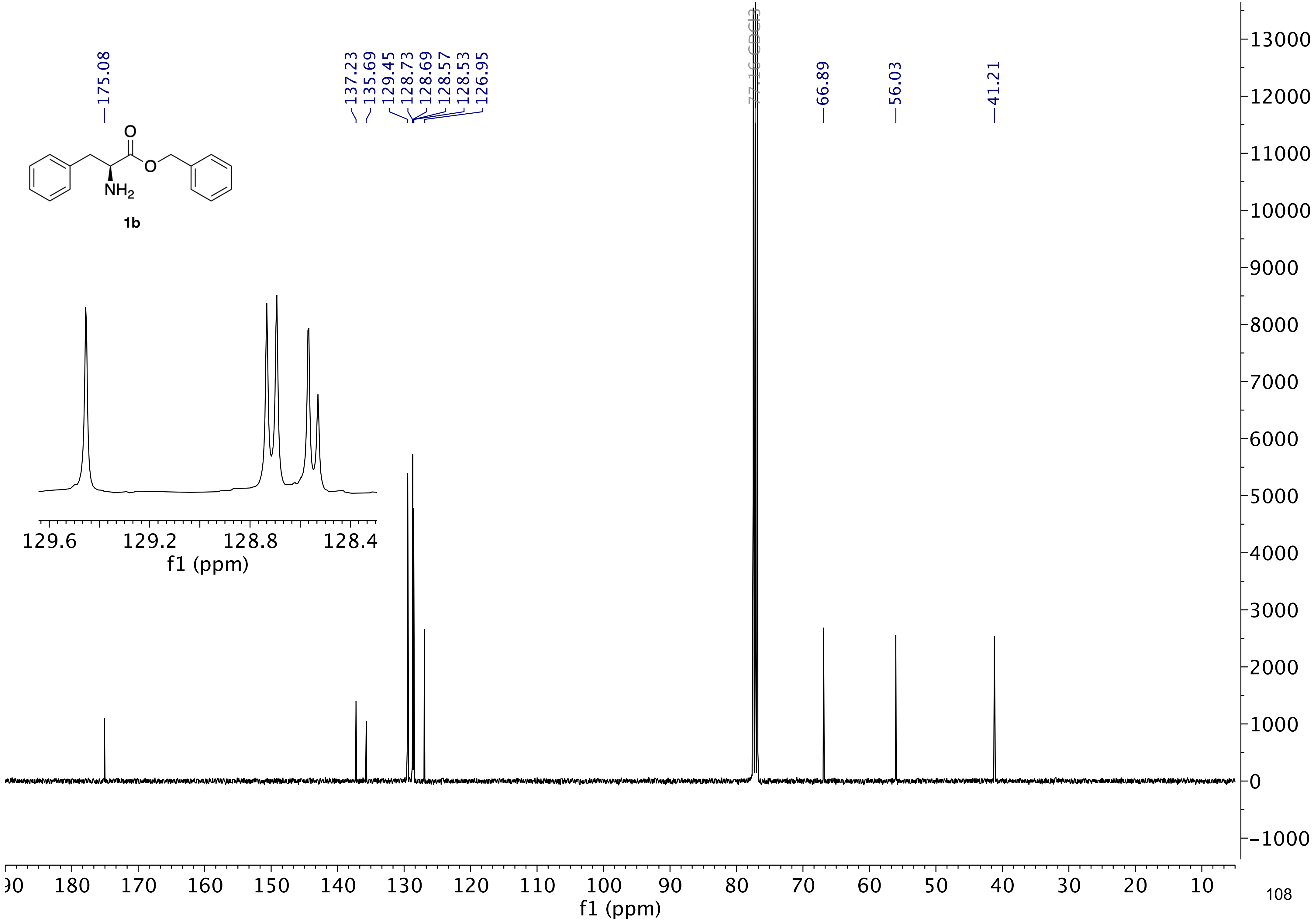
175.08
137.23
135.69
129.45
128.73
128.69
128.57
128.53
126.95
77.16 CDCl3
66.89
56.03
41.21
O
O
NH2
1b
129.6
129.2
128.8
128.4
f1 (ppm)
13000
12000
11000
10000
9000
8000
7000
6000
5000
4000
3000
2000
1000
0
−1000
90
180
170
160
150
140
130
120
110
100
90
80
70
60
50
40
30
20
10
f1 (ppm)

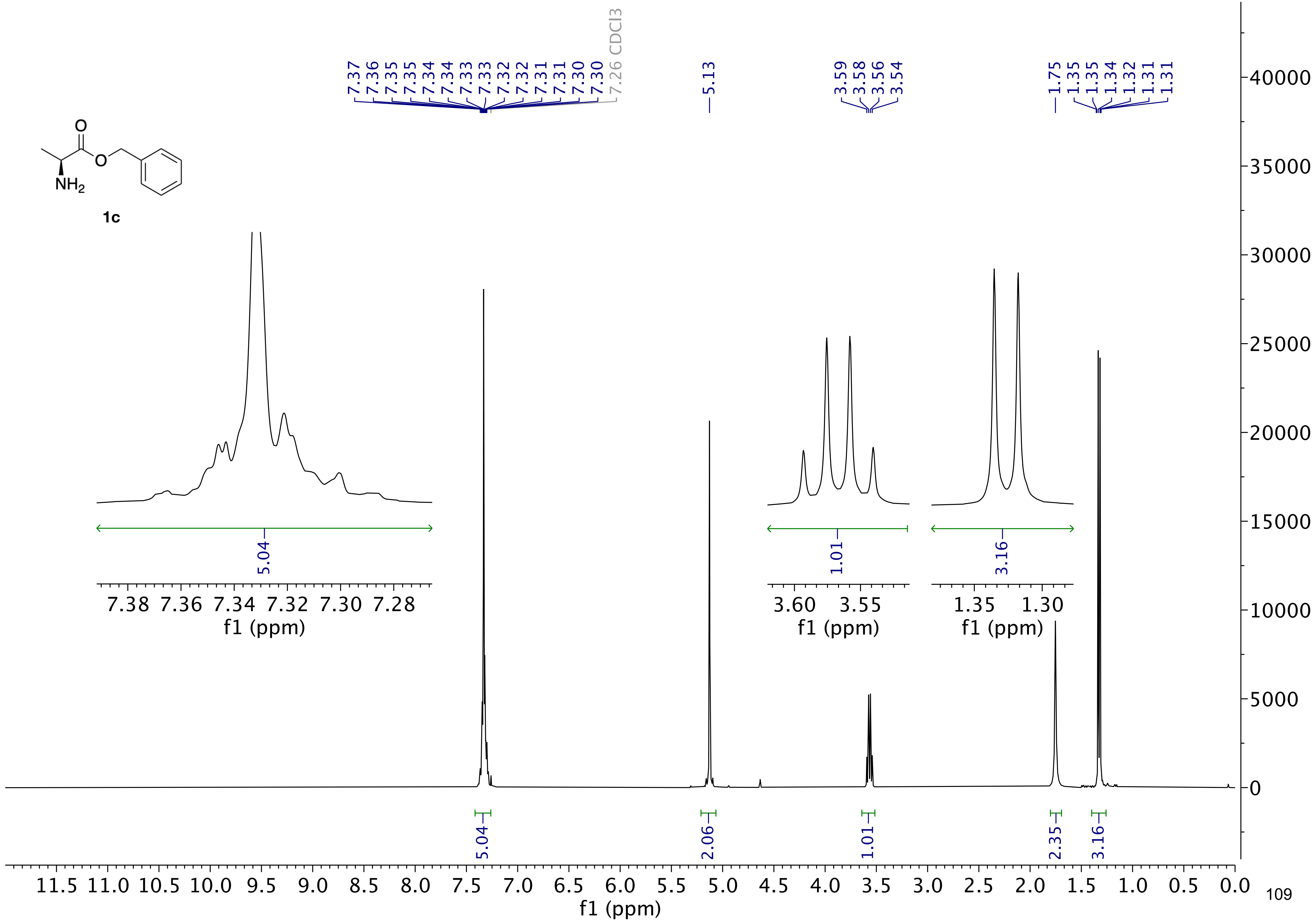

7.37 7.36 7.35 7.35 7.34 7.34 7.33 7.33 7.32 7.32 7.31 7.31 7.30 7.30 7.26 CDCl3
5.13
3.59 3.58 3.56 3.54
1.75 1.35 1.35 1.34 1.32 1.31 1.31
O
O
NH2
1c
5.04
1.01
3.16
7.38 7.36 7.34 7.32 7.30 7.28
3.60 3.55
1.35 1.30
f1 (ppm)
5.04 2.06 1.01 2.35 3.16
11.5 11.0 10.5 10.0 9.5 9.0 8.5 8.0 7.5 7.0 6.5 6.0 5.5 5.0 4.5 4.0 3.5 3.0 2.5 2.0 1.5 1.0 0.5 0.0
f1 (ppm)
40000 35000 30000 25000 20000 15000 10000 5000 0

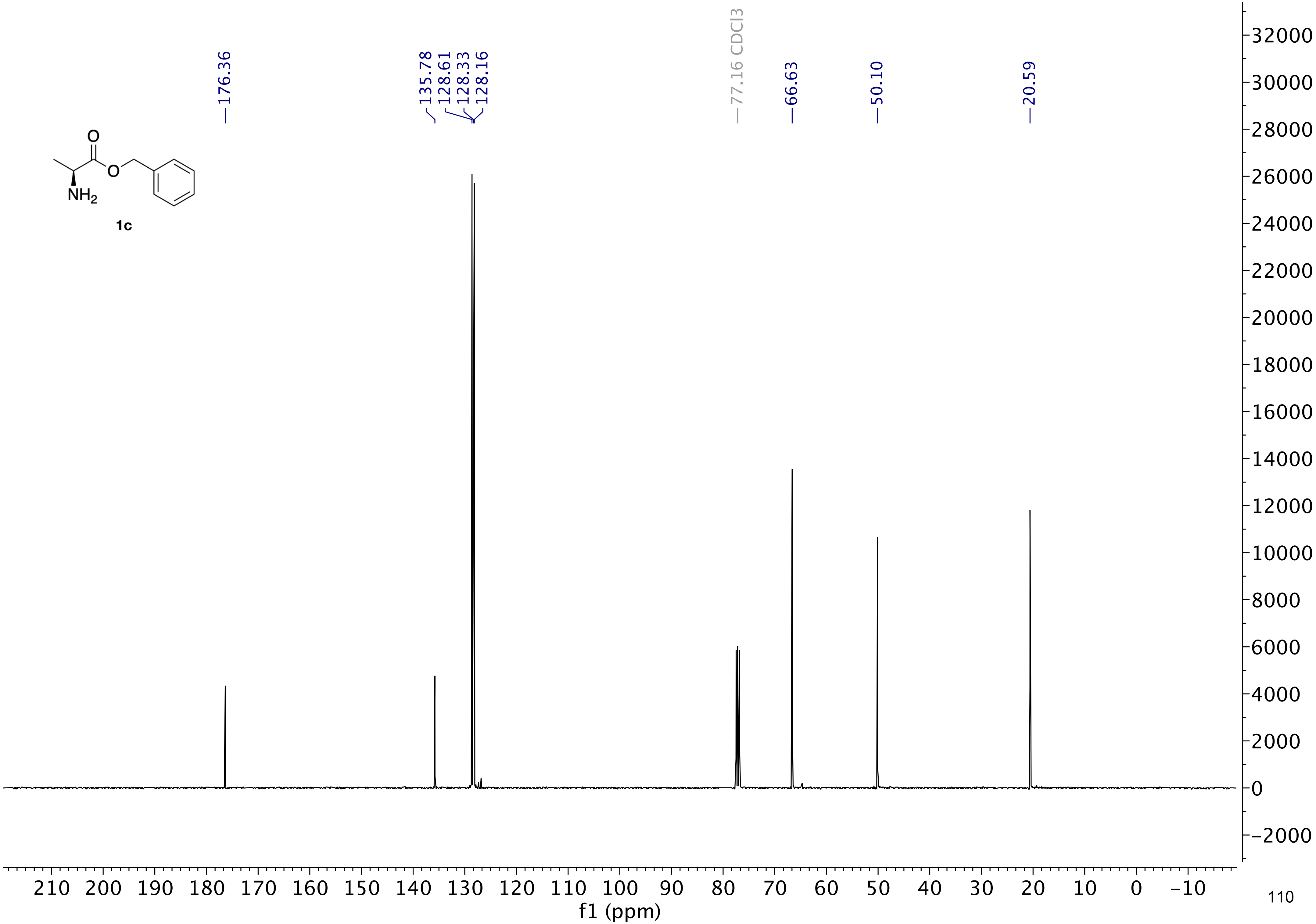

176.36
135.78
128.61
128.33
128.16
77.16 CDCl3
66.63
50.10
20.59
O
O
NH2
1c
f1 (ppm)

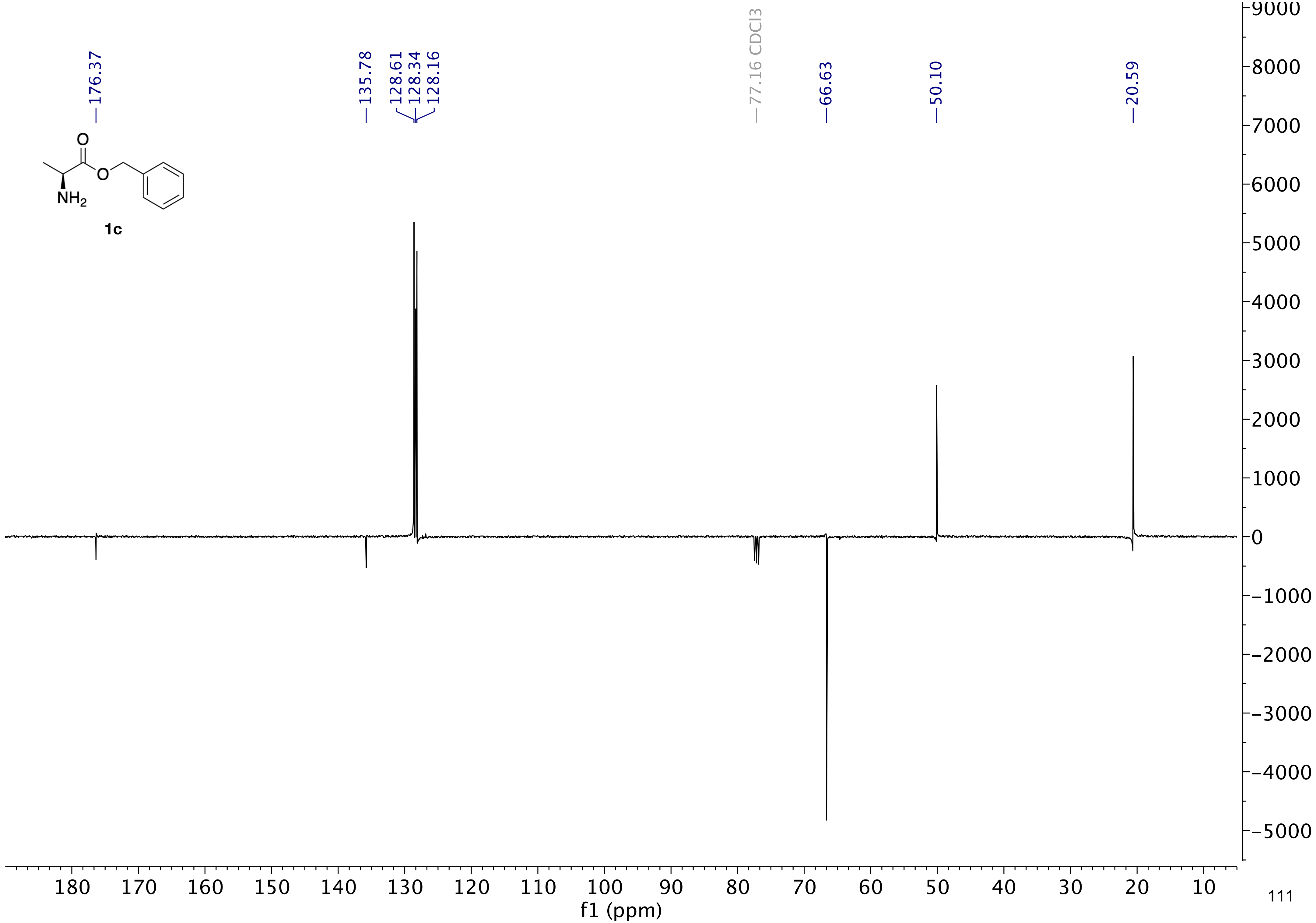

176.37
135.78
128.61
128.34
128.16
77.16 CDCl3
66.63
50.10
20.59
NH2
O
1c
f1 (ppm)

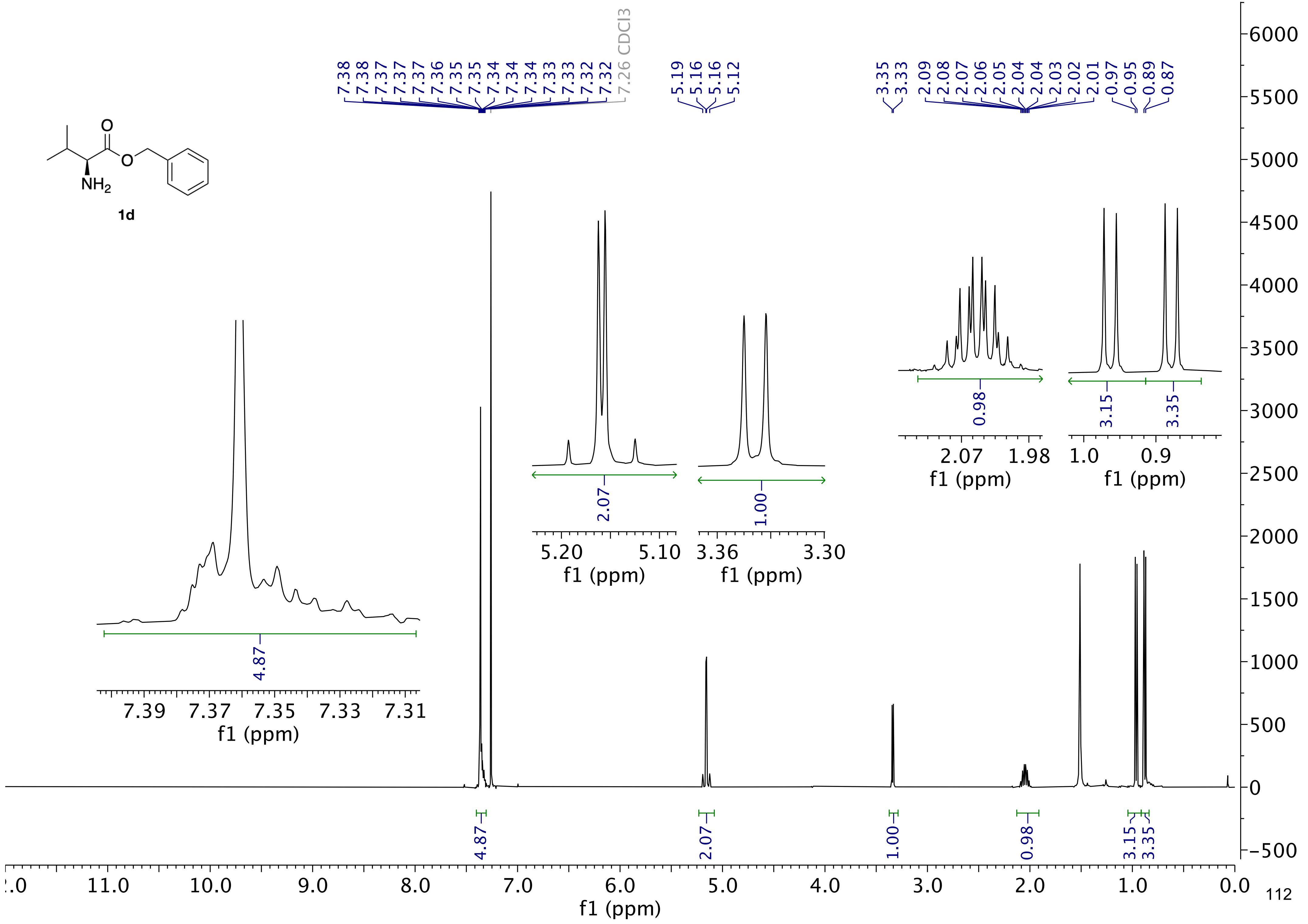
1d
NH2
O
7.38 7.38 7.37 7.37 7.37 7.36 7.35 7.35 7.34 7.34 7.34 7.33 7.33 7.32 7.32
7.26 CDCl3
5.19 5.16 5.16 5.12
3.35 3.33
2.09 2.08 2.07 2.06 2.05 2.04 2.04 2.03 2.02 2.01
0.97 0.95 0.89 0.87
4.87
2.07
1.00
0.98
3.15
3.35
f1 (ppm)

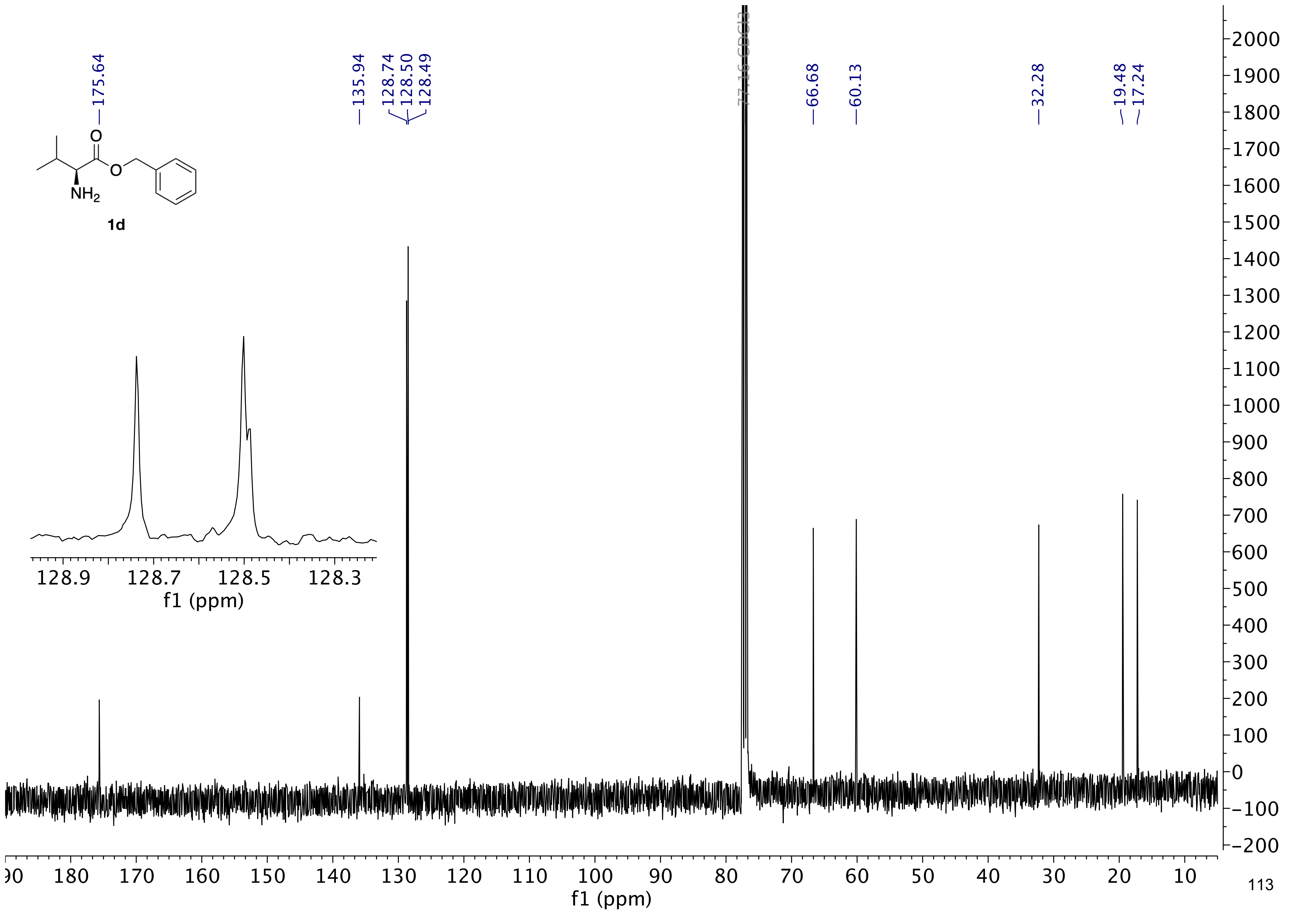
175.64
135.94
128.74
128.50
128.49
77.16 CDCl3
66.68
60.13
32.28
19.48
17.24
O
O
NH2
1d
128.9
128.7
128.5
128.3
f1 (ppm)
2000
1900
1800
1700
1600
1500
1400
1300
1200
1100
1000
900
800
700
600
500
400
300
200
100
0
−100
−200
180
170
160
150
140
130
120
110
100
90
80
70
60
50
40
30
20
10
f1 (ppm)

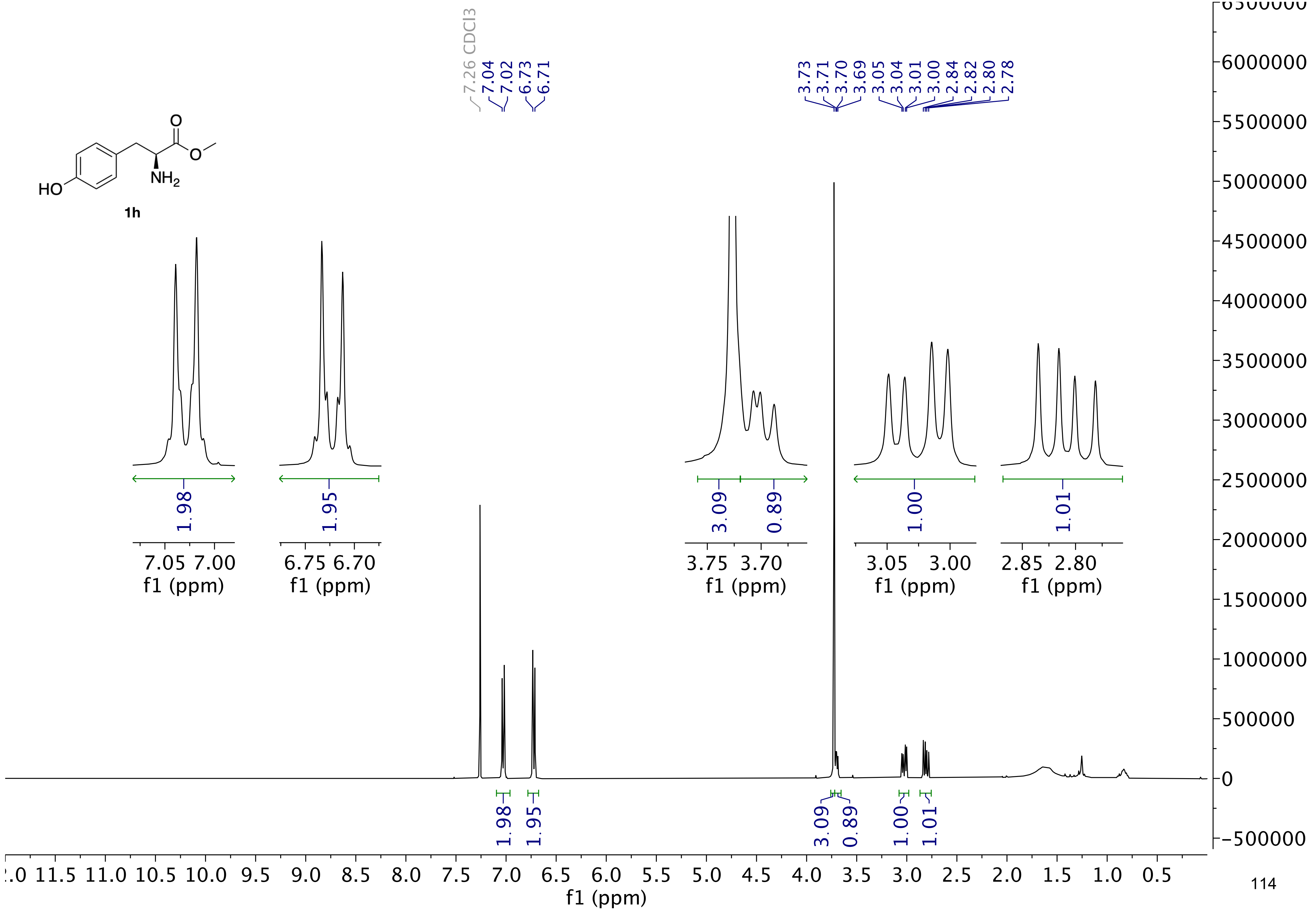
7.26 CDCl3
7.04
7.02
6.73
6.71
3.73
3.71
3.70
3.69
3.05
3.04
3.01
3.00
2.84
2.82
2.80
2.78
HO
O
O
NH2
1h
1.98
1.95
3.09
0.89
1.00
1.01
7.05 7.00
6.75 6.70
3.75 3.70
3.05 3.00
2.85 2.80
f1 (ppm)
11.5 11.0 10.5 10.0 9.5 9.0 8.5 8.0 7.5 7.0 6.5 6.0 5.5 5.0 4.5 4.0 3.5 3.0 2.5 2.0 1.5 1.0 0.5
6000000
5500000
5000000
4500000
4000000
3500000
3000000
2500000
2000000
1500000
1000000
500000
0
-500000

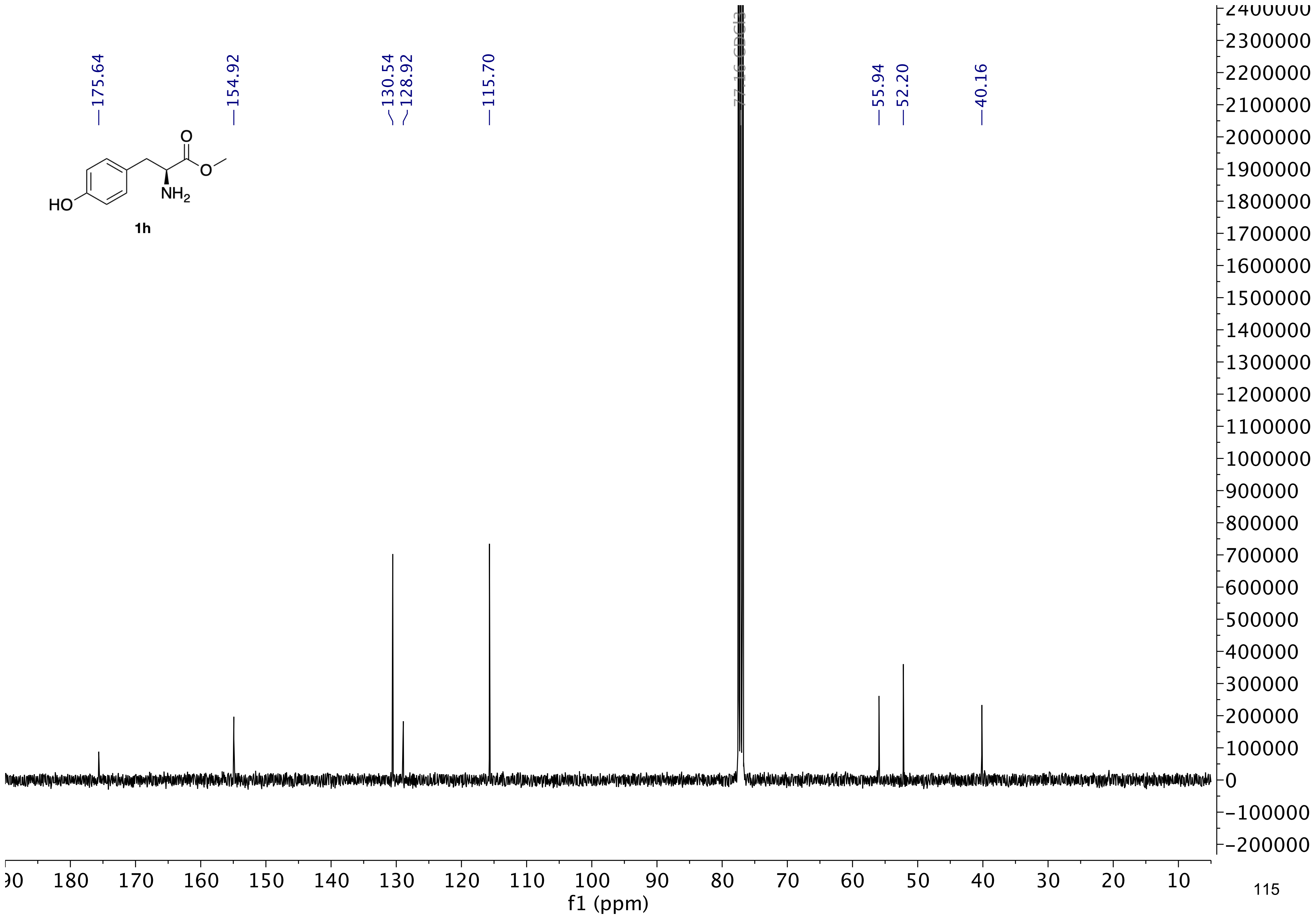

175.64
154.92
130.54
128.92
115.70
77.16 CDCl3
55.94
52.20
40.16
O
O
NH2
HO
1h
f1 (ppm)

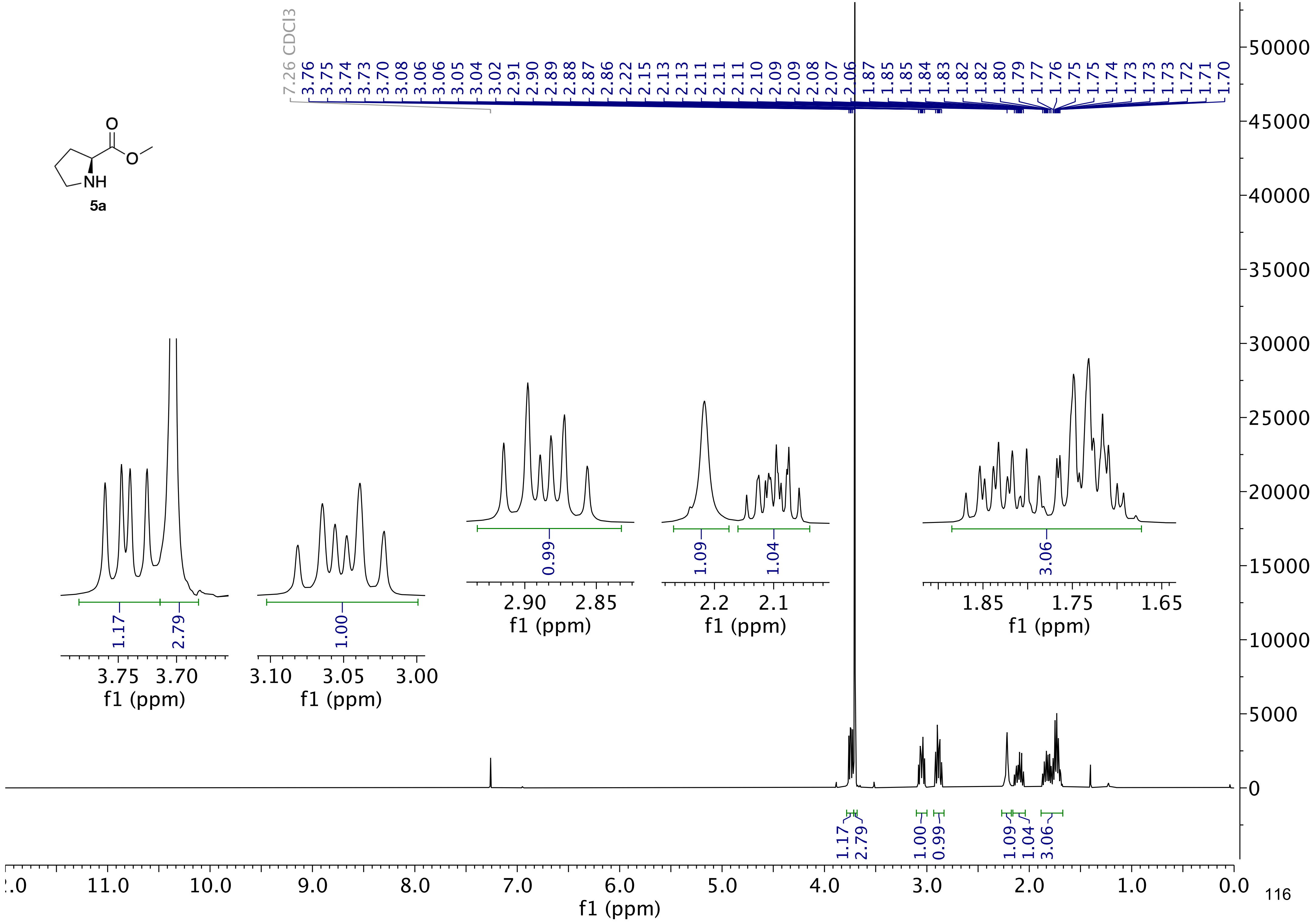

5a
7.26 CDCl3
3.76 3.75 3.74 3.73 3.70 3.08 3.06 3.06 3.05 3.04 3.02 2.91 2.90 2.89 2.88 2.87 2.86 2.22 2.15 2.13 2.13 2.11 2.11 2.11 2.10 2.09 2.09 2.08 2.07 2.06 1.87 1.85 1.85 1.84 1.83 1.82 1.82 1.80 1.79 1.77 1.76 1.75 1.75 1.74 1.73 1.73 1.73 1.72 1.71 1.70
1.17 2.79 1.00 0.99 1.09 1.04 3.06
f1 (ppm)

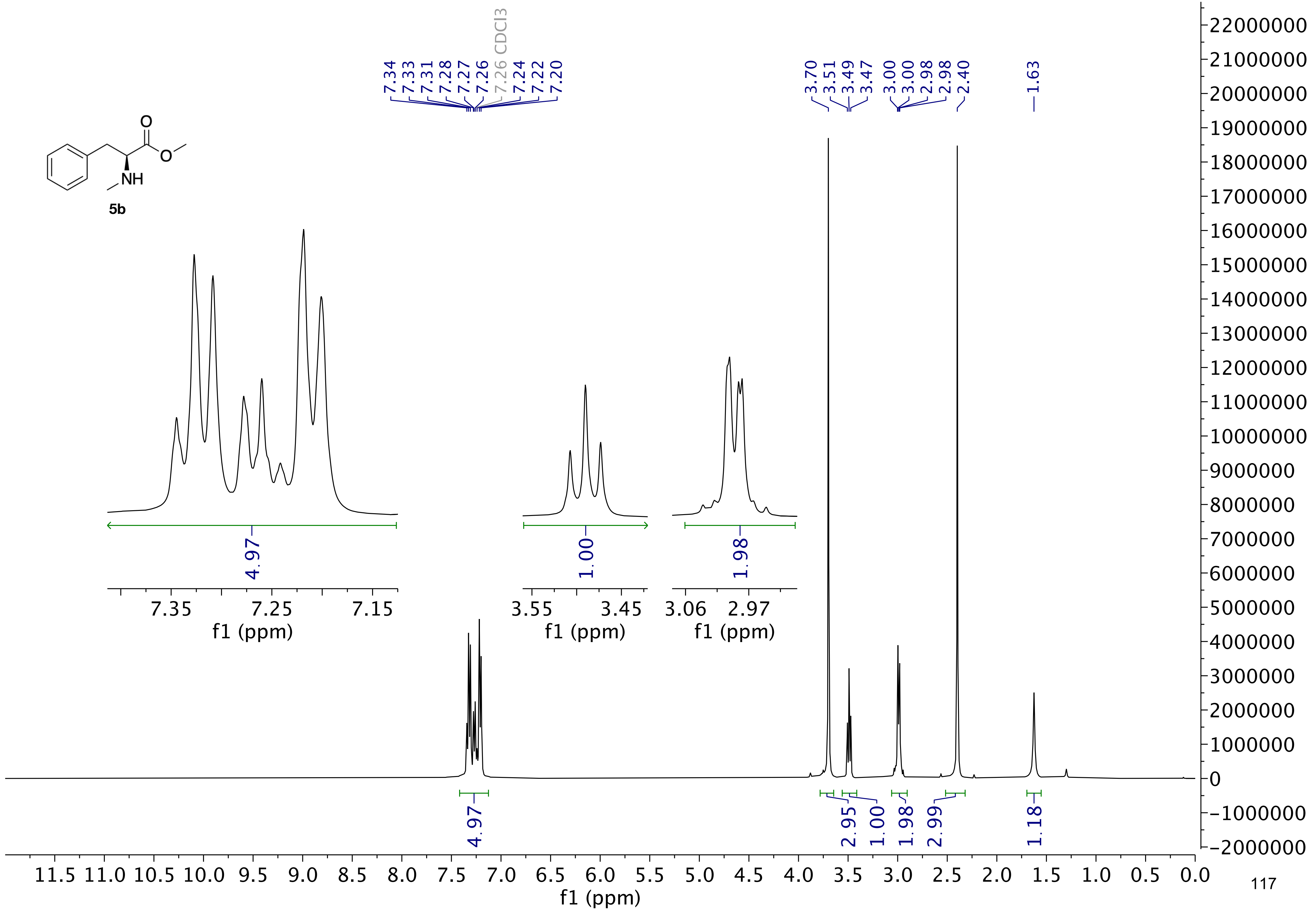
7.34
7.33
7.31
7.28
7.27
7.26
7.26 CDCl3
7.24
7.22
7.20
3.70
3.51
3.49
3.47
3.00
3.00
2.98
2.98
2.40
1.63
4.97
1.00
1.98
2.95
2.99
1.18
f1 (ppm)

5b

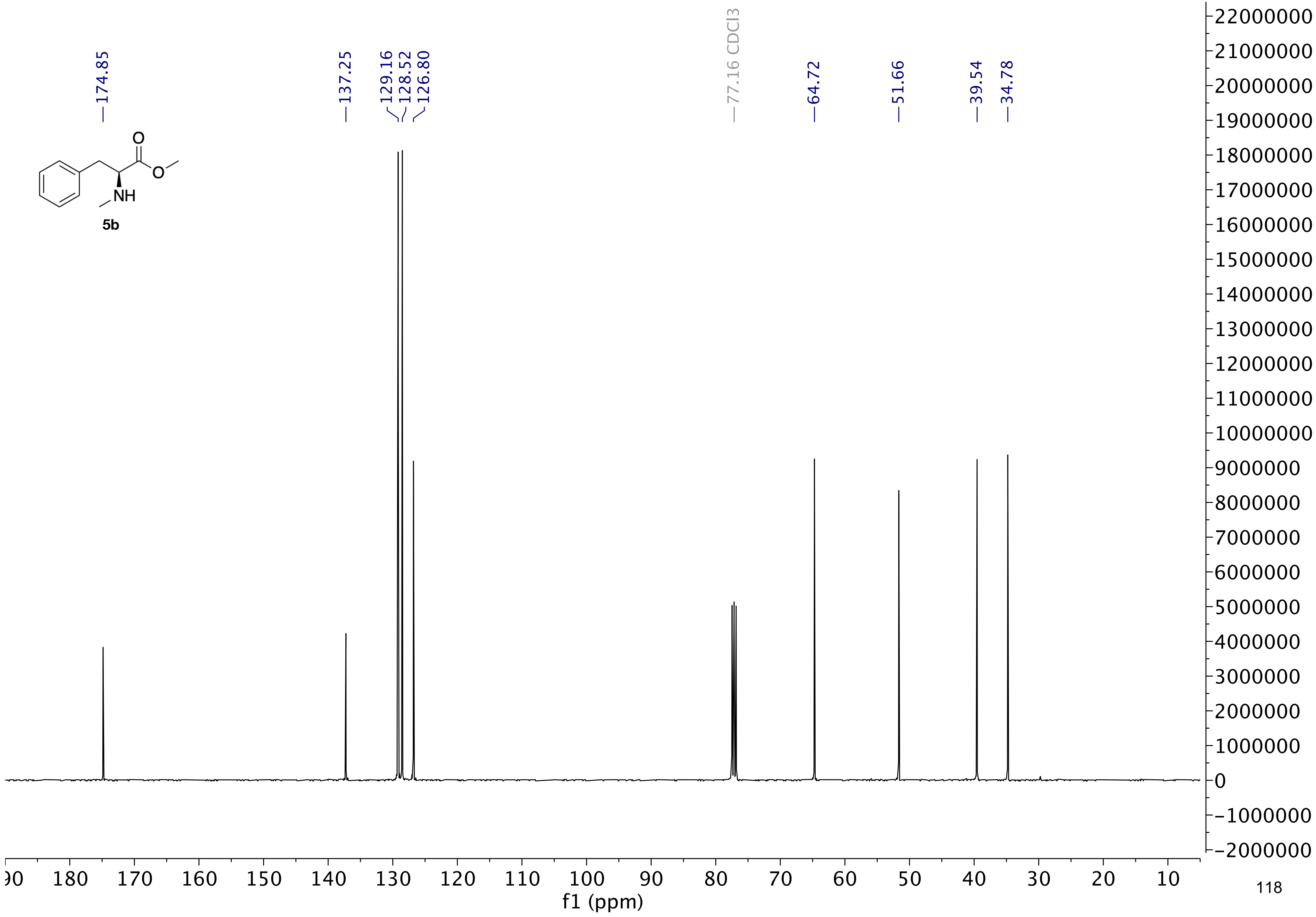
174.85
137.25
129.16
128.52
126.80
77.16 CDCl3
64.72
51.66
39.54
34.78
O
O
NH
5b
f1 (ppm)

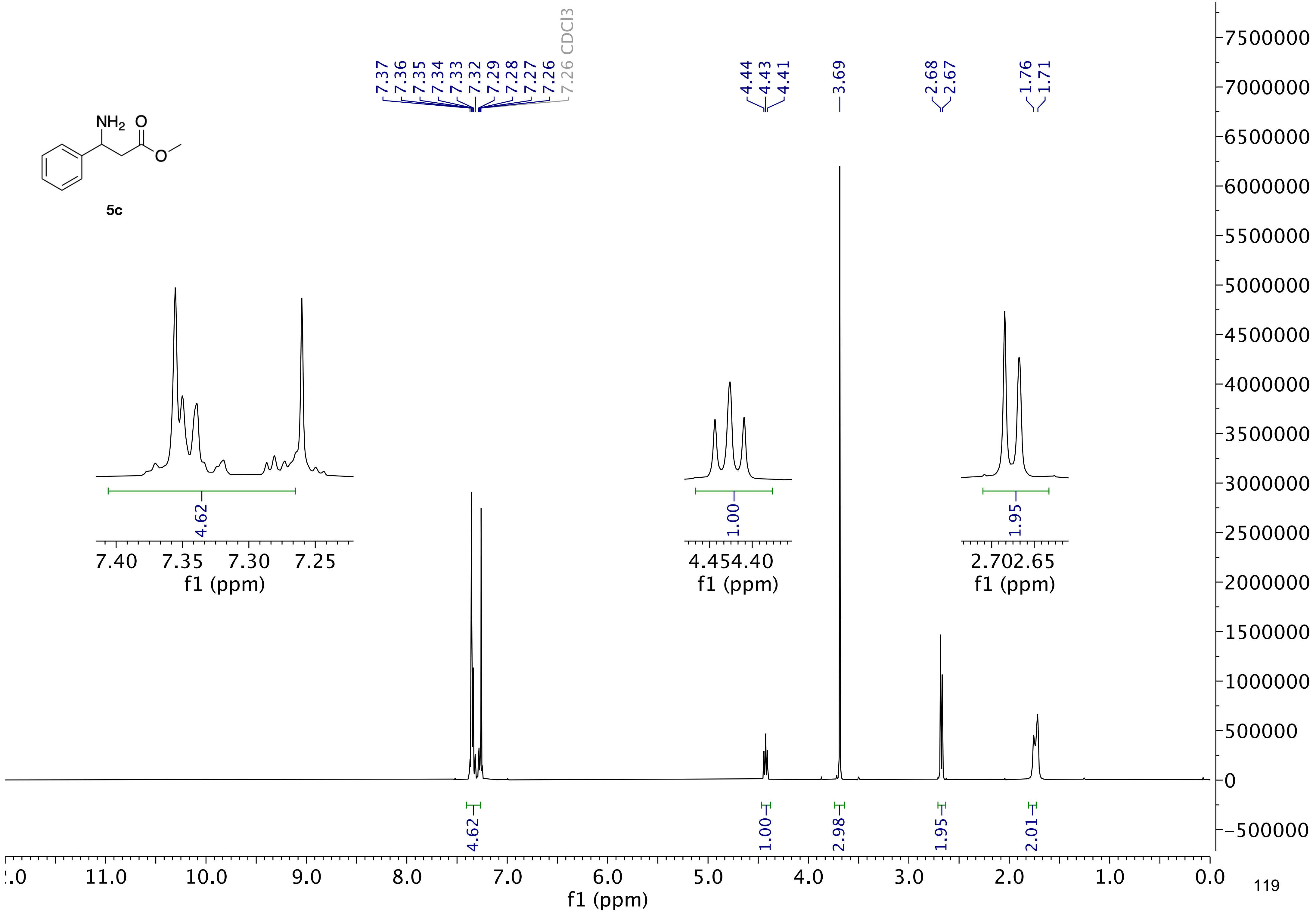

NH2 O
O
5c
7.37 7.36 7.35 7.34 7.33 7.32 7.29 7.28 7.27 7.26
7.26 CDCl3
4.44 4.43 4.41
3.69
2.68 2.67
1.76 1.71
4.62
1.00
2.98
1.95
2.01
f1 (ppm)

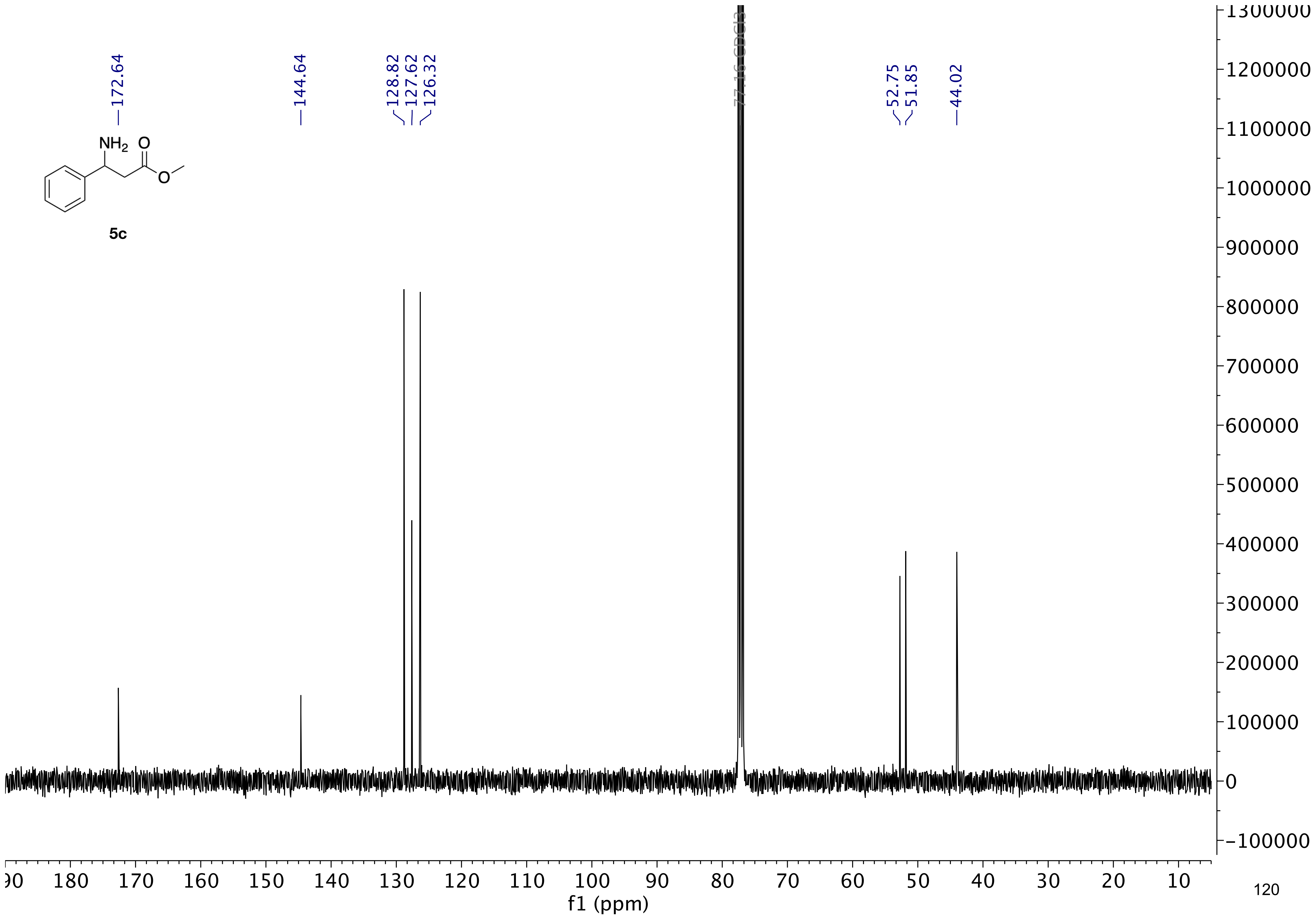
172.64
144.64
128.82
127.62
126.32
77.16 CDCl3
52.75
51.85
44.02
NH2
O
O
5c
f1 (ppm)

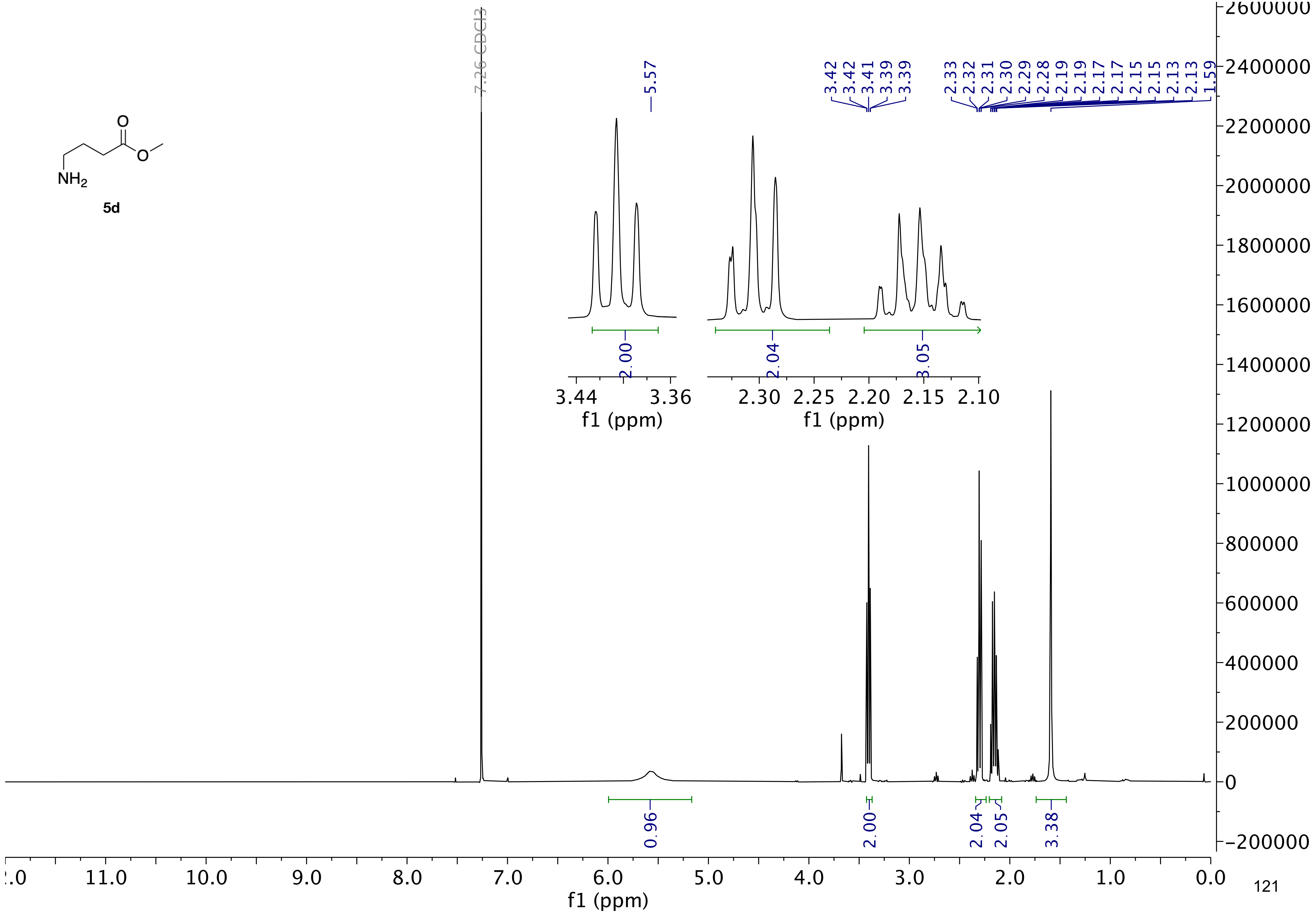
O
O
NH2
5d
7.26 CDCl3
5.57
3.42
3.42
3.41
3.39
3.39
2.33
2.32
2.31
2.30
2.29
2.28
2.19
2.19
2.17
2.17
2.15
2.15
2.13
2.13
1.59
2.00
2.04
3.05
3.44
3.36
2.30 2.25 2.20 2.15 2.10
f1 (ppm)
0.96
2.00
2.04
2.05
3.38
11.0 10.0 9.0 8.0 7.0 6.0 5.0 4.0 3.0 2.0 1.0 0.0
f1 (ppm)
2600000
2400000
2200000
2000000
1800000
1600000
1400000
1200000
1000000
800000
600000
400000
200000
0
−200000

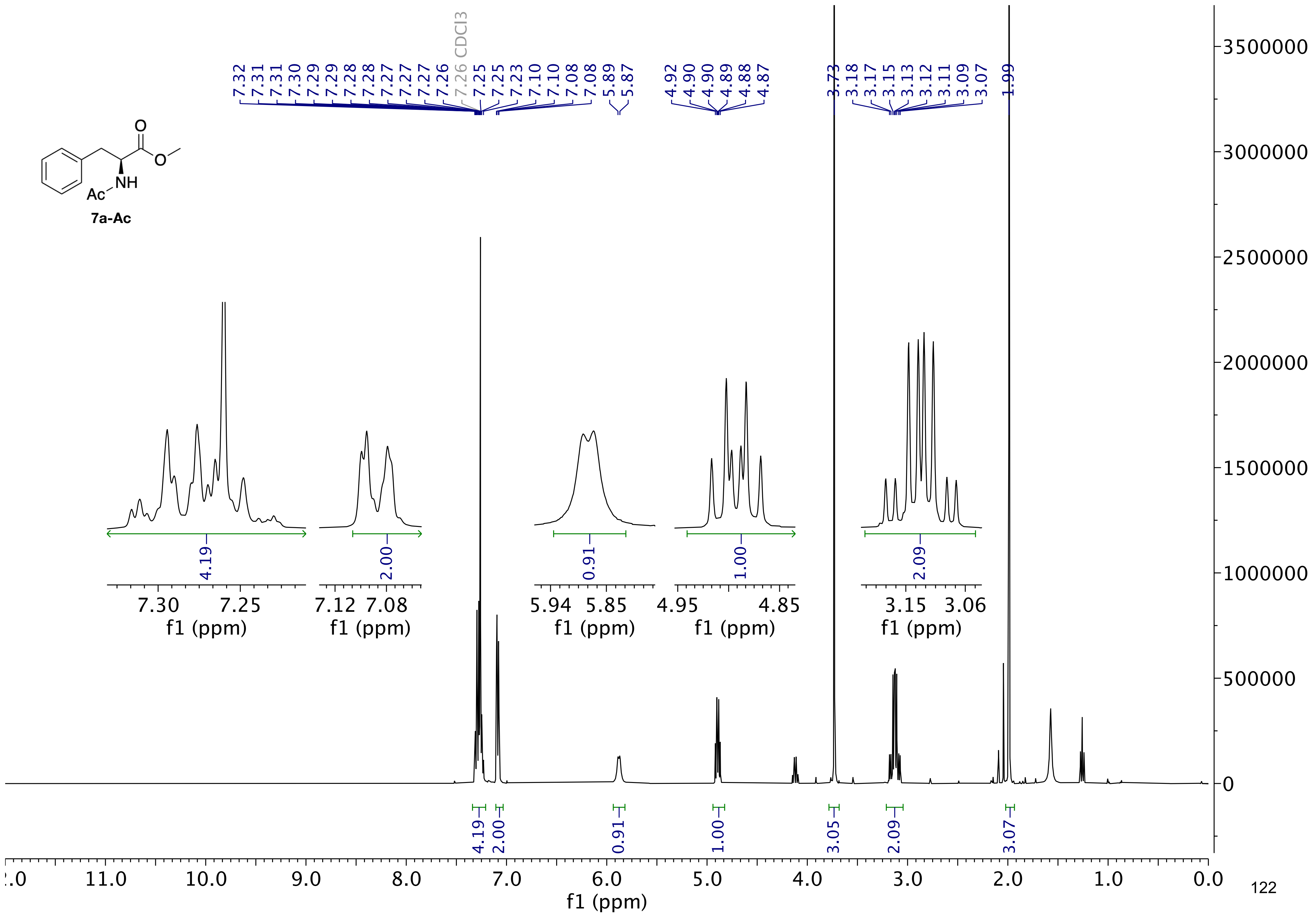

O
O
NH
Ac
7a-Ac
7.32 7.31 7.31 7.30 7.29 7.29 7.28 7.28 7.27 7.27 7.27 7.26 7.26 CDCl3 7.25 7.25 7.23 7.10 7.10 7.08 7.08 5.89 5.87
4.92 4.90 4.90 4.89 4.88 4.87
3.73 3.18 3.17 3.15 3.13 3.12 3.11 3.09 3.07
1.99
4.19 2.00 0.91 1.00 3.05 2.09 3.07
f1 (ppm)

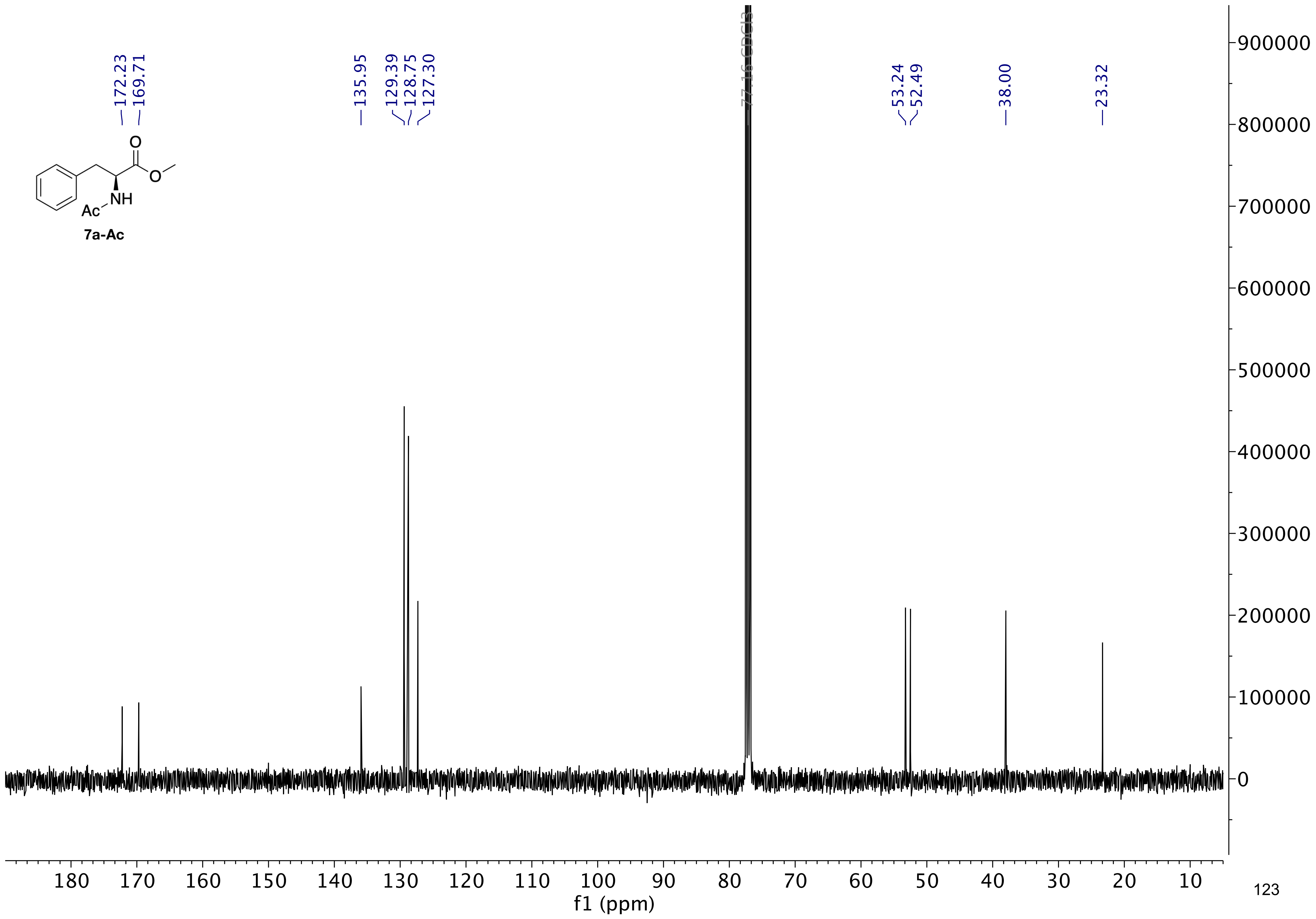

172.23
169.71
135.95
129.39
128.75
127.30
77.16 CDCl3
53.24
52.49
38.00
23.32
O
O
NH
Ac
7a-Ac
900000
800000
700000
600000
500000
400000
300000
200000
100000
0
180
170
160
150
140
130
120
110
100
90
80
70
60
50
40
30
20
10
f1 (ppm)

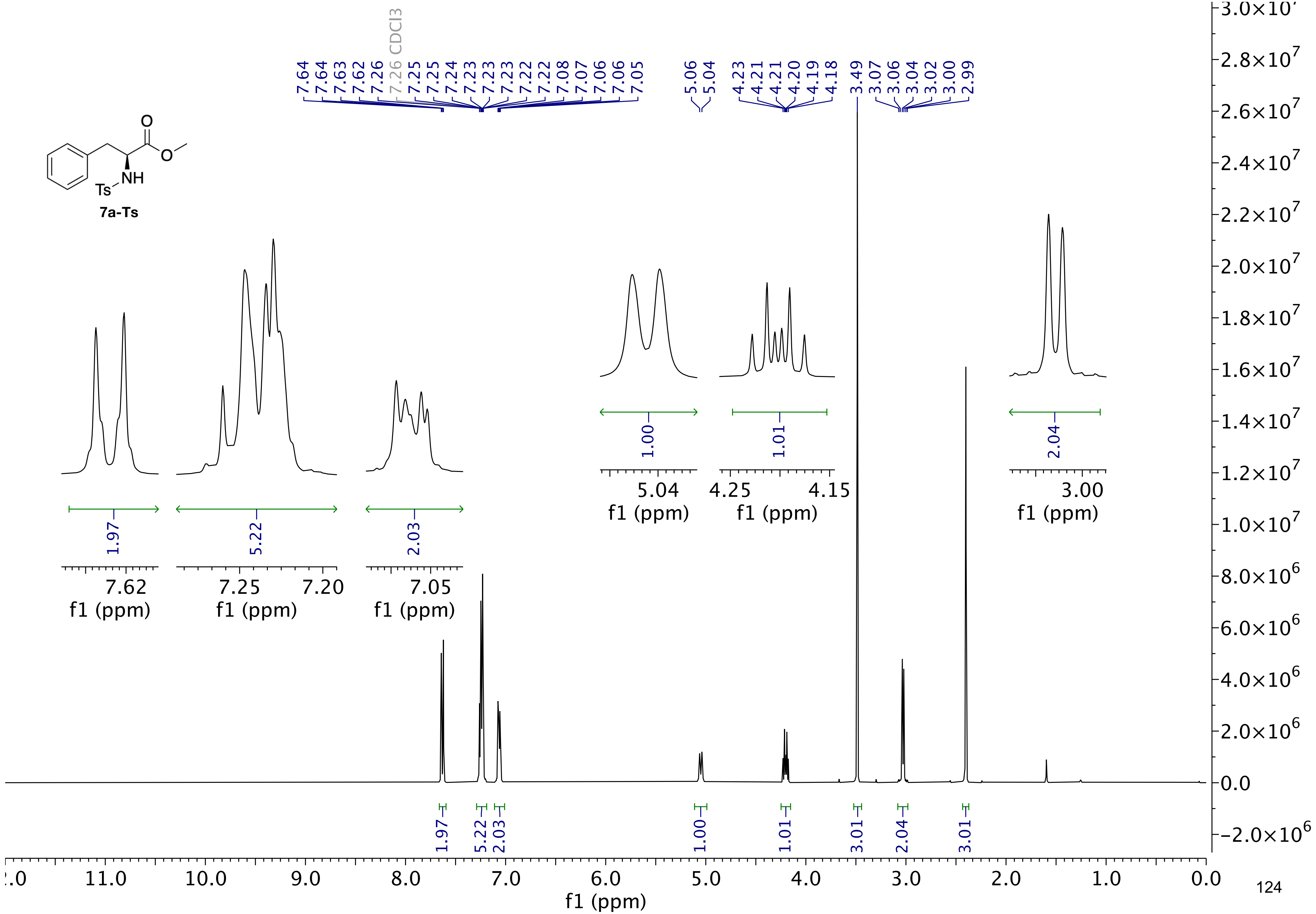

7a-Ts
f1 (ppm)

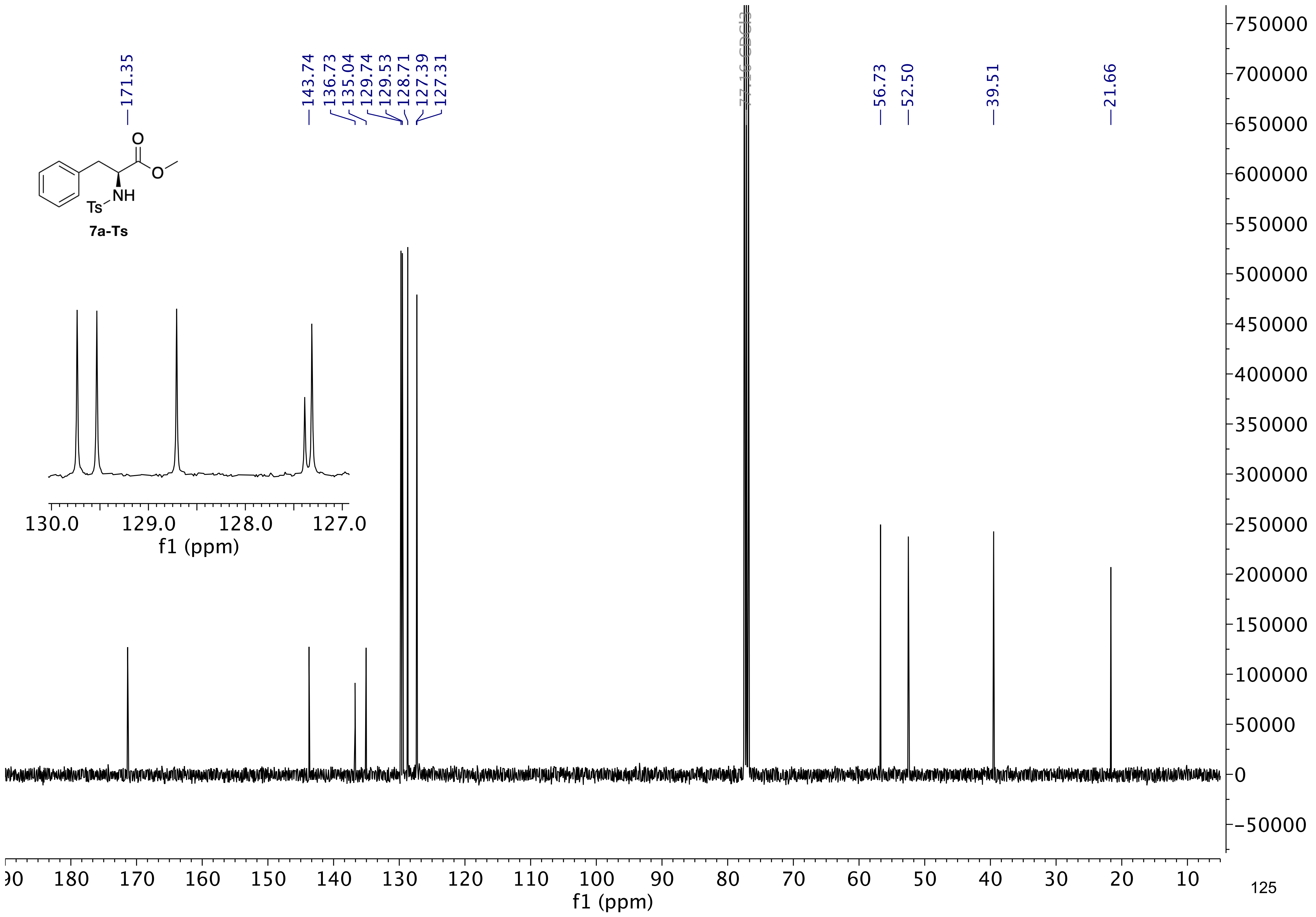
171.35
143.74
136.73
135.04
129.74
129.53
128.71
127.39
127.31
77.16 CDCl3
56.73
52.50
39.51
21.66
O
O
NH
Ts
7a-Ts
130.0
129.0
128.0
127.0
f1 (ppm)
750000
700000
650000
600000
550000
500000
450000
400000
350000
300000
250000
200000
150000
100000
50000
0
−50000
180
170
160
150
140
130
120
110
100
90
80
70
60
50
40
30
20
10
f1 (ppm)

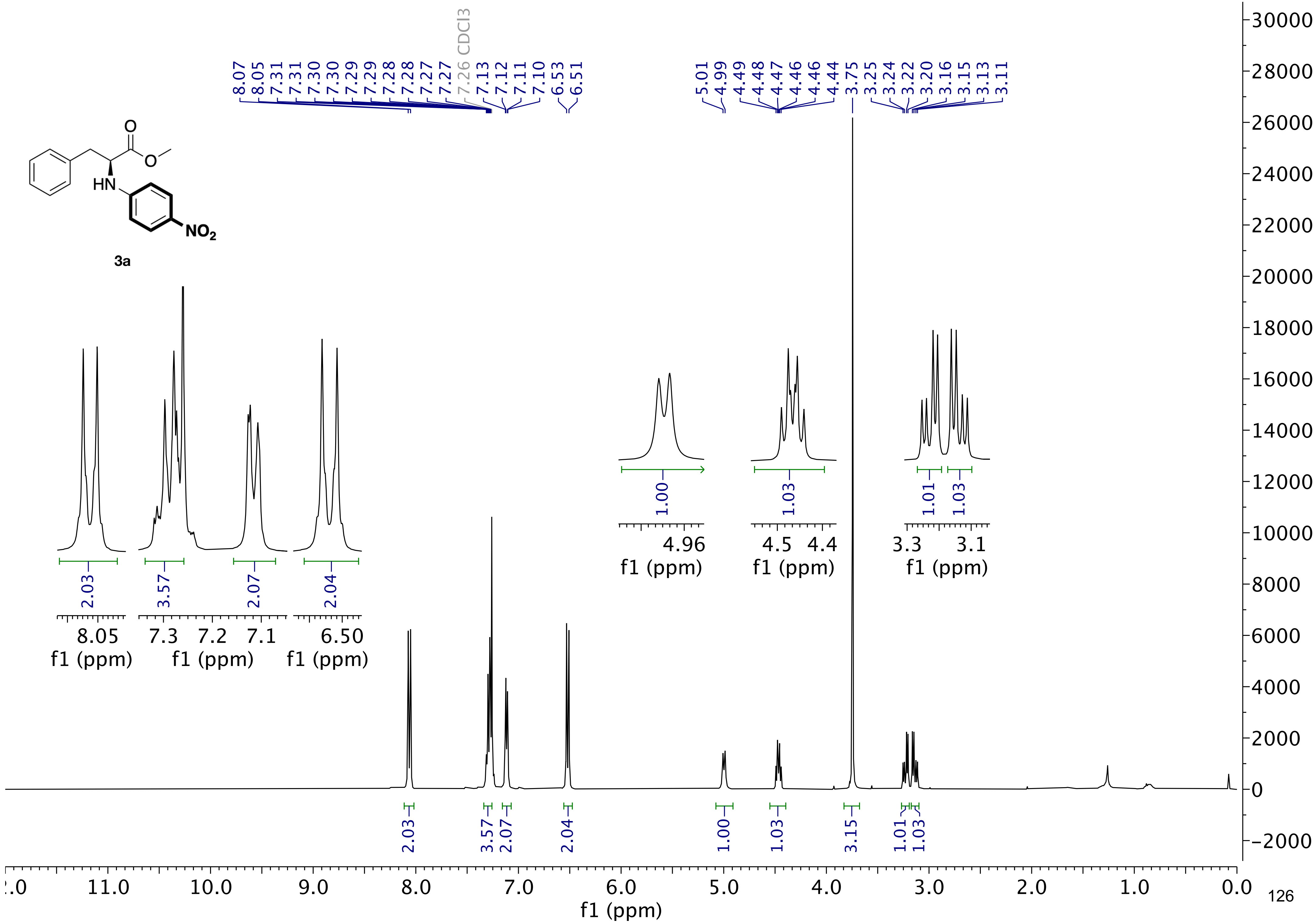
3a
f1 (ppm)

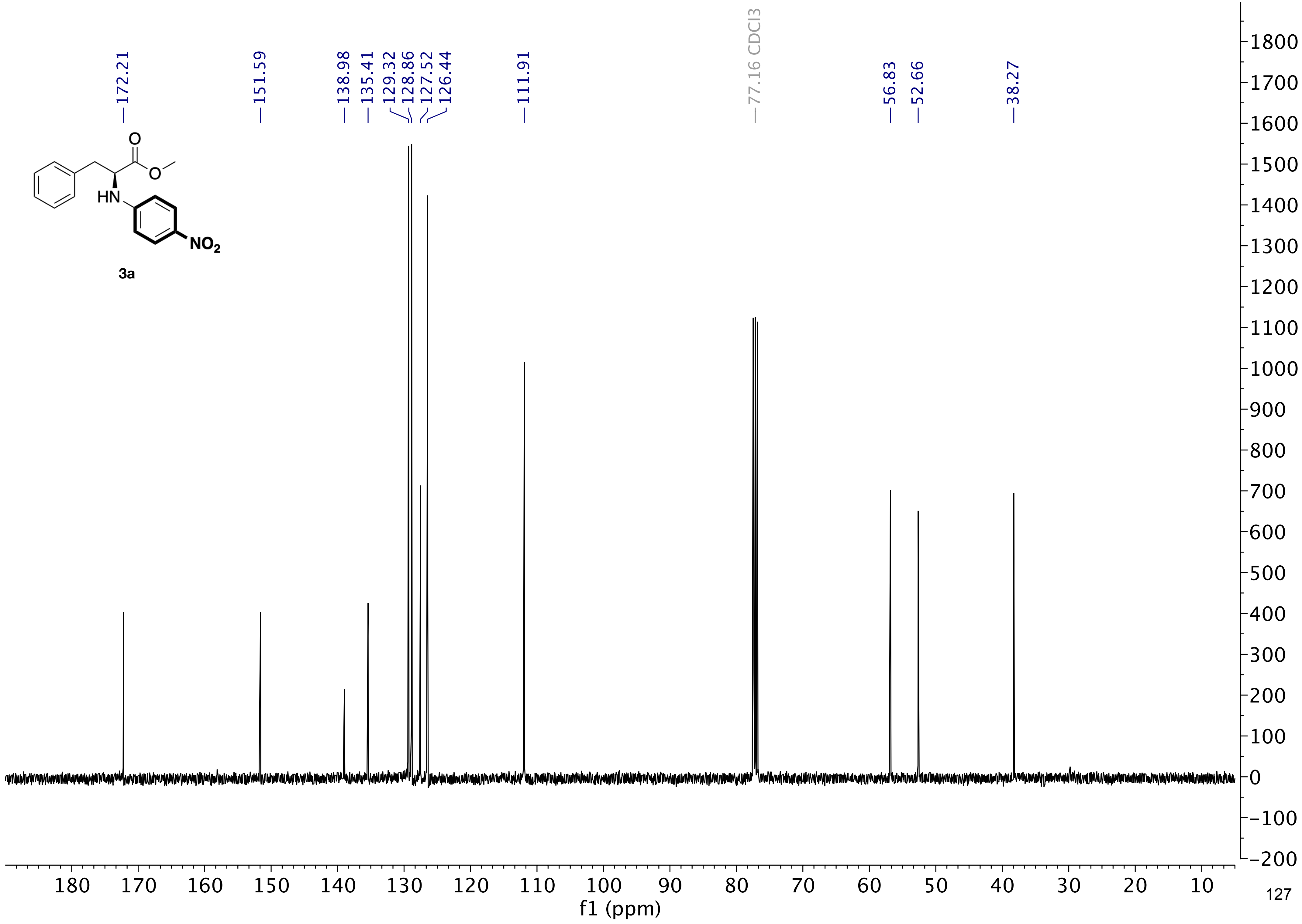
172.21
151.59
138.98
135.41
129.32
128.86
127.52
126.44
111.91
77.16 CDCl3
56.83
52.66
38.27
HN
O
O
NO2
3a
f1 (ppm)

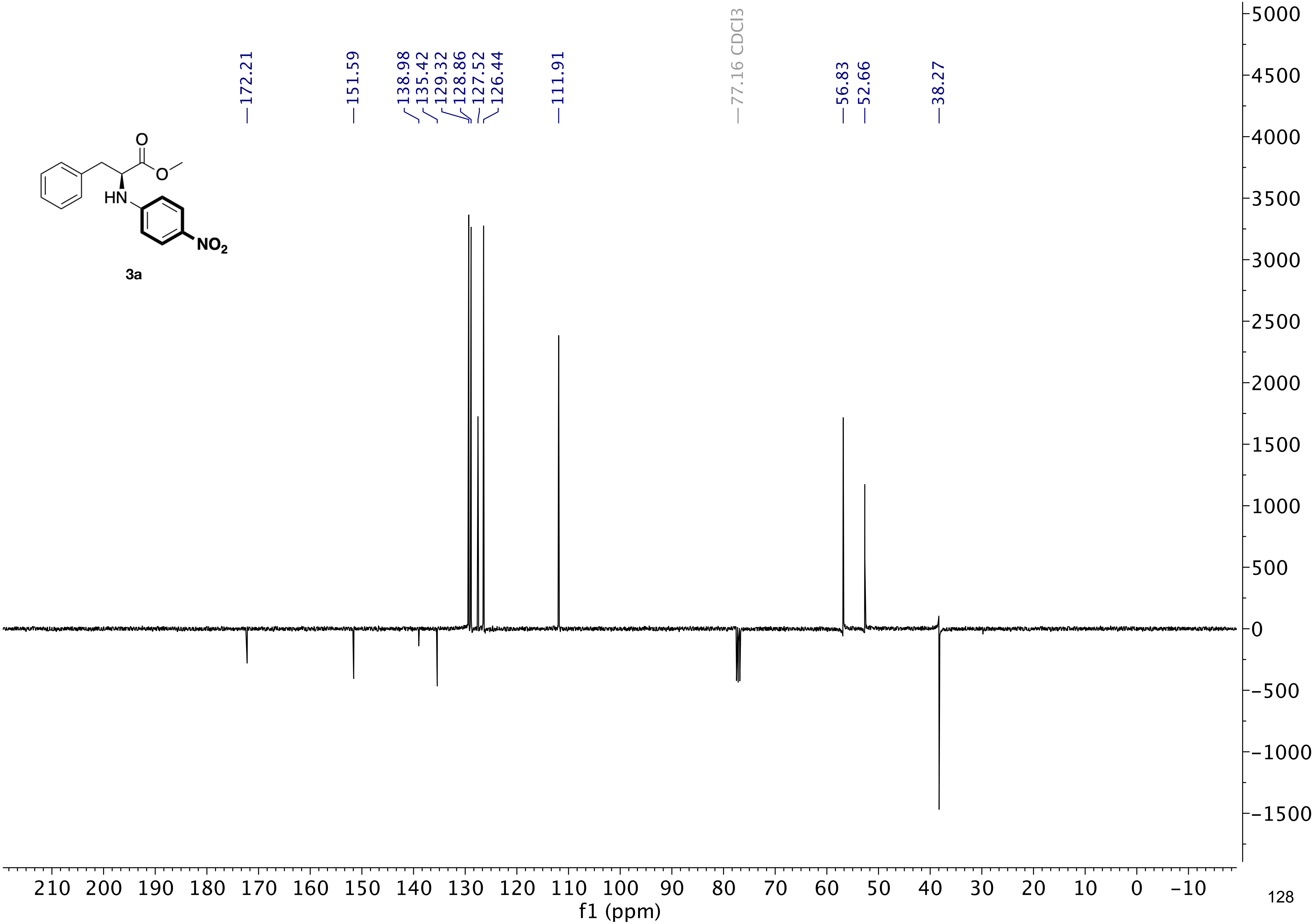
172.21
151.59
138.98
135.42
129.32
128.86
127.52
126.44
111.91
77.16 CDCl3
56.83
52.66
38.27
O
O
HN
NO_2
3a
5000
4500
4000
3500
3000
2500
2000
1500
1000
500
0
−500
−1000
−1500
210 200 190 180 170 160 150 140 130 120 110 100 90 80 70 60 50 40 30 20 10 0 −10
f1 (ppm)

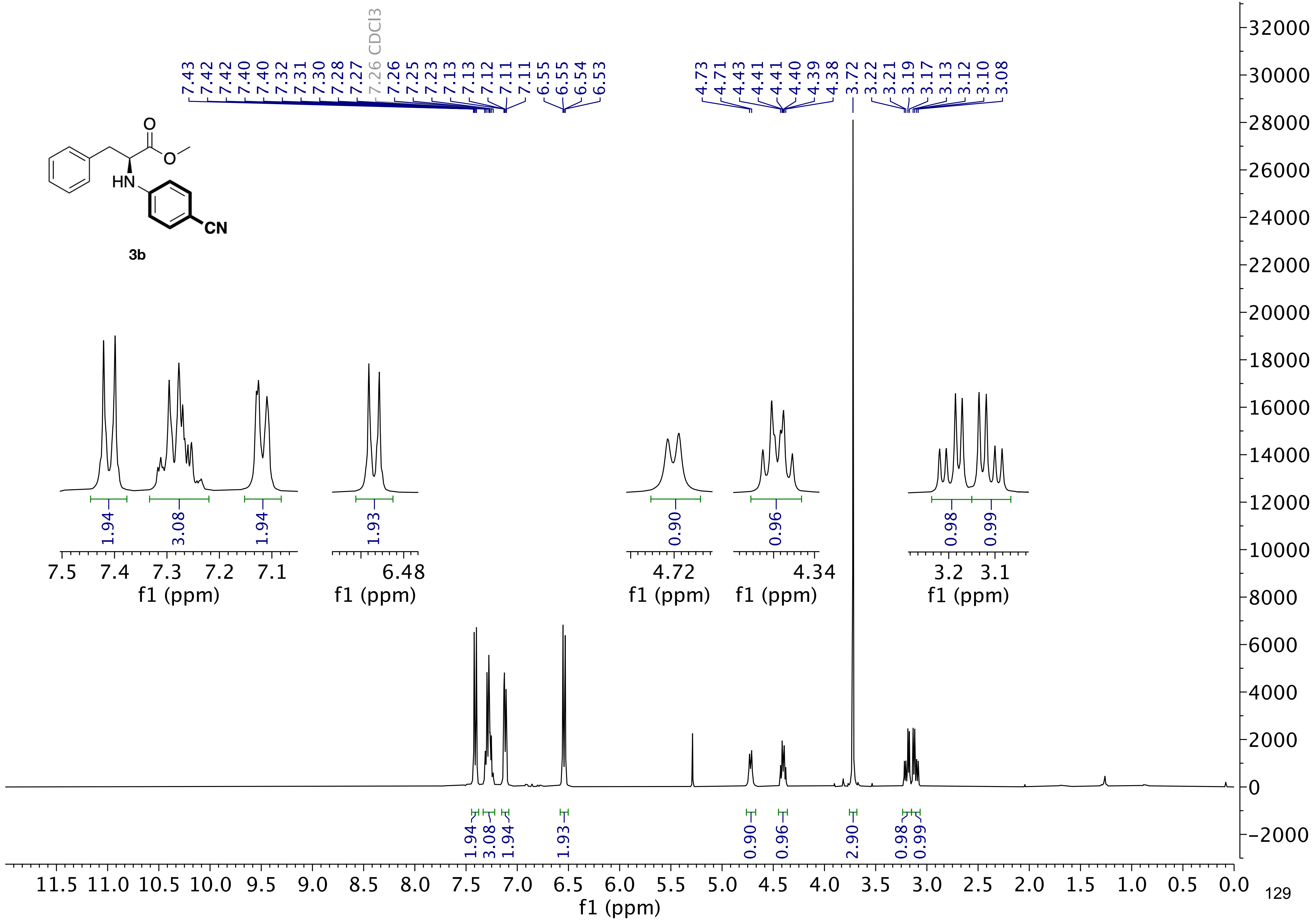
7.43 7.42 7.42 7.40 7.40 7.32 7.31 7.30 7.28 7.27 7.26 CDCl3 7.26 7.25 7.23 7.13 7.13 7.12 7.11 7.11 6.55 6.55 6.54 6.53
4.73 4.71 4.43 4.41 4.41 4.40 4.39 4.38 3.72 3.22 3.21 3.19 3.17 3.13 3.12 3.10 3.08
O
O
HN
CN
3b
1.94 3.08 1.94 1.93 0.90 0.96 2.90 0.98 0.99
f1 (ppm)

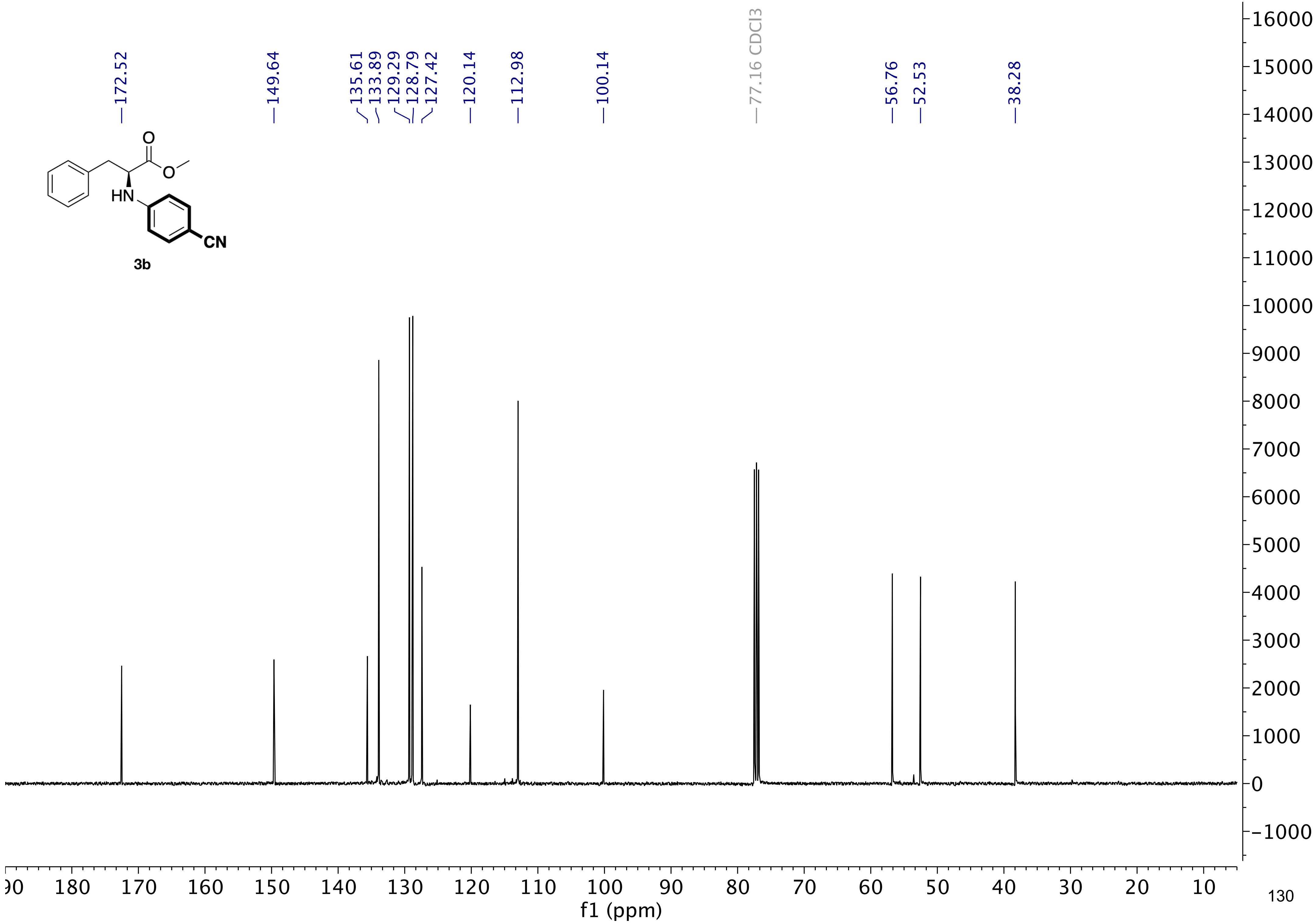
172.52
149.64
135.61
133.89
129.29
128.79
127.42
120.14
112.98
100.14
77.16 CDCl3
56.76
52.53
38.28
O
O
HN
CN
3b
f1 (ppm)

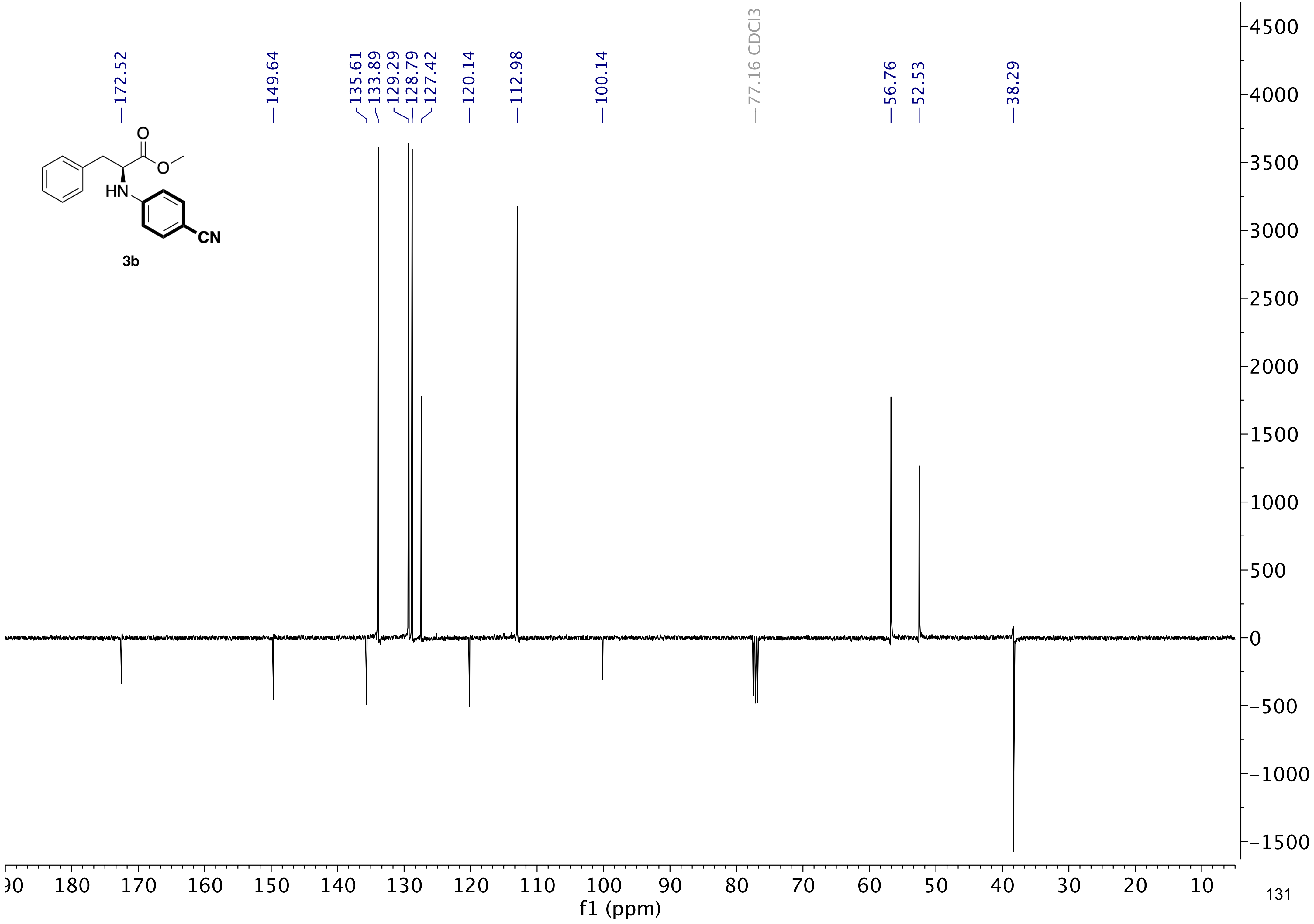

172.52
149.64
135.61
133.89
129.29
128.79
127.42
120.14
112.98
100.14
77.16 CDCl3
56.76
52.53
38.29
O
O
HN
CN
3b
4500
4000
3500
3000
2500
2000
1500
1000
500
0
−500
−1000
−1500
190 180 170 160 150 140 130 120 110 100 90 80 70 60 50 40 30 20 10
f1 (ppm)

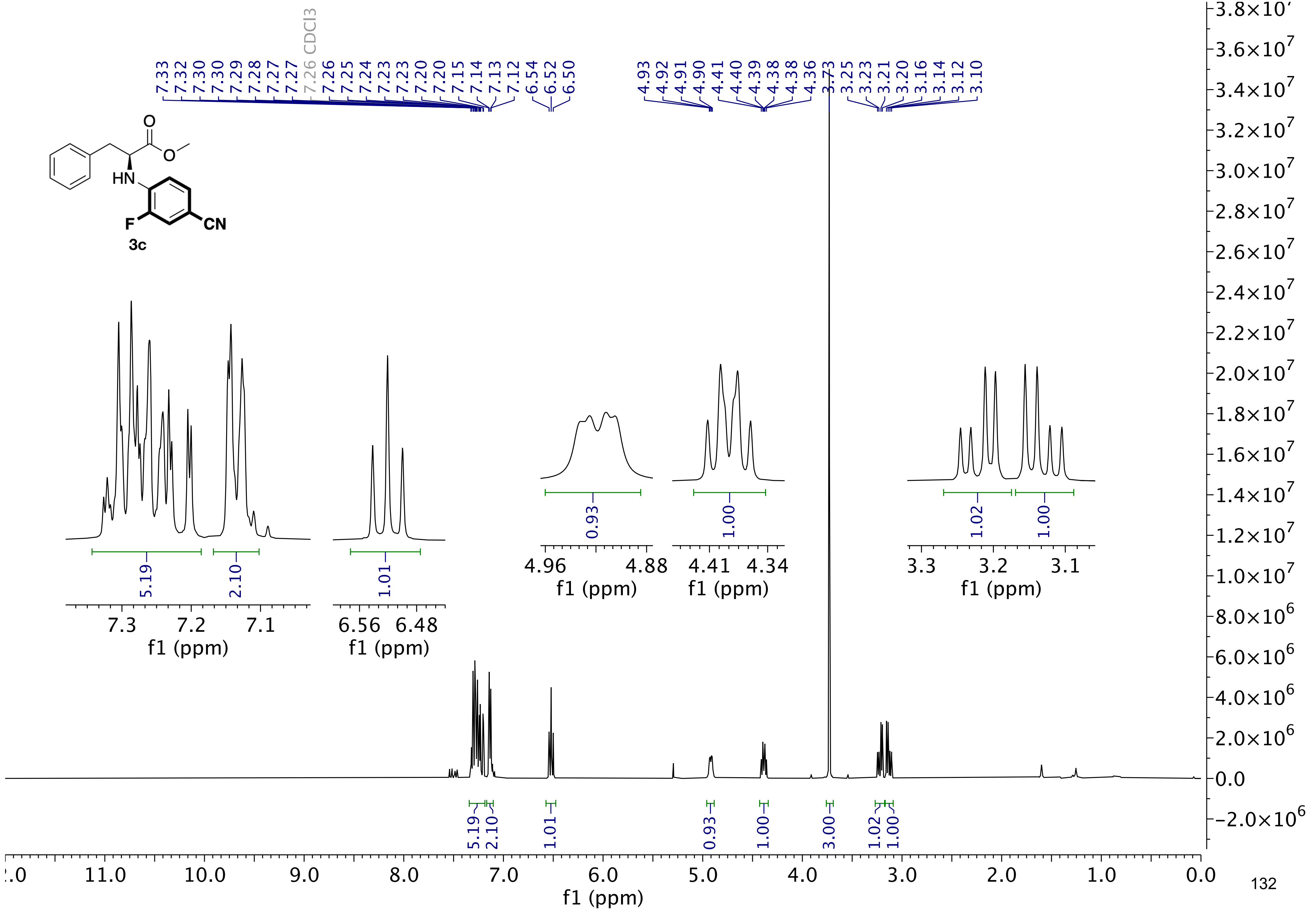

7.33
7.32
7.30
7.30
7.29
7.28
7.27
7.27
7.26 CDCl3
7.26
7.25
7.24
7.23
7.23
7.20
7.20
7.15
7.14
7.13
7.12
6.54
6.52
6.50
4.93
4.92
4.91
4.90
4.41
4.40
4.39
4.38
4.38
4.36
3.73
3.25
3.23
3.21
3.20
3.16
3.14
3.12
3.10
O
O
HN
F
CN
3c
5.19
2.10
1.01
0.93
1.00
3.00
1.02
1.00
f1 (ppm)

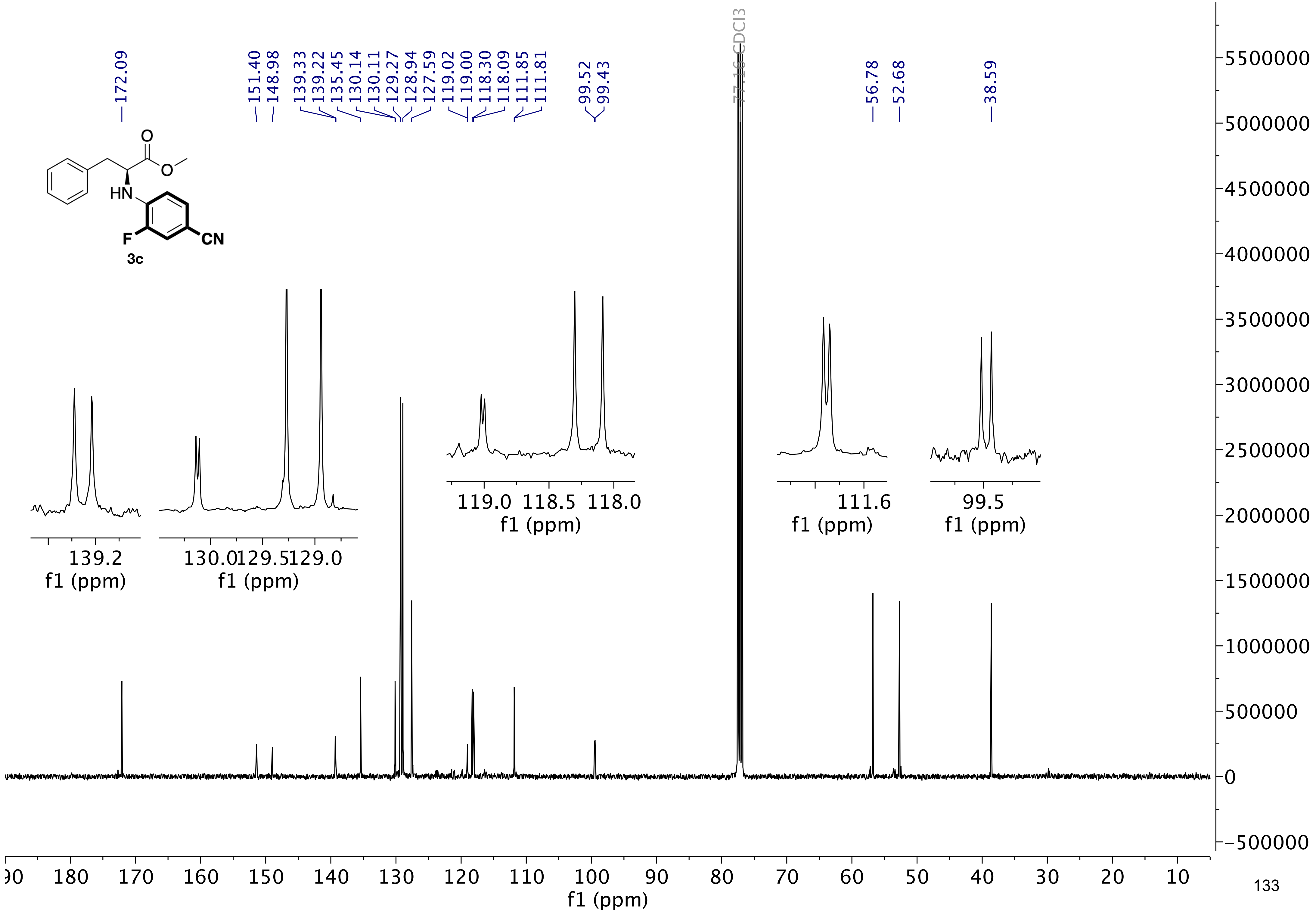
172.09
151.40
148.98
139.33
139.22
135.45
130.14
130.11
129.27
128.94
127.59
119.02
119.00
118.30
118.09
111.85
111.81
99.52
99.43
77.16 CDCl3
56.78
52.68
38.59
HN
O
O
F
CN
3c
f1 (ppm)

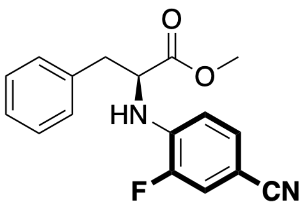
O
O
HN
F
CN

3c

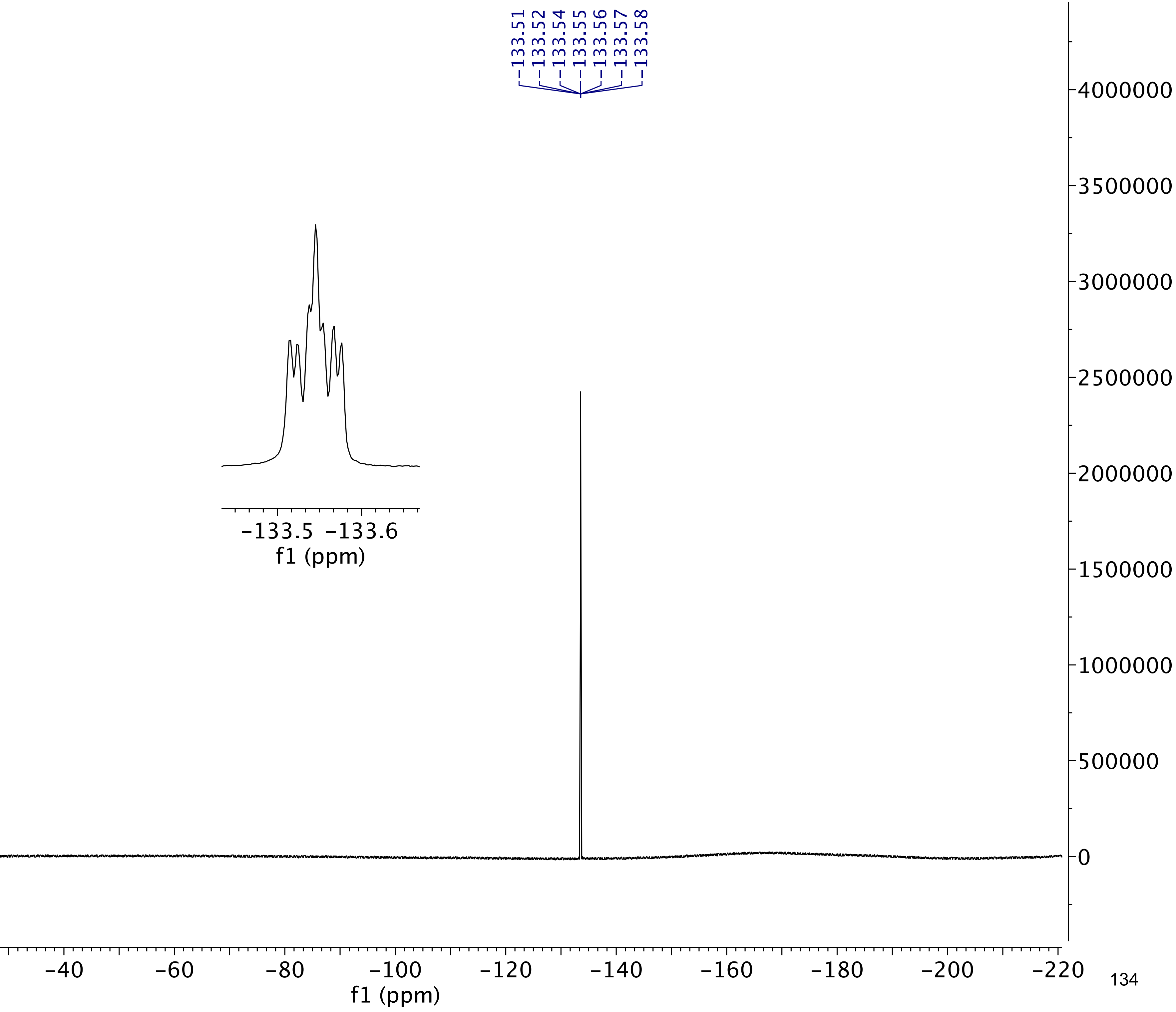
-133.51
-133.52
-133.54
-133.55
-133.56
-133.57
-133.58
-133.5 -133.6
f1 (ppm)
4000000
3500000
3000000
2500000
2000000
1500000
1000000
500000
0
0 -20 -40 -60 -80 -100 -120 -140 -160 -180 -200 -220
f1 (ppm)

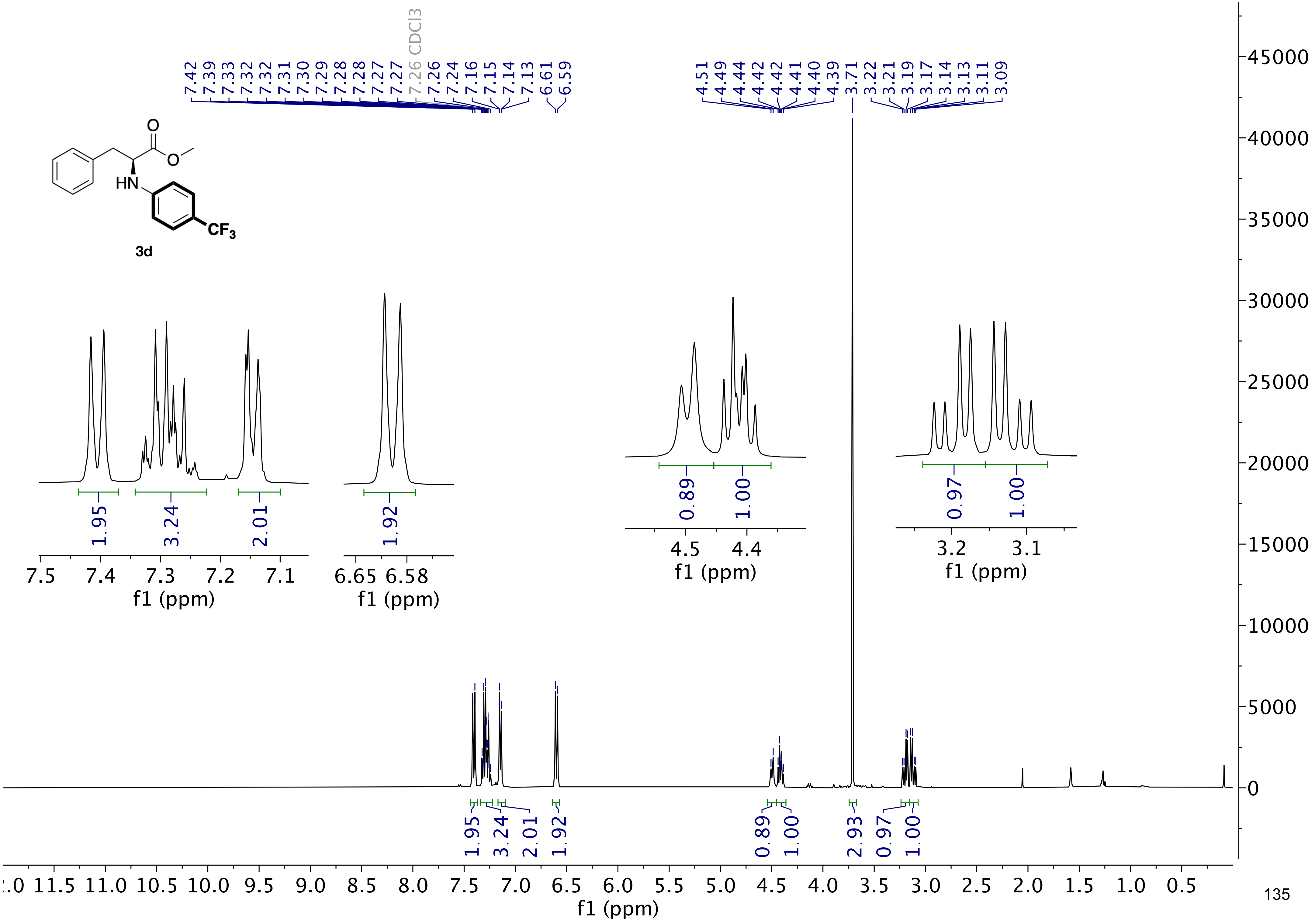
7.42 7.39 7.33 7.32 7.32 7.31 7.30 7.29 7.28 7.28 7.27 7.27 7.26 CDCl3 7.26 7.24 7.16 7.15 7.14 7.13 6.61 6.59
4.51 4.49 4.44 4.42 4.42 4.41 4.40 4.39 3.71 3.22 3.21 3.19 3.17 3.14 3.13 3.11 3.09
HN
O
CF3
3d
1.95
3.24
2.01
1.92
0.89
1.00
2.93
0.97
1.00
f1 (ppm)

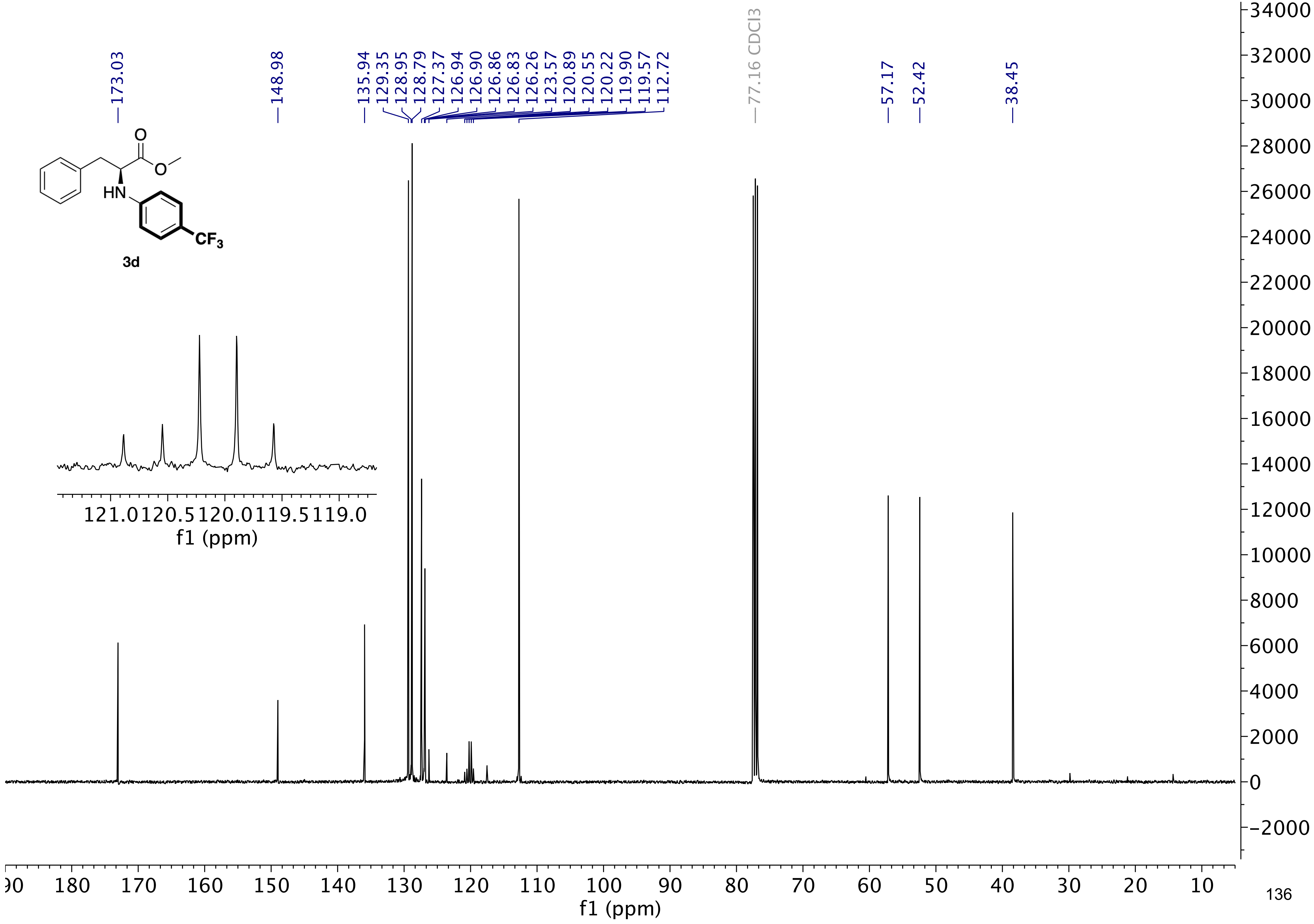
173.03
148.98
135.94
129.35
128.95
128.79
127.37
126.94
126.90
126.86
126.83
126.26
123.57
120.89
120.55
120.22
119.90
119.57
112.72
77.16 CDCl3
57.17
52.42
38.45
HN
O
O
CF3
3d
121.0 120.5 120.0 119.5 119.0
f1 (ppm)
f1 (ppm)

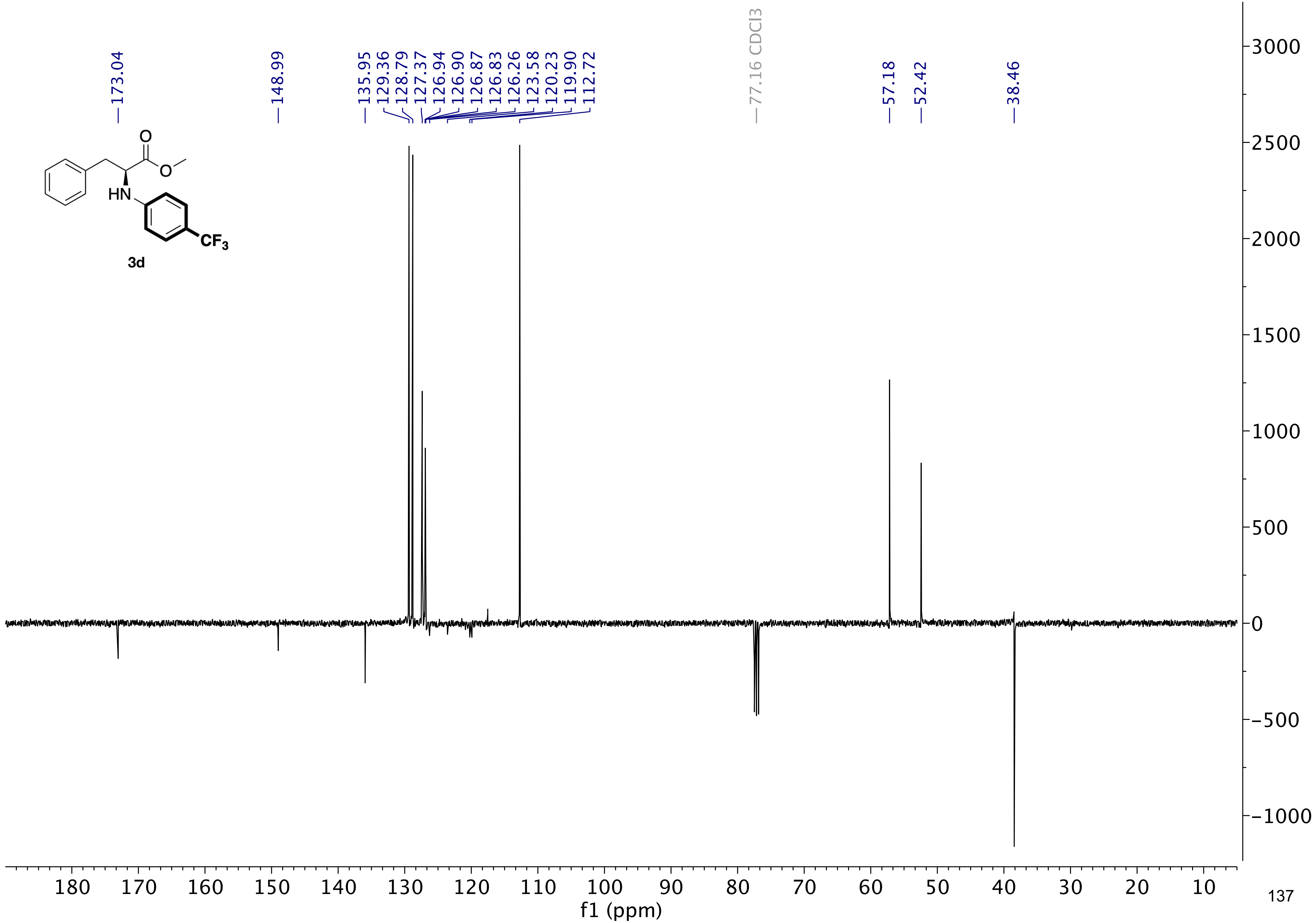

173.04
148.99
135.95
129.36
128.79
127.37
126.94
126.90
126.87
126.83
126.26
123.58
120.23
119.90
112.72
77.16 CDCl3
57.18
52.42
38.46
O
O
HN
CF3
3d
3000
2500
2000
1500
1000
500
0
−500
−1000
180
170
160
150
140
130
120
110
100
90
80
70
60
50
40
30
20
10
f1 (ppm)

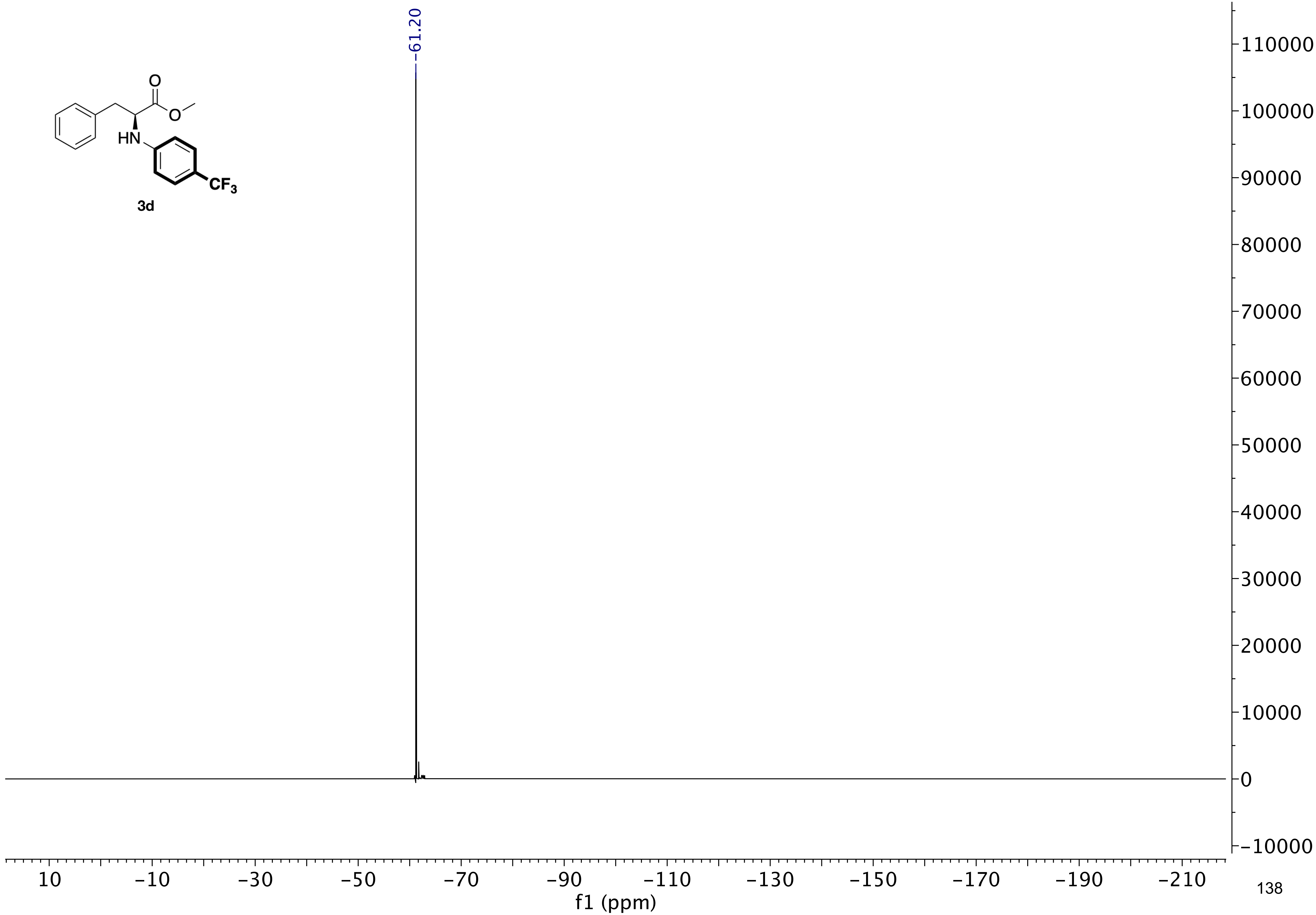
-61.20
HN
O
O
CF3
3d
110000
100000
90000
80000
70000
60000
50000
40000
30000
20000
10000
0
-10000
10
-10
-30
-50
-70
-90
-110
-130
-150
-170
-190
-210
f1 (ppm)

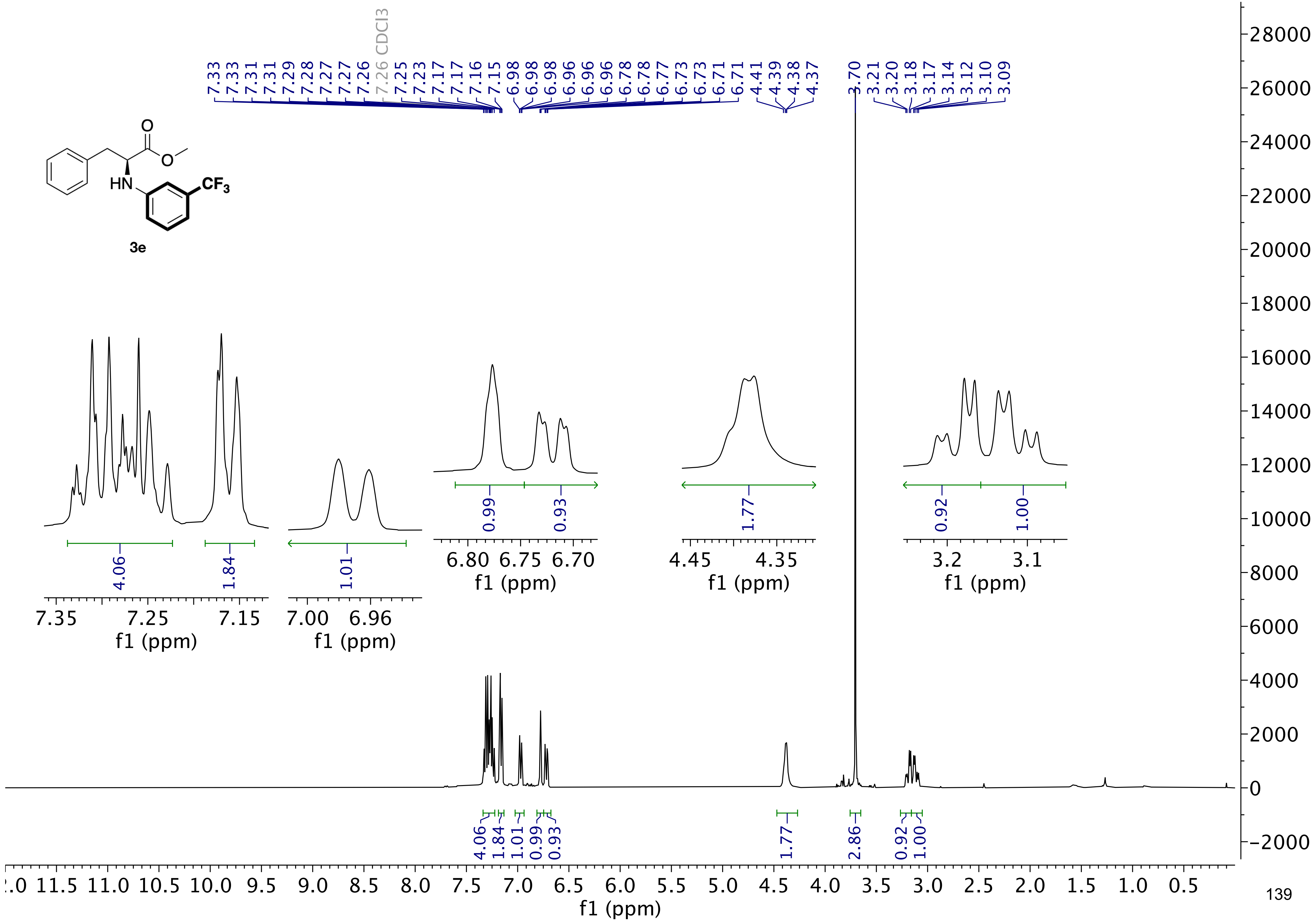
7.33 7.33 7.31 7.31 7.29 7.28 7.27 7.27 7.26 7.26 CDCl3 7.25 7.23 7.17 7.17 7.16 7.15 6.98 6.98 6.98 6.96 6.96 6.96 6.78 6.78 6.77 6.73 6.73 6.71 6.71 4.41 4.39 4.38 4.37 3.70 3.21 3.20 3.18 3.17 3.14 3.12 3.10 3.09
O
O
HN
CF3
3e
4.06
1.84
1.01
0.99
0.93
1.77
2.86
0.92
1.00
f1 (ppm)

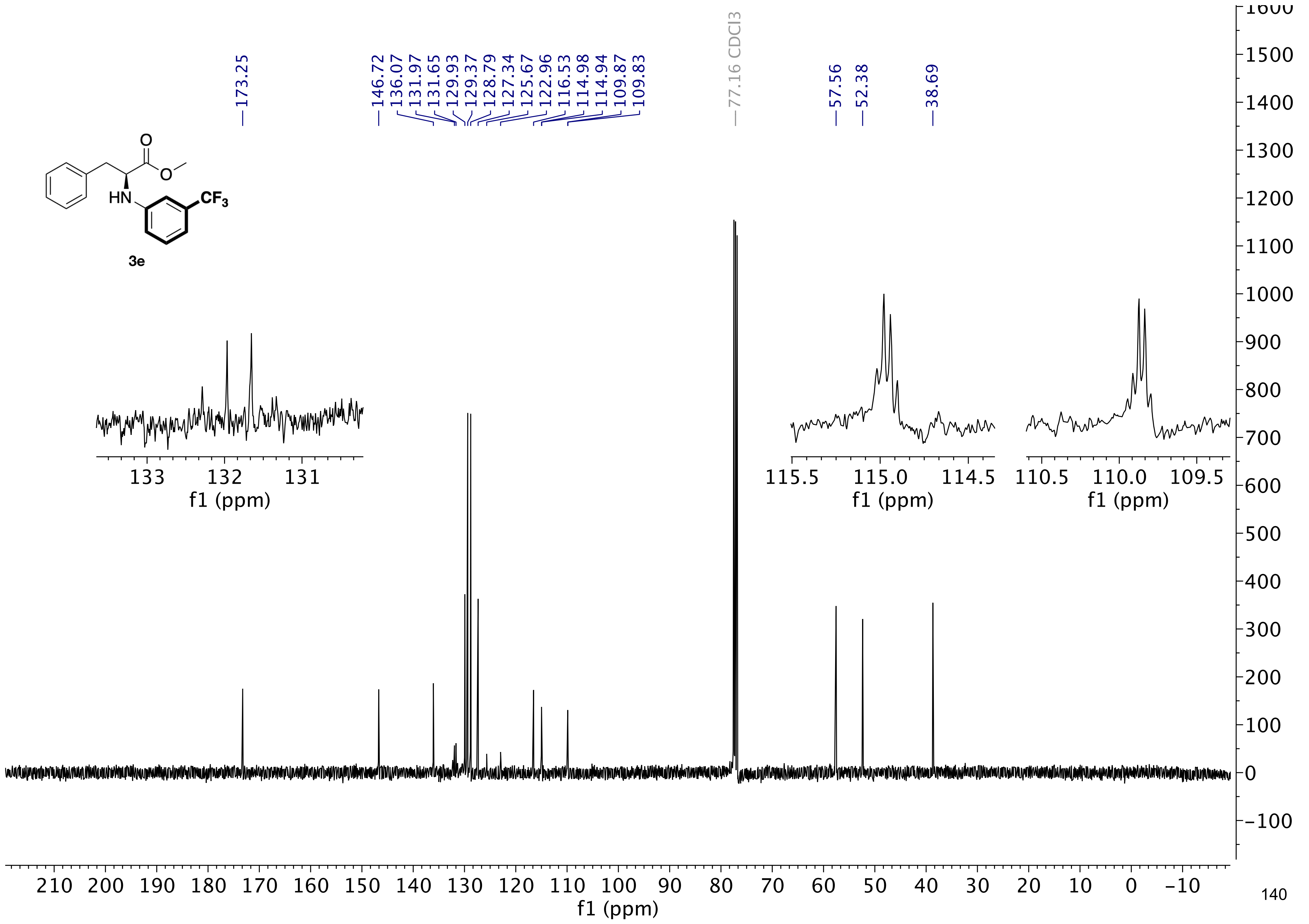
173.25
146.72
136.07
131.97
131.65
129.93
129.37
128.79
127.34
125.67
122.96
116.53
114.98
114.94
109.87
109.83
77.16 CDCl3
57.56
52.38
38.69
O
O
HN
CF3
3e
f1 (ppm)

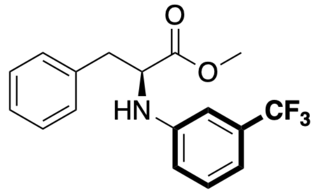

3e

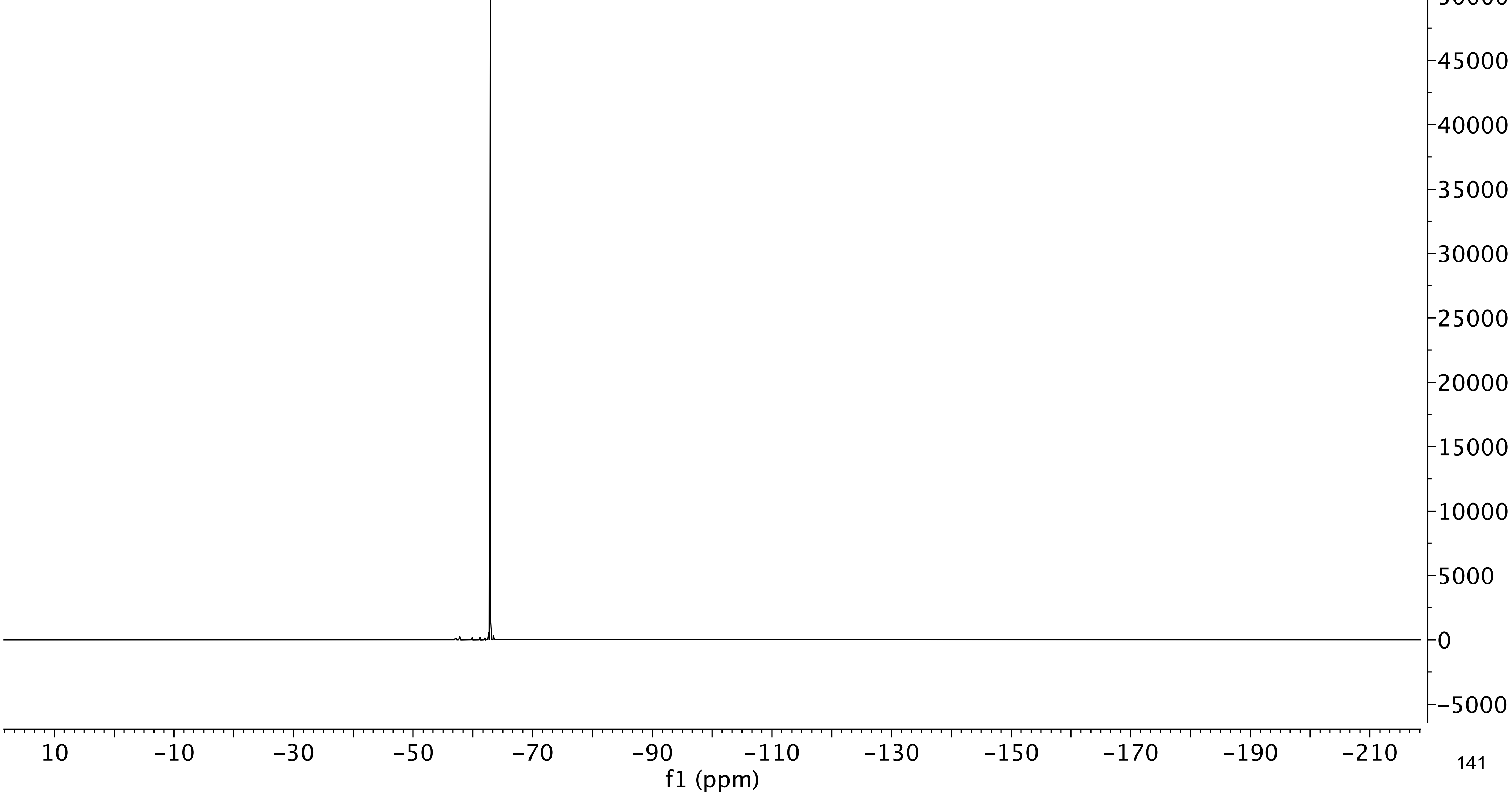

−62.90
70000
65000
60000
55000
50000
45000
40000
35000
30000
25000
20000
15000
10000
5000
0
−5000
10
−10
−30
−50
−70
−90
−110
−130
−150
−170
−190
−210
f1 (ppm)

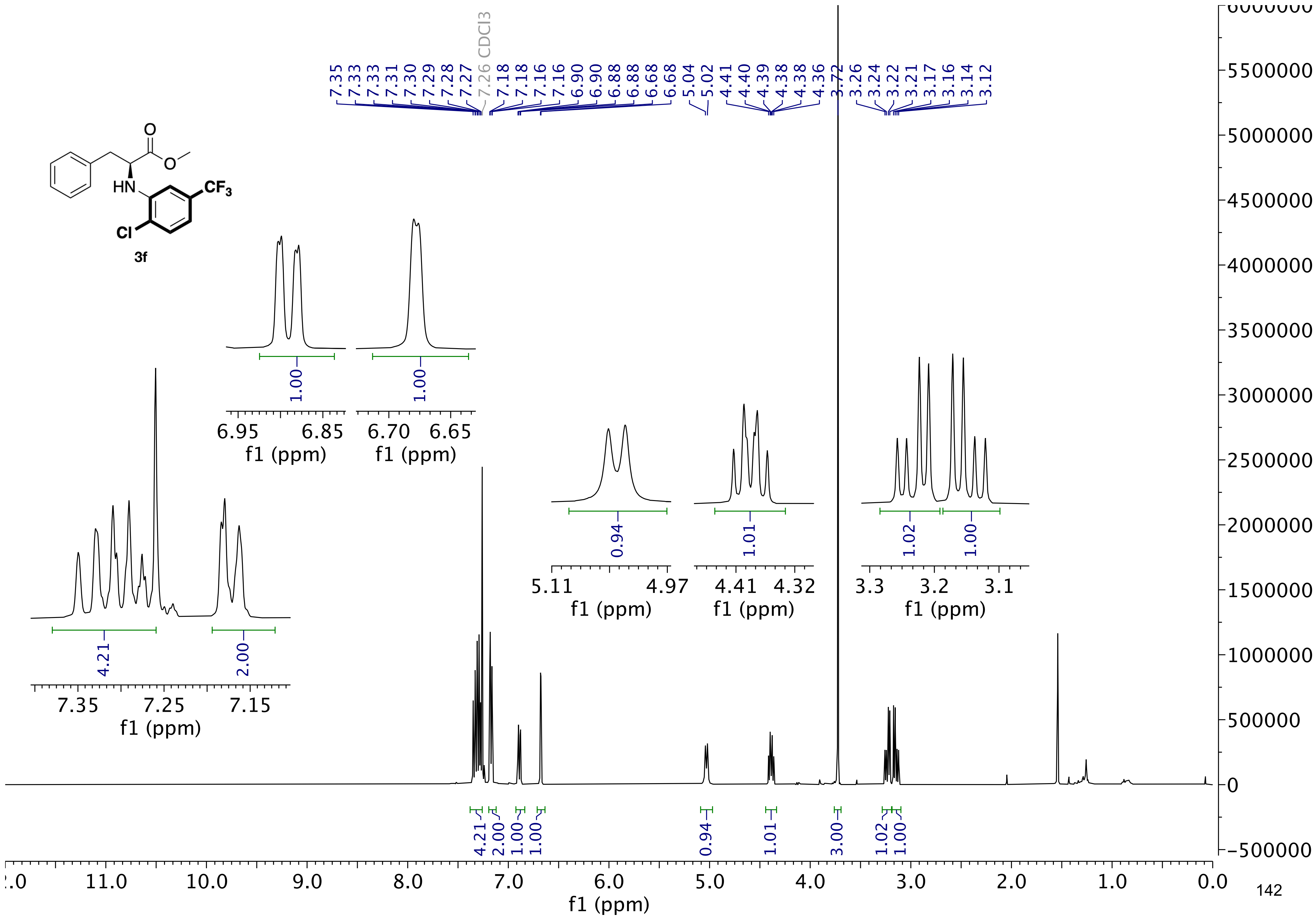

3f
CF3
HN
Cl
O
7.35 7.33 7.33 7.31 7.30 7.29 7.28 7.27 7.26 CDCl3 7.18 7.18 7.16 7.16 6.90 6.90 6.88 6.88 6.68 6.68 5.04 5.02 4.41 4.40 4.39 4.38 4.38 4.36 3.72 3.26 3.24 3.22 3.21 3.17 3.16 3.14 3.12
4.21 2.00 1.00 1.00 0.94 1.01 3.00 1.02 1.00
f1 (ppm)

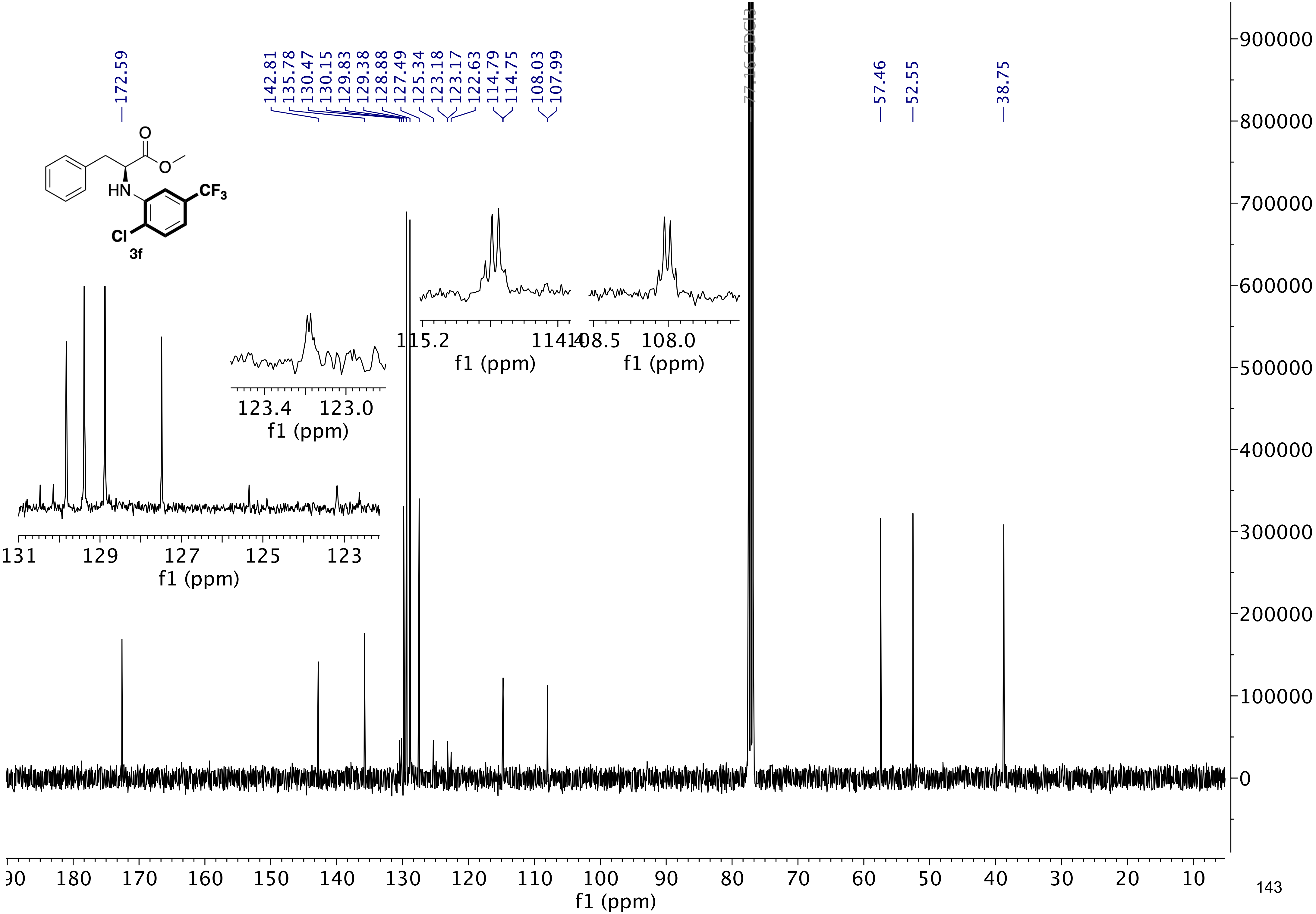

172.59
142.81
135.78
130.47
130.15
129.83
129.38
128.88
127.49
125.34
123.18
123.17
122.63
114.79
114.75
108.03
107.99
77.16 CDCl3
57.46
52.55
38.75
O
O
HN
CF3
Cl
3f
f1 (ppm)

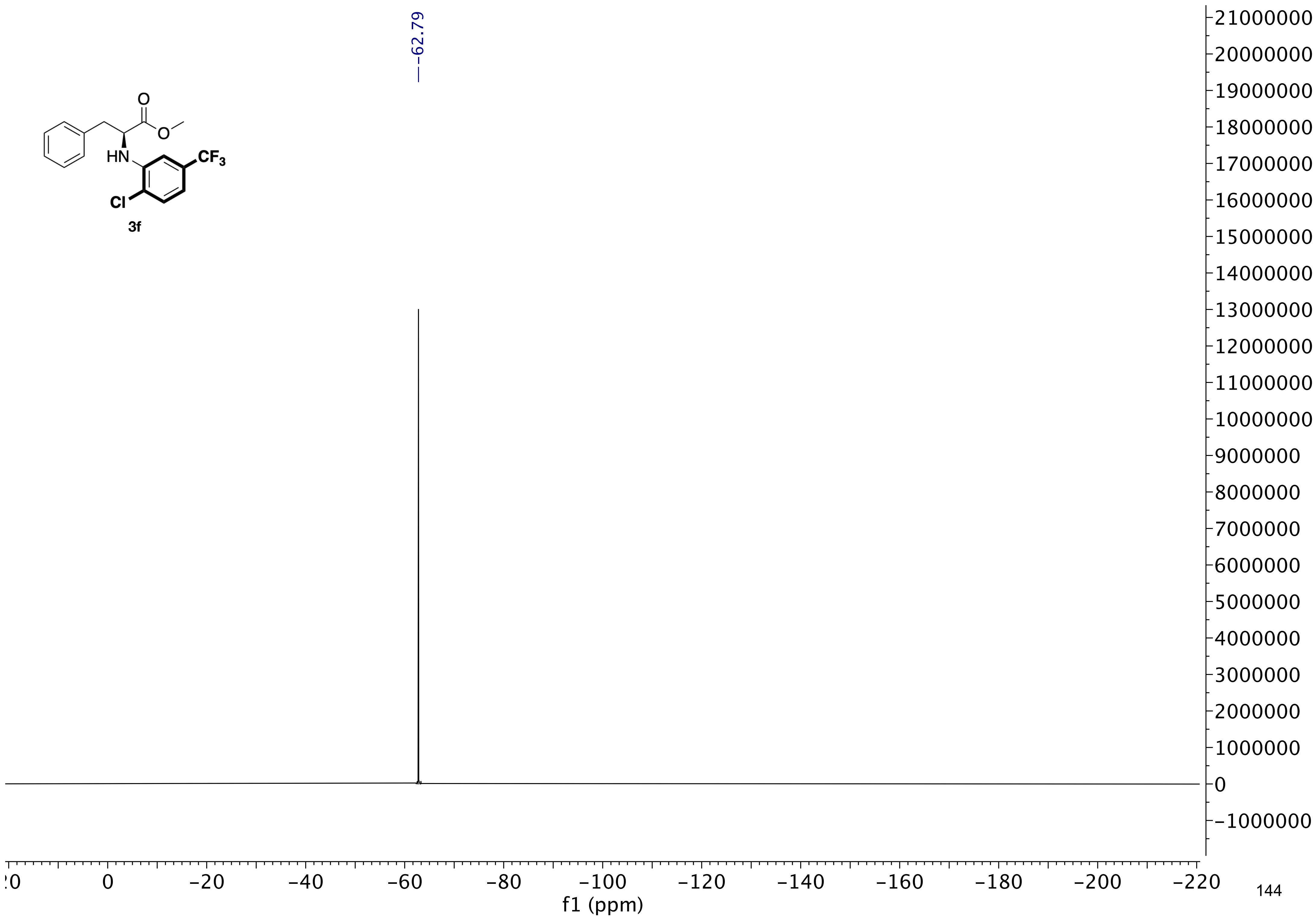

3f
CF3
HN
Cl
O
-62.79
f1 (ppm)

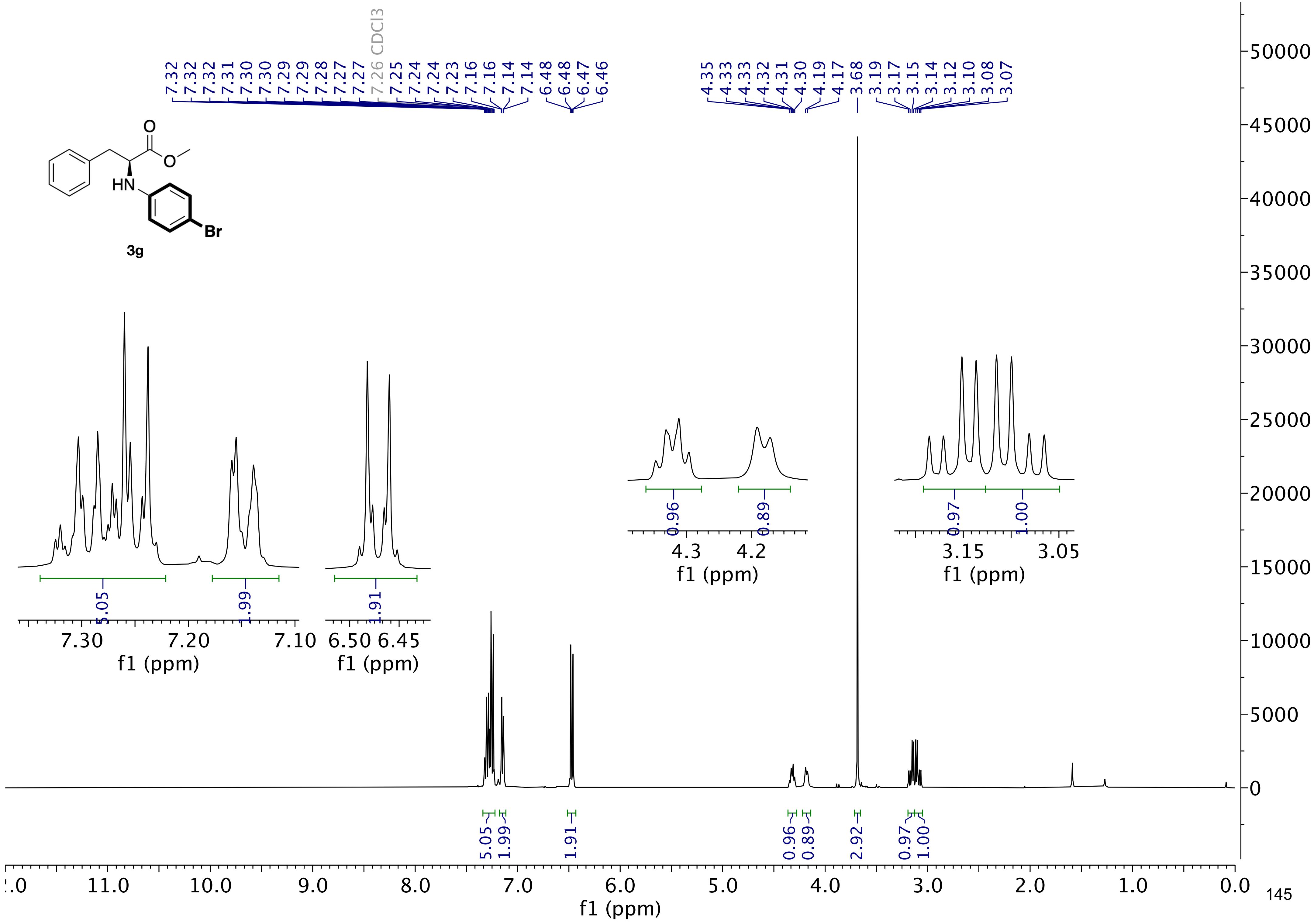

3g

145

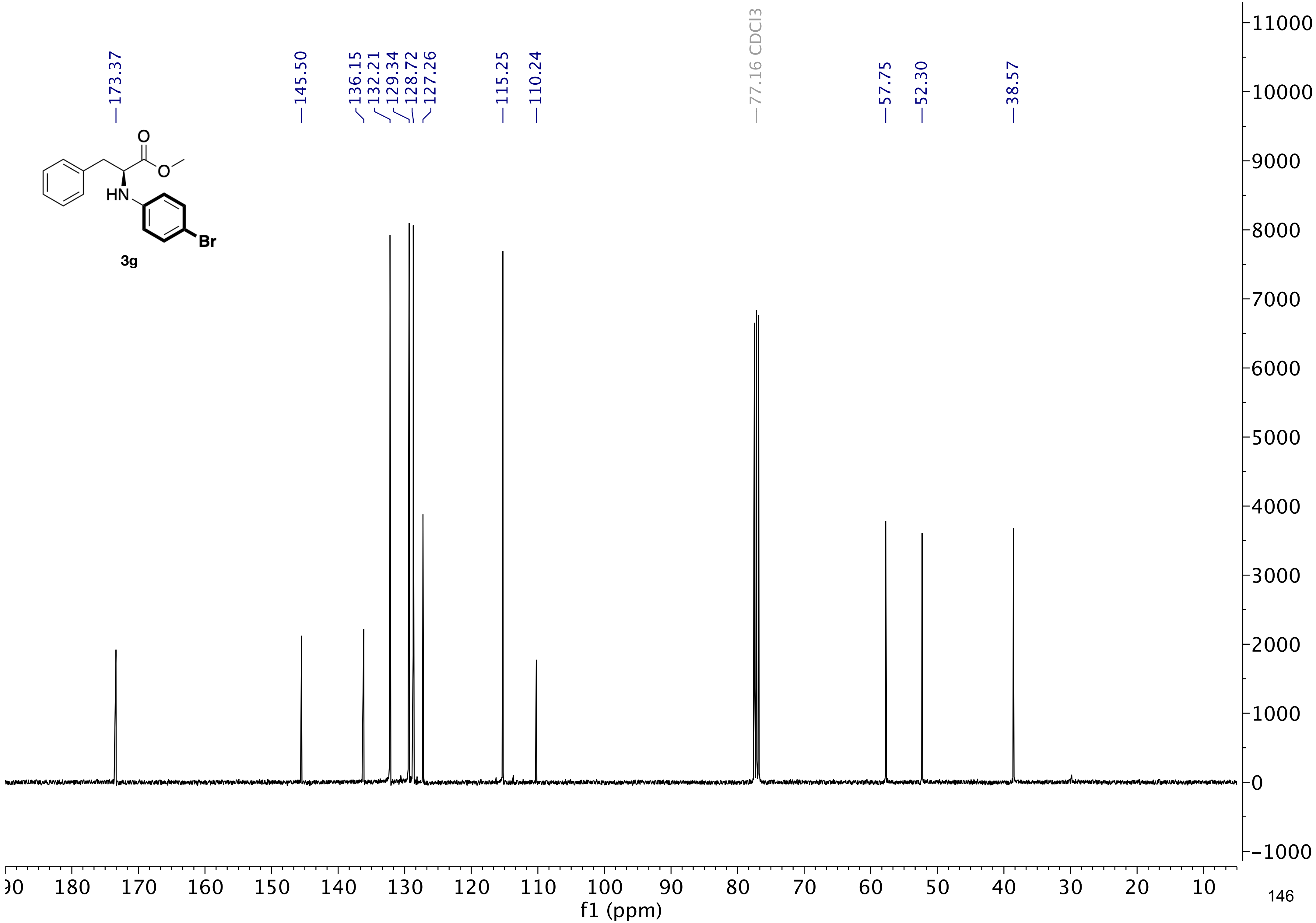
173.37
145.50
136.15
132.21
129.34
128.72
127.26
115.25
110.24
77.16 CDCl3
57.75
52.30
38.57
HN
O
Br
3g
f1 (ppm)

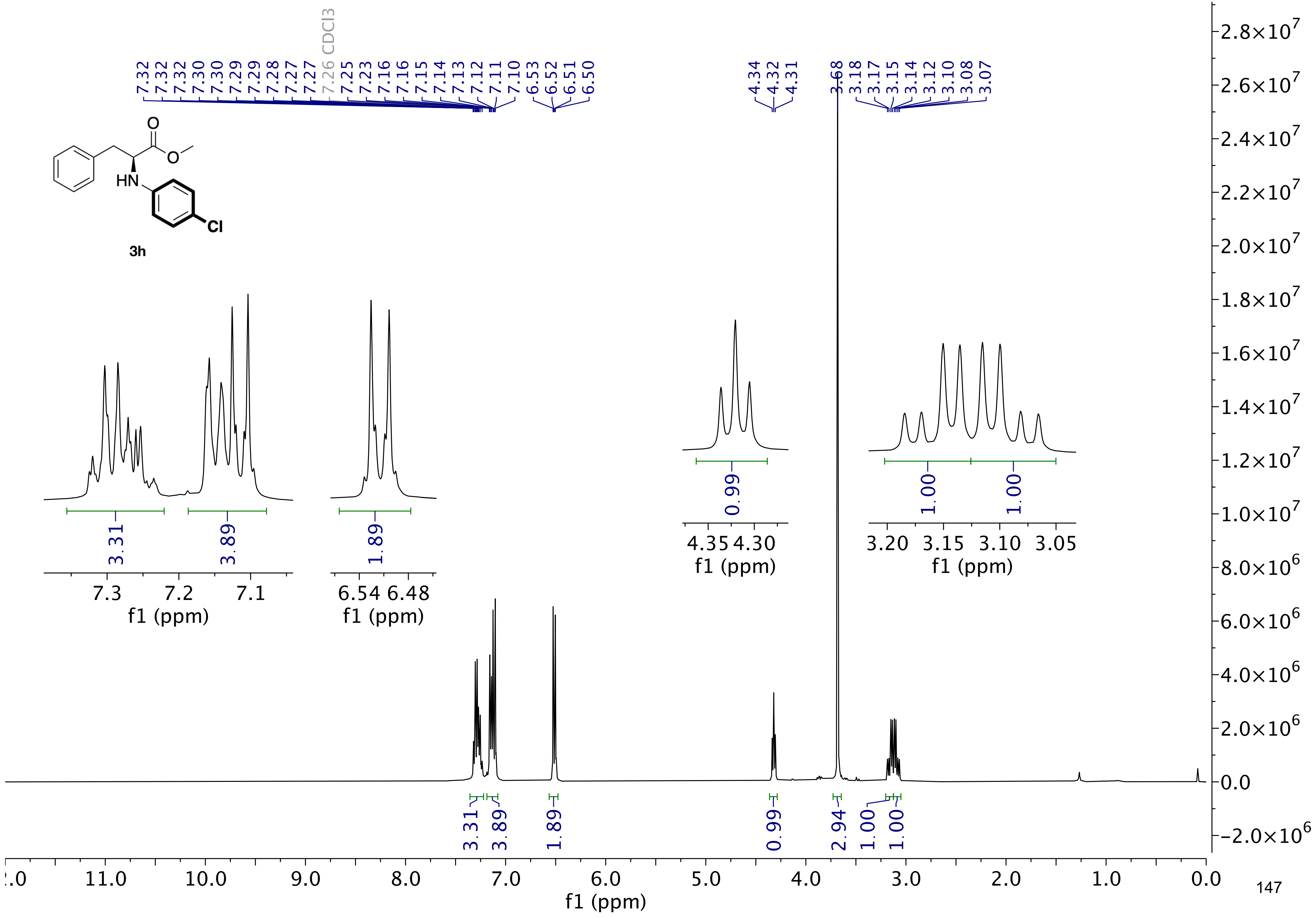
7.32
7.32
7.32
7.30
7.30
7.29
7.29
7.28
7.27
7.27
7.26 CDCl3
7.25
7.23
7.16
7.16
7.15
7.14
7.13
7.12
7.11
7.10
6.53
6.52
6.51
6.50
4.34
4.32
4.31
3.68
3.18
3.17
3.15
3.14
3.12
3.10
3.08
3.07
O
O
HN
Cl
3h
3.31
3.89
1.89
0.99
2.94
1.00
1.00
f1 (ppm)

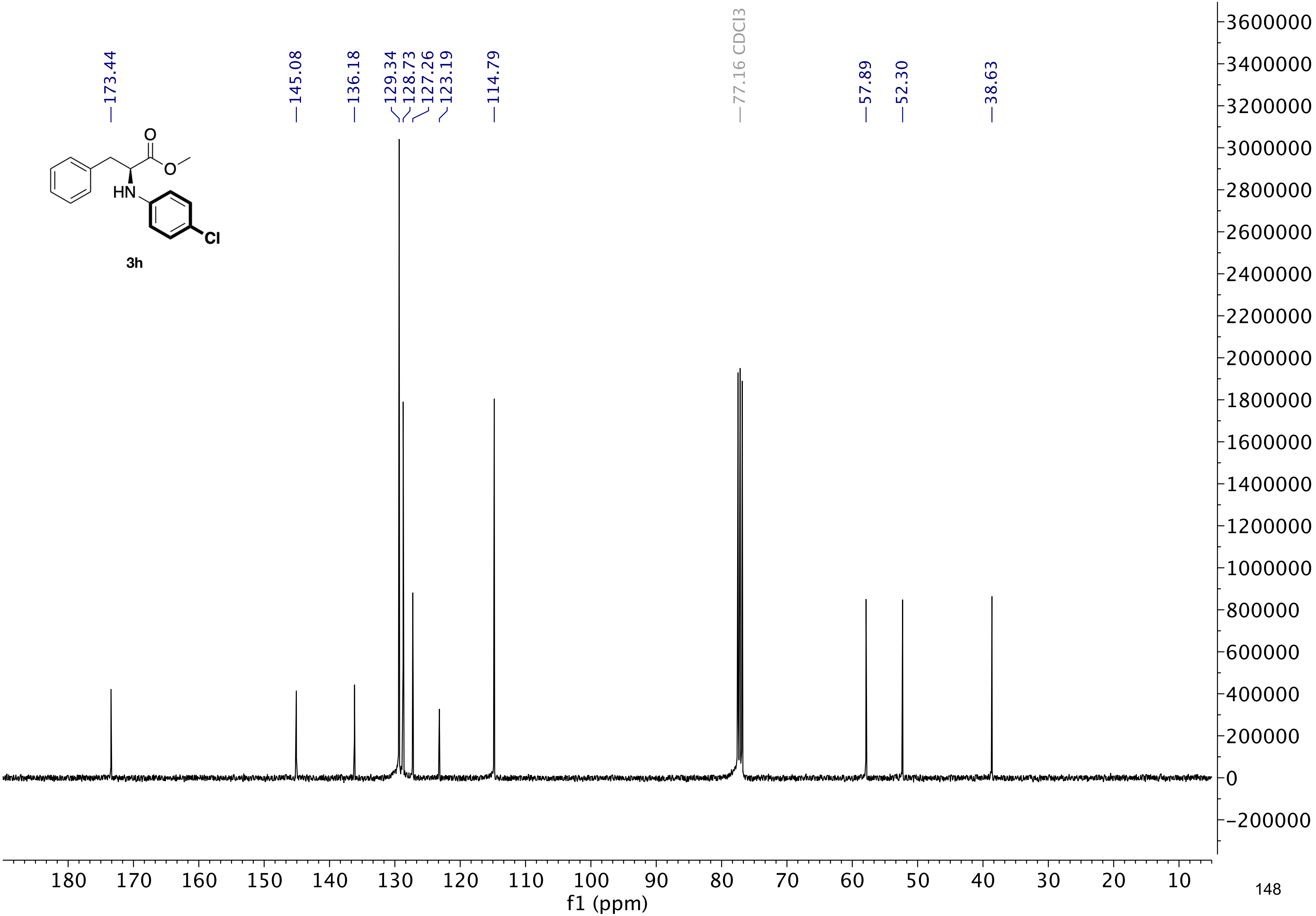
173.44
145.08
136.18
129.34
128.73
127.26
123.19
114.79
77.16 CDCl3
57.89
52.30
38.63
O
O
HN
Cl
3h
3600000
3400000
3200000
3000000
2800000
2600000
2400000
2200000
2000000
1800000
1600000
1400000
1200000
1000000
800000
600000
400000
200000
0
-200000
180 170 160 150 140 130 120 110 100 90 80 70 60 50 40 30 20 10
f1 (ppm)

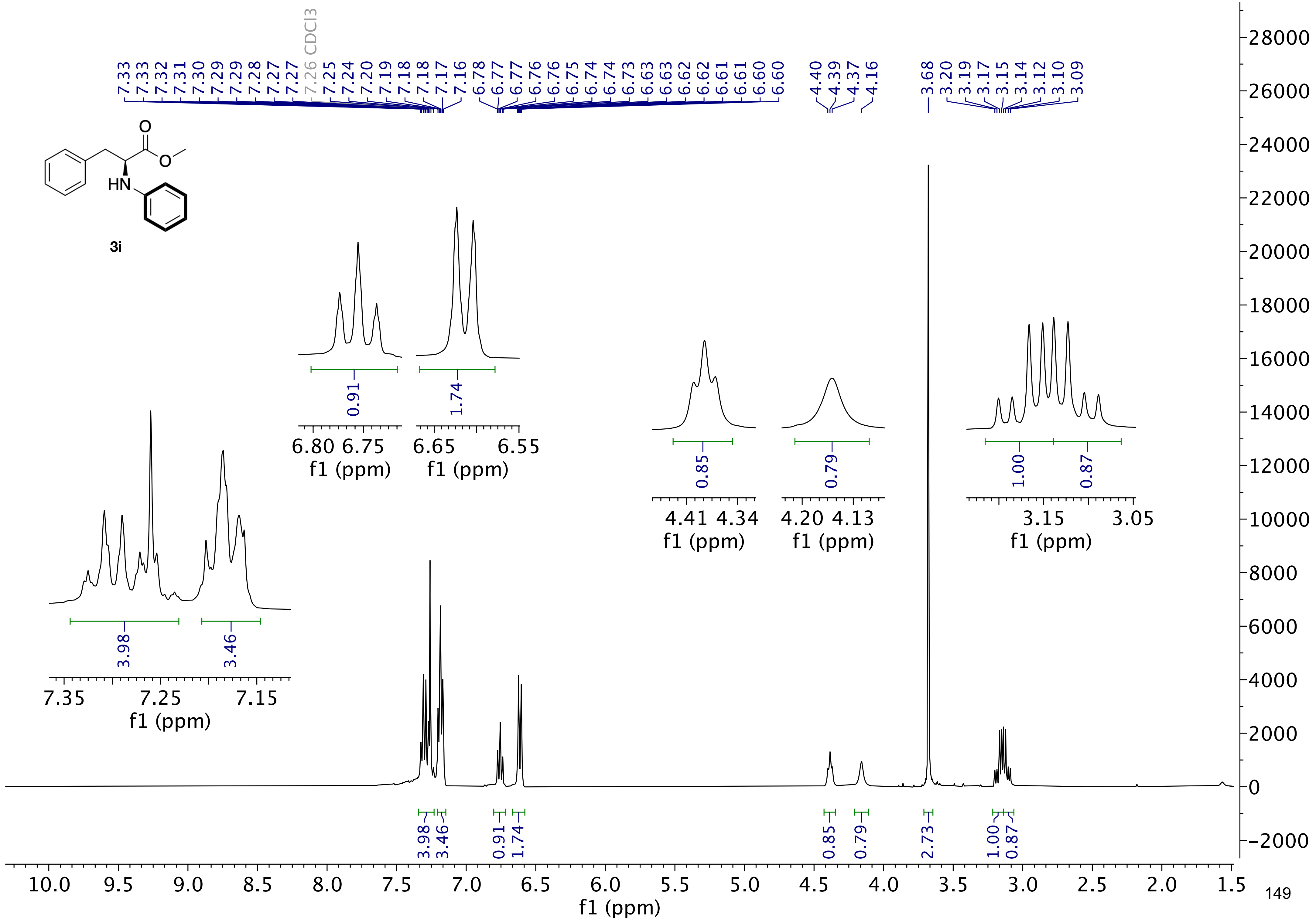
7.33 7.33 7.32 7.31 7.30 7.29 7.29 7.28 7.27 7.27 7.26 CDCl3 7.25 7.24 7.20 7.19 7.18 7.18 7.17 7.16 6.78 6.77 6.77 6.76 6.76 6.75 6.74 6.74 6.73 6.63 6.63 6.62 6.62 6.61 6.61 6.60 6.60 4.40 4.39 4.37 4.16 3.68 3.20 3.19 3.17 3.15 3.14 3.12 3.10 3.09
O
O
HN
3i
3.98
3.46
0.91
1.74
0.85
0.79
2.73
1.00
0.87
f1 (ppm)

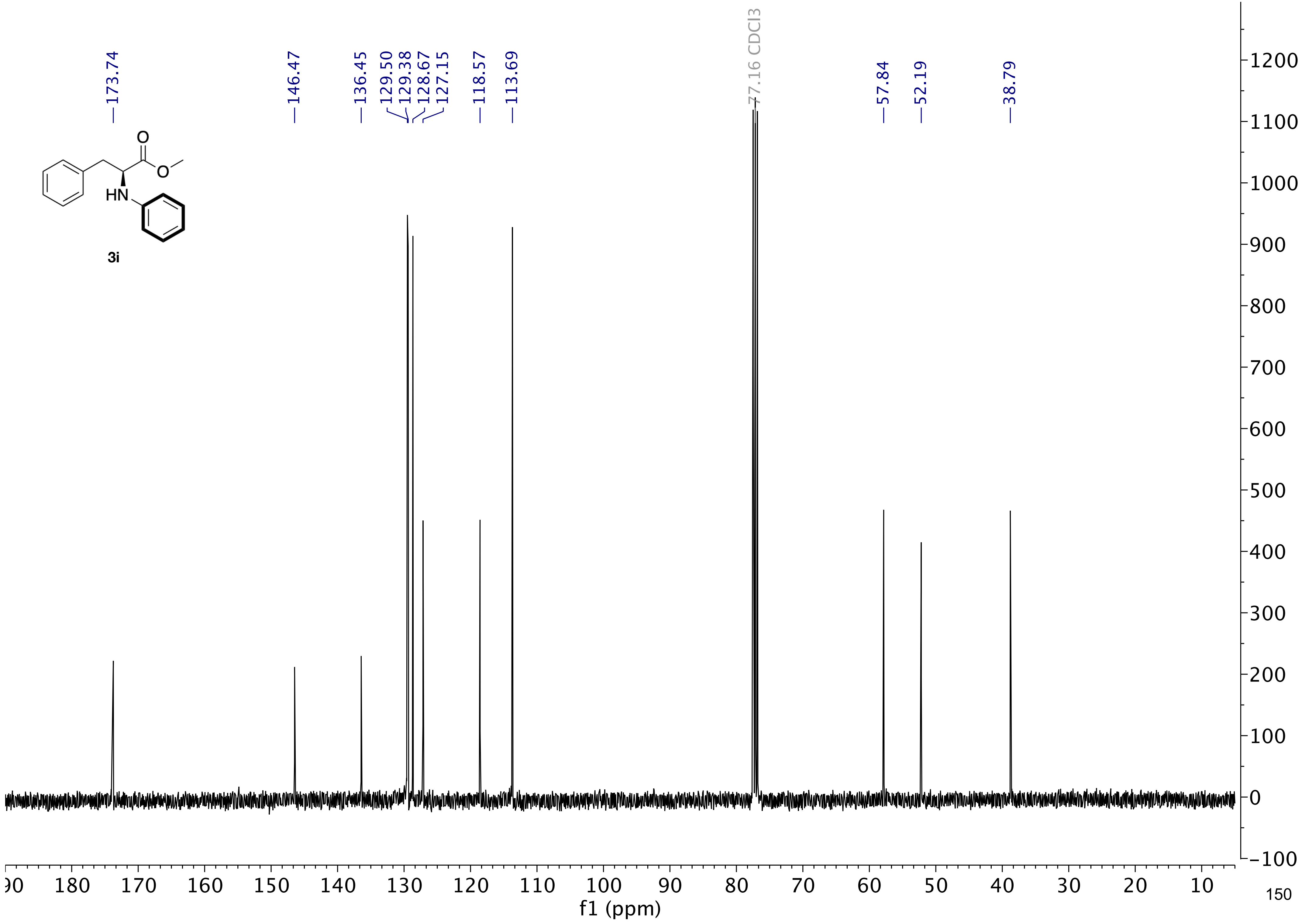
173.74
146.47
136.45
129.50
129.38
128.67
127.15
118.57
113.69
77.16 CDCl3
57.84
52.19
38.79
O
O
HN
3i
f1 (ppm)

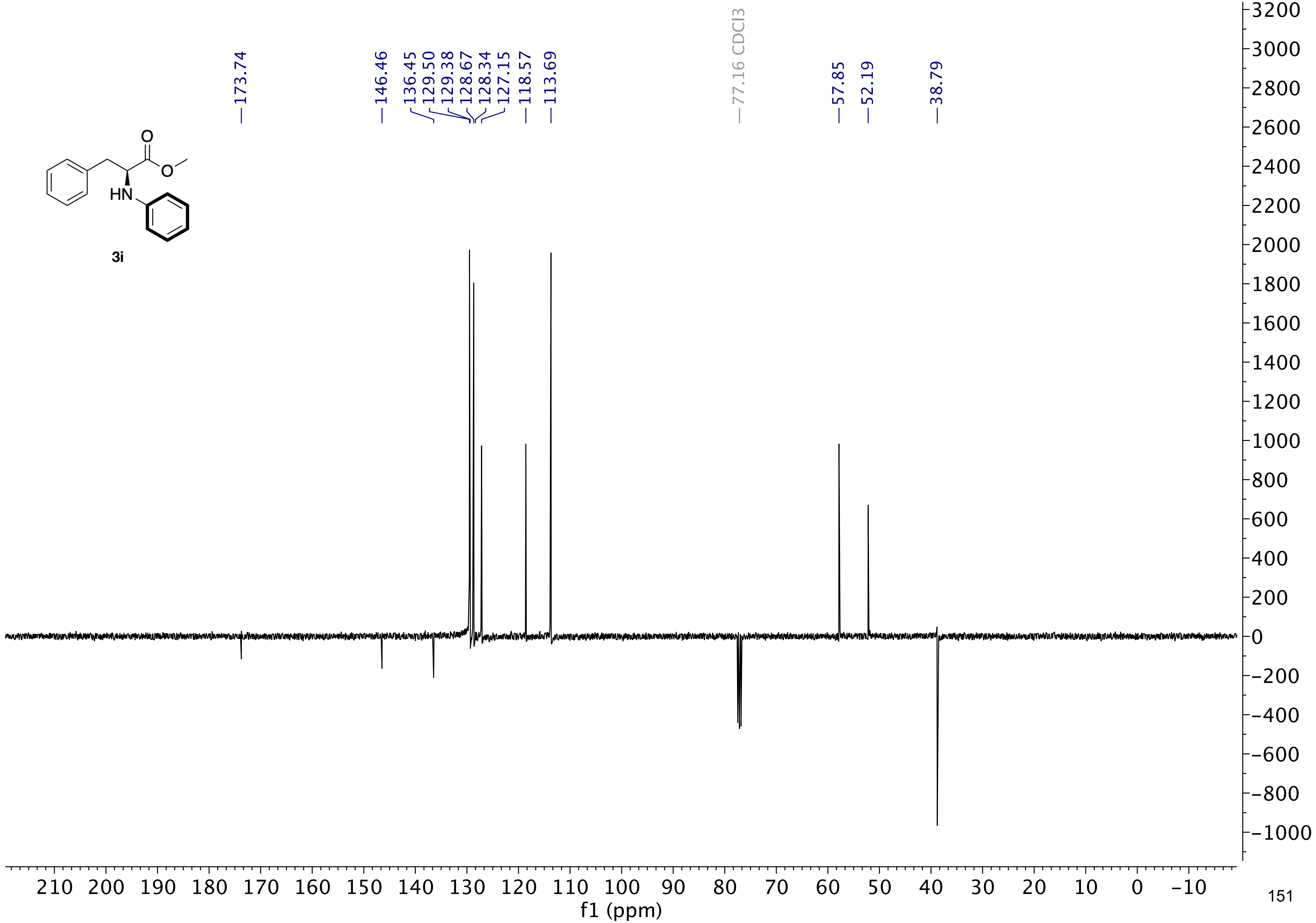
173.74
146.46
136.45
129.50
129.38
128.67
128.34
127.15
118.57
113.69
77.16 CDCl3
57.85
52.19
38.79
O
O
HN
3i
f1 (ppm)

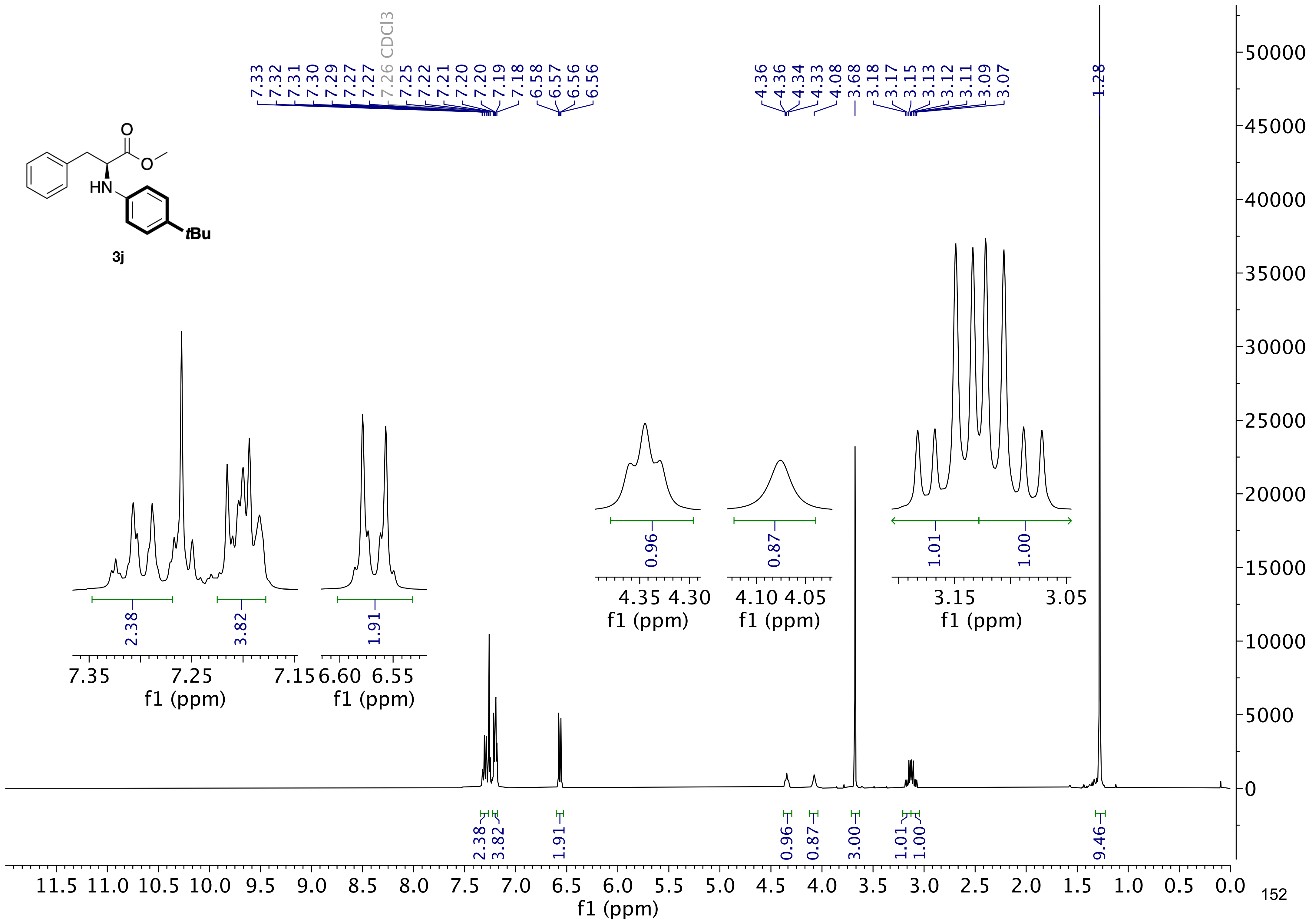

HN
O
O
tBu
3j
7.33 7.32 7.31 7.30 7.29 7.27 7.27 7.26 CDCl3 7.25 7.22 7.21 7.20 7.20 7.19 7.18 6.58 6.57 6.56 6.56
4.36 4.36 4.34 4.33 4.08 3.68 3.18 3.17 3.15 3.13 3.12 3.11 3.09 3.07
1.28
2.38 3.82 1.91 0.96 0.87 3.00 1.01 1.00 9.46
f1 (ppm)

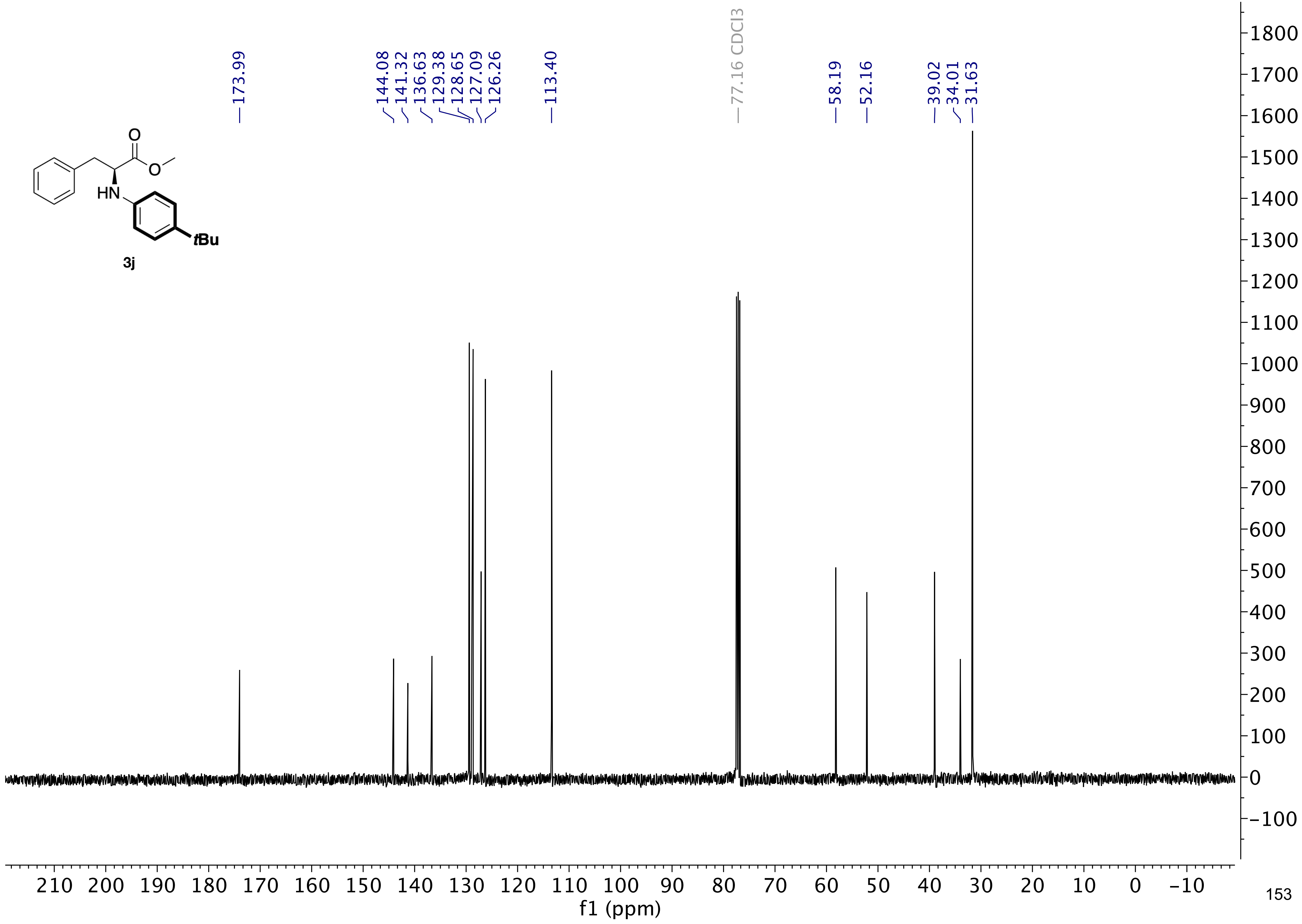
173.99
144.08
141.32
136.63
129.38
128.65
127.09
126.26
113.40
77.16 CDCl3
58.19
52.16
39.02
34.01
31.63
O
O
HN
tBu
3j
f1 (ppm)

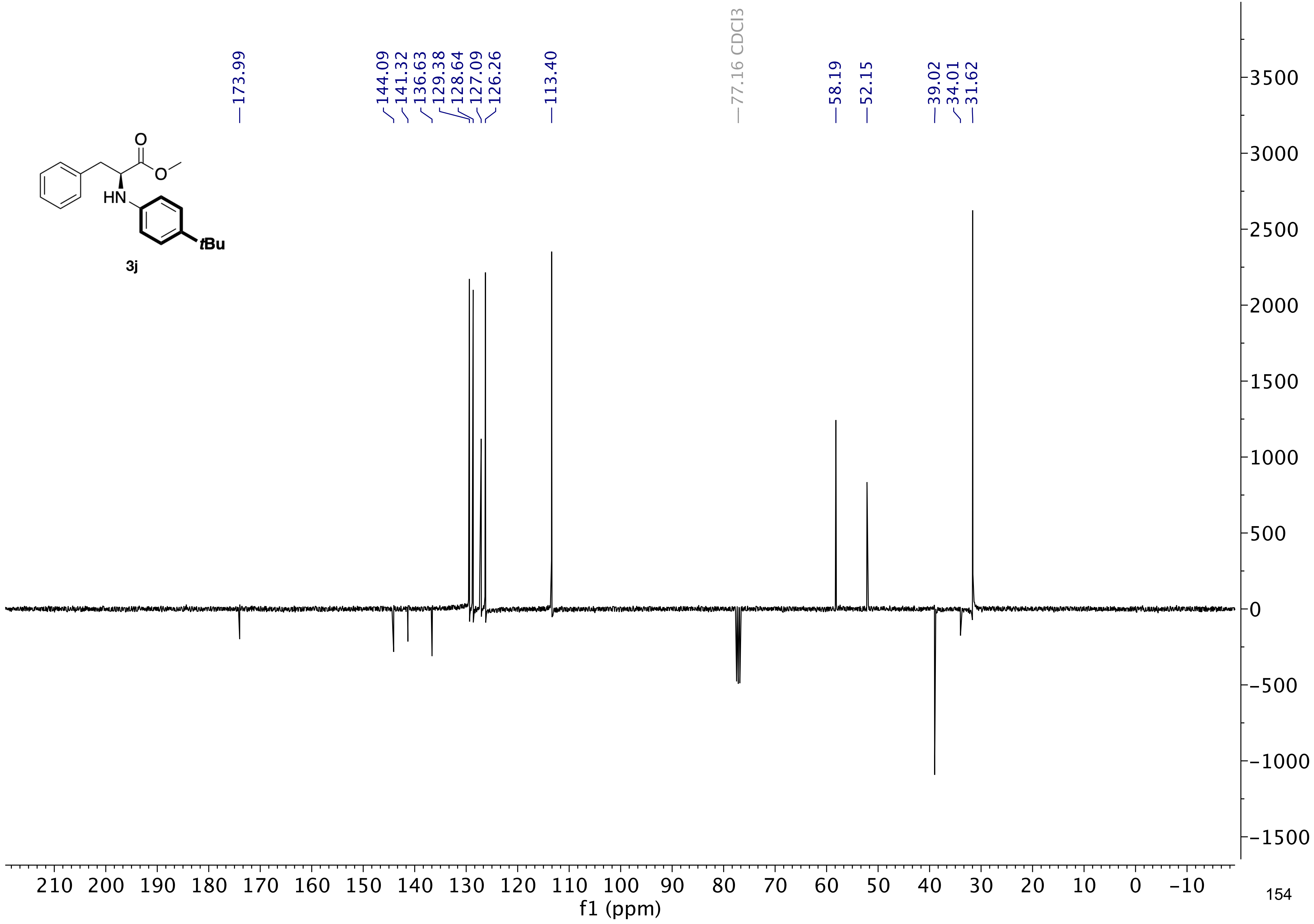
173.99
144.09
141.32
136.63
129.38
128.64
127.09
126.26
113.40
77.16 CDCl3
58.19
52.15
39.02
34.01
31.62
O
O
HN
tBu
3j
3500
3000
2500
2000
1500
1000
500
0
−500
−1000
−1500
210 200 190 180 170 160 150 140 130 120 110 100 90 80 70 60 50 40 30 20 10 0 −10
f1 (ppm)

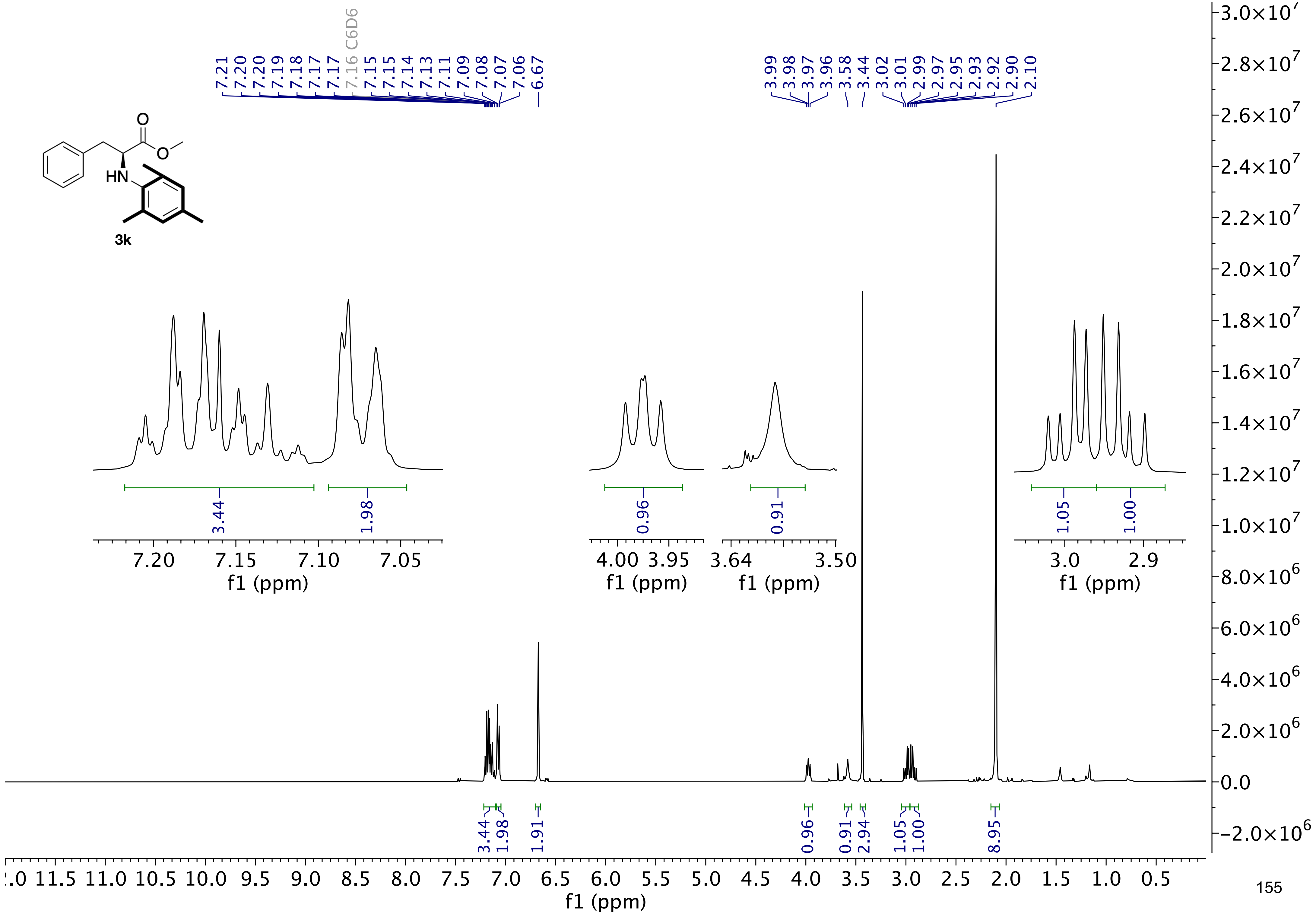

3k

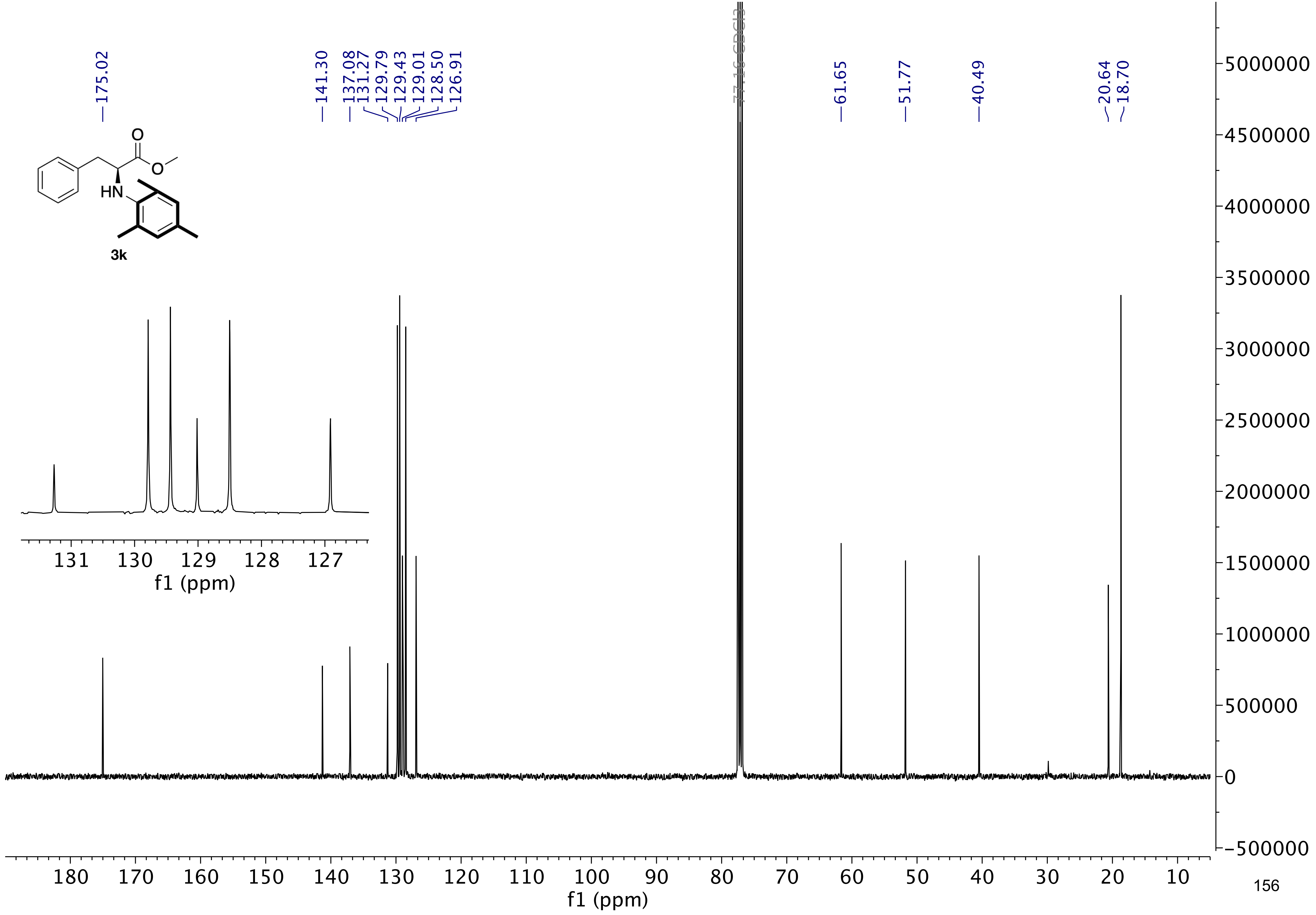
175.02
141.30
137.08
131.27
129.79
129.43
129.01
128.50
126.91
77.16 CDCl3
61.65
51.77
40.49
20.64
18.70
HN
O
3k
f1 (ppm)

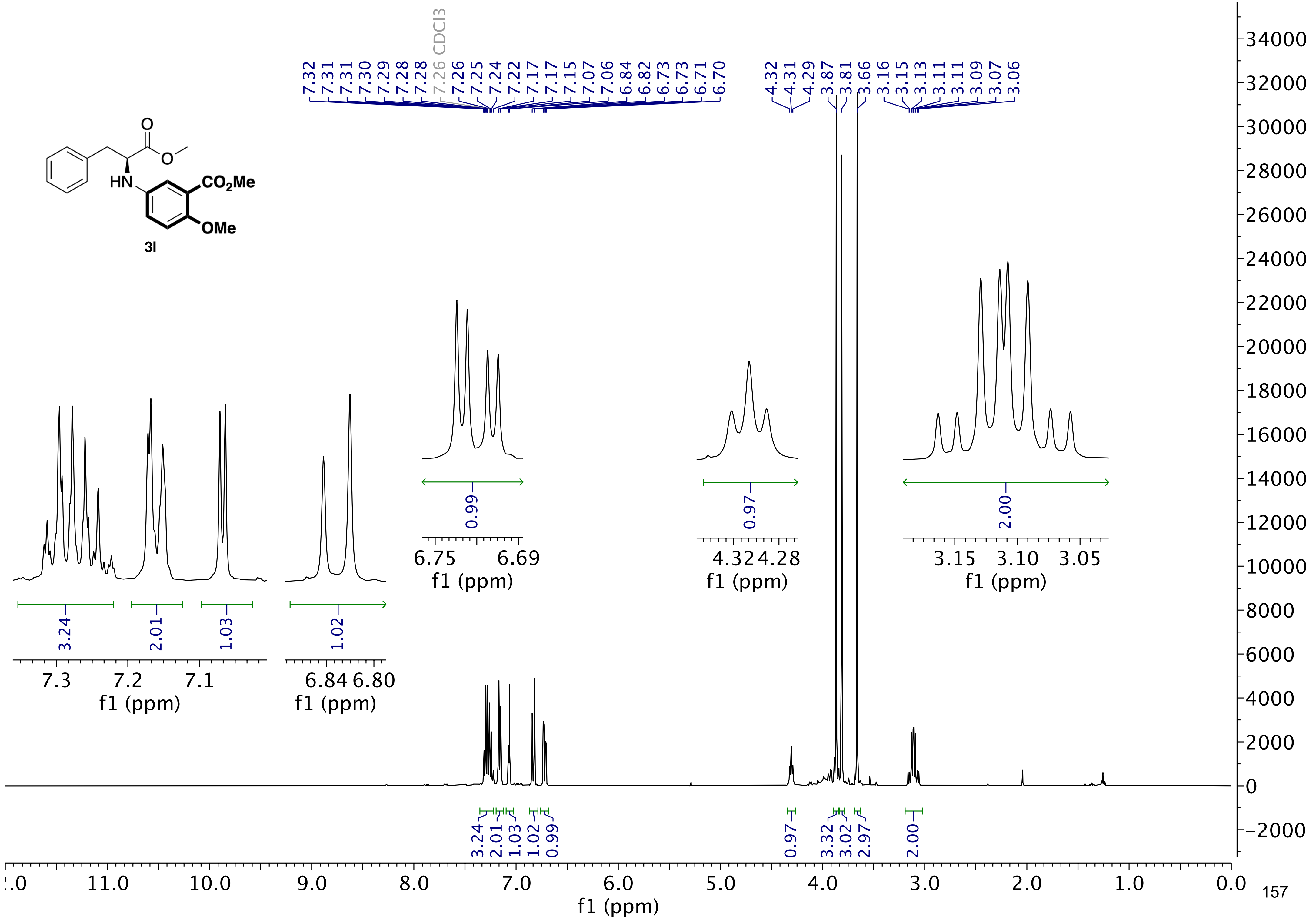
CO2Me
HN
O
O
OMe
3l
CDCl3
f1 (ppm)

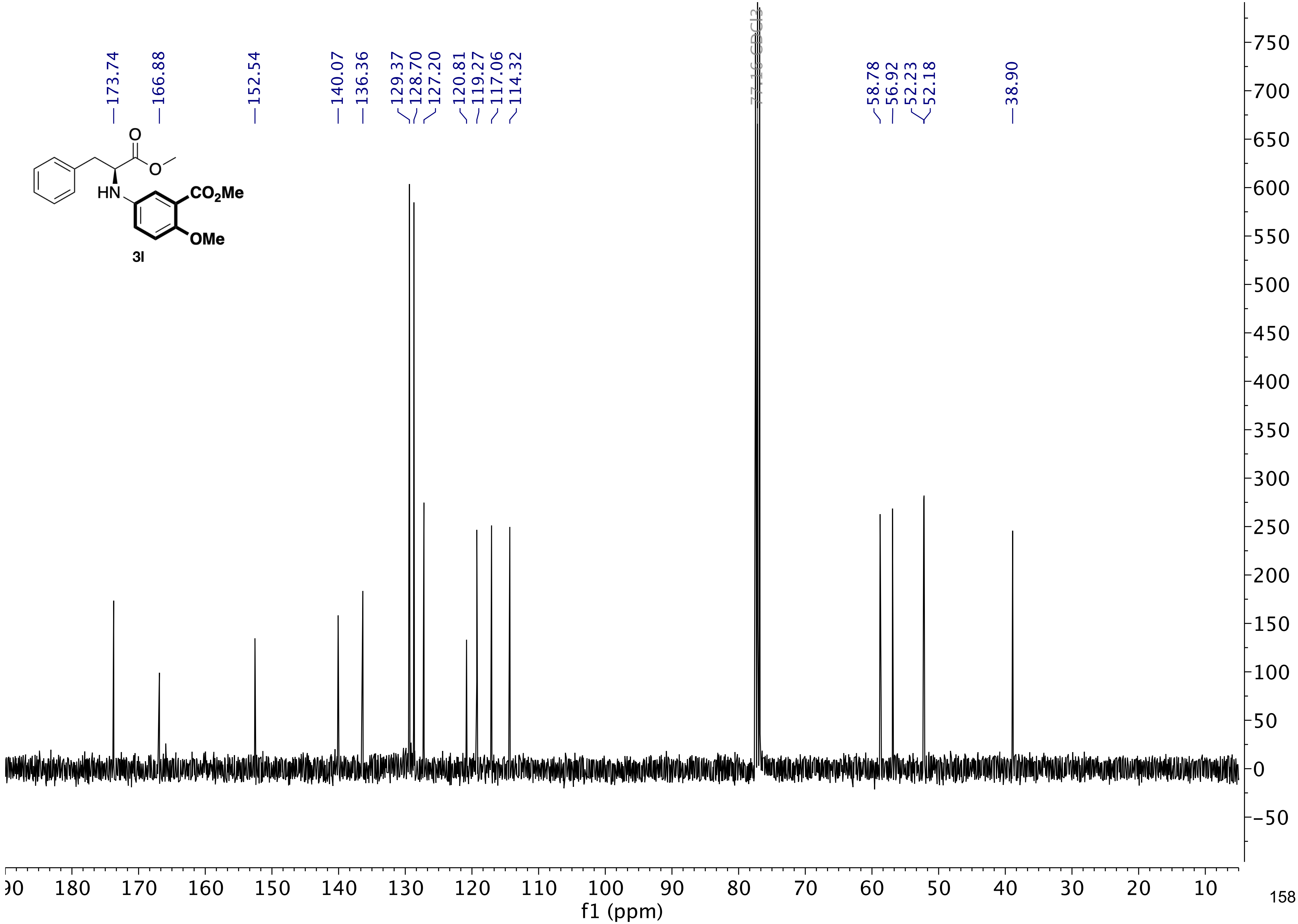
173.74
166.88
152.54
140.07
136.36
129.37
128.70
127.20
120.81
119.27
117.06
114.32
77.16 CDCl3
58.78
56.92
52.23
52.18
38.90
O
O
HN
CO_2Me
OMe
3l
f1 (ppm)

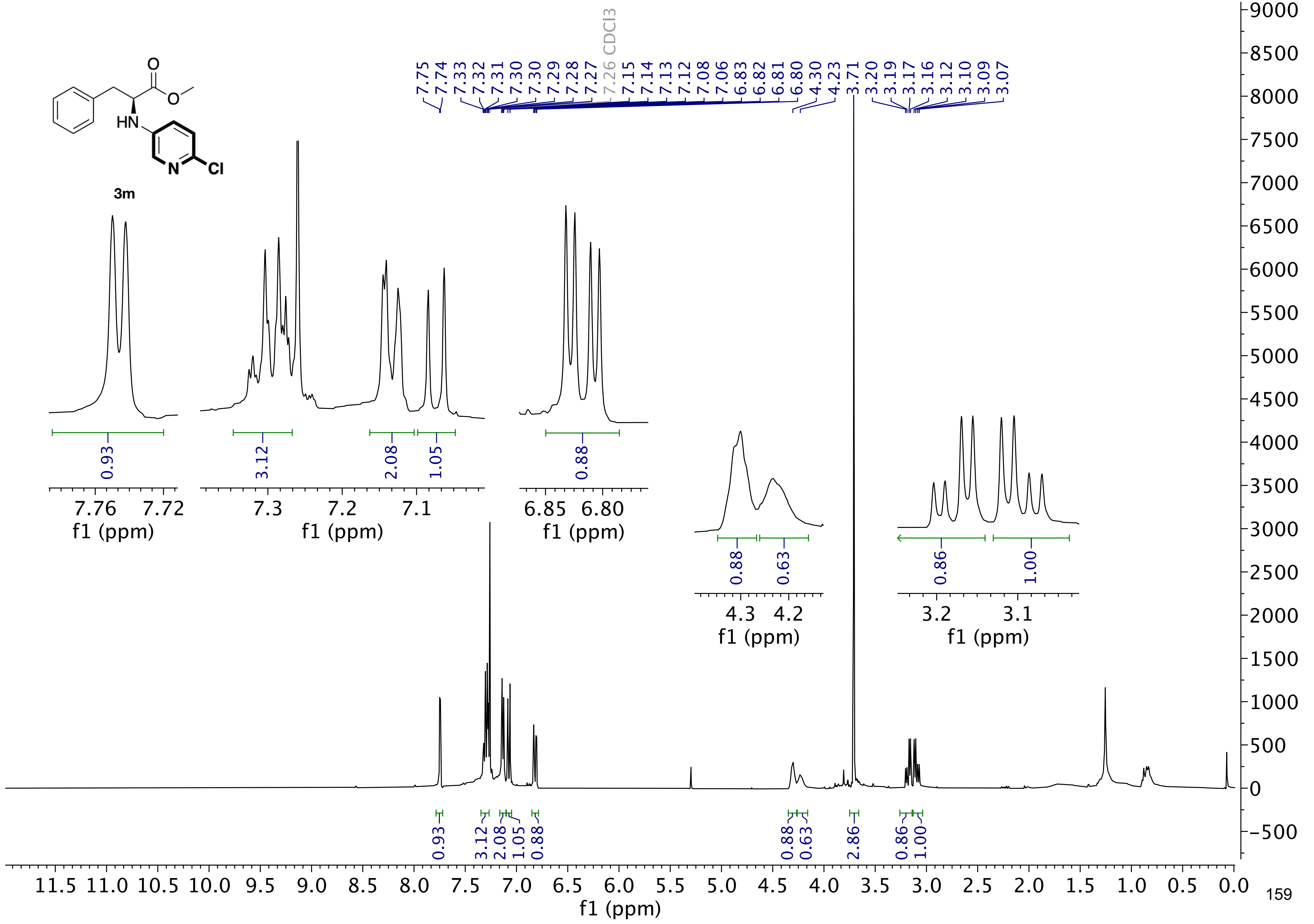

3m
HN
N
Cl
O
CDCl3
f1 (ppm)

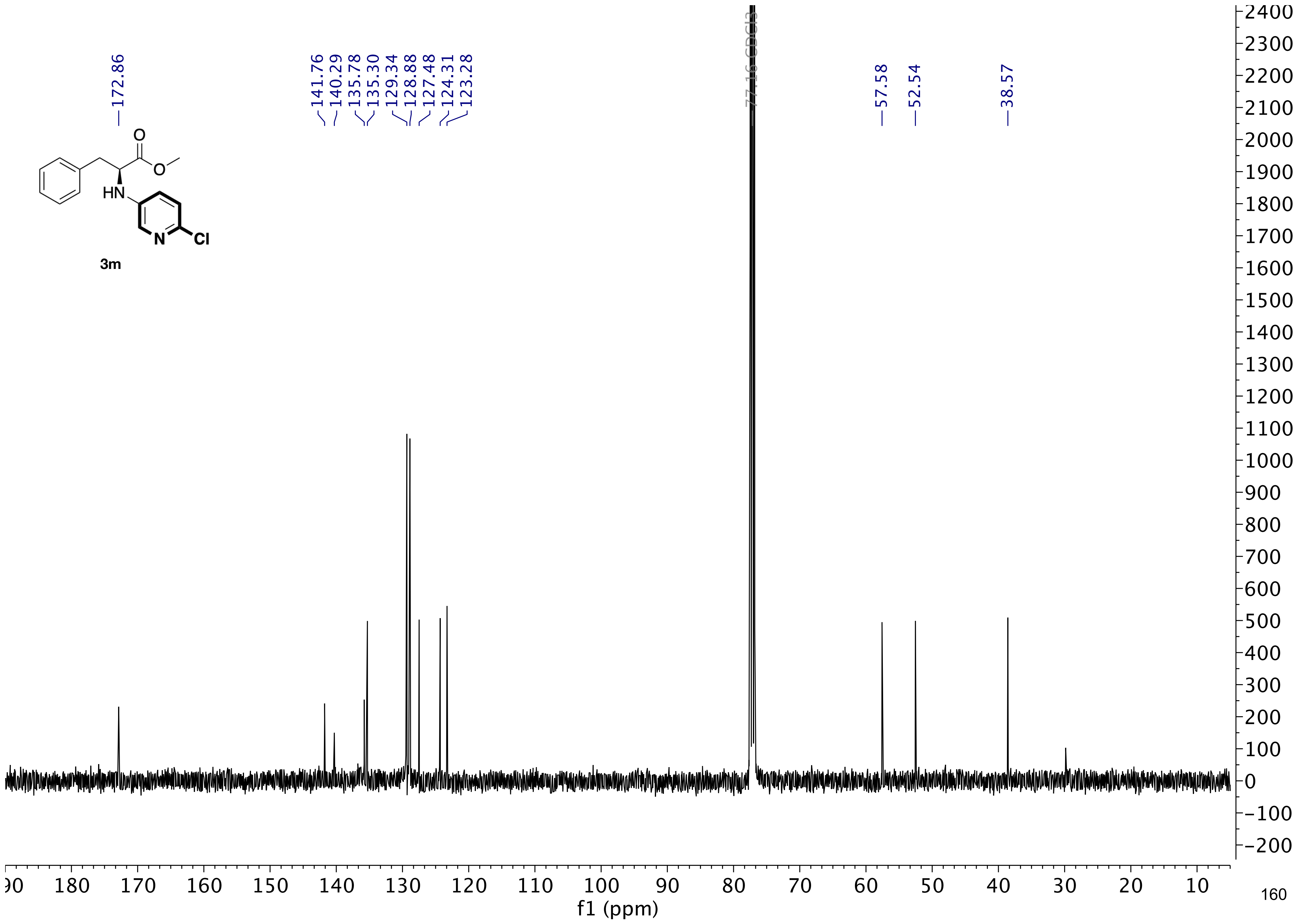
172.86
141.76
140.29
135.78
135.30
129.34
128.88
127.48
124.31
123.28
77.16 CDCl3
57.58
52.54
38.57
O
HN
N
Cl
3m
f1 (ppm)

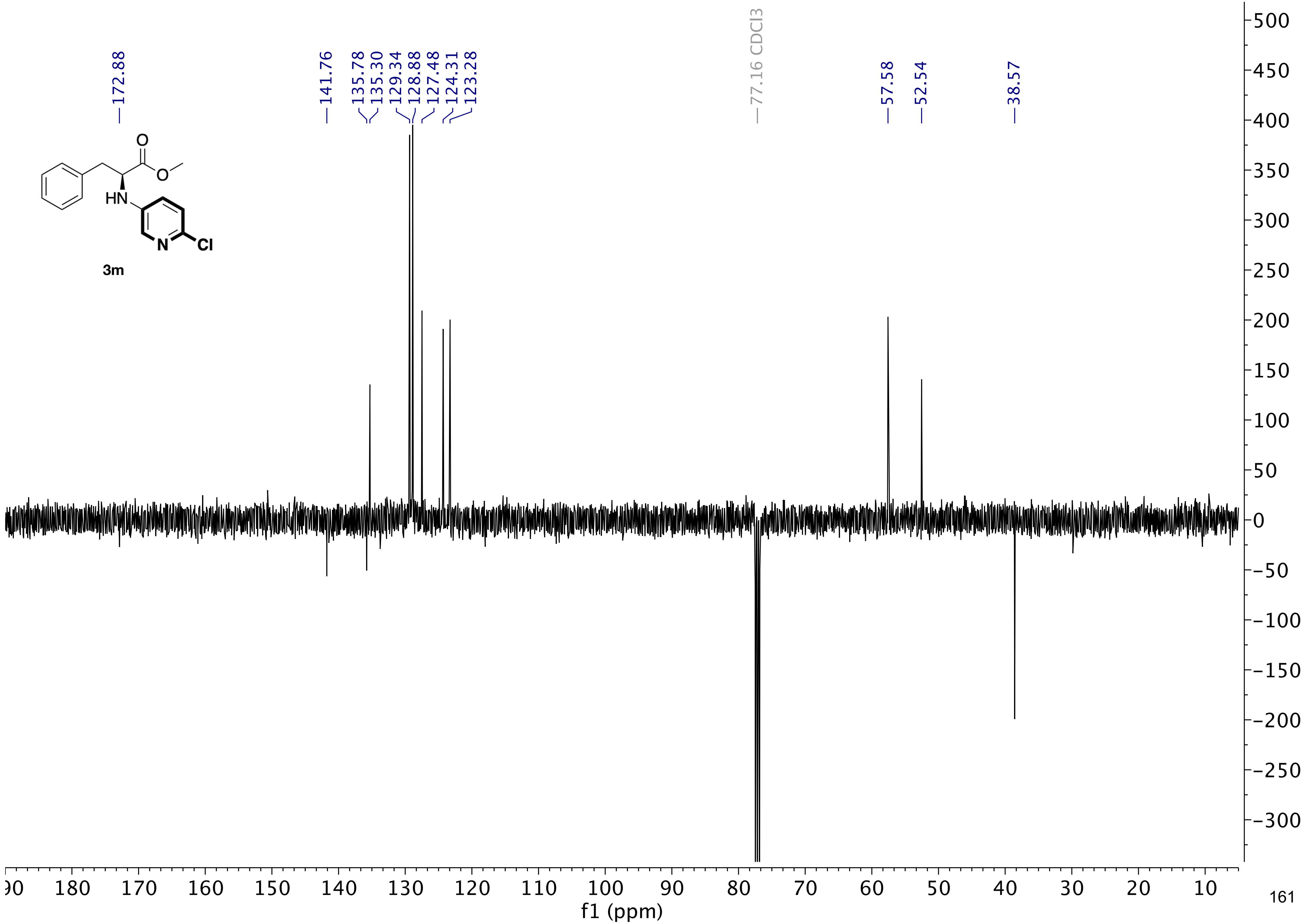
172.88
141.76
135.78
135.30
129.34
128.88
127.48
124.31
123.28
77.16 CDCl3
57.58
52.54
38.57
3m
f1 (ppm)

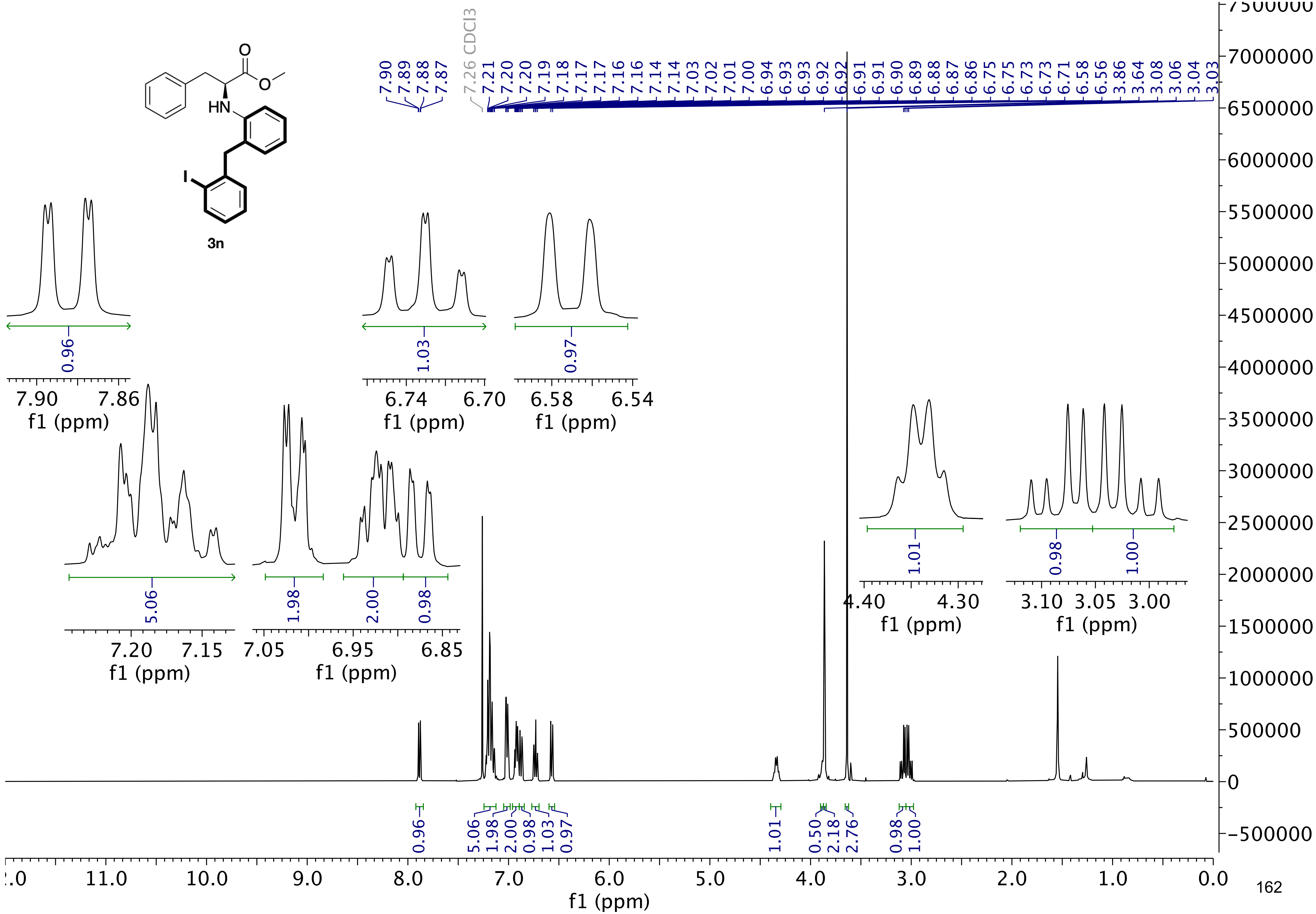
3n
f1 (ppm)
7.26 CDCl3

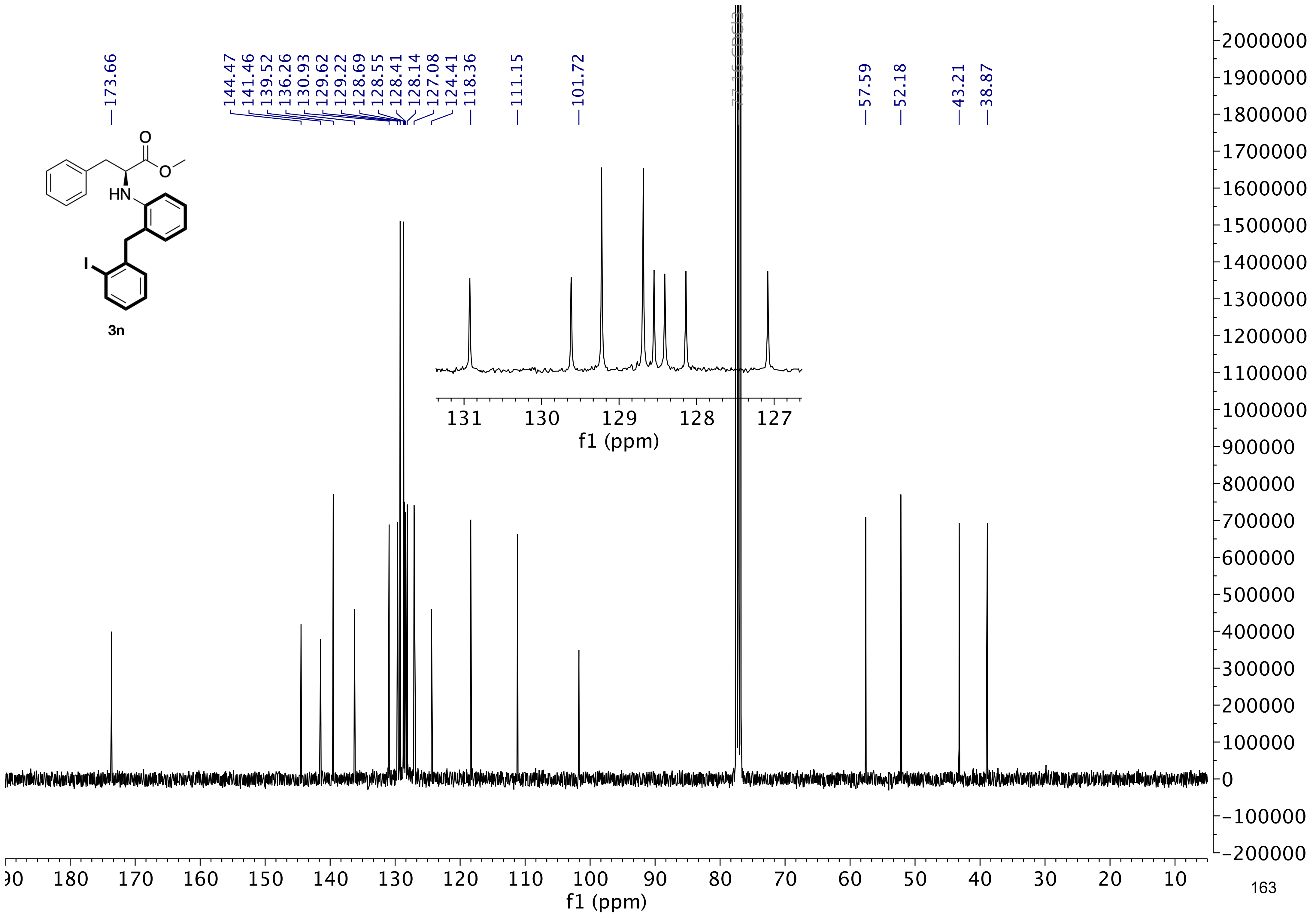
173.66
144.47
141.46
139.52
136.26
130.93
129.62
129.22
128.69
128.55
128.41
128.14
127.08
124.41
118.36
111.15
101.72
77.16 CDCl3
57.59
52.18
43.21
38.87
3n
f1 (ppm)

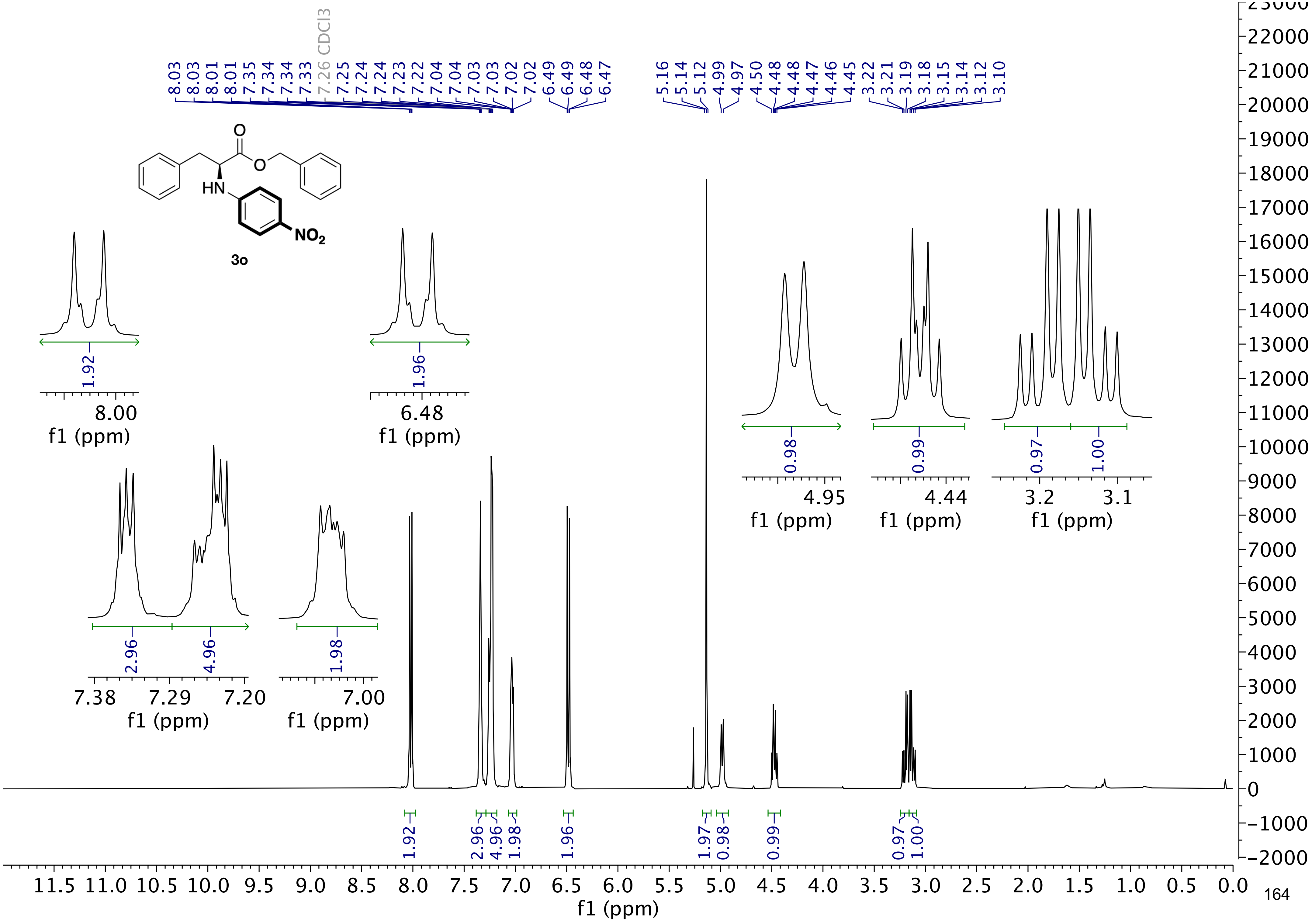
8.03 8.03 8.01 8.01 7.35 7.34 7.34 7.33 7.26 CDCl3 7.25 7.24 7.24 7.23 7.22 7.04 7.04 7.03 7.03 7.02 7.02 6.49 6.49 6.48 6.47
5.16 5.14 5.12 4.99 4.97 4.50 4.48 4.48 4.47 4.46 4.45 3.22 3.21 3.19 3.18 3.15 3.14 3.12 3.10
O
HN
O
NO2
3o
1.92
1.96
0.98
0.99
0.97
1.00
2.96
4.96
1.98
1.97
f1 (ppm)

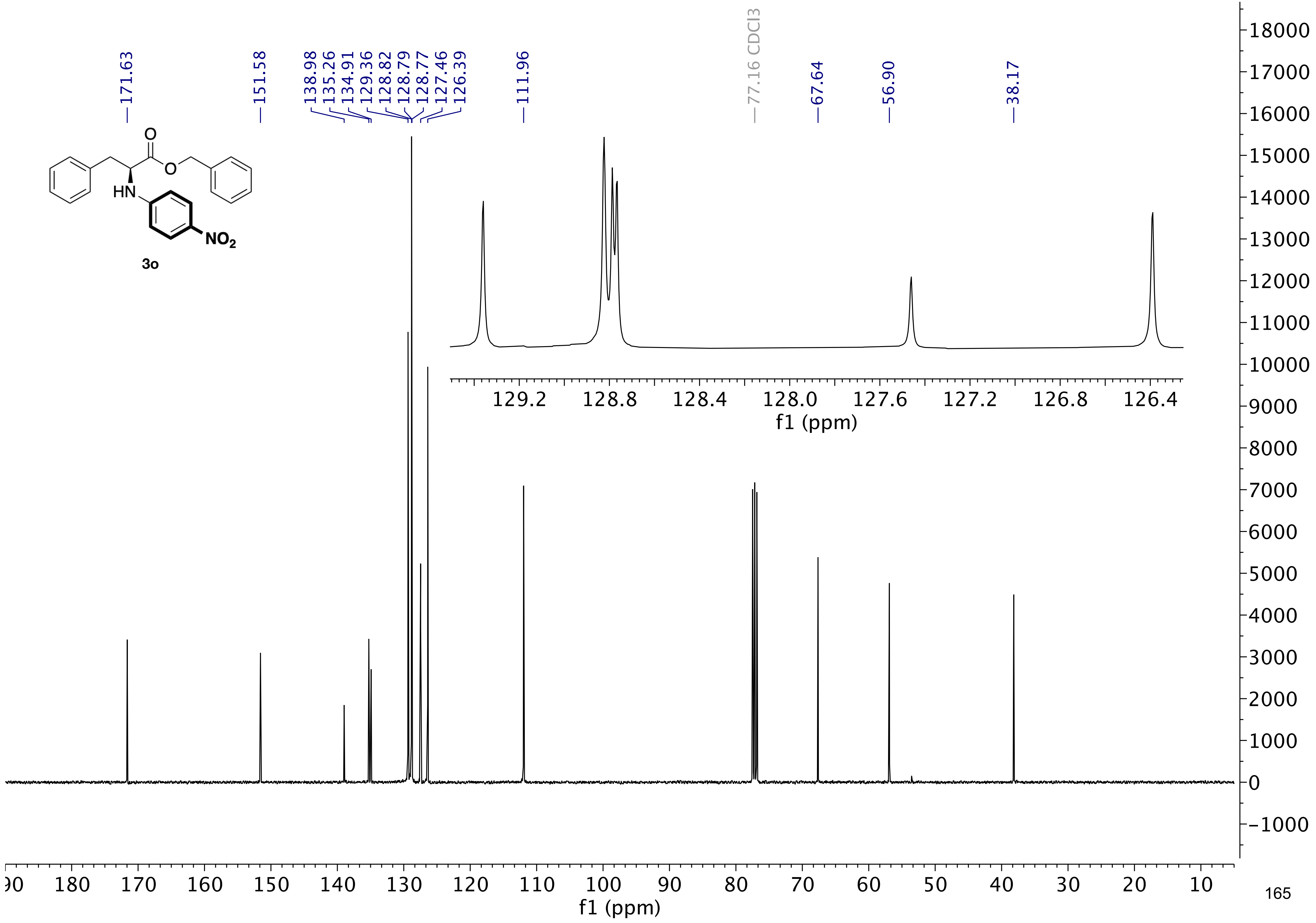

171.63
151.58
138.98
135.26
134.91
129.36
128.82
128.79
128.77
127.46
126.39
111.96
77.16 CDCl3
67.64
56.90
38.17
O
O
HN
NO2
3o
129.2
128.8
128.4
128.0
127.6
127.2
126.8
126.4
f1 (ppm)
18000
17000
16000
15000
14000
13000
12000
11000
10000
9000
8000
7000
6000
5000
4000
3000
2000
1000
0
-1000
180
170
160
150
140
130
120
110
100
90
80
70
60
50
40
30
20
10
f1 (ppm)

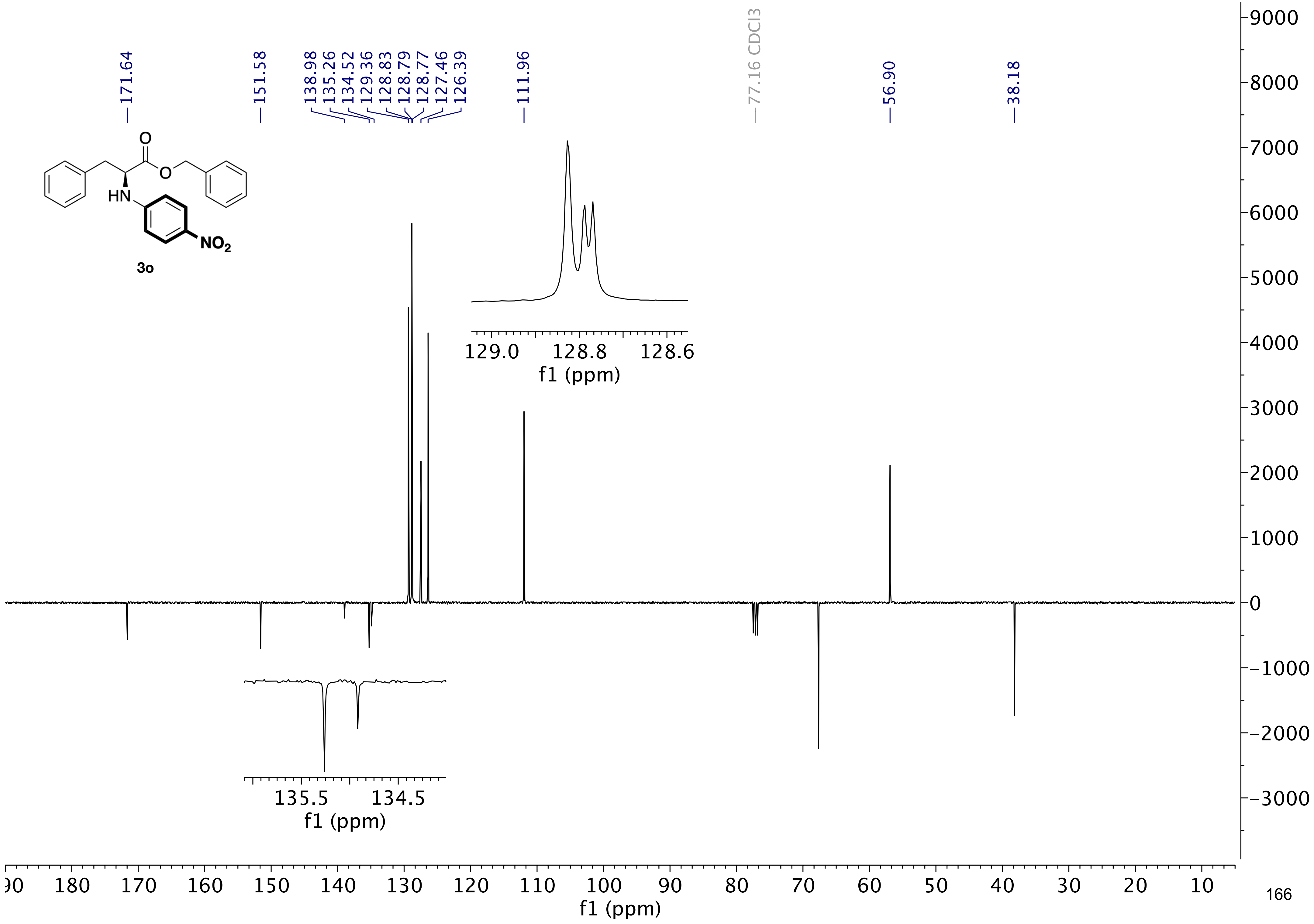
171.64
151.58
138.98
135.26
134.52
129.36
128.83
128.79
128.77
127.46
126.39
111.96
77.16 CDCl3
56.90
38.18
HN
O
O
NO_2
3o
129.0
128.8
128.6
f1 (ppm)
135.5
134.5
f1 (ppm)
9000
8000
7000
6000
5000
4000
3000
2000
1000
0
−1000
−2000
−3000
180
170
160
150
140
130
120
110
100
90
80
70
60
50
40
30
20
10
f1 (ppm)

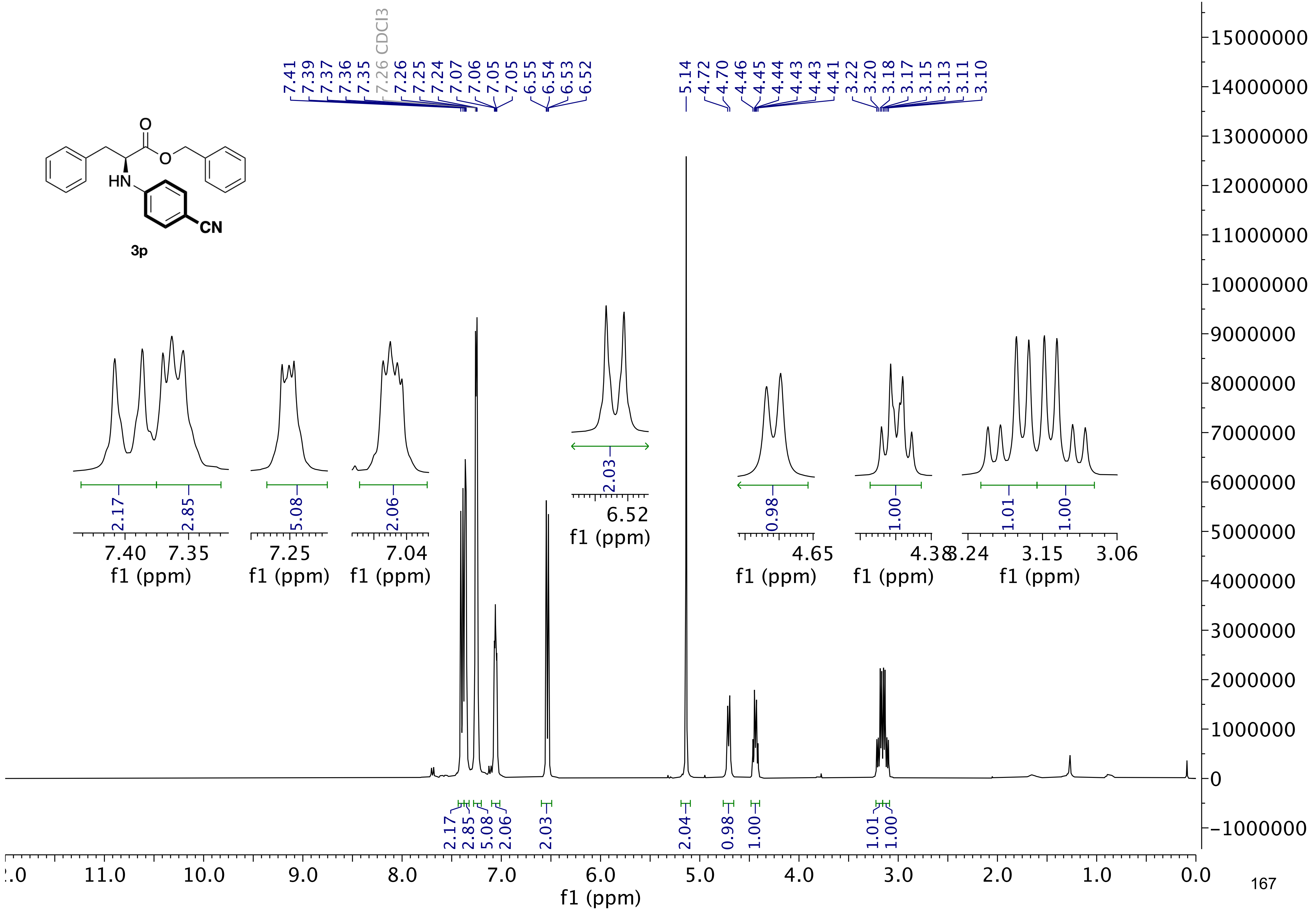

7.41
7.39
7.37
7.36
7.35
7.26 CDCl3
7.26
7.25
7.24
7.07
7.06
7.05
7.05
6.55
6.54
6.53
6.52
5.14
4.72
4.70
4.46
4.45
4.44
4.43
4.43
4.41
3.22
3.20
3.18
3.17
3.15
3.13
3.11
3.10
O
O
HN
CN
3p
2.17
2.85
5.08
2.06
2.03
2.04
0.98
1.00
1.01
1.00
f1 (ppm)

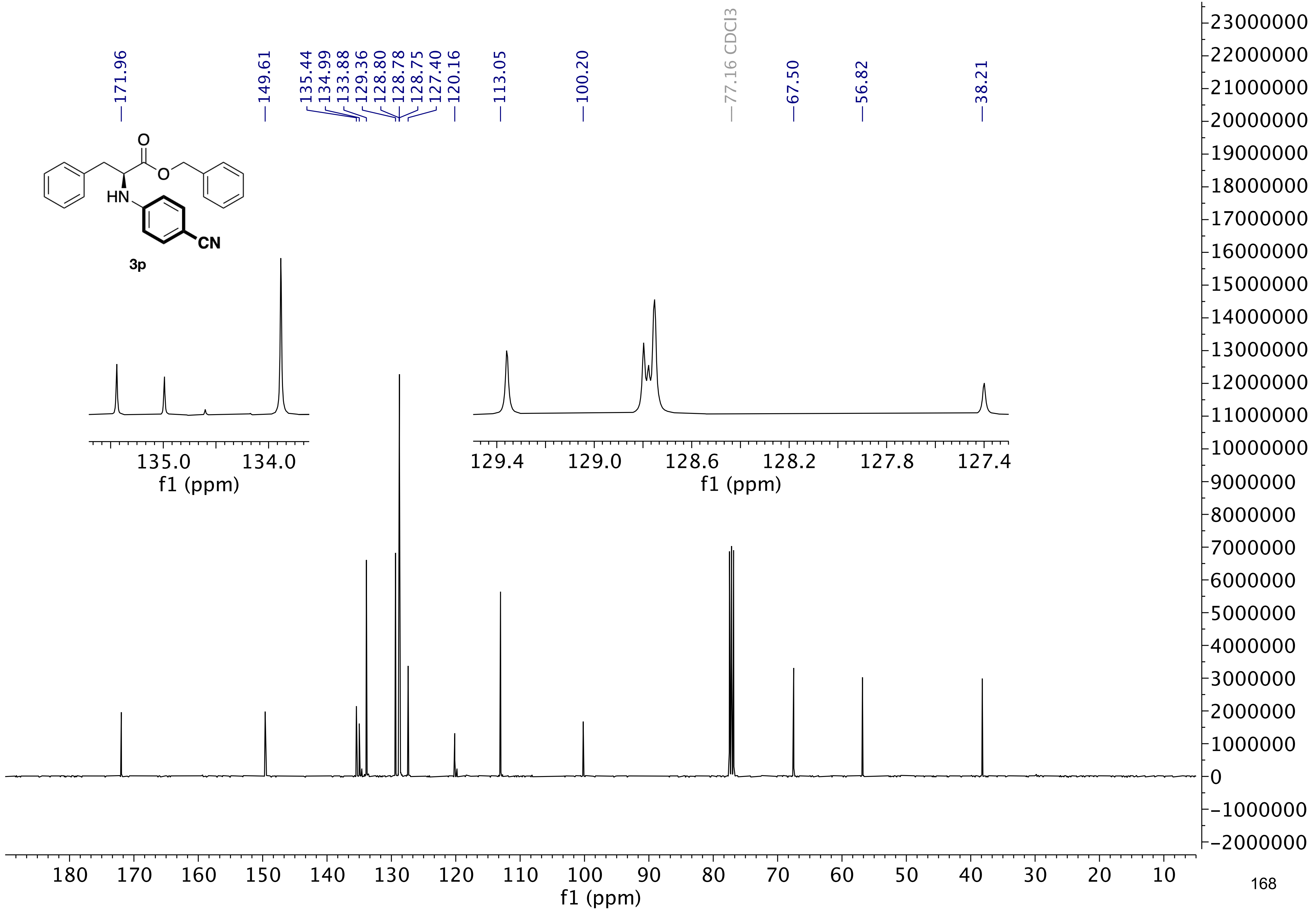

171.96
149.61
135.44
134.99
133.88
129.36
128.80
128.78
128.75
127.40
120.16
113.05
100.20
77.16 CDCl3
67.50
56.82
38.21
O
O
HN
CN
3p
135.0
134.0
f1 (ppm)
129.4
129.0
128.6
128.2
127.8
127.4
f1 (ppm)
180
170
160
150
140
130
120
110
100
90
80
70
60
50
40
30
20
10
f1 (ppm)

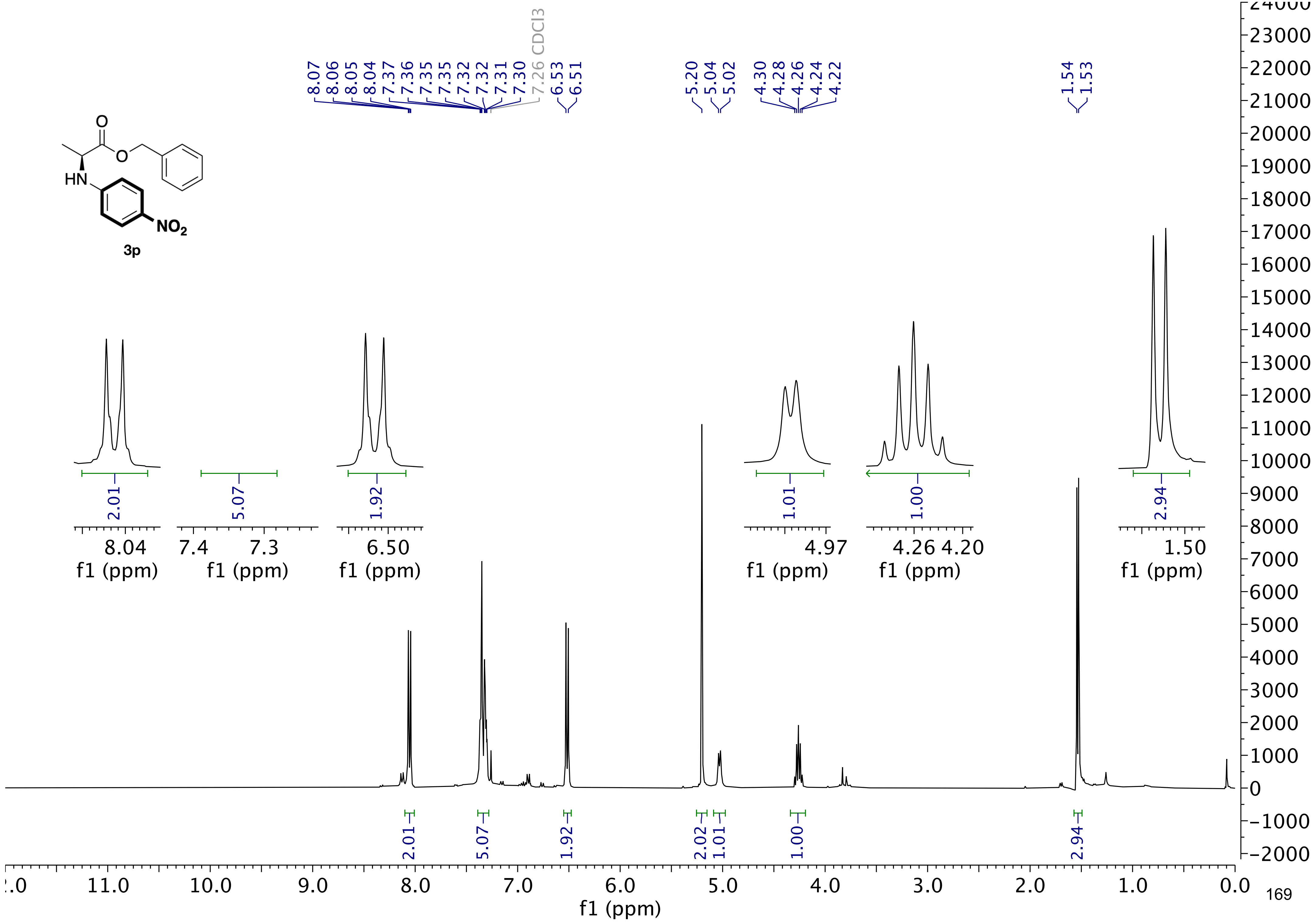

8.07 8.06 8.05 8.04 7.37 7.36 7.35 7.35 7.32 7.32 7.31 7.30 7.26 CDCl3 6.53 6.51
5.20 5.04 5.02
4.30 4.28 4.26 4.24 4.22
1.54 1.53
O
O
HN
NO2
3p
2.01
5.07
1.92
1.01
1.00
2.94
2.02
f1 (ppm)

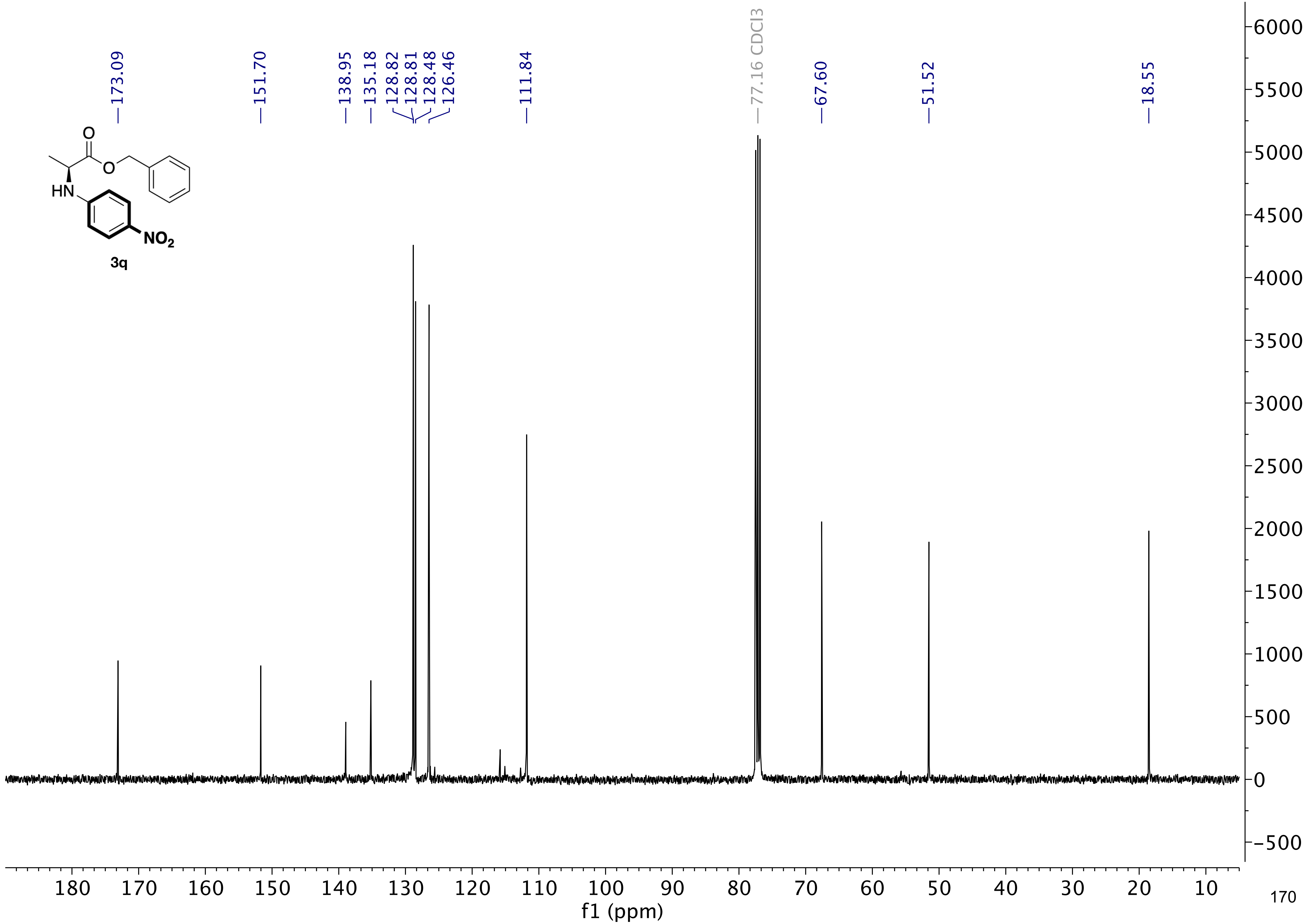
173.09
151.70
138.95
135.18
128.82
128.81
128.48
126.46
111.84
77.16 CDCl3
67.60
51.52
18.55
O
O
HN
NO2
3q
f1 (ppm)

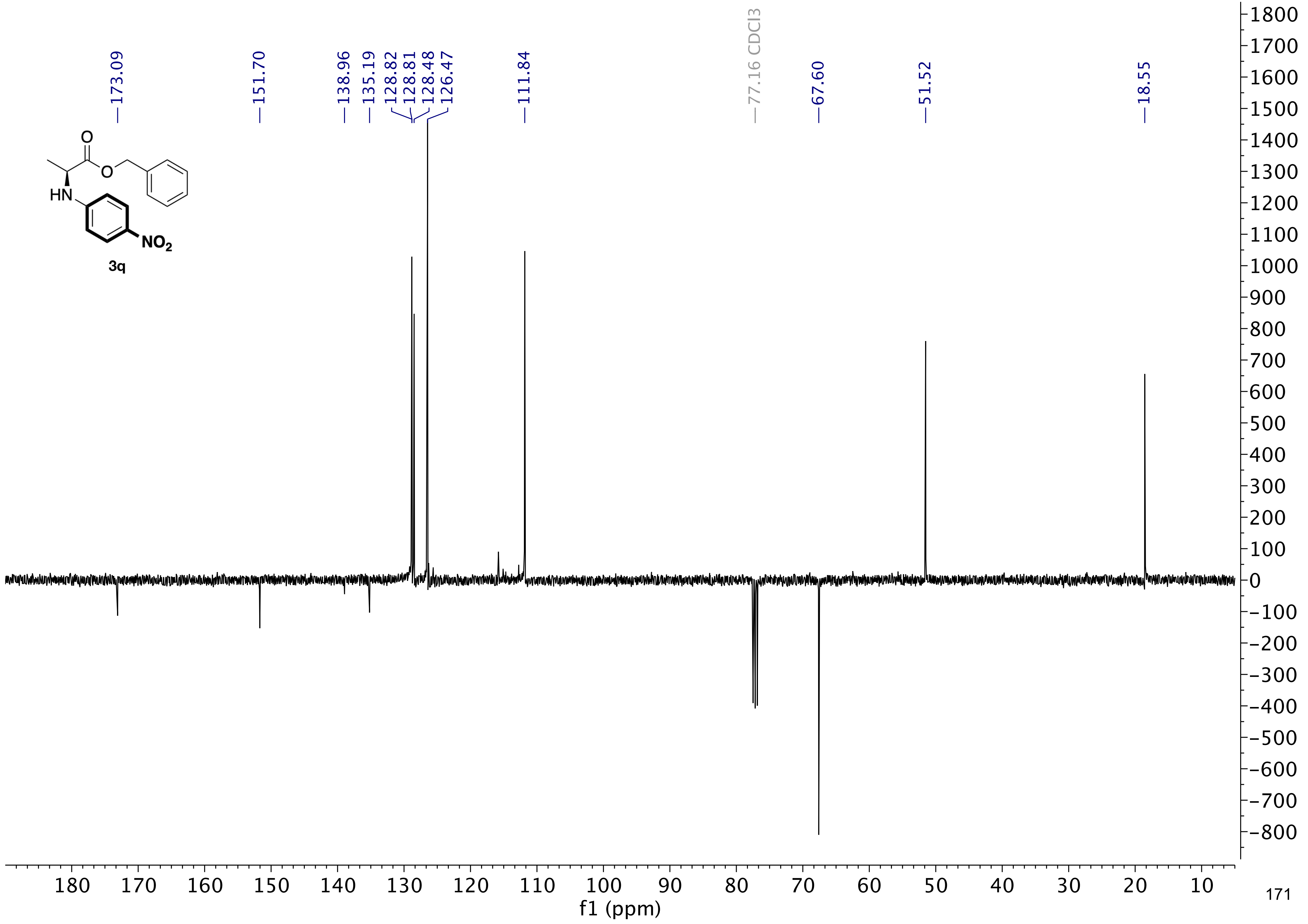

173.09
151.70
138.96
135.19
128.82
128.81
128.48
126.47
111.84
77.16 CDCl3
67.60
51.52
18.55
O
O
HN
NO2
3q
f1 (ppm)

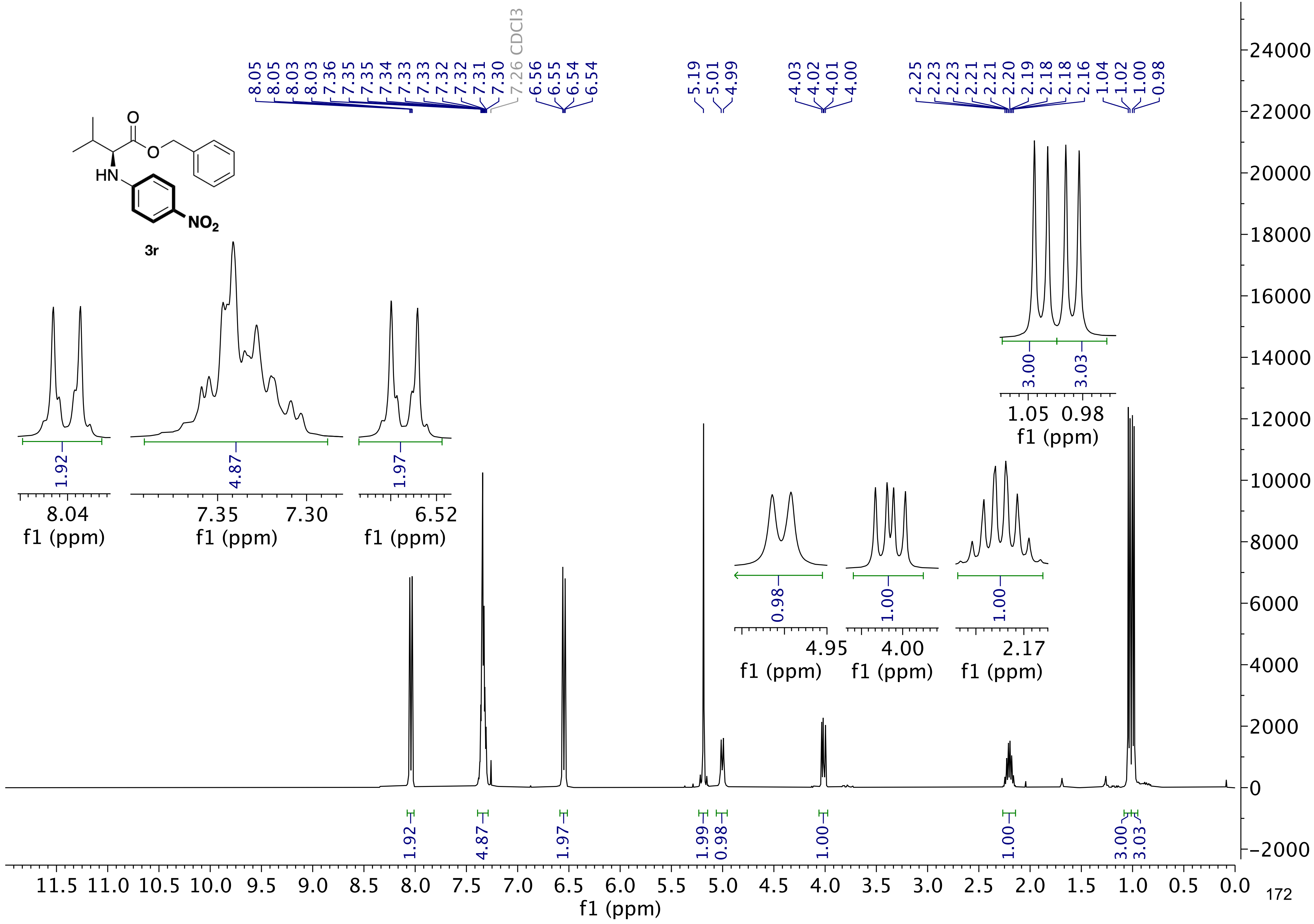

8.05
8.05
8.03
8.03
7.36
7.35
7.35
7.34
7.33
7.33
7.32
7.32
7.31
7.30
7.26 CDCl3
6.56
6.55
6.54
6.54
5.19
5.01
4.99
4.03
4.02
4.01
4.00
2.25
2.23
2.23
2.21
2.21
2.20
2.19
2.18
2.18
2.16
1.04
1.02
1.00
0.98
O
O
HN
NO2
3r
1.92
4.87
1.97
1.99
0.98
1.00
1.00
3.00
3.03
f1 (ppm)

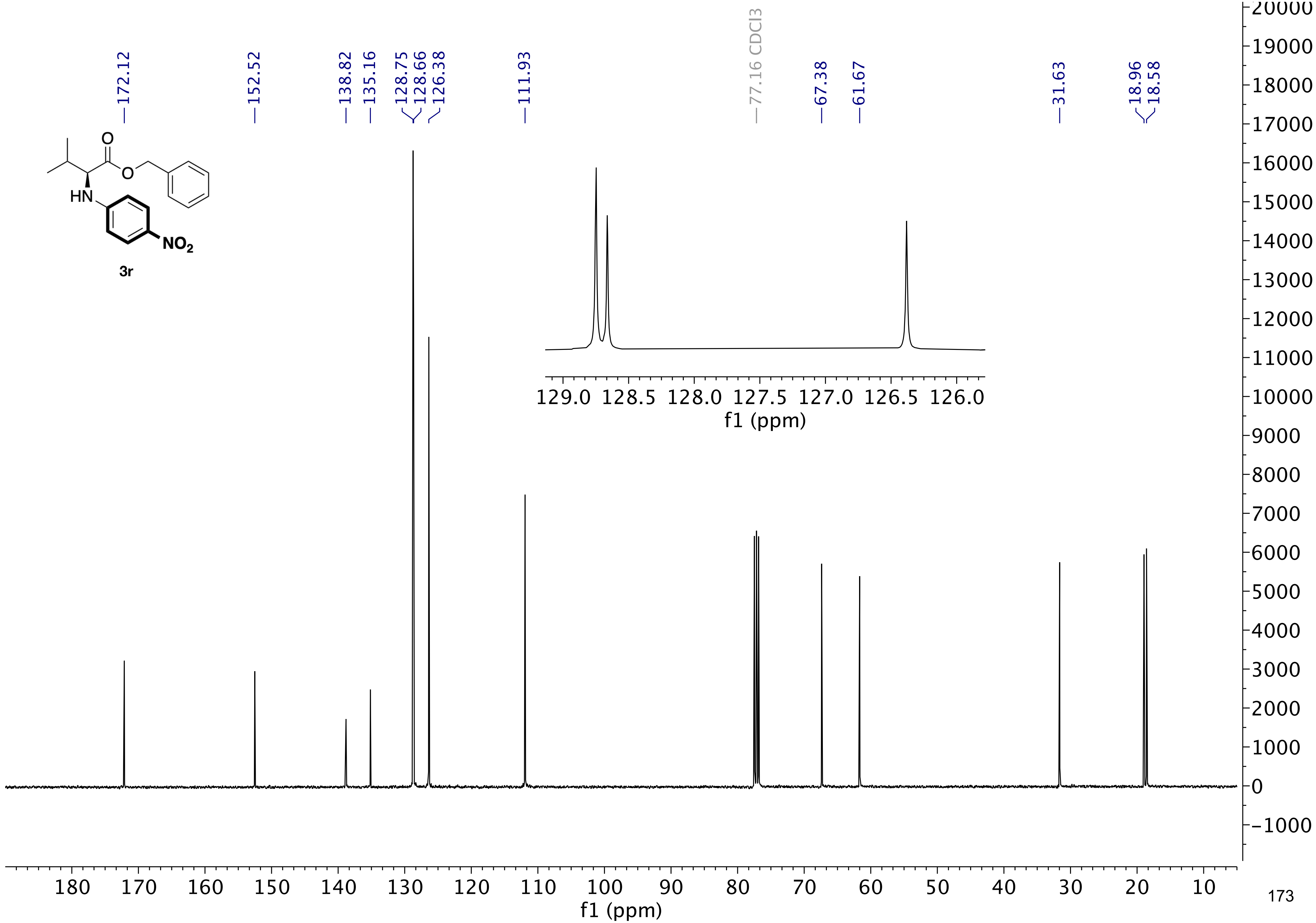
172.12
152.52
138.82
135.16
128.75
128.66
126.38
111.93
77.16 CDCl3
67.38
61.67
31.63
18.96
18.58
HN
O
O
NO2
3r
129.0 128.5 128.0 127.5 127.0 126.5 126.0
f1 (ppm)
f1 (ppm)

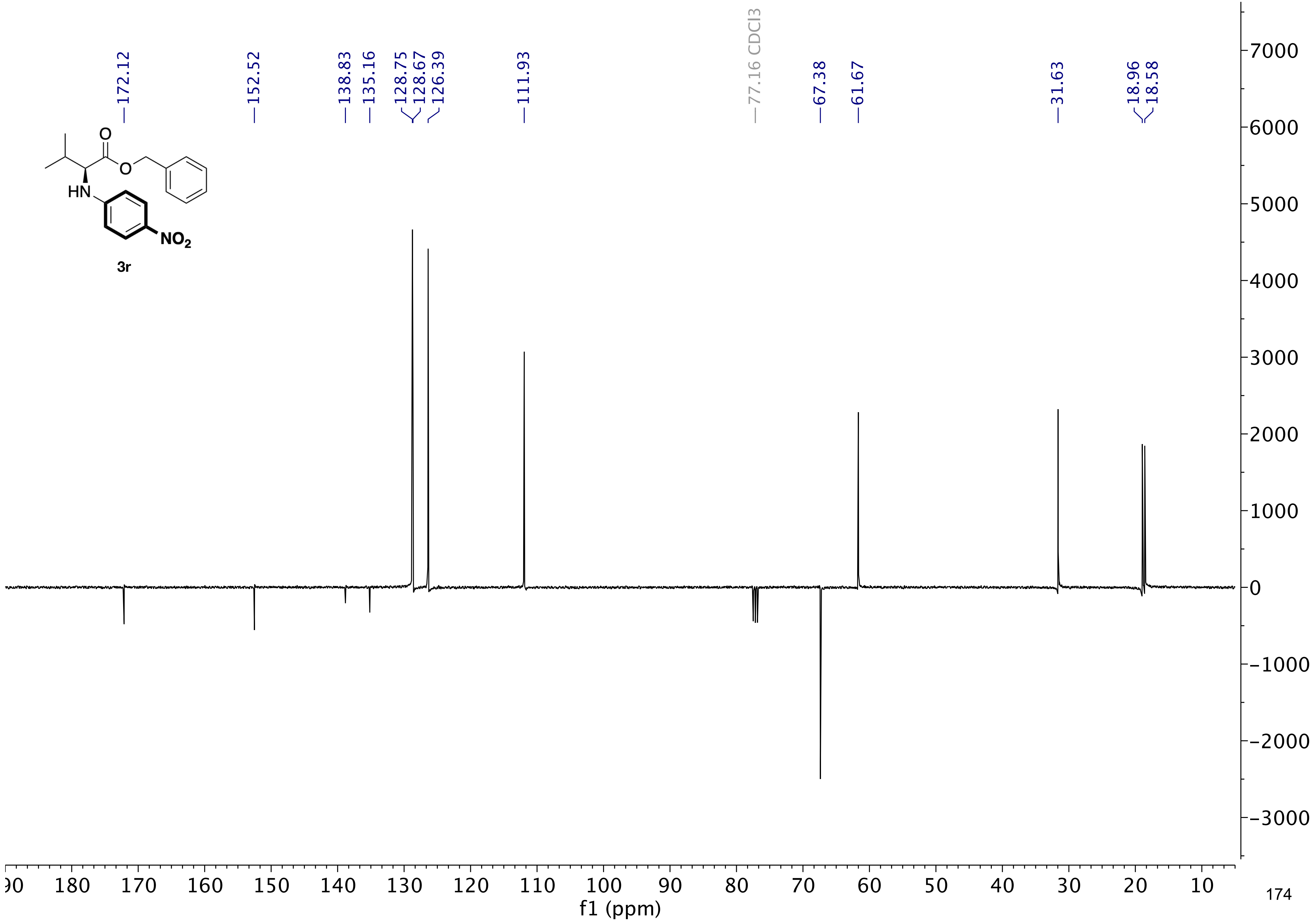

172.12
152.52
138.83
135.16
128.75
128.67
126.39
111.93
77.16 CDCl3
67.38
61.67
31.63
18.96
18.58
O
O
HN
NO2
3r
7000
6000
5000
4000
3000
2000
1000
0
−1000
−2000
−3000
180
170
160
150
140
130
120
110
100
90
80
70
60
50
40
30
20
10
f1 (ppm)

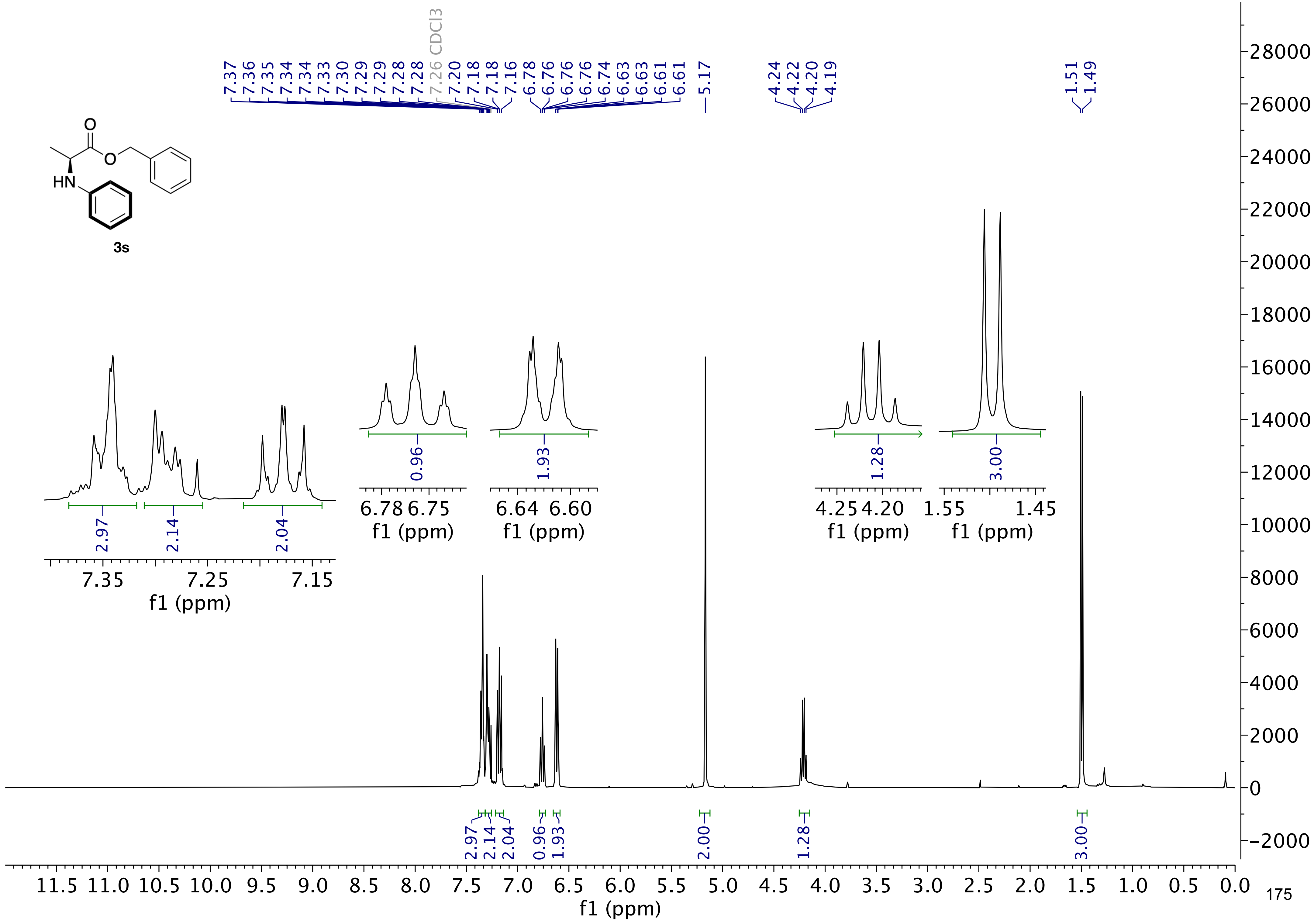

7.37 7.36 7.35 7.34 7.34 7.33 7.30 7.29 7.29 7.28 7.28 7.26 CDCl3 7.20 7.18 7.18 7.16 6.78 6.76 6.76 6.76 6.74 6.63 6.63 6.61 6.61 5.17 4.24 4.22 4.20 4.19 1.51 1.49
O
O
HN
3s
2.97 2.14 2.04 0.96 1.93 2.00 1.28 3.00
f1 (ppm)

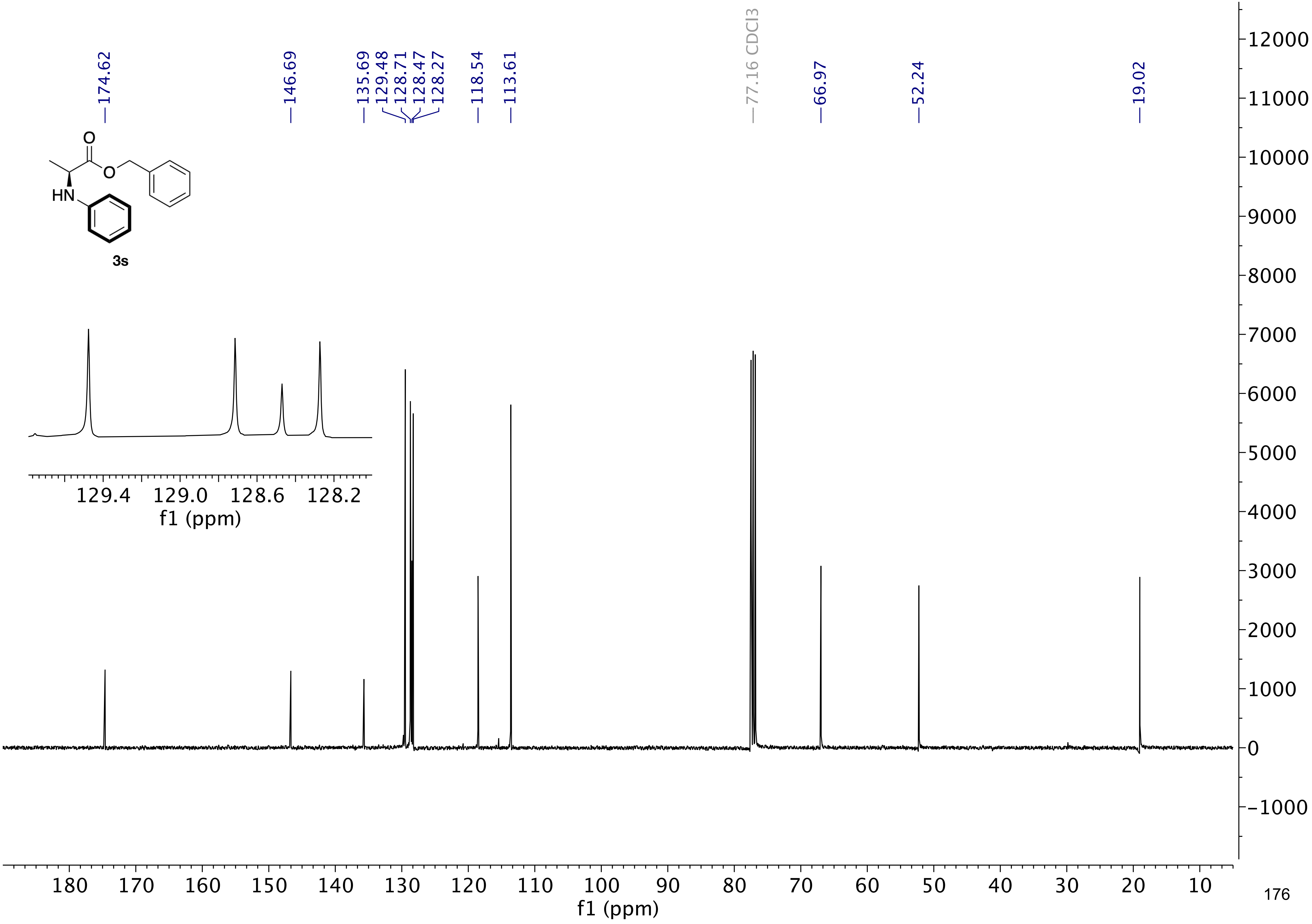
174.62
146.69
135.69
129.48
128.71
128.47
128.27
118.54
113.61
77.16 CDCl3
66.97
52.24
19.02
O
O
HN
3s
129.4
129.0
128.6
128.2
f1 (ppm)
12000
11000
10000
9000
8000
7000
6000
5000
4000
3000
2000
1000
0
−1000
180
170
160
150
140
130
120
110
100
90
80
70
60
50
40
30
20
10
f1 (ppm)

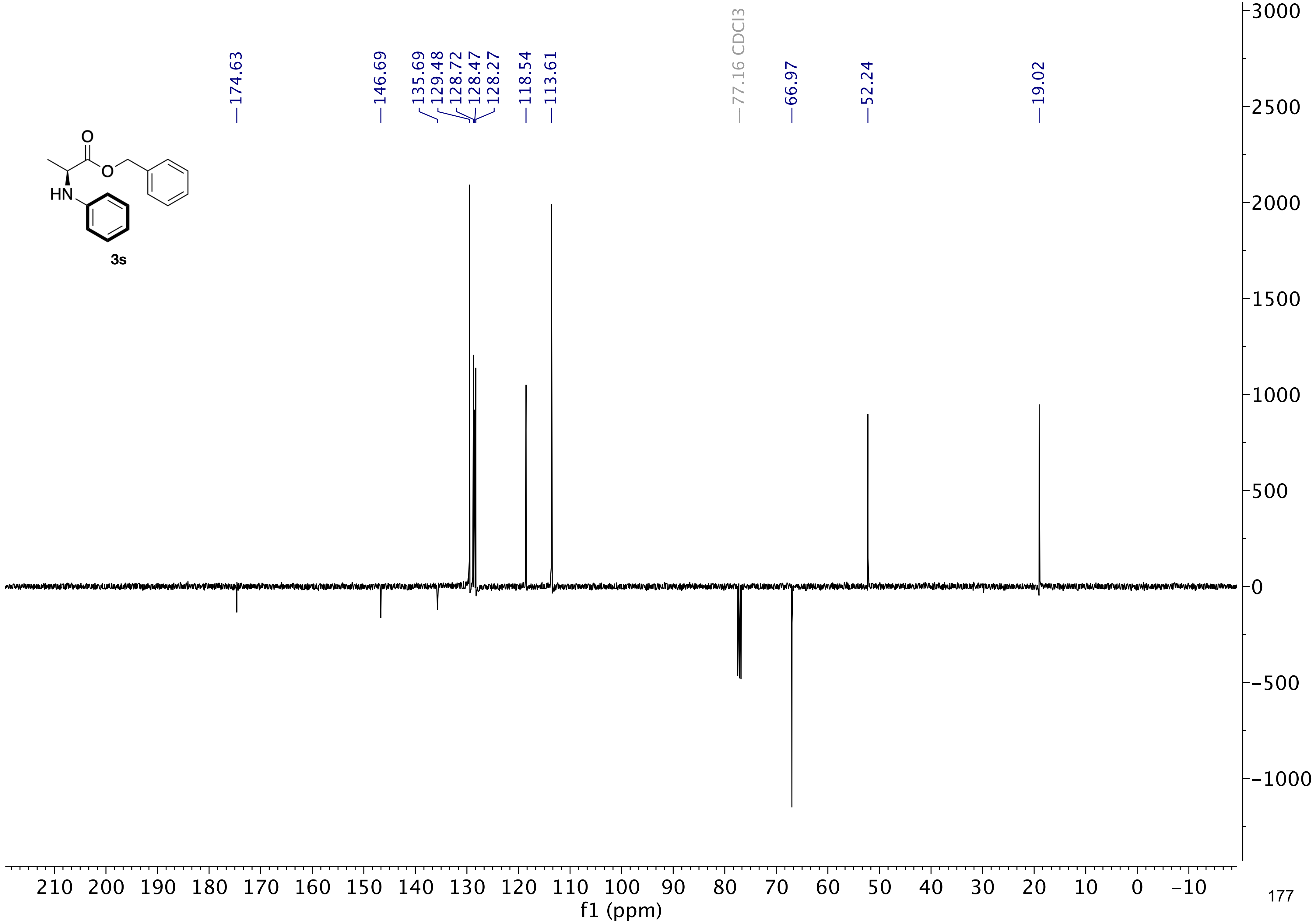

174.63
146.69
135.69
129.48
128.72
128.47
128.27
118.54
113.61
77.16 CDCl3
66.97
52.24
19.02
O
O
HN
3s
f1 (ppm)

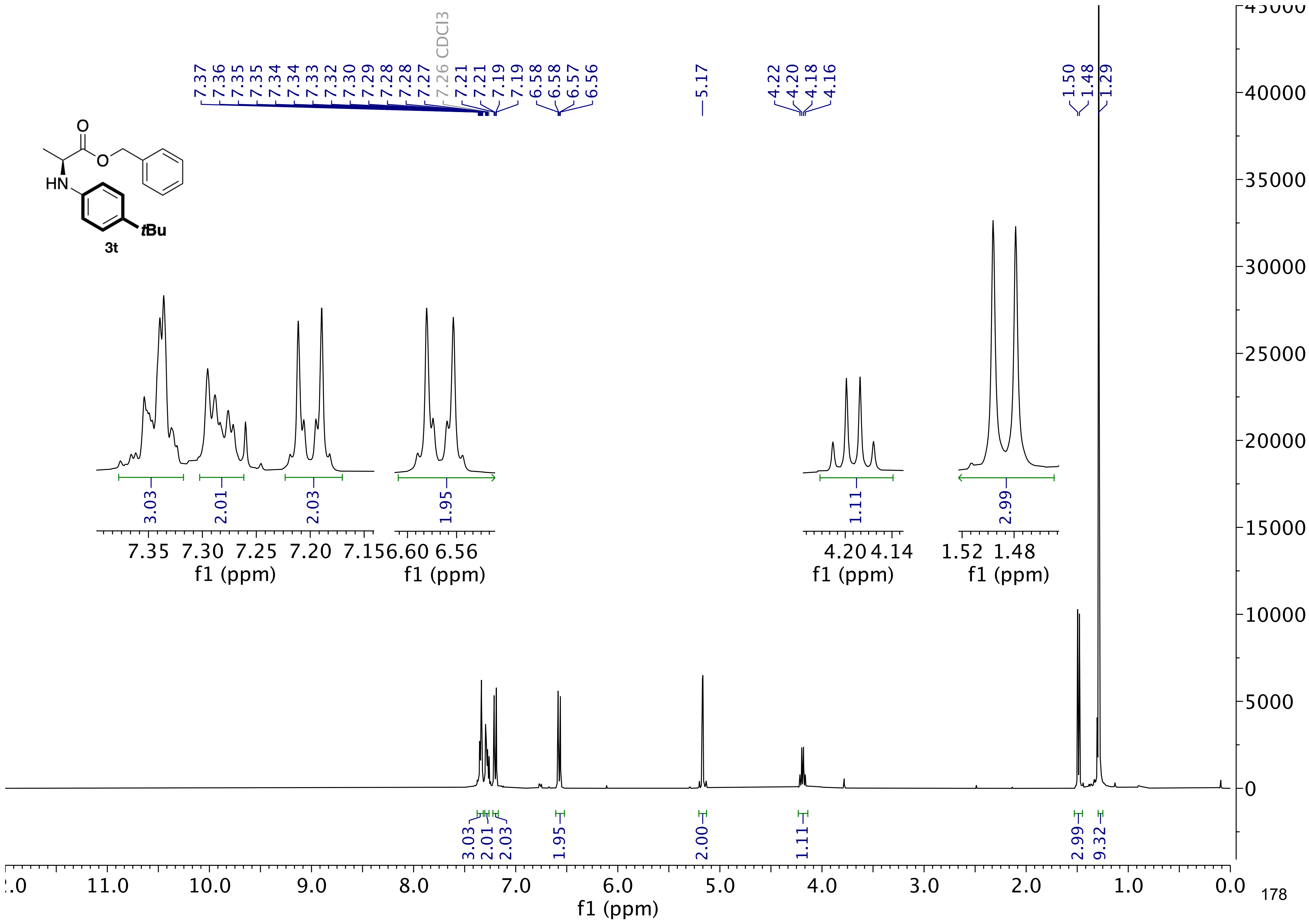

HN
O
O
tBu
3t
7.37 7.36 7.35 7.35 7.34 7.34 7.33 7.32 7.30 7.29 7.28 7.28 7.27 7.26 CDCl3 7.21 7.21 7.19 7.19 6.58 6.58 6.57 6.56
5.17
4.22 4.20 4.18 4.16
1.50 1.48 1.29
3.03 2.01 2.03 1.95 2.00 1.11 2.99 9.32
f1 (ppm)

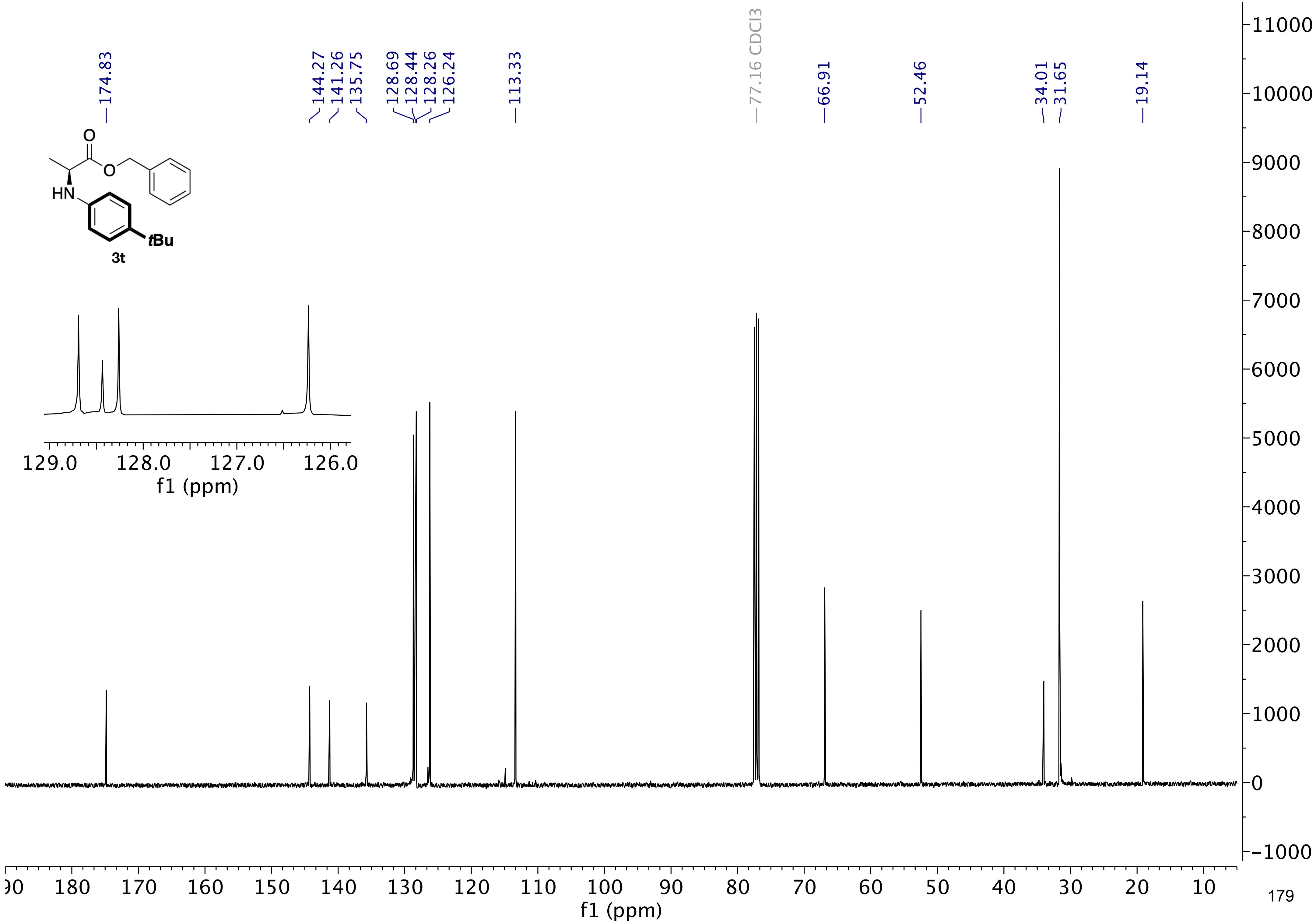

174.83
144.27
141.26
135.75
128.69
128.44
128.26
126.24
113.33
77.16 CDCl3
66.91
52.46
34.01
31.65
19.14
O
O
HN
tBu
3t
129.0
128.0
127.0
126.0
f1 (ppm)
11000
10000
9000
8000
7000
6000
5000
4000
3000
2000
1000
0
−1000
180
170
160
150
140
130
120
110
100
90
80
70
60
50
40
30
20
10
f1 (ppm)

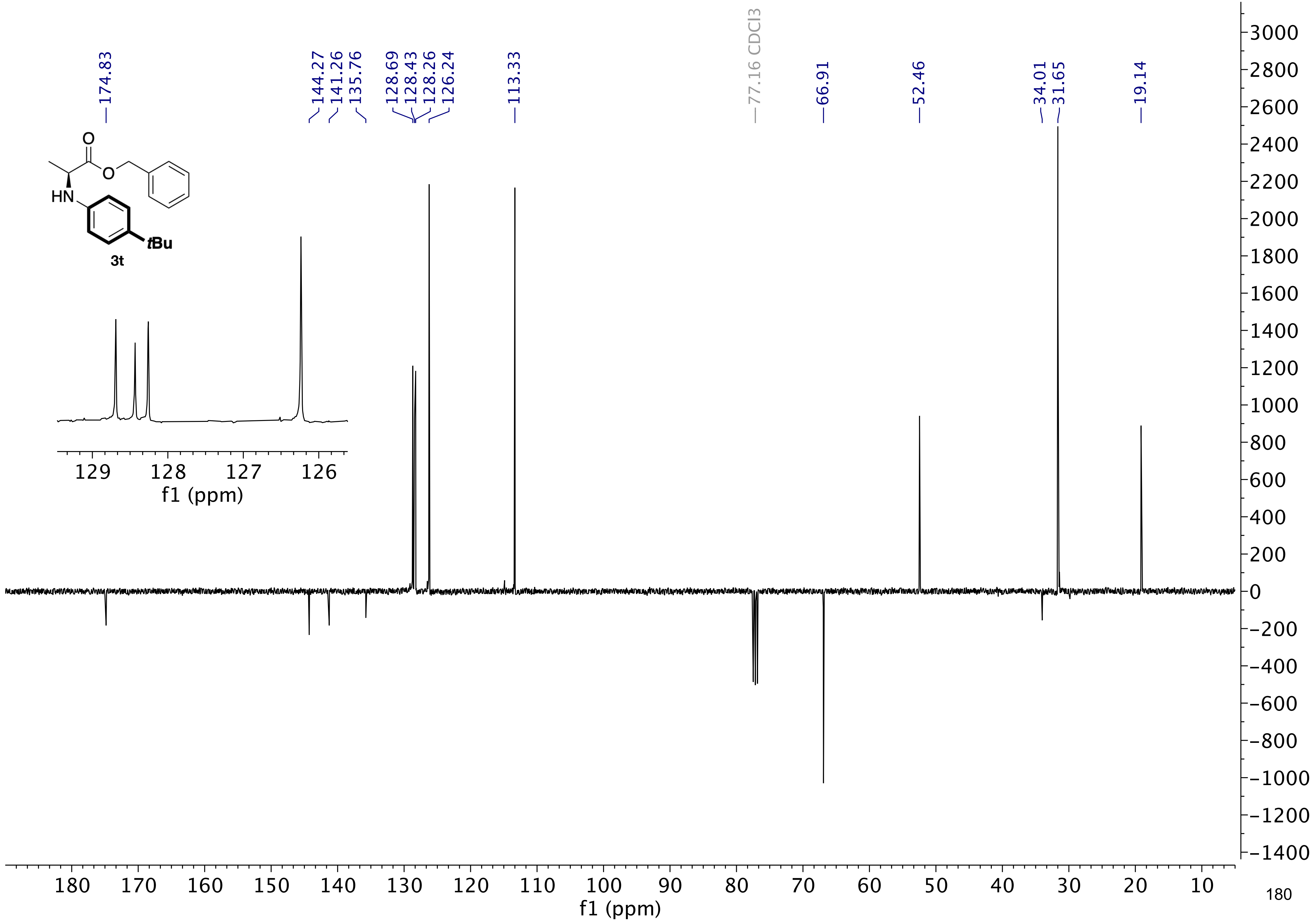

174.83
144.27
141.26
135.76
128.69
128.43
128.26
126.24
113.33
77.16 CDCl3
66.91
52.46
34.01
31.65
19.14
O
O
HN
tBu
3t
129
128
127
126
f1 (ppm)
3000
2800
2600
2400
2200
2000
1800
1600
1400
1200
1000
800
600
400
200
0
−200
−400
−600
−800
−1000
−1200
−1400
180
170
160
150
140
130
120
110
100
90
80
70
60
50
40
30
20
10
f1 (ppm)

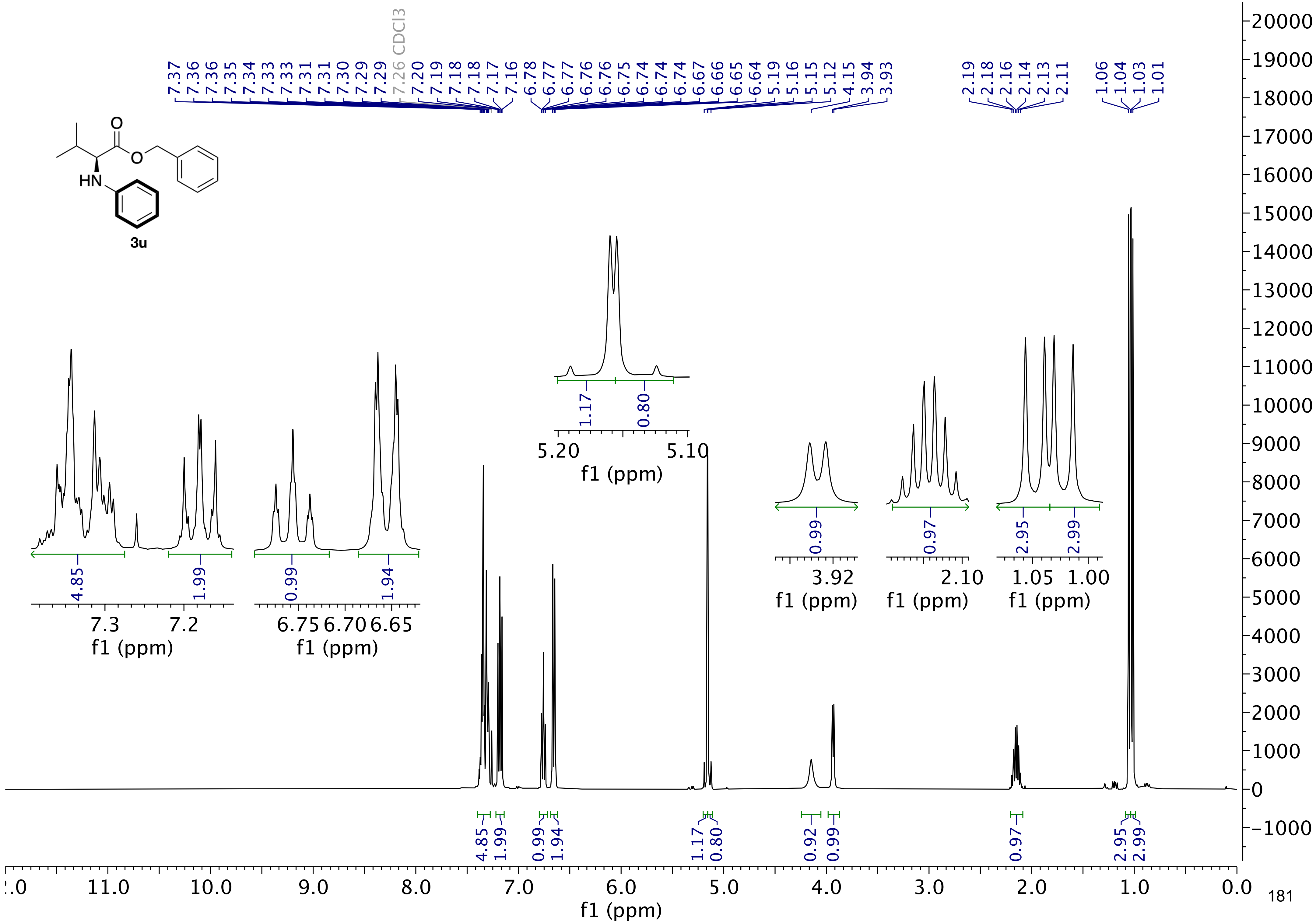

HN
O
O
3u
CDCl3
f1 (ppm)

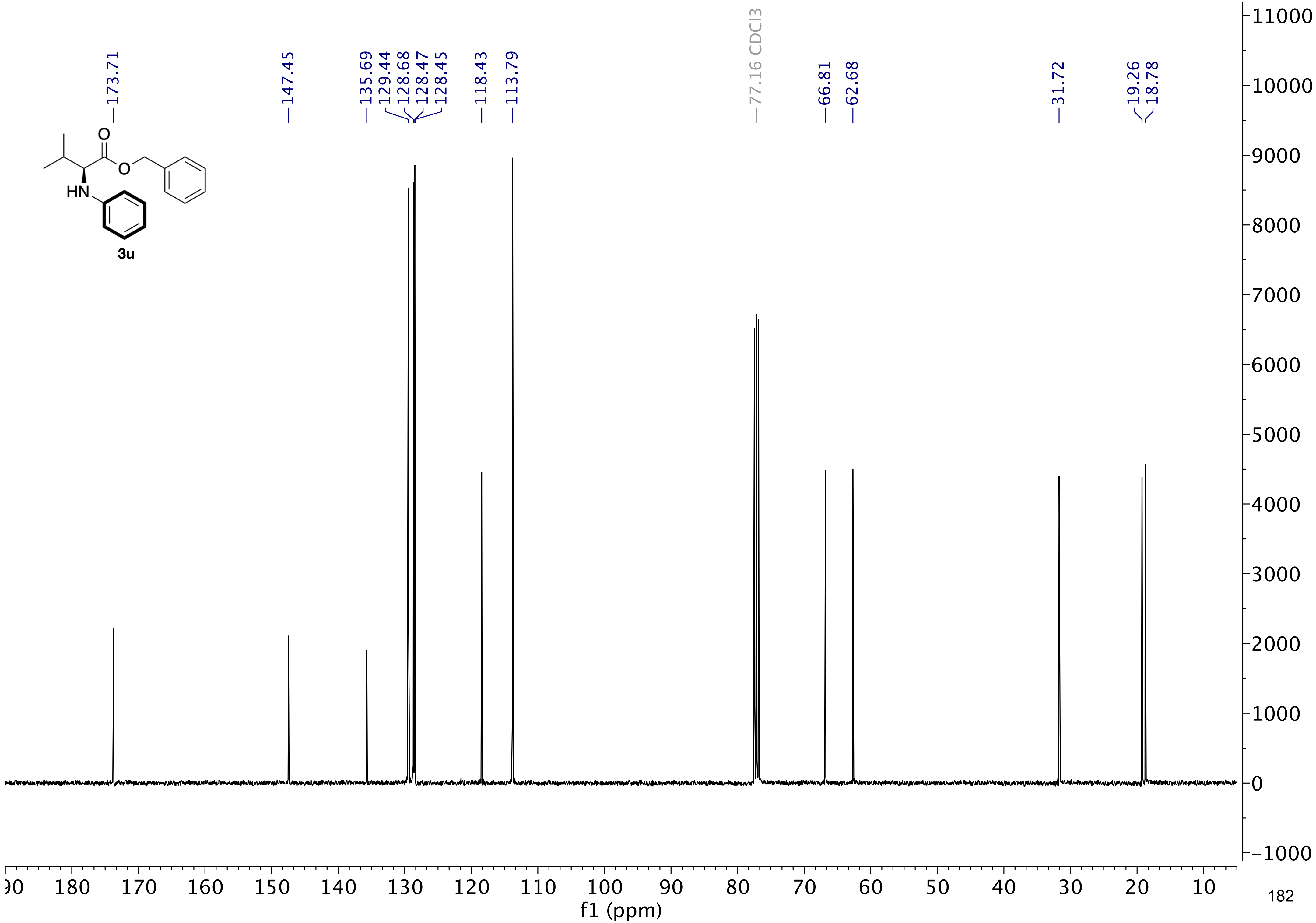
173.71
147.45
135.69
129.44
128.68
128.47
128.45
118.43
113.79
77.16 CDCl3
66.81
62.68
31.72
19.26
18.78
3u
f1 (ppm)

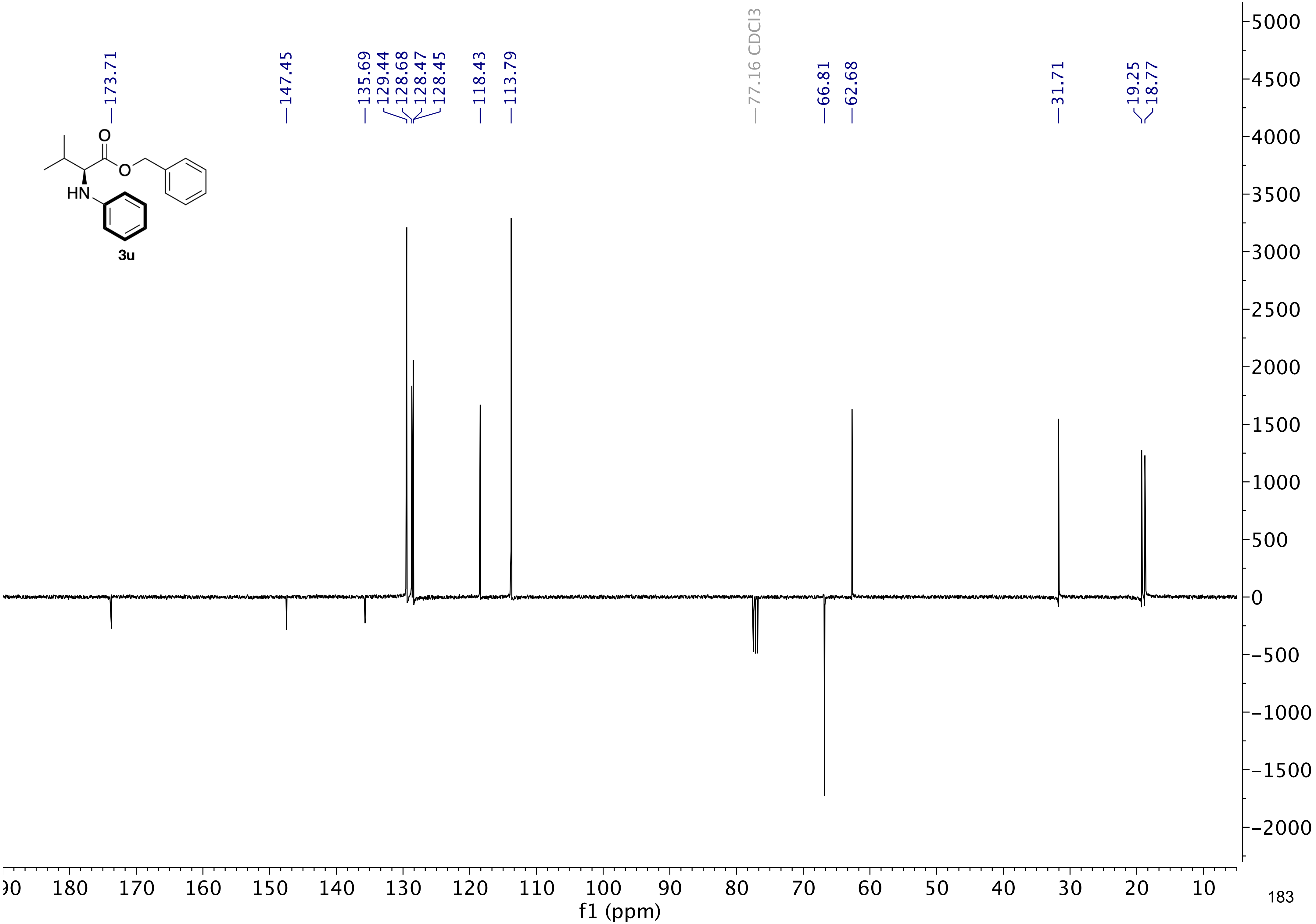

173.71
147.45
135.69
129.44
128.68
128.47
128.45
118.43
113.79
77.16 CDCl3
66.81
62.68
31.71
19.25
18.77
HN
O
O
3u
f1 (ppm)

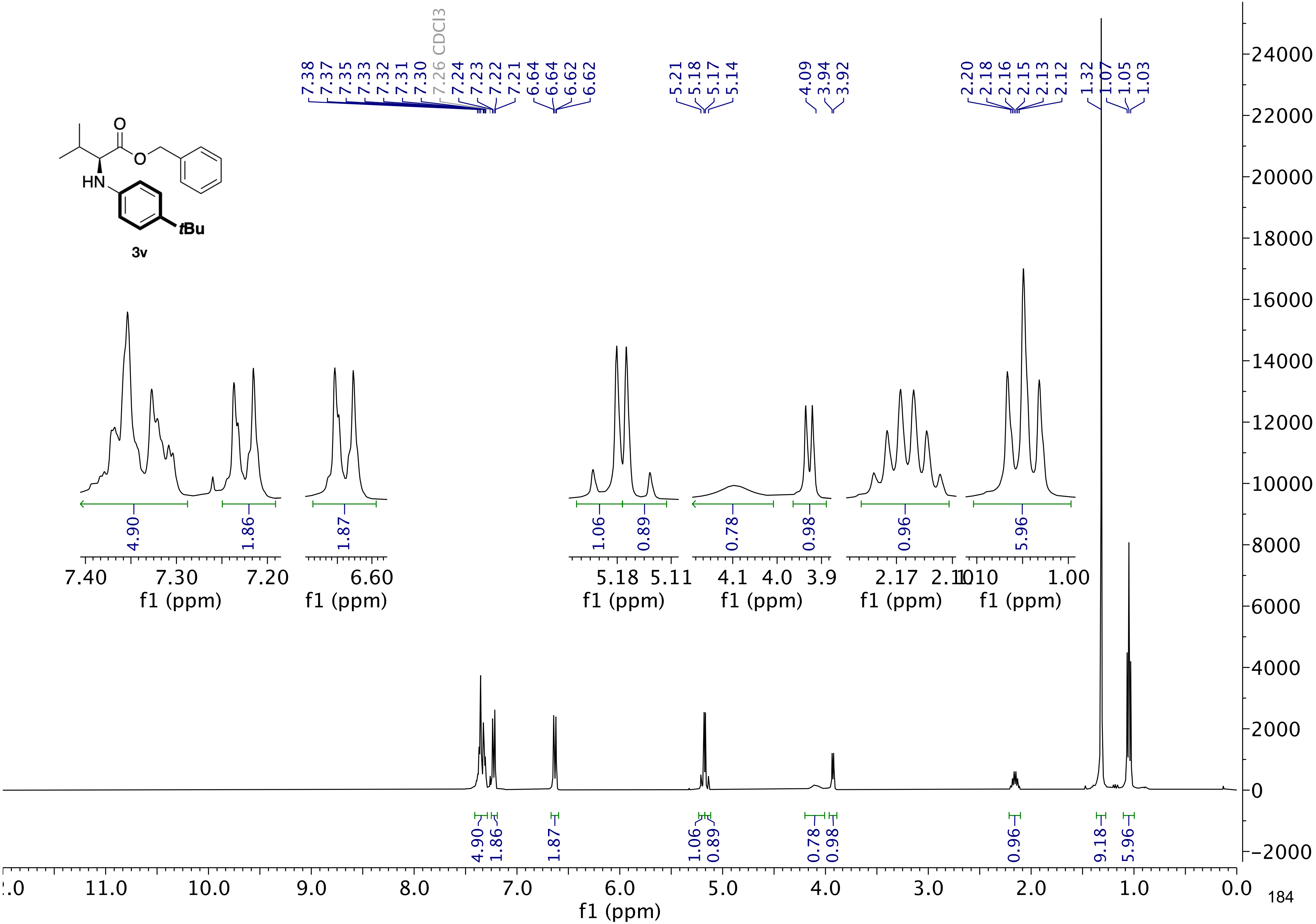
7.38 7.37 7.35 7.33 7.32 7.31 7.30 7.26 CDCl3 7.24 7.23 7.22 7.21 6.64 6.64 6.62 6.62
5.21 5.18 5.17 5.14
4.09 3.94 3.92
2.20 2.18 2.16 2.15 2.13 2.12 1.32 1.07 1.05 1.03
HN
O
O
tBu
3v
4.90 1.86 1.87 1.06 0.89 0.78 0.98 0.96 5.96
9.18
f1 (ppm)
24000 22000 20000 18000 16000 14000 12000 10000 8000 6000 4000 2000 0 −2000

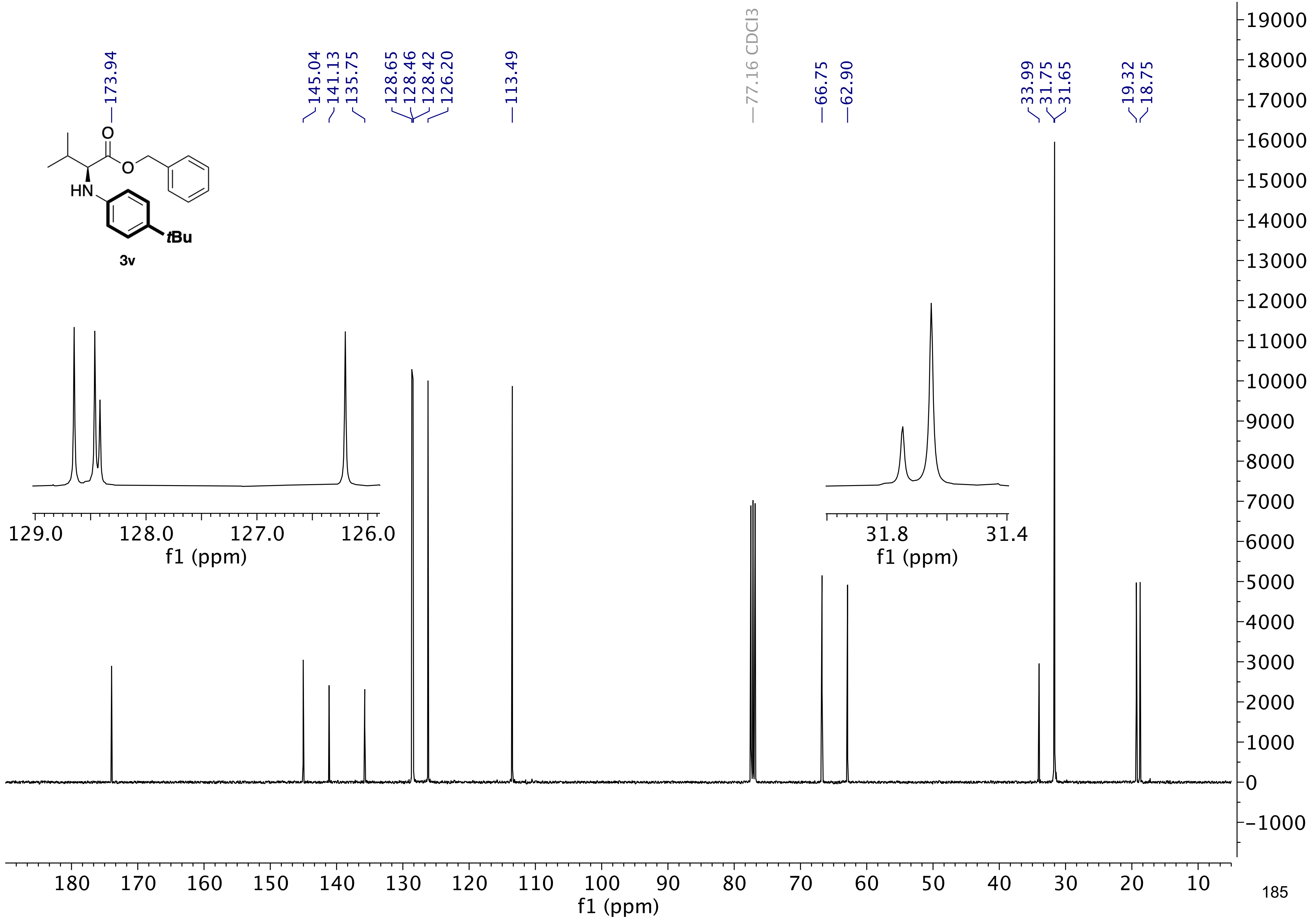
173.94
145.04
141.13
135.75
128.65
128.46
128.42
126.20
113.49
77.16 CDCl3
66.75
62.90
33.99
31.75
31.65
19.32
18.75
O
O
HN
tBu
3v
f1 (ppm)

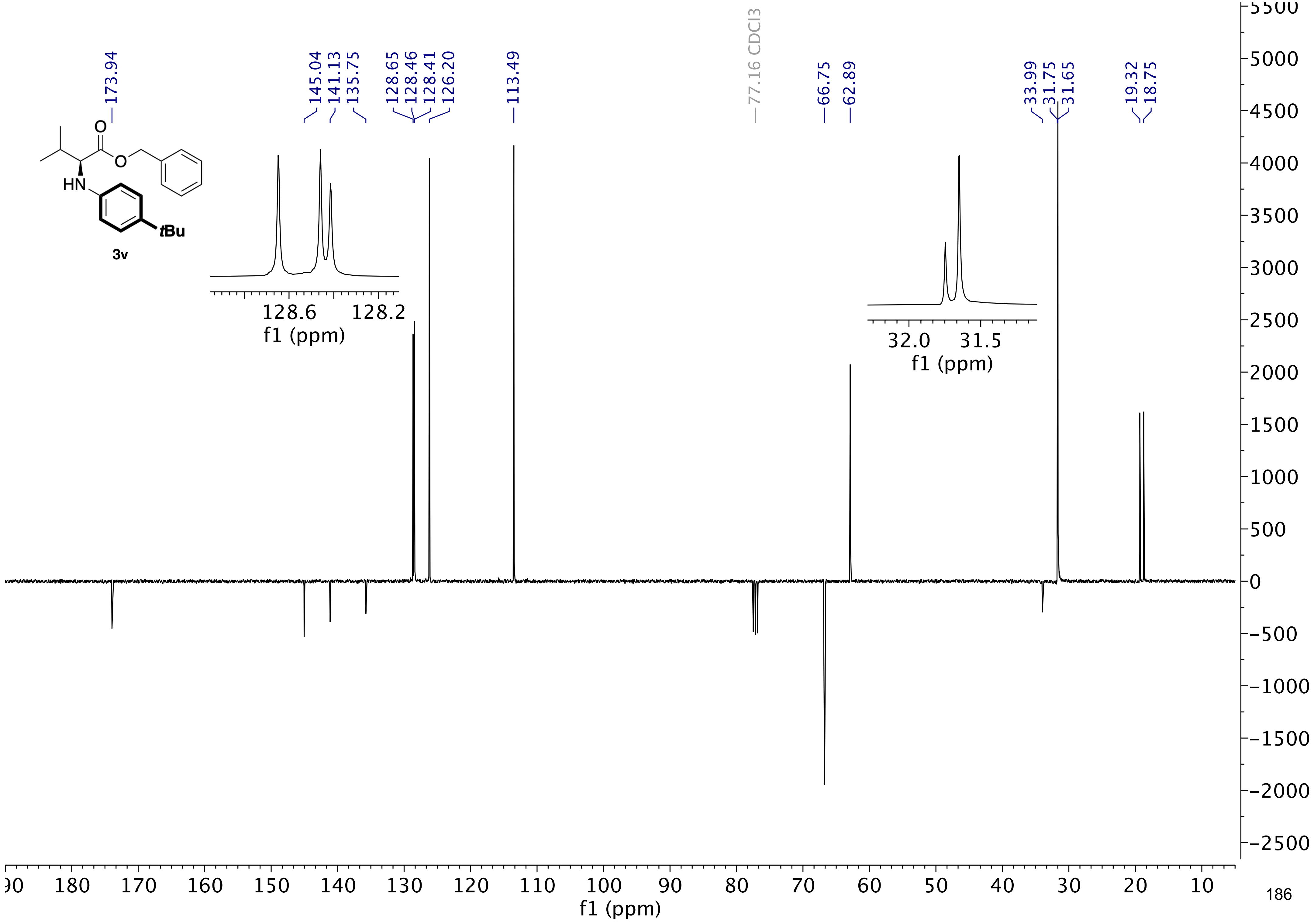

173.94
145.04
141.13
135.75
128.65
128.46
128.41
126.20
113.49
77.16 CDCl3
66.75
62.89
33.99
31.75
31.65
19.32
18.75
O
O
HN
tBu
3v
128.6
128.2
f1 (ppm)
32.0
31.5
f1 (ppm)
f1 (ppm)

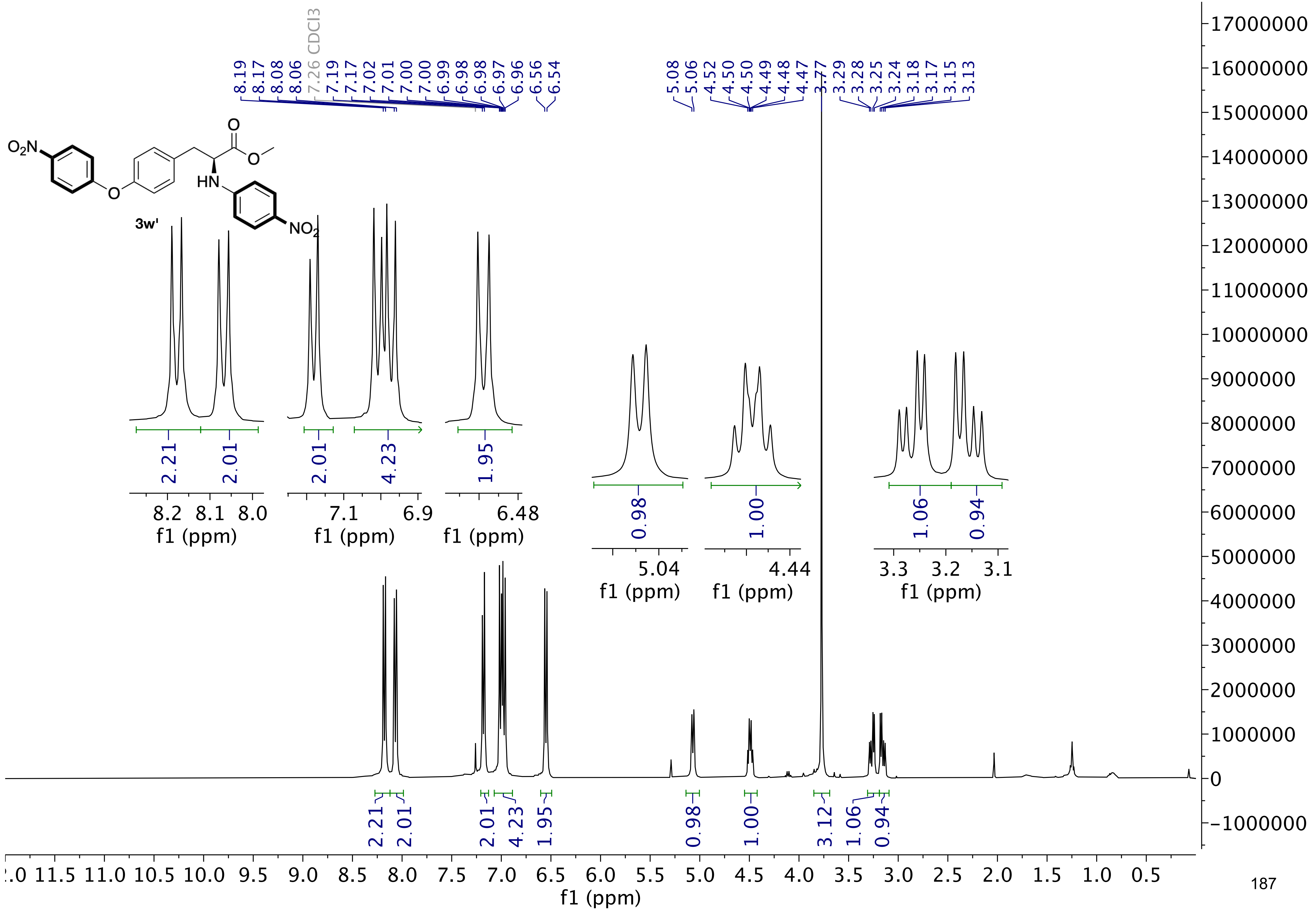

O2N
HN
NO2
O
3w'
7.26 CDCl3
8.19 8.17 8.08 8.06 7.19 7.17 7.02 7.01 7.00 7.00 6.99 6.98 6.98 6.97 6.96 6.56 6.54
5.08 5.06 4.52 4.50 4.50 4.49 4.48 4.47 3.77 3.29 3.28 3.25 3.24 3.18 3.17 3.15 3.13
2.21 2.01 2.01 4.23 1.95 0.98 1.00 3.12 1.06 0.94
f1 (ppm)

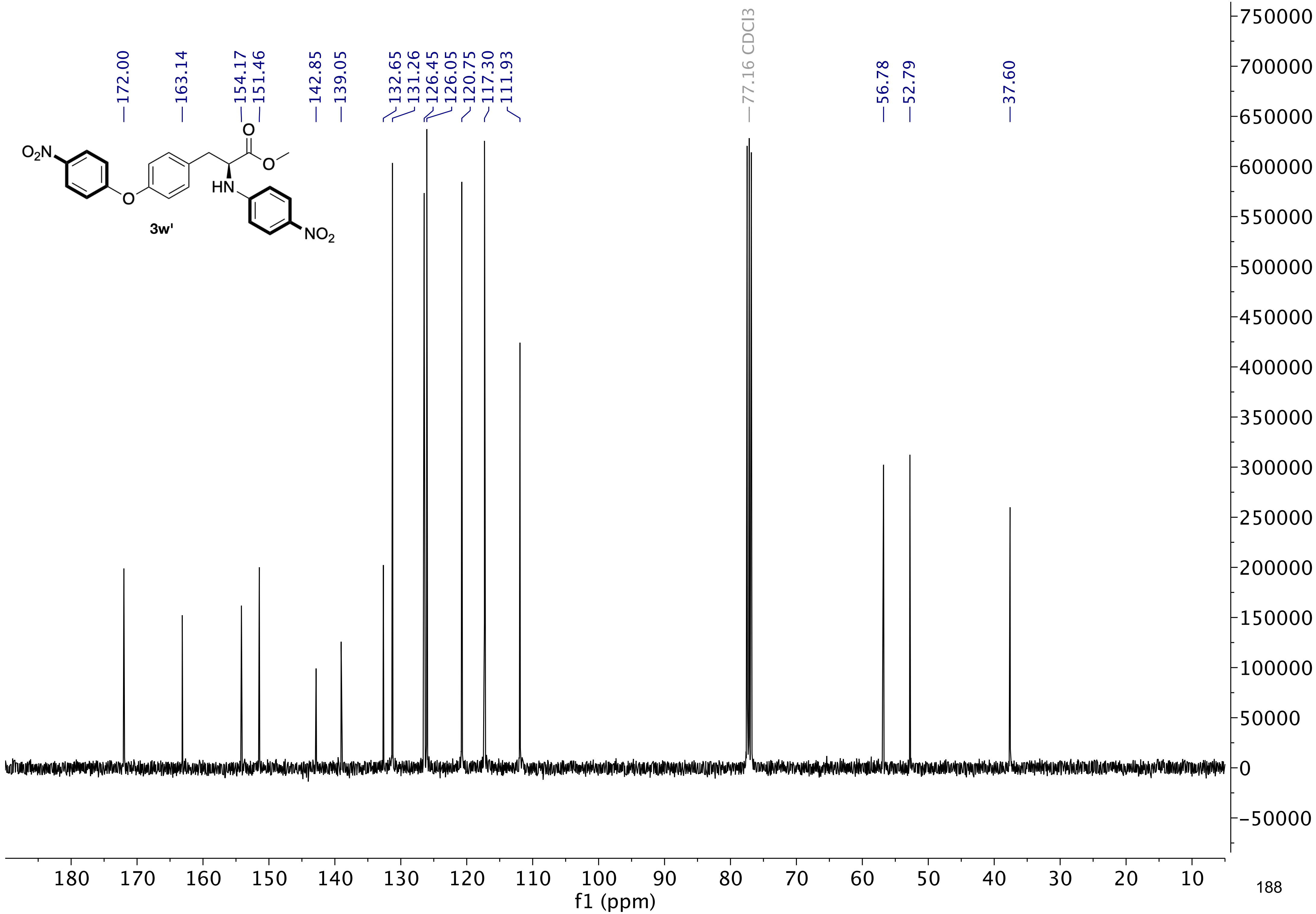

172.00
163.14
154.17
151.46
142.85
139.05
132.65
131.26
126.45
126.05
120.75
117.30
111.93
77.16 CDCl3
56.78
52.79
37.60
O2N
O
O
O
HN
NO2
3w'
f1 (ppm)

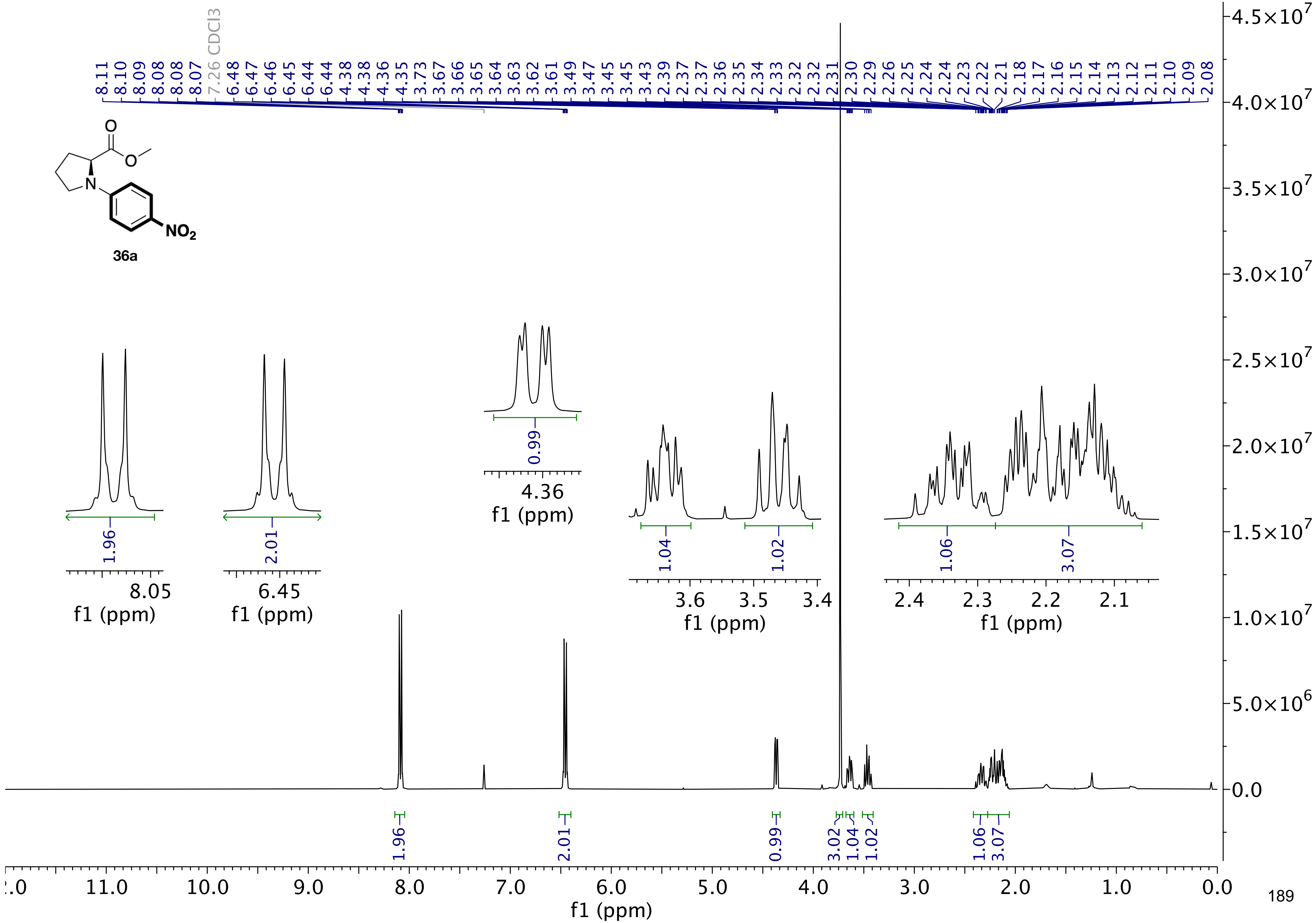
8.11 8.10 8.09 8.08 8.08 8.07 7.26 CDCl3 6.48 6.47 6.46 6.45 6.44 6.44 4.38 4.38 4.36 4.35 3.73 3.67 3.66 3.65 3.64 3.63 3.62 3.61 3.49 3.47 3.45 3.45 3.43 2.39 2.37 2.37 2.36 2.35 2.34 2.33 2.32 2.32 2.31 2.30 2.29 2.26 2.25 2.24 2.24 2.23 2.22 2.21 2.18 2.17 2.16 2.15 2.14 2.13 2.12 2.11 2.10 2.09 2.08
36a
1.96
2.01
0.99
3.02
1.04
1.02
1.06
3.07
f1 (ppm)

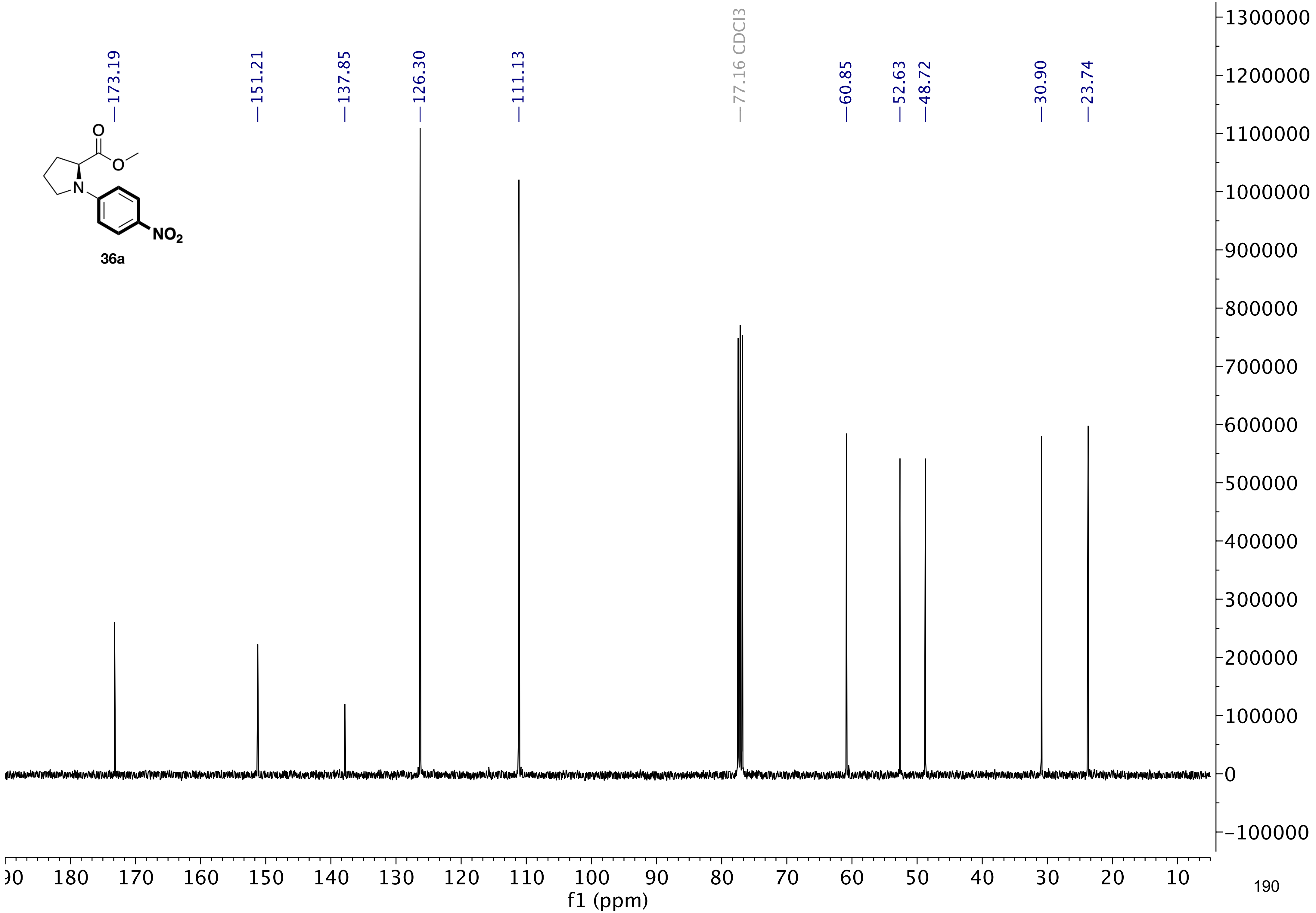
173.19
151.21
137.85
126.30
111.13
77.16 CDCl3
60.85
52.63
48.72
30.90
23.74
O
O
N
NO2
36a
f1 (ppm)

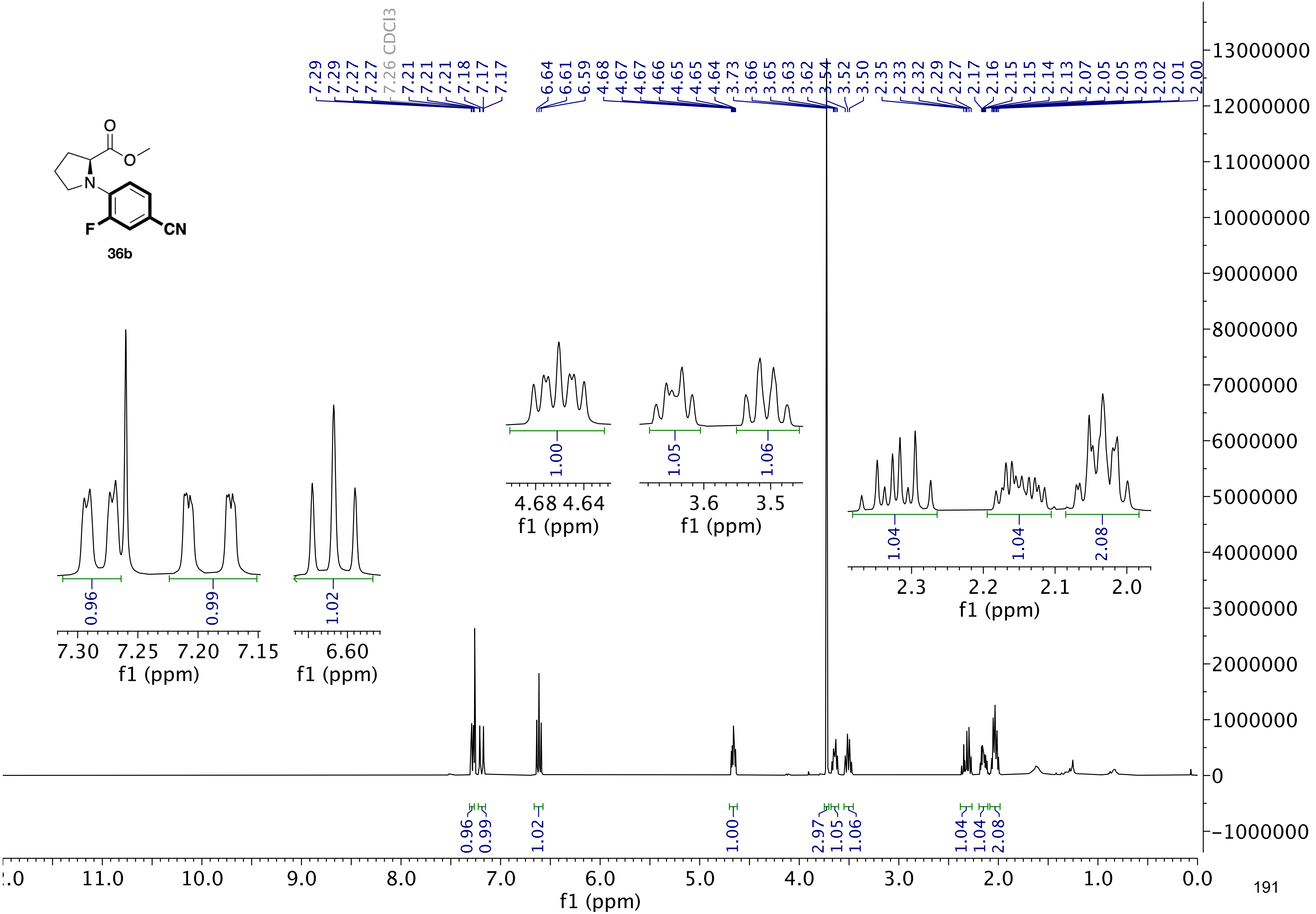

36b
F
CN
N
O
7.29 7.29 7.27 7.27 7.26 CDCl3 7.21 7.21 7.21 7.21 7.18 7.17 7.17
6.64 6.61 6.59
4.68 4.67 4.67 4.66 4.65 4.65 4.64
3.73 3.66 3.65 3.63 3.62 3.54 3.52 3.50
2.35 2.33 2.32 2.29 2.27 2.17 2.16 2.15 2.15 2.14 2.13 2.07 2.05 2.05 2.03 2.02 2.01 2.00
0.96 0.99 1.02 1.00 2.97 1.05 1.06 1.04 1.04 2.08
f1 (ppm)

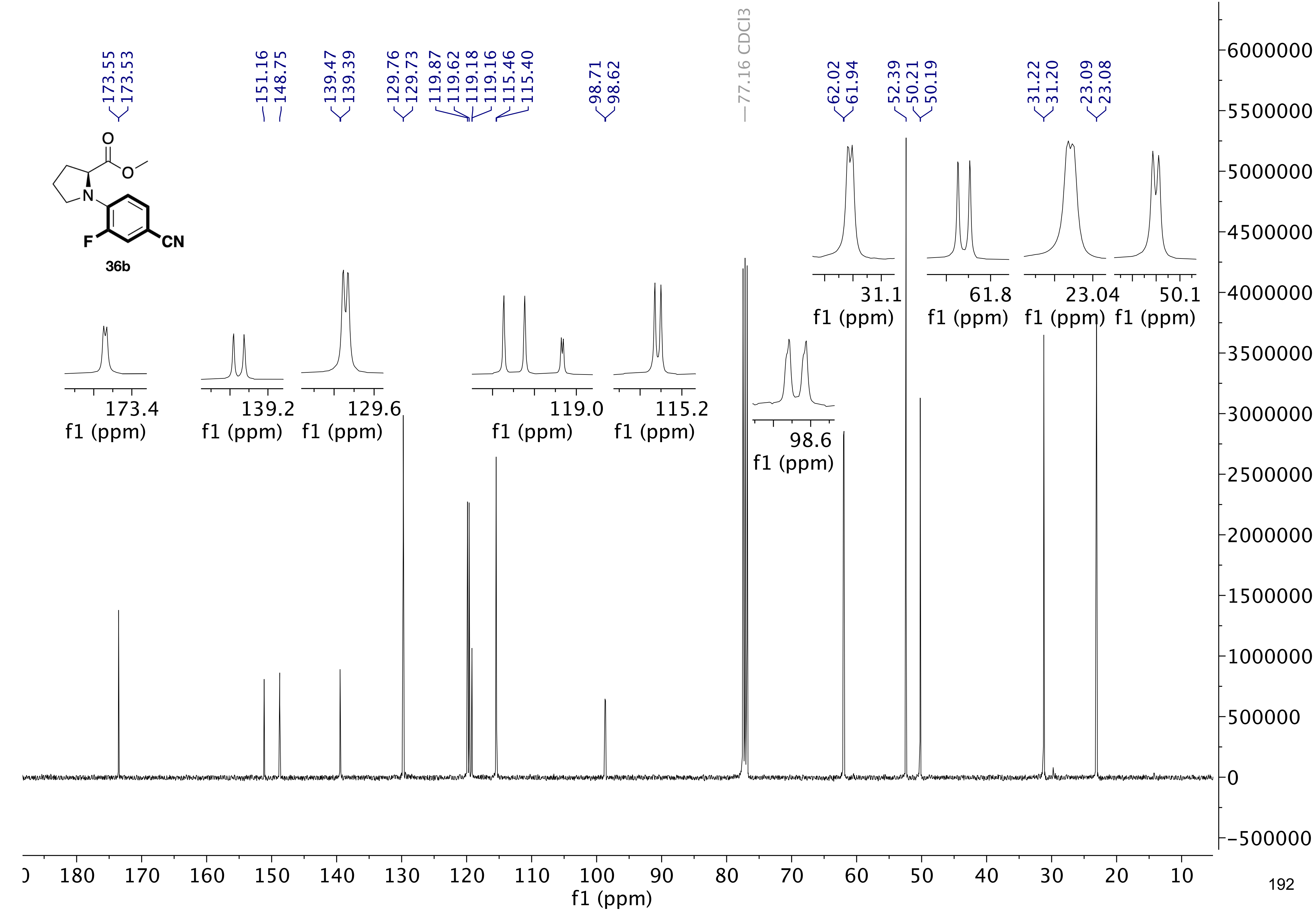
173.55
173.53
151.16
148.75
139.47
139.39
129.76
129.73
119.87
119.62
119.18
119.16
115.46
115.40
98.71
98.62
77.16 CDCl3
62.02
61.94
52.39
50.21
50.19
31.22
31.20
23.09
23.08
O
O
N
F
CN
36b
173.4
139.2
129.6
119.0
115.2
98.6
31.1
61.8
23.04
50.1
f1 (ppm)
6000000
5500000
5000000
4500000
4000000
3500000
3000000
2500000
2000000
1500000
1000000
500000
0
−500000
180
170
160
150
140
130
120
110
100
90
80
70
60
50
40
30
20
10

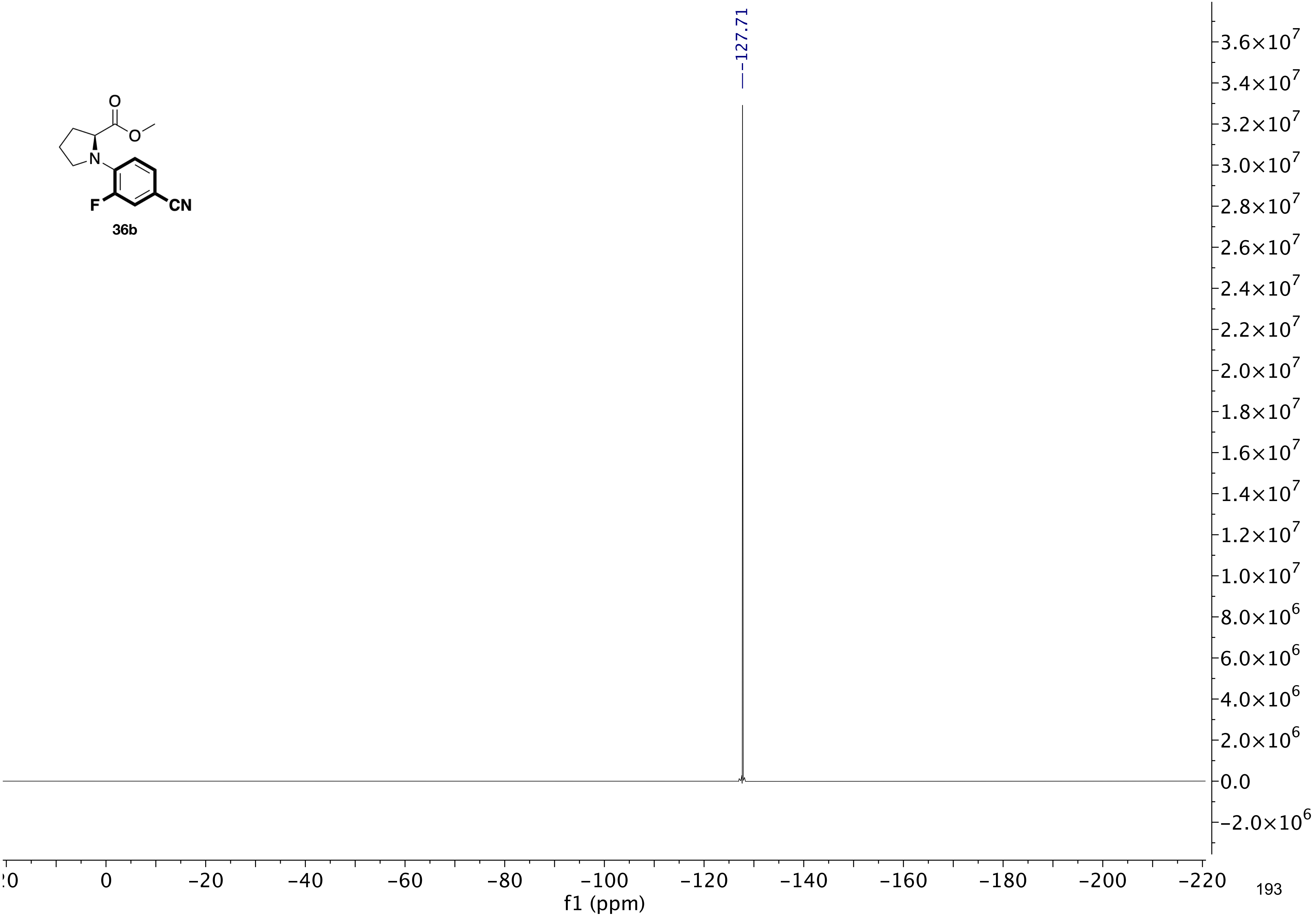

-127.71
O
O
N
F
CN
36b
3.6×10^7
3.4×10^7
3.2×10^7
3.0×10^7
2.8×10^7
2.6×10^7
2.4×10^7
2.2×10^7
2.0×10^7
1.8×10^7
1.6×10^7
1.4×10^7
1.2×10^7
1.0×10^7
8.0×10^6
6.0×10^6
4.0×10^6
2.0×10^6
0.0
-2.0×10^6
0
−20
−40
−60
−80
−100
−120
−140
−160
−180
−200
−220
f1 (ppm)

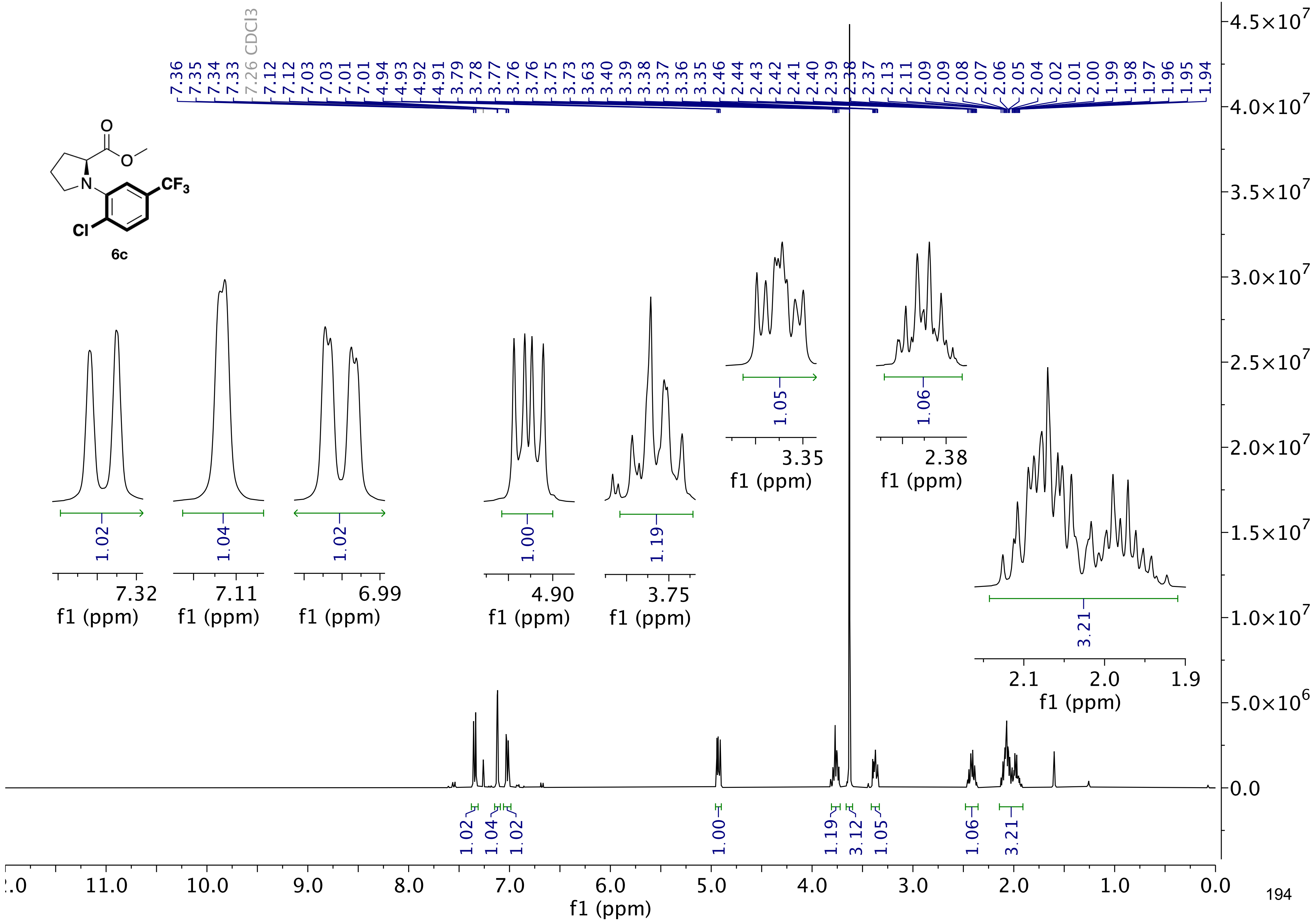

CF3
Cl
N
O
6c
f1 (ppm)

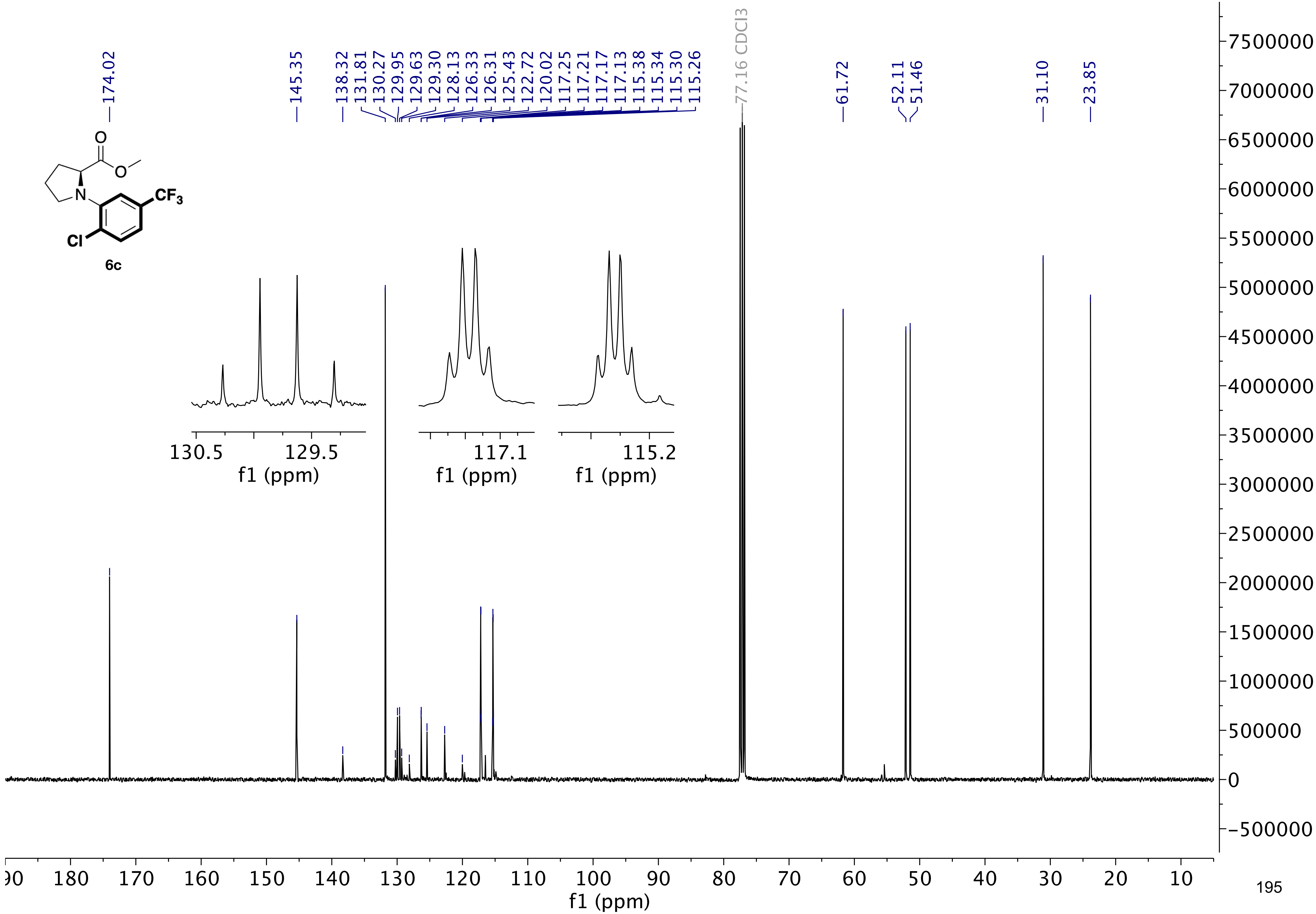
174.02
145.35
138.32
131.81
130.27
129.95
129.63
129.30
128.13
126.33
126.31
125.43
122.72
120.02
117.25
117.21
117.17
117.13
115.38
115.34
115.30
115.26
77.16 CDCl3
61.72
52.11
51.46
31.10
23.85
O
O
N
CF3
Cl
6c
130.5
129.5
f1 (ppm)
117.1
f1 (ppm)
115.2
f1 (ppm)
f1 (ppm)

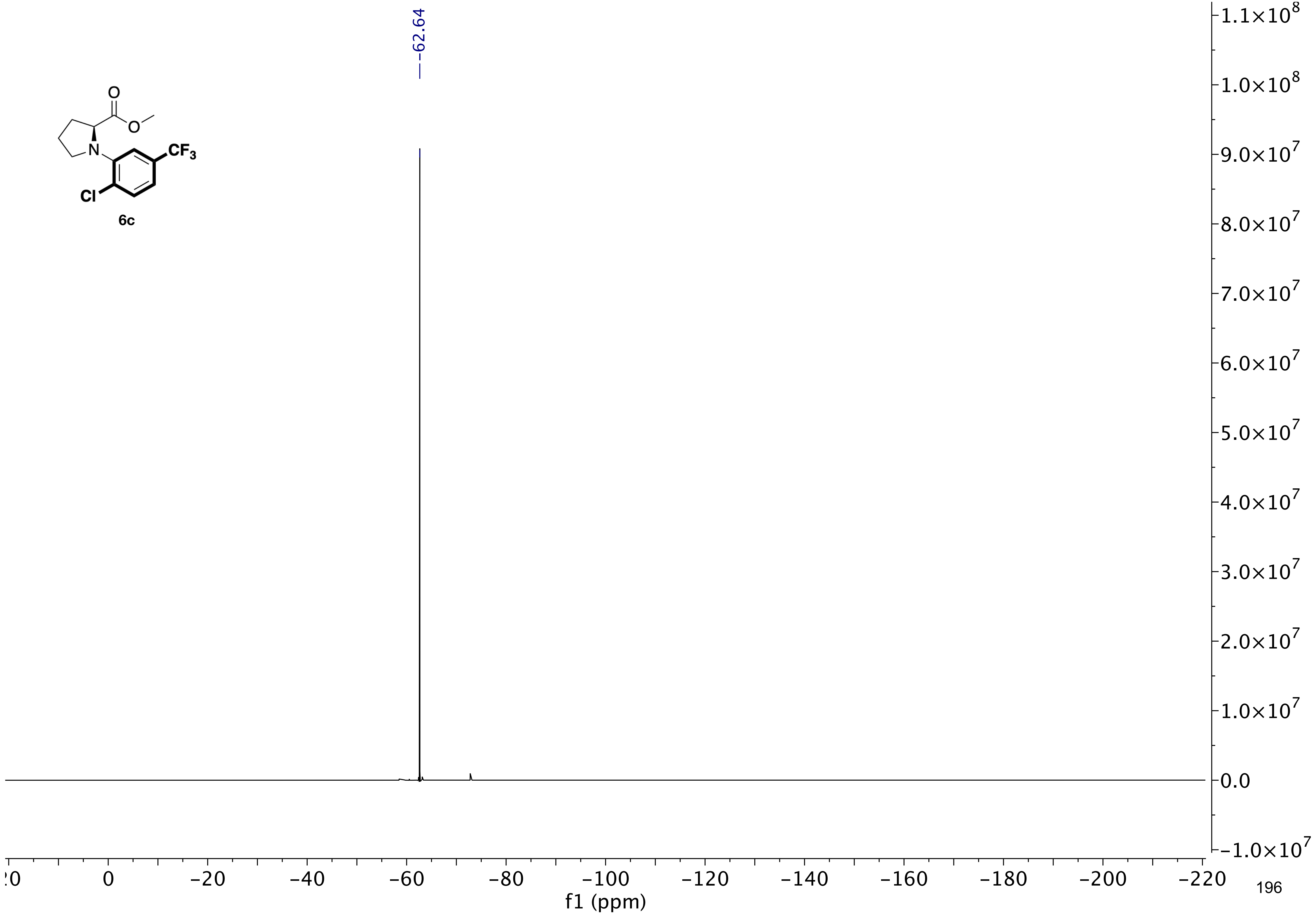

-62.64
O
O
N
CF3
Cl
6c
f1 (ppm)

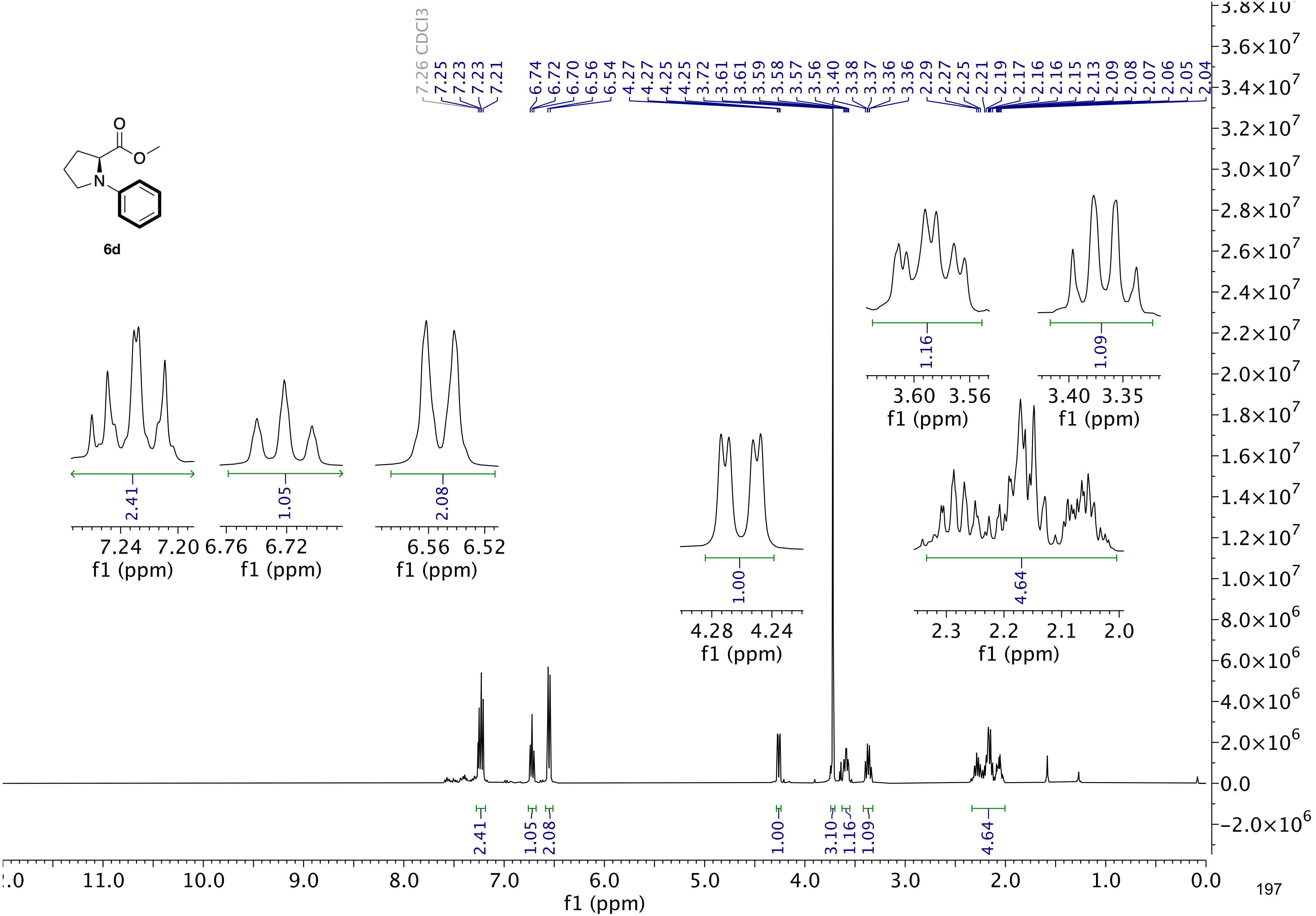

6d

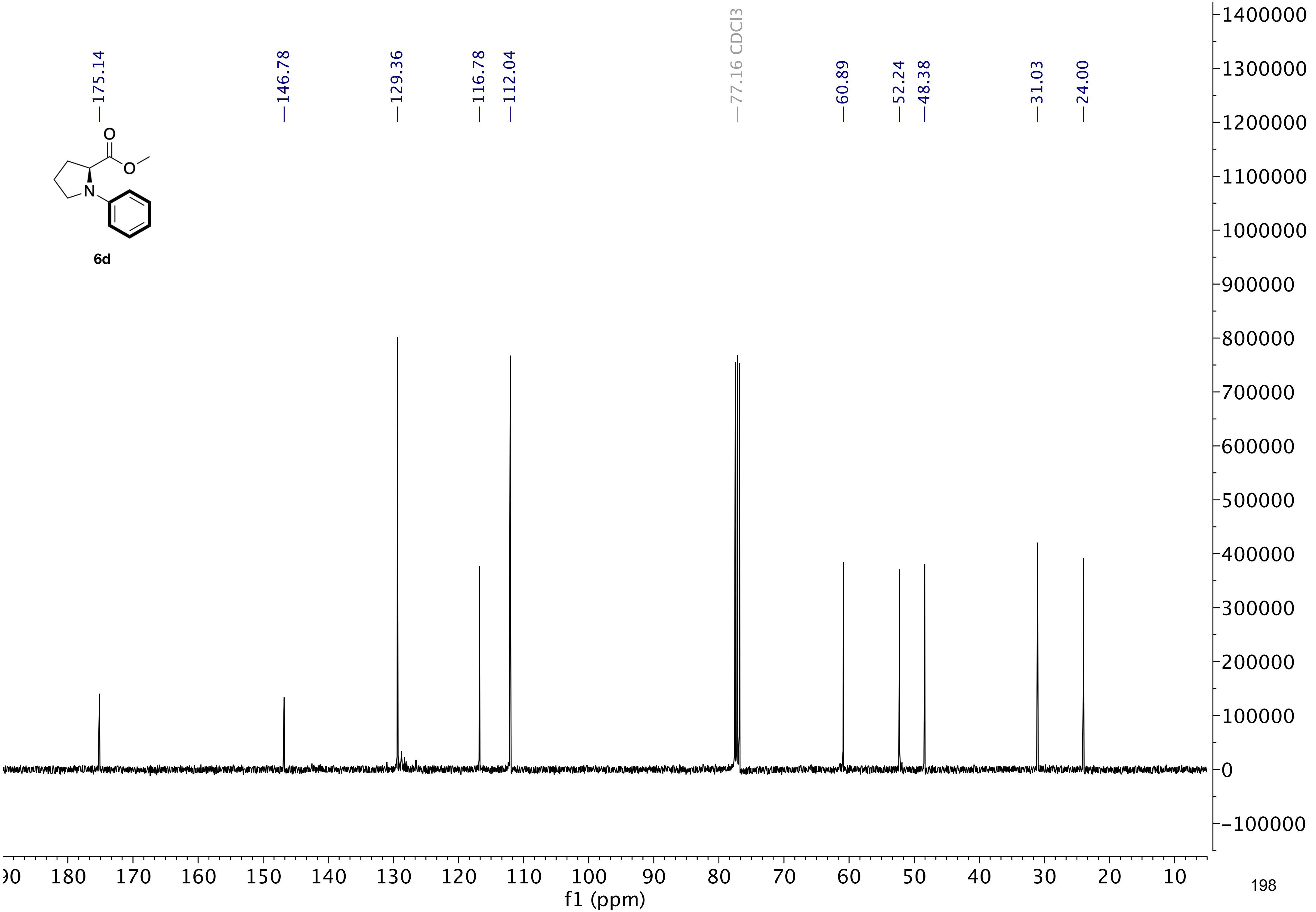
175.14
146.78
129.36
116.78
112.04
77.16 CDCl3
60.89
52.24
48.38
31.03
24.00
6d
f1 (ppm)

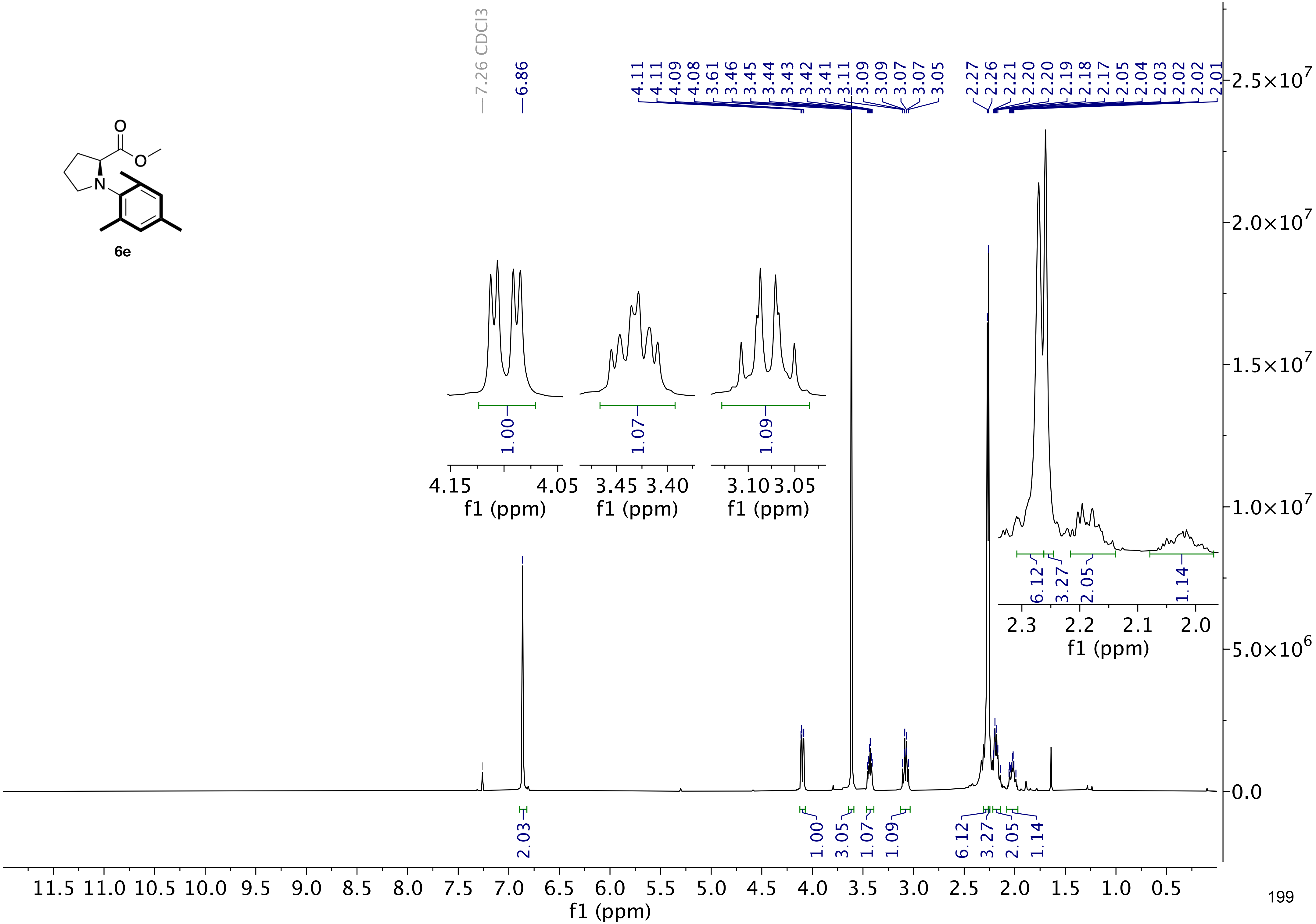
6e
7.26 CDCl3
6.86
4.11 4.11 4.09 4.08 3.61 3.46 3.45 3.44 3.43 3.42 3.41 3.11 3.09 3.09 3.07 3.07 3.05
2.27 2.26 2.21 2.20 2.20 2.19 2.18 2.17 2.05 2.04 2.03 2.02 2.02 2.01
2.03 1.00 3.05 1.07 1.09 6.12 3.27 2.05 1.14
f1 (ppm)

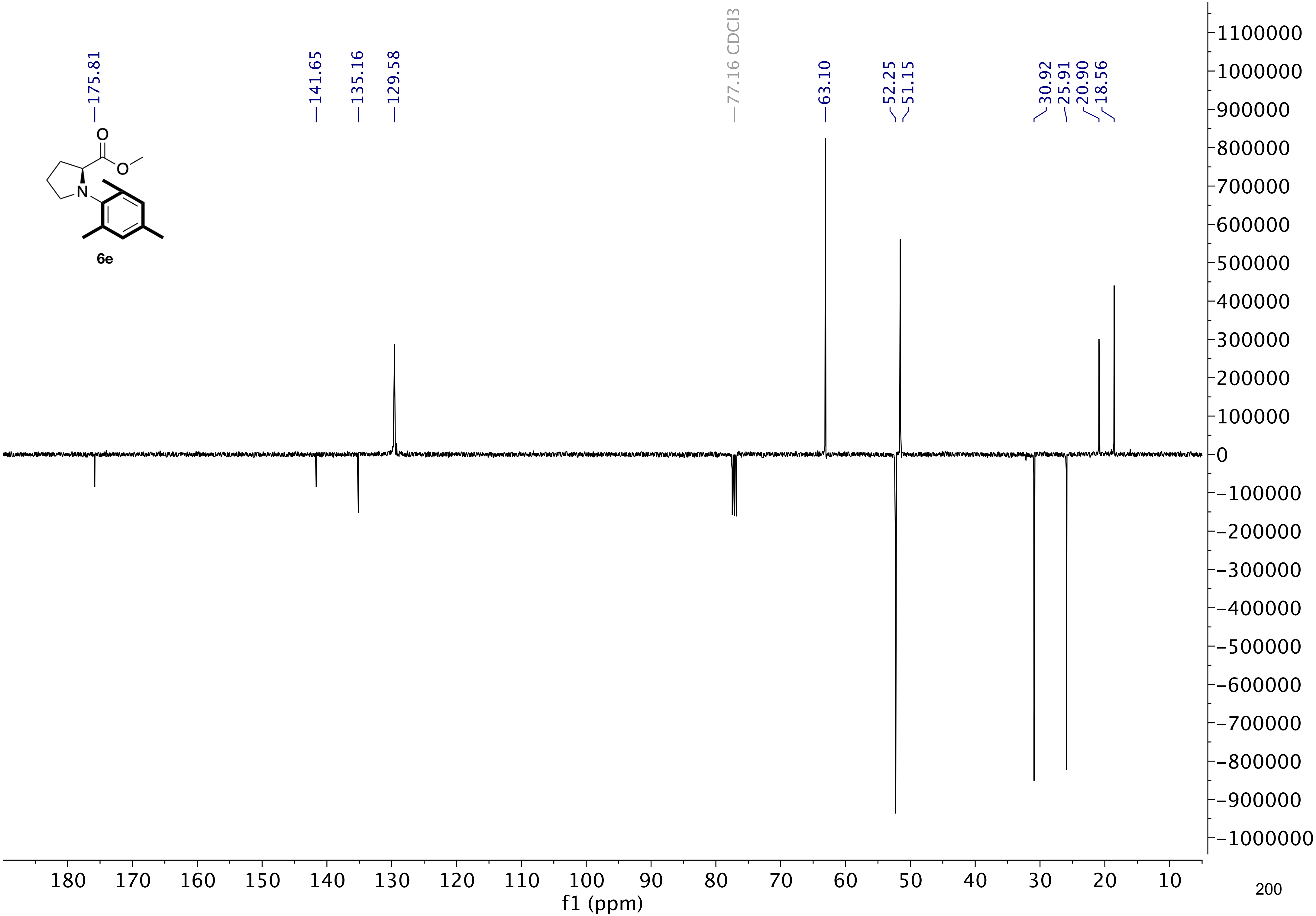

6e
175.81
141.65
135.16
129.58
77.16 CDCl3
63.10
52.25
51.15
30.92
25.91
20.90
18.56
1100000
1000000
900000
800000
700000
600000
500000
400000
300000
200000
100000
0
−100000
−200000
−300000
−400000
−500000
−600000
−700000
−800000
−900000
−1000000
180 170 160 150 140 130 120 110 100 90 80 70 60 50 40 30 20 10
f1 (ppm)

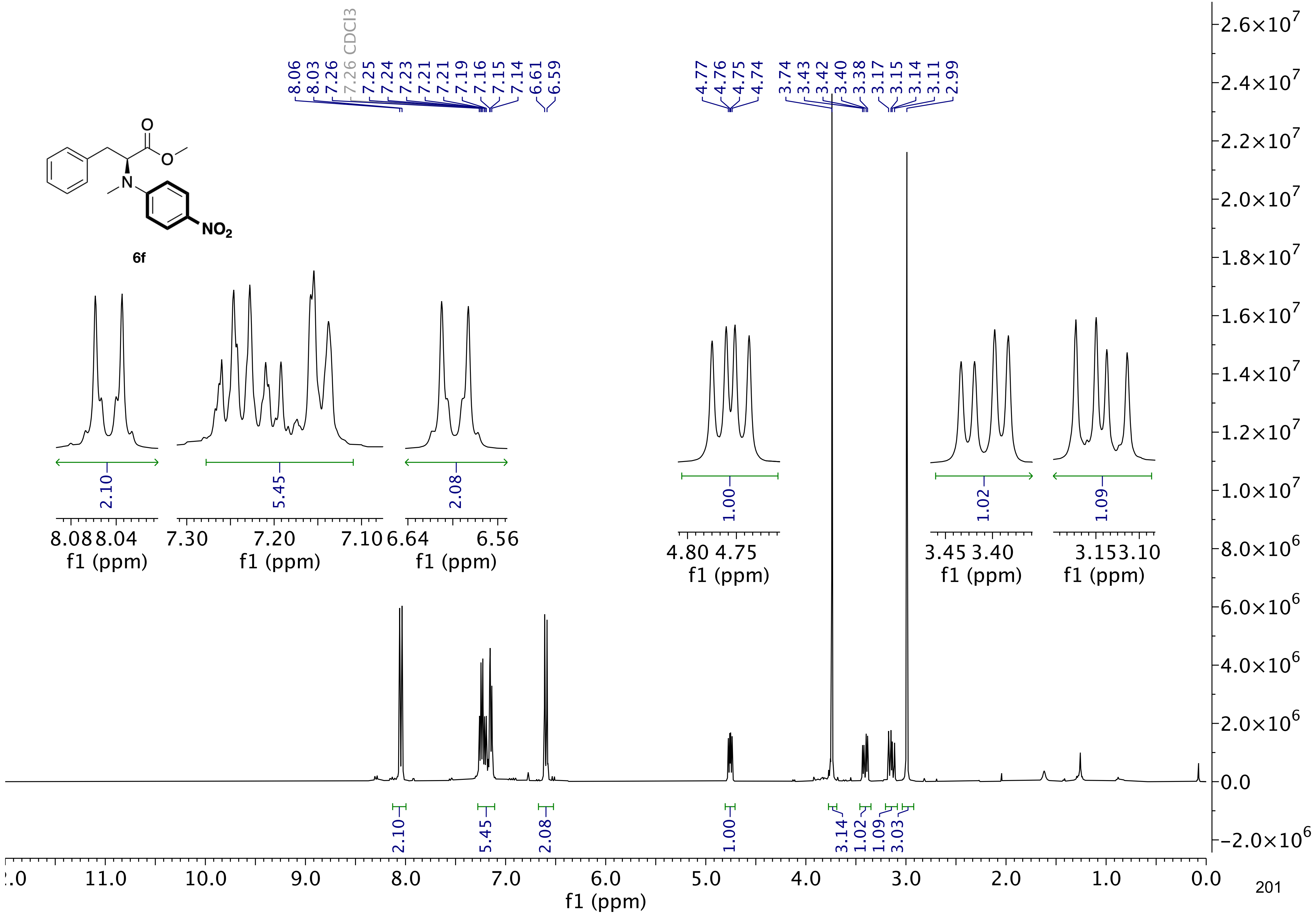

6f
NO2
f1 (ppm)
2.10
5.45
2.08
1.00
1.02
1.09
3.14
3.03
CDCl3

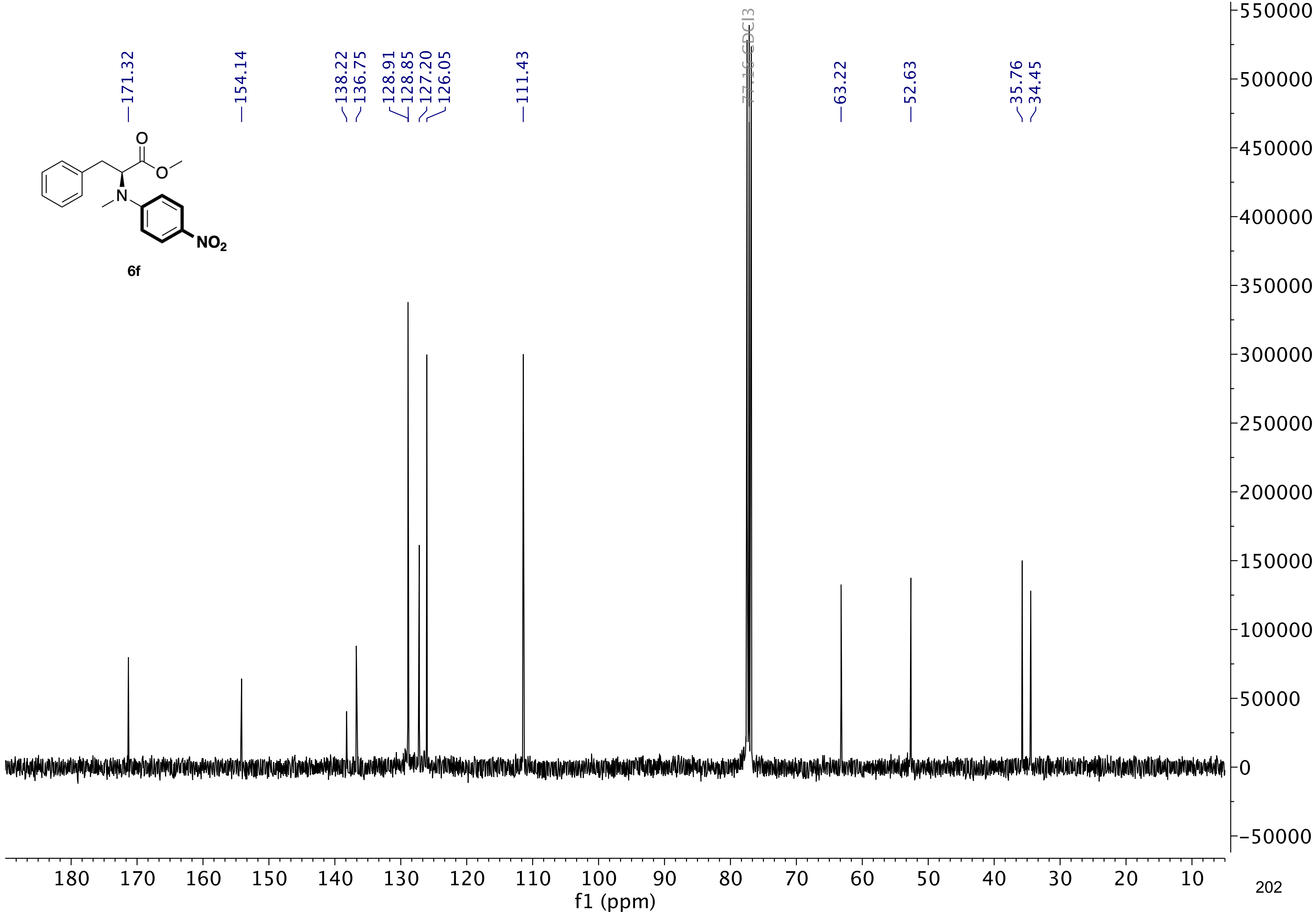
171.32
154.14
138.22
136.75
128.91
128.85
127.20
126.05
111.43
77.16 CDCl3
63.22
52.63
35.76
34.45
NO2
6f
f1 (ppm)
180 170 160 150 140 130 120 110 100 90 80 70 60 50 40 30 20 10
550000
500000
450000
400000
350000
300000
250000
200000
150000
100000
50000
0
−50000

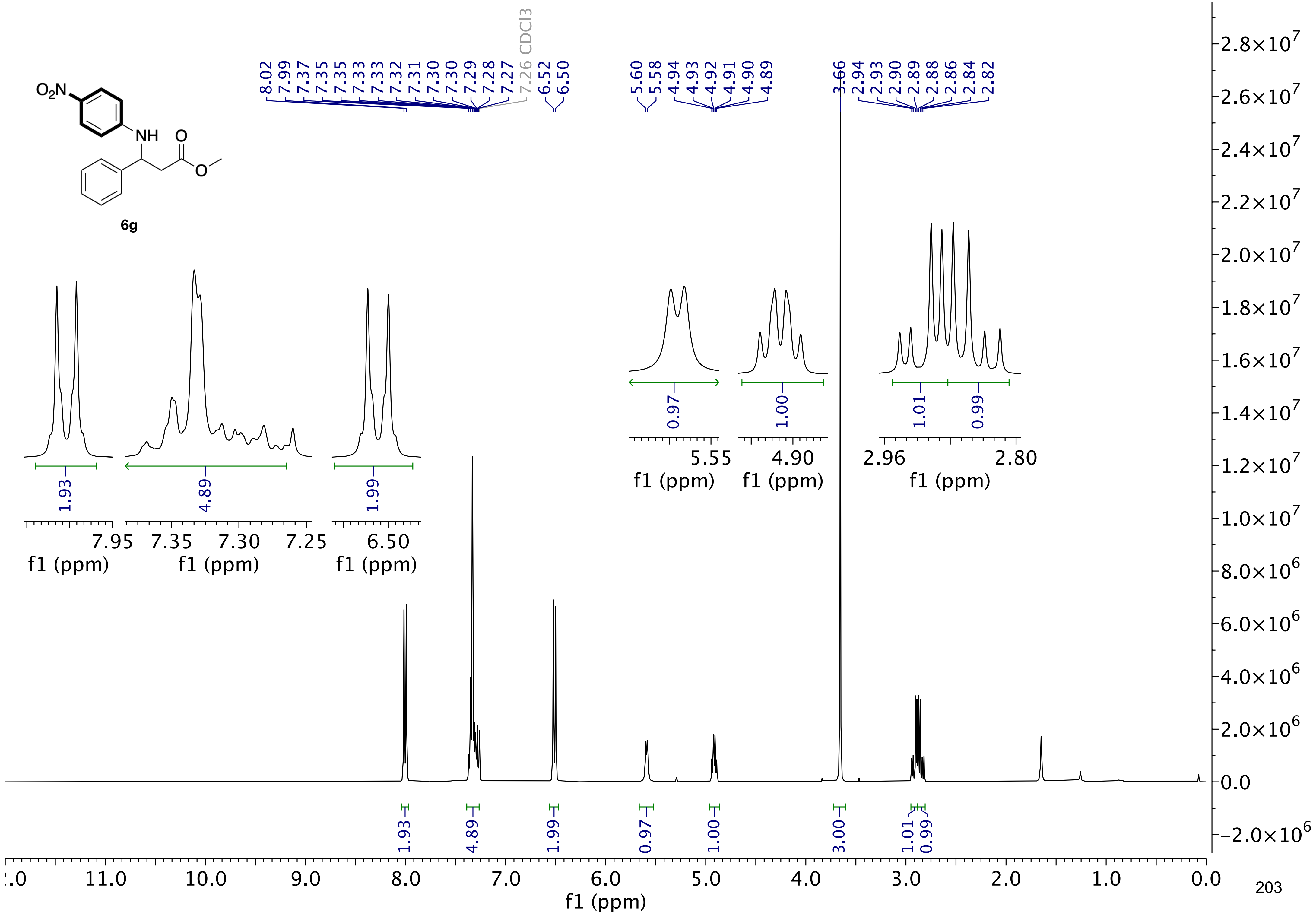

O2N
NH
O
O
6g
8.02 7.99 7.37 7.35 7.35 7.33 7.33 7.33 7.32 7.31 7.30 7.30 7.29 7.28 7.27 7.26 CDCl3 6.52 6.50
5.60 5.58 4.94 4.93 4.92 4.91 4.90 4.89
3.66 2.94 2.93 2.90 2.89 2.88 2.86 2.84 2.82
1.93 4.89 1.99 0.97 1.00 3.00 1.01 0.99
f1 (ppm)

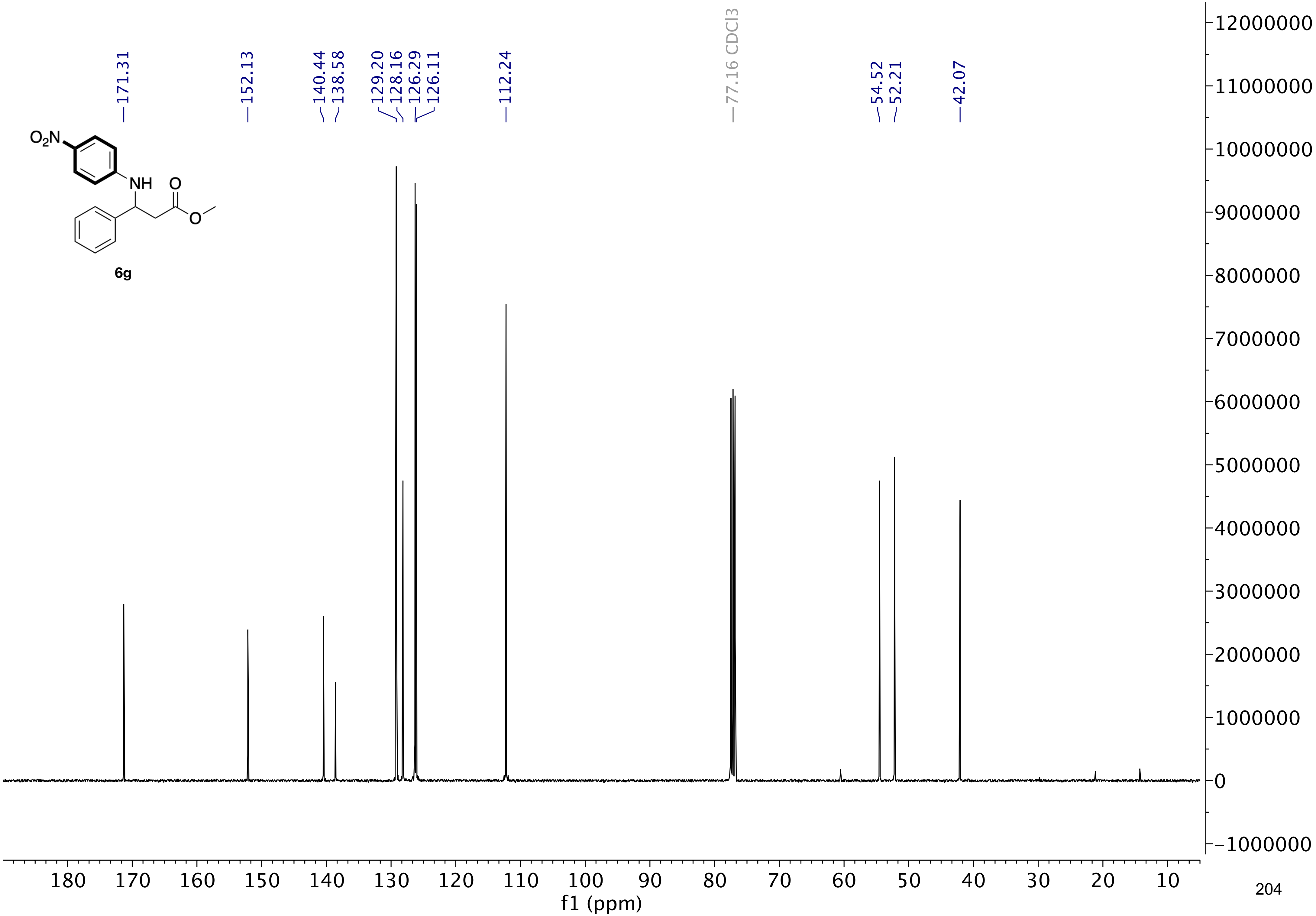
171.31
152.13
140.44
138.58
129.20
128.16
126.29
126.11
112.24
77.16 CDCl3
54.52
52.21
42.07
O2N
NH
O
O
6g
12000000
11000000
10000000
9000000
8000000
7000000
6000000
5000000
4000000
3000000
2000000
1000000
0
−1000000
180 170 160 150 140 130 120 110 100 90 80 70 60 50 40 30 20 10
f1 (ppm)
